# Data set of proteomic analysis of food borne pathogens after treatment with the disinfectants based on pyridoxal oxime derivatives

**DOI:** 10.1016/j.dib.2017.09.060

**Published:** 2017-09-29

**Authors:** Martina Gajdošik Šrajer, Uroš Andjelković, Dajana Gašo-Sokač, Hrvoje Pavlović, Olga Shevchuk, Tamara Martinović, James Clifton, Djuro Josić

**Affiliations:** a Department of Chemistry, J.J. Strossmayer University, HR-31000 Osijek, Croatia; b Department of Biotechnology, University of Rijeka, HR-51000 Rijeka, Croatia; c Faculty of Food Technology, J.J. Strossmayer University, HR-31000 Osijek, Croatia; d Center for Proteomics, Medical School, University of Rijeka, HR-51000 Rijeka, Croatia; e Department of Molecular Pharmacology, Physiology and Biotechnology, Brown University, Providence, RI, USA; f Department of Medicine, Warren Alpert Medical School, Brown University, Providence, RI, USA

## Abstract

Food borne pathogens, namely the Gram-positive bacterium *Bacillus subtilis* and the Gram-negative bacterium *Escherichia coli*, were grown under the inhibition with four different disinfectants based on chloride and bromide salts of pyridinium oxime. Bacterial samples were subjected to the sequential extraction of proteins and the in-solution tryptic digestion of obtained extracts was performed prior to the identification of proteins with LC-ESI-MS/MS. Proteomic analysis identified up- and down-regulated proteins in these bacteria after treatment with each compound. The tables with differently expressed proteins are presented with this article.

**Specifications Table**

**Table:** 

Subject area	*Biochemistry*
More specific subject area	*Proteomics of foodborne bacteria*
Type of data	*Tables, Excel files, Word documents*
How data was acquired	*Tandem mass spectrometry (LC-MS/MS) using LTQ Velos Orbitrap mass spectrometer (Thermo Electron Corp., San Hose, CA, USA)*
Data format	*Filtered and raw*
Experimental factors	*Bacterial cultures of Gram-positive bacterium Bacillus subtilis and Gram-negative bacterium Escherichia coli were treated with four different ammonium salts of pyridinium oxime*
Experimental features	*Sequential extraction of proteins from bacterial samples was performed using ReadyPrep Extraction kit (BioRad, Hercules, CA, USA). Tryptic peptides, obtained by in-solution digestion, were separated on a 12 cmx75 µm I.D. C18 RP column and eluted peptides were analyzed with LTQ Velos Orbitrap mass spectrometer (Thermo Electron Corp., San Hose, CA, USA). Protein quantification was performed using ProteoIQ software with spectra count data.*
Data source location	*Cities of Osijek and Rijeka, Croatia*
Data accessibility	*Filtered data sets are provided as tables with this article*

**Value of the data**

• MS data provide information about alterations in the proteomes of food borne pathogen bacteria *B. subtilis* and *E. coli*, after treatment with newly synthetized quaternary ammonium salts.
• Quantitative gel-free proteomic investigations by LC-ESI MS/MS were used
• Up- and down-regulated proteins in bacterial samples after treatment with four different compounds were identified.
• Protein expression profiles point to the mechanisms of inhibition of used compounds.

## 1 Data

This report contains the complete list of proteins identified in control (not treated) samples of Gram-positive bacterium *Bacillus subtilis* and Gram-negative bacterium *Escherichia coli* (Supplementary 1 and 2).

Proteomic data for each bacteria treated with four different disinfectants based on chloride and bromide salts of pyridinium oxime are also provided in the form of tables containing all up- and- down regulated proteins compared to the control samples (Supplementary 3–18). Data presented in this article is related to the research article [1].

## 2 Experimental design, materials and methods

### 2.1 Stepwise extraction of proteins

Bacterial cultures of Gram-positive bacterium *B. subtilis* and Gram-negative bacterium *E. coli* used for antimicrobial testing were clinical isolates, isolated from patients and identified according to API tests (API® bioMérieux, Marcy l'Etoile, France).

### 2.2 Stepwise extraction of proteins

Bacterial suspensions were centrifuged at 5000 g for 20 min, pellets were re-suspended in extraction buffers and subjected to sequential extraction using the ReadyPrep Extraction kit (BioRad, Hercules, CA, USA) as reported [2]. The procedure consisted of three extraction steps, each giving a separate supernatant (protein extracts 1–3). The protein content in recovered supernatants was determined with the Bicinchoninic Acid (BCA) Protein Assay Kit (Pierce, Rockford, IL, USA), according to the manufacturer's instructions. The amount of protein in the supernatant was adjusted to standard protein loads for an LC-ESI-MS/MS analysis (roughly 20 µg/mL) and subjected to a clean-up via precipitation using a ReadyPrep 2D BioRad kit. All experiments were performed in triplicate.

### 2.3 Identification of proteins with LC-ESI-MS/MS

The “in solution” tryptic digestion of obtained protein extracts was performed as described elsewhere [2]. Tryptic peptides were separated on a 12 cm × 75 µm I.D. C18 RP column (Column Engineering, Ontario, CA, USA) and eluted using a linear gradient starting with 100% solvent A (0.1 M acetic acid in water) to 70% solvent B (0.1 M acetic acid in acetonitrile) over 60 min (Agilent Technologies, Paolo Alto, CA, USA). Eluted peptides were introduced into a LTQ Velos Orbitrap mass spectrometer (Thermo Electron Corp., San Hose, CA, USA) and analyzed as described [2], [3]. Peptide spectrum matching was performed against species-specific databases that were downloaded from NCBI (links from www.ncbi.nlm.nih.gov/Taxonomy/taxonomyhome.html) using Mascot v.2.3. (Matrix Science, Ltd.).

Parameters used in the database search were: fixed modification of cysteines (S-carbamidomethyl), variable modification of methionines (oxidation), two missed cleavages allowed with trypsin, 20 ppm mass tolerance for precursor ions, and 0.5 Da for fragment ions. Concatenated databases with both “target” and decoy sequences were utilized to estimate the false discovery rate (FDR), Protein identifications were derived from the peptide matches using ProteoIQ v.2. 3.08 [4]. To provide high confidence on peptide sequence assignment and protein identification, data were filtered following stringent criteria: Mowse score of more than 28 for all charge states, at least two peptides per protein, 1% peptide and 1% protein FDR. Protein label free quantification (LFQ) was performed by spectral counting of identified MS/MS spectra using ProteoIQ software.
