# Supplementary material for: Data set of proteomic analysis of food borne pathogens after treatment with the disinfectants based on pyridoxal oxime derivatives
Source: Data Brief. 2017 Sep 29;15:738–41. doi: 10.1016/j.dib.2017.09.060 (PMC5671409; doi:10.1016/j.dib.2017.09.060)
Supplement: Supplementary file 1 — Supplementary material [file mmc1.docx]

**List of Supplementary material:**

**Table S1.** Complete list of identified proteins in *B. subtilis* extracts

**Table S2.** Complete list of identified proteins in *E. coli* extracts

To make them easier to navigate in following **Tables S3-S18** complete protein report obtained after analysis was edited by removing multiple names (synonyms) for those proteins that have one or more. Also some additional mathematical analysis information was removed, leaving only the most relevant data regarding this manuscript.

**Table S3.** List of complete down regulated proteins in *B. subtilis* after treatment with Drug 1.

**Table S4.** List of complete down regulated proteins in *B. subtilis* after treatment with Drug 2.

**Table S5.** List of complete down regulated proteins in *B. subtilis* after treatment with Drug 3.

**Table S6.** List of complete down regulated proteins in *B. subtilis* after treatment with Drug 4.

**Table S7.** List of complete up regulated proteins in *B. subtilis* after treatment with Drug 1.

**Table S8.** List of complete up regulated proteins in *B. subtilis* after treatment with Drug 2.

**Table S9.** List of complete up regulated proteins in *B. subtilis* after treatment with Drug 3.

**Table S10.** List of complete up regulated proteins in *B. subtilis* after treatment with Drug 4.

**Table S11.** List of complete down regulated proteins in *E. coli* after treatment with Drug 1.

**Table S12.** List of complete down regulated proteins in *E. coli* after treatment with Drug 2.

**Table S13.** List of complete down regulated proteins in *E. coli* after treatment with Drug 3.

**Table S14.** List of complete down regulated proteins in *E. coli* after treatment with Drug 4.

**Table S15.** List of complete up regulated proteins in *E. coli* after treatment with Drug 1.

**Table S16.** List of complete up regulated proteins in *E. coli* after treatment with Drug 2.

**Table S17.** List of complete up regulated proteins in *E. coli* after treatment with Drug 3.

**Table S18.** List of complete up regulated proteins in *E. coli* after treatment with Drug 4.
