# Supplementary material for: Data set of proteomic analysis of food borne pathogens after treatment with the disinfectants based on pyridoxal oxime derivatives
Source: Data Brief. 2017 Sep 29;15:738–41. doi: 10.1016/j.dib.2017.09.060 (PMC5671409; doi:10.1016/j.dib.2017.09.060)
Supplement: Supplementary file 2 — Supplementary material [file mmc2.docx]

RI NSF/EPSCoR Proteomics Facility

Mass Spectrometry Report

Protein Groups and Peptide-Spectrum Matches (PSMs)

Sample: Bsubtilis 01

Researcher: Djuro Josic

Results filtering: Unique PSMs; MOWSE Score > 0; 5 Proteins/Group max; Proteins from NCBI database

Input PSMs: 2151 'Target'; 1 'Decoy'; 0.0% FDR

Output PSMs: 2151

| **Protein IDs** | | | | | | | |
| --- | --- | --- | --- | --- | --- | --- | --- |
| *Grp Nr.* | *Accession Number* | *Protein Name* | *Protein Score* | *Unique PSMs* | *PSM Serial Nrs.* | *Other Grp.* | *Score (other)* |
| 1 | [255767023](http://www.ncbi.nlm.nih.gov/entrez/query.fcgi?cmd=Search&db=Protein&term=255767023&doptcmdl=GenPept) | DNA-directed RNA polymerase subunit beta' [Bacillus subtilis subsp. subtilis str. 168] | 1228.96 | 28 | 24 203 207 279 316 343 438 460 674 702 899 982 1173 1217 1291 1295 1377 1404 1406 1456 1608 1795 1824 1850 1976 2009 2051 2112 |  |  |
| 2 | [RPOB_BACSU](http://us.expasy.org/uniprot/RPOB_BACSU) | DNA-directed RNA polymerase beta chain (EC 2.7.7.6) (RNAP beta subunit) (Transcriptase beta chain) (RNA polymerase beta subunit) | 1187.47 | 26 | 275 304 354 466 640 662 745 769 877 948 961 1067 1148 1203 1214 1261 1287 1543 1565 1619 1645 1648 1659 1690 1825 1838 |  |  |
|  | [255767022](http://www.ncbi.nlm.nih.gov/entrez/query.fcgi?cmd=Search&db=Protein&term=255767022&doptcmdl=GenPept) | DNA-directed RNA polymerase subunit beta [Bacillus subtilis subsp. subtilis str. 168] | 1129.79 | 25 | 275 304 354 640 662 745 769 877 948 961 1067 1148 1203 1214 1261 1287 1543 1565 1619 1645 1648 1659 1690 1825 1838 |  |  |
| 3 | [16077181](http://www.ncbi.nlm.nih.gov/entrez/query.fcgi?cmd=Search&db=Protein&term=16077181&doptcmdl=GenPept) | elongation factor Tu [Bacillus subtilis subsp. subtilis str. 168] | 1143.63 | 19 | 61 183 195 250 291 322 658 711 741 916 925 1133 1142 1429 1620 1673 1845 1936 2029 |  |  |
| 4 | [255767026](http://www.ncbi.nlm.nih.gov/entrez/query.fcgi?cmd=Search&db=Protein&term=255767026&doptcmdl=GenPept) | elongation factor G [Bacillus subtilis subsp. subtilis str. 168] | 1099.64 | 17 | 80 214 253 311 372 613 707 772 940 1134 1229 1440 1646 1675 1988 2001 2011 |  |  |
| 5 | [16077137](http://www.ncbi.nlm.nih.gov/entrez/query.fcgi?cmd=Search&db=Protein&term=16077137&doptcmdl=GenPept) | cell-division protein and general stress protein [Bacillus subtilis subsp. subtilis str. 168] | 960.81 | 22 | 20 106 134 164 266 339 475 557 576 774 841 854 997 1187 1498 1518 1827 1856 1999 2002 2015 2070 |  |  |
| 6 | [255767218](http://www.ncbi.nlm.nih.gov/entrez/query.fcgi?cmd=Search&db=Protein&term=255767218&doptcmdl=GenPept) | glycerol-3-phosphate oxidase [Bacillus subtilis subsp. subtilis str. 168] | 934.12 | 19 | 94 230 280 332 425 531 569 673 919 999 1055 1127 1212 1231 1484 1610 1652 1697 1970 |  |  |
| 7 | [16077246](http://www.ncbi.nlm.nih.gov/entrez/query.fcgi?cmd=Search&db=Protein&term=16077246&doptcmdl=GenPept) | glucosamine--fructose-6-phosphate aminotransferase [Bacillus subtilis subsp. subtilis str. 168] | 839.75 | 14 | 232 286 656 829 930 1150 1211 1306 1388 1454 1651 1688 1876 1955 |  |  |
| 8 | [971341](http://www.ncbi.nlm.nih.gov/entrez/query.fcgi?cmd=Search&db=Protein&term=971341&doptcmdl=GenPept) | nitrate reductase alpha subunit [Bacillus subtilis] | 798.79 | 16 | 405 537 676 849 1019 1081 1307 1489 1583 1596 1631 1715 1759 1835 1853 2123 |  |  |
|  | [2636265](http://www.ncbi.nlm.nih.gov/entrez/query.fcgi?cmd=Search&db=Protein&term=2636265&doptcmdl=GenPept) | nitrate reductase (alpha subunit) [Bacillus subtilis subsp. subtilis str. 168] | 798.79 | 16 | 405 537 676 849 1019 1081 1307 1489 1583 1596 1631 1715 1759 1835 1853 2123 |  |  |
|  | [255767790](http://www.ncbi.nlm.nih.gov/entrez/query.fcgi?cmd=Search&db=Protein&term=255767790&doptcmdl=GenPept) | nitrate reductase alpha subunit [Bacillus subtilis subsp. subtilis str. 168] | 798.79 | 16 | 405 537 676 849 1019 1081 1307 1489 1583 1596 1631 1715 1759 1835 1853 2123 |  |  |
|  | [2117583](http://www.ncbi.nlm.nih.gov/entrez/query.fcgi?cmd=Search&db=Protein&term=2117583&doptcmdl=GenPept) | nitrate reductase (EC 1.7.99.4) alpha chain narG - Bacillus subtilis | 798.79 | 16 | 405 537 676 849 1019 1081 1307 1489 1583 1596 1631 1715 1759 1835 1853 2123 |  |  |
|  | [16080781](http://www.ncbi.nlm.nih.gov/entrez/query.fcgi?cmd=Search&db=Protein&term=16080781&doptcmdl=GenPept) | nitrate reductase (alpha subunit) [Bacillus subtilis] | 798.79 | 16 | 405 537 676 849 1019 1081 1307 1489 1583 1596 1631 1715 1759 1835 1853 2123 |  |  |
| 9 | [16077736](http://www.ncbi.nlm.nih.gov/entrez/query.fcgi?cmd=Search&db=Protein&term=16077736&doptcmdl=GenPept) | aspartyl/glutamyl-tRNA amidotransferase subunit A [Bacillus subtilis subsp. subtilis str. 168] | 783.97 | 13 | 67 223 452 632 643 1204 1423 1475 1556 1573 1793 2000 2144 |  |  |
| 10 | [16079999](http://www.ncbi.nlm.nih.gov/entrez/query.fcgi?cmd=Search&db=Protein&term=16079999&doptcmdl=GenPept) | acetate kinase [Bacillus subtilis subsp. subtilis str. 168] | 782.00 | 15 | 379 657 759 870 871 975 1001 1003 1179 1260 1473 1532 1893 1939 1944 |  |  |
| 11 | [7434535](http://www.ncbi.nlm.nih.gov/entrez/query.fcgi?cmd=Search&db=Protein&term=7434535&doptcmdl=GenPept) | phosphoglycerate kinase (EC 2.7.2.3) pgk - Bacillus subtilis | 778.71 | 14 | 4 30 489 570 736 752 867 1023 1099 1132 1205 1492 1852 2056 |  |  |
|  | [3123234](http://www.ncbi.nlm.nih.gov/entrez/query.fcgi?cmd=Search&db=Protein&term=3123234&doptcmdl=GenPept) | Phosphoglycerate kinase | 778.71 | 14 | 4 30 489 570 736 752 867 1023 1099 1132 1205 1492 1852 2056 |  |  |
|  | [2635906](http://www.ncbi.nlm.nih.gov/entrez/query.fcgi?cmd=Search&db=Protein&term=2635906&doptcmdl=GenPept) | phosphoglycerate kinase [Bacillus subtilis subsp. subtilis str. 168] | 778.71 | 14 | 4 30 489 570 736 752 867 1023 1099 1132 1205 1492 1852 2056 |  |  |
|  | [16080446](http://www.ncbi.nlm.nih.gov/entrez/query.fcgi?cmd=Search&db=Protein&term=16080446&doptcmdl=GenPept) | phosphoglycerate kinase [Bacillus subtilis] | 778.71 | 14 | 4 30 489 570 736 752 867 1023 1099 1132 1205 1492 1852 2056 |  |  |
| 12 | [7436878](http://www.ncbi.nlm.nih.gov/entrez/query.fcgi?cmd=Search&db=Protein&term=7436878&doptcmdl=GenPept) | phosphopyruvate hydratase (EC 4.2.1.11) [validated] - Bacillus subtilis | 765.19 | 13 | 222 272 380 498 796 1227 1265 1383 1655 1667 1676 1870 2114 |  |  |
|  | [6166147](http://www.ncbi.nlm.nih.gov/entrez/query.fcgi?cmd=Search&db=Protein&term=6166147&doptcmdl=GenPept) | Enolase (2-phosphoglycerate dehydratase) (2-phospho-D-glycerate hydro-lyase) | 765.19 | 13 | 222 272 380 498 796 1227 1265 1383 1655 1667 1676 1870 2114 |  |  |
|  | [2635903](http://www.ncbi.nlm.nih.gov/entrez/query.fcgi?cmd=Search&db=Protein&term=2635903&doptcmdl=GenPept) | enolase [Bacillus subtilis subsp. subtilis str. 168] | 765.19 | 13 | 222 272 380 498 796 1227 1265 1383 1655 1667 1676 1870 2114 |  |  |
|  | [16080443](http://www.ncbi.nlm.nih.gov/entrez/query.fcgi?cmd=Search&db=Protein&term=16080443&doptcmdl=GenPept) | enolase [Bacillus subtilis] | 765.19 | 13 | 222 272 380 498 796 1227 1265 1383 1655 1667 1676 1870 2114 |  |  |
| 13 | [16078672](http://www.ncbi.nlm.nih.gov/entrez/query.fcgi?cmd=Search&db=Protein&term=16078672&doptcmdl=GenPept) | succinyl-CoA synthetase subunit beta [Bacillus subtilis subsp. subtilis str. 168] | 713.17 | 13 | 348 454 529 555 626 697 1006 1035 1183 1290 1293 1722 1826 |  |  |
| 14 | [16079954](http://www.ncbi.nlm.nih.gov/entrez/query.fcgi?cmd=Search&db=Protein&term=16079954&doptcmdl=GenPept) | glyceraldehyde-3-phosphate dehydrogenase [Bacillus subtilis subsp. subtilis str. 168] | 710.66 | 13 | 192 301 479 480 714 724 771 823 952 1037 1161 1470 1861 |  |  |
| 15 | [7437039](http://www.ncbi.nlm.nih.gov/entrez/query.fcgi?cmd=Search&db=Protein&term=7437039&doptcmdl=GenPept) | aconitate hydratase (EC 4.2.1.3) citB - Bacillus subtilis | 705.42 | 13 | 176 441 645 830 935 965 998 1082 1343 1422 1441 1892 1922 |  |  |
|  | [2634184](http://www.ncbi.nlm.nih.gov/entrez/query.fcgi?cmd=Search&db=Protein&term=2634184&doptcmdl=GenPept) | aconitate hydratase [Bacillus subtilis subsp. subtilis str. 168] | 705.42 | 13 | 176 441 645 830 935 965 998 1082 1343 1422 1441 1892 1922 |  |  |
|  | [2506131](http://www.ncbi.nlm.nih.gov/entrez/query.fcgi?cmd=Search&db=Protein&term=2506131&doptcmdl=GenPept) | Aconitate hydratase (Citrate hydro-lyase) (Aconitase) | 705.42 | 13 | 176 441 645 830 935 965 998 1082 1343 1422 1441 1892 1922 |  |  |
|  | [16078863](http://www.ncbi.nlm.nih.gov/entrez/query.fcgi?cmd=Search&db=Protein&term=16078863&doptcmdl=GenPept) | aconitate hydratase [Bacillus subtilis] | 705.42 | 13 | 176 441 645 830 935 965 998 1082 1343 1422 1441 1892 1922 |  |  |
|  | [1405454](http://www.ncbi.nlm.nih.gov/entrez/query.fcgi?cmd=Search&db=Protein&term=1405454&doptcmdl=GenPept) | aconitase [Bacillus subtilis] | 705.42 | 13 | 176 441 645 830 935 965 998 1082 1343 1422 1441 1892 1922 |  |  |
| 16 | [16078801](http://www.ncbi.nlm.nih.gov/entrez/query.fcgi?cmd=Search&db=Protein&term=16078801&doptcmdl=GenPept) | ribonucleotide-diphosphate reductase subunit alpha [Bacillus subtilis subsp. subtilis str. 168] | 678.70 | 13 | 181 327 409 858 946 960 1002 1202 1207 1358 1367 1372 1586 |  |  |
| 17 | [255767204](http://www.ncbi.nlm.nih.gov/entrez/query.fcgi?cmd=Search&db=Protein&term=255767204&doptcmdl=GenPept) | vegetative catalase 1 [Bacillus subtilis subsp. subtilis str. 168] | 676.80 | 15 | 43 330 341 368 791 956 1047 1124 1159 1228 1312 1463 1512 1529 1566 |  |  |
| 18 | [16077670](http://www.ncbi.nlm.nih.gov/entrez/query.fcgi?cmd=Search&db=Protein&term=16077670&doptcmdl=GenPept) | chaperonin GroEL [Bacillus subtilis subsp. subtilis str. 168] | 654.05 | 12 | 293 362 402 434 443 592 601 806 832 941 1455 1539 |  |  |
| 19 | [16077119](http://www.ncbi.nlm.nih.gov/entrez/query.fcgi?cmd=Search&db=Protein&term=16077119&doptcmdl=GenPept) | ribose-phosphate pyrophosphokinase [Bacillus subtilis subsp. subtilis str. 168] | 652.43 | 12 | 18 550 693 725 761 862 1256 1348 1417 1625 1794 1906 |  |  |
| 20 | [255767656](http://www.ncbi.nlm.nih.gov/entrez/query.fcgi?cmd=Search&db=Protein&term=255767656&doptcmdl=GenPept) | succinate dehydrogenase flavoprotein subunit [Bacillus subtilis subsp. subtilis str. 168] | 637.18 | 13 | 197 262 777 859 890 1086 1165 1451 1476 1545 1635 2041 2063 |  |  |
| 21 | [16077729](http://www.ncbi.nlm.nih.gov/entrez/query.fcgi?cmd=Search&db=Protein&term=16077729&doptcmdl=GenPept) | ATP-dependent DNA helicase [Bacillus subtilis subsp. subtilis str. 168] | 634.99 | 13 | 58 742 818 838 1014 1041 1122 1130 1400 1472 1633 1868 2140 |  |  |
| 22 | [729813](http://www.ncbi.nlm.nih.gov/entrez/query.fcgi?cmd=Search&db=Protein&term=729813&doptcmdl=GenPept) | Isocitrate dehydrogenase [NADP] (Oxalosuccinate decarboxylase) (IDH) (NADP+-specific ICDH) (IDP) | 613.84 | 14 | 637 684 831 865 1000 1135 1162 1281 1357 1361 1628 1837 1857 2046 |  |  |
|  | [487434](http://www.ncbi.nlm.nih.gov/entrez/query.fcgi?cmd=Search&db=Protein&term=487434&doptcmdl=GenPept) | isocitrate dehydrogenase | 613.84 | 14 | 637 684 831 865 1000 1135 1162 1281 1357 1361 1628 1837 1857 2046 |  |  |
|  | [2635378](http://www.ncbi.nlm.nih.gov/entrez/query.fcgi?cmd=Search&db=Protein&term=2635378&doptcmdl=GenPept) | isocitrate dehydrogenase [Bacillus subtilis subsp. subtilis str. 168] | 613.84 | 14 | 637 684 831 865 1000 1135 1162 1281 1357 1361 1628 1837 1857 2046 |  |  |
|  | [2293268](http://www.ncbi.nlm.nih.gov/entrez/query.fcgi?cmd=Search&db=Protein&term=2293268&doptcmdl=GenPept) | isocitrate dehydrogenase [Bacillus subtilis] | 613.84 | 14 | 637 684 831 865 1000 1135 1162 1281 1357 1361 1628 1837 1857 2046 |  |  |
|  | [2117469](http://www.ncbi.nlm.nih.gov/entrez/query.fcgi?cmd=Search&db=Protein&term=2117469&doptcmdl=GenPept) | isocitrate dehydrogenase (NADP) (EC 1.1.1.42) - Bacillus subtilis | 613.84 | 14 | 637 684 831 865 1000 1135 1162 1281 1357 1361 1628 1837 1857 2046 |  |  |
| 23 | [16080447](http://www.ncbi.nlm.nih.gov/entrez/query.fcgi?cmd=Search&db=Protein&term=16080447&doptcmdl=GenPept) | glyceraldehyde-3-phosphate dehydrogenase [Bacillus subtilis subsp. subtilis str. 168] | 611.50 | 10 | 215 298 715 753 1215 1368 1424 1589 1616 1702 |  |  |
| 24 | [50812307](http://www.ncbi.nlm.nih.gov/entrez/query.fcgi?cmd=Search&db=Protein&term=50812307&doptcmdl=GenPept) | pyrimidine-nucleoside phosphorylase [Bacillus subtilis subsp. subtilis str. 168] | 609.30 | 9 | 194 317 325 559 790 1131 1272 1380 1530 |  |  |
| 25 | [16077399](http://www.ncbi.nlm.nih.gov/entrez/query.fcgi?cmd=Search&db=Protein&term=16077399&doptcmdl=GenPept) | assimilatory nitrite reductase subunit [Bacillus subtilis subsp. subtilis str. 168] | 599.83 | 12 | 25 281 439 523 581 664 1069 1365 1509 1696 1771 1804 |  |  |
| 26 | [16078078](http://www.ncbi.nlm.nih.gov/entrez/query.fcgi?cmd=Search&db=Protein&term=16078078&doptcmdl=GenPept) | protoporphyrinogen oxidase [Bacillus subtilis subsp. subtilis str. 168] | 593.50 | 9 | 149 211 228 874 1413 1504 1777 1969 1991 |  |  |
| 27 | [16078726](http://www.ncbi.nlm.nih.gov/entrez/query.fcgi?cmd=Search&db=Protein&term=16078726&doptcmdl=GenPept) | translation initiation factor IF-2 [Bacillus subtilis subsp. subtilis str. 168] | 585.11 | 10 | 133 169 403 472 908 1072 1194 1305 1311 1562 |  |  |
| 28 | [16080736](http://www.ncbi.nlm.nih.gov/entrez/query.fcgi?cmd=Search&db=Protein&term=16080736&doptcmdl=GenPept) | F0F1 ATP synthase subunit alpha [Bacillus subtilis subsp. subtilis str. 168] | 579.18 | 13 | 406 431 659 744 906 1025 1434 1526 1632 1711 1820 1997 2027 |  |  |
| 29 | [16080789](http://www.ncbi.nlm.nih.gov/entrez/query.fcgi?cmd=Search&db=Protein&term=16080789&doptcmdl=GenPept) | antilisterial bacteriocin (subtilosin) production enzyme [Bacillus subtilis subsp. subtilis str. 168] | 574.41 | 12 | 87 190 589 628 775 1060 1443 1665 1965 1983 2010 2053 |  |  |
| 30 | [16080743](http://www.ncbi.nlm.nih.gov/entrez/query.fcgi?cmd=Search&db=Protein&term=16080743&doptcmdl=GenPept) | serine hydroxymethyltransferase [Bacillus subtilis subsp. subtilis str. 168] | 567.78 | 9 | 177 584 814 992 1112 1196 1245 1373 1921 |  |  |
| 31 | [255767789](http://www.ncbi.nlm.nih.gov/entrez/query.fcgi?cmd=Search&db=Protein&term=255767789&doptcmdl=GenPept) | translaldolase [Bacillus subtilis subsp. subtilis str. 168] | 560.13 | 12 | 240 271 335 383 490 521 1012 1029 1375 1981 2026 2149 |  |  |
| 32 | [16077201](http://www.ncbi.nlm.nih.gov/entrez/query.fcgi?cmd=Search&db=Protein&term=16077201&doptcmdl=GenPept) | 30S ribosomal protein S5 [Bacillus subtilis subsp. subtilis str. 168] | 557.04 | 11 | 3 285 467 758 799 928 1063 1216 1270 1569 1836 |  |  |
| 33 | [16078541](http://www.ncbi.nlm.nih.gov/entrez/query.fcgi?cmd=Search&db=Protein&term=16078541&doptcmdl=GenPept) | GTPase [Bacillus subtilis subsp. subtilis str. 168] | 541.81 | 11 | 535 805 932 994 1197 1243 1277 1328 1394 2127 2129 |  |  |
| 34 | [16077077](http://www.ncbi.nlm.nih.gov/entrez/query.fcgi?cmd=Search&db=Protein&term=16077077&doptcmdl=GenPept) | inosine 5'-monophosphate dehydrogenase [Bacillus subtilis subsp. subtilis str. 168] | 536.67 | 11 | 113 150 420 688 706 733 802 813 1984 2087 2097 |  |  |
| 35 | [16080583](http://www.ncbi.nlm.nih.gov/entrez/query.fcgi?cmd=Search&db=Protein&term=16080583&doptcmdl=GenPept) | preprotein translocase subunit SecA [Bacillus subtilis subsp. subtilis str. 168] | 531.80 | 12 | 168 398 408 428 430 528 699 778 968 1501 2118 2133 |  |  |
| 36 | [16080334](http://www.ncbi.nlm.nih.gov/entrez/query.fcgi?cmd=Search&db=Protein&term=16080334&doptcmdl=GenPept) | acyl-CoA dehydrogenase [Bacillus subtilis subsp. subtilis str. 168] | 498.08 | 12 | 224 564 620 671 1200 1230 1269 1363 1534 1568 1855 2125 |  |  |
| 37 | [255767389](http://www.ncbi.nlm.nih.gov/entrez/query.fcgi?cmd=Search&db=Protein&term=255767389&doptcmdl=GenPept) | recombinase A [Bacillus subtilis subsp. subtilis str. 168] | 497.14 | 9 | 187 451 532 801 920 973 1746 2057 2093 |  |  |
| 38 | [16078550](http://www.ncbi.nlm.nih.gov/entrez/query.fcgi?cmd=Search&db=Protein&term=16078550&doptcmdl=GenPept) | pyruvate carboxylase [Bacillus subtilis subsp. subtilis str. 168] | 496.39 | 10 | 515 602 682 936 1247 1567 1609 1841 2143 2148 |  |  |
| 39 | [16078712](http://www.ncbi.nlm.nih.gov/entrez/query.fcgi?cmd=Search&db=Protein&term=16078712&doptcmdl=GenPept) | 30S ribosomal protein S2 [Bacillus subtilis subsp. subtilis str. 168] | 491.10 | 8 | 129 141 456 654 735 1285 1829 1912 |  |  |
| 40 | [255767648](http://www.ncbi.nlm.nih.gov/entrez/query.fcgi?cmd=Search&db=Protein&term=255767648&doptcmdl=GenPept) | ATP-dependent protease ATP-binding subunit ClpX [Bacillus subtilis subsp. subtilis str. 168] | 487.58 | 10 | 746 800 811 847 1020 1255 1319 1558 1958 2104 |  |  |
| 41 | [50812194](http://www.ncbi.nlm.nih.gov/entrez/query.fcgi?cmd=Search&db=Protein&term=50812194&doptcmdl=GenPept) | aspartyl/glutamyl-tRNA amidotransferase subunit B [Bacillus subtilis subsp. subtilis str. 168] | 485.03 | 8 | 955 995 1106 1199 1242 1493 1683 1695 |  |  |
| 42 | [16077691](http://www.ncbi.nlm.nih.gov/entrez/query.fcgi?cmd=Search&db=Protein&term=16077691&doptcmdl=GenPept) | acetoin reductase/2,3-butanediol dehydrogenase [Bacillus subtilis subsp. subtilis str. 168] | 473.30 | 9 | 216 235 237 284 770 1191 1384 2065 2081 |  |  |
| 43 | [255767698](http://www.ncbi.nlm.nih.gov/entrez/query.fcgi?cmd=Search&db=Protein&term=255767698&doptcmdl=GenPept) | phosphoenolpyruvate carboxykinase [Bacillus subtilis subsp. subtilis str. 168] | 466.06 | 7 | 96 359 954 1297 1694 1700 2033 |  |  |
| 44 | [16080734](http://www.ncbi.nlm.nih.gov/entrez/query.fcgi?cmd=Search&db=Protein&term=16080734&doptcmdl=GenPept) | F0F1 ATP synthase subunit beta [Bacillus subtilis subsp. subtilis str. 168] | 464.28 | 8 | 173 913 1115 1181 1275 1592 1881 1985 |  |  |
| 45 | [50812298](http://www.ncbi.nlm.nih.gov/entrez/query.fcgi?cmd=Search&db=Protein&term=50812298&doptcmdl=GenPept) | rod shape-determining protein Mbl [Bacillus subtilis subsp. subtilis str. 168] | 460.07 | 10 | 344 352 577 786 888 927 1330 1352 1516 1817 |  |  |
| 46 | [16080107](http://www.ncbi.nlm.nih.gov/entrez/query.fcgi?cmd=Search&db=Protein&term=16080107&doptcmdl=GenPept) | S-adenosylmethionine synthetase [Bacillus subtilis subsp. subtilis str. 168] | 457.90 | 9 | 138 333 486 598 608 987 1163 1170 2131 |  |  |
| 47 | [16080768](http://www.ncbi.nlm.nih.gov/entrez/query.fcgi?cmd=Search&db=Protein&term=16080768&doptcmdl=GenPept) | CTP synthetase [Bacillus subtilis subsp. subtilis str. 168] | 456.28 | 9 | 157 605 1062 1457 1499 1571 1678 1691 2007 |  |  |
| 48 | [ENGD_BACSU](http://us.expasy.org/uniprot/ENGD_BACSU) | GTP-dependent nucleic acid-binding protein engD | 450.70 | 9 | 16 202 273 397 503 541 586 1559 1901 |  |  |
|  | [16081144](http://www.ncbi.nlm.nih.gov/entrez/query.fcgi?cmd=Search&db=Protein&term=16081144&doptcmdl=GenPept) | GTP-dependent nucleic acid-binding protein EngD [Bacillus subtilis subsp. subtilis str. 168] | 392.96 | 8 | 16 202 273 397 503 541 586 1901 |  |  |
| 49 | [16078517](http://www.ncbi.nlm.nih.gov/entrez/query.fcgi?cmd=Search&db=Protein&term=16078517&doptcmdl=GenPept) | ribonuclease J1 [Bacillus subtilis subsp. subtilis str. 168] | 440.22 | 10 | 74 377 450 514 939 1149 1371 1415 1601 2091 |  |  |
| 50 | [255767494](http://www.ncbi.nlm.nih.gov/entrez/query.fcgi?cmd=Search&db=Protein&term=255767494&doptcmdl=GenPept) | cryptic glutamate dehydrogenase [Bacillus subtilis subsp. subtilis str. 168] | 438.68 | 8 | 338 540 670 889 1362 1449 1553 2064 |  |  |
| 51 | [16078764](http://www.ncbi.nlm.nih.gov/entrez/query.fcgi?cmd=Search&db=Protein&term=16078764&doptcmdl=GenPept) | (dimethylallyl)adenosine tRNA methylthiotransferase [Bacillus subtilis subsp. subtilis str. 168] | 437.94 | 10 | 386 544 846 876 972 1083 1087 1496 1770 1789 |  |  |
| 52 | [16079872](http://www.ncbi.nlm.nih.gov/entrez/query.fcgi?cmd=Search&db=Protein&term=16079872&doptcmdl=GenPept) | class III heat-shock ATP-dependent LonA protease [Bacillus subtilis subsp. subtilis str. 168] | 437.76 | 11 | 71 370 787 1048 1053 1094 1237 1268 1786 1803 2105 |  |  |
| 53 | [255767030](http://www.ncbi.nlm.nih.gov/entrez/query.fcgi?cmd=Search&db=Protein&term=255767030&doptcmdl=GenPept) | 30S ribosomal protein S3 [Bacillus subtilis subsp. subtilis str. 168] | 437.15 | 7 | 864 1011 1017 1235 1239 1513 1594 |  |  |
| 54 | [16078511](http://www.ncbi.nlm.nih.gov/entrez/query.fcgi?cmd=Search&db=Protein&term=16078511&doptcmdl=GenPept) | rod-share determining protein MreBH [Bacillus subtilis subsp. subtilis str. 168] | 435.45 | 8 | 504 687 1206 1420 1520 1732 2076 2151 |  |  |
| 55 | [16080013](http://www.ncbi.nlm.nih.gov/entrez/query.fcgi?cmd=Search&db=Protein&term=16080013&doptcmdl=GenPept) | septation ring formation regulator EzrA [Bacillus subtilis subsp. subtilis str. 168] | 418.48 | 8 | 259 435 470 950 953 1544 1992 2147 |  |  |
| 56 | [16078522](http://www.ncbi.nlm.nih.gov/entrez/query.fcgi?cmd=Search&db=Protein&term=16078522&doptcmdl=GenPept) | pyruvate dehydrogenase (E1 alpha subunit) [Bacillus subtilis subsp. subtilis str. 168] | 416.73 | 9 | 246 302 824 1049 1241 1460 1640 2023 2086 |  |  |
| 57 | [16078809](http://www.ncbi.nlm.nih.gov/entrez/query.fcgi?cmd=Search&db=Protein&term=16078809&doptcmdl=GenPept) | glutamine synthetase [Bacillus subtilis subsp. subtilis str. 168] | 414.91 | 10 | 357 462 633 708 788 1647 1726 1760 1761 2132 |  |  |
| 58 | [255767778](http://www.ncbi.nlm.nih.gov/entrez/query.fcgi?cmd=Search&db=Protein&term=255767778&doptcmdl=GenPept) | acetolactate synthase [Bacillus subtilis subsp. subtilis str. 168] | 412.81 | 7 | 62 931 1332 1557 1563 1626 1894 |  |  |
| 59 | [16080027](http://www.ncbi.nlm.nih.gov/entrez/query.fcgi?cmd=Search&db=Protein&term=16080027&doptcmdl=GenPept) | bifunctional 3-deoxy-7-phosphoheptulonate synthase/chorismate mutase [Bacillus subtilis subsp. subtilis str. 168] | 402.93 | 6 | 14 64 401 1193 1598 1755 |  |  |
| 60 | [255767020](http://www.ncbi.nlm.nih.gov/entrez/query.fcgi?cmd=Search&db=Protein&term=255767020&doptcmdl=GenPept) | 50S ribosomal protein L1 [Bacillus subtilis subsp. subtilis str. 168] | 392.60 | 7 | 360 437 509 717 754 1575 1754 |  |  |
| 61 | [16078991](http://www.ncbi.nlm.nih.gov/entrez/query.fcgi?cmd=Search&db=Protein&term=16078991&doptcmdl=GenPept) | aldehyde dehydrogenase [Bacillus subtilis subsp. subtilis str. 168] | 390.96 | 9 | 536 538 607 785 807 850 1079 1497 1548 |  |  |
| 62 | [16080602](http://www.ncbi.nlm.nih.gov/entrez/query.fcgi?cmd=Search&db=Protein&term=16080602&doptcmdl=GenPept) | two-component response regulator [Bacillus subtilis subsp. subtilis str. 168] | 389.85 | 8 | 329 404 625 652 690 1119 1728 1729 |  |  |
| 63 | [16079864](http://www.ncbi.nlm.nih.gov/entrez/query.fcgi?cmd=Search&db=Protein&term=16079864&doptcmdl=GenPept) | glutamate-1-semialdehyde aminotransferase [Bacillus subtilis subsp. subtilis str. 168] | 385.24 | 9 | 393 597 765 768 1084 1379 1593 1614 1986 |  |  |
| 64 | [16080314](http://www.ncbi.nlm.nih.gov/entrez/query.fcgi?cmd=Search&db=Protein&term=16080314&doptcmdl=GenPept) | fructoselysine-6-P-deglycase [Bacillus subtilis subsp. subtilis str. 168] | 384.32 | 5 | 143 855 1506 1574 2037 |  |  |
| 65 | [16077154](http://www.ncbi.nlm.nih.gov/entrez/query.fcgi?cmd=Search&db=Protein&term=16077154&doptcmdl=GenPept) | class III stress response-related ATPase [Bacillus subtilis subsp. subtilis str. 168] | 383.61 | 8 | 461 837 967 1240 1258 1263 1396 1603 |  |  |
| 66 | [16079605](http://www.ncbi.nlm.nih.gov/entrez/query.fcgi?cmd=Search&db=Protein&term=16079605&doptcmdl=GenPept) | GTP-binding protein LepA [Bacillus subtilis subsp. subtilis str. 168] | 381.38 | 6 | 231 743 1622 1731 1758 2094 |  |  |
| 67 | [16078616](http://www.ncbi.nlm.nih.gov/entrez/query.fcgi?cmd=Search&db=Protein&term=16078616&doptcmdl=GenPept) | carbamoyl phosphate synthase large subunit [Bacillus subtilis subsp. subtilis str. 168] | 380.68 | 9 | 13 500 1412 1636 1735 1791 1810 1928 1951 |  |  |
| 68 | [16081148](http://www.ncbi.nlm.nih.gov/entrez/query.fcgi?cmd=Search&db=Protein&term=16081148&doptcmdl=GenPept) | site-specific DNA-binding protein [Bacillus subtilis subsp. subtilis str. 168] | 375.83 | 9 | 188 366 647 839 1447 1570 1698 1725 1866 |  |  |
| 69 | [16078720](http://www.ncbi.nlm.nih.gov/entrez/query.fcgi?cmd=Search&db=Protein&term=16078720&doptcmdl=GenPept) | prolyl-tRNA synthetase [Bacillus subtilis subsp. subtilis str. 168] | 371.29 | 8 | 356 511 1056 1152 1662 1718 1895 2117 |  |  |
| 70 | [16080620](http://www.ncbi.nlm.nih.gov/entrez/query.fcgi?cmd=Search&db=Protein&term=16080620&doptcmdl=GenPept) | UTP-glucose-1-phosphate uridylyltransferase [Bacillus subtilis subsp. subtilis str. 168] | 366.18 | 9 | 261 636 666 875 943 1008 1104 1226 1249 |  |  |
| 71 | [16080729](http://www.ncbi.nlm.nih.gov/entrez/query.fcgi?cmd=Search&db=Protein&term=16080729&doptcmdl=GenPept) | UDP-N-acetylglucosamine 1-carboxyvinyltransferase [Bacillus subtilis subsp. subtilis str. 168] | 359.33 | 6 | 83 103 978 1100 1822 1874 |  |  |
| 72 | [255767378](http://www.ncbi.nlm.nih.gov/entrez/query.fcgi?cmd=Search&db=Protein&term=255767378&doptcmdl=GenPept) | ribonuclease J2 [Bacillus subtilis subsp. subtilis str. 168] | 356.85 | 8 | 179 289 779 1390 1398 1414 1416 1546 |  |  |
| 73 | [98440](http://www.ncbi.nlm.nih.gov/entrez/query.fcgi?cmd=Search&db=Protein&term=98440&doptcmdl=GenPept) | dihydrolipoamide dehydrogenase (EC 1.8.1.4) - Bacillus subtilis | 356.44 | 7 | 861 902 1158 1515 1537 1744 2074 |  |  |
|  | [3282145](http://www.ncbi.nlm.nih.gov/entrez/query.fcgi?cmd=Search&db=Protein&term=3282145&doptcmdl=GenPept) | dihydrolipoamide dehydrogenase E3 [Bacillus subtilis] | 356.44 | 7 | 861 902 1158 1515 1537 1744 2074 |  |  |
|  | [2633832](http://www.ncbi.nlm.nih.gov/entrez/query.fcgi?cmd=Search&db=Protein&term=2633832&doptcmdl=GenPept) | dihydrolipoamide dehydrogenase E3 subunit of both pyruvate dehydrogenase and 2-oxoglutarate dehydrogenase complexes [Bacillus subtilis subsp. subtilis str. 168] | 356.44 | 7 | 861 902 1158 1515 1537 1744 2074 |  |  |
|  | [16078525](http://www.ncbi.nlm.nih.gov/entrez/query.fcgi?cmd=Search&db=Protein&term=16078525&doptcmdl=GenPept) | dihydrolipoamide dehydrogenase E3 subunit of both pyruvate dehydrogenase and 2-oxoglutarate dehydrogenase complexes [Bacillus subtilis] | 356.44 | 7 | 861 902 1158 1515 1537 1744 2074 |  |  |
|  | [143380](http://www.ncbi.nlm.nih.gov/entrez/query.fcgi?cmd=Search&db=Protein&term=143380&doptcmdl=GenPept) | dihydrolipoamide dehydrogenase E3 subunit | 356.44 | 7 | 861 902 1158 1515 1537 1744 2074 |  |  |
| 74 | [16079851](http://www.ncbi.nlm.nih.gov/entrez/query.fcgi?cmd=Search&db=Protein&term=16079851&doptcmdl=GenPept) | ATPase activator of MinC [Bacillus subtilis subsp. subtilis str. 168] | 351.34 | 6 | 1071 1252 1681 1900 1909 2096 |  |  |
| 75 | [16080336](http://www.ncbi.nlm.nih.gov/entrez/query.fcgi?cmd=Search&db=Protein&term=16080336&doptcmdl=GenPept) | enoyl-CoA hydratase / 3-hydroxyacyl-CoA dehydrogenase [Bacillus subtilis subsp. subtilis str. 168] | 351.13 | 7 | 90 844 1109 1713 1784 1884 1904 |  |  |
| 76 | [16079455](http://www.ncbi.nlm.nih.gov/entrez/query.fcgi?cmd=Search&db=Protein&term=16079455&doptcmdl=GenPept) | hypothetical protein BSU23990 [Bacillus subtilis subsp. subtilis str. 168] | 350.00 | 6 | 274 621 905 1234 1654 2048 |  |  |
| 77 | [16079971](http://www.ncbi.nlm.nih.gov/entrez/query.fcgi?cmd=Search&db=Protein&term=16079971&doptcmdl=GenPept) | 6-phosphofructokinase [Bacillus subtilis subsp. subtilis str. 168] | 348.73 | 6 | 8 415 513 843 1360 1459 |  |  |
| 78 | [16080319](http://www.ncbi.nlm.nih.gov/entrez/query.fcgi?cmd=Search&db=Protein&term=16080319&doptcmdl=GenPept) | FeS cluster formation protein [Bacillus subtilis subsp. subtilis str. 168] | 347.75 | 6 | 7 162 649 1195 1719 1720 |  |  |
| 79 | [16080018](http://www.ncbi.nlm.nih.gov/entrez/query.fcgi?cmd=Search&db=Protein&term=16080018&doptcmdl=GenPept) | 30S ribosomal protein S4 [Bacillus subtilis subsp. subtilis str. 168] | 345.57 | 8 | 238 416 481 622 635 884 1073 2116 |  |  |
| 80 | [16080196](http://www.ncbi.nlm.nih.gov/entrez/query.fcgi?cmd=Search&db=Protein&term=16080196&doptcmdl=GenPept) | C-S lyase [Bacillus subtilis subsp. subtilis str. 168] | 344.54 | 4 | 152 883 1477 2102 |  |  |
| 81 | [16078350](http://www.ncbi.nlm.nih.gov/entrez/query.fcgi?cmd=Search&db=Protein&term=16078350&doptcmdl=GenPept) | Pit accessory protein [Bacillus subtilis subsp. subtilis str. 168] | 336.28 | 8 | 241 254 1005 1151 1253 1911 1957 1960 |  |  |
| 82 | [16077150](http://www.ncbi.nlm.nih.gov/entrez/query.fcgi?cmd=Search&db=Protein&term=16077150&doptcmdl=GenPept) | lysyl-tRNA synthetase [Bacillus subtilis subsp. subtilis str. 168] | 334.58 | 7 | 527 789 1174 1450 1522 1599 2017 |  |  |
| 83 | [16080106](http://www.ncbi.nlm.nih.gov/entrez/query.fcgi?cmd=Search&db=Protein&term=16080106&doptcmdl=GenPept) | asparagine synthetase [Bacillus subtilis subsp. subtilis str. 168] | 334.17 | 7 | 738 868 966 1004 1102 1387 1528 |  |  |
| 84 | [255767352](http://www.ncbi.nlm.nih.gov/entrez/query.fcgi?cmd=Search&db=Protein&term=255767352&doptcmdl=GenPept) | cell-division protein essential fo Z-ring assembly [Bacillus subtilis subsp. subtilis str. 168] | 332.52 | 7 | 696 985 1091 1143 1326 1389 2115 |  |  |
| 85 | [7436686](http://www.ncbi.nlm.nih.gov/entrez/query.fcgi?cmd=Search&db=Protein&term=7436686&doptcmdl=GenPept) | citrate (si)-synthase (EC 4.1.3.7) citZ [validated] - Bacillus subtilis | 331.40 | 7 | 132 268 303 468 1188 1438 1579 |  |  |
|  | [487433](http://www.ncbi.nlm.nih.gov/entrez/query.fcgi?cmd=Search&db=Protein&term=487433&doptcmdl=GenPept) | citrate synthase II | 331.40 | 7 | 132 268 303 468 1188 1438 1579 |  |  |
|  | [3123196](http://www.ncbi.nlm.nih.gov/entrez/query.fcgi?cmd=Search&db=Protein&term=3123196&doptcmdl=GenPept) | Citrate synthase II | 331.40 | 7 | 132 268 303 468 1188 1438 1579 |  |  |
|  | [2635379](http://www.ncbi.nlm.nih.gov/entrez/query.fcgi?cmd=Search&db=Protein&term=2635379&doptcmdl=GenPept) | citrate synthase II [Bacillus subtilis subsp. subtilis str. 168] | 331.40 | 7 | 132 268 303 468 1188 1438 1579 |  |  |
|  | [2293267](http://www.ncbi.nlm.nih.gov/entrez/query.fcgi?cmd=Search&db=Protein&term=2293267&doptcmdl=GenPept) | citrate synthase subunit II [Bacillus subtilis] | 331.40 | 7 | 132 268 303 468 1188 1438 1579 |  |  |

| **Protein IDs*, cont.*** | | | | | | | |
| --- | --- | --- | --- | --- | --- | --- | --- |
| *Grp Nr.* | *Accession Number* | *Protein Name* | *Protein Score* | *Unique PSMs* | *PSM Serial Nrs.* | *Other Grp.* | *Score (other)* |
| 86 | [16079895](http://www.ncbi.nlm.nih.gov/entrez/query.fcgi?cmd=Search&db=Protein&term=16079895&doptcmdl=GenPept) | succinate dehydrogenase iron-sulfur subunit [Bacillus subtilis subsp. subtilis str. 168] | 327.16 | 7 | 276 709 781 1013 1585 1745 1915 |  |  |
| 87 | [16080226](http://www.ncbi.nlm.nih.gov/entrez/query.fcgi?cmd=Search&db=Protein&term=16080226&doptcmdl=GenPept) | nicotinate phosphoribosyltransferase [Bacillus subtilis subsp. subtilis str. 168] | 324.53 | 6 | 115 1338 1346 1474 1482 2109 |  |  |
| 88 | [255767453](http://www.ncbi.nlm.nih.gov/entrez/query.fcgi?cmd=Search&db=Protein&term=255767453&doptcmdl=GenPept) | 2-oxoglutarate dehydrogenase E1 component [Bacillus subtilis subsp. subtilis str. 168] | 320.03 | 7 | 148 186 516 1076 1765 1796 1816 |  |  |
| 89 | [16077160](http://www.ncbi.nlm.nih.gov/entrez/query.fcgi?cmd=Search&db=Protein&term=16077160&doptcmdl=GenPept) | glutamyl-tRNA synthetase [Bacillus subtilis subsp. subtilis str. 168] | 318.71 | 6 | 180 599 1016 2039 2080 2106 |  |  |
| 90 | [16079601](http://www.ncbi.nlm.nih.gov/entrez/query.fcgi?cmd=Search&db=Protein&term=16079601&doptcmdl=GenPept) | molecular chaperone DnaK [Bacillus subtilis subsp. subtilis str. 168] | 314.52 | 6 | 89 154 446 1032 1642 1661 |  |  |
| 91 | [98277](http://www.ncbi.nlm.nih.gov/entrez/query.fcgi?cmd=Search&db=Protein&term=98277&doptcmdl=GenPept) | dihydrolipoamide S-succinyltransferase (EC 2.3.1.61) odhB - Bacillus subtilis | 312.48 | 5 | 130 199 378 976 1634 |  |  |
|  | [2634329](http://www.ncbi.nlm.nih.gov/entrez/query.fcgi?cmd=Search&db=Protein&term=2634329&doptcmdl=GenPept) | 2-oxoglutarate dehydrogenase complex (dihydrolipoamide transsuccinylase, E2 subunit) [Bacillus subtilis subsp. subtilis str. 168] | 312.48 | 5 | 130 199 378 976 1634 |  |  |
|  | [255767452](http://www.ncbi.nlm.nih.gov/entrez/query.fcgi?cmd=Search&db=Protein&term=255767452&doptcmdl=GenPept) | dihydrolipoamide succinyltransferase [Bacillus subtilis subsp. subtilis str. 168] | 312.48 | 5 | 130 199 378 976 1634 |  |  |
|  | [16078996](http://www.ncbi.nlm.nih.gov/entrez/query.fcgi?cmd=Search&db=Protein&term=16078996&doptcmdl=GenPept) | 2-oxoglutarate dehydrogenase complex (dihydrolipoamide transsuccinylase, E2 subunit) [Bacillus subtilis] | 312.48 | 5 | 130 199 378 976 1634 |  |  |
|  | [143268](http://www.ncbi.nlm.nih.gov/entrez/query.fcgi?cmd=Search&db=Protein&term=143268&doptcmdl=GenPept) | dihydrolipoamide transsuccinylase (odhB; EC 2.3.1.61) | 312.48 | 5 | 130 199 378 976 1634 |  |  |
| 92 | [16081029](http://www.ncbi.nlm.nih.gov/entrez/query.fcgi?cmd=Search&db=Protein&term=16081029&doptcmdl=GenPept) | aldo-keto reductase [Bacillus subtilis subsp. subtilis str. 168] | 309.76 | 7 | 189 227 727 728 1123 1481 1671 |  |  |
| 93 | [16077141](http://www.ncbi.nlm.nih.gov/entrez/query.fcgi?cmd=Search&db=Protein&term=16077141&doptcmdl=GenPept) | cysteine synthase [Bacillus subtilis subsp. subtilis str. 168] | 306.84 | 7 | 663 798 897 1015 1098 1905 1994 |  |  |
| 94 | [971342](http://www.ncbi.nlm.nih.gov/entrez/query.fcgi?cmd=Search&db=Protein&term=971342&doptcmdl=GenPept) | nitrate reductase beta subunit [Bacillus subtilis] | 305.17 | 7 | 172 243 505 912 1052 1128 2052 |  |  |
|  | [7432735](http://www.ncbi.nlm.nih.gov/entrez/query.fcgi?cmd=Search&db=Protein&term=7432735&doptcmdl=GenPept) | nitrate reductase (EC 1.7.99.4) beta chain narH - Bacillus subtilis | 305.17 | 7 | 172 243 505 912 1052 1128 2052 |  |  |
|  | [2636264](http://www.ncbi.nlm.nih.gov/entrez/query.fcgi?cmd=Search&db=Protein&term=2636264&doptcmdl=GenPept) | nitrate reductase (beta subunit) [Bacillus subtilis subsp. subtilis str. 168] | 305.17 | 7 | 172 243 505 912 1052 1128 2052 |  |  |
|  | [16080780](http://www.ncbi.nlm.nih.gov/entrez/query.fcgi?cmd=Search&db=Protein&term=16080780&doptcmdl=GenPept) | nitrate reductase (beta subunit) [Bacillus subtilis] | 305.17 | 7 | 172 243 505 912 1052 1128 2052 |  |  |
|  | [1171652](http://www.ncbi.nlm.nih.gov/entrez/query.fcgi?cmd=Search&db=Protein&term=1171652&doptcmdl=GenPept) | Nitrate reductase beta chain | 305.17 | 7 | 172 243 505 912 1052 1128 2052 |  |  |
| 95 | [16079915](http://www.ncbi.nlm.nih.gov/entrez/query.fcgi?cmd=Search&db=Protein&term=16079915&doptcmdl=GenPept) | phenylalanyl-tRNA synthetase subunit beta [Bacillus subtilis subsp. subtilis str. 168] | 304.41 | 5 | 32 1208 1773 1774 2084 |  |  |
| 96 | [16079776](http://www.ncbi.nlm.nih.gov/entrez/query.fcgi?cmd=Search&db=Protein&term=16079776&doptcmdl=GenPept) | oxidoreductase [Bacillus subtilis subsp. subtilis str. 168] | 299.81 | 7 | 534 629 986 1176 1184 1551 1600 |  |  |
| 97 | [16080132](http://www.ncbi.nlm.nih.gov/entrez/query.fcgi?cmd=Search&db=Protein&term=16080132&doptcmdl=GenPept) | naphthoate synthase [Bacillus subtilis subsp. subtilis str. 168] | 298.01 | 6 | 483 546 776 1283 1301 1485 |  |  |
| 98 | [16080738](http://www.ncbi.nlm.nih.gov/entrez/query.fcgi?cmd=Search&db=Protein&term=16080738&doptcmdl=GenPept) | F0F1 ATP synthase subunit B [Bacillus subtilis subsp. subtilis str. 168] | 296.96 | 7 | 278 296 426 496 499 614 1090 |  |  |
| 99 | [16077525](http://www.ncbi.nlm.nih.gov/entrez/query.fcgi?cmd=Search&db=Protein&term=16077525&doptcmdl=GenPept) | ATP-dependent RNA helicase; cold shock [Bacillus subtilis subsp. subtilis str. 168] | 294.51 | 5 | 363 606 1246 1495 1605 |  |  |
| 100 | [16080569](http://www.ncbi.nlm.nih.gov/entrez/query.fcgi?cmd=Search&db=Protein&term=16080569&doptcmdl=GenPept) | excinuclease ABC subunit A [Bacillus subtilis subsp. subtilis str. 168] | 294.16 | 6 | 193 519 815 1111 1650 2136 |  |  |
| 101 | [16078759](http://www.ncbi.nlm.nih.gov/entrez/query.fcgi?cmd=Search&db=Protein&term=16078759&doptcmdl=GenPept) | phosphodiesterase [Bacillus subtilis subsp. subtilis str. 168] | 290.05 | 6 | 424 885 896 900 1066 1674 |  |  |
| 102 | [16079293](http://www.ncbi.nlm.nih.gov/entrez/query.fcgi?cmd=Search&db=Protein&term=16079293&doptcmdl=GenPept) | asparaginyl-tRNA synthetase [Bacillus subtilis subsp. subtilis str. 168] | 286.25 | 6 | 314 1147 1419 1580 1629 1706 |  |  |
| 103 | [16078587](http://www.ncbi.nlm.nih.gov/entrez/query.fcgi?cmd=Search&db=Protein&term=16078587&doptcmdl=GenPept) | UDP-N-acetylenolpyruvoylglucosamine reductase [Bacillus subtilis subsp. subtilis str. 168] | 279.31 | 6 | 487 886 1136 1577 1843 1863 |  |  |
| 104 | [255767369](http://www.ncbi.nlm.nih.gov/entrez/query.fcgi?cmd=Search&db=Protein&term=255767369&doptcmdl=GenPept) | flagellar basal body rod protein FlgG [Bacillus subtilis subsp. subtilis str. 168] | 279.26 | 4 | 160 923 1739 2137 |  |  |
| 105 | [255767543](http://www.ncbi.nlm.nih.gov/entrez/query.fcgi?cmd=Search&db=Protein&term=255767543&doptcmdl=GenPept) | acetyl-CoA carboxylase biotin carboxylase subunit [Bacillus subtilis subsp. subtilis str. 168] | 277.50 | 5 | 206 719 1267 1581 1842 |  |  |
| 106 | [RL7_BACSU](http://us.expasy.org/uniprot/RL7_BACSU) | 50S ribosomal protein L7/L12 (BL9) ('A' type) (Vegetative protein 341) (VEG341) | 275.85 | 6 | 78 251 476 510 878 1778 |  |  |
|  | [16077173](http://www.ncbi.nlm.nih.gov/entrez/query.fcgi?cmd=Search&db=Protein&term=16077173&doptcmdl=GenPept) | 50S ribosomal protein L7/L12 [Bacillus subtilis subsp. subtilis str. 168] | 222.56 | 5 | 251 476 510 878 1778 |  |  |
| 107 | [255767628](http://www.ncbi.nlm.nih.gov/entrez/query.fcgi?cmd=Search&db=Protein&term=255767628&doptcmdl=GenPept) | tRNA-specific 2-thiouridylase MnmA [Bacillus subtilis subsp. subtilis str. 168] | 274.20 | 7 | 566 1350 1624 1801 1828 1869 1971 |  |  |
| 108 | [16081107](http://www.ncbi.nlm.nih.gov/entrez/query.fcgi?cmd=Search&db=Protein&term=16081107&doptcmdl=GenPept) | manganese-dependent inorganic pyrophosphatase [Bacillus subtilis subsp. subtilis str. 168] | 271.34 | 4 | 256 803 1508 1604 |  |  |
| 109 | [7427681](http://www.ncbi.nlm.nih.gov/entrez/query.fcgi?cmd=Search&db=Protein&term=7427681&doptcmdl=GenPept) | phosphoglycerate dehydrogenase (EC 1.1.1.95) serA - Bacillus subtilis | 271.09 | 5 | 23 478 833 1169 1259 |  |  |
|  | [3123275](http://www.ncbi.nlm.nih.gov/entrez/query.fcgi?cmd=Search&db=Protein&term=3123275&doptcmdl=GenPept) | D-3-phosphoglycerate dehydrogenase (PGDH) | 271.09 | 5 | 23 478 833 1169 1259 |  |  |
|  | [2634742](http://www.ncbi.nlm.nih.gov/entrez/query.fcgi?cmd=Search&db=Protein&term=2634742&doptcmdl=GenPept) | phosphoglycerate dehydrogenase [Bacillus subtilis subsp. subtilis str. 168] | 271.09 | 5 | 23 478 833 1169 1259 |  |  |
|  | [255767495](http://www.ncbi.nlm.nih.gov/entrez/query.fcgi?cmd=Search&db=Protein&term=255767495&doptcmdl=GenPept) | D-3-phosphoglycerate dehydrogenase [Bacillus subtilis subsp. subtilis str. 168] | 271.09 | 5 | 23 478 833 1169 1259 |  |  |
|  | [16079364](http://www.ncbi.nlm.nih.gov/entrez/query.fcgi?cmd=Search&db=Protein&term=16079364&doptcmdl=GenPept) | phosphoglycerate dehydrogenase [Bacillus subtilis] | 271.09 | 5 | 23 478 833 1169 1259 |  |  |
| 110 | [16077081](http://www.ncbi.nlm.nih.gov/entrez/query.fcgi?cmd=Search&db=Protein&term=16077081&doptcmdl=GenPept) | seryl-tRNA synthetase [Bacillus subtilis subsp. subtilis str. 168] | 266.77 | 5 | 681 1175 1734 1878 1964 |  |  |
| 111 | [16077153](http://www.ncbi.nlm.nih.gov/entrez/query.fcgi?cmd=Search&db=Protein&term=16077153&doptcmdl=GenPept) | ATP:guanido phosphotransferase [Bacillus subtilis subsp. subtilis str. 168] | 264.82 | 5 | 689 1058 1244 1811 1935 |  |  |
| 112 | [16080937](http://www.ncbi.nlm.nih.gov/entrez/query.fcgi?cmd=Search&db=Protein&term=16080937&doptcmdl=GenPept) | UDP-glucose 4-epimerase [Bacillus subtilis subsp. subtilis str. 168] | 263.27 | 7 | 417 929 993 1075 1382 1560 2090 |  |  |
| 113 | [255767290](http://www.ncbi.nlm.nih.gov/entrez/query.fcgi?cmd=Search&db=Protein&term=255767290&doptcmdl=GenPept) | hypothetical protein BSU12650 [Bacillus subtilis subsp. subtilis str. 168] | 263.15 | 6 | 305 1095 1310 1344 1435 2014 |  |  |
| 114 | [16077070](http://www.ncbi.nlm.nih.gov/entrez/query.fcgi?cmd=Search&db=Protein&term=16077070&doptcmdl=GenPept) | DNA polymerase III subunit beta [Bacillus subtilis subsp. subtilis str. 168] | 262.95 | 5 | 76 739 821 1101 1595 |  |  |
| 115 | [16079578](http://www.ncbi.nlm.nih.gov/entrez/query.fcgi?cmd=Search&db=Protein&term=16079578&doptcmdl=GenPept) | hypothetical protein BSU25240 [Bacillus subtilis subsp. subtilis str. 168] | 260.27 | 6 | 15 144 153 988 1018 1391 |  |  |
| 116 | [255767161](http://www.ncbi.nlm.nih.gov/entrez/query.fcgi?cmd=Search&db=Protein&term=255767161&doptcmdl=GenPept) | GMP synthase [Bacillus subtilis subsp. subtilis str. 168] | 259.92 | 6 | 52 542 1144 1757 2083 2119 |  |  |
| 117 | [16078293](http://www.ncbi.nlm.nih.gov/entrez/query.fcgi?cmd=Search&db=Protein&term=16078293&doptcmdl=GenPept) | transcriptional repressor of the rex ndh operon [Bacillus subtilis subsp. subtilis str. 168] | 259.11 | 4 | 31 1630 1741 1989 |  |  |
| 118 | [16078066](http://www.ncbi.nlm.nih.gov/entrez/query.fcgi?cmd=Search&db=Protein&term=16078066&doptcmdl=GenPept) | phosphoserine aminotransferase [Bacillus subtilis subsp. subtilis str. 168] | 255.80 | 5 | 977 1213 1467 1483 1974 |  |  |
| 119 | [16077302](http://www.ncbi.nlm.nih.gov/entrez/query.fcgi?cmd=Search&db=Protein&term=16077302&doptcmdl=GenPept) | hypothetical protein BSU02330 [Bacillus subtilis subsp. subtilis str. 168] | 255.30 | 6 | 1027 1155 1329 1364 1649 1686 |  |  |
| 120 | [255767031](http://www.ncbi.nlm.nih.gov/entrez/query.fcgi?cmd=Search&db=Protein&term=255767031&doptcmdl=GenPept) | 30S ribosomal protein S8 [Bacillus subtilis subsp. subtilis str. 168] | 255.30 | 6 | 85 290 685 748 903 2103 |  |  |
| 121 | [255767489](http://www.ncbi.nlm.nih.gov/entrez/query.fcgi?cmd=Search&db=Protein&term=255767489&doptcmdl=GenPept) | chorismate synthase [Bacillus subtilis subsp. subtilis str. 168] | 254.64 | 5 | 655 783 1118 1709 2022 |  |  |
| 122 | [16080275](http://www.ncbi.nlm.nih.gov/entrez/query.fcgi?cmd=Search&db=Protein&term=16080275&doptcmdl=GenPept) | iron-sulfur scaffold protein [Bacillus subtilis subsp. subtilis str. 168] | 252.56 | 5 | 226 567 1077 1129 1587 |  |  |
| 123 | [16078523](http://www.ncbi.nlm.nih.gov/entrez/query.fcgi?cmd=Search&db=Protein&term=16078523&doptcmdl=GenPept) | pyruvate dehydrogenase (E1 beta subunit) [Bacillus subtilis subsp. subtilis str. 168] | 250.79 | 6 | 166 947 1074 1345 1714 2122 |  |  |
| 124 | [255767787](http://www.ncbi.nlm.nih.gov/entrez/query.fcgi?cmd=Search&db=Protein&term=255767787&doptcmdl=GenPept) | transcription termination factor Rho [Bacillus subtilis subsp. subtilis str. 168] | 249.69 | 6 | 50 421 852 915 1503 2025 |  |  |
| 125 | [16079964](http://www.ncbi.nlm.nih.gov/entrez/query.fcgi?cmd=Search&db=Protein&term=16079964&doptcmdl=GenPept) | malate dehydrogenase [Bacillus subtilis subsp. subtilis str. 168] | 248.88 | 4 | 19 55 721 2082 |  |  |
| 126 | [255767325](http://www.ncbi.nlm.nih.gov/entrez/query.fcgi?cmd=Search&db=Protein&term=255767325&doptcmdl=GenPept) | aminotransferase A [Bacillus subtilis subsp. subtilis str. 168] | 246.46 | 7 | 410 910 942 1116 1701 1723 1809 |  |  |
| 127 | [255767083](http://www.ncbi.nlm.nih.gov/entrez/query.fcgi?cmd=Search&db=Protein&term=255767083&doptcmdl=GenPept) | L-lactate dehydrogenase [Bacillus subtilis subsp. subtilis str. 168] | 245.98 | 5 | 334 389 1514 1527 1533 |  |  |
| 128 | [16079509](http://www.ncbi.nlm.nih.gov/entrez/query.fcgi?cmd=Search&db=Protein&term=16079509&doptcmdl=GenPept) | lipoate protein ligase [Bacillus subtilis subsp. subtilis str. 168] | 245.08 | 5 | 86 757 1140 1621 2044 |  |  |
| 129 | [255767391](http://www.ncbi.nlm.nih.gov/entrez/query.fcgi?cmd=Search&db=Protein&term=255767391&doptcmdl=GenPept) | DNA mismatch repair protein MutS [Bacillus subtilis subsp. subtilis str. 168] | 244.53 | 4 | 54 353 548 1956 |  |  |
| 130 | [16079871](http://www.ncbi.nlm.nih.gov/entrez/query.fcgi?cmd=Search&db=Protein&term=16079871&doptcmdl=GenPept) | ribosome biogenesis GTP-binding protein YsxC [Bacillus subtilis subsp. subtilis str. 168] | 244.37 | 5 | 82 1641 1873 1946 2058 |  |  |
| 131 | [255767567](http://www.ncbi.nlm.nih.gov/entrez/query.fcgi?cmd=Search&db=Protein&term=255767567&doptcmdl=GenPept) | superoxide dismutase [Bacillus subtilis subsp. subtilis str. 168] | 241.28 | 4 | 73 247 1038 2092 |  |  |
| 132 | [255767527](http://www.ncbi.nlm.nih.gov/entrez/query.fcgi?cmd=Search&db=Protein&term=255767527&doptcmdl=GenPept) | butyrate kinase [Bacillus subtilis subsp. subtilis str. 168] | 238.42 | 5 | 299 337 646 808 1166 |  |  |
| 133 | [255767374](http://www.ncbi.nlm.nih.gov/entrez/query.fcgi?cmd=Search&db=Protein&term=255767374&doptcmdl=GenPept) | chemotactic two-component sensor histidine kinase [Bacillus subtilis subsp. subtilis str. 168] | 237.65 | 5 | 11 747 881 983 2085 |  |  |
| 134 | [255767094](http://www.ncbi.nlm.nih.gov/entrez/query.fcgi?cmd=Search&db=Protein&term=255767094&doptcmdl=GenPept) | 1-pyrroline-5-carboxylate dehydrogenase [Bacillus subtilis subsp. subtilis str. 168] | 237.53 | 7 | 667 695 834 1334 1500 1749 1934 |  |  |
| 135 | [16079970](http://www.ncbi.nlm.nih.gov/entrez/query.fcgi?cmd=Search&db=Protein&term=16079970&doptcmdl=GenPept) | pyruvate kinase [Bacillus subtilis subsp. subtilis str. 168] | 237.25 | 4 | 1126 1340 1766 1775 |  |  |
| 136 | [16079336](http://www.ncbi.nlm.nih.gov/entrez/query.fcgi?cmd=Search&db=Protein&term=16079336&doptcmdl=GenPept) | non-specific DNA-binding protein HBsu; signal recognition particle-like (SRP) component [Bacillus subtilis subsp. subtilis str. 168] | 236.81 | 4 | 236 780 1750 2073 |  |  |
| 137 | [16077196](http://www.ncbi.nlm.nih.gov/entrez/query.fcgi?cmd=Search&db=Protein&term=16077196&doptcmdl=GenPept) | 50S ribosomal protein L5 [Bacillus subtilis subsp. subtilis str. 168] | 230.87 | 6 | 524 703 1178 1401 1847 1995 |  |  |
| 138 | [16079974](http://www.ncbi.nlm.nih.gov/entrez/query.fcgi?cmd=Search&db=Protein&term=16079974&doptcmdl=GenPept) | NADP-dependent malic enzyme [Bacillus subtilis subsp. subtilis str. 168] | 224.98 | 4 | 118 1096 1331 2024 |  |  |
| 139 | [16080589](http://www.ncbi.nlm.nih.gov/entrez/query.fcgi?cmd=Search&db=Protein&term=16080589&doptcmdl=GenPept) | flagellin [Bacillus subtilis subsp. subtilis str. 168] | 224.53 | 3 | 170 653 1883 |  |  |
| 140 | [16081162](http://www.ncbi.nlm.nih.gov/entrez/query.fcgi?cmd=Search&db=Protein&term=16081162&doptcmdl=GenPept) | bifunctional preprotein translocase subunit SecD/SecF [Bacillus subtilis subsp. subtilis str. 168] | 223.26 | 4 | 56 691 1844 2124 |  |  |
| 141 | [16080153](http://www.ncbi.nlm.nih.gov/entrez/query.fcgi?cmd=Search&db=Protein&term=16080153&doptcmdl=GenPept) | flotillin-like protein [Bacillus subtilis subsp. subtilis str. 168] | 223.23 | 3 | 198 390 391 |  |  |
| 142 | [50812289](http://www.ncbi.nlm.nih.gov/entrez/query.fcgi?cmd=Search&db=Protein&term=50812289&doptcmdl=GenPept) | lipoyl synthase [Bacillus subtilis subsp. subtilis str. 168] | 223.21 | 5 | 242 593 1057 1180 1437 |  |  |
| 143 | [16079275](http://www.ncbi.nlm.nih.gov/entrez/query.fcgi?cmd=Search&db=Protein&term=16079275&doptcmdl=GenPept) | methylase with RNA interaction domain [Bacillus subtilis subsp. subtilis str. 168] | 222.52 | 6 | 29 471 1153 1430 1488 1618 |  |  |
| 144 | [16079904](http://www.ncbi.nlm.nih.gov/entrez/query.fcgi?cmd=Search&db=Protein&term=16079904&doptcmdl=GenPept) | electron transfer flavoprotein alpha subunit [Bacillus subtilis subsp. subtilis str. 168] | 222.33 | 5 | 473 1220 1302 1426 1465 |  |  |
| 145 | [255767705](http://www.ncbi.nlm.nih.gov/entrez/query.fcgi?cmd=Search&db=Protein&term=255767705&doptcmdl=GenPept) | methyl-accepting chemotaxis protein [Bacillus subtilis subsp. subtilis str. 168] | 219.68 | 4 | 49 485 1007 1471 |  |  |
| 146 | [16077802](http://www.ncbi.nlm.nih.gov/entrez/query.fcgi?cmd=Search&db=Protein&term=16077802&doptcmdl=GenPept) | aldehyde dehydrogenase [Bacillus subtilis subsp. subtilis str. 168] | 219.33 | 5 | 263 457 840 1342 1782 |  |  |
| 147 | [16080570](http://www.ncbi.nlm.nih.gov/entrez/query.fcgi?cmd=Search&db=Protein&term=16080570&doptcmdl=GenPept) | excinuclease ABC subunit B [Bacillus subtilis subsp. subtilis str. 168] | 217.69 | 6 | 318 494 568 1432 1511 1682 |  |  |
| 148 | [50812220](http://www.ncbi.nlm.nih.gov/entrez/query.fcgi?cmd=Search&db=Protein&term=50812220&doptcmdl=GenPept) | enoyl-(acyl carrier protein) reductase [Bacillus subtilis subsp. subtilis str. 168] | 217.56 | 5 | 27 549 1141 1271 1591 |  |  |
| 149 | [16080445](http://www.ncbi.nlm.nih.gov/entrez/query.fcgi?cmd=Search&db=Protein&term=16080445&doptcmdl=GenPept) | triosephosphate isomerase [Bacillus subtilis subsp. subtilis str. 168] | 217.41 | 4 | 1182 1747 1753 1808 |  |  |
| 150 | [255767552](http://www.ncbi.nlm.nih.gov/entrez/query.fcgi?cmd=Search&db=Protein&term=255767552&doptcmdl=GenPept) | glycine cleavage system aminomethyltransferase T [Bacillus subtilis subsp. subtilis str. 168] | 216.82 | 4 | 185 294 1156 2006 |  |  |
| 151 | [255767373](http://www.ncbi.nlm.nih.gov/entrez/query.fcgi?cmd=Search&db=Protein&term=255767373&doptcmdl=GenPept) | chemotaxis-specific methylesterase [Bacillus subtilis subsp. subtilis str. 168] | 213.81 | 6 | 277 560 575 634 669 737 |  |  |
| 152 | [255767219](http://www.ncbi.nlm.nih.gov/entrez/query.fcgi?cmd=Search&db=Protein&term=255767219&doptcmdl=GenPept) | alpha-phosphoglucomutase [Bacillus subtilis subsp. subtilis str. 168] | 210.63 | 3 | 75 125 165 |  |  |
| 153 | [255767498](http://www.ncbi.nlm.nih.gov/entrez/query.fcgi?cmd=Search&db=Protein&term=255767498&doptcmdl=GenPept) | two-component response regulator [Bacillus subtilis subsp. subtilis str. 168] | 209.86 | 5 | 40 100 729 1478 1578 |  |  |
| 154 | [7404369](http://www.ncbi.nlm.nih.gov/entrez/query.fcgi?cmd=Search&db=Protein&term=7404369&doptcmdl=GenPept) | Fumarate hydratase, class-II (Fumarase) | 209.02 | 4 | 267 345 627 1967 |  |  |
|  | [68297](http://www.ncbi.nlm.nih.gov/entrez/query.fcgi?cmd=Search&db=Protein&term=68297&doptcmdl=GenPept) | fumarate hydratase (EC 4.2.1.2) - Bacillus subtilis | 209.02 | 4 | 267 345 627 1967 |  |  |
|  | [39844](http://www.ncbi.nlm.nih.gov/entrez/query.fcgi?cmd=Search&db=Protein&term=39844&doptcmdl=GenPept) | fumarase [Bacillus subtilis] | 209.02 | 4 | 267 345 627 1967 |  |  |
|  | [2832825](http://www.ncbi.nlm.nih.gov/entrez/query.fcgi?cmd=Search&db=Protein&term=2832825&doptcmdl=GenPept) | fumarase protein, CitG [Bacillus subtilis] | 209.02 | 4 | 267 345 627 1967 |  |  |
|  | [2635801](http://www.ncbi.nlm.nih.gov/entrez/query.fcgi?cmd=Search&db=Protein&term=2635801&doptcmdl=GenPept) | fumarate hydratase [Bacillus subtilis subsp. subtilis str. 168] | 209.02 | 4 | 267 345 627 1967 |  |  |
| 155 | [16080786](http://www.ncbi.nlm.nih.gov/entrez/query.fcgi?cmd=Search&db=Protein&term=16080786&doptcmdl=GenPept) | arginyl-tRNA synthetase [Bacillus subtilis subsp. subtilis str. 168] | 207.48 | 5 | 220 836 1198 1839 2005 |  |  |
| 156 | [16077211](http://www.ncbi.nlm.nih.gov/entrez/query.fcgi?cmd=Search&db=Protein&term=16077211&doptcmdl=GenPept) | DNA-directed RNA polymerase subunit alpha [Bacillus subtilis subsp. subtilis str. 168] | 207.11 | 4 | 891 1374 1862 2110 |  |  |
| 157 | [255767807](http://www.ncbi.nlm.nih.gov/entrez/query.fcgi?cmd=Search&db=Protein&term=255767807&doptcmdl=GenPept) | cytochrome aa3-600 quinol oxidase subunit II [Bacillus subtilis subsp. subtilis str. 168] | 206.76 | 4 | 309 672 1262 1466 |  |  |
| 158 | [255767586](http://www.ncbi.nlm.nih.gov/entrez/query.fcgi?cmd=Search&db=Protein&term=255767586&doptcmdl=GenPept) | chaperone protein DnaJ [Bacillus subtilis subsp. subtilis str. 168] | 206.40 | 4 | 351 395 545 1386 |  |  |
| 159 | [255767642](http://www.ncbi.nlm.nih.gov/entrez/query.fcgi?cmd=Search&db=Protein&term=255767642&doptcmdl=GenPept) | rod shape-determining protein MreB [Bacillus subtilis subsp. subtilis str. 168] | 204.35 | 5 | 751 767 848 1448 1576 |  |  |
| 160 | [585942](http://www.ncbi.nlm.nih.gov/entrez/query.fcgi?cmd=Search&db=Protein&term=585942&doptcmdl=GenPept) | 30S ribosomal protein S1 homolog | 202.14 | 4 | 413 600 760 1923 |  |  |
|  | [533106](http://www.ncbi.nlm.nih.gov/entrez/query.fcgi?cmd=Search&db=Protein&term=533106&doptcmdl=GenPept) | unknown | 202.14 | 4 | 413 600 760 1923 |  |  |
|  | [2634706](http://www.ncbi.nlm.nih.gov/entrez/query.fcgi?cmd=Search&db=Protein&term=2634706&doptcmdl=GenPept) | ypfD [Bacillus subtilis subsp. subtilis str. 168] [MASS=42402] | 202.14 | 4 | 413 600 760 1923 |  |  |
|  | [221323727](http://www.ncbi.nlm.nih.gov/entrez/query.fcgi?cmd=Search&db=Protein&term=221323727&doptcmdl=GenPept) | 30S ribosomal protein S1 [Bacillus subtilis subsp. subtilis str. SMY] | 202.14 | 4 | 413 600 760 1923 |  |  |
|  | [221319451](http://www.ncbi.nlm.nih.gov/entrez/query.fcgi?cmd=Search&db=Protein&term=221319451&doptcmdl=GenPept) | 30S ribosomal protein S1 [Bacillus subtilis subsp. subtilis str. JH642] | 202.14 | 4 | 413 600 760 1923 |  |  |
| 161 | [255767309](http://www.ncbi.nlm.nih.gov/entrez/query.fcgi?cmd=Search&db=Protein&term=255767309&doptcmdl=GenPept) | gamma-glutamyl phosphate reductase [Bacillus subtilis subsp. subtilis str. 168] | 200.81 | 4 | 178 901 1278 1785 |  |  |
| 162 | [255767217](http://www.ncbi.nlm.nih.gov/entrez/query.fcgi?cmd=Search&db=Protein&term=255767217&doptcmdl=GenPept) | glycerol kinase [Bacillus subtilis subsp. subtilis str. 168] | 200.66 | 6 | 539 595 979 1531 1834 1851 |  |  |
| 163 | [16077451](http://www.ncbi.nlm.nih.gov/entrez/query.fcgi?cmd=Search&db=Protein&term=16077451&doptcmdl=GenPept) | iron-siderophore ABC transporter binding lipoprotein [Bacillus subtilis subsp. subtilis str. 168] | 199.70 | 4 | 34 448 1833 1929 |  |  |
| 164 | [16078076](http://www.ncbi.nlm.nih.gov/entrez/query.fcgi?cmd=Search&db=Protein&term=16078076&doptcmdl=GenPept) | uroporphyrinogen decarboxylase [Bacillus subtilis subsp. subtilis str. 168] | 199.43 | 3 | 2 128 1779 |  |  |
| 165 | [16079245](http://www.ncbi.nlm.nih.gov/entrez/query.fcgi?cmd=Search&db=Protein&term=16079245&doptcmdl=GenPept) | hypothetical protein BSU21860 [Bacillus subtilis subsp. subtilis str. 168] | 198.37 | 4 | 248 731 917 981 |  |  |
| 166 | [255767032](http://www.ncbi.nlm.nih.gov/entrez/query.fcgi?cmd=Search&db=Protein&term=255767032&doptcmdl=GenPept) | 50S ribosomal protein L18 [Bacillus subtilis subsp. subtilis str. 168] | 198.24 | 3 | 533 1351 2078 |  |  |
| 167 | [255767185](http://www.ncbi.nlm.nih.gov/entrez/query.fcgi?cmd=Search&db=Protein&term=255767185&doptcmdl=GenPept) | ABC efflux transporter ATP-binding protein [Bacillus subtilis subsp. subtilis str. 168] | 198.18 | 4 | 432 1327 1554 1821 |  |  |
| 168 | [16079294](http://www.ncbi.nlm.nih.gov/entrez/query.fcgi?cmd=Search&db=Protein&term=16079294&doptcmdl=GenPept) | aspartate aminotransferase [Bacillus subtilis subsp. subtilis str. 168] | 197.64 | 4 | 989 991 1137 1257 |  |  |
| 169 | [16079972](http://www.ncbi.nlm.nih.gov/entrez/query.fcgi?cmd=Search&db=Protein&term=16079972&doptcmdl=GenPept) | acetyl-CoA carboxylase carboxyltransferase subunit alpha [Bacillus subtilis subsp. subtilis str. 168] | 196.34 | 3 | 447 794 1110 |  |  |
| 170 | [16080579](http://www.ncbi.nlm.nih.gov/entrez/query.fcgi?cmd=Search&db=Protein&term=16080579&doptcmdl=GenPept) | cell-division ABC transporter ATP-binding protein [Bacillus subtilis subsp. subtilis str. 168] | 194.17 | 3 | 42 1521 1547 |  |  |
| 171 | [16077212](http://www.ncbi.nlm.nih.gov/entrez/query.fcgi?cmd=Search&db=Protein&term=16077212&doptcmdl=GenPept) | 50S ribosomal protein L17 [Bacillus subtilis subsp. subtilis str. 168] | 194.14 | 3 | 346 911 1736 |  |  |
| 172 | [16081062](http://www.ncbi.nlm.nih.gov/entrez/query.fcgi?cmd=Search&db=Protein&term=16081062&doptcmdl=GenPept) | alkyl hydroperoxide reductase large subunit [Bacillus subtilis subsp. subtilis str. 168] | 193.01 | 3 | 392 827 1080 |  |  |
| 173 | [255767128](http://www.ncbi.nlm.nih.gov/entrez/query.fcgi?cmd=Search&db=Protein&term=255767128&doptcmdl=GenPept) | phosphatase [Bacillus subtilis subsp. subtilis str. 168] | 191.62 | 4 | 694 980 1776 1819 |  |  |
| 174 | [16080735](http://www.ncbi.nlm.nih.gov/entrez/query.fcgi?cmd=Search&db=Protein&term=16080735&doptcmdl=GenPept) | F0F1 ATP synthase subunit gamma [Bacillus subtilis subsp. subtilis str. 168] | 189.24 | 4 | 225 474 1040 1335 |  |  |

| **Protein IDs*, cont.*** | | | | | | | |
| --- | --- | --- | --- | --- | --- | --- | --- |
| *Grp Nr.* | *Accession Number* | *Protein Name* | *Protein Score* | *Unique PSMs* | *PSM Serial Nrs.* | *Other Grp.* | *Score (other)* |
| 175 | [16080565](http://www.ncbi.nlm.nih.gov/entrez/query.fcgi?cmd=Search&db=Protein&term=16080565&doptcmdl=GenPept) | hypothetical protein BSU35120 [Bacillus subtilis subsp. subtilis str. 168] | 188.28 | 4 | 588 1088 1918 2038 |  |  |
| 176 | [16080135](http://www.ncbi.nlm.nih.gov/entrez/query.fcgi?cmd=Search&db=Protein&term=16080135&doptcmdl=GenPept) | menaquinone-specific isochorismate synthase [Bacillus subtilis subsp. subtilis str. 168] | 187.60 | 4 | 146 365 367 2031 |  |  |
| 177 | [16079289](http://www.ncbi.nlm.nih.gov/entrez/query.fcgi?cmd=Search&db=Protein&term=16079289&doptcmdl=GenPept) | peptidoglycan glycosyltransferase [Bacillus subtilis subsp. subtilis str. 168] | 187.10 | 3 | 543 675 1724 |  |  |
| 178 | [16080322](http://www.ncbi.nlm.nih.gov/entrez/query.fcgi?cmd=Search&db=Protein&term=16080322&doptcmdl=GenPept) | FeS assembly protein SufD [Bacillus subtilis subsp. subtilis str. 168] | 186.80 | 4 | 949 1685 1882 2062 |  |  |
| 179 | [50812227](http://www.ncbi.nlm.nih.gov/entrez/query.fcgi?cmd=Search&db=Protein&term=50812227&doptcmdl=GenPept) | uridylate kinase [Bacillus subtilis subsp. subtilis str. 168] | 186.03 | 3 | 497 1316 1672 |  |  |
| 180 | [16081094](http://www.ncbi.nlm.nih.gov/entrez/query.fcgi?cmd=Search&db=Protein&term=16081094&doptcmdl=GenPept) | adenylosuccinate synthetase [Bacillus subtilis subsp. subtilis str. 168] | 185.65 | 4 | 101 957 1705 2088 |  |  |
| 181 | [16081092](http://www.ncbi.nlm.nih.gov/entrez/query.fcgi?cmd=Search&db=Protein&term=16081092&doptcmdl=GenPept) | two-component sensor histidine kinase YycF [Bacillus subtilis subsp. subtilis str. 168] | 183.87 | 4 | 37 683 1070 2040 |  |  |
| 182 | [16078703](http://www.ncbi.nlm.nih.gov/entrez/query.fcgi?cmd=Search&db=Protein&term=16078703&doptcmdl=GenPept) | flagellar biosynthesis regulator FlhF [Bacillus subtilis subsp. subtilis str. 168] | 183.83 | 3 | 145 1318 1949 |  |  |
| 183 | [16078680](http://www.ncbi.nlm.nih.gov/entrez/query.fcgi?cmd=Search&db=Protein&term=16078680&doptcmdl=GenPept) | transcriptional repressor CodY [Bacillus subtilis subsp. subtilis str. 168] | 183.54 | 3 | 213 1848 1887 |  |  |
| 184 | [16077462](http://www.ncbi.nlm.nih.gov/entrez/query.fcgi?cmd=Search&db=Protein&term=16077462&doptcmdl=GenPept) | hypothetical protein BSU03940 [Bacillus subtilis subsp. subtilis str. 168] | 182.99 | 3 | 1469 1611 1920 |  |  |
| 185 | [16078606](http://www.ncbi.nlm.nih.gov/entrez/query.fcgi?cmd=Search&db=Protein&term=16078606&doptcmdl=GenPept) | cell-division initiation protein [Bacillus subtilis subsp. subtilis str. 168] | 182.43 | 3 | 740 887 2055 |  |  |
| 186 | [16079905](http://www.ncbi.nlm.nih.gov/entrez/query.fcgi?cmd=Search&db=Protein&term=16079905&doptcmdl=GenPept) | electron transfer flavoprotein beta subunit [Bacillus subtilis subsp. subtilis str. 168] | 181.93 | 2 | 9 1854 |  |  |
| 187 | [16080321](http://www.ncbi.nlm.nih.gov/entrez/query.fcgi?cmd=Search&db=Protein&term=16080321&doptcmdl=GenPept) | cysteine desulfurase [Bacillus subtilis subsp. subtilis str. 168] | 179.96 | 4 | 292 710 1561 2134 |  |  |
| 188 | [16080507](http://www.ncbi.nlm.nih.gov/entrez/query.fcgi?cmd=Search&db=Protein&term=16080507&doptcmdl=GenPept) | ATP-dependent Clp protease proteolytic subunit [Bacillus subtilis subsp. subtilis str. 168] | 179.86 | 4 | 579 926 1061 1510 |  |  |
| 189 | [16079788](http://www.ncbi.nlm.nih.gov/entrez/query.fcgi?cmd=Search&db=Protein&term=16079788&doptcmdl=GenPept) | hydrolase [Bacillus subtilis subsp. subtilis str. 168] | 179.86 | 3 | 47 117 638 |  |  |
| 190 | [16078020](http://www.ncbi.nlm.nih.gov/entrez/query.fcgi?cmd=Search&db=Protein&term=16078020&doptcmdl=GenPept) | transporter or sensor [Bacillus subtilis subsp. subtilis str. 168] | 179.71 | 5 | 453 964 1223 1612 1742 |  |  |
| 191 | [16079313](http://www.ncbi.nlm.nih.gov/entrez/query.fcgi?cmd=Search&db=Protein&term=16079313&doptcmdl=GenPept) | menaquinol:cytochrome c oxidoreductase iron-sulfur subunit [Bacillus subtilis subsp. subtilis str. 168] | 179.31 | 4 | 1043 1304 1431 1590 |  |  |
| 192 | [7431186](http://www.ncbi.nlm.nih.gov/entrez/query.fcgi?cmd=Search&db=Protein&term=7431186&doptcmdl=GenPept) | 3-hydroxyisobutyrate dehydrogenase homolog ykwC - Bacillus subtilis | 179.19 | 2 | 518 1768 |  |  |
|  | [3183454](http://www.ncbi.nlm.nih.gov/entrez/query.fcgi?cmd=Search&db=Protein&term=3183454&doptcmdl=GenPept) | Hypothetical oxidoreductase ykwC | 179.19 | 2 | 518 1768 |  |  |
|  | [2633767](http://www.ncbi.nlm.nih.gov/entrez/query.fcgi?cmd=Search&db=Protein&term=2633767&doptcmdl=GenPept) | ykwC [Bacillus subtilis subsp. subtilis str. 168] | 179.19 | 2 | 518 1768 |  |  |
|  | [2632217](http://www.ncbi.nlm.nih.gov/entrez/query.fcgi?cmd=Search&db=Protein&term=2632217&doptcmdl=GenPept) | YkwC protein [Bacillus subtilis] | 179.19 | 2 | 518 1768 |  |  |
|  | [16078460](http://www.ncbi.nlm.nih.gov/entrez/query.fcgi?cmd=Search&db=Protein&term=16078460&doptcmdl=GenPept) | similar to 3-hydroxyisobutyrate dehydrogenase [Bacillus subtilis] | 179.19 | 2 | 518 1768 |  |  |
| 193 | [16080163](http://www.ncbi.nlm.nih.gov/entrez/query.fcgi?cmd=Search&db=Protein&term=16080163&doptcmdl=GenPept) | hypothetical protein BSU31110 [Bacillus subtilis subsp. subtilis str. 168] | 178.14 | 2 | 631 720 |  |  |
| 194 | [16080760](http://www.ncbi.nlm.nih.gov/entrez/query.fcgi?cmd=Search&db=Protein&term=16080760&doptcmdl=GenPept) | 50S ribosomal protein L31 [Bacillus subtilis subsp. subtilis str. 168] | 177.87 | 3 | 484 623 914 |  |  |
| 195 | [16078623](http://www.ncbi.nlm.nih.gov/entrez/query.fcgi?cmd=Search&db=Protein&term=16078623&doptcmdl=GenPept) | sulfate adenylyltransferase [Bacillus subtilis subsp. subtilis str. 168] | 175.71 | 3 | 104 123 1065 |  |  |
| 196 | [255767673](http://www.ncbi.nlm.nih.gov/entrez/query.fcgi?cmd=Search&db=Protein&term=255767673&doptcmdl=GenPept) | acetyl-CoA carboxylase subunit beta [Bacillus subtilis subsp. subtilis str. 168] | 173.98 | 3 | 412 580 2018 |  |  |
| 197 | [16078673](http://www.ncbi.nlm.nih.gov/entrez/query.fcgi?cmd=Search&db=Protein&term=16078673&doptcmdl=GenPept) | succinyl-CoA synthetase subunit alpha [Bacillus subtilis subsp. subtilis str. 168] | 173.54 | 3 | 1089 1339 1370 |  |  |
| 198 | [16080936](http://www.ncbi.nlm.nih.gov/entrez/query.fcgi?cmd=Search&db=Protein&term=16080936&doptcmdl=GenPept) | hypothetical protein BSU38850 [Bacillus subtilis subsp. subtilis str. 168] | 173.29 | 5 | 469 562 603 617 1009 |  |  |
| 199 | [2634702](http://www.ncbi.nlm.nih.gov/entrez/query.fcgi?cmd=Search&db=Protein&term=2634702&doptcmdl=GenPept) | yphC [Bacillus subtilis subsp. subtilis str. 168] [MASS=48769] | 172.97 | 3 | 350 442 1519 |  |  |
|  | [221323721](http://www.ncbi.nlm.nih.gov/entrez/query.fcgi?cmd=Search&db=Protein&term=221323721&doptcmdl=GenPept) | GTP-binding protein EngA [Bacillus subtilis subsp. subtilis str. SMY] | 172.97 | 3 | 350 442 1519 |  |  |
|  | [221319445](http://www.ncbi.nlm.nih.gov/entrez/query.fcgi?cmd=Search&db=Protein&term=221319445&doptcmdl=GenPept) | GTP-binding protein EngA [Bacillus subtilis subsp. subtilis str. JH642] | 172.97 | 3 | 350 442 1519 |  |  |
|  | [221314523](http://www.ncbi.nlm.nih.gov/entrez/query.fcgi?cmd=Search&db=Protein&term=221314523&doptcmdl=GenPept) | GTP-binding protein EngA [Bacillus subtilis subsp. subtilis str. NCIB 3610] | 172.97 | 3 | 350 442 1519 |  |  |
|  | [221310200](http://www.ncbi.nlm.nih.gov/entrez/query.fcgi?cmd=Search&db=Protein&term=221310200&doptcmdl=GenPept) | GTP-binding protein EngA [Bacillus subtilis subsp. subtilis str. 168] | 172.97 | 3 | 350 442 1519 |  |  |
| 200 | [255767573](http://www.ncbi.nlm.nih.gov/entrez/query.fcgi?cmd=Search&db=Protein&term=255767573&doptcmdl=GenPept) | RNA polymerase sigma factor RpoD [Bacillus subtilis subsp. subtilis str. 168] | 172.02 | 5 | 12 1021 1042 1238 1832 |  |  |
| 201 | [16079770](http://www.ncbi.nlm.nih.gov/entrez/query.fcgi?cmd=Search&db=Protein&term=16079770&doptcmdl=GenPept) | cytochrome P450 [Bacillus subtilis subsp. subtilis str. 168] | 171.31 | 4 | 1168 1627 1687 2016 |  |  |
| 202 | [16078768](http://www.ncbi.nlm.nih.gov/entrez/query.fcgi?cmd=Search&db=Protein&term=16078768&doptcmdl=GenPept) | DNA mismatch repair protein [Bacillus subtilis subsp. subtilis str. 168] | 170.34 | 4 | 615 845 1221 1769 |  |  |
| 203 | [16080976](http://www.ncbi.nlm.nih.gov/entrez/query.fcgi?cmd=Search&db=Protein&term=16080976&doptcmdl=GenPept) | phosphate starvation protein [Bacillus subtilis subsp. subtilis str. 168] | 169.84 | 3 | 578 1222 1353 |  |  |
| 204 | [16077069](http://www.ncbi.nlm.nih.gov/entrez/query.fcgi?cmd=Search&db=Protein&term=16077069&doptcmdl=GenPept) | chromosomal replication initiation protein [Bacillus subtilis subsp. subtilis str. 168] | 169.45 | 4 | 374 863 2013 2100 |  |  |
| 205 | [50812264](http://www.ncbi.nlm.nih.gov/entrez/query.fcgi?cmd=Search&db=Protein&term=50812264&doptcmdl=GenPept) | 6-phosphogluconate dehydrogenase [Bacillus subtilis subsp. subtilis str. 168] | 168.30 | 3 | 95 121 204 |  |  |
| 206 | [16079483](http://www.ncbi.nlm.nih.gov/entrez/query.fcgi?cmd=Search&db=Protein&term=16079483&doptcmdl=GenPept) | 1-deoxy-D-xylulose-5-phosphate synthase [Bacillus subtilis subsp. subtilis str. 168] | 168.30 | 4 | 108 388 552 1549 |  |  |
| 207 | [255767388](http://www.ncbi.nlm.nih.gov/entrez/query.fcgi?cmd=Search&db=Protein&term=255767388&doptcmdl=GenPept) | competence damage-inducible protein A [Bacillus subtilis subsp. subtilis str. 168] | 167.24 | 4 | 156 755 1875 1888 |  |  |
| 208 | [16078524](http://www.ncbi.nlm.nih.gov/entrez/query.fcgi?cmd=Search&db=Protein&term=16078524&doptcmdl=GenPept) | branched-chain alpha-keto acid dehydrogenase subunit E2 [Bacillus subtilis subsp. subtilis str. 168] | 167.14 | 2 | 244 1903 |  |  |
| 209 | [16080932](http://www.ncbi.nlm.nih.gov/entrez/query.fcgi?cmd=Search&db=Protein&term=16080932&doptcmdl=GenPept) | multiple sugar-binding transporter ATP-binding protein [Bacillus subtilis subsp. subtilis str. 168] | 164.67 | 3 | 734 756 1670 |  |  |
| 210 | [16080925](http://www.ncbi.nlm.nih.gov/entrez/query.fcgi?cmd=Search&db=Protein&term=16080925&doptcmdl=GenPept) | ABC membrane transporter ATP-binding protein [Bacillus subtilis subsp. subtilis str. 168] | 164.15 | 6 | 70 445 856 1085 1907 1919 |  |  |
| 211 | [16078685](http://www.ncbi.nlm.nih.gov/entrez/query.fcgi?cmd=Search&db=Protein&term=16078685&doptcmdl=GenPept) | flagellar motor switch protein G [Bacillus subtilis subsp. subtilis str. 168] | 163.74 | 3 | 22 306 1897 |  |  |
| 212 | [16079562](http://www.ncbi.nlm.nih.gov/entrez/query.fcgi?cmd=Search&db=Protein&term=16079562&doptcmdl=GenPept) | 4-hydroxy-3-methylbut-2-en-1-yl diphosphate synthase [Bacillus subtilis subsp. subtilis str. 168] | 163.57 | 3 | 508 1145 1717 |  |  |
| 213 | [255767572](http://www.ncbi.nlm.nih.gov/entrez/query.fcgi?cmd=Search&db=Protein&term=255767572&doptcmdl=GenPept) | 4-hydroxy-3-methylbut-2-enyl diphosphate reductase [Bacillus subtilis subsp. subtilis str. 168] | 163.12 | 4 | 660 869 990 1154 |  |  |
| 214 | [16081086](http://www.ncbi.nlm.nih.gov/entrez/query.fcgi?cmd=Search&db=Protein&term=16081086&doptcmdl=GenPept) | ornithine--oxo-acid transaminase [Bacillus subtilis subsp. subtilis str. 168] | 162.92 | 3 | 126 1550 1639 |  |  |
| 215 | [483136](http://www.ncbi.nlm.nih.gov/entrez/query.fcgi?cmd=Search&db=Protein&term=483136&doptcmdl=GenPept) | porphobilinogen synthase (EC 4.2.1.24) hemB - Bacillus subtilis | 162.45 | 4 | 210 321 506 1405 |  |  |
|  | [399870](http://www.ncbi.nlm.nih.gov/entrez/query.fcgi?cmd=Search&db=Protein&term=399870&doptcmdl=GenPept) | Delta-aminolevulinic acid dehydratase (Porphobilinogen synthase) (ALAD) (ALADH) | 162.45 | 4 | 210 321 506 1405 |  |  |
|  | [2635278](http://www.ncbi.nlm.nih.gov/entrez/query.fcgi?cmd=Search&db=Protein&term=2635278&doptcmdl=GenPept) | delta-aminolevulinic acid dehydratase (porphobilinogen synthase) [Bacillus subtilis subsp. subtilis str. 168] | 162.45 | 4 | 210 321 506 1405 |  |  |
|  | [16079865](http://www.ncbi.nlm.nih.gov/entrez/query.fcgi?cmd=Search&db=Protein&term=16079865&doptcmdl=GenPept) | delta-aminolevulinic acid dehydratase (porphobilinogen synthase) [Bacillus subtilis] | 162.45 | 4 | 210 321 506 1405 |  |  |
|  | [143039](http://www.ncbi.nlm.nih.gov/entrez/query.fcgi?cmd=Search&db=Protein&term=143039&doptcmdl=GenPept) | aminolevulinic acid dehydratase | 162.45 | 4 | 210 321 506 1405 |  |  |
| 216 | [16080603](http://www.ncbi.nlm.nih.gov/entrez/query.fcgi?cmd=Search&db=Protein&term=16080603&doptcmdl=GenPept) | two-component sensor histidine kinase [Bacillus subtilis subsp. subtilis str. 168] | 161.70 | 3 | 1452 1871 1943 |  |  |
| 217 | [16078852](http://www.ncbi.nlm.nih.gov/entrez/query.fcgi?cmd=Search&db=Protein&term=16078852&doptcmdl=GenPept) | transketolase [Bacillus subtilis subsp. subtilis str. 168] | 161.14 | 4 | 384 894 1044 1638 |  |  |
| 218 | [16080754](http://www.ncbi.nlm.nih.gov/entrez/query.fcgi?cmd=Search&db=Protein&term=16080754&doptcmdl=GenPept) | peptide chain release factor 1 [Bacillus subtilis subsp. subtilis str. 168] | 161.12 | 2 | 1298 1666 |  |  |
| 219 | [255767112](http://www.ncbi.nlm.nih.gov/entrez/query.fcgi?cmd=Search&db=Protein&term=255767112&doptcmdl=GenPept) | cystine ABC transporter substrate-binding lipoprotein [Bacillus subtilis subsp. subtilis str. 168] | 160.63 | 3 | 373 1541 1564 |  |  |
| 220 | [16079807](http://www.ncbi.nlm.nih.gov/entrez/query.fcgi?cmd=Search&db=Protein&term=16079807&doptcmdl=GenPept) | recombination factor protein RarA [Bacillus subtilis subsp. subtilis str. 168] | 159.84 | 2 | 17 163 |  |  |
| 221 | [16079848](http://www.ncbi.nlm.nih.gov/entrez/query.fcgi?cmd=Search&db=Protein&term=16079848&doptcmdl=GenPept) | 50S ribosomal protein L21 [Bacillus subtilis subsp. subtilis str. 168] | 159.64 | 3 | 449 563 591 |  |  |
| 222 | [255767353](http://www.ncbi.nlm.nih.gov/entrez/query.fcgi?cmd=Search&db=Protein&term=255767353&doptcmdl=GenPept) | cell division protein FtsZ [Bacillus subtilis subsp. subtilis str. 168] | 157.71 | 3 | 1 407 1623 |  |  |
| 223 | [16080019](http://www.ncbi.nlm.nih.gov/entrez/query.fcgi?cmd=Search&db=Protein&term=16080019&doptcmdl=GenPept) | tyrosyl-tRNA synthetase [Bacillus subtilis subsp. subtilis str. 168] | 156.55 | 4 | 171 520 1716 1954 |  |  |
| 224 | [16081061](http://www.ncbi.nlm.nih.gov/entrez/query.fcgi?cmd=Search&db=Protein&term=16081061&doptcmdl=GenPept) | alkyl hydroperoxide reductase small subunit [Bacillus subtilis subsp. subtilis str. 168] | 156.44 | 2 | 1341 1815 |  |  |
| 225 | [862985](http://www.ncbi.nlm.nih.gov/entrez/query.fcgi?cmd=Search&db=Protein&term=862985&doptcmdl=GenPept) | SpoVS | 155.87 | 2 | 1536 1762 |  |  |
|  | [7475827](http://www.ncbi.nlm.nih.gov/entrez/query.fcgi?cmd=Search&db=Protein&term=7475827&doptcmdl=GenPept) | spore coat dehydratation and assembly protein spoVS - Bacillus subtilis | 155.87 | 2 | 1536 1762 |  |  |
|  | [2634070](http://www.ncbi.nlm.nih.gov/entrez/query.fcgi?cmd=Search&db=Protein&term=2634070&doptcmdl=GenPept) | spoVS [Bacillus subtilis subsp. subtilis str. 168] | 155.87 | 2 | 1536 1762 |  |  |
|  | [16078761](http://www.ncbi.nlm.nih.gov/entrez/query.fcgi?cmd=Search&db=Protein&term=16078761&doptcmdl=GenPept) | spoVS [Bacillus subtilis] | 155.87 | 2 | 1536 1762 |  |  |
|  | [1174408](http://www.ncbi.nlm.nih.gov/entrez/query.fcgi?cmd=Search&db=Protein&term=1174408&doptcmdl=GenPept) | Stage V sporulation protein S | 155.87 | 2 | 1536 1762 |  |  |
| 226 | [50812262](http://www.ncbi.nlm.nih.gov/entrez/query.fcgi?cmd=Search&db=Protein&term=50812262&doptcmdl=GenPept) | methyl-accepting chemotaxis protein [Bacillus subtilis subsp. subtilis str. 168] | 155.72 | 2 | 175 1932 |  |  |
| 227 | [16079794](http://www.ncbi.nlm.nih.gov/entrez/query.fcgi?cmd=Search&db=Protein&term=16079794&doptcmdl=GenPept) | alanyl-tRNA synthetase [Bacillus subtilis subsp. subtilis str. 168] | 153.01 | 4 | 264 399 1439 1987 |  |  |
| 228 | [16077210](http://www.ncbi.nlm.nih.gov/entrez/query.fcgi?cmd=Search&db=Protein&term=16077210&doptcmdl=GenPept) | 30S ribosomal protein S11 [Bacillus subtilis subsp. subtilis str. 168] | 152.65 | 2 | 686 1300 |  |  |
| 229 | [16080160](http://www.ncbi.nlm.nih.gov/entrez/query.fcgi?cmd=Search&db=Protein&term=16080160&doptcmdl=GenPept) | hypothetical protein BSU31080 [Bacillus subtilis subsp. subtilis str. 168] | 152.45 | 2 | 1064 1783 |  |  |
| 230 | [16080128](http://www.ncbi.nlm.nih.gov/entrez/query.fcgi?cmd=Search&db=Protein&term=16080128&doptcmdl=GenPept) | manganese ABC transporter ATP-binding protein [Bacillus subtilis subsp. subtilis str. 168] | 151.71 | 3 | 282 433 1366 |  |  |
| 231 | [16077363](http://www.ncbi.nlm.nih.gov/entrez/query.fcgi?cmd=Search&db=Protein&term=16077363&doptcmdl=GenPept) | hypothetical protein BSU02940 [Bacillus subtilis subsp. subtilis str. 168] | 151.37 | 3 | 147 582 713 |  |  |
| 232 | [50812190](http://www.ncbi.nlm.nih.gov/entrez/query.fcgi?cmd=Search&db=Protein&term=50812190&doptcmdl=GenPept) | co-chaperonin GroES [Bacillus subtilis subsp. subtilis str. 168] | 149.50 | 3 | 793 1274 1517 |  |  |
| 233 | [16080457](http://www.ncbi.nlm.nih.gov/entrez/query.fcgi?cmd=Search&db=Protein&term=16080457&doptcmdl=GenPept) | iron-sulfur oxidoreductase [Bacillus subtilis subsp. subtilis str. 168] | 147.72 | 2 | 105 1433 |  |  |
| 234 | [16079276](http://www.ncbi.nlm.nih.gov/entrez/query.fcgi?cmd=Search&db=Protein&term=16079276&doptcmdl=GenPept) | cell division protein GpsB [Bacillus subtilis subsp. subtilis str. 168] | 147.46 | 3 | 959 1280 2049 |  |  |
| 235 | [255767563](http://www.ncbi.nlm.nih.gov/entrez/query.fcgi?cmd=Search&db=Protein&term=255767563&doptcmdl=GenPept) | hypothetical protein BSU24940 [Bacillus subtilis subsp. subtilis str. 168] | 147.28 | 2 | 1186 1891 |  |  |
| 236 | [255767632](http://www.ncbi.nlm.nih.gov/entrez/query.fcgi?cmd=Search&db=Protein&term=255767632&doptcmdl=GenPept) | preprotein translocase subunit YajC [Bacillus subtilis subsp. subtilis str. 168] | 147.17 | 2 | 1114 1354 |  |  |
| 237 | [16078089](http://www.ncbi.nlm.nih.gov/entrez/query.fcgi?cmd=Search&db=Protein&term=16078089&doptcmdl=GenPept) | lipoate-protein ligase [Bacillus subtilis subsp. subtilis str. 168] | 146.68 | 3 | 419 458 1617 |  |  |
| 238 | [255767838](http://www.ncbi.nlm.nih.gov/entrez/query.fcgi?cmd=Search&db=Protein&term=255767838&doptcmdl=GenPept) | deoxyribose-phosphate aldolase [Bacillus subtilis subsp. subtilis str. 168] | 145.14 | 3 | 678 1031 1916 |  |  |
| 239 | [16077222](http://www.ncbi.nlm.nih.gov/entrez/query.fcgi?cmd=Search&db=Protein&term=16077222&doptcmdl=GenPept) | Mrp family regulator [Bacillus subtilis subsp. subtilis str. 168] | 145.01 | 3 | 369 944 1219 |  |  |
| 240 | [16081149](http://www.ncbi.nlm.nih.gov/entrez/query.fcgi?cmd=Search&db=Protein&term=16081149&doptcmdl=GenPept) | chromosome partitioning protein; transcriptional regulator [Bacillus subtilis subsp. subtilis str. 168] | 144.78 | 3 | 429 1333 1877 |  |  |
| 241 | [16078059](http://www.ncbi.nlm.nih.gov/entrez/query.fcgi?cmd=Search&db=Protein&term=16078059&doptcmdl=GenPept) | molecular chaperone lipoprotein [Bacillus subtilis subsp. subtilis str. 168] | 144.42 | 3 | 561 1555 2111 |  |  |
| 242 | [16080005](http://www.ncbi.nlm.nih.gov/entrez/query.fcgi?cmd=Search&db=Protein&term=16080005&doptcmdl=GenPept) | signal peptide peptidase [Bacillus subtilis subsp. subtilis str. 168] | 144.14 | 3 | 558 970 1453 |  |  |
| 243 | [16078198](http://www.ncbi.nlm.nih.gov/entrez/query.fcgi?cmd=Search&db=Protein&term=16078198&doptcmdl=GenPept) | 3-oxoacyl-(acyl carrier protein) synthase III [Bacillus subtilis subsp. subtilis str. 168] | 144.09 | 4 | 122 455 604 1251 |  |  |
| 244 | [16078694](http://www.ncbi.nlm.nih.gov/entrez/query.fcgi?cmd=Search&db=Protein&term=16078694&doptcmdl=GenPept) | flagellar motor switch protein FliM [Bacillus subtilis subsp. subtilis str. 168] | 143.85 | 2 | 810 1973 |  |  |
| 245 | [16077192](http://www.ncbi.nlm.nih.gov/entrez/query.fcgi?cmd=Search&db=Protein&term=16077192&doptcmdl=GenPept) | 50S ribosomal protein L29 [Bacillus subtilis subsp. subtilis str. 168] | 143.54 | 3 | 700 1113 1411 |  |  |
| 246 | [16080401](http://www.ncbi.nlm.nih.gov/entrez/query.fcgi?cmd=Search&db=Protein&term=16080401&doptcmdl=GenPept) | thiol-disulfide oxidoreductase [Bacillus subtilis subsp. subtilis str. 168] | 143.44 | 3 | 495 1677 2068 |  |  |
| 247 | [7437409](http://www.ncbi.nlm.nih.gov/entrez/query.fcgi?cmd=Search&db=Protein&term=7437409&doptcmdl=GenPept) | phosphoglycerate mutase (EC 5.4.2.1), 2, 3-diphosphoglycerate-independent [validated] - Bacillus subtilis | 142.24 | 4 | 517 924 1164 2098 |  |  |
|  | [460258](http://www.ncbi.nlm.nih.gov/entrez/query.fcgi?cmd=Search&db=Protein&term=460258&doptcmdl=GenPept) | phosphoglycerate mutase | 142.24 | 4 | 517 924 1164 2098 |  |  |
|  | [3915798](http://www.ncbi.nlm.nih.gov/entrez/query.fcgi?cmd=Search&db=Protein&term=3915798&doptcmdl=GenPept) | 2,3-bisphosphoglycerate-independent phosphoglycerate mutase (Phosphoglyceromutase) (BPG-independent PGAM) (iPGM) (Vegetative protein 107) (VEG107) | 142.24 | 4 | 517 924 1164 2098 |  |  |
|  | [2635904](http://www.ncbi.nlm.nih.gov/entrez/query.fcgi?cmd=Search&db=Protein&term=2635904&doptcmdl=GenPept) | phosphoglycerate mutase [Bacillus subtilis subsp. subtilis str. 168] | 142.24 | 4 | 517 924 1164 2098 |  |  |
|  | [16080444](http://www.ncbi.nlm.nih.gov/entrez/query.fcgi?cmd=Search&db=Protein&term=16080444&doptcmdl=GenPept) | phosphoglycerate mutase [Bacillus subtilis] | 142.24 | 4 | 517 924 1164 2098 |  |  |
| 248 | [16078558](http://www.ncbi.nlm.nih.gov/entrez/query.fcgi?cmd=Search&db=Protein&term=16078558&doptcmdl=GenPept) | hypothetical protein BSU14940 [Bacillus subtilis subsp. subtilis str. 168] | 141.53 | 3 | 587 611 1266 |  |  |
| 249 | [255767149](http://www.ncbi.nlm.nih.gov/entrez/query.fcgi?cmd=Search&db=Protein&term=255767149&doptcmdl=GenPept) | glycosyltransferase [Bacillus subtilis subsp. subtilis str. 168] | 141.50 | 3 | 782 795 1908 |  |  |
| 250 | [16077397](http://www.ncbi.nlm.nih.gov/entrez/query.fcgi?cmd=Search&db=Protein&term=16077397&doptcmdl=GenPept) | uroporphyrin-III C-methyltransferase [Bacillus subtilis subsp. subtilis str. 168] | 141.08 | 3 | 502 1572 2138 |  |  |
| 251 | [16080006](http://www.ncbi.nlm.nih.gov/entrez/query.fcgi?cmd=Search&db=Protein&term=16080006&doptcmdl=GenPept) | inorganic polyphosphate/ATP-NAD kinase [Bacillus subtilis subsp. subtilis str. 168] | 139.95 | 4 | 477 1190 1250 1436 |  |  |
| 252 | [16080765](http://www.ncbi.nlm.nih.gov/entrez/query.fcgi?cmd=Search&db=Protein&term=16080765&doptcmdl=GenPept) | fructose-bisphosphate aldolase [Bacillus subtilis subsp. subtilis str. 168] | 139.39 | 3 | 493 1185 1490 |  |  |
| 253 | [16077204](http://www.ncbi.nlm.nih.gov/entrez/query.fcgi?cmd=Search&db=Protein&term=16077204&doptcmdl=GenPept) | preprotein translocase subunit SecY [Bacillus subtilis subsp. subtilis str. 168] | 139.12 | 2 | 349 1602 |  |  |
| 254 | [LUXS_BACSU](http://us.expasy.org/uniprot/LUXS_BACSU) | S-ribosylhomocysteine lyase (EC 4.4.1.21) (Autoinducer-2 production protein luxS) (AI-2 synthesis protein) | 138.61 | 3 | 1059 1507 1615 |  |  |
|  | [16080119](http://www.ncbi.nlm.nih.gov/entrez/query.fcgi?cmd=Search&db=Protein&term=16080119&doptcmdl=GenPept) | S-ribosylhomocysteinase [Bacillus subtilis subsp. subtilis str. 168] | 100.56 | 2 | 1059 1507 |  |  |
| 255 | [16080448](http://www.ncbi.nlm.nih.gov/entrez/query.fcgi?cmd=Search&db=Protein&term=16080448&doptcmdl=GenPept) | transcriptional regulator of gapA [Bacillus subtilis subsp. subtilis str. 168] | 138.26 | 3 | 1491 1899 2059 |  |  |
| 256 | [16080183](http://www.ncbi.nlm.nih.gov/entrez/query.fcgi?cmd=Search&db=Protein&term=16080183&doptcmdl=GenPept) | metal-dependent protease/peptidase [Bacillus subtilis subsp. subtilis str. 168] | 137.69 | 3 | 459 722 2008 |  |  |
| 257 | [16078519](http://www.ncbi.nlm.nih.gov/entrez/query.fcgi?cmd=Search&db=Protein&term=16078519&doptcmdl=GenPept) | hydrolase [Bacillus subtilis subsp. subtilis str. 168] | 137.17 | 3 | 996 1525 1704 |  |  |
| 258 | [16080031](http://www.ncbi.nlm.nih.gov/entrez/query.fcgi?cmd=Search&db=Protein&term=16080031&doptcmdl=GenPept) | UDP-N-acetylmuramate--L-alanine ligase [Bacillus subtilis subsp. subtilis str. 168] | 137.10 | 3 | 312 921 1303 |  |  |
| 259 | [16080742](http://www.ncbi.nlm.nih.gov/entrez/query.fcgi?cmd=Search&db=Protein&term=16080742&doptcmdl=GenPept) | uracil phosphoribosyltransferase [Bacillus subtilis subsp. subtilis str. 168] | 136.89 | 4 | 307 1192 1369 1402 |  |  |
| 260 | [16078696](http://www.ncbi.nlm.nih.gov/entrez/query.fcgi?cmd=Search&db=Protein&term=16078696&doptcmdl=GenPept) | regulator of chemotaxis and motility [Bacillus subtilis subsp. subtilis str. 168] | 136.63 | 2 | 1308 1582 |  |  |
| 261 | [16078438](http://www.ncbi.nlm.nih.gov/entrez/query.fcgi?cmd=Search&db=Protein&term=16078438&doptcmdl=GenPept) | queuosine biosynthesis enzyme [Bacillus subtilis subsp. subtilis str. 168] | 135.79 | 3 | 39 1707 1823 |  |  |

| **Protein IDs*, cont.*** | | | | | | | |
| --- | --- | --- | --- | --- | --- | --- | --- |
| *Grp Nr.* | *Accession Number* | *Protein Name* | *Protein Score* | *Unique PSMs* | *PSM Serial Nrs.* | *Other Grp.* | *Score (other)* |
| 262 | [16078500](http://www.ncbi.nlm.nih.gov/entrez/query.fcgi?cmd=Search&db=Protein&term=16078500&doptcmdl=GenPept) | ABC transporter ATP-binding protein [Bacillus subtilis subsp. subtilis str. 168] | 135.26 | 2 | 158 1292 |  |  |
| 263 | [255767038](http://www.ncbi.nlm.nih.gov/entrez/query.fcgi?cmd=Search&db=Protein&term=255767038&doptcmdl=GenPept) | 50S ribosomal protein L13 [Bacillus subtilis subsp. subtilis str. 168] | 134.72 | 4 | 364 766 962 1210 |  |  |
| 264 | [7430017](http://www.ncbi.nlm.nih.gov/entrez/query.fcgi?cmd=Search&db=Protein&term=7430017&doptcmdl=GenPept) | 2-succinyl-6-hydroxy-2,4-cyclohexadiene-1-carboxylate synthase (EC 4.1.3.-) [validated] - Bacillus subtilis | 134.16 | 4 | 554 1968 2019 2128 |  |  |
|  | [6166546](http://www.ncbi.nlm.nih.gov/entrez/query.fcgi?cmd=Search&db=Protein&term=6166546&doptcmdl=GenPept) | Menaquinone biosynthesis protein menD [Includes: 2-succinyl-6-hydroxy-2,4-cyclohexadiene-1-carboxylate synthase (SHCHC synthase); 2-oxoglutarate decarboxylase (Alpha-ketoglutarate decarboxylase) (KDC)] | 134.16 | 4 | 554 1968 2019 2128 |  |  |
|  | [2635566](http://www.ncbi.nlm.nih.gov/entrez/query.fcgi?cmd=Search&db=Protein&term=2635566&doptcmdl=GenPept) | 2-oxoglutarate decarboxylase and 2-succinyl-6-hydroxy-2,4-cyclohexadiene-1-carboxylate synthase [Bacillus subtilis subsp. subtilis str. 168] | 134.16 | 4 | 554 1968 2019 2128 |  |  |
|  | [2293146](http://www.ncbi.nlm.nih.gov/entrez/query.fcgi?cmd=Search&db=Protein&term=2293146&doptcmdl=GenPept) | SHCHC synthase [Bacillus subtilis] | 134.16 | 4 | 554 1968 2019 2128 |  |  |
|  | [16080134](http://www.ncbi.nlm.nih.gov/entrez/query.fcgi?cmd=Search&db=Protein&term=16080134&doptcmdl=GenPept) | 2-oxoglutarate decarboxylase and 2-succinyl-6-hydroxy-2,4-cyclohexadiene-1-carboxylate synthase [Bacillus subtilis] | 134.16 | 4 | 554 1968 2019 2128 |  |  |
| 265 | [16080335](http://www.ncbi.nlm.nih.gov/entrez/query.fcgi?cmd=Search&db=Protein&term=16080335&doptcmdl=GenPept) | acetyl-CoA acetyltransferase [Bacillus subtilis subsp. subtilis str. 168] | 132.25 | 3 | 619 1315 2071 |  |  |
| 266 | [16078090](http://www.ncbi.nlm.nih.gov/entrez/query.fcgi?cmd=Search&db=Protein&term=16078090&doptcmdl=GenPept) | epimerase [Bacillus subtilis subsp. subtilis str. 168] | 129.21 | 3 | 116 1410 1959 |  |  |
| 267 | [255767576](http://www.ncbi.nlm.nih.gov/entrez/query.fcgi?cmd=Search&db=Protein&term=255767576&doptcmdl=GenPept) | glycyl-tRNA synthetase subunit beta [Bacillus subtilis subsp. subtilis str. 168] | 129.20 | 3 | 260 882 2032 |  |  |
| 268 | [255767486](http://www.ncbi.nlm.nih.gov/entrez/query.fcgi?cmd=Search&db=Protein&term=255767486&doptcmdl=GenPept) | histidinol-phosphate aminotransferase [Bacillus subtilis subsp. subtilis str. 168] | 129.03 | 2 | 336 387 |  |  |
| 269 | [16080578](http://www.ncbi.nlm.nih.gov/entrez/query.fcgi?cmd=Search&db=Protein&term=16080578&doptcmdl=GenPept) | cell-division ABC transporter [Bacillus subtilis subsp. subtilis str. 168] | 128.49 | 2 | 1643 1689 |  |  |
| 270 | [16077106](http://www.ncbi.nlm.nih.gov/entrez/query.fcgi?cmd=Search&db=Protein&term=16077106&doptcmdl=GenPept) | methionyl-tRNA synthetase [Bacillus subtilis subsp. subtilis str. 168] | 128.45 | 1 | 44 |  |  |
| 271 | [16077074](http://www.ncbi.nlm.nih.gov/entrez/query.fcgi?cmd=Search&db=Protein&term=16077074&doptcmdl=GenPept) | DNA gyrase subunit B [Bacillus subtilis subsp. subtilis str. 168] | 127.91 | 3 | 88 265 418 |  |  |
| 272 | [16080582](http://www.ncbi.nlm.nih.gov/entrez/query.fcgi?cmd=Search&db=Protein&term=16080582&doptcmdl=GenPept) | peptide chain release factor 2 [Bacillus subtilis subsp. subtilis str. 168] | 127.23 | 3 | 668 851 1487 |  |  |
| 273 | [16080191](http://www.ncbi.nlm.nih.gov/entrez/query.fcgi?cmd=Search&db=Protein&term=16080191&doptcmdl=GenPept) | general stress protein 13 [Bacillus subtilis subsp. subtilis str. 168] | 126.98 | 2 | 937 963 |  |  |
| 274 | [16078306](http://www.ncbi.nlm.nih.gov/entrez/query.fcgi?cmd=Search&db=Protein&term=16078306&doptcmdl=GenPept) | hypothetical protein BSU12410 [Bacillus subtilis subsp. subtilis str. 168] | 126.89 | 2 | 1026 1378 |  |  |
| 275 | [255767029](http://www.ncbi.nlm.nih.gov/entrez/query.fcgi?cmd=Search&db=Protein&term=255767029&doptcmdl=GenPept) | 50S ribosomal protein L2 [Bacillus subtilis subsp. subtilis str. 168] | 126.69 | 3 | 464 525 1097 |  |  |
| 276 | [16078667](http://www.ncbi.nlm.nih.gov/entrez/query.fcgi?cmd=Search&db=Protein&term=16078667&doptcmdl=GenPept) | 50S ribosomal protein L19 [Bacillus subtilis subsp. subtilis str. 168] | 125.37 | 3 | 958 1177 1703 |  |  |
| 277 | [16078707](http://www.ncbi.nlm.nih.gov/entrez/query.fcgi?cmd=Search&db=Protein&term=16078707&doptcmdl=GenPept) | modulation of CheA activity in response to attractants (chemotaxis) [Bacillus subtilis subsp. subtilis str. 168] | 125.21 | 3 | 1392 1399 1446 |  |  |
| 278 | [16081142](http://www.ncbi.nlm.nih.gov/entrez/query.fcgi?cmd=Search&db=Protein&term=16081142&doptcmdl=GenPept) | single-strand DNA-binding protein [Bacillus subtilis subsp. subtilis str. 168] | 124.47 | 2 | 1699 2020 |  |  |
| 279 | [16077199](http://www.ncbi.nlm.nih.gov/entrez/query.fcgi?cmd=Search&db=Protein&term=16077199&doptcmdl=GenPept) | 50S ribosomal protein L6 [Bacillus subtilis subsp. subtilis str. 168] | 123.54 | 2 | 522 1376 |  |  |
| 280 | [16079466](http://www.ncbi.nlm.nih.gov/entrez/query.fcgi?cmd=Search&db=Protein&term=16079466&doptcmdl=GenPept) | transcriptional regulator [Bacillus subtilis subsp. subtilis str. 168] | 123.31 | 2 | 221 1418 |  |  |
| 281 | [16078676](http://www.ncbi.nlm.nih.gov/entrez/query.fcgi?cmd=Search&db=Protein&term=16078676&doptcmdl=GenPept) | tRNA (uracil-5-)-methyltransferase Gid [Bacillus subtilis subsp. subtilis str. 168] | 122.92 | 3 | 295 1763 1941 |  |  |
| 282 | [16079875](http://www.ncbi.nlm.nih.gov/entrez/query.fcgi?cmd=Search&db=Protein&term=16079875&doptcmdl=GenPept) | trigger factor [Bacillus subtilis subsp. subtilis str. 168] | 122.51 | 3 | 142 651 1050 |  |  |
| 283 | [16077184](http://www.ncbi.nlm.nih.gov/entrez/query.fcgi?cmd=Search&db=Protein&term=16077184&doptcmdl=GenPept) | 50S ribosomal protein L3 [Bacillus subtilis subsp. subtilis str. 168] | 122.26 | 2 | 1160 1930 |  |  |
| 284 | [255767047](http://www.ncbi.nlm.nih.gov/entrez/query.fcgi?cmd=Search&db=Protein&term=255767047&doptcmdl=GenPept) | hypothetical protein BSU01750 [Bacillus subtilis subsp. subtilis str. 168] | 121.76 | 2 | 1288 2139 |  |  |
| 285 | [16078453](http://www.ncbi.nlm.nih.gov/entrez/query.fcgi?cmd=Search&db=Protein&term=16078453&doptcmdl=GenPept) | phosphotransferase system (PTS) glucose-specific enzyme IICBA component [Bacillus subtilis subsp. subtilis str. 168] | 121.18 | 3 | 102 287 1356 |  |  |
| 286 | [16078069](http://www.ncbi.nlm.nih.gov/entrez/query.fcgi?cmd=Search&db=Protein&term=16078069&doptcmdl=GenPept) | ABC transporter membrane protein [Bacillus subtilis subsp. subtilis str. 168] | 120.70 | 3 | 139 1657 2101 |  |  |
| 287 | [16079368](http://www.ncbi.nlm.nih.gov/entrez/query.fcgi?cmd=Search&db=Protein&term=16079368&doptcmdl=GenPept) | two-component sensor histidine kinase [Bacillus subtilis subsp. subtilis str. 168] | 119.96 | 3 | 110 1171 1830 |  |  |
| 288 | [16079548](http://www.ncbi.nlm.nih.gov/entrez/query.fcgi?cmd=Search&db=Protein&term=16079548&doptcmdl=GenPept) | hypothetical protein BSU24930 [Bacillus subtilis subsp. subtilis str. 168] | 119.32 | 2 | 1078 1218 |  |  |
| 289 | [16080762](http://www.ncbi.nlm.nih.gov/entrez/query.fcgi?cmd=Search&db=Protein&term=16080762&doptcmdl=GenPept) | fructose 1,6-bisphosphatase II [Bacillus subtilis subsp. subtilis str. 168] | 119.03 | 2 | 1933 1998 |  |  |
| 290 | [255767500](http://www.ncbi.nlm.nih.gov/entrez/query.fcgi?cmd=Search&db=Protein&term=255767500&doptcmdl=GenPept) | thiol-disulfide oxidoreductase [Bacillus subtilis subsp. subtilis str. 168] | 116.41 | 2 | 109 1849 |  |  |
| 291 | [16080001](http://www.ncbi.nlm.nih.gov/entrez/query.fcgi?cmd=Search&db=Protein&term=16080001&doptcmdl=GenPept) | thiol peroxidase [Bacillus subtilis subsp. subtilis str. 168] | 116.24 | 2 | 48 1917 |  |  |
| 292 | [16078713](http://www.ncbi.nlm.nih.gov/entrez/query.fcgi?cmd=Search&db=Protein&term=16078713&doptcmdl=GenPept) | elongation factor Ts [Bacillus subtilis subsp. subtilis str. 168] | 114.57 | 2 | 182 1036 |  |  |
| 293 | [255767503](http://www.ncbi.nlm.nih.gov/entrez/query.fcgi?cmd=Search&db=Protein&term=255767503&doptcmdl=GenPept) | hypothetical protein BSU23328 [Bacillus subtilis subsp. subtilis str. 168] | 114.24 | 3 | 355 423 730 |  |  |
| 294 | [16080559](http://www.ncbi.nlm.nih.gov/entrez/query.fcgi?cmd=Search&db=Protein&term=16080559&doptcmdl=GenPept) | monooxygenase [Bacillus subtilis subsp. subtilis str. 168] | 114.14 | 3 | 974 1813 1924 |  |  |
| 295 | [16078436](http://www.ncbi.nlm.nih.gov/entrez/query.fcgi?cmd=Search&db=Protein&term=16078436&doptcmdl=GenPept) | pre-queuosine 0 synthase [Bacillus subtilis subsp. subtilis str. 168] | 113.62 | 2 | 1314 1320 |  |  |
| 296 | [16078655](http://www.ncbi.nlm.nih.gov/entrez/query.fcgi?cmd=Search&db=Protein&term=16078655&doptcmdl=GenPept) | acyl carrier protein [Bacillus subtilis subsp. subtilis str. 168] | 113.25 | 2 | 512 1710 |  |  |
| 297 | [16077193](http://www.ncbi.nlm.nih.gov/entrez/query.fcgi?cmd=Search&db=Protein&term=16077193&doptcmdl=GenPept) | 30S ribosomal protein S17 [Bacillus subtilis subsp. subtilis str. 168] | 113.12 | 3 | 65 704 1051 |  |  |
| 298 | [16077189](http://www.ncbi.nlm.nih.gov/entrez/query.fcgi?cmd=Search&db=Protein&term=16077189&doptcmdl=GenPept) | 50S ribosomal protein L22 [Bacillus subtilis subsp. subtilis str. 168] | 112.96 | 2 | 526 1730 |  |  |
| 299 | [16079602](http://www.ncbi.nlm.nih.gov/entrez/query.fcgi?cmd=Search&db=Protein&term=16079602&doptcmdl=GenPept) | heat shock protein GrpE [Bacillus subtilis subsp. subtilis str. 168] | 112.61 | 2 | 219 320 |  |  |
| 300 | [255767631](http://www.ncbi.nlm.nih.gov/entrez/query.fcgi?cmd=Search&db=Protein&term=255767631&doptcmdl=GenPept) | GTP pyrophosphokinase RelA/SpoT [Bacillus subtilis subsp. subtilis str. 168] | 112.25 | 2 | 1708 1846 |  |  |
| 301 | [7431580](http://www.ncbi.nlm.nih.gov/entrez/query.fcgi?cmd=Search&db=Protein&term=7431580&doptcmdl=GenPept) | 3-methyl-2-oxobutanoate dehydrogenase (lipoamide) (EC 1.2.4.4) E1 beta chain bfmBAB - Bacillus subtilis | 111.62 | 2 | 1225 1397 |  |  |
|  | [585607](http://www.ncbi.nlm.nih.gov/entrez/query.fcgi?cmd=Search&db=Protein&term=585607&doptcmdl=GenPept) | 2-oxoisovalerate dehydrogenase beta subunit (Branched-chain alpha-keto acid dehydrogenase E1 component beta chain) (BCKDH E1-beta) | 111.62 | 2 | 1225 1397 |  |  |
|  | [2634838](http://www.ncbi.nlm.nih.gov/entrez/query.fcgi?cmd=Search&db=Protein&term=2634838&doptcmdl=GenPept) | branched-chain alpha-keto acid dehydrogenase E1 (2-oxoisovalerate dehydrogenase beta subunit) [Bacillus subtilis subsp. subtilis str. 168] | 111.62 | 2 | 1225 1397 |  |  |
|  | [16079460](http://www.ncbi.nlm.nih.gov/entrez/query.fcgi?cmd=Search&db=Protein&term=16079460&doptcmdl=GenPept) | branched-chain alpha-keto acid dehydrogenase E1 (2-oxoisovalerate dehydrogenase beta subunit) [Bacillus subtilis] | 111.62 | 2 | 1225 1397 |  |  |
|  | [142612](http://www.ncbi.nlm.nih.gov/entrez/query.fcgi?cmd=Search&db=Protein&term=142612&doptcmdl=GenPept) | branched chain alpha-keto acid dehydrogenase E1-beta | 111.62 | 2 | 1225 1397 |  |  |
| 302 | [16081103](http://www.ncbi.nlm.nih.gov/entrez/query.fcgi?cmd=Search&db=Protein&term=16081103&doptcmdl=GenPept) | phosphodiesterase [Bacillus subtilis subsp. subtilis str. 168] | 111.14 | 2 | 28 1927 |  |  |
| 303 | [16077443](http://www.ncbi.nlm.nih.gov/entrez/query.fcgi?cmd=Search&db=Protein&term=16077443&doptcmdl=GenPept) | two-component response regulator YclK [Bacillus subtilis subsp. subtilis str. 168] | 111.11 | 3 | 201 1660 1978 |  |  |
| 304 | [16078695](http://www.ncbi.nlm.nih.gov/entrez/query.fcgi?cmd=Search&db=Protein&term=16078695&doptcmdl=GenPept) | flagellar motor switch protein [Bacillus subtilis subsp. subtilis str. 168] | 110.66 | 2 | 872 1458 |  |  |
| 305 | [16080094](http://www.ncbi.nlm.nih.gov/entrez/query.fcgi?cmd=Search&db=Protein&term=16080094&doptcmdl=GenPept) | ABC transporter ATP-binding protein [Bacillus subtilis subsp. subtilis str. 168] | 110.10 | 1 | 167 |  |  |
| 306 | [98474](http://www.ncbi.nlm.nih.gov/entrez/query.fcgi?cmd=Search&db=Protein&term=98474&doptcmdl=GenPept) | oligopeptide ABC transport system substrate-binding protein oppA precursor - Bacillus subtilis | 109.36 | 2 | 1033 1898 |  |  |
|  | [2633497](http://www.ncbi.nlm.nih.gov/entrez/query.fcgi?cmd=Search&db=Protein&term=2633497&doptcmdl=GenPept) | oligopeptide ABC transporter (binding protein) [Bacillus subtilis subsp. subtilis str. 168] | 109.36 | 2 | 1033 1898 |  |  |
|  | [16078208](http://www.ncbi.nlm.nih.gov/entrez/query.fcgi?cmd=Search&db=Protein&term=16078208&doptcmdl=GenPept) | oligopeptide ABC transporter (binding protein) [Bacillus subtilis] | 109.36 | 2 | 1033 1898 |  |  |
|  | [143603](http://www.ncbi.nlm.nih.gov/entrez/query.fcgi?cmd=Search&db=Protein&term=143603&doptcmdl=GenPept) | sporulation protein | 109.36 | 2 | 1033 1898 |  |  |
| 307 | [16080129](http://www.ncbi.nlm.nih.gov/entrez/query.fcgi?cmd=Search&db=Protein&term=16080129&doptcmdl=GenPept) | manganese ABC transporter manganese binding lipoprotein [Bacillus subtilis subsp. subtilis str. 168] | 109.18 | 2 | 196 492 |  |  |
| 308 | [255767600](http://www.ncbi.nlm.nih.gov/entrez/query.fcgi?cmd=Search&db=Protein&term=255767600&doptcmdl=GenPept) | hypothetical protein BSU26060 [Bacillus subtilis subsp. subtilis str. 168] | 108.88 | 2 | 507 1505 |  |  |
|  | [16078331](http://www.ncbi.nlm.nih.gov/entrez/query.fcgi?cmd=Search&db=Protein&term=16078331&doptcmdl=GenPept) | hypothetical protein BSU12660 [Bacillus subtilis subsp. subtilis str. 168] | 108.88 | 2 | 507 1505 |  |  |
| 309 | [255767180](http://www.ncbi.nlm.nih.gov/entrez/query.fcgi?cmd=Search&db=Protein&term=255767180&doptcmdl=GenPept) | hypothetical protein BSU06840 [Bacillus subtilis subsp. subtilis str. 168] | 108.24 | 2 | 361 572 |  |  |
| 310 | [16079487](http://www.ncbi.nlm.nih.gov/entrez/query.fcgi?cmd=Search&db=Protein&term=16079487&doptcmdl=GenPept) | bifunctional 5,10-methylene-tetrahydrofolate dehydrogenase/ 5,10-methylene-tetrahydrofolate cyclohydrolase [Bacillus subtilis subsp. subtilis str. 168] | 108.12 | 3 | 764 971 1990 |  |  |
| 311 | [16077105](http://www.ncbi.nlm.nih.gov/entrez/query.fcgi?cmd=Search&db=Protein&term=16077105&doptcmdl=GenPept) | transcriptional regulator for transition state genes [Bacillus subtilis subsp. subtilis str. 168] | 107.35 | 2 | 212 1479 |  |  |
| 312 | [16080899](http://www.ncbi.nlm.nih.gov/entrez/query.fcgi?cmd=Search&db=Protein&term=16080899&doptcmdl=GenPept) | (p)ppGpp synthetase [Bacillus subtilis subsp. subtilis str. 168] | 107.34 | 3 | 750 817 1024 |  |  |
| 313 | [16077194](http://www.ncbi.nlm.nih.gov/entrez/query.fcgi?cmd=Search&db=Protein&term=16077194&doptcmdl=GenPept) | 50S ribosomal protein L14 [Bacillus subtilis subsp. subtilis str. 168] | 107.05 | 2 | 1146 1462 |  |  |
| 314 | [16078602](http://www.ncbi.nlm.nih.gov/entrez/query.fcgi?cmd=Search&db=Protein&term=16078602&doptcmdl=GenPept) | hypothetical protein BSU15380 [Bacillus subtilis subsp. subtilis str. 168] | 106.99 | 2 | 880 945 |  |  |
| 315 | [16078016](http://www.ncbi.nlm.nih.gov/entrez/query.fcgi?cmd=Search&db=Protein&term=16078016&doptcmdl=GenPept) | negative regulator of the activity of sigma-M [Bacillus subtilis subsp. subtilis str. 168] | 106.86 | 2 | 127 1236 |  |  |
| 316 | [16078515](http://www.ncbi.nlm.nih.gov/entrez/query.fcgi?cmd=Search&db=Protein&term=16078515&doptcmdl=GenPept) | potassium uptake protein [Bacillus subtilis subsp. subtilis str. 168] | 106.35 | 3 | 642 1385 1502 |  |  |
| 317 | [255767084](http://www.ncbi.nlm.nih.gov/entrez/query.fcgi?cmd=Search&db=Protein&term=255767084&doptcmdl=GenPept) | L-lactate permease [Bacillus subtilis subsp. subtilis str. 168] | 106.04 | 1 | 1867 |  |  |
| 318 | [16079300](http://www.ncbi.nlm.nih.gov/entrez/query.fcgi?cmd=Search&db=Protein&term=16079300&doptcmdl=GenPept) | 3-methyl-2-oxobutanoate hydroxymethyltransferase [Bacillus subtilis subsp. subtilis str. 168] | 105.07 | 2 | 36 1349 |  |  |
| 319 | [16078584](http://www.ncbi.nlm.nih.gov/entrez/query.fcgi?cmd=Search&db=Protein&term=16078584&doptcmdl=GenPept) | UDP-N-acetylmuramoyl-L-alanyl-D-glutamate synthetase [Bacillus subtilis subsp. subtilis str. 168] | 103.95 | 2 | 630 2028 |  |  |
| 320 | [16077169](http://www.ncbi.nlm.nih.gov/entrez/query.fcgi?cmd=Search&db=Protein&term=16077169&doptcmdl=GenPept) | transcription antitermination protein NusG [Bacillus subtilis subsp. subtilis str. 168] | 103.43 | 3 | 784 1787 2126 |  |  |
| 321 | [255767590](http://www.ncbi.nlm.nih.gov/entrez/query.fcgi?cmd=Search&db=Protein&term=255767590&doptcmdl=GenPept) | shikimate 5-dehydrogenase [Bacillus subtilis subsp. subtilis str. 168] | 103.10 | 2 | 1107 2141 |  |  |
| 322 | [16080924](http://www.ncbi.nlm.nih.gov/entrez/query.fcgi?cmd=Search&db=Protein&term=16080924&doptcmdl=GenPept) | ABC membrane transporter ATP-binding protein [Bacillus subtilis subsp. subtilis str. 168] | 102.84 | 2 | 252 270 |  |  |
| 323 | [16077444](http://www.ncbi.nlm.nih.gov/entrez/query.fcgi?cmd=Search&db=Protein&term=16077444&doptcmdl=GenPept) | two-component sensor histidine kinase YclJ [Bacillus subtilis subsp. subtilis str. 168] | 102.51 | 2 | 233 922 |  |  |
| 324 | [16079844](http://www.ncbi.nlm.nih.gov/entrez/query.fcgi?cmd=Search&db=Protein&term=16079844&doptcmdl=GenPept) | GTPase ObgE [Bacillus subtilis subsp. subtilis str. 168] | 101.50 | 2 | 573 1925 |  |  |
| 325 | [16077428](http://www.ncbi.nlm.nih.gov/entrez/query.fcgi?cmd=Search&db=Protein&term=16077428&doptcmdl=GenPept) | cystine ABC transporter permease [Bacillus subtilis subsp. subtilis str. 168] | 100.37 | 2 | 119 217 |  |  |
| 326 | [16079312](http://www.ncbi.nlm.nih.gov/entrez/query.fcgi?cmd=Search&db=Protein&term=16079312&doptcmdl=GenPept) | cytochrome b6 [Bacillus subtilis subsp. subtilis str. 168] | 100.13 | 2 | 1273 1743 |  |  |
| 327 | [16078578](http://www.ncbi.nlm.nih.gov/entrez/query.fcgi?cmd=Search&db=Protein&term=16078578&doptcmdl=GenPept) | S-adenosyl-methyltransferase MraW [Bacillus subtilis subsp. subtilis str. 168] | 98.81 | 2 | 120 1818 |  |  |
| 328 | [16079809](http://www.ncbi.nlm.nih.gov/entrez/query.fcgi?cmd=Search&db=Protein&term=16079809&doptcmdl=GenPept) | aspartyl-tRNA synthetase [Bacillus subtilis subsp. subtilis str. 168] | 98.48 | 2 | 712 1656 |  |  |
| 329 | [732387](http://www.ncbi.nlm.nih.gov/entrez/query.fcgi?cmd=Search&db=Protein&term=732387&doptcmdl=GenPept) | Putative sugar phosphate isomerase ywlF | 98.26 | 3 | 400 422 1788 |  |  |
|  | [556884](http://www.ncbi.nlm.nih.gov/entrez/query.fcgi?cmd=Search&db=Protein&term=556884&doptcmdl=GenPept) | Similar to Escherichia coli yjcA gene product [Bacillus subtilis] | 98.26 | 3 | 400 422 1788 |  |  |
|  | [2636217](http://www.ncbi.nlm.nih.gov/entrez/query.fcgi?cmd=Search&db=Protein&term=2636217&doptcmdl=GenPept) | ywlF [Bacillus subtilis subsp. subtilis str. 168] | 98.26 | 3 | 400 422 1788 |  |  |
|  | [221325176](http://www.ncbi.nlm.nih.gov/entrez/query.fcgi?cmd=Search&db=Protein&term=221325176&doptcmdl=GenPept) | ribose-5-phosphate isomerase B [Bacillus subtilis subsp. subtilis str. SMY] | 98.26 | 3 | 400 422 1788 |  |  |
|  | [221320892](http://www.ncbi.nlm.nih.gov/entrez/query.fcgi?cmd=Search&db=Protein&term=221320892&doptcmdl=GenPept) | ribose-5-phosphate isomerase B [Bacillus subtilis subsp. subtilis str. JH642] | 98.26 | 3 | 400 422 1788 |  |  |
| 330 | [16078652](http://www.ncbi.nlm.nih.gov/entrez/query.fcgi?cmd=Search&db=Protein&term=16078652&doptcmdl=GenPept) | glycerol-3-phosphate acyltransferase PlsX [Bacillus subtilis subsp. subtilis str. 168] | 98.22 | 3 | 331 396 1764 |  |  |
| 331 | [7431562](http://www.ncbi.nlm.nih.gov/entrez/query.fcgi?cmd=Search&db=Protein&term=7431562&doptcmdl=GenPept) | 3-methyl-2-oxobutanoate dehydrogenase (lipoamide) (EC 1.2.4.4) E1 alpha chain bfmBAA - Bacillus subtilis | 97.95 | 2 | 255 283 |  |  |
|  | [585606](http://www.ncbi.nlm.nih.gov/entrez/query.fcgi?cmd=Search&db=Protein&term=585606&doptcmdl=GenPept) | 2-oxoisovalerate dehydrogenase alpha subunit (Branched-chain alpha-keto acid dehydrogenase E1 component alpha chain) (BCKDH E1-alpha) | 97.95 | 2 | 255 283 |  |  |
|  | [2634839](http://www.ncbi.nlm.nih.gov/entrez/query.fcgi?cmd=Search&db=Protein&term=2634839&doptcmdl=GenPept) | branched-chain alpha-keto acid dehydrogenase E1 (2-oxoisovalerate dehydrogenase alpha subunit) [Bacillus subtilis subsp. subtilis str. 168] | 97.95 | 2 | 255 283 |  |  |
|  | [16079461](http://www.ncbi.nlm.nih.gov/entrez/query.fcgi?cmd=Search&db=Protein&term=16079461&doptcmdl=GenPept) | branched-chain alpha-keto acid dehydrogenase E1 (2-oxoisovalerate dehydrogenase alpha subunit) [Bacillus subtilis] | 97.95 | 2 | 255 283 |  |  |
|  | [142611](http://www.ncbi.nlm.nih.gov/entrez/query.fcgi?cmd=Search&db=Protein&term=142611&doptcmdl=GenPept) | branched chain alpha-keto acid dehydrogenase E1-alpha | 97.95 | 2 | 255 283 |  |  |
| 332 | [16081153](http://www.ncbi.nlm.nih.gov/entrez/query.fcgi?cmd=Search&db=Protein&term=16081153&doptcmdl=GenPept) | tRNA uridine 5-carboxymethylaminomethyl modification enzyme GidA [Bacillus subtilis subsp. subtilis str. 168] | 96.52 | 3 | 553 762 1860 |  |  |
| 333 | [904201](http://www.ncbi.nlm.nih.gov/entrez/query.fcgi?cmd=Search&db=Protein&term=904201&doptcmdl=GenPept) | probable HtpG protein [Bacillus subtilis] | 96.36 | 3 | 644 1712 1931 |  |  |
|  | [7441890](http://www.ncbi.nlm.nih.gov/entrez/query.fcgi?cmd=Search&db=Protein&term=7441890&doptcmdl=GenPept) | class III heat-shock protein (chaperonin) htpG - Bacillus subtilis | 96.36 | 3 | 644 1712 1931 |  |  |
|  | [2636528](http://www.ncbi.nlm.nih.gov/entrez/query.fcgi?cmd=Search&db=Protein&term=2636528&doptcmdl=GenPept) | class III heat-shock protein (chaperonin) [Bacillus subtilis subsp. subtilis str. 168] | 96.36 | 3 | 644 1712 1931 |  |  |
|  | [16081033](http://www.ncbi.nlm.nih.gov/entrez/query.fcgi?cmd=Search&db=Protein&term=16081033&doptcmdl=GenPept) | class III heat-shock protein (chaperonin) [Bacillus subtilis] | 96.36 | 3 | 644 1712 1931 |  |  |
|  | [1170412](http://www.ncbi.nlm.nih.gov/entrez/query.fcgi?cmd=Search&db=Protein&term=1170412&doptcmdl=GenPept) | Chaperone protein htpG (Heat shock protein htpG) (High temperature protein G) | 96.36 | 3 | 644 1712 1931 |  |  |
| 334 | [16081143](http://www.ncbi.nlm.nih.gov/entrez/query.fcgi?cmd=Search&db=Protein&term=16081143&doptcmdl=GenPept) | 30S ribosomal protein S6 [Bacillus subtilis subsp. subtilis str. 168] | 95.63 | 3 | 239 639 1282 |  |  |
| 335 | [16077847](http://www.ncbi.nlm.nih.gov/entrez/query.fcgi?cmd=Search&db=Protein&term=16077847&doptcmdl=GenPept) | phosphotransferase system (PTS) trehalose-specific enzyme IIBC component [Bacillus subtilis subsp. subtilis str. 168] | 94.85 | 2 | 1030 1248 |  |  |
| 336 | [16078499](http://www.ncbi.nlm.nih.gov/entrez/query.fcgi?cmd=Search&db=Protein&term=16078499&doptcmdl=GenPept) | efflux permease [Bacillus subtilis subsp. subtilis str. 168] | 94.50 | 1 | 1286 |  |  |
| 337 | [50812277](http://www.ncbi.nlm.nih.gov/entrez/query.fcgi?cmd=Search&db=Protein&term=50812277&doptcmdl=GenPept) | Holliday junction DNA helicase RuvB [Bacillus subtilis subsp. subtilis str. 168] | 94.49 | 2 | 1864 2113 |  |  |
| 338 | [16079340](http://www.ncbi.nlm.nih.gov/entrez/query.fcgi?cmd=Search&db=Protein&term=16079340&doptcmdl=GenPept) | NAD(P)H-dependent glycerol-3-phosphate dehydrogenase [Bacillus subtilis subsp. subtilis str. 168] | 93.50 | 1 | 84 |  |  |
| 339 | [255767728](http://www.ncbi.nlm.nih.gov/entrez/query.fcgi?cmd=Search&db=Protein&term=255767728&doptcmdl=GenPept) | hypothetical protein BSU31910 [Bacillus subtilis subsp. subtilis str. 168] | 93.44 | 1 | 1172 |  |  |
| 340 | [16080235](http://www.ncbi.nlm.nih.gov/entrez/query.fcgi?cmd=Search&db=Protein&term=16080235&doptcmdl=GenPept) | short chain dehydrogenase [Bacillus subtilis subsp. subtilis str. 168] | 93.42 | 2 | 140 1858 |  |  |
| 341 | [16078797](http://www.ncbi.nlm.nih.gov/entrez/query.fcgi?cmd=Search&db=Protein&term=16078797&doptcmdl=GenPept) | RNA-binding protein Hfq [Bacillus subtilis subsp. subtilis str. 168] | 93.32 | 2 | 324 2036 |  |  |
| 342 | [255767025](http://www.ncbi.nlm.nih.gov/entrez/query.fcgi?cmd=Search&db=Protein&term=255767025&doptcmdl=GenPept) | 30S ribosomal protein S7 [Bacillus subtilis subsp. subtilis str. 168] | 93.24 | 2 | 1279 1317 |  |  |
| 343 | [81097391](http://www.ncbi.nlm.nih.gov/entrez/query.fcgi?cmd=Search&db=Protein&term=81097391&doptcmdl=GenPept) | Ribosomal protein L11, bacterial [Streptococcus suis 89/1591] | 92.83 | 2 | 866 1117 |  |  |
|  | [80976555](http://www.ncbi.nlm.nih.gov/entrez/query.fcgi?cmd=Search&db=Protein&term=80976555&doptcmdl=GenPept) | Ribosomal protein L11, bacterial [Streptococcus suis 89/1591] | 92.83 | 2 | 866 1117 |  |  |
|  | [284800542](http://www.ncbi.nlm.nih.gov/entrez/query.fcgi?cmd=Search&db=Protein&term=284800542&doptcmdl=GenPept) | 50S ribosomal protein L11 [Listeria monocytogenes 08-5578] | 92.83 | 2 | 866 1117 |  |  |
|  | [255767019](http://www.ncbi.nlm.nih.gov/entrez/query.fcgi?cmd=Search&db=Protein&term=255767019&doptcmdl=GenPept) | 50S ribosomal protein L11 [Bacillus subtilis subsp. subtilis str. 168] | 92.83 | 2 | 866 1117 |  |  |
|  | [166230936](http://www.ncbi.nlm.nih.gov/entrez/query.fcgi?cmd=Search&db=Protein&term=166230936&doptcmdl=GenPept) | 50S ribosomal protein L11 | 92.83 | 2 | 866 1117 |  |  |

| **Protein IDs*, cont.*** | | | | | | | |
| --- | --- | --- | --- | --- | --- | --- | --- |
| *Grp Nr.* | *Accession Number* | *Protein Name* | *Protein Score* | *Unique PSMs* | *PSM Serial Nrs.* | *Other Grp.* | *Score (other)* |
| 344 | [16078430](http://www.ncbi.nlm.nih.gov/entrez/query.fcgi?cmd=Search&db=Protein&term=16078430&doptcmdl=GenPept) | histidine kinase phosphorylating Spo0A [Bacillus subtilis subsp. subtilis str. 168] | 92.57 | 1 | 200 |  |  |
| 345 | [16079511](http://www.ncbi.nlm.nih.gov/entrez/query.fcgi?cmd=Search&db=Protein&term=16079511&doptcmdl=GenPept) | glycine dehydrogenase subunit 2 [Bacillus subtilis subsp. subtilis str. 168] | 92.54 | 2 | 895 1644 |  |  |
| 346 | [284802853](http://www.ncbi.nlm.nih.gov/entrez/query.fcgi?cmd=Search&db=Protein&term=284802853&doptcmdl=GenPept) | hypothetical protein LM5578_2610 [Listeria monocytogenes 08-5578] | 91.81 | 2 | 609 1428 |  |  |
|  | [16080323](http://www.ncbi.nlm.nih.gov/entrez/query.fcgi?cmd=Search&db=Protein&term=16080323&doptcmdl=GenPept) | sulfur mobilizing ABC protein, ATPase [Bacillus subtilis subsp. subtilis str. 168] | 91.81 | 2 | 609 1428 |  |  |
| 347 | [16080313](http://www.ncbi.nlm.nih.gov/entrez/query.fcgi?cmd=Search&db=Protein&term=16080313&doptcmdl=GenPept) | fructose amino acid-binding lipoprotein [Bacillus subtilis subsp. subtilis str. 168] | 90.92 | 2 | 245 1324 |  |  |
| 348 | [16080871](http://www.ncbi.nlm.nih.gov/entrez/query.fcgi?cmd=Search&db=Protein&term=16080871&doptcmdl=GenPept) | galactokinase [Bacillus subtilis subsp. subtilis str. 168] | 89.70 | 2 | 315 1427 |  |  |
| 349 | [16078064](http://www.ncbi.nlm.nih.gov/entrez/query.fcgi?cmd=Search&db=Protein&term=16078064&doptcmdl=GenPept) | hypothetical protein BSU10000 [Bacillus subtilis subsp. subtilis str. 168] | 88.20 | 2 | 898 1092 |  |  |
| 350 | [16080903](http://www.ncbi.nlm.nih.gov/entrez/query.fcgi?cmd=Search&db=Protein&term=16080903&doptcmdl=GenPept) | D-alanine--poly(phosphoribitol) ligase subunit 2 [Bacillus subtilis subsp. subtilis str. 168] | 87.96 | 2 | 98 111 |  |  |
| 351 | [255767547](http://www.ncbi.nlm.nih.gov/entrez/query.fcgi?cmd=Search&db=Protein&term=255767547&doptcmdl=GenPept) | elongation factor P [Bacillus subtilis subsp. subtilis str. 168] | 87.85 | 2 | 269 313 |  |  |
| 352 | [50812284](http://www.ncbi.nlm.nih.gov/entrez/query.fcgi?cmd=Search&db=Protein&term=50812284&doptcmdl=GenPept) | hypothetical protein BSU31400 [Bacillus subtilis subsp. subtilis str. 168] | 87.59 | 1 | 2072 |  |  |
| 353 | [16078080](http://www.ncbi.nlm.nih.gov/entrez/query.fcgi?cmd=Search&db=Protein&term=16078080&doptcmdl=GenPept) | methyl-accepting protein [Bacillus subtilis subsp. subtilis str. 168] | 86.41 | 2 | 616 1945 |  |  |
| 354 | [16077231](http://www.ncbi.nlm.nih.gov/entrez/query.fcgi?cmd=Search&db=Protein&term=16077231&doptcmdl=GenPept) | iron hydroxamate-binding lipoprotein [Bacillus subtilis subsp. subtilis str. 168] | 86.38 | 3 | 257 1120 1486 |  |  |
| 355 | [255767216](http://www.ncbi.nlm.nih.gov/entrez/query.fcgi?cmd=Search&db=Protein&term=255767216&doptcmdl=GenPept) | aminotransferase [Bacillus subtilis subsp. subtilis str. 168] | 86.27 | 2 | 1653 1798 |  |  |
| 356 | [16079789](http://www.ncbi.nlm.nih.gov/entrez/query.fcgi?cmd=Search&db=Protein&term=16079789&doptcmdl=GenPept) | hydrolase [Bacillus subtilis subsp. subtilis str. 168] | 84.97 | 2 | 444 1780 |  |  |
| 357 | [255767208](http://www.ncbi.nlm.nih.gov/entrez/query.fcgi?cmd=Search&db=Protein&term=255767208&doptcmdl=GenPept) | rRNA methylase [Bacillus subtilis subsp. subtilis str. 168] | 84.82 | 1 | 218 |  |  |
| 358 | [16079592](http://www.ncbi.nlm.nih.gov/entrez/query.fcgi?cmd=Search&db=Protein&term=16079592&doptcmdl=GenPept) | hypothetical protein BSU25380 [Bacillus subtilis subsp. subtilis str. 168] | 83.55 | 2 | 749 1336 |  |  |
| 359 | [81345682](http://www.ncbi.nlm.nih.gov/entrez/query.fcgi?cmd=Search&db=Protein&term=81345682&doptcmdl=GenPept) | RecName: Full=Putative phosphinothricin acetyltransferase ywnH; Short=PPT N-acetyltransferase | 83.06 | 1 | 155 |  |  |
|  | [2636181](http://www.ncbi.nlm.nih.gov/entrez/query.fcgi?cmd=Search&db=Protein&term=2636181&doptcmdl=GenPept) | ywnH [Bacillus subtilis subsp. subtilis str. 168] [MASS=18364] | 83.06 | 1 | 155 |  |  |
|  | [16080709](http://www.ncbi.nlm.nih.gov/entrez/query.fcgi?cmd=Search&db=Protein&term=16080709&doptcmdl=GenPept) | hypothetical protein BSU36560 [Bacillus subtilis subsp. subtilis str. 168] | 83.06 | 1 | 155 |  |  |
|  | [1592704](http://www.ncbi.nlm.nih.gov/entrez/query.fcgi?cmd=Search&db=Protein&term=1592704&doptcmdl=GenPept) | Unknown, similar to Streptomyces coelicolor phosphinothricin N-acetyltransferase [Bacillus subtilis] | 83.06 | 1 | 155 |  |  |
| 360 | [RS12_BACSU](http://us.expasy.org/uniprot/RS12_BACSU) | 30S ribosomal protein S12 (BS12) | 83.02 | 2 | 440 1068 |  |  |
|  | [58699021](http://www.ncbi.nlm.nih.gov/entrez/query.fcgi?cmd=Search&db=Protein&term=58699021&doptcmdl=GenPept) | ribosomal protein S12 [Wolbachia endosymbiont of Drosophila ananassae] | 61.09 | 1 | 1068 |  |  |
| 361 | [16079393](http://www.ncbi.nlm.nih.gov/entrez/query.fcgi?cmd=Search&db=Protein&term=16079393&doptcmdl=GenPept) | peptidyl-prolyl isomerase [Bacillus subtilis subsp. subtilis str. 168] | 83.02 | 2 | 1139 1425 |  |  |
| 362 | [255767493](http://www.ncbi.nlm.nih.gov/entrez/query.fcgi?cmd=Search&db=Protein&term=255767493&doptcmdl=GenPept) | FAD-dependent disulfide oxidoreductase [Bacillus subtilis subsp. subtilis str. 168] | 82.86 | 2 | 382 1524 |  |  |
| 363 | [16080867](http://www.ncbi.nlm.nih.gov/entrez/query.fcgi?cmd=Search&db=Protein&term=16080867&doptcmdl=GenPept) | cytochrome aa3-600 quinol oxidase subunit I [Bacillus subtilis subsp. subtilis str. 168] | 82.57 | 2 | 81 918 |  |  |
| 364 | [16079583](http://www.ncbi.nlm.nih.gov/entrez/query.fcgi?cmd=Search&db=Protein&term=16079583&doptcmdl=GenPept) | GTP-binding protein Era [Bacillus subtilis subsp. subtilis str. 168] | 82.56 | 2 | 93 1880 |  |  |
| 365 | [16078604](http://www.ncbi.nlm.nih.gov/entrez/query.fcgi?cmd=Search&db=Protein&term=16078604&doptcmdl=GenPept) | factor involved in shape determination [Bacillus subtilis subsp. subtilis str. 168] | 82.27 | 1 | 2061 |  |  |
| 366 | [16077203](http://www.ncbi.nlm.nih.gov/entrez/query.fcgi?cmd=Search&db=Protein&term=16077203&doptcmdl=GenPept) | 50S ribosomal protein L15 [Bacillus subtilis subsp. subtilis str. 168] | 81.95 | 2 | 97 208 |  |  |
| 367 | [16077079](http://www.ncbi.nlm.nih.gov/entrez/query.fcgi?cmd=Search&db=Protein&term=16077079&doptcmdl=GenPept) | pyridoxal biosynthesis lyase PdxS [Bacillus subtilis subsp. subtilis str. 168] | 81.86 | 2 | 38 825 |  |  |
| 368 | [16079603](http://www.ncbi.nlm.nih.gov/entrez/query.fcgi?cmd=Search&db=Protein&term=16079603&doptcmdl=GenPept) | heat-inducible transcription repressor [Bacillus subtilis subsp. subtilis str. 168] | 80.68 | 2 | 1790 1886 |  |  |
| 369 | [16079825](http://www.ncbi.nlm.nih.gov/entrez/query.fcgi?cmd=Search&db=Protein&term=16079825&doptcmdl=GenPept) | S-adenosylmethionine:tRNA ribosyltransferase-isomerase [Bacillus subtilis subsp. subtilis str. 168] | 80.49 | 2 | 375 1613 |  |  |
| 370 | [16080020](http://www.ncbi.nlm.nih.gov/entrez/query.fcgi?cmd=Search&db=Protein&term=16080020&doptcmdl=GenPept) | acetyl-CoA synthetase [Bacillus subtilis subsp. subtilis str. 168] | 80.28 | 3 | 323 1289 1294 |  |  |
| 371 | [50812224](http://www.ncbi.nlm.nih.gov/entrez/query.fcgi?cmd=Search&db=Protein&term=50812224&doptcmdl=GenPept) | phosphotransferase system (PTS) enzyme I [Bacillus subtilis subsp. subtilis str. 168] | 80.23 | 2 | 310 1224 |  |  |
| 372 | [284802890](http://www.ncbi.nlm.nih.gov/entrez/query.fcgi?cmd=Search&db=Protein&term=284802890&doptcmdl=GenPept) | preprotein translocase subunit SecG [Listeria monocytogenes 08-5578] | 80.05 | 1 | 1806 |  |  |
|  | [16080416](http://www.ncbi.nlm.nih.gov/entrez/query.fcgi?cmd=Search&db=Protein&term=16080416&doptcmdl=GenPept) | preprotein translocase subunit SecG [Bacillus subtilis subsp. subtilis str. 168] | 80.05 | 1 | 1806 |  |  |
| 373 | [16079419](http://www.ncbi.nlm.nih.gov/entrez/query.fcgi?cmd=Search&db=Protein&term=16079419&doptcmdl=GenPept) | NADPH-dependent aldo-keto reductase [Bacillus subtilis subsp. subtilis str. 168] | 79.59 | 1 | 2135 |  |  |
| 374 | [16080853](http://www.ncbi.nlm.nih.gov/entrez/query.fcgi?cmd=Search&db=Protein&term=16080853&doptcmdl=GenPept) | pyridoxal kinase [Bacillus subtilis subsp. subtilis str. 168] | 79.45 | 2 | 792 2004 |  |  |
| 375 | [255767741](http://www.ncbi.nlm.nih.gov/entrez/query.fcgi?cmd=Search&db=Protein&term=255767741&doptcmdl=GenPept) | hypothetical protein BSU32310 [Bacillus subtilis subsp. subtilis str. 168] | 78.60 | 1 | 1961 |  |  |
| 376 | [16078661](http://www.ncbi.nlm.nih.gov/entrez/query.fcgi?cmd=Search&db=Protein&term=16078661&doptcmdl=GenPept) | signal recognition particle-like (SRP) GTPase [Bacillus subtilis subsp. subtilis str. 168] | 78.45 | 1 | 1767 |  |  |
| 377 | [50812188](http://www.ncbi.nlm.nih.gov/entrez/query.fcgi?cmd=Search&db=Protein&term=50812188&doptcmdl=GenPept) | RNA helicase [Bacillus subtilis subsp. subtilis str. 168] | 78.42 | 2 | 1299 1607 |  |  |
| 378 | [255767377](http://www.ncbi.nlm.nih.gov/entrez/query.fcgi?cmd=Search&db=Protein&term=255767377&doptcmdl=GenPept) | aspartate kinase I [Bacillus subtilis subsp. subtilis str. 168] | 78.22 | 2 | 594 1940 |  |  |
| 379 | [16077524](http://www.ncbi.nlm.nih.gov/entrez/query.fcgi?cmd=Search&db=Protein&term=16077524&doptcmdl=GenPept) | UDP-N-acetylmuramoylalanyl-D-glutamyl-2, 6-diaminopimelate-D-alanyl-D-alanine ligase [Bacillus subtilis subsp. subtilis str. 168] | 78.09 | 2 | 873 1359 |  |  |
| 380 | [16080849](http://www.ncbi.nlm.nih.gov/entrez/query.fcgi?cmd=Search&db=Protein&term=16080849&doptcmdl=GenPept) | glycosyltransferase [Bacillus subtilis subsp. subtilis str. 168] | 76.87 | 2 | 66 385 |  |  |
| 381 | [255767153](http://www.ncbi.nlm.nih.gov/entrez/query.fcgi?cmd=Search&db=Protein&term=255767153&doptcmdl=GenPept) | ABC transporter ATP-binding protein [Bacillus subtilis subsp. subtilis str. 168] | 76.64 | 1 | 2035 |  |  |
| 382 | [16078067](http://www.ncbi.nlm.nih.gov/entrez/query.fcgi?cmd=Search&db=Protein&term=16078067&doptcmdl=GenPept) | Hit-family hydrolase [Bacillus subtilis subsp. subtilis str. 168] | 76.62 | 2 | 1523 1597 |  |  |
| 383 | [16077523](http://www.ncbi.nlm.nih.gov/entrez/query.fcgi?cmd=Search&db=Protein&term=16077523&doptcmdl=GenPept) | D-alanyl-alanine synthetase A [Bacillus subtilis subsp. subtilis str. 168] | 76.48 | 3 | 816 1409 1540 |  |  |
| 384 | [255767332](http://www.ncbi.nlm.nih.gov/entrez/query.fcgi?cmd=Search&db=Protein&term=255767332&doptcmdl=GenPept) | tetrahydrodipicolinate N-acetyltransferase [Bacillus subtilis subsp. subtilis str. 168] | 75.37 | 2 | 501 1254 |  |  |
| 385 | [255767319](http://www.ncbi.nlm.nih.gov/entrez/query.fcgi?cmd=Search&db=Protein&term=255767319&doptcmdl=GenPept) | hypothetical protein BSU13810 [Bacillus subtilis subsp. subtilis str. 168] | 75.00 | 2 | 909 1480 |  |  |
| 386 | [16077078](http://www.ncbi.nlm.nih.gov/entrez/query.fcgi?cmd=Search&db=Protein&term=16077078&doptcmdl=GenPept) | D-alanyl-D-alanine carboxypeptidase [Bacillus subtilis subsp. subtilis str. 168] | 74.85 | 2 | 583 934 |  |  |
| 387 | [16078512](http://www.ncbi.nlm.nih.gov/entrez/query.fcgi?cmd=Search&db=Protein&term=16078512&doptcmdl=GenPept) | transcriptional regulator [Bacillus subtilis subsp. subtilis str. 168] | 74.64 | 2 | 904 1800 |  |  |
| 388 | [16078615](http://www.ncbi.nlm.nih.gov/entrez/query.fcgi?cmd=Search&db=Protein&term=16078615&doptcmdl=GenPept) | carbamoyl phosphate synthase small subunit [Bacillus subtilis subsp. subtilis str. 168] | 74.64 | 2 | 1325 1812 |  |  |
| 389 | [255767358](http://www.ncbi.nlm.nih.gov/entrez/query.fcgi?cmd=Search&db=Protein&term=255767358&doptcmdl=GenPept) | isoleucyl-tRNA synthetase [Bacillus subtilis subsp. subtilis str. 168] | 74.38 | 2 | 692 1407 |  |  |
| 390 | [16077688](http://www.ncbi.nlm.nih.gov/entrez/query.fcgi?cmd=Search&db=Protein&term=16077688&doptcmdl=GenPept) | hypothetical protein BSU06210 [Bacillus subtilis subsp. subtilis str. 168] | 74.31 | 2 | 1337 1535 |  |  |
| 391 | [895752](http://www.ncbi.nlm.nih.gov/entrez/query.fcgi?cmd=Search&db=Protein&term=895752&doptcmdl=GenPept) | Unknown, highly similar to yeast TWT1 protein and to Mouse ECA39 protein, similar to amino acid aminotransferases [Bacillus subtilis] | 73.59 | 1 | 2066 |  |  |
|  | [2636390](http://www.ncbi.nlm.nih.gov/entrez/query.fcgi?cmd=Search&db=Protein&term=2636390&doptcmdl=GenPept) | ywaA [Bacillus subtilis subsp. subtilis str. 168] | 73.59 | 1 | 2066 |  |  |
|  | [255767817](http://www.ncbi.nlm.nih.gov/entrez/query.fcgi?cmd=Search&db=Protein&term=255767817&doptcmdl=GenPept) | branched-chain amino acid aminotransferase [Bacillus subtilis subsp. subtilis str. 168] | 73.59 | 1 | 2066 |  |  |
|  | [16080906](http://www.ncbi.nlm.nih.gov/entrez/query.fcgi?cmd=Search&db=Protein&term=16080906&doptcmdl=GenPept) | alternate gene name: ipa-0r~similar to branched-chain amino acid aminotransferase [Bacillus subtilis] | 73.59 | 1 | 2066 |  |  |
|  | [1361389](http://www.ncbi.nlm.nih.gov/entrez/query.fcgi?cmd=Search&db=Protein&term=1361389&doptcmdl=GenPept) | branched-chain amino acid aminotransferase homolog ywaA - Bacillus subtilis | 73.59 | 1 | 2066 |  |  |
| 392 | [16079266](http://www.ncbi.nlm.nih.gov/entrez/query.fcgi?cmd=Search&db=Protein&term=16079266&doptcmdl=GenPept) | metal-dependent carboxypeptidase [Bacillus subtilis subsp. subtilis str. 168] | 72.97 | 1 | 2089 |  |  |
| 393 | [16080133](http://www.ncbi.nlm.nih.gov/entrez/query.fcgi?cmd=Search&db=Protein&term=16080133&doptcmdl=GenPept) | esterase [Bacillus subtilis subsp. subtilis str. 168] | 72.84 | 1 | 79 |  |  |
| 394 | [16080039](http://www.ncbi.nlm.nih.gov/entrez/query.fcgi?cmd=Search&db=Protein&term=16080039&doptcmdl=GenPept) | hypothetical protein BSU29870 [Bacillus subtilis subsp. subtilis str. 168] | 72.77 | 1 | 124 |  |  |
| 395 | [ALBC_BACSU](http://us.expasy.org/uniprot/ALBC_BACSU) | Putative ABC transporter ATP-binding protein albC (Antilisterial bacteriocin subtilosin biosynthesis protein albC) | 72.72 | 2 | 135 1977 |  |  |
|  | [16080791](http://www.ncbi.nlm.nih.gov/entrez/query.fcgi?cmd=Search&db=Protein&term=16080791&doptcmdl=GenPept) | transporter involved in subtilosin production [Bacillus subtilis subsp. subtilis str. 168] | 51.21 | 1 | 135 |  |  |
| 396 | [255767048](http://www.ncbi.nlm.nih.gov/entrez/query.fcgi?cmd=Search&db=Protein&term=255767048&doptcmdl=GenPept) | hypothetical protein BSU01760 [Bacillus subtilis subsp. subtilis str. 168] | 72.51 | 1 | 892 |  |  |
| 397 | [16078668](http://www.ncbi.nlm.nih.gov/entrez/query.fcgi?cmd=Search&db=Protein&term=16078668&doptcmdl=GenPept) | ribosomal biogenesis GTPase [Bacillus subtilis subsp. subtilis str. 168] | 72.29 | 1 | 131 |  |  |
| 398 | [16077803](http://www.ncbi.nlm.nih.gov/entrez/query.fcgi?cmd=Search&db=Protein&term=16077803&doptcmdl=GenPept) | chemotaxis sensory transducer [Bacillus subtilis subsp. subtilis str. 168] | 71.36 | 2 | 53 716 |  |  |
| 399 | [16078288](http://www.ncbi.nlm.nih.gov/entrez/query.fcgi?cmd=Search&db=Protein&term=16078288&doptcmdl=GenPept) | uroporphyrinogen-III synthase [Bacillus subtilis subsp. subtilis str. 168] | 71.15 | 1 | 698 |  |  |
| 400 | [255767587](http://www.ncbi.nlm.nih.gov/entrez/query.fcgi?cmd=Search&db=Protein&term=255767587&doptcmdl=GenPept) | coproporphyrinogen III oxidase [Bacillus subtilis subsp. subtilis str. 168] | 70.36 | 1 | 41 |  |  |
| 401 | [16081156](http://www.ncbi.nlm.nih.gov/entrez/query.fcgi?cmd=Search&db=Protein&term=16081156&doptcmdl=GenPept) | OxaA-like protein precursor [Bacillus subtilis subsp. subtilis str. 168] | 69.97 | 1 | 209 |  |  |
| 402 | [16077136](http://www.ncbi.nlm.nih.gov/entrez/query.fcgi?cmd=Search&db=Protein&term=16077136&doptcmdl=GenPept) | hypoxanthine-guanine phosphoribosyltransferase [Bacillus subtilis subsp. subtilis str. 168] | 69.84 | 2 | 718 1879 |  |  |
| 403 | [16077123](http://www.ncbi.nlm.nih.gov/entrez/query.fcgi?cmd=Search&db=Protein&term=16077123&doptcmdl=GenPept) | transcription-repair coupling factor [Bacillus subtilis subsp. subtilis str. 168] | 69.76 | 2 | 1393 1752 |  |  |
| 404 | [255767028](http://www.ncbi.nlm.nih.gov/entrez/query.fcgi?cmd=Search&db=Protein&term=255767028&doptcmdl=GenPept) | 50S ribosomal protein L23 [Bacillus subtilis subsp. subtilis str. 168] | 69.61 | 2 | 1740 1814 |  |  |
| 405 | [16080377](http://www.ncbi.nlm.nih.gov/entrez/query.fcgi?cmd=Search&db=Protein&term=16080377&doptcmdl=GenPept) | oxalate decarboxylase [Bacillus subtilis subsp. subtilis str. 168] | 69.01 | 2 | 482 822 |  |  |
| 406 | [16078721](http://www.ncbi.nlm.nih.gov/entrez/query.fcgi?cmd=Search&db=Protein&term=16078721&doptcmdl=GenPept) | DNA polymerase III PolC [Bacillus subtilis subsp. subtilis str. 168] | 68.65 | 2 | 59 1408 |  |  |
| 407 | [16077185](http://www.ncbi.nlm.nih.gov/entrez/query.fcgi?cmd=Search&db=Protein&term=16077185&doptcmdl=GenPept) | 50S ribosomal protein L4 [Bacillus subtilis subsp. subtilis str. 168] | 68.62 | 1 | 2047 |  |  |
| 408 | [16080737](http://www.ncbi.nlm.nih.gov/entrez/query.fcgi?cmd=Search&db=Protein&term=16080737&doptcmdl=GenPept) | F0F1 ATP synthase subunit delta [Bacillus subtilis subsp. subtilis str. 168] | 68.52 | 2 | 574 1953 |  |  |
| 409 | [16080531](http://www.ncbi.nlm.nih.gov/entrez/query.fcgi?cmd=Search&db=Protein&term=16080531&doptcmdl=GenPept) | triphosphate pyrophosphate hydrolase [Bacillus subtilis subsp. subtilis str. 168] | 68.49 | 1 | 411 |  |  |
| 410 | [255767349](http://www.ncbi.nlm.nih.gov/entrez/query.fcgi?cmd=Search&db=Protein&term=255767349&doptcmdl=GenPept) | undecaprenyldiphospho-muramoylpentapeptide beta-N- acetylglucosaminyltransferase [Bacillus subtilis subsp. subtilis str. 168] | 68.30 | 2 | 1233 1309 |  |  |
| 411 | [255767197](http://www.ncbi.nlm.nih.gov/entrez/query.fcgi?cmd=Search&db=Protein&term=255767197&doptcmdl=GenPept) | glutamate-1-semialdehyde aminotransferase [Bacillus subtilis subsp. subtilis str. 168] | 68.27 | 1 | 612 |  |  |
| 412 | [16078507](http://www.ncbi.nlm.nih.gov/entrez/query.fcgi?cmd=Search&db=Protein&term=16078507&doptcmdl=GenPept) | ABC efflux transporter ATP-binding protein [Bacillus subtilis subsp. subtilis str. 168] | 68.10 | 2 | 1045 1664 |  |  |
| 413 | [16077848](http://www.ncbi.nlm.nih.gov/entrez/query.fcgi?cmd=Search&db=Protein&term=16077848&doptcmdl=GenPept) | trehalose-6-phosphate hydrolase [Bacillus subtilis subsp. subtilis str. 168] | 67.87 | 2 | 161 1781 |  |  |
| 414 | [161511066](http://www.ncbi.nlm.nih.gov/entrez/query.fcgi?cmd=Search&db=Protein&term=161511066&doptcmdl=GenPept) | heme peroxidase [Bacillus subtilis subsp. subtilis str. 168] | 66.06 | 2 | 1684 1872 |  |  |
| 415 | [255767372](http://www.ncbi.nlm.nih.gov/entrez/query.fcgi?cmd=Search&db=Protein&term=255767372&doptcmdl=GenPept) | flagellar biosynthesis protein FlhA [Bacillus subtilis subsp. subtilis str. 168] | 65.37 | 1 | 234 |  |  |
| 416 | [16079280](http://www.ncbi.nlm.nih.gov/entrez/query.fcgi?cmd=Search&db=Protein&term=16079280&doptcmdl=GenPept) | ATP-dependent helicase [Bacillus subtilis subsp. subtilis str. 168] | 64.43 | 2 | 63 1948 |  |  |
| 417 | [255767345](http://www.ncbi.nlm.nih.gov/entrez/query.fcgi?cmd=Search&db=Protein&term=255767345&doptcmdl=GenPept) | nucleoid associated protein [Bacillus subtilis subsp. subtilis str. 168] | 63.99 | 1 | 2045 |  |  |
| 418 | [16079317](http://www.ncbi.nlm.nih.gov/entrez/query.fcgi?cmd=Search&db=Protein&term=16079317&doptcmdl=GenPept) | 3-phosphoshikimate 1-carboxyvinyltransferase [Bacillus subtilis subsp. subtilis str. 168] | 63.70 | 1 | 2021 |  |  |
| 419 | [255767021](http://www.ncbi.nlm.nih.gov/entrez/query.fcgi?cmd=Search&db=Protein&term=255767021&doptcmdl=GenPept) | 50S ribosomal protein L10 [Bacillus subtilis subsp. subtilis str. 168] | 63.51 | 2 | 618 835 |  |  |
| 420 | [16077082](http://www.ncbi.nlm.nih.gov/entrez/query.fcgi?cmd=Search&db=Protein&term=16077082&doptcmdl=GenPept) | deoxyadenosine/deoxycytidine kinase [Bacillus subtilis subsp. subtilis str. 168] | 63.11 | 1 | 1947 |  |  |
| 421 | [16079962](http://www.ncbi.nlm.nih.gov/entrez/query.fcgi?cmd=Search&db=Protein&term=16079962&doptcmdl=GenPept) | two-component sensor histidine kinase [Bacillus subtilis subsp. subtilis str. 168] | 62.09 | 1 | 205 |  |  |
| 422 | [16079371](http://www.ncbi.nlm.nih.gov/entrez/query.fcgi?cmd=Search&db=Protein&term=16079371&doptcmdl=GenPept) | factor required for cytochrome c synthesis [Bacillus subtilis subsp. subtilis str. 168] | 61.41 | 1 | 2099 |  |  |
| 423 | [255767526](http://www.ncbi.nlm.nih.gov/entrez/query.fcgi?cmd=Search&db=Protein&term=255767526&doptcmdl=GenPept) | dihydrolipoamide dehydrogenase [Bacillus subtilis subsp. subtilis str. 168] | 60.81 | 1 | 2142 |  |  |
| 424 | [16080604](http://www.ncbi.nlm.nih.gov/entrez/query.fcgi?cmd=Search&db=Protein&term=16080604&doptcmdl=GenPept) | hypothetical protein BSU35510 [Bacillus subtilis subsp. subtilis str. 168] | 60.73 | 1 | 1108 |  |  |
| 425 | [16078251](http://www.ncbi.nlm.nih.gov/entrez/query.fcgi?cmd=Search&db=Protein&term=16078251&doptcmdl=GenPept) | hydrolase [Bacillus subtilis subsp. subtilis str. 168] | 60.28 | 2 | 10 1321 |  |  |
| 426 | [255767764](http://www.ncbi.nlm.nih.gov/entrez/query.fcgi?cmd=Search&db=Protein&term=255767764&doptcmdl=GenPept) | copper(I)-transporting ATPase [Bacillus subtilis subsp. subtilis str. 168] | 60.23 | 1 | 1093 |  |  |
| 427 | [16080458](http://www.ncbi.nlm.nih.gov/entrez/query.fcgi?cmd=Search&db=Protein&term=16080458&doptcmdl=GenPept) | iron-sulfur heterodisulfide reductase [Bacillus subtilis subsp. subtilis str. 168] | 59.87 | 1 | 879 |  |  |
| 428 | [16080310](http://www.ncbi.nlm.nih.gov/entrez/query.fcgi?cmd=Search&db=Protein&term=16080310&doptcmdl=GenPept) | fructoselysine kinase [Bacillus subtilis subsp. subtilis str. 168] | 59.58 | 1 | 465 |  |  |
| 429 | [16080000](http://www.ncbi.nlm.nih.gov/entrez/query.fcgi?cmd=Search&db=Protein&term=16080000&doptcmdl=GenPept) | nucleic acid methyltransferase [Bacillus subtilis subsp. subtilis str. 168] | 59.38 | 2 | 984 1421 |  |  |
| 430 | [16080035](http://www.ncbi.nlm.nih.gov/entrez/query.fcgi?cmd=Search&db=Protein&term=16080035&doptcmdl=GenPept) | hypothetical protein BSU29830 [Bacillus subtilis subsp. subtilis str. 168] | 59.05 | 1 | 35 |  |  |
| 431 | [255767605](http://www.ncbi.nlm.nih.gov/entrez/query.fcgi?cmd=Search&db=Protein&term=255767605&doptcmdl=GenPept) | phage capsid protein; skin element [Bacillus subtilis subsp. subtilis str. 168] | 58.92 | 2 | 358 1395 |  |  |
|  | [16078326](http://www.ncbi.nlm.nih.gov/entrez/query.fcgi?cmd=Search&db=Protein&term=16078326&doptcmdl=GenPept) | capsid protein of PBSX prophage [Bacillus subtilis subsp. subtilis str. 168] | 58.92 | 2 | 358 1395 |  |  |
| 432 | [255767034](http://www.ncbi.nlm.nih.gov/entrez/query.fcgi?cmd=Search&db=Protein&term=255767034&doptcmdl=GenPept) | 30S ribosomal protein S13 [Bacillus subtilis subsp. subtilis str. 168] | 58.91 | 2 | 648 1039 |  |  |
| 433 | [50812297](http://www.ncbi.nlm.nih.gov/entrez/query.fcgi?cmd=Search&db=Protein&term=50812297&doptcmdl=GenPept) | gluconeogenesis factor [Bacillus subtilis subsp. subtilis str. 168] | 58.82 | 1 | 45 |  |  |
| 434 | [255767262](http://www.ncbi.nlm.nih.gov/entrez/query.fcgi?cmd=Search&db=Protein&term=255767262&doptcmdl=GenPept) | thiol management oxidoreductase component [Bacillus subtilis subsp. subtilis str. 168] | 58.68 | 1 | 677 |  |  |

| **Protein IDs*, cont.*** | | | | | | | |
| --- | --- | --- | --- | --- | --- | --- | --- |
| *Grp Nr.* | *Accession Number* | *Protein Name* | *Protein Score* | *Unique PSMs* | *PSM Serial Nrs.* | *Other Grp.* | *Score (other)* |
| 435 | [16080386](http://www.ncbi.nlm.nih.gov/entrez/query.fcgi?cmd=Search&db=Protein&term=16080386&doptcmdl=GenPept) | ferrichrome ABC transporter ferrichrome-binding lipoprotein [Bacillus subtilis subsp. subtilis str. 168] | 58.66 | 1 | 1799 |  |  |
| 436 | [16077931](http://www.ncbi.nlm.nih.gov/entrez/query.fcgi?cmd=Search&db=Protein&term=16077931&doptcmdl=GenPept) | enoyl-(acyl carrier protein) reductase [Bacillus subtilis subsp. subtilis str. 168] | 58.54 | 1 | 705 |  |  |
| 437 | [16079777](http://www.ncbi.nlm.nih.gov/entrez/query.fcgi?cmd=Search&db=Protein&term=16079777&doptcmdl=GenPept) | hypothetical protein BSU27230 [Bacillus subtilis subsp. subtilis str. 168] | 58.53 | 2 | 46 1914 |  |  |
| 438 | [16077191](http://www.ncbi.nlm.nih.gov/entrez/query.fcgi?cmd=Search&db=Protein&term=16077191&doptcmdl=GenPept) | 50S ribosomal protein L16 [Bacillus subtilis subsp. subtilis str. 168] | 57.93 | 1 | 1748 |  |  |
| 439 | [16078413](http://www.ncbi.nlm.nih.gov/entrez/query.fcgi?cmd=Search&db=Protein&term=16078413&doptcmdl=GenPept) | heat shock protein HtpX [Bacillus subtilis subsp. subtilis str. 168] | 57.27 | 1 | 679 |  |  |
| 440 | [16079587](http://www.ncbi.nlm.nih.gov/entrez/query.fcgi?cmd=Search&db=Protein&term=16079587&doptcmdl=GenPept) | membrane associate hydrolase [Bacillus subtilis subsp. subtilis str. 168] | 57.23 | 1 | 1442 |  |  |
| 441 | [16078611](http://www.ncbi.nlm.nih.gov/entrez/query.fcgi?cmd=Search&db=Protein&term=16078611&doptcmdl=GenPept) | bifunctional pyrimidine regulatory protein PyrR uracil phosphoribosyltransferase [Bacillus subtilis subsp. subtilis str. 168] | 56.85 | 1 | 1022 |  |  |
| 442 | [16078654](http://www.ncbi.nlm.nih.gov/entrez/query.fcgi?cmd=Search&db=Protein&term=16078654&doptcmdl=GenPept) | beta-ketoacyl-acyl carrier protein reductase [Bacillus subtilis subsp. subtilis str. 168] | 56.82 | 1 | 1693 |  |  |
| 443 | [255767727](http://www.ncbi.nlm.nih.gov/entrez/query.fcgi?cmd=Search&db=Protein&term=255767727&doptcmdl=GenPept) | cell division protein [Bacillus subtilis subsp. subtilis str. 168] | 56.54 | 1 | 427 |  |  |
| 444 | [16079815](http://www.ncbi.nlm.nih.gov/entrez/query.fcgi?cmd=Search&db=Protein&term=16079815&doptcmdl=GenPept) | adenine phosphoribosyltransferase [Bacillus subtilis subsp. subtilis str. 168] | 56.49 | 1 | 1054 |  |  |
| 445 | [16079610](http://www.ncbi.nlm.nih.gov/entrez/query.fcgi?cmd=Search&db=Protein&term=16079610&doptcmdl=GenPept) | DNA polymerase III subunit delta [Bacillus subtilis subsp. subtilis str. 168] | 56.31 | 1 | 151 |  |  |
| 446 | [255767561](http://www.ncbi.nlm.nih.gov/entrez/query.fcgi?cmd=Search&db=Protein&term=255767561&doptcmdl=GenPept) | 50S ribosomal protein L33 [Bacillus subtilis subsp. subtilis str. 168] | 55.95 | 1 | 907 |  |  |
| 447 | [7442853](http://www.ncbi.nlm.nih.gov/entrez/query.fcgi?cmd=Search&db=Protein&term=7442853&doptcmdl=GenPept) | ribose ABC transporter (ribose-binding protein) rbsB - Bacillus subtilis | 55.49 | 1 | 556 |  |  |
|  | [6174949](http://www.ncbi.nlm.nih.gov/entrez/query.fcgi?cmd=Search&db=Protein&term=6174949&doptcmdl=GenPept) | D-ribose-binding protein precursor | 55.49 | 1 | 556 |  |  |
|  | [2636121](http://www.ncbi.nlm.nih.gov/entrez/query.fcgi?cmd=Search&db=Protein&term=2636121&doptcmdl=GenPept) | ribose ABC transporter (ribose-binding protein) [Bacillus subtilis subsp. subtilis str. 168] | 55.49 | 1 | 556 |  |  |
|  | [1894757](http://www.ncbi.nlm.nih.gov/entrez/query.fcgi?cmd=Search&db=Protein&term=1894757&doptcmdl=GenPept) | periplasmic substrate-binding protein [Bacillus subtilis] | 55.49 | 1 | 556 |  |  |
|  | [16080649](http://www.ncbi.nlm.nih.gov/entrez/query.fcgi?cmd=Search&db=Protein&term=16080649&doptcmdl=GenPept) | ribose ABC transporter (ribose-binding protein) [Bacillus subtilis] | 55.49 | 1 | 556 |  |  |
| 448 | [16079568](http://www.ncbi.nlm.nih.gov/entrez/query.fcgi?cmd=Search&db=Protein&term=16079568&doptcmdl=GenPept) | endonuclease IV [Bacillus subtilis subsp. subtilis str. 168] | 55.44 | 1 | 1157 |  |  |
| 449 | [16080130](http://www.ncbi.nlm.nih.gov/entrez/query.fcgi?cmd=Search&db=Protein&term=16080130&doptcmdl=GenPept) | O-succinylbenzoate-CoA synthase [Bacillus subtilis subsp. subtilis str. 168] | 54.80 | 2 | 723 1972 |  |  |
| 450 | [16080282](http://www.ncbi.nlm.nih.gov/entrez/query.fcgi?cmd=Search&db=Protein&term=16080282&doptcmdl=GenPept) | p-nitrophenyl phosphatase [Bacillus subtilis subsp. subtilis str. 168] | 54.57 | 1 | 1296 |  |  |
| 451 | [16080653](http://www.ncbi.nlm.nih.gov/entrez/query.fcgi?cmd=Search&db=Protein&term=16080653&doptcmdl=GenPept) | alpha-acetolactate decarboxylase [Bacillus subtilis subsp. subtilis str. 168] | 54.21 | 1 | 2012 |  |  |
| 452 | [16078719](http://www.ncbi.nlm.nih.gov/entrez/query.fcgi?cmd=Search&db=Protein&term=16078719&doptcmdl=GenPept) | inner membrane zinc metalloprotease [Bacillus subtilis subsp. subtilis str. 168] | 54.14 | 1 | 1738 |  |  |
| 453 | [255767641](http://www.ncbi.nlm.nih.gov/entrez/query.fcgi?cmd=Search&db=Protein&term=255767641&doptcmdl=GenPept) | rod shape-determining protein MreC [Bacillus subtilis subsp. subtilis str. 168] | 54.12 | 2 | 1542 1721 |  |  |
| 454 | [971344](http://www.ncbi.nlm.nih.gov/entrez/query.fcgi?cmd=Search&db=Protein&term=971344&doptcmdl=GenPept) | nitrate reductase gamma subunit [Bacillus subtilis] | 54.07 | 2 | 1322 1444 |  |  |
|  | [2636262](http://www.ncbi.nlm.nih.gov/entrez/query.fcgi?cmd=Search&db=Protein&term=2636262&doptcmdl=GenPept) | nitrate reductase (gamma subunit) [Bacillus subtilis subsp. subtilis str. 168] | 54.07 | 2 | 1322 1444 |  |  |
|  | [2127148](http://www.ncbi.nlm.nih.gov/entrez/query.fcgi?cmd=Search&db=Protein&term=2127148&doptcmdl=GenPept) | nitrate reductase (EC 1.7.99.4) gamma chain NarI - Bacillus subtilis | 54.07 | 2 | 1322 1444 |  |  |
|  | [16080778](http://www.ncbi.nlm.nih.gov/entrez/query.fcgi?cmd=Search&db=Protein&term=16080778&doptcmdl=GenPept) | nitrate reductase (gamma subunit) [Bacillus subtilis] | 54.07 | 2 | 1322 1444 |  |  |
|  | [1171653](http://www.ncbi.nlm.nih.gov/entrez/query.fcgi?cmd=Search&db=Protein&term=1171653&doptcmdl=GenPept) | Nitrate reductase gamma chain | 54.07 | 2 | 1322 1444 |  |  |
| 455 | [NUSA_BACSU](http://us.expasy.org/uniprot/NUSA_BACSU) | Transcription elongation protein nusA | 54.01 | 1 | 137 |  |  |
| 456 | [16077888](http://www.ncbi.nlm.nih.gov/entrez/query.fcgi?cmd=Search&db=Protein&term=16077888&doptcmdl=GenPept) | maltose and maltodextrin ABC transporter subunit ATP-binding protein [Bacillus subtilis subsp. subtilis str. 168] | 53.86 | 1 | 1910 |  |  |
| 457 | [16078647](http://www.ncbi.nlm.nih.gov/entrez/query.fcgi?cmd=Search&db=Protein&term=16078647&doptcmdl=GenPept) | dihydroxyacetone/glyceraldehyde kinase [Bacillus subtilis subsp. subtilis str. 168] | 53.81 | 2 | 1552 1902 |  |  |
| 458 | [16079355](http://www.ncbi.nlm.nih.gov/entrez/query.fcgi?cmd=Search&db=Protein&term=16079355&doptcmdl=GenPept) | phosphoesterase [Bacillus subtilis subsp. subtilis str. 168] | 53.79 | 2 | 1010 1264 |  |  |
| 459 | [16077368](http://www.ncbi.nlm.nih.gov/entrez/query.fcgi?cmd=Search&db=Protein&term=16077368&doptcmdl=GenPept) | glycine betaine ABC transporter permease [Bacillus subtilis subsp. subtilis str. 168] | 53.70 | 1 | 2030 |  |  |
| 460 | [16078658](http://www.ncbi.nlm.nih.gov/entrez/query.fcgi?cmd=Search&db=Protein&term=16078658&doptcmdl=GenPept) | signal recognition particle (docking protein) [Bacillus subtilis subsp. subtilis str. 168] | 53.61 | 2 | 773 1751 |  |  |
| 461 | [16078762](http://www.ncbi.nlm.nih.gov/entrez/query.fcgi?cmd=Search&db=Protein&term=16078762&doptcmdl=GenPept) | L-threonine 3-dehydrogenase [Bacillus subtilis subsp. subtilis str. 168] | 53.41 | 1 | 1201 |  |  |
| 462 | [16078850](http://www.ncbi.nlm.nih.gov/entrez/query.fcgi?cmd=Search&db=Protein&term=16078850&doptcmdl=GenPept) | cell division protein [Bacillus subtilis subsp. subtilis str. 168] | 52.88 | 1 | 174 |  |  |
| 463 | [255767286](http://www.ncbi.nlm.nih.gov/entrez/query.fcgi?cmd=Search&db=Protein&term=255767286&doptcmdl=GenPept) | phage capsid protein [Bacillus subtilis subsp. subtilis str. 168] | 52.69 | 1 | 624 |  |  |
| 464 | [7433705](http://www.ncbi.nlm.nih.gov/entrez/query.fcgi?cmd=Search&db=Protein&term=7433705&doptcmdl=GenPept) | glycine C-acetyltransferase (EC 2.3.1.29) kbl - Bacillus subtilis | 52.23 | 2 | 571 585 |  |  |
|  | [6685543](http://www.ncbi.nlm.nih.gov/entrez/query.fcgi?cmd=Search&db=Protein&term=6685543&doptcmdl=GenPept) | 2-amino-3-ketobutyrate coenzyme A ligase (AKB ligase) (Glycine acetyltransferase) | 52.23 | 2 | 571 585 |  |  |
|  | [2634072](http://www.ncbi.nlm.nih.gov/entrez/query.fcgi?cmd=Search&db=Protein&term=2634072&doptcmdl=GenPept) | 2-amino-3-ketobutyrate CoA ligase (glycine acetyl transferase) [Bacillus subtilis subsp. subtilis str. 168] | 52.23 | 2 | 571 585 |  |  |
|  | [16078763](http://www.ncbi.nlm.nih.gov/entrez/query.fcgi?cmd=Search&db=Protein&term=16078763&doptcmdl=GenPept) | 2-amino-3-ketobutyrate CoA ligase (glycine acetyl transferase) [Bacillus subtilis] | 52.23 | 2 | 571 585 |  |  |
| 465 | [CCPA_BACSU](http://us.expasy.org/uniprot/CCPA_BACSU) | Catabolite control protein A (Glucose-resistance amylase regulator) | 51.53 | 2 | 328 1105 |  |  |
|  | [16080026](http://www.ncbi.nlm.nih.gov/entrez/query.fcgi?cmd=Search&db=Protein&term=16080026&doptcmdl=GenPept) | Lacl family transcriptional regulator [Bacillus subtilis subsp. subtilis str. 168] | 25.70 | 1 | 328 |  |  |
| 466 | [16078637](http://www.ncbi.nlm.nih.gov/entrez/query.fcgi?cmd=Search&db=Protein&term=16078637&doptcmdl=GenPept) | RNA-binding Sun protein [Bacillus subtilis subsp. subtilis str. 168] | 51.51 | 1 | 1669 |  |  |
| 467 | [255767243](http://www.ncbi.nlm.nih.gov/entrez/query.fcgi?cmd=Search&db=Protein&term=255767243&doptcmdl=GenPept) | ATP-dependent deoxyribonuclease subunit A [Bacillus subtilis subsp. subtilis str. 168] | 51.42 | 1 | 1937 |  |  |
| 468 | [16077155](http://www.ncbi.nlm.nih.gov/entrez/query.fcgi?cmd=Search&db=Protein&term=16077155&doptcmdl=GenPept) | DNA repair protein RadA [Bacillus subtilis subsp. subtilis str. 168] | 51.10 | 1 | 99 |  |  |
| 469 | [50812263](http://www.ncbi.nlm.nih.gov/entrez/query.fcgi?cmd=Search&db=Protein&term=50812263&doptcmdl=GenPept) | pantothenate kinase [Bacillus subtilis subsp. subtilis str. 168] | 51.10 | 1 | 57 |  |  |
| 470 | [16080093](http://www.ncbi.nlm.nih.gov/entrez/query.fcgi?cmd=Search&db=Protein&term=16080093&doptcmdl=GenPept) | metabolite permease [Bacillus subtilis subsp. subtilis str. 168] | 51.09 | 1 | 69 |  |  |
| 471 | [16078335](http://www.ncbi.nlm.nih.gov/entrez/query.fcgi?cmd=Search&db=Protein&term=16078335&doptcmdl=GenPept) | hypothetical protein BSU12700 [Bacillus subtilis subsp. subtilis str. 168] | 50.73 | 1 | 1980 |  |  |
| 472 | [255767164](http://www.ncbi.nlm.nih.gov/entrez/query.fcgi?cmd=Search&db=Protein&term=255767164&doptcmdl=GenPept) | phosphoribosylformylglycinamidine synthase II [Bacillus subtilis subsp. subtilis str. 168] | 50.73 | 1 | 842 |  |  |
| 473 | [16080733](http://www.ncbi.nlm.nih.gov/entrez/query.fcgi?cmd=Search&db=Protein&term=16080733&doptcmdl=GenPept) | F0F1 ATP synthase subunit epsilon [Bacillus subtilis subsp. subtilis str. 168] | 49.79 | 1 | 1733 |  |  |
| 474 | [16078474](http://www.ncbi.nlm.nih.gov/entrez/query.fcgi?cmd=Search&db=Protein&term=16078474&doptcmdl=GenPept) | hypothetical protein BSU14100 [Bacillus subtilis subsp. subtilis str. 168] | 49.63 | 1 | 1637 |  |  |
| 475 | [16079979](http://www.ncbi.nlm.nih.gov/entrez/query.fcgi?cmd=Search&db=Protein&term=16079979&doptcmdl=GenPept) | hypothetical protein BSU29270 [Bacillus subtilis subsp. subtilis str. 168] | 49.54 | 1 | 184 |  |  |
| 476 | [255767568](http://www.ncbi.nlm.nih.gov/entrez/query.fcgi?cmd=Search&db=Protein&term=255767568&doptcmdl=GenPept) | factor involved in motility [Bacillus subtilis subsp. subtilis str. 168] | 48.83 | 1 | 860 |  |  |
| 477 | [16078646](http://www.ncbi.nlm.nih.gov/entrez/query.fcgi?cmd=Search&db=Protein&term=16078646&doptcmdl=GenPept) | hypothetical protein BSU15830 [Bacillus subtilis subsp. subtilis str. 168] | 48.66 | 1 | 1468 |  |  |
| 478 | [16079869](http://www.ncbi.nlm.nih.gov/entrez/query.fcgi?cmd=Search&db=Protein&term=16079869&doptcmdl=GenPept) | glutamyl-tRNA reductase [Bacillus subtilis subsp. subtilis str. 168] | 48.56 | 2 | 112 2150 |  |  |
| 479 | [16080887](http://www.ncbi.nlm.nih.gov/entrez/query.fcgi?cmd=Search&db=Protein&term=16080887&doptcmdl=GenPept) | AdoMet-dependent methyltransferase [Bacillus subtilis subsp. subtilis str. 168] | 47.56 | 1 | 136 |  |  |
| 480 | [16079311](http://www.ncbi.nlm.nih.gov/entrez/query.fcgi?cmd=Search&db=Protein&term=16079311&doptcmdl=GenPept) | menaquinol:cytochrome c oxidoreductase cytochrome cc subunit [Bacillus subtilis subsp. subtilis str. 168] | 47.49 | 1 | 77 |  |  |
| 481 | [16079332](http://www.ncbi.nlm.nih.gov/entrez/query.fcgi?cmd=Search&db=Protein&term=16079332&doptcmdl=GenPept) | ubiquinone/menaquinone biosynthesis methyltransferase [Bacillus subtilis subsp. subtilis str. 168] | 47.28 | 1 | 1865 |  |  |
| 482 | [16078432](http://www.ncbi.nlm.nih.gov/entrez/query.fcgi?cmd=Search&db=Protein&term=16078432&doptcmdl=GenPept) | flagellar motor protein MotB [Bacillus subtilis subsp. subtilis str. 168] | 46.86 | 2 | 288 1963 |  |  |
| 483 | [16078022](http://www.ncbi.nlm.nih.gov/entrez/query.fcgi?cmd=Search&db=Protein&term=16078022&doptcmdl=GenPept) | aspartate aminotransferase [Bacillus subtilis subsp. subtilis str. 168] | 46.36 | 1 | 1727 |  |  |
| 484 | [7546509](http://www.ncbi.nlm.nih.gov/entrez/query.fcgi?cmd=Search&db=Protein&term=7546509&doptcmdl=GenPept) | Chain A, Crystal Structure Of Bacillus Subtilis Ferrochelatase In Complex With N-Methyl Mesoporphyrin | 46.18 | 1 | 809 |  |  |
|  | [421492](http://www.ncbi.nlm.nih.gov/entrez/query.fcgi?cmd=Search&db=Protein&term=421492&doptcmdl=GenPept) | ferrochelatase (EC 4.99.1.1) hemH [validated] - Bacillus subtilis | 46.18 | 1 | 809 |  |  |
|  | [417116](http://www.ncbi.nlm.nih.gov/entrez/query.fcgi?cmd=Search&db=Protein&term=417116&doptcmdl=GenPept) | Ferrochelatase (Protoheme ferro-lyase) (Heme synthetase) | 46.18 | 1 | 809 |  |  |
|  | [2781160](http://www.ncbi.nlm.nih.gov/entrez/query.fcgi?cmd=Search&db=Protein&term=2781160&doptcmdl=GenPept) | Ferrochelatase From Bacillus Subtilis | 46.18 | 1 | 809 |  |  |
|  | [2633349](http://www.ncbi.nlm.nih.gov/entrez/query.fcgi?cmd=Search&db=Protein&term=2633349&doptcmdl=GenPept) | ferrochelatase [Bacillus subtilis subsp. subtilis str. 168] | 46.18 | 1 | 809 |  |  |
| 485 | [16080155](http://www.ncbi.nlm.nih.gov/entrez/query.fcgi?cmd=Search&db=Protein&term=16080155&doptcmdl=GenPept) | hypothetical protein BSU31030 [Bacillus subtilis subsp. subtilis str. 168] | 46.00 | 1 | 1403 |  |  |
| 486 | [254384649](http://www.ncbi.nlm.nih.gov/entrez/query.fcgi?cmd=Search&db=Protein&term=254384649&doptcmdl=GenPept) | sugar hydrolase [Streptomyces sp. Mg1] | 45.88 | 1 | 1103 |  |  |
|  | [254377488](http://www.ncbi.nlm.nih.gov/entrez/query.fcgi?cmd=Search&db=Protein&term=254377488&doptcmdl=GenPept) | sugar hydrolase [Streptomyces sp. SPB74] | 45.88 | 1 | 1103 |  |  |
|  | [239982214](http://www.ncbi.nlm.nih.gov/entrez/query.fcgi?cmd=Search&db=Protein&term=239982214&doptcmdl=GenPept) | putative sugar hydrolase [Streptomyces albus J1074] | 45.88 | 1 | 1103 |  |  |
|  | [197697291](http://www.ncbi.nlm.nih.gov/entrez/query.fcgi?cmd=Search&db=Protein&term=197697291&doptcmdl=GenPept) | sugar hydrolase [Streptomyces sp. SPB74] | 45.88 | 1 | 1103 |  |  |
|  | [194343533](http://www.ncbi.nlm.nih.gov/entrez/query.fcgi?cmd=Search&db=Protein&term=194343533&doptcmdl=GenPept) | sugar hydrolase [Streptomyces sp. Mg1] | 45.88 | 1 | 1103 |  |  |
| 487 | [255767872](http://www.ncbi.nlm.nih.gov/entrez/query.fcgi?cmd=Search&db=Protein&term=255767872&doptcmdl=GenPept) | replicative DNA helicase [Bacillus subtilis subsp. subtilis str. 168] | 45.61 | 1 | 2060 |  |  |
| 488 | [16080523](http://www.ncbi.nlm.nih.gov/entrez/query.fcgi?cmd=Search&db=Protein&term=16080523&doptcmdl=GenPept) | ABC transporter ATP-binding protein [Bacillus subtilis subsp. subtilis str. 168] | 45.43 | 1 | 1966 |  |  |
| 489 | [16080793](http://www.ncbi.nlm.nih.gov/entrez/query.fcgi?cmd=Search&db=Protein&term=16080793&doptcmdl=GenPept) | hydrolase [Bacillus subtilis subsp. subtilis str. 168] | 45.39 | 1 | 1885 |  |  |
| 490 | [16080766](http://www.ncbi.nlm.nih.gov/entrez/query.fcgi?cmd=Search&db=Protein&term=16080766&doptcmdl=GenPept) | two-component response regulator [Bacillus subtilis subsp. subtilis str. 168] | 44.86 | 1 | 1584 |  |  |
| 491 | [50812242](http://www.ncbi.nlm.nih.gov/entrez/query.fcgi?cmd=Search&db=Protein&term=50812242&doptcmdl=GenPept) | DNA topoisomerase IV subunit B [Bacillus subtilis subsp. subtilis str. 168] | 44.69 | 1 | 1942 |  |  |
|  | [284801671](http://www.ncbi.nlm.nih.gov/entrez/query.fcgi?cmd=Search&db=Protein&term=284801671&doptcmdl=GenPept) | DNA topoisomerase IV subunit B [Listeria monocytogenes 08-5578] | 44.69 | 1 | 1942 |  |  |
| 492 | [16079852](http://www.ncbi.nlm.nih.gov/entrez/query.fcgi?cmd=Search&db=Protein&term=16079852&doptcmdl=GenPept) | septum formation inhibitor [Bacillus subtilis subsp. subtilis str. 168] | 44.62 | 1 | 1445 |  |  |
| 493 | [255767657](http://www.ncbi.nlm.nih.gov/entrez/query.fcgi?cmd=Search&db=Protein&term=255767657&doptcmdl=GenPept) | excinuclease ABC subunit C [Bacillus subtilis subsp. subtilis str. 168] | 44.57 | 1 | 51 |  |  |
| 494 | [16081091](http://www.ncbi.nlm.nih.gov/entrez/query.fcgi?cmd=Search&db=Protein&term=16081091&doptcmdl=GenPept) | regulator of YycFG [Bacillus subtilis subsp. subtilis str. 168] | 43.97 | 1 | 1347 |  |  |
| 495 | [16077166](http://www.ncbi.nlm.nih.gov/entrez/query.fcgi?cmd=Search&db=Protein&term=16077166&doptcmdl=GenPept) | RNA polymerase factor sigma-70 [Bacillus subtilis subsp. subtilis str. 168] | 43.81 | 1 | 1756 |  |  |
| 496 | [16078037](http://www.ncbi.nlm.nih.gov/entrez/query.fcgi?cmd=Search&db=Protein&term=16078037&doptcmdl=GenPept) | ABC transporter ATP-binding protein [Bacillus subtilis subsp. subtilis str. 168] | 43.51 | 1 | 114 |  |  |
| 497 | [255767682](http://www.ncbi.nlm.nih.gov/entrez/query.fcgi?cmd=Search&db=Protein&term=255767682&doptcmdl=GenPept) | hypothetical protein BSU29770 [Bacillus subtilis subsp. subtilis str. 168] | 43.44 | 1 | 463 |  |  |
| 498 | [16078366](http://www.ncbi.nlm.nih.gov/entrez/query.fcgi?cmd=Search&db=Protein&term=16078366&doptcmdl=GenPept) | 6-phosphogluconolactonase [Bacillus subtilis subsp. subtilis str. 168] | 43.41 | 1 | 530 |  |  |
| 499 | [16078030](http://www.ncbi.nlm.nih.gov/entrez/query.fcgi?cmd=Search&db=Protein&term=16078030&doptcmdl=GenPept) | NAD-dependent deacetylase [Bacillus subtilis subsp. subtilis str. 168] | 43.22 | 1 | 820 |  |  |
| 500 | [16080530](http://www.ncbi.nlm.nih.gov/entrez/query.fcgi?cmd=Search&db=Protein&term=16080530&doptcmdl=GenPept) | hypothetical protein BSU34770 [Bacillus subtilis subsp. subtilis str. 168] | 43.17 | 1 | 1679 |  |  |
| 501 | [16077493](http://www.ncbi.nlm.nih.gov/entrez/query.fcgi?cmd=Search&db=Protein&term=16077493&doptcmdl=GenPept) | DNA topoisomerase III [Bacillus subtilis subsp. subtilis str. 168] | 42.78 | 1 | 1913 |  |  |
| 502 | [16079464](http://www.ncbi.nlm.nih.gov/entrez/query.fcgi?cmd=Search&db=Protein&term=16079464&doptcmdl=GenPept) | branched-chain amino acid dehydrogenase [Bacillus subtilis subsp. subtilis str. 168] | 42.06 | 1 | 551 |  |  |
| 503 | [16079331](http://www.ncbi.nlm.nih.gov/entrez/query.fcgi?cmd=Search&db=Protein&term=16079331&doptcmdl=GenPept) | heptaprenyl diphosphate synthase component II [Bacillus subtilis subsp. subtilis str. 168] | 41.55 | 1 | 1831 |  |  |
| 504 | [16079622](http://www.ncbi.nlm.nih.gov/entrez/query.fcgi?cmd=Search&db=Protein&term=16079622&doptcmdl=GenPept) | hydrolase [Bacillus subtilis subsp. subtilis str. 168] | 41.37 | 1 | 72 |  |  |
| 505 | [16077837](http://www.ncbi.nlm.nih.gov/entrez/query.fcgi?cmd=Search&db=Protein&term=16077837&doptcmdl=GenPept) | phosphotransferase system (PTS) N-acetylglucosamine-specific enzyme IICB component [Bacillus subtilis subsp. subtilis str. 168] | 41.35 | 1 | 2034 |  |  |
| 506 | [16077926](http://www.ncbi.nlm.nih.gov/entrez/query.fcgi?cmd=Search&db=Protein&term=16077926&doptcmdl=GenPept) | glycosyl transferase [Bacillus subtilis subsp. subtilis str. 168] | 41.33 | 1 | 2145 |  |  |
| 507 | [16080782](http://www.ncbi.nlm.nih.gov/entrez/query.fcgi?cmd=Search&db=Protein&term=16080782&doptcmdl=GenPept) | transcriptional regulator [Bacillus subtilis subsp. subtilis str. 168] | 41.30 | 1 | 308 |  |  |
| 508 | [255767124](http://www.ncbi.nlm.nih.gov/entrez/query.fcgi?cmd=Search&db=Protein&term=255767124&doptcmdl=GenPept) | mannitol-1-phosphate 5-dehydrogenase [Bacillus subtilis subsp. subtilis str. 168] | 41.30 | 2 | 376 1355 |  |  |
| 509 | [16080767](http://www.ncbi.nlm.nih.gov/entrez/query.fcgi?cmd=Search&db=Protein&term=16080767&doptcmdl=GenPept) | oxidoreductase [Bacillus subtilis subsp. subtilis str. 168] | 41.23 | 1 | 1950 |  |  |
| 510 | [255767654](http://www.ncbi.nlm.nih.gov/entrez/query.fcgi?cmd=Search&db=Protein&term=255767654&doptcmdl=GenPept) | glutamate racemase [Bacillus subtilis subsp. subtilis str. 168] | 41.17 | 1 | 6 |  |  |

| **Protein IDs*, cont.*** | | | | | | | |
| --- | --- | --- | --- | --- | --- | --- | --- |
| *Grp Nr.* | *Accession Number* | *Protein Name* | *Protein Score* | *Unique PSMs* | *PSM Serial Nrs.* | *Other Grp.* | *Score (other)* |
| 511 | [16080017](http://www.ncbi.nlm.nih.gov/entrez/query.fcgi?cmd=Search&db=Protein&term=16080017&doptcmdl=GenPept) | diguanylate cyclase-related enzyme [Bacillus subtilis subsp. subtilis str. 168] | 41.05 | 1 | 797 |  |  |
| 512 | [16080623](http://www.ncbi.nlm.nih.gov/entrez/query.fcgi?cmd=Search&db=Protein&term=16080623&doptcmdl=GenPept) | ATP-binding teichoic acid precursor transporter component [Bacillus subtilis subsp. subtilis str. 168] | 41.01 | 1 | 1982 |  |  |
| 513 | [16079439](http://www.ncbi.nlm.nih.gov/entrez/query.fcgi?cmd=Search&db=Protein&term=16079439&doptcmdl=GenPept) | NADPH dehydrogenase NamA [Bacillus subtilis subsp. subtilis str. 168] | 40.65 | 1 | 1807 |  |  |
| 514 | [16080189](http://www.ncbi.nlm.nih.gov/entrez/query.fcgi?cmd=Search&db=Protein&term=16080189&doptcmdl=GenPept) | NADH-dependent butanol dehydrogenase [Bacillus subtilis subsp. subtilis str. 168] | 40.63 | 1 | 2146 |  |  |
| 515 | [16079950](http://www.ncbi.nlm.nih.gov/entrez/query.fcgi?cmd=Search&db=Protein&term=16079950&doptcmdl=GenPept) | primosomal protein DnaI [Bacillus subtilis subsp. subtilis str. 168] | 40.38 | 1 | 1464 |  |  |
| 516 | [16080240](http://www.ncbi.nlm.nih.gov/entrez/query.fcgi?cmd=Search&db=Protein&term=16080240&doptcmdl=GenPept) | membrane-associated enzyme involved in bacteriocin production [Bacillus subtilis subsp. subtilis str. 168] | 40.22 | 1 | 2077 |  |  |
| 517 | [16077632](http://www.ncbi.nlm.nih.gov/entrez/query.fcgi?cmd=Search&db=Protein&term=16077632&doptcmdl=GenPept) | hypothetical protein BSU05650 [Bacillus subtilis subsp. subtilis str. 168] | 39.90 | 1 | 732 |  |  |
| 518 | [16078639](http://www.ncbi.nlm.nih.gov/entrez/query.fcgi?cmd=Search&db=Protein&term=16078639&doptcmdl=GenPept) | phosphorylated protein phosphatase [Bacillus subtilis subsp. subtilis str. 168] | 39.90 | 1 | 596 |  |  |
| 519 | [16079816](http://www.ncbi.nlm.nih.gov/entrez/query.fcgi?cmd=Search&db=Protein&term=16079816&doptcmdl=GenPept) | single-strand DNA-specific exonuclease [Bacillus subtilis subsp. subtilis str. 168] | 39.89 | 1 | 2107 |  |  |
| 520 | [16080552](http://www.ncbi.nlm.nih.gov/entrez/query.fcgi?cmd=Search&db=Protein&term=16080552&doptcmdl=GenPept) | prolipoprotein diacylglyceryl transferase [Bacillus subtilis subsp. subtilis str. 168] | 39.82 | 1 | 2121 |  |  |
| 521 | [284803003](http://www.ncbi.nlm.nih.gov/entrez/query.fcgi?cmd=Search&db=Protein&term=284803003&doptcmdl=GenPept) | hypothetical protein LM5578_2760 [Listeria monocytogenes 08-5578] | 39.61 | 1 | 1189 |  |  |
|  | [16080812](http://www.ncbi.nlm.nih.gov/entrez/query.fcgi?cmd=Search&db=Protein&term=16080812&doptcmdl=GenPept) | metal-dependent phosphohydrolase [Bacillus subtilis subsp. subtilis str. 168] | 39.61 | 1 | 1189 |  |  |
| 522 | [16078505](http://www.ncbi.nlm.nih.gov/entrez/query.fcgi?cmd=Search&db=Protein&term=16078505&doptcmdl=GenPept) | type I signal peptidase [Bacillus subtilis subsp. subtilis str. 168] | 39.59 | 1 | 1034 |  |  |
| 523 | [16079316](http://www.ncbi.nlm.nih.gov/entrez/query.fcgi?cmd=Search&db=Protein&term=16079316&doptcmdl=GenPept) | hypothetical protein BSU22590 [Bacillus subtilis subsp. subtilis str. 168] | 38.75 | 1 | 488 |  |  |
| 524 | [16078582](http://www.ncbi.nlm.nih.gov/entrez/query.fcgi?cmd=Search&db=Protein&term=16078582&doptcmdl=GenPept) | UDP-N-acetylmuramoylalanyl-D-glutamate--2,6-diaminopimelate ligase [Bacillus subtilis subsp. subtilis str. 168] | 38.44 | 1 | 436 |  |  |
| 525 | [16077664](http://www.ncbi.nlm.nih.gov/entrez/query.fcgi?cmd=Search&db=Protein&term=16077664&doptcmdl=GenPept) | redox-sensing transcriptional repressor Rex [Bacillus subtilis subsp. subtilis str. 168] | 38.32 | 1 | 610 |  |  |
| 526 | [16077816](http://www.ncbi.nlm.nih.gov/entrez/query.fcgi?cmd=Search&db=Protein&term=16077816&doptcmdl=GenPept) | iron-dicitrate ABC transporter ATP-binding protein [Bacillus subtilis subsp. subtilis str. 168] | 38.26 | 1 | 2120 |  |  |
| 527 | [284801854](http://www.ncbi.nlm.nih.gov/entrez/query.fcgi?cmd=Search&db=Protein&term=284801854&doptcmdl=GenPept) | hypothetical protein LM5578_1609 [Listeria monocytogenes 08-5578] | 38.02 | 1 | 1996 |  |  |
|  | [255767581](http://www.ncbi.nlm.nih.gov/entrez/query.fcgi?cmd=Search&db=Protein&term=255767581&doptcmdl=GenPept) | phosphate starvation-induced protein [Bacillus subtilis subsp. subtilis str. 168] | 38.02 | 1 | 1996 |  |  |
| 528 | [255767688](http://www.ncbi.nlm.nih.gov/entrez/query.fcgi?cmd=Search&db=Protein&term=255767688&doptcmdl=GenPept) | NAD(FAD) dehydrogenase [Bacillus subtilis subsp. subtilis str. 168] | 37.06 | 1 | 2042 |  |  |
| 529 | [21398119](http://www.ncbi.nlm.nih.gov/entrez/query.fcgi?cmd=Search&db=Protein&term=21398119&doptcmdl=GenPept) | FGGY_C, FGGY family of carbohydrate kinases, C-terminal domain [Bacillus anthracis A2012] | 36.84 | 1 | 1797 |  |  |
|  | [16081058](http://www.ncbi.nlm.nih.gov/entrez/query.fcgi?cmd=Search&db=Protein&term=16081058&doptcmdl=GenPept) | gluconate kinase [Bacillus subtilis subsp. subtilis str. 168] | 36.84 | 1 | 1797 |  |  |
| 530 | [16077503](http://www.ncbi.nlm.nih.gov/entrez/query.fcgi?cmd=Search&db=Protein&term=16077503&doptcmdl=GenPept) | manganese transport protein MntH [Bacillus subtilis subsp. subtilis str. 168] | 36.63 | 1 | 804 |  |  |
| 531 | [255767704](http://www.ncbi.nlm.nih.gov/entrez/query.fcgi?cmd=Search&db=Protein&term=255767704&doptcmdl=GenPept) | methyl-accepting chemotaxis protein [Bacillus subtilis subsp. subtilis str. 168] | 36.50 | 1 | 1028 | *145* | *61.93* |
|  | [9506813](http://www.ncbi.nlm.nih.gov/entrez/query.fcgi?cmd=Search&db=Protein&term=9506813&doptcmdl=GenPept) | inositol polyphosphate-5-phosphatase D; Inositol polyphosphate-5-phosphatase; Inositol polyphosphate-5-phosphatase, 145 kDa [Rattus norvegicus] | 36.50 | 1 | 1028 |  |  |
|  | [82186403](http://www.ncbi.nlm.nih.gov/entrez/query.fcgi?cmd=Search&db=Protein&term=82186403&doptcmdl=GenPept) | RecName: Full=Phosphatidylinositol-3,4,5-trisphosphate 5-phosphatase 1; AltName: Full=SH2 domain-containing inositol-5'-phosphatase 1; Short=SH2 domain-containing inositol phosphatase 1; Short=SHIP-1 | 36.50 | 1 | 1028 |  |  |
|  | [81861707](http://www.ncbi.nlm.nih.gov/entrez/query.fcgi?cmd=Search&db=Protein&term=81861707&doptcmdl=GenPept) | Phosphatidylinositol-3,4,5-trisphosphate 5-phosphatase 1 (SH2 domain-containing inositol-5'-phosphatase 1) (SH2 domain-containing inositol phosphatase 1) (SHIP-1) | 36.50 | 1 | 1028 |  |  |
|  | [80474664](http://www.ncbi.nlm.nih.gov/entrez/query.fcgi?cmd=Search&db=Protein&term=80474664&doptcmdl=GenPept) | Inositol polyphosphate-5-phosphatase D [Mus musculus] [MASS=133442] | 36.50 | 1 | 1028 |  |  |
| 532 | [16077992](http://www.ncbi.nlm.nih.gov/entrez/query.fcgi?cmd=Search&db=Protein&term=16077992&doptcmdl=GenPept) | glycerol-3-phosphate responding transcription antiterminator [Bacillus subtilis subsp. subtilis str. 168] | 36.01 | 1 | 2043 |  |  |
| 533 | [16078212](http://www.ncbi.nlm.nih.gov/entrez/query.fcgi?cmd=Search&db=Protein&term=16078212&doptcmdl=GenPept) | oligopeptide ABC transporter ATP-binding protein [Bacillus subtilis subsp. subtilis str. 168] | 35.89 | 1 | 2079 |  |  |
| 534 | [16078999](http://www.ncbi.nlm.nih.gov/entrez/query.fcgi?cmd=Search&db=Protein&term=16078999&doptcmdl=GenPept) | nitric-oxide reductase [Bacillus subtilis subsp. subtilis str. 168] | 35.35 | 1 | 2003 |  |  |
| 535 | [16079259](http://www.ncbi.nlm.nih.gov/entrez/query.fcgi?cmd=Search&db=Protein&term=16079259&doptcmdl=GenPept) | 5'3'-exonuclease [Bacillus subtilis subsp. subtilis str. 168] | 35.30 | 1 | 68 |  |  |
| 536 | [16080110](http://www.ncbi.nlm.nih.gov/entrez/query.fcgi?cmd=Search&db=Protein&term=16080110&doptcmdl=GenPept) | hydrolase [Bacillus subtilis subsp. subtilis str. 168] | 35.15 | 1 | 1125 |  |  |
| 537 | [301630541](http://www.ncbi.nlm.nih.gov/entrez/query.fcgi?cmd=Search&db=Protein&term=301630541&doptcmdl=GenPept) | PREDICTED: probable 3-hydroxyacyl-CoA dehydrogenase-like [Xenopus (Silurana) tropicalis] | 35.05 | 1 | 680 | *75* | *53.92* |
|  | [94967395](http://www.ncbi.nlm.nih.gov/entrez/query.fcgi?cmd=Search&db=Protein&term=94967395&doptcmdl=GenPept) | putative nitric-oxide reductase [Acidobacteria bacterium Ellin345] | 35.05 | 1 | 680 |  |  |
|  | [94549445](http://www.ncbi.nlm.nih.gov/entrez/query.fcgi?cmd=Search&db=Protein&term=94549445&doptcmdl=GenPept) | putative nitric-oxide reductase [Acidobacteria bacterium Ellin345] [MASS=84257] | 35.05 | 1 | 680 |  |  |
|  | [90101363](http://www.ncbi.nlm.nih.gov/entrez/query.fcgi?cmd=Search&db=Protein&term=90101363&doptcmdl=GenPept) | Translation initiation factor IF-2 | 35.05 | 1 | 680 |  |  |
|  | [8894780](http://www.ncbi.nlm.nih.gov/entrez/query.fcgi?cmd=Search&db=Protein&term=8894780&doptcmdl=GenPept) | putative ABC transporter ATP-binding subunit [Streptomyces coelicolor A3(2)] [MASS=66959] | 35.05 | 1 | 680 |  |  |
| 538 | [16079911](http://www.ncbi.nlm.nih.gov/entrez/query.fcgi?cmd=Search&db=Protein&term=16079911&doptcmdl=GenPept) | hypothetical protein BSU28590 [Bacillus subtilis subsp. subtilis str. 168] | 34.74 | 1 | 319 |  |  |
| 539 | [16079908](http://www.ncbi.nlm.nih.gov/entrez/query.fcgi?cmd=Search&db=Protein&term=16079908&doptcmdl=GenPept) | long chain acyl-CoA ligase [Bacillus subtilis subsp. subtilis str. 168] | 34.60 | 1 | 5 |  |  |
| 540 | [255767425](http://www.ncbi.nlm.nih.gov/entrez/query.fcgi?cmd=Search&db=Protein&term=255767425&doptcmdl=GenPept) | DNA topoisomerase IV subunit A [Bacillus subtilis subsp. subtilis str. 168] | 34.13 | 1 | 1938 |  |  |
| 541 | [16080415](http://www.ncbi.nlm.nih.gov/entrez/query.fcgi?cmd=Search&db=Protein&term=16080415&doptcmdl=GenPept) | carboxylesterase [Bacillus subtilis subsp. subtilis str. 168] | 33.96 | 1 | 1926 |  |  |
| 542 | [16077369](http://www.ncbi.nlm.nih.gov/entrez/query.fcgi?cmd=Search&db=Protein&term=16077369&doptcmdl=GenPept) | glycine betaine ABC transporter glycine betaine-binding lipoprotein [Bacillus subtilis subsp. subtilis str. 168] | 33.92 | 1 | 641 |  |  |
| 543 | [16078662](http://www.ncbi.nlm.nih.gov/entrez/query.fcgi?cmd=Search&db=Protein&term=16078662&doptcmdl=GenPept) | 30S ribosomal protein S16 [Bacillus subtilis subsp. subtilis str. 168] | 33.75 | 1 | 828 |  |  |
| 544 | [16080169](http://www.ncbi.nlm.nih.gov/entrez/query.fcgi?cmd=Search&db=Protein&term=16080169&doptcmdl=GenPept) | biofilm formation protein [Bacillus subtilis subsp. subtilis str. 168] | 33.73 | 1 | 2054 |  |  |
| 545 | [16078395](http://www.ncbi.nlm.nih.gov/entrez/query.fcgi?cmd=Search&db=Protein&term=16078395&doptcmdl=GenPept) | magnesium transporter [Bacillus subtilis subsp. subtilis str. 168] | 33.67 | 1 | 1232 |  |  |
| 546 | [255767776](http://www.ncbi.nlm.nih.gov/entrez/query.fcgi?cmd=Search&db=Protein&term=255767776&doptcmdl=GenPept) | transcriptional regulator [Bacillus subtilis subsp. subtilis str. 168] | 33.29 | 1 | 229 |  |  |
| 547 | [37805387](http://www.ncbi.nlm.nih.gov/entrez/query.fcgi?cmd=Search&db=Protein&term=37805387&doptcmdl=GenPept) | MGC68448 protein [Xenopus laevis] | 33.16 | 1 | 1046 |  |  |
|  | [284800854](http://www.ncbi.nlm.nih.gov/entrez/query.fcgi?cmd=Search&db=Protein&term=284800854&doptcmdl=GenPept) | hypothetical protein LM5578_0602 [Listeria monocytogenes 08-5578] | 33.16 | 1 | 1046 |  |  |
|  | [27887534](http://www.ncbi.nlm.nih.gov/entrez/query.fcgi?cmd=Search&db=Protein&term=27887534&doptcmdl=GenPept) | Sugar epimerase/dehydratase [Fusobacterium nucleatum subsp. vincentii ATCC 49256] | 33.16 | 1 | 1046 |  |  |
|  | [255767737](http://www.ncbi.nlm.nih.gov/entrez/query.fcgi?cmd=Search&db=Protein&term=255767737&doptcmdl=GenPept) | guanosine 5'-monophosphate oxidoreductase [Bacillus subtilis subsp. subtilis str. 168] | 33.16 | 1 | 1046 |  |  |
|  | [242003824](http://www.ncbi.nlm.nih.gov/entrez/query.fcgi?cmd=Search&db=Protein&term=242003824&doptcmdl=GenPept) | conserved hypothetical protein [Pediculus humanus corporis] | 33.16 | 1 | 1046 |  |  |
| 548 | [16077938](http://www.ncbi.nlm.nih.gov/entrez/query.fcgi?cmd=Search&db=Protein&term=16077938&doptcmdl=GenPept) | Fur family transcriptional regulator [Bacillus subtilis subsp. subtilis str. 168] | 33.03 | 1 | 371 |  |  |
| 549 | [16080225](http://www.ncbi.nlm.nih.gov/entrez/query.fcgi?cmd=Search&db=Protein&term=16080225&doptcmdl=GenPept) | phosphodiesterase [Bacillus subtilis subsp. subtilis str. 168] | 32.96 | 1 | 2130 |  |  |
| 550 | [16077117](http://www.ncbi.nlm.nih.gov/entrez/query.fcgi?cmd=Search&db=Protein&term=16077117&doptcmdl=GenPept) | regulatory protein SpoVG [Bacillus subtilis subsp. subtilis str. 168] | 32.64 | 1 | 661 |  |  |
| 551 | [16080327](http://www.ncbi.nlm.nih.gov/entrez/query.fcgi?cmd=Search&db=Protein&term=16080327&doptcmdl=GenPept) | methionine ABC transporter ATP-binding protein [Bacillus subtilis subsp. subtilis str. 168] | 32.45 | 1 | 342 |  |  |
| 552 | [16080456](http://www.ncbi.nlm.nih.gov/entrez/query.fcgi?cmd=Search&db=Protein&term=16080456&doptcmdl=GenPept) | subunit of an iron-sulfur protein [Bacillus subtilis subsp. subtilis str. 168] | 32.39 | 1 | 2075 |  |  |
| 553 | [16079612](http://www.ncbi.nlm.nih.gov/entrez/query.fcgi?cmd=Search&db=Protein&term=16079612&doptcmdl=GenPept) | hypothetical protein BSU25580 [Bacillus subtilis subsp. subtilis str. 168] | 32.27 | 1 | 812 |  |  |
| 554 | [16080820](http://www.ncbi.nlm.nih.gov/entrez/query.fcgi?cmd=Search&db=Protein&term=16080820&doptcmdl=GenPept) | carrier protein reductase of bacilysin synthesis [Bacillus subtilis subsp. subtilis str. 168] | 32.23 | 1 | 1993 |  |  |
| 555 | [16080880](http://www.ncbi.nlm.nih.gov/entrez/query.fcgi?cmd=Search&db=Protein&term=16080880&doptcmdl=GenPept) | thiamine-phosphate pyrophosphorylase [Bacillus subtilis subsp. subtilis str. 168] | 31.78 | 1 | 414 |  |  |
| 556 | [255767284](http://www.ncbi.nlm.nih.gov/entrez/query.fcgi?cmd=Search&db=Protein&term=255767284&doptcmdl=GenPept) | hypothetical protein BSU12530 [Bacillus subtilis subsp. subtilis str. 168] | 31.47 | 1 | 297 |  |  |
| 557 | [16079897](http://www.ncbi.nlm.nih.gov/entrez/query.fcgi?cmd=Search&db=Protein&term=16079897&doptcmdl=GenPept) | succinate dehydrogenase (cytochrome b558 subunit) [Bacillus subtilis subsp. subtilis str. 168] | 31.31 | 1 | 2050 |  |  |
| 558 | [971343](http://www.ncbi.nlm.nih.gov/entrez/query.fcgi?cmd=Search&db=Protein&term=971343&doptcmdl=GenPept) | nitrate reductase protein J [Bacillus subtilis] | 31.22 | 1 | 1167 |  |  |
|  | [2636263](http://www.ncbi.nlm.nih.gov/entrez/query.fcgi?cmd=Search&db=Protein&term=2636263&doptcmdl=GenPept) | nitrate reductase (protein J) [Bacillus subtilis subsp. subtilis str. 168] | 31.22 | 1 | 1167 |  |  |
|  | [2127191](http://www.ncbi.nlm.nih.gov/entrez/query.fcgi?cmd=Search&db=Protein&term=2127191&doptcmdl=GenPept) | nitrate reductase (EC 1.7.99.4) protein J - Bacillus subtilis | 31.22 | 1 | 1167 |  |  |
|  | [16080779](http://www.ncbi.nlm.nih.gov/entrez/query.fcgi?cmd=Search&db=Protein&term=16080779&doptcmdl=GenPept) | nitrate reductase (protein J) [Bacillus subtilis] | 31.22 | 1 | 1167 |  |  |
|  | [1171654](http://www.ncbi.nlm.nih.gov/entrez/query.fcgi?cmd=Search&db=Protein&term=1171654&doptcmdl=GenPept) | Nitrate reductase delta chain | 31.22 | 1 | 1167 |  |  |
| 559 | [255767339](http://www.ncbi.nlm.nih.gov/entrez/query.fcgi?cmd=Search&db=Protein&term=255767339&doptcmdl=GenPept) | cytochrome caa3 oxidase subunit I [Bacillus subtilis subsp. subtilis str. 168] | 31.04 | 1 | 1737 |  |  |
| 560 | [16078740](http://www.ncbi.nlm.nih.gov/entrez/query.fcgi?cmd=Search&db=Protein&term=16078740&doptcmdl=GenPept) | dihydrodipicolinate synthase [Bacillus subtilis subsp. subtilis str. 168] | 31.00 | 1 | 91 |  |  |
| 561 | [16077724](http://www.ncbi.nlm.nih.gov/entrez/query.fcgi?cmd=Search&db=Protein&term=16077724&doptcmdl=GenPept) | amidohydrolase [Bacillus subtilis subsp. subtilis str. 168] | 30.92 | 1 | 1802 |  |  |
| 562 | [27730023](http://www.ncbi.nlm.nih.gov/entrez/query.fcgi?cmd=Search&db=Protein&term=27730023&doptcmdl=GenPept) | similar to membrane-associated guanylate kinase-interacting protein 1 Maguin-1 [Rattus norvegicus] | 30.91 | 1 | 491 |  |  |
|  | [17536849](http://www.ncbi.nlm.nih.gov/entrez/query.fcgi?cmd=Search&db=Protein&term=17536849&doptcmdl=GenPept) | W10G11.4 [Caenorhabditis elegans] | 30.91 | 1 | 491 |  |  |
|  | [16081027](http://www.ncbi.nlm.nih.gov/entrez/query.fcgi?cmd=Search&db=Protein&term=16081027&doptcmdl=GenPept) | methylmalonate-semialdehyde dehydrogenase [Bacillus subtilis subsp. subtilis str. 168] | 30.91 | 1 | 491 |  |  |
|  | [10864500](http://www.ncbi.nlm.nih.gov/entrez/query.fcgi?cmd=Search&db=Protein&term=10864500&doptcmdl=GenPept) | Hypothetical protein W10G11.4 [Caenorhabditis elegans] [MASS=18355] | 30.91 | 1 | 491 |  |  |
| 563 | [16077800](http://www.ncbi.nlm.nih.gov/entrez/query.fcgi?cmd=Search&db=Protein&term=16077800&doptcmdl=GenPept) | hydrolase [Bacillus subtilis subsp. subtilis str. 168] | 30.89 | 1 | 60 |  |  |
| 564 | [16077158](http://www.ncbi.nlm.nih.gov/entrez/query.fcgi?cmd=Search&db=Protein&term=16077158&doptcmdl=GenPept) | 2-C-methyl-D-erythritol 4-phosphate cytidylyltransferase [Bacillus subtilis subsp. subtilis str. 168] | 30.82 | 1 | 1680 |  |  |
| 565 | [16077202](http://www.ncbi.nlm.nih.gov/entrez/query.fcgi?cmd=Search&db=Protein&term=16077202&doptcmdl=GenPept) | 50S ribosomal protein L30 [Bacillus subtilis subsp. subtilis str. 168] | 30.55 | 1 | 258 |  |  |
| 566 | [50812254](http://www.ncbi.nlm.nih.gov/entrez/query.fcgi?cmd=Search&db=Protein&term=50812254&doptcmdl=GenPept) | SPbeta phage ribonucleoside-diphosphate reductase, alpha subunit [Bacillus subtilis subsp. subtilis str. 168] | 30.51 | 1 | 21 | *16* | *180.91* |
| 567 | [16079306](http://www.ncbi.nlm.nih.gov/entrez/query.fcgi?cmd=Search&db=Protein&term=16079306&doptcmdl=GenPept) | dihydrodipicolinate reductase [Bacillus subtilis subsp. subtilis str. 168] | 30.41 | 1 | 191 |  |  |
| 568 | [16079784](http://www.ncbi.nlm.nih.gov/entrez/query.fcgi?cmd=Search&db=Protein&term=16079784&doptcmdl=GenPept) | hypothetical protein BSU27300 [Bacillus subtilis subsp. subtilis str. 168] | 30.10 | 1 | 547 |  |  |
| 569 | [255767242](http://www.ncbi.nlm.nih.gov/entrez/query.fcgi?cmd=Search&db=Protein&term=255767242&doptcmdl=GenPept) | ATP-dependent deoxyribonuclease subunit B [Bacillus subtilis subsp. subtilis str. 168] | 29.94 | 1 | 1896 |  |  |
| 570 | [65321755](http://www.ncbi.nlm.nih.gov/entrez/query.fcgi?cmd=Search&db=Protein&term=65321755&doptcmdl=GenPept) | COG4472: Uncharacterized protein conserved in bacteria [Bacillus anthracis str. A2012] [MASS=10703] | 29.86 | 1 | 1840 |  |  |
|  | [50812272](http://www.ncbi.nlm.nih.gov/entrez/query.fcgi?cmd=Search&db=Protein&term=50812272&doptcmdl=GenPept) | hypothetical protein BSU27400 [Bacillus subtilis subsp. subtilis str. 168] | 29.86 | 1 | 1840 |  |  |
|  | [229198514](http://www.ncbi.nlm.nih.gov/entrez/query.fcgi?cmd=Search&db=Protein&term=229198514&doptcmdl=GenPept) | hypothetical protein bcere0001_40420 [Bacillus cereus m1293] | 29.86 | 1 | 1840 |  |  |
|  | [229192599](http://www.ncbi.nlm.nih.gov/entrez/query.fcgi?cmd=Search&db=Protein&term=229192599&doptcmdl=GenPept) | hypothetical protein bcere0002_42500 [Bacillus cereus ATCC 10876] | 29.86 | 1 | 1840 |  |  |
|  | [229186627](http://www.ncbi.nlm.nih.gov/entrez/query.fcgi?cmd=Search&db=Protein&term=229186627&doptcmdl=GenPept) | hypothetical protein bcere0004_41700 [Bacillus cereus BGSC 6E1] | 29.86 | 1 | 1840 |  |  |
| 571 | [16080308](http://www.ncbi.nlm.nih.gov/entrez/query.fcgi?cmd=Search&db=Protein&term=16080308&doptcmdl=GenPept) | multiple sugar ABC transporter ATP-binding protein [Bacillus subtilis subsp. subtilis str. 168] | 29.61 | 1 | 1538 |  |  |
| 572 | [16080390](http://www.ncbi.nlm.nih.gov/entrez/query.fcgi?cmd=Search&db=Protein&term=16080390&doptcmdl=GenPept) | molybdate binding regulator [Bacillus subtilis subsp. subtilis str. 168] | 29.57 | 1 | 1890 |  |  |

| **Protein IDs*, cont.*** | | | | | | | |
| --- | --- | --- | --- | --- | --- | --- | --- |
| *Grp Nr.* | *Accession Number* | *Protein Name* | *Protein Score* | *Unique PSMs* | *PSM Serial Nrs.* | *Other Grp.* | *Score (other)* |
| 573 | [16080325](http://www.ncbi.nlm.nih.gov/entrez/query.fcgi?cmd=Search&db=Protein&term=16080325&doptcmdl=GenPept) | methionine ABC transporter, substrate binding lipoprotein [Bacillus subtilis subsp. subtilis str. 168] | 29.44 | 1 | 1606 |  |  |
| 574 | [16078226](http://www.ncbi.nlm.nih.gov/entrez/query.fcgi?cmd=Search&db=Protein&term=16078226&doptcmdl=GenPept) | inorganic polyphosphate/ATP-NAD kinase [Bacillus subtilis subsp. subtilis str. 168] | 29.36 | 1 | 1313 |  |  |
| 575 | [16078046](http://www.ncbi.nlm.nih.gov/entrez/query.fcgi?cmd=Search&db=Protein&term=16078046&doptcmdl=GenPept) | hypothetical protein BSU09810 [Bacillus subtilis subsp. subtilis str. 168] | 29.14 | 1 | 1588 |  |  |
| 576 | [16077810](http://www.ncbi.nlm.nih.gov/entrez/query.fcgi?cmd=Search&db=Protein&term=16077810&doptcmdl=GenPept) | ATP-dependent RNA helicase [Bacillus subtilis subsp. subtilis str. 168] | 28.87 | 1 | 819 |  |  |
| 577 | [16077850](http://www.ncbi.nlm.nih.gov/entrez/query.fcgi?cmd=Search&db=Protein&term=16077850&doptcmdl=GenPept) | NAD(P)H-flavin oxidoreductase [Bacillus subtilis subsp. subtilis str. 168] | 28.84 | 1 | 1692 |  |  |
| 578 | [16077114](http://www.ncbi.nlm.nih.gov/entrez/query.fcgi?cmd=Search&db=Protein&term=16077114&doptcmdl=GenPept) | 4-diphosphocytidyl-2-C-methyl-D-erythritol kinase [Bacillus subtilis subsp. subtilis str. 168] | 28.81 | 1 | 1658 |  |  |
| 579 | [16077145](http://www.ncbi.nlm.nih.gov/entrez/query.fcgi?cmd=Search&db=Protein&term=16077145&doptcmdl=GenPept) | dihydropteroate synthase [Bacillus subtilis subsp. subtilis str. 168] | 28.36 | 1 | 1772 |  |  |
| 580 | [16078346](http://www.ncbi.nlm.nih.gov/entrez/query.fcgi?cmd=Search&db=Protein&term=16078346&doptcmdl=GenPept) | bacteriophage PBSX N-acetylmuramoyl-L-alanine amidase [Bacillus subtilis subsp. subtilis str. 168] | 28.18 | 1 | 1461 |  |  |
| 581 | [255767229](http://www.ncbi.nlm.nih.gov/entrez/query.fcgi?cmd=Search&db=Protein&term=255767229&doptcmdl=GenPept) | Na+-efflux ABC transporter ATP-binding protein [Bacillus subtilis subsp. subtilis str. 168] | 28.11 | 1 | 394 |  |  |
| 582 | [284802667](http://www.ncbi.nlm.nih.gov/entrez/query.fcgi?cmd=Search&db=Protein&term=284802667&doptcmdl=GenPept) | 3'-5' exoribonuclease YhaM [Listeria monocytogenes 08-5578] | 28.09 | 1 | 33 |  |  |
|  | [16078057](http://www.ncbi.nlm.nih.gov/entrez/query.fcgi?cmd=Search&db=Protein&term=16078057&doptcmdl=GenPept) | 3'-5' exoribonuclease YhaM [Bacillus subtilis subsp. subtilis str. 168] | 28.09 | 1 | 33 |  |  |
| 583 | [255767550](http://www.ncbi.nlm.nih.gov/entrez/query.fcgi?cmd=Search&db=Protein&term=255767550&doptcmdl=GenPept) | sulfur transferase [Bacillus subtilis subsp. subtilis str. 168] | 27.91 | 1 | 951 |  |  |
| 584 | [93115453](http://www.ncbi.nlm.nih.gov/entrez/query.fcgi?cmd=Search&db=Protein&term=93115453&doptcmdl=GenPept) | glycosyltransferase family 2 [Escherichia coli] [MASS=36429] | 27.89 | 1 | 665 |  |  |
|  | [7378691](http://www.ncbi.nlm.nih.gov/entrez/query.fcgi?cmd=Search&db=Protein&term=7378691&doptcmdl=GenPept) | mod(mdg4)58.6 [Drosophila melanogaster] | 27.89 | 1 | 665 |  |  |
|  | [39939184](http://www.ncbi.nlm.nih.gov/entrez/query.fcgi?cmd=Search&db=Protein&term=39939184&doptcmdl=GenPept) | bacterial nucleoid DNA-binding protein [Onion yellows phytoplasma OY-M] | 27.89 | 1 | 665 |  |  |
|  | [39939020](http://www.ncbi.nlm.nih.gov/entrez/query.fcgi?cmd=Search&db=Protein&term=39939020&doptcmdl=GenPept) | bacterial nucleoid DNA-binding protein [Onion yellows phytoplasma OY-M] | 27.89 | 1 | 665 |  |  |
|  | [39938906](http://www.ncbi.nlm.nih.gov/entrez/query.fcgi?cmd=Search&db=Protein&term=39938906&doptcmdl=GenPept) | bacterial nucleoid DNA-binding protein [Onion yellows phytoplasma OY-M] [MASS=12526] | 27.89 | 1 | 665 |  |  |
| 585 | [255767837](http://www.ncbi.nlm.nih.gov/entrez/query.fcgi?cmd=Search&db=Protein&term=255767837&doptcmdl=GenPept) | pyrimidine-nucleoside Na+(H+) cotransporter [Bacillus subtilis subsp. subtilis str. 168] | 27.61 | 1 | 933 |  |  |
| 586 | [16078588](http://www.ncbi.nlm.nih.gov/entrez/query.fcgi?cmd=Search&db=Protein&term=16078588&doptcmdl=GenPept) | cell-division initiation protein [Bacillus subtilis subsp. subtilis str. 168] | 27.55 | 1 | 1138 |  |  |
| 587 | [16078632](http://www.ncbi.nlm.nih.gov/entrez/query.fcgi?cmd=Search&db=Protein&term=16078632&doptcmdl=GenPept) | DNA-directed RNA polymerase subunit omega [Bacillus subtilis subsp. subtilis str. 168] | 27.52 | 1 | 1889 |  |  |
| 588 | [16078178](http://www.ncbi.nlm.nih.gov/entrez/query.fcgi?cmd=Search&db=Protein&term=16078178&doptcmdl=GenPept) | phosphatase [Bacillus subtilis subsp. subtilis str. 168] | 27.41 | 1 | 1962 |  |  |
| 589 | [16079621](http://www.ncbi.nlm.nih.gov/entrez/query.fcgi?cmd=Search&db=Protein&term=16079621&doptcmdl=GenPept) | GTP-binding protein YqeH [Bacillus subtilis subsp. subtilis str. 168] | 27.36 | 1 | 1979 |  |  |
| 590 | [927531](http://www.ncbi.nlm.nih.gov/entrez/query.fcgi?cmd=Search&db=Protein&term=927531&doptcmdl=GenPept) | unknown [Saccharomyces cerevisiae] | 26.88 | 1 | 726 |  |  |
|  | [9055356](http://www.ncbi.nlm.nih.gov/entrez/query.fcgi?cmd=Search&db=Protein&term=9055356&doptcmdl=GenPept) | syntaxin 8; syntaxin-like protein 3I35 [Mus musculus] | 26.88 | 1 | 726 |  |  |
|  | [89272091](http://www.ncbi.nlm.nih.gov/entrez/query.fcgi?cmd=Search&db=Protein&term=89272091&doptcmdl=GenPept) | CDC7 cell division cycle 7 [Xenopus (Silurana) tropicalis] | 26.88 | 1 | 726 |  |  |
|  | [81674104](http://www.ncbi.nlm.nih.gov/entrez/query.fcgi?cmd=Search&db=Protein&term=81674104&doptcmdl=GenPept) | EF-hand calcium binding domain 6 [Bos taurus] [MASS=41754] | 26.88 | 1 | 726 |  |  |
|  | [74582329](http://www.ncbi.nlm.nih.gov/entrez/query.fcgi?cmd=Search&db=Protein&term=74582329&doptcmdl=GenPept) | Protein TAPT1 homolog | 26.88 | 1 | 726 |  |  |
| 591 | [255767245](http://www.ncbi.nlm.nih.gov/entrez/query.fcgi?cmd=Search&db=Protein&term=255767245&doptcmdl=GenPept) | DNA ATP-dependent repair enzyme [Bacillus subtilis subsp. subtilis str. 168] | 26.83 | 1 | 1668 |  |  |
| 592 | [255767671](http://www.ncbi.nlm.nih.gov/entrez/query.fcgi?cmd=Search&db=Protein&term=255767671&doptcmdl=GenPept) | formamidopyrimidine-DNA glycosylase [Bacillus subtilis subsp. subtilis str. 168] | 26.62 | 1 | 347 |  |  |
| 593 | [16078553](http://www.ncbi.nlm.nih.gov/entrez/query.fcgi?cmd=Search&db=Protein&term=16078553&doptcmdl=GenPept) | cytochrome caa3 oxidase subunit II [Bacillus subtilis subsp. subtilis str. 168] | 26.61 | 1 | 1792 |  |  |
| 594 | [16080686](http://www.ncbi.nlm.nih.gov/entrez/query.fcgi?cmd=Search&db=Protein&term=16080686&doptcmdl=GenPept) | hypothetical protein BSU36330 [Bacillus subtilis subsp. subtilis str. 168] | 26.24 | 1 | 300 |  |  |
| 595 | [16078141](http://www.ncbi.nlm.nih.gov/entrez/query.fcgi?cmd=Search&db=Protein&term=16078141&doptcmdl=GenPept) | cell wall-associated protease [Bacillus subtilis subsp. subtilis str. 168] | 25.56 | 1 | 1323 |  |  |
| 596 | [16078294](http://www.ncbi.nlm.nih.gov/entrez/query.fcgi?cmd=Search&db=Protein&term=16078294&doptcmdl=GenPept) | NADH dehydrogenase [Bacillus subtilis subsp. subtilis str. 168] | 25.35 | 1 | 1975 |  |  |
| 597 | [16078488](http://www.ncbi.nlm.nih.gov/entrez/query.fcgi?cmd=Search&db=Protein&term=16078488&doptcmdl=GenPept) | repressor of comK [Bacillus subtilis subsp. subtilis str. 168] | 25.31 | 1 | 2067 |  |  |
| 598 | [16077889](http://www.ncbi.nlm.nih.gov/entrez/query.fcgi?cmd=Search&db=Protein&term=16077889&doptcmdl=GenPept) | ABC transporter ATP-binding protein [Bacillus subtilis subsp. subtilis str. 168] | 25.20 | 1 | 92 |  |  |
| 599 | [16080219](http://www.ncbi.nlm.nih.gov/entrez/query.fcgi?cmd=Search&db=Protein&term=16080219&doptcmdl=GenPept) | two-component response regulator [Bacillus subtilis subsp. subtilis str. 168] | 25.18 | 1 | 1663 |  |  |
| 600 | [RS9_OCEIH](http://us.expasy.org/uniprot/RS9_OCEIH) | 30S ribosomal protein S9 | 25.08 | 1 | 381 |  |  |
|  | [RS9_LISMO](http://us.expasy.org/uniprot/RS9_LISMO) | 30S ribosomal protein S9 | 25.08 | 1 | 381 |  |  |
|  | [RS9_LISMF](http://us.expasy.org/uniprot/RS9_LISMF) | 30S ribosomal protein S9 | 25.08 | 1 | 381 |  |  |
|  | [RS9_LISIN](http://us.expasy.org/uniprot/RS9_LISIN) | 30S ribosomal protein S9 | 25.08 | 1 | 381 |  |  |
|  | [RS9_BACSU](http://us.expasy.org/uniprot/RS9_BACSU) | 30S ribosomal protein S9 (BS10) | 25.08 | 1 | 381 |  |  |
| 601 | [16078275](http://www.ncbi.nlm.nih.gov/entrez/query.fcgi?cmd=Search&db=Protein&term=16078275&doptcmdl=GenPept) | secreted deoxyriboendonuclease [Bacillus subtilis subsp. subtilis str. 168] | 24.69 | 1 | 107 |  |  |
| 602 | [16078638](http://www.ncbi.nlm.nih.gov/entrez/query.fcgi?cmd=Search&db=Protein&term=16078638&doptcmdl=GenPept) | ribosomal RNA large subunit methyltransferase N [Bacillus subtilis subsp. subtilis str. 168] | 24.65 | 1 | 857 |  |  |
| 603 | [16080131](http://www.ncbi.nlm.nih.gov/entrez/query.fcgi?cmd=Search&db=Protein&term=16080131&doptcmdl=GenPept) | O-succinylbenzoic acid--CoA ligase [Bacillus subtilis subsp. subtilis str. 168] | 24.58 | 1 | 26 |  |  |
| 604 | [16080168](http://www.ncbi.nlm.nih.gov/entrez/query.fcgi?cmd=Search&db=Protein&term=16080168&doptcmdl=GenPept) | integral inner membrane protein [Bacillus subtilis subsp. subtilis str. 168] | 24.50 | 1 | 2108 |  |  |
| 605 | [2634664](http://www.ncbi.nlm.nih.gov/entrez/query.fcgi?cmd=Search&db=Protein&term=2634664&doptcmdl=GenPept) | ypjH [Bacillus subtilis subsp. subtilis str. 168] | 24.35 | 1 | 1859 |  |  |
|  | [221323682](http://www.ncbi.nlm.nih.gov/entrez/query.fcgi?cmd=Search&db=Protein&term=221323682&doptcmdl=GenPept) | hypothetical protein BsubsS_12322 [Bacillus subtilis subsp. subtilis str. SMY] | 24.35 | 1 | 1859 |  |  |
|  | [221319406](http://www.ncbi.nlm.nih.gov/entrez/query.fcgi?cmd=Search&db=Protein&term=221319406&doptcmdl=GenPept) | hypothetical protein BsubsJ_12193 [Bacillus subtilis subsp. subtilis str. JH642] | 24.35 | 1 | 1859 |  |  |
|  | [221314484](http://www.ncbi.nlm.nih.gov/entrez/query.fcgi?cmd=Search&db=Protein&term=221314484&doptcmdl=GenPept) | hypothetical protein BsubsN3_12267 [Bacillus subtilis subsp. subtilis str. NCIB 3610] | 24.35 | 1 | 1859 |  |  |
|  | [221310162](http://www.ncbi.nlm.nih.gov/entrez/query.fcgi?cmd=Search&db=Protein&term=221310162&doptcmdl=GenPept) | hypothetical protein Bsubs1_12346 [Bacillus subtilis subsp. subtilis str. 168] | 24.35 | 1 | 1859 |  |  |
| 606 | [16077609](http://www.ncbi.nlm.nih.gov/entrez/query.fcgi?cmd=Search&db=Protein&term=16077609&doptcmdl=GenPept) | two-component response regulator YdfH [Bacillus subtilis subsp. subtilis str. 168] | 23.84 | 1 | 1276 |  |  |
| 607 | [255767683](http://www.ncbi.nlm.nih.gov/entrez/query.fcgi?cmd=Search&db=Protein&term=255767683&doptcmdl=GenPept) | hypothetical protein BSU29780 [Bacillus subtilis subsp. subtilis str. 168] | 23.44 | 1 | 969 |  |  |
| 608 | [16078619](http://www.ncbi.nlm.nih.gov/entrez/query.fcgi?cmd=Search&db=Protein&term=16078619&doptcmdl=GenPept) | orotidine 5'-phosphate decarboxylase [Bacillus subtilis subsp. subtilis str. 168] | 23.36 | 1 | 1952 |  |  |
| 609 | [16078911](http://www.ncbi.nlm.nih.gov/entrez/query.fcgi?cmd=Search&db=Protein&term=16078911&doptcmdl=GenPept) | 3-ketoacyl-(acyl-carrier-protein) reductase [Bacillus subtilis subsp. subtilis str. 168] | 23.29 | 1 | 1284 |  |  |
| 610 | [255767198](http://www.ncbi.nlm.nih.gov/entrez/query.fcgi?cmd=Search&db=Protein&term=255767198&doptcmdl=GenPept) | sporulation-control gene [Bacillus subtilis subsp. subtilis str. 168] | 23.25 | 1 | 326 |  |  |
| 611 | [16080926](http://www.ncbi.nlm.nih.gov/entrez/query.fcgi?cmd=Search&db=Protein&term=16080926&doptcmdl=GenPept) | cytochrome bd ubiquinol oxidase subunit II [Bacillus subtilis subsp. subtilis str. 168] | 22.94 | 1 | 565 |  |  |
| 612 | [16079775](http://www.ncbi.nlm.nih.gov/entrez/query.fcgi?cmd=Search&db=Protein&term=16079775&doptcmdl=GenPept) | hypothetical protein BSU27210 [Bacillus subtilis subsp. subtilis str. 168] | 22.88 | 1 | 938 |  |  |
| 613 | [255767213](http://www.ncbi.nlm.nih.gov/entrez/query.fcgi?cmd=Search&db=Protein&term=255767213&doptcmdl=GenPept) | RNA pseudouridine synthase [Bacillus subtilis subsp. subtilis str. 168] | 22.74 | 1 | 1121 |  |  |
| 614 | [255767820](http://www.ncbi.nlm.nih.gov/entrez/query.fcgi?cmd=Search&db=Protein&term=255767820&doptcmdl=GenPept) | methyltetrahydrofolate methyltransferase [Bacillus subtilis subsp. subtilis str. 168] | 22.71 | 1 | 650 |  |  |
| 615 | [16079330](http://www.ncbi.nlm.nih.gov/entrez/query.fcgi?cmd=Search&db=Protein&term=16079330&doptcmdl=GenPept) | nucleoside diphosphate kinase [Bacillus subtilis subsp. subtilis str. 168] | 22.69 | 1 | 590 |  |  |
| 616 | [16077208](http://www.ncbi.nlm.nih.gov/entrez/query.fcgi?cmd=Search&db=Protein&term=16077208&doptcmdl=GenPept) | 50S ribosomal protein L36 [Bacillus subtilis subsp. subtilis str. 168] | 22.37 | 1 | 249 |  |  |
| 617 | [16080771](http://www.ncbi.nlm.nih.gov/entrez/query.fcgi?cmd=Search&db=Protein&term=16080771&doptcmdl=GenPept) | iron-sulphur-binding reductase [Bacillus subtilis subsp. subtilis str. 168] | 22.20 | 1 | 826 |  |  |
| 618 | [16079412](http://www.ncbi.nlm.nih.gov/entrez/query.fcgi?cmd=Search&db=Protein&term=16079412&doptcmdl=GenPept) | NAD-dependent malic enzyme [Bacillus subtilis subsp. subtilis str. 168] | 21.98 | 1 | 763 |  |  |
| 619 | [16080451](http://www.ncbi.nlm.nih.gov/entrez/query.fcgi?cmd=Search&db=Protein&term=16080451&doptcmdl=GenPept) | alkanal monooxygenase [Bacillus subtilis subsp. subtilis str. 168] | 21.96 | 1 | 340 |  |  |
| 620 | [255767078](http://www.ncbi.nlm.nih.gov/entrez/query.fcgi?cmd=Search&db=Protein&term=255767078&doptcmdl=GenPept) | glycine betaine ABC transporter ATP-binding protein [Bacillus subtilis subsp. subtilis str. 168] | 21.91 | 1 | 893 |  |  |
| 621 | [255767736](http://www.ncbi.nlm.nih.gov/entrez/query.fcgi?cmd=Search&db=Protein&term=255767736&doptcmdl=GenPept) | ferredoxin-NADP+ reductase [Bacillus subtilis subsp. subtilis str. 168] | 21.72 | 1 | 1805 |  |  |
| 622 | [16077620](http://www.ncbi.nlm.nih.gov/entrez/query.fcgi?cmd=Search&db=Protein&term=16077620&doptcmdl=GenPept) | hypothetical protein BSU05530 [Bacillus subtilis subsp. subtilis str. 168] | 21.71 | 1 | 2095 |  |  |
| 623 | [85986601](http://www.ncbi.nlm.nih.gov/entrez/query.fcgi?cmd=Search&db=Protein&term=85986601&doptcmdl=GenPept) | relaxin/insulin-like family peptide receptor 1 [Homo sapiens] | 21.25 | 1 | 853 |  |  |
|  | [85567577](http://www.ncbi.nlm.nih.gov/entrez/query.fcgi?cmd=Search&db=Protein&term=85567577&doptcmdl=GenPept) | Leucine-rich repeat-containing G protein-coupled receptor 7 [Homo sapiens] | 21.25 | 1 | 853 |  |  |
|  | [82583720](http://www.ncbi.nlm.nih.gov/entrez/query.fcgi?cmd=Search&db=Protein&term=82583720&doptcmdl=GenPept) | Muscle M-line assembly protein unc-89 (Uncoordinated protein 89) | 21.25 | 1 | 853 |  |  |
|  | [81864127](http://www.ncbi.nlm.nih.gov/entrez/query.fcgi?cmd=Search&db=Protein&term=81864127&doptcmdl=GenPept) | Relaxin receptor 1 (Relaxin family peptide receptor 1) (Leucine-rich repeat-containing G-protein coupled receptor 7) | 21.25 | 1 | 853 |  |  |
|  | [81864126](http://www.ncbi.nlm.nih.gov/entrez/query.fcgi?cmd=Search&db=Protein&term=81864126&doptcmdl=GenPept) | Relaxin receptor 1 (Relaxin family peptide receptor 1) (Leucine-rich repeat-containing G-protein coupled receptor 7) | 21.25 | 1 | 853 |  |  |
| 624 | [16080901](http://www.ncbi.nlm.nih.gov/entrez/query.fcgi?cmd=Search&db=Protein&term=16080901&doptcmdl=GenPept) | D-alanine--poly(phosphoribitol) ligase subunit 1 [Bacillus subtilis subsp. subtilis str. 168] | 20.91 | 1 | 1209 |  |  |
| 625 | [16078684](http://www.ncbi.nlm.nih.gov/entrez/query.fcgi?cmd=Search&db=Protein&term=16078684&doptcmdl=GenPept) | flagellar MS-ring protein [Bacillus subtilis subsp. subtilis str. 168] | 20.64 | 1 | 1381 |  |  |
| 626 | [7451678](http://www.ncbi.nlm.nih.gov/entrez/query.fcgi?cmd=Search&db=Protein&term=7451678&doptcmdl=GenPept) | phosphoglycerate mutase (glycolysis) homolog yhfR - Bacillus subtilis | 20.42 | 1 | 701 |  |  |
|  | [2633370](http://www.ncbi.nlm.nih.gov/entrez/query.fcgi?cmd=Search&db=Protein&term=2633370&doptcmdl=GenPept) | yhfR [Bacillus subtilis subsp. subtilis str. 168] | 20.42 | 1 | 701 |  |  |
|  | [2226254](http://www.ncbi.nlm.nih.gov/entrez/query.fcgi?cmd=Search&db=Protein&term=2226254&doptcmdl=GenPept) | hypothetical protein [Bacillus subtilis] | 20.42 | 1 | 701 |  |  |
|  | [16078098](http://www.ncbi.nlm.nih.gov/entrez/query.fcgi?cmd=Search&db=Protein&term=16078098&doptcmdl=GenPept) | similar to phosphoglycerate mutase (glycolysis) [Bacillus subtilis] | 20.42 | 1 | 701 |  |  |
| 627 | [16080079](http://www.ncbi.nlm.nih.gov/entrez/query.fcgi?cmd=Search&db=Protein&term=16080079&doptcmdl=GenPept) | multiple sugar-binding lipoprotein [Bacillus subtilis subsp. subtilis str. 168] | 20.37 | 1 | 2069 |  |  |
| 628 | [16079829](http://www.ncbi.nlm.nih.gov/entrez/query.fcgi?cmd=Search&db=Protein&term=16079829&doptcmdl=GenPept) | oxidoreductase [Bacillus subtilis subsp. subtilis str. 168] | 20.35 | 1 | 1494 |  |  |
| 629 | [255767437](http://www.ncbi.nlm.nih.gov/entrez/query.fcgi?cmd=Search&db=Protein&term=255767437&doptcmdl=GenPept) | molybdopterin cofactor oxido-reductase [Bacillus subtilis subsp. subtilis str. 168] | 20.25 | 1 | 159 |  |  |

**Note: Number of proteins per group ≤ 5.**

*Group Nr.* By the rule of parsimony, we have gathered all proteins sharing the same identified peptides into a single group.

*Accession Nr.* The accession number from the searched database (DB).

*Protein Name* The protein name in the DB.

*Protein Score* Sum of the contributing peptide scores.

NOTE: This score can only be used to support the identification of **one** protein per group.

*Unique PSMs.* The number of unique peptide-spectrum matches that contribute to the protein assignment.

*PSM Serial Nrs.* Sequential number of assigned peptide (in the following table.)

*Other Grp.* If the protein is assigned to another group, that (those) group number(s).

*Score (Other)* Protein score (as above) for this protein in other group(s).

| **Assigned Peptides** | | | | | | | | | | | |
| --- | --- | --- | --- | --- | --- | --- | --- | --- | --- | --- | --- |
| *#* | *Sequence* | *PTM Site* | *Nr. Scans* | *Logistic Score* | *MOWSE Score* | *Delta Cn* | *Isolated Mass* | *Delta Mass* | *Charge State* | *Matched Ions* | *Scan Nr.* |
| 1 | K.DLGALTVGVVTRPFTFEGR.K |  | 1 | 0.6635 | 36.490 | 0.978 | 679.04 | 0 | 3 | 11/36 | 10002 |
| 2 | K.LGQIDPEQDVPYVLETIK.L |  | 1 | 0.4156 | 21.690 | 0.000 | 686.36 | 1 | 3 | 20/34 | 10004 |
| 3 | R.AVLELAGVADILSK.S |  | 3 | 0.4049 | 95.370 | 0.000 | 699.91 | 1 | 2 | 18/26 | 10006 |
| 4 | K.VDNLIIGGGLAYTFVK.A |  | 1 | 0.3677 | 43.420 | 0.000 | 560.65 | 0 | 3 | 16/30 | 10009 |
| 5 | K.LTFHDILTDALK.L |  | 1 | 0.3394 | 34.600 | 0.000 | 462.93 | 1 | 3 | 8/22 | 10018 |
| 6 | K.VENLACPLLVPFVESGK.F |  | 2 | 0.4097 | 41.170 | 0.000 | 936.50 | 1 | 2 | 22/32 | 10020 |
| 7 | K.FAALNSAVWSGGSFIYVPK.G |  | 2 | 0.6608 | 89.740 | 0.000 | 1007.53 | 0 | 2 | 28/36 | 10021 |
| 8 | R.LGAYAVELLLEGK.G |  | 2 | 0.3072 | 86.210 | 0.000 | 688.40 | 0 | 2 | 10/24 | 10024 |
| 9 | R.LAELLDIPCITTITK.L |  | 1 | 0.2266 | 75.960 | 0.000 | 850.98 | 1 | 2 | 8/28 | 10027 |
| 10 | K.WTYWQPLITPALK.K |  | 1 | 0.8590 | 26.980 | 0.909 | 539.63 | 0 | 3 | 6/24 | 10029 |
| 11 | R.TVIDEIGDPLVHLIR.N |  | 2 | 0.4556 | 57.740 | 0.000 | 845.48 | 1 | 2 | 11/28 | 10032 |
| 12 | K.EQLEDVLDTLTDR.E |  | 1 | 0.5941 | 49.130 | 0.000 | 773.88 | 1 | 2 | 10/24 | 10035 |
| 13 | R.ESVENGVACLTSLDTAEAILR.V |  | 1 | 0.1753 | 51.400 | 0.000 | 750.04 | 1 | 3 | 14/40 | 10037 |
| 14 | R.FIVGPCAVESYEQVAEVAAAAK.K |  | 2 | 0.1319 | 68.900 | 0.000 | 1155.08 | 1 | 2 | 12/42 | 10039 |
| 15 | K.VANVPIVPEVDPPEELFNVDPK.K |  | 1 | 0.1892 | 22.280 | 0.000 | 1209.13 | 1 | 2 | 18/42 | 10051 |
| 16 | K.TVPTAFEFTDIAGIVK.G |  | 1 | 0.2382 | 85.660 | 0.000 | 854.96 | 0 | 2 | 13/30 | 10069 |
| 17 | R.TQIFELEPLTPELIK.Q |  | 2 | 0.9273 | 52.050 | 0.846 | 886.00 | 0 | 2 | 11/28 | 10070 |
| 18 | K.QLSVGPLLAEAIIR.V |  | 2 | 0.2421 | 59.060 | 0.000 | 740.45 | 0 | 2 | 9/26 | 10074 |
| 19 | K.VSVIGAGFTGATTAFLIAQK.E |  | 1 | 0.1190 | 87.410 | 0.000 | 976.55 | 0 | 2 | 14/38 | 10083 |
| 20 | K.FKDVAGADEEKQELVEVVEFLK.D |  | 1 | 0.4990 | 29.830 | 0.937 | 631.33 | 1 | 4 | 8/42 | 10088 |
| 21 | K.EAVHSYFVDYINQNTVFFHDLK.E |  | 1 | 0.5853 | 30.510 | 0.000 | 672.33 | 1 | 4 | 17/42 | 10090 |
| 22 | K.TILDSLEIQDPDLAEEIK.K |  | 1 | 0.6454 | 62.670 | 0.000 | 1021.53 | 0 | 2 | 22/34 | 10093 |
| 23 | K.NVADAEDELHTFDALLVR.S |  | 1 | 0.3614 | 90.340 | 0.000 | 1014.51 | 0 | 2 | 14/34 | 10096 |
| 24 | R.EGLTVLEYFISTHGAR.K |  | 2 | 0.2901 | 68.650 | 0.000 | 896.96 | 1 | 2 | 9/30 | 10103 |
| 25 | K.TNEEVLEYAGAYLQYYR.E |  | 2 | 0.3664 | 89.710 | 0.000 | 1041.49 | 0 | 2 | 10/32 | 10114 |
| 26 | R.CILLGGGPAPLPLLEECR.E |  | 1 | 0.9679 | 24.580 | 0.987 | 655.68 | 0 | 3 | 27/34 | 10119 |
| 27 | R.NDSIILPCDVTNDAEIETCFASIK.E |  | 1 | 0.2128 | 28.180 | 0.000 | 909.09 | 0 | 3 | 13/46 | 10129 |
| 28 | K.ELGDLAQSSLDLALGR.G |  | 1 | 0.2184 | 59.900 | 0.000 | 829.44 | 1 | 2 | 11/30 | 10130 |
| 29 | R.DFVSEDWEWIGK.D |  | 1 | 0.8689 | 60.200 | 0.000 | 755.85 | 0 | 2 | 17/22 | 10132 |
| 30 | K.IVPISEIPSDLEAIDIGTK.T |  | 3 | 0.1795 | 58.290 | 0.000 | 1005.55 | 0 | 2 | 13/36 | 10133 |
| 31 | R.AEASNETIGLFGLLR.M |  | 1 | 0.8599 | 86.790 | 0.995 | 795.93 | 1 | 2 | 10/28 | 10142 |
| 32 | R.QTDSVALYEVGSVFLTK.E |  | 2 | 0.2356 | 84.830 | 0.000 | 928.99 | 0 | 2 | 13/32 | 10146 |
| 33 | R.DLLYAGVILHDLGK.V |  | 1 | 0.8487 | 28.090 | 0.890 | 509.62 | 0 | 3 | 9/26 | 10162 |
| 34 | K.VVVFDFGSLDTLDK.L |  | 1 | 0.4066 | 67.910 | 0.000 | 777.91 | 0 | 2 | 12/26 | 10172 |
| 35 | K.EDTVAGNYFYFFR.A |  | 1 | 0.4909 | 59.050 | 0.000 | 814.87 | 0 | 2 | 8/24 | 10176 |
| 36 | R.ALTLGGIPVVSHLGLTPQSVGVLGGYK.V |  | 1 | 0.0776 | 44.050 | 0.000 | 878.51 | 0 | 3 | 20/52 | 10185 |
| 37 | K.ITQVLDNIISNALK.Y |  | 1 | 0.9485 | 62.880 | 0.875 | 771.45 | 0 | 2 | 18/26 | 10186 |
| 38 | K.NLGAPYELLLQIK.K |  | 2 | 0.8388 | 49.110 | 0.886 | 736.43 | 0 | 2 | 8/24 | 10189 |
| 39 | K.QLDAFIELLK.E |  | 1 | 0.3811 | 36.710 | 0.000 | 595.35 | 0 | 2 | 7/18 | 10193 |
| 40 | K.EVWQYEFFGDLR.T |  | 1 | 0.7308 | 71.870 | 0.000 | 794.87 | 0 | 2 | 9/22 | 10194 |
| 41 | R.LSFGVQTFEDDLLEK.I |  | 1 | 0.3192 | 70.360 | 0.000 | 870.94 | 1 | 2 | 10/28 | 10196 |
| 42 | R.VLEVLDLVQLK.H |  | 2 | 0.4998 | 85.750 | 0.000 | 634.89 | 0 | 2 | 9/20 | 10200 |
| 43 | K.FYTEEGNYDIVGNNTPVFFIR.D |  | 1 | 0.5140 | 39.390 | 0.000 | 832.40 | 0 | 3 | 17/40 | 10202 |
| 44 | R.INYDLANDLGNLLNR.T |  | 1 | 0.9585 | 128.450 | 0.987 | 859.45 | 1 | 2 | 12/28 | 10203 |
| 45 | K.VASLLVDLLKE.- |  | 1 | 0.3363 | 58.820 | 0.000 | 600.37 | 0 | 2 | 11/20 | 10212 |
| 46 | K.VGILDLLK.A |  | 1 | 0.8807 | 34.310 | 0.759 | 435.79 | 0 | 2 | 10/14 | 10214 |
| 47 | R.SNADNFTIEEIAEGVEFAK.K |  | 1 | 0.2061 | 111.130 | 0.000 | 1042.49 | 0 | 2 | 12/36 | 10222 |
| 48 | K.LGDVNVYTISADLPFAQAR.W |  | 2 | 0.2274 | 48.200 | 0.000 | 1025.53 | 1 | 2 | 12/36 | 10226 |
| 49 | R.VINGIADQTNLLALNAAIEAAR.A |  | 1 | 0.2690 | 61.930 | 0.000 | 751.08 | 0 | 3 | 19/42 | 10232 |
| 50 | K.TNQEFFDILNQEWK.Q |  | 2 | 0.9773 | 72.380 | 0.988 | 906.43 | 1 | 2 | 9/26 | 10239 |
| 51 | R.ENLPLPDLIIIDGGK.G |  | 1 | 0.1291 | 44.570 | 0.000 | 803.96 | 0 | 2 | 10/28 | 10240 |
| 52 | K.IIGNEFIYVFDDEADKLK.G |  | 1 | 0.2459 | 29.490 | 0.000 | 710.37 | 1 | 3 | 15/34 | 10257 |
| 53 | K.ELFGFPLIAR.A |  | 1 | 0.9045 | 32.840 | 0.949 | 581.84 | 1 | 2 | 7/18 | 10258 |
| 54 | K.GSLLWLLDETK.T |  | 1 | 0.4764 | 61.370 | 0.000 | 637.85 | 1 | 2 | 15/20 | 10262 |
| 55 | K.ELADVVLVDIPQLENPTK.G |  | 1 | 0.0961 | 52.500 | 0.000 | 997.05 | 1 | 2 | 7/34 | 10280 |
| 56 | K.TFADLNHIVNLSLQQTFTR.S |  | 1 | 0.6449 | 52.310 | 0.000 | 740.06 | 0 | 3 | 16/36 | 10300 |
| 57 | K.ALLEFLNDLK.S |  | 1 | 0.4034 | 51.100 | 0.000 | 588.34 | 0 | 2 | 7/18 | 10321 |
| 58 | R.FIAEIPDDLLENLNEK.K |  | 2 | 0.6767 | 75.650 | 0.000 | 936.98 | 1 | 2 | 18/30 | 10324 |
| 59 | K.DGHDIPFETFLGFK.G |  | 1 | 0.3698 | 39.420 | 0.000 | 541.60 | 0 | 3 | 8/26 | 10326 |
| 60 | R.DSGLFPFFK.D |  | 1 | 0.7887 | 30.890 | 0.635 | 529.27 | 0 | 2 | 9/16 | 10330 |
| 61 | K.NVGVPYIVVFLNK.C |  | 6 | 0.4622 | 59.270 | 0.000 | 731.43 | 1 | 2 | 16/24 | 10333 |
| 62 | R.IASAGQAGAAFVSFPQDVVNEVTNTK.N |  | 2 | 0.1664 | 66.720 | 0.000 | 874.45 | 1 | 3 | 18/50 | 10334 |
| 63 | R.INPENLIILVDHLK.C |  | 1 | 0.9293 | 37.770 | 0.832 | 544.32 | 0 | 3 | 15/26 | 10336 |
| 64 | R.TSPYDFQGLGVEGLQILK.R |  | 2 | 0.9487 | 90.440 | 0.965 | 655.68 | 1 | 3 | 15/34 | 10343 |
| 65 | R.LVEVVEEAVII.- |  | 1 | 0.3438 | 35.120 | 0.000 | 606.86 | 0 | 2 | 8/20 | 10353 |
| 66 | K.ALYPELPIAVINLEK.N |  | 1 | 0.3008 | 53.450 | 0.000 | 561.66 | 1 | 3 | 9/28 | 10359 |
| 67 | R.YGLVAFASSLDQIGPITR.T |  | 2 | 0.8958 | 78.230 | 0.995 | 954.52 | 0 | 2 | 13/34 | 10363 |
| 68 | R.DLLQLLTDK.V |  | 1 | 0.5378 | 35.300 | 0.000 | 529.81 | 0 | 2 | 7/16 | 10365 |
| 69 | K.DFSEFLDFK.G |  | 1 | 0.9585 | 51.090 | 0.704 | 574.27 | 0 | 2 | 6/16 | 10367 |
| 70 | K.SFLDQLFR.L |  | 1 | 0.9825 | 33.870 | 0.966 | 513.27 | 0 | 2 | 7/14 | 10370 |
| 71 | K.LNPVFLLDEIDK.M |  | 1 | 0.4450 | 59.520 | 0.000 | 708.39 | 1 | 2 | 10/22 | 10371 |
| 72 | K.LFSEPLGIPFIYK.A |  | 1 | 0.5468 | 41.370 | 0.000 | 762.43 | 0 | 2 | 12/24 | 10390 |
| 73 | K.SVEELVADLDSVPENIR.T |  | 2 | 0.1835 | 64.650 | 0.000 | 942.98 | 1 | 2 | 10/32 | 10398 |
| 74 | K.NEITDTLAPFLYEK.T |  | 2 | 0.6325 | 86.180 | 0.000 | 827.42 | 0 | 2 | 19/26 | 10405 |
| 75 | R.AVASSFGLDTIDTLTGFK.F |  | 1 | 0.1605 | 106.340 | 0.000 | 921.98 | 0 | 2 | 14/34 | 10410 |
| 76 | R.TPNDETIVQLILPVR.T |  | 2 | 0.2791 | 65.600 | 0.000 | 854.49 | 0 | 2 | 8/28 | 10413 |
| 77 | K.TEAFWPNFLLK.E |  | 1 | 0.4481 | 47.490 | 0.000 | 683.36 | 1 | 2 | 8/20 | 10415 |
| 78 | M.ALNIEEIIASVK.E |  | 1 | 0.4224 | 53.290 | 0.000 | 433.92 | 0 | 3 | 14/22 | 10419 |
| 79 | R.QVSDLAEIFDQLK.L |  | 1 | 0.4109 | 72.840 | 0.000 | 753.40 | 0 | 2 | 10/24 | 10422 |
| 80 | K.GTLNVEFYPVLVGSAFK.N |  | 2 | 0.5469 | 40.690 | 0.000 | 614.33 | 0 | 3 | 16/32 | 10432 |
| 81 | K.WLWSEWITTVDHK.K |  | 1 | 0.7494 | 36.770 | 0.000 | 567.62 | 0 | 3 | 15/24 | 10433 |
| 82 | R.QTLNIDPEDELILFSSETK.K |  | 1 | 0.2110 | 60.110 | 0.000 | 1096.55 | 0 | 2 | 12/36 | 10435 |
| 83 | K.NAVLPVIAASLLASEEK.S |  | 2 | 0.3850 | 88.400 | 0.000 | 862.99 | 0 | 2 | 21/32 | 10442 |
| 84 | K.NIIALAAGITDGLGYGDNAK.A |  | 1 | 0.1243 | 93.500 | 0.000 | 974.01 | 0 | 2 | 16/38 | 10447 |
| 85 | R.VLNGLGIAIISTSQGVLTDK.E |  | 2 | 0.0874 | 90.280 | 0.000 | 1000.07 | 0 | 2 | 14/38 | 10451 |
| 86 | K.LFDLFLYPSER.V |  | 1 | 0.5429 | 34.520 | 0.000 | 700.37 | 0 | 2 | 12/20 | 10454 |
| 87 | K.NEQLEDVVQLI.- |  | 1 | 0.7759 | 39.740 | 0.000 | 650.34 | 1 | 2 | 14/20 | 10468 |
| 88 | R.TLLLTFFYR.Y |  | 1 | 0.7508 | 47.470 | 0.000 | 587.34 | 1 | 2 | 9/16 | 10472 |
| 89 | K.DYTPQEVSAIILQHLK.S |  | 1 | 0.8042 | 24.780 | 0.741 | 619.00 | 0 | 3 | 14/30 | 10474 |
| 90 | K.NSGASLIDLGDDVALLEFHSK.S |  | 1 | 0.2348 | 71.530 | 0.000 | 734.37 | 1 | 3 | 14/40 | 10481 |
| 91 | K.LSTLIDYLLK.N |  | 1 | 0.4960 | 31.000 | 0.000 | 589.86 | 0 | 2 | 13/18 | 10482 |
| 92 | K.TSGFWAQTISGFIPK.V |  | 1 | 0.0943 | 25.200 | 0.000 | 820.43 | 0 | 2 | 8/28 | 10487 |
| 93 | K.IHPDQLLLLIDEYR.K |  | 1 | 0.5841 | 35.880 | 0.000 | 579.99 | 2 | 3 | 13/26 | 10499 |
| 94 | R.LFFDINWVR.T |  | 2 | 0.9858 | 56.350 | 0.995 | 605.32 | 0 | 2 | 7/16 | 10505 |
| 95 | K.QVLGLSADELHEVFAEWNK.G |  | 1 | 0.6707 | 54.530 | 0.000 | 729.04 | 0 | 3 | 22/36 | 10518 |
| 96 | R.LPITVVNEFAWHNLFAR.Q |  | 1 | 0.8454 | 55.770 | 0.000 | 676.37 | 0 | 3 | 21/32 | 10523 |
| 97 | K.LNGFAEGTEVTPELLLETGVISK.L |  | 1 | 0.9010 | 57.500 | 0.897 | 806.43 | 1 | 3 | 21/44 | 10524 |
| 98 | R.DVWNTPNNIVNQLSELK.- |  | 2 | 0.6331 | 66.690 | 0.000 | 992.51 | 0 | 2 | 20/32 | 10530 |
| 99 | R.EEGLTEVLNPSEIFLEER.S |  | 1 | 0.2922 | 51.100 | 0.000 | 1052.53 | 0 | 2 | 12/34 | 10533 |
| 100 | K.EYELLYFLAK.T |  | 1 | 0.9124 | 30.740 | 0.949 | 644.85 | 1 | 2 | 6/18 | 10536 |
| 101 | R.VSQLTGIPLSIFSVGPDR.S |  | 1 | 0.1347 | 52.600 | 0.000 | 943.52 | 0 | 2 | 11/34 | 10540 |
| 102 | K.QLGASGVLEVGNNIQAIFGPR.S |  | 1 | 0.1368 | 54.050 | 0.000 | 714.06 | 0 | 3 | 15/40 | 10550 |
| 103 | K.SVICDVPTLSDVYTINEVLR.H |  | 2 | 0.2403 | 46.100 | 0.000 | 1147.09 | 1 | 2 | 12/38 | 10554 |
| 104 | R.LSSGVVWSLPITLPVDAQK.A |  | 2 | 0.1163 | 73.310 | 0.000 | 1005.57 | 0 | 2 | 11/36 | 10556 |
| 105 | R.SNILGTEFQSVLQCIR.C |  | 1 | 0.4852 | 84.050 | 0.000 | 932.98 | 1 | 2 | 10/30 | 10557 |
| 106 | K.NAPCLIFIDEIDAVGR.Q |  | 3 | 0.3216 | 72.810 | 0.000 | 901.96 | 0 | 2 | 12/30 | 10560 |
| 107 | K.QAFLSIDDIFK.E |  | 1 | 0.2393 | 24.690 | 0.000 | 648.84 | 2 | 2 | 7/20 | 10567 |
| 108 | R.FIEHGSVTALLEEIGLTK.Q |  | 1 | 0.3630 | 29.030 | 0.000 | 653.03 | 1 | 3 | 16/34 | 10581 |
| 109 | R.QVLDAYDVSPLPTTFLINPEGK.V |  | 1 | 0.1454 | 50.900 | 0.000 | 1209.13 | 2 | 2 | 15/42 | 10591 |
| 110 | K.DSGSGIPEEDLPFIFER.F |  | 1 | 0.2398 | 37.860 | 0.000 | 954.45 | 0 | 2 | 13/32 | 10598 |
| 111 | R.FDILVPITEFDR.D |  | 1 | 0.9094 | 21.270 | 0.987 | 488.93 | 0 | 3 | 7/22 | 10602 |
| 112 | K.TIGTIFNELFK.Q |  | 1 | 0.2893 | 22.700 | 0.000 | 641.86 | 0 | 2 | 8/20 | 10619 |
| 113 | K.GPVEETVYQLVGGLR.S |  | 2 | 0.2156 | 83.440 | 0.000 | 808.94 | 0 | 2 | 9/28 | 10621 |
| 114 | K.SSILNLLFR.F |  | 1 | 0.5404 | 43.510 | 0.000 | 531.82 | 1 | 2 | 6/16 | 10624 |
| 115 | K.AIFELFFR.R |  | 1 | 0.9259 | 53.570 | 0.725 | 521.79 | 1 | 2 | 6/14 | 10625 |
| 116 | K.ASLEAAGAEAVLANLEGSPEEIAAAAK.G |  | 2 | 0.1129 | 54.120 | 0.000 | 851.77 | 0 | 3 | 21/52 | 10664 |
| 117 | K.ALGDANVAGIIVADPLIIETCR.R |  | 1 | 0.0438 | 48.090 | 0.000 | 1141.11 | 0 | 2 | 10/42 | 10672 |
| 118 | R.SDFPNQVNNVLAFPGIFR.G |  | 1 | 0.2243 | 54.600 | 0.000 | 1018.02 | 0 | 2 | 10/34 | 10690 |
| 119 | R.VSIPPLSNTFISLIK.D |  | 2 | 0.2198 | 39.680 | 0.000 | 543.66 | 1 | 3 | 10/28 | 10695 |
| 120 | R.VFEEALEQAIEVLKPGGR.V |  | 1 | 0.1694 | 43.810 | 0.000 | 662.36 | 0 | 3 | 14/34 | 10699 |
| 121 | R.EPELDNLLLDSYFK.N |  | 1 | 0.5151 | 85.370 | 0.000 | 848.43 | 0 | 2 | 11/26 | 10705 |
| 122 | K.YGNTSAASIPISLVEELEAGK.I |  | 2 | 0.1620 | 39.260 | 0.000 | 717.04 | 1 | 3 | 16/40 | 10714 |
| 123 | K.TALETVDGLFLNPLVGETK.S |  | 2 | 0.8380 | 78.960 | 0.956 | 1009.05 | 1 | 2 | 13/36 | 10715 |
| 124 | K.TGEIVDVIDTIAS.- |  | 1 | 0.9307 | 72.770 | 0.965 | 666.85 | 0 | 2 | 9/24 | 10722 |
| 125 | K.QGAEQIEAILASFR.Q |  | 2 | 0.3249 | 64.040 | 0.000 | 766.91 | 0 | 2 | 10/26 | 10726 |
| 126 | K.EENVLFIADEIQTGLGR.T |  | 2 | 0.9013 | 85.790 | 0.974 | 952.49 | 0 | 2 | 12/32 | 10733 |
| 127 | K.SANVGEVELWNW.- |  | 1 | 0.7516 | 54.520 | 0.000 | 702.33 | 0 | 2 | 15/22 | 10742 |
| 128 | K.AIQIFDSWVGALNQADYR.T |  | 2 | 0.4313 | 102.300 | 0.000 | 1034.02 | 0 | 2 | 14/34 | 10745 |
| 129 | K.DLPDALFIIDPR.K |  | 2 | 0.9477 | 56.670 | 0.992 | 692.88 | 0 | 2 | 9/22 | 10752 |
| 130 | K.VPELAESISEGTIAQWLK.Q |  | 2 | 0.3583 | 100.700 | 0.000 | 986.03 | 0 | 2 | 14/34 | 10756 |
| 131 | K.ELELLDTPGILWPK.F |  | 2 | 0.4092 | 72.290 | 0.000 | 812.45 | 0 | 2 | 10/26 | 10765 |
| 132 | R.TAISLLGLLDSEADTM#NPEANYR.K |  | 1 | 0.4153 | 57.200 | 0.000 | 837.41 | 1 | 3 | 22/44 | 10766 |
| 133 | K.LSLDDLFEQIK.Q |  | 2 | 0.8882 | 65.680 | 0.749 | 660.86 | 0 | 2 | 7/20 | 10771 |
| 134 | K.DVAGADEEKQELVEVVEFLKDPR.K |  | 1 | 0.7585 | 33.170 | 0.977 | 872.44 | 0 | 3 | 20/44 | 10786 |
| 135 | R.YFAADYPLLFTEITAK.D |  | 2 | 0.3409 | 51.210 | 0.000 | 931.98 | 0 | 2 | 10/30 | 10795 |
| 136 | R.FDLIILDPPSFAR.T |  | 1 | 0.2875 | 47.560 | 0.000 | 752.41 | 0 | 2 | 8/24 | 10806 |
| 137 | M.SSELLDALTILEK.E |  | 1 | 0.3493 | 54.010 | 0.000 | 477.94 | 1 | 3 | 8/24 | 10808 |
| 138 | K.ICDQISDSILDEILK.K |  | 1 | 0.6821 | 42.170 | 0.510 | 587.97 | 1 | 3 | 9/28 | 10811 |
| 139 | K.EADLVFLLPLEAK.M |  | 1 | 0.1442 | 22.280 | 0.000 | 729.41 | 3 | 2 | 9/24 | 10818 |
| 140 | R.HYQLNLTAPVLLSQLFTK.R |  | 2 | 0.4233 | 63.210 | 0.000 | 1043.59 | 0 | 2 | 13/34 | 10822 |
| 141 | K.QAQDSVKEEAQR.S |  | 2 | 0.2339 | 64.300 | 0.000 | 694.84 | 0 | 2 | 8/22 | 1082 |
| 142 | K.DIDEEVETLAELTEK.T |  | 1 | 0.9298 | 68.700 | 0.981 | 867.42 | 2 | 2 | 11/28 | 10833 |
| 143 | K.LTVLDAASYDFTAIDDSVK.G |  | 5 | 0.2063 | 123.950 | 0.000 | 1022.51 | 0 | 2 | 13/36 | 10837 |
| 144 | R.IPYVEDIGTINEVISLAK.A |  | 2 | 0.2037 | 55.820 | 0.000 | 987.54 | 0 | 2 | 8/34 | 10839 |
| 145 | K.IDETTSLGSVFNILAESK.I |  | 1 | 0.1980 | 74.780 | 0.000 | 962.50 | 0 | 2 | 12/34 | 10849 |
| 146 | R.QIESLDPLSFFNYGAK.K |  | 2 | 0.3920 | 82.420 | 0.000 | 914.96 | 0 | 2 | 12/30 | 10850 |
| 147 | K.INSAFIITIPIFK.N |  | 2 | 0.2922 | 52.120 | 0.000 | 738.95 | 0 | 2 | 7/24 | 10852 |
| 148 | K.LNGELINWPESFNVFGK.L |  | 1 | 0.1658 | 38.770 | 0.000 | 982.50 | 2 | 2 | 6/32 | 10856 |
| 149 | K.KQGQFQTLSTGLQTLVEEIEK.Q |  | 1 | 0.1059 | 78.000 | 0.000 | 793.09 | 0 | 3 | 11/40 | 10859 |
| 150 | R.ESHPHDVQITK.E |  | 1 | 0.3533 | 49.180 | 0.000 | 645.82 | 1 | 2 | 8/20 | 1085 |
| 151 | R.TESLQIFYDLLK.Q |  | 1 | 0.4610 | 56.310 | 0.000 | 735.40 | 0 | 2 | 9/22 | 10861 |
| 152 | R.NGLGGLNAFAVTAIEAAYSK.G |  | 2 | 0.0622 | 121.310 | 0.000 | 984.02 | 0 | 2 | 13/38 | 10872 |
| 153 | K.ANVLYYDIIGPLIDK.M |  | 1 | 0.3076 | 70.120 | 0.000 | 853.97 | 1 | 2 | 11/28 | 10876 |
| 154 | R.LGGDDFDQVIIDHLVSEFKK.E |  | 1 | 0.9306 | 38.830 | 0.949 | 569.55 | 0 | 4 | 16/38 | 10879 |
| 155 | R.DLEAVVAIYNSTIASR.M |  | 1 | 0.2897 | 83.060 | 0.000 | 574.64 | 1 | 3 | 12/30 | 10884 |
| 156 | K.KAEIIAVGSELLLGQIANTNAQFISK.Q |  | 1 | 0.1304 | 23.430 | 0.000 | 910.18 | 1 | 3 | 24/50 | 10896 |
| 157 | K.YVELPDAYISVVESLR.H |  | 1 | 0.4438 | 64.950 | 0.000 | 926.99 | 1 | 2 | 10/30 | 10897 |
| 158 | R.SIGFVFQQFQLLPR.L |  | 2 | 0.5278 | 85.310 | 0.000 | 840.47 | 1 | 2 | 10/26 | 10906 |
| 159 | K.RLTTPLK.R |  | 1 | 0.1191 | 20.250 | 0.075 | 414.77 | 1 | 2 | 5/12 | 10910 |
| 160 | K.IITTSDEILQELVNLK.R |  | 2 | 0.4023 | 95.990 | 0.000 | 915.02 | 0 | 2 | 12/30 | 10915 |
| 161 | K.WALAPFDFLK.L |  | 1 | 0.2815 | 29.380 | 0.000 | 604.33 | 1 | 2 | 7/18 | 10930 |
| 162 | R.YTTIQNWANNVYNLVTK.R |  | 2 | 0.4741 | 100.550 | 0.000 | 1021.52 | 0 | 2 | 12/32 | 10947 |
| 163 | K.SNSAILAIDEALADIR.A |  | 2 | 0.1910 | 107.790 | 0.000 | 836.45 | 0 | 2 | 12/30 | 10948 |
| 164 | K.DVAGADEEKQELVEVVEFLK.D |  | 2 | 0.2374 | 49.980 | 0.000 | 1124.07 | 1 | 2 | 13/38 | 10952 |
| 165 | K.YTVLTGNQTGALLLHYLLSEK.K |  | 1 | 0.8836 | 40.250 | 0.965 | 778.76 | 0 | 3 | 16/40 | 10962 |
| 166 | R.TVSPLDIDTIIASVEK.T |  | 2 | 0.2959 | 86.040 | 0.000 | 850.97 | 0 | 2 | 12/30 | 10967 |
| 167 | K.STLLNLISGYISPTK.G |  | 2 | 0.9090 | 110.100 | 0.984 | 803.96 | 0 | 2 | 12/28 | 10970 |
| 168 | K.GATTDDLLVEAFAVVR.E |  | 2 | 0.4676 | 88.780 | 0.000 | 838.95 | 0 | 2 | 21/30 | 10973 |
| 169 | R.EGDILEAFVM#QEIER.T |  | 1 | 0.2824 | 43.550 | 0.000 | 897.93 | 4 | 2 | 9/28 | 10975 |
| 170 | K.NSQDGISLIQTAEGALTETHAILQR.V |  | 3 | 0.1186 | 107.790 | 0.000 | 1333.69 | 1 | 2 | 13/48 | 10981 |
| 171 | R.EALEQAINISQALFSGNIK.E |  | 1 | 0.4443 | 44.870 | 0.000 | 682.70 | 1 | 3 | 18/36 | 10992 |
| 172 | R.IPIDYLAQLLTAGDTDHIR.S |  | 1 | 0.6796 | 64.230 | 0.000 | 709.05 | 0 | 3 | 23/36 | 11005 |
| 173 | R.QAPSFDQLSTEVEILETGIK.V |  | 2 | 0.3085 | 69.260 | 0.000 | 735.71 | 0 | 3 | 11/38 | 11014 |
| 174 | R.DGVFELLDEIK.N |  | 1 | 0.9357 | 52.880 | 0.871 | 639.33 | 0 | 2 | 8/20 | 11015 |
| 175 | R.WEVLETAILPLIK.T |  | 2 | 0.7433 | 74.860 | 0.000 | 762.96 | 0 | 2 | 17/24 | 11016 |
| 176 | R.VILQDFTGVPAVVDLASLR.K |  | 2 | 0.1238 | 101.950 | 0.000 | 1007.07 | 1 | 2 | 12/36 | 11026 |
| 177 | K.NHEDEGKLEEAR.Q |  | 1 | 0.4169 | 43.660 | 0.000 | 476.22 | 0 | 3 | 9/22 | 1102 |
| 178 | K.ELFTLNDGLDVLIPR.G |  | 2 | 0.3469 | 95.630 | 0.000 | 857.97 | 1 | 2 | 10/28 | 11036 |
| 179 | R.VHVSGHGSQEELK.L |  | 1 | 0.3729 | 41.950 | 0.000 | 469.57 | 0 | 3 | 10/24 | 1103 |
| 180 | R.DESIIQFIEQYK.E |  | 1 | 0.5558 | 71.630 | 0.000 | 756.88 | 0 | 2 | 10/22 | 11043 |
| 181 | R.ISIVALFFANGDTEK.A |  | 2 | 0.2305 | 77.940 | 0.000 | 812.93 | 1 | 2 | 11/28 | 11059 |
| 182 | K.FFEEICLLDQAFVK.N |  | 2 | 0.7296 | 87.800 | 0.000 | 879.94 | 0 | 2 | 12/26 | 11074 |
| 183 | K.IFELMDAVDEYIPTPER.D |  | 4 | 0.4718 | 57.610 | 0.000 | 1019.49 | 1 | 2 | 12/32 | 11086 |
| 184 | K.LTYAEVVNVIDGQVLGGR.A |  | 1 | 0.3072 | 49.540 | 0.000 | 635.01 | 0 | 3 | 16/34 | 11092 |
| 185 | K.TIDFGGWELPVQFSSIK.K |  | 1 | 0.2044 | 80.840 | 0.000 | 962.49 | 1 | 2 | 12/32 | 11103 |
| 186 | K.DFLIDLIGYR.R |  | 1 | 0.5824 | 40.780 | 0.000 | 612.83 | 0 | 2 | 12/18 | 11116 |
| 187 | R.SGAVDIVVVDSVAALVPK.A |  | 6 | 0.2022 | 109.320 | 0.000 | 870.00 | 0 | 2 | 21/34 | 11120 |
| 188 | K.SRPHIANHLR.L |  | 1 | 0.3740 | 25.190 | 0.000 | 300.92 | 0 | 4 | 13/18 | 1113 |
| 189 | K.DGLVDVLQGEYNLLNR.E |  | 1 | 0.5669 | 45.270 | 0.000 | 606.65 | 1 | 3 | 15/30 | 11151 |
| 190 | K.EFSLGNIFHDSYESIFNSPLVHK.L |  | 1 | 0.5219 | 59.150 | 0.000 | 894.11 | 0 | 3 | 19/44 | 11166 |
| 191 | K.IDQLVYGLENIID.- |  | 1 | 0.5941 | 30.410 | 0.000 | 752.90 | 0 | 2 | 11/24 | 11169 |
| 192 | R.VPVPNVSLVDLVVDLK.T |  | 2 | 0.3411 | 109.510 | 0.000 | 853.51 | 0 | 2 | 14/30 | 11187 |
| 193 | K.ITELSVADALAFFK.D |  | 2 | 0.4238 | 94.950 | 0.000 | 762.92 | 0 | 2 | 11/26 | 11193 |
| 194 | K.HSTGGVGDTTTLVLAPLVAALDVPVAK.M |  | 2 | 0.1581 | 70.250 | 0.000 | 1301.73 | 0 | 2 | 27/52 | 11197 |
| 195 | K.CDM#VDDEELLELVEM#EVR.D |  | 1 | 0.5931 | 39.820 | 0.000 | 752.66 | 0 | 3 | 11/34 | 11200 |
| 196 | R.DVQELVDLLTEK.Q |  | 1 | 0.9190 | 60.480 | 0.858 | 467.92 | 0 | 3 | 8/22 | 11210 |
| 197 | K.YEGWEFLGAVLDDDR.T |  | 1 | 0.9639 | 102.000 | 0.972 | 892.91 | 0 | 2 | 11/28 | 11217 |
| 198 | K.IGGSIGEIATAAEQFLGK.S |  | 1 | 0.6697 | 39.610 | 0.899 | 587.98 | 1 | 3 | 18/34 | 11220 |
| 199 | K.NLLEDPEQLLLEG.- |  | 1 | 0.7514 | 58.280 | 0.000 | 741.89 | 0 | 2 | 9/24 | 11226 |
| 200 | R.NPLTGISGFIQLLQK.K |  | 2 | 0.4271 | 92.570 | 0.000 | 814.97 | 0 | 2 | 12/28 | 11235 |
| 201 | K.EFDLLYYLVQNPR.Q |  | 2 | 0.5227 | 55.570 | 0.000 | 835.43 | 0 | 2 | 7/24 | 11246 |
| 202 | K.ASYSLLGLATYFTAGEQEVR.A |  | 2 | 0.9150 | 50.560 | 0.979 | 726.04 | 0 | 3 | 14/38 | 11260 |
| 203 | R.VQPEVWDVLESVIK.E |  | 1 | 0.4682 | 49.260 | 0.000 | 820.95 | 0 | 2 | 11/26 | 11291 |
| 204 | K.NVVGTYSIEEFVQSLETPR.K |  | 1 | 0.1528 | 28.400 | 0.000 | 723.37 | 1 | 3 | 8/36 | 11300 |
| 205 | R.LQSLVQDLLDLSK.I |  | 1 | 0.7015 | 62.090 | 0.000 | 736.42 | 0 | 2 | 17/24 | 11304 |
| 206 | R.ALSEFVIEGIETTIPFHLK.L |  | 1 | 0.4422 | 48.890 | 0.000 | 715.39 | 0 | 3 | 17/36 | 11309 |
| 207 | R.GRPVTGPGNRPLK.S |  | 3 | 0.1131 | 52.040 | 0.000 | 450.26 | 0 | 3 | 14/24 | 1130 |
| 208 | K.LHELKPSEGSR.K |  | 1 | 0.6520 | 24.450 | 0.811 | 418.23 | 2 | 3 | 9/20 | 1131 |
| 209 | K.YVVYPLSELITYVAK.L |  | 2 | 0.8251 | 69.970 | 0.000 | 879.49 | 0 | 2 | 23/28 | 11328 |
| 210 | R.LHPSDFIYPIFVVEGLEGK.K |  | 1 | 0.4398 | 46.680 | 0.000 | 720.72 | 0 | 3 | 16/36 | 11342 |
| 211 | K.AAVSDALTYLFS.- |  | 1 | 0.3643 | 71.590 | 0.000 | 629.32 | 1 | 2 | 9/22 | 11349 |
| 212 | K.EGAEQIISEIQNQLQNLK.- |  | 2 | 0.8433 | 82.730 | 0.911 | 1028.04 | 0 | 2 | 11/34 | 11378 |
| 213 | R.DLFQAGLTTIVPIIGGGER.L |  | 2 | 0.2107 | 78.350 | 0.000 | 979.04 | 1 | 2 | 16/36 | 11381 |
| 214 | R.NSLIEAVCELDEELM#DK.Y |  | 2 | 0.4630 | 84.980 | 0.000 | 1012.46 | 1 | 2 | 10/32 | 11399 |
| 215 | K.QGVEIVVESTGFFTK.R |  | 14 | 0.2379 | 97.940 | 0.000 | 820.93 | 2 | 2 | 12/28 | 11404 |
| 216 | K.KIVLDDLIEEGFGALIK.E |  | 1 | 0.3204 | 44.030 | 0.000 | 625.03 | 0 | 3 | 18/32 | 11407 |
| 217 | K.DTSLASQILVAELFR.K |  | 1 | 0.5801 | 60.690 | 0.000 | 554.97 | 1 | 3 | 15/28 | 11409 |
| 218 | R.SLNLSNTAAILVYEALR.Q |  | 2 | 0.2607 | 84.820 | 0.000 | 924.51 | 0 | 2 | 13/32 | 11414 |
| 219 | R.SQNIVTDLLPALDSFER.A |  | 1 | 0.2103 | 60.290 | 0.000 | 959.50 | 1 | 2 | 8/32 | 11418 |
| 220 | R.VTNYIYDLASALHSFYNAEK.V |  | 1 | 0.3196 | 32.710 | 0.000 | 773.71 | 3 | 3 | 9/38 | 11429 |
| 221 | K.DITDAVELAEEVTNLK.Q |  | 2 | 0.4990 | 90.850 | 0.000 | 880.45 | 0 | 2 | 19/30 | 11444 |
| 222 | R.AAADFLQIPLYQYLGGFNSK.T |  | 2 | 0.4820 | 43.880 | 0.000 | 739.39 | 1 | 3 | 18/38 | 11449 |
| 223 | R.VAHAFEQATDHHK.A |  | 1 | 0.0893 | 20.700 | 0.000 | 497.58 | 0 | 3 | 7/24 | 1144 |
| 224 | K.VLYTEIFVQEAFNEIEAHAK.E |  | 1 | 0.6001 | 45.280 | 0.000 | 784.40 | 1 | 3 | 21/38 | 11468 |
| 225 | R.QAAITQEITEIVGGAAALE.- |  | 1 | 0.1554 | 23.390 | 0.000 | 943.00 | 0 | 2 | 15/36 | 11487 |
| 226 | R.ALLEEVPGVVEVEQVF.- |  | 1 | 0.2764 | 53.730 | 0.000 | 586.32 | 1 | 3 | 7/30 | 11525 |
| 227 | K.EHNISFIPYFPLVSGLLAGK.Y |  | 1 | 0.8941 | 50.210 | 0.997 | 734.74 | 1 | 3 | 11/38 | 11535 |
| 228 | K.QGQFQTLSTGLQTLVEEIEK.Q |  | 1 | 0.1433 | 68.450 | 0.000 | 1125.09 | 0 | 2 | 13/38 | 11536 |
| 229 | K.DAIGLFPFITSLK.S |  | 1 | 0.2873 | 33.290 | 0.000 | 711.41 | 1 | 2 | 13/24 | 11537 |
| 230 | R.VEALKDEAAKR.N |  | 1 | 0.4278 | 31.770 | 0.802 | 410.57 | 1 | 3 | 14/20 | 1155 |
| 231 | K.AGIGIEEILEQIVEK.V |  | 2 | 0.8871 | 101.510 | 0.992 | 820.96 | 0 | 2 | 10/28 | 11561 |
| 232 | K.AYTAQIAVLAVLASVAADK.N |  | 2 | 0.3716 | 100.300 | 0.000 | 938.03 | 0 | 2 | 23/36 | 11612 |
| 233 | K.LDVPLIEVFEIVK.E |  | 1 | 0.9607 | 82.010 | 0.944 | 757.45 | 0 | 2 | 11/24 | 11615 |
| 234 | R.NLVTIFETLADYGK.L |  | 1 | 0.3756 | 65.370 | 0.000 | 792.42 | 1 | 2 | 12/26 | 11621 |
| 235 | K.AAVFGCGPIGLLVIEALK.A |  | 1 | 0.2630 | 76.720 | 0.000 | 914.52 | 1 | 2 | 11/34 | 11662 |
| 236 | K.AVDSVFDTILDALK.N |  | 1 | 0.5365 | 80.900 | 0.000 | 753.91 | 0 | 2 | 19/26 | 11686 |
| 237 | K.IVLDDLIEEGFGALIK.E |  | 1 | 0.3948 | 56.530 | 0.000 | 582.33 | 1 | 3 | 12/30 | 11757 |
| 238 | K.RPYAPGPHGPGQR.K |  | 1 | 0.1104 | 57.040 | 0.000 | 463.91 | 0 | 3 | 9/24 | 1175 |
| 239 | R.HIVVKEEE.- |  | 1 | 0.2708 | 24.360 | 0.000 | 491.76 | 0 | 2 | 6/14 | 1187 |
| 240 | R.HPQHVTEAALR.G |  | 2 | 0.4859 | 54.030 | 0.000 | 420.23 | 0 | 3 | 8/20 | 1221 |
| 241 | K.EYEHSCDNLHR.K |  | 1 | 0.7795 | 31.560 | 0.000 | 487.21 | 0 | 3 | 16/20 | 1234 |
| 242 | R.SSYHADEQVNEASK.K |  | 1 | 0.1807 | 35.390 | 0.000 | 522.23 | 0 | 3 | 7/26 | 1332 |
| 243 | K.CIGCHTCSVTCK.N |  | 1 | 0.6267 | 39.520 | 0.000 | 494.87 | 0 | 3 | 12/22 | 1336 |
| 244 | K.ATGAGQQDQAEVDPNKR.V |  | 2 | 0.1287 | 91.070 | 0.000 | 892.93 | 0 | 2 | 12/32 | 1345 |
| 245 | K.HVNSVDHEYVR.Q |  | 1 | 0.5415 | 44.880 | 0.000 | 452.22 | 0 | 3 | 12/20 | 1352 |
| 246 | R.YGPHTM#AGDDPTK.Y |  | 1 | 0.4655 | 40.570 | 0.000 | 469.21 | 0 | 3 | 9/24 | 1357 |
| 247 | K.HHNTYVTNLNK.A |  | 1 | 0.9227 | 34.930 | 0.914 | 447.56 | 0 | 3 | 12/20 | 1372 |
| 248 | R.HEIEGHDM#EEIM#K.N |  | 1 | 0.6054 | 21.700 | 0.000 | 543.90 | 1 | 3 | 15/24 | 1380 |
| 249 | K.VRPSVKPICEK.C |  | 1 | 0.0787 | 22.370 | 0.000 | 328.94 | 1 | 4 | 10/20 | 1401 |
| 250 | K.SHANIGTIGHVDHGK.T |  | 2 | 0.4366 | 84.120 | 0.000 | 771.89 | 0 | 2 | 19/28 | 1402 |
| 251 | K.LEEVGASVEVK.- |  | 2 | 0.8098 | 36.330 | 0.766 | 580.31 | 0 | 2 | 12/20 | 14134 |
| 252 | R.LGNGEASDEDVRR.A |  | 1 | 0.2005 | 29.020 | 0.000 | 709.34 | 1 | 2 | 15/24 | 1413 |
| 253 | K.GTRPDTNEEIER.H |  | 2 | 0.6313 | 45.520 | 0.843 | 472.90 | 0 | 3 | 5/22 | 1445 |
| 254 | K.IKEYEHSCDNLHR.K |  | 2 | 0.4477 | 48.410 | 0.000 | 567.60 | 0 | 3 | 13/24 | 1446 |
| 255 | R.LTPHSSDDDDSSYR.G |  | 1 | 0.3411 | 52.590 | 0.000 | 532.23 | 0 | 3 | 10/26 | 1473 |
| 256 | K.SPTCTDQDVAAAK.E |  | 1 | 0.3734 | 72.540 | 0.000 | 682.31 | 0 | 2 | 10/24 | 1478 |
| 257 | K.KKIEYLDK.T |  | 1 | 0.0823 | 26.420 | 0.000 | 346.21 | 0 | 3 | 6/14 | 1479 |
| 258 | R.SVIGRPEDQR.V |  | 1 | 0.7199 | 30.550 | 0.949 | 578.81 | 1 | 2 | 4/18 | 1483 |
| 259 | K.SREEIEQVR.E |  | 1 | 0.8708 | 27.910 | 0.764 | 573.30 | 1 | 2 | 11/16 | 1490 |
| 260 | K.DVAEKQDDIKEEAK.G |  | 1 | 0.0503 | 20.820 | 0.000 | 539.94 | 0 | 3 | 10/26 | 1500 |
| 261 | K.NFVEKPPK.G |  | 1 | 0.1534 | 24.170 | 0.000 | 320.18 | 0 | 3 | 8/14 | 1521 |
| 262 | R.GAHYKPDYPER.N |  | 1 | 0.1797 | 36.580 | 0.000 | 444.88 | 0 | 3 | 7/20 | 1536 |
| 263 | R.FTHQGQICM#SANR.V |  | 1 | 0.3975 | 24.500 | 0.000 | 522.57 | 1 | 3 | 12/24 | 1565 |
| 264 | K.APNGQHVHEGVVESGTVQK.G |  | 1 | 0.2240 | 22.090 | 0.000 | 494.00 | 1 | 4 | 16/36 | 1575 |
| 265 | K.HPDPQFEGQTK.T |  | 1 | 0.3359 | 39.330 | 0.000 | 642.30 | 0 | 2 | 6/20 | 1610 |
| 266 | R.NFSDDEKNDDVK.V |  | 1 | 0.8525 | 21.550 | 0.980 | 475.88 | 0 | 3 | 12/22 | 1611 |
| 267 | K.NSDQTIHPNDDVNR.S |  | 2 | 0.5669 | 72.270 | 0.000 | 812.87 | 0 | 2 | 11/26 | 1620 |
| 268 | K.RLTNLTGESK.W |  | 1 | 0.7047 | 38.840 | 0.831 | 559.81 | 1 | 2 | 7/18 | 1621 |
| 269 | K.VVETEPGIKGDTASGGTKPAK.T |  | 1 | 0.1091 | 41.110 | 0.000 | 681.36 | 0 | 3 | 31/40 | 1675 |
| 270 | R.LGNGEASDEDVR.R |  | 1 | 0.3925 | 73.820 | 0.000 | 631.29 | 1 | 2 | 10/22 | 1684 |
| 271 | R.GAHIGTM#PLK.V |  | 1 | 0.1258 | 21.730 | 0.000 | 347.52 | 0 | 3 | 10/18 | 1697 |
| 272 | K.RAGYTAVISHR.S |  | 1 | 0.1841 | 28.470 | 0.000 | 410.90 | 0 | 3 | 12/20 | 1701 |
| 273 | K.LKEAFESEKPAR.S |  | 2 | 0.6346 | 38.040 | 0.971 | 468.92 | 1 | 3 | 9/22 | 1712 |
| 274 | R.HEIEGHEPM#AVVAK.L |  | 1 | 0.5295 | 36.570 | 0.000 | 521.59 | 1 | 3 | 14/26 | 1736 |
| 275 | R.FRGENTVVSR.N |  | 2 | 0.2368 | 48.920 | 0.000 | 582.81 | 1 | 2 | 8/18 | 1738 |
| 276 | R.LFNAHPTGAM#NK.S |  | 1 | 0.9001 | 26.250 | 0.936 | 439.55 | 0 | 3 | 10/22 | 1756 |
| 277 | K.IKEQLVER.V |  | 1 | 0.2500 | 30.850 | 0.000 | 338.87 | 0 | 3 | 5/14 | 1762 |
| 278 | K.QKEEIIQAAR.A |  | 2 | 0.2551 | 41.200 | 0.000 | 593.33 | 0 | 2 | 6/18 | 1770 |
| 279 | R.FHITETSK.M |  | 1 | 0.6291 | 20.840 | 0.619 | 321.50 | 0 | 3 | 5/14 | 1781 |
| 280 | R.LKEEGEKDFGPCK.T |  | 2 | 0.7938 | 83.280 | 0.958 | 768.87 | 0 | 2 | 11/24 | 1809 |
| 281 | K.KFEGLNTPHK.V |  | 2 | 0.8467 | 29.860 | 0.983 | 390.88 | 0 | 3 | 12/18 | 1814 |
| 282 | R.QISQLSGGQQQR.V |  | 1 | 0.3177 | 76.760 | 0.000 | 665.35 | 0 | 2 | 9/22 | 1823 |
| 283 | K.QVACENISDR.A |  | 1 | 0.9542 | 45.360 | 0.955 | 596.27 | 0 | 2 | 8/18 | 1828 |
| 284 | R.IEHIEEPKTEPGK.V |  | 2 | 0.2556 | 41.980 | 0.000 | 502.93 | 0 | 3 | 11/24 | 1835 |
| 285 | K.AQEVPEAIRK.A |  | 2 | 0.8318 | 36.080 | 0.996 | 570.82 | 0 | 2 | 8/18 | 1858 |
| 286 | R.KIIQTYQDENGK.L |  | 2 | 0.9237 | 78.780 | 0.970 | 718.87 | 1 | 2 | 9/22 | 1871 |
| 287 | K.HLDACITR.L |  | 1 | 0.2773 | 22.990 | 0.000 | 493.25 | 1 | 2 | 7/14 | 1889 |
| 288 | K.GYGEYKPVASNK.T |  | 1 | 0.1151 | 20.970 | 0.000 | 438.22 | 0 | 3 | 10/22 | 1897 |
| 289 | R.IQQVIHAAAQNGR.K |  | 2 | 0.9238 | 54.790 | 0.924 | 703.39 | 1 | 2 | 11/24 | 1917 |
| 290 | R.HEKLEIPASK.L |  | 1 | 0.4496 | 24.360 | 0.766 | 384.55 | 1 | 3 | 7/18 | 1919 |
| 291 | R.HYAHVDCPGHADYVK.N |  | 4 | 0.5114 | 66.720 | 0.000 | 884.90 | 1 | 2 | 12/28 | 1921 |
| 292 | K.YYNQYNSNVHR.G |  | 2 | 0.5251 | 37.320 | 0.000 | 729.33 | 1 | 2 | 6/20 | 1929 |
| 293 | K.VGAATETELK.E |  | 1 | 0.8340 | 38.490 | 0.956 | 509.77 | 1 | 2 | 6/18 | 1930 |
| 294 | K.VTTGTQSPTLGK.N |  | 1 | 0.7748 | 50.490 | 0.859 | 595.32 | 2 | 2 | 9/22 | 1941 |
| 295 | R.HEFAASVTNR.V |  | 1 | 0.2026 | 25.100 | 0.000 | 566.28 | 1 | 2 | 5/18 | 1958 |
| 296 | R.QESQTLIENAKK.L |  | 1 | 0.0767 | 23.160 | 0.000 | 463.58 | 2 | 3 | 5/22 | 1961 |
| 297 | R.RPLLADKPAESDASR.Y |  | 1 | 0.1485 | 31.470 | 0.000 | 542.62 | 2 | 3 | 17/28 | 1967 |
| 298 | K.TIEVSAERDPAK.L |  | 1 | 0.2217 | 55.010 | 0.000 | 658.35 | 0 | 2 | 8/22 | 1984 |
| 299 | R.HNIEELQR.F |  | 1 | 0.5969 | 31.140 | 0.000 | 519.77 | 0 | 2 | 6/14 | 1999 |
| 300 | K.SNRPAQLTAAVK.N |  | 1 | 0.4831 | 26.240 | 0.729 | 419.24 | 1 | 3 | 12/22 | 2001 |
| 301 | K.KVILTAPGK.N |  | 1 | 0.1732 | 32.500 | 0.000 | 463.80 | 1 | 2 | 6/16 | 2004 |
| 302 | K.VIEDAKEEIK.Q |  | 1 | 0.9162 | 45.970 | 0.909 | 587.32 | 1 | 2 | 8/18 | 2007 |
| 303 | R.KGLEPVEPR.E |  | 1 | 0.2490 | 37.510 | 0.000 | 512.79 | 0 | 2 | 5/16 | 2012 |
| 304 | R.VPHGGGGIIHDVK.V |  | 2 | 0.1360 | 48.270 | 0.000 | 643.35 | 0 | 2 | 11/24 | 2013 |
| 305 | R.LDHDTIVER.L |  | 1 | 0.4251 | 30.560 | 0.000 | 366.52 | 0 | 3 | 6/16 | 2031 |
| 306 | K.HLTDEEIER.L |  | 1 | 0.4917 | 40.260 | 0.000 | 571.28 | 1 | 2 | 5/16 | 2033 |
| 307 | K.LPSDVEER.E |  | 1 | 0.1853 | 25.670 | 0.000 | 472.74 | 0 | 2 | 7/14 | 2034 |
| 308 | R.YLHTSQDENAEPGS.- |  | 1 | 0.5272 | 41.300 | 0.000 | 774.33 | 0 | 2 | 8/26 | 2040 |
| 309 | R.LGYQAVSPHSK.T |  | 1 | 0.4243 | 68.070 | 0.000 | 593.81 | 1 | 2 | 9/20 | 2041 |
| 310 | K.LVNEPTVSK.D |  | 1 | 0.1473 | 20.970 | 0.000 | 493.78 | 1 | 2 | 5/16 | 2042 |
| 311 | K.DTTTGDTLCDEK.D |  | 1 | 0.2789 | 51.240 | 0.000 | 678.28 | 1 | 2 | 7/22 | 2060 |
| 312 | K.FTSVAVTGAHGK.T |  | 1 | 0.2110 | 43.460 | 0.000 | 587.81 | 0 | 2 | 11/22 | 2079 |
| 313 | K.VVETEPGIK.G |  | 1 | 0.2412 | 46.740 | 0.000 | 486.27 | 0 | 2 | 5/16 | 2093 |
| 314 | R.LKEHGLDSDAYK.W |  | 2 | 0.2683 | 51.040 | 0.000 | 459.23 | 0 | 3 | 10/22 | 2094 |
| 315 | R.TLADSSYNTR.R |  | 1 | 0.2846 | 32.770 | 0.000 | 564.27 | 1 | 2 | 7/18 | 2096 |
| 316 | K.FQASM#GAEAIHK.L |  | 1 | 0.7455 | 39.570 | 0.969 | 435.88 | 0 | 3 | 8/22 | 2097 |
| 317 | K.TEEDAAELAK.A |  | 1 | 0.1605 | 28.010 | 0.000 | 538.76 | 2 | 2 | 7/18 | 2103 |
| 318 | K.HQTLLGATGTGK.T |  | 1 | 0.1246 | 43.910 | 0.000 | 592.32 | 1 | 2 | 8/22 | 2107 |
| 319 | K.HVDIIAHPTGR.L |  | 1 | 0.4413 | 34.740 | 0.000 | 405.89 | 0 | 3 | 12/20 | 2115 |
| 320 | R.ALQVEADNEQTK.S |  | 1 | 0.9631 | 52.320 | 0.946 | 673.33 | 0 | 2 | 18/22 | 2122 |
| 321 | K.DDCGTQAYHDHGIVQK.A |  | 1 | 0.4095 | 40.180 | 0.000 | 615.27 | 0 | 3 | 13/30 | 2125 |
| 322 | R.EHILLSK.N |  | 1 | 0.5061 | 33.890 | 0.000 | 420.25 | 0 | 2 | 5/12 | 2136 |
| 323 | K.HFSWHETGK.L |  | 1 | 0.8403 | 24.750 | 0.753 | 376.85 | 0 | 3 | 7/16 | 2138 |
| 324 | K.HAISTFAPQK.N |  | 1 | 0.3066 | 37.990 | 0.000 | 550.30 | 0 | 2 | 8/18 | 2139 |
| 325 | K.NQGGDSSIVDDPSK.L |  | 1 | 0.2579 | 71.790 | 0.000 | 709.82 | 0 | 2 | 8/26 | 2152 |
| 326 | K.LAASAGIGAAK.V |  | 1 | 0.0576 | 23.250 | 0.000 | 465.27 | 0 | 2 | 8/20 | 2153 |
| 327 | R.AKGEAIKDVENATK.G |  | 1 | 0.7117 | 71.120 | 0.975 | 737.40 | 0 | 2 | 11/26 | 2155 |
| 328 | R.LGYRPNAVAR.G |  | 1 | 0.6860 | 25.700 | 0.736 | 372.88 | 0 | 3 | 8/18 | 2180 |
| 329 | R.RPLHILTR.R |  | 1 | 0.4220 | 44.780 | 0.000 | 335.88 | 0 | 3 | 7/14 | 2181 |
| 330 | R.LFAYHDAHR.Y |  | 1 | 0.6625 | 49.970 | 0.000 | 565.28 | 1 | 2 | 7/16 | 2194 |
| 331 | K.LAAAVLKPK.L |  | 1 | 0.1432 | 22.610 | 0.000 | 304.21 | 0 | 3 | 7/16 | 2195 |
| 332 | K.TKEGIAAGLSEK.D |  | 2 | 0.0791 | 38.080 | 0.000 | 602.33 | 0 | 2 | 8/22 | 2202 |
| 333 | K.QTAAYGHFGR.H |  | 1 | 0.5071 | 61.040 | 0.000 | 554.27 | 2 | 2 | 7/18 | 2214 |
| 334 | K.TSYGTYEDCK.D |  | 1 | 0.5770 | 61.930 | 0.000 | 612.25 | 1 | 2 | 8/18 | 2216 |
| 335 | K.IPM#TSDGLK.A |  | 1 | 0.8530 | 27.730 | 0.967 | 489.25 | 0 | 2 | 7/16 | 2225 |
| 336 | K.HNAVIEGAEVR.E |  | 1 | 0.6103 | 53.180 | 0.000 | 597.81 | 1 | 2 | 15/20 | 2228 |
| 337 | K.IGVFHNER.S |  | 2 | 0.5726 | 34.720 | 0.000 | 486.26 | 1 | 2 | 7/14 | 2231 |
| 338 | K.GGIVCDPR.D |  | 1 | 0.4651 | 42.260 | 0.000 | 437.22 | 0 | 2 | 6/14 | 2233 |
| 339 | R.KFAELGAR.I |  | 1 | 0.6278 | 28.910 | 0.730 | 446.26 | 0 | 2 | 6/14 | 2243 |
| 340 | R.APGTDQLTAR.A |  | 1 | 0.0555 | 21.960 | 0.000 | 515.27 | 5 | 2 | 7/18 | 2252 |
| 341 | R.QIEHFYK.A |  | 1 | 0.9339 | 20.500 | 0.833 | 482.75 | 0 | 2 | 5/12 | 2256 |
| 342 | K.SYPSQLSGGQK.Q |  | 1 | 0.8008 | 32.450 | 0.930 | 576.29 | 1 | 2 | 5/20 | 2268 |
| 343 | R.SAFTCNTPHGVCK.R |  | 1 | 0.5799 | 66.880 | 0.000 | 739.83 | 1 | 2 | 10/24 | 2276 |
| 344 | K.GLFSKPR.M |  | 1 | 0.8710 | 35.470 | 0.942 | 402.74 | 0 | 2 | 4/12 | 2284 |
| 345 | K.LTGQTFSSSPNK.F |  | 1 | 0.2572 | 54.050 | 0.000 | 633.82 | 1 | 2 | 9/22 | 2285 |
| 346 | R.NEVANEENNQDALQK.L |  | 2 | 0.5178 | 97.370 | 0.000 | 858.39 | 0 | 2 | 12/28 | 2286 |
| 347 | K.RPAEPEEFAR.K |  | 2 | 0.3508 | 26.620 | 0.000 | 601.30 | 0 | 2 | 9/18 | 2316 |
| 348 | R.LEGTNVDLGKK.I |  | 2 | 0.7097 | 54.720 | 0.956 | 587.33 | 0 | 2 | 7/20 | 2322 |
| 349 | R.SPAGGGQSTHLPLK.V |  | 2 | 0.7660 | 76.060 | 0.797 | 675.36 | 0 | 2 | 11/26 | 2323 |
| 350 | R.EGVTAADEEVAK.I |  | 1 | 0.1768 | 52.410 | 0.000 | 609.80 | 0 | 2 | 10/22 | 2332 |
| 351 | K.EAYETLSDDQKR.A |  | 1 | 0.4134 | 69.250 | 0.000 | 727.84 | 0 | 2 | 10/22 | 2335 |
| 352 | K.HVYLEEEPK.V |  | 1 | 0.7844 | 36.750 | 0.000 | 572.29 | 0 | 2 | 13/16 | 2337 |
| 353 | K.VAICEQTEDPK.A |  | 1 | 0.4923 | 87.230 | 0.000 | 645.31 | 1 | 2 | 8/20 | 2342 |
| 354 | R.LRPGEPPTVENAK.S |  | 2 | 0.8377 | 48.060 | 0.972 | 704.38 | 0 | 2 | 10/24 | 2343 |
| 355 | K.EIATTGTGLSK.L |  | 1 | 0.1519 | 46.110 | 0.000 | 539.29 | 1 | 2 | 7/20 | 2349 |
| 356 | K.RADEGIVEVK.I |  | 1 | 0.8668 | 51.390 | 0.969 | 558.31 | 0 | 2 | 7/18 | 2351 |
| 357 | R.TQVHPWER.E |  | 2 | 0.5711 | 36.150 | 0.000 | 526.77 | 0 | 2 | 7/14 | 2355 |
| 358 | R.IEVDKDIR.S |  | 1 | 0.4883 | 29.190 | 0.542 | 329.85 | 0 | 3 | 6/14 | 2362 |
| 359 | R.HNLSAAQLTEK.V |  | 2 | 0.3807 | 60.600 | 0.000 | 606.32 | 1 | 2 | 9/20 | 2364 |
| 360 | K.NVAVTSTM#GPGVK.V |  | 1 | 0.4706 | 63.330 | 0.000 | 638.83 | 0 | 2 | 16/24 | 2366 |
| 361 | K.QGEPDPESNQALK.V |  | 1 | 0.2494 | 54.800 | 0.000 | 706.84 | 0 | 2 | 10/24 | 2367 |
| 362 | R.IEDALNSTR.A |  | 1 | 0.9619 | 57.930 | 0.992 | 509.76 | 0 | 2 | 6/16 | 2372 |
| 363 | K.DVIGQAQTGTGK.T |  | 1 | 0.7851 | 44.300 | 0.913 | 587.81 | 1 | 2 | 9/22 | 2385 |
| 364 | R.GSEHPHEAQKPEVYELR.G |  | 2 | 0.3254 | 42.630 | 0.000 | 669.33 | 1 | 3 | 9/32 | 2396 |
| 365 | K.TFVGASPER.L |  | 1 | 0.9113 | 30.010 | 0.886 | 482.25 | 1 | 2 | 7/16 | 2398 |
| 366 | K.NKLEPLVQK.V |  | 1 | 0.1954 | 40.100 | 0.000 | 534.82 | 1 | 2 | 7/16 | 2403 |
| 367 | K.AIETATSQIK.E |  | 1 | 0.3772 | 40.650 | 0.000 | 531.30 | 1 | 2 | 11/18 | 2404 |
| 368 | R.VGANHQALPINR.A |  | 2 | 0.4046 | 56.540 | 0.000 | 645.36 | 1 | 2 | 16/22 | 2412 |
| 369 | K.GAGAETVGLR.F |  | 1 | 0.6449 | 62.280 | 0.569 | 465.75 | 0 | 2 | 8/18 | 2422 |
| 370 | K.TGEVQTLTEK.I |  | 1 | 0.4317 | 61.490 | 0.000 | 553.29 | 0 | 2 | 7/18 | 2429 |
| 371 | K.ELTYGDASSR.F |  | 1 | 0.2161 | 33.030 | 0.000 | 549.76 | 0 | 2 | 5/18 | 2430 |
| 372 | R.QATTYGVPR.I |  | 1 | 0.9332 | 43.100 | 0.939 | 496.76 | 0 | 2 | 8/16 | 2436 |
| 373 | K.TSAQSLTSNYNK.L |  | 1 | 0.4510 | 75.170 | 0.000 | 657.32 | 0 | 2 | 10/22 | 2447 |
| 374 | K.QIVISSDRPPK.E |  | 1 | 0.8179 | 42.880 | 0.934 | 620.36 | 0 | 2 | 6/20 | 2453 |
| 375 | K.HTGELTDSSFK.H |  | 1 | 0.2284 | 36.350 | 0.000 | 611.29 | 1 | 2 | 7/20 | 2454 |
| 376 | K.EAIDHPEIR.R |  | 1 | 0.3006 | 20.730 | 0.000 | 540.28 | 1 | 2 | 9/16 | 2455 |
| 377 | R.NKLEEHGLLR.Q |  | 1 | 0.2516 | 26.170 | 0.000 | 403.56 | 0 | 3 | 10/18 | 2460 |
| 378 | K.AQQSFDKPVEVQK.M |  | 1 | 0.9000 | 60.220 | 0.777 | 752.39 | 0 | 2 | 16/24 | 2470 |
| 379 | K.YIGSYAAR.M |  | 1 | 0.3324 | 27.970 | 0.000 | 450.73 | 0 | 2 | 7/14 | 2480 |
| 380 | R.AGYTAVISHR.S |  | 2 | 0.4366 | 54.470 | 0.000 | 537.79 | 0 | 2 | 9/18 | 2496 |
| 381 | M.AQVQYYGTGR.R |  | 1 | 0.2583 | 25.080 | 0.000 | 571.78 | 0 | 2 | 7/18 | 2510 |
| 382 | R.FHGGHIAAEIAK.R |  | 1 | 0.9031 | 31.000 | 0.933 | 417.56 | 0 | 3 | 9/22 | 2516 |
| 383 | K.AEEM#IEEGKELAK.I |  | 1 | 0.7948 | 20.960 | 0.988 | 498.24 | 2 | 3 | 12/24 | 2526 |
| 384 | K.KPTLIEVK.T |  | 1 | 0.1833 | 29.740 | 0.000 | 464.30 | 1 | 2 | 6/14 | 2531 |
| 385 | K.HGLGELPTK.N |  | 1 | 0.1579 | 23.420 | 0.000 | 476.27 | 1 | 2 | 6/16 | 2533 |
| 386 | K.EAVTYHNDFK.I |  | 1 | 0.6267 | 40.540 | 0.000 | 612.29 | 2 | 2 | 8/18 | 2544 |
| 387 | K.LASNENPYGCSEAAK.E |  | 1 | 0.4916 | 75.850 | 0.000 | 805.86 | 1 | 2 | 20/28 | 2546 |
| 388 | R.FIKPIDEK.M |  | 1 | 0.7541 | 31.220 | 0.745 | 330.53 | 1 | 3 | 6/14 | 2551 |
| 389 | K.AFAPQPVK.T |  | 1 | 0.2168 | 29.470 | 0.000 | 429.25 | 0 | 2 | 7/14 | 2554 |
| 390 | R.DADIATAEADKETR.I |  | 1 | 0.8845 | 83.080 | 0.959 | 753.36 | 1 | 2 | 11/26 | 2562 |
| 391 | K.ITVVDTGGSGESSGANK.V |  | 1 | 0.1033 | 100.540 | 0.000 | 789.88 | 0 | 2 | 12/32 | 2569 |
| 392 | K.LAASLEEHVK.E |  | 2 | 0.9604 | 60.130 | 0.985 | 548.80 | 1 | 2 | 8/18 | 2570 |
| 393 | K.QLTPESYK.N |  | 1 | 0.3107 | 34.030 | 0.000 | 483.25 | 1 | 2 | 5/14 | 2588 |
| 394 | K.NVTIHSDDDLR.F |  | 1 | 0.3683 | 28.110 | 0.000 | 642.81 | 1 | 2 | 9/20 | 2594 |
| 395 | R.GYGQGDQHIVVR.V |  | 1 | 0.3799 | 60.620 | 0.000 | 664.84 | 1 | 2 | 9/22 | 2617 |
| 396 | K.TTIESHLTTTSDR.I |  | 1 | 0.1808 | 20.500 | 0.000 | 487.91 | 0 | 3 | 10/24 | 2623 |
| 397 | R.SVEFTEEQQK.L |  | 1 | 0.4745 | 49.180 | 0.000 | 612.79 | 0 | 2 | 8/18 | 2625 |
| 398 | R.AIAAYTPR.E |  | 1 | 0.3051 | 27.290 | 0.000 | 431.74 | 0 | 2 | 7/14 | 2626 |
| 399 | K.IVSESGIGAGTR.R |  | 1 | 0.2913 | 70.280 | 0.000 | 573.81 | 0 | 2 | 10/22 | 2627 |
| 400 | R.CALAHDTFSAK.A |  | 1 | 0.4065 | 29.650 | 0.000 | 407.53 | 0 | 3 | 12/20 | 2628 |
| 401 | K.AGLELQEEDHSK.A |  | 2 | 0.5215 | 64.610 | 0.000 | 678.33 | 0 | 2 | 13/22 | 2629 |
| 402 | K.ENTTIVEGAGETDK.I |  | 1 | 0.2012 | 64.590 | 0.000 | 732.35 | 0 | 2 | 12/26 | 2634 |
| 403 | K.STAEAENVDIR.L |  | 1 | 0.9496 | 67.980 | 0.910 | 602.79 | 0 | 2 | 9/20 | 2635 |
| 404 | K.VVAEGGSYLHPK.V |  | 2 | 0.9520 | 78.910 | 0.991 | 628.83 | 1 | 2 | 10/22 | 2637 |
| 405 | K.HHADYNVLAAR.L |  | 1 | 0.1932 | 23.170 | 0.000 | 633.82 | 1 | 2 | 9/20 | 2651 |
| 406 | K.SVHEPLQTGIK.A |  | 2 | 0.8308 | 39.000 | 0.907 | 403.56 | 0 | 3 | 12/20 | 2653 |
| 407 | K.VIGVGGGGNNAVNR.M |  | 1 | 0.1805 | 84.910 | 0.000 | 642.34 | 1 | 2 | 12/26 | 2655 |
| 408 | K.AEIENNLER.E |  | 1 | 0.3723 | 26.390 | 0.000 | 544.27 | 0 | 2 | 6/16 | 2657 |
| 409 | K.GEAIKDVENATK.G |  | 1 | 0.8072 | 74.210 | 0.965 | 637.83 | 1 | 2 | 9/22 | 2661 |
| 410 | K.VHQYNVSCASSISQK.A |  | 1 | 0.4597 | 31.340 | 0.000 | 569.94 | 0 | 3 | 13/28 | 2669 |
| 411 | R.VTNCVLQTDDK.V |  | 1 | 0.6273 | 68.490 | 0.000 | 646.81 | 0 | 2 | 9/20 | 2673 |
| 412 | K.HGQLDAVIHR.D |  | 1 | 0.9005 | 57.380 | 0.880 | 382.54 | 0 | 3 | 7/18 | 2675 |
| 413 | K.VLDVNENEER.I |  | 1 | 0.5462 | 45.460 | 0.000 | 608.79 | 0 | 2 | 8/18 | 2676 |
| 414 | K.ADGIHIGQEDANAK.E |  | 1 | 0.8462 | 31.780 | 0.817 | 480.24 | 0 | 3 | 14/26 | 2680 |
| 415 | R.IEEETNLETR.V |  | 1 | 0.7465 | 63.060 | 0.000 | 617.30 | 0 | 2 | 8/18 | 2688 |
| 416 | R.LDNVVYK.L |  | 1 | 0.9499 | 30.330 | 0.765 | 425.74 | 0 | 2 | 10/12 | 2697 |
| 417 | R.WQSSNVNGYK.S |  | 1 | 0.1751 | 23.140 | 0.000 | 591.78 | 1 | 2 | 5/18 | 2698 |
| 418 | K.ENDPNLSGDDVR.E |  | 1 | 0.1894 | 41.110 | 0.000 | 665.80 | 0 | 2 | 7/22 | 2706 |
| 419 | K.HYFGNITK.E |  | 1 | 0.9449 | 24.640 | 0.973 | 490.25 | 0 | 2 | 9/14 | 2708 |
| 420 | R.QGGLGIIHK.N |  | 1 | 0.6861 | 25.070 | 0.675 | 308.19 | 0 | 3 | 10/16 | 2712 |
| 421 | R.KEELLVPK.E |  | 1 | 0.7268 | 24.700 | 0.830 | 478.29 | 1 | 2 | 6/14 | 2717 |
| 422 | K.VAIASDHGGVHIR.N |  | 1 | 0.2545 | 22.140 | 0.000 | 333.69 | 1 | 4 | 8/24 | 2733 |
| 423 | K.LFDVQHNR.F |  | 1 | 0.3249 | 22.640 | 0.000 | 343.51 | 1 | 3 | 7/14 | 2735 |
| 424 | K.RAGLLHDIGK.A |  | 1 | 0.2604 | 36.580 | 0.000 | 360.55 | 0 | 3 | 8/18 | 2737 |
| 425 | K.VKELLYEK.G |  | 1 | 0.8248 | 27.400 | 0.898 | 511.30 | 0 | 2 | 5/14 | 2742 |
| 426 | R.QESQTLIENAK.K |  | 1 | 0.9443 | 58.620 | 0.979 | 630.83 | 0 | 2 | 9/20 | 2743 |
| 427 | K.NSDAANITVTGR.G |  | 1 | 0.2131 | 56.540 | 0.000 | 609.81 | 0 | 2 | 9/22 | 2750 |
| 428 | R.AYAQTNPLR.E |  | 1 | 0.5137 | 37.630 | 0.000 | 517.28 | 0 | 2 | 10/16 | 2754 |
| 429 | K.IIAITNQK.G |  | 1 | 0.8803 | 30.610 | 0.937 | 450.78 | 0 | 2 | 4/14 | 2757 |
| 430 | R.YSEGLHQAIEAK.E |  | 1 | 0.3924 | 28.250 | 0.000 | 449.23 | 0 | 3 | 13/22 | 2764 |
| 431 | K.QDLNKPLPVEK.Q |  | 2 | 0.2298 | 38.850 | 0.000 | 640.86 | 0 | 2 | 9/20 | 2767 |
| 432 | R.DVTDLSGGQR.T |  | 1 | 0.2397 | 33.970 | 0.000 | 524.26 | 0 | 2 | 8/18 | 2785 |
| 433 | R.IGYVPQR.G |  | 1 | 0.9457 | 34.580 | 0.924 | 416.74 | 0 | 2 | 4/12 | 2797 |
| 434 | K.NVTAGANPVGVR.K |  | 1 | 0.2300 | 52.240 | 0.000 | 577.82 | 0 | 2 | 10/22 | 2798 |
| 435 | K.LEHIQLDK.E |  | 1 | 0.5998 | 22.150 | 0.698 | 332.52 | 0 | 3 | 8/14 | 2800 |
| 436 | K.NTTPESVTLQK.T |  | 1 | 0.1821 | 38.440 | 0.000 | 609.32 | 0 | 2 | 7/20 | 2814 |
| 437 | K.AGNIHVPIGK.V |  | 1 | 0.4455 | 34.740 | 0.000 | 335.87 | 0 | 3 | 14/18 | 2821 |
| 438 | K.NVTFTEEQR.S |  | 1 | 0.4692 | 39.980 | 0.000 | 562.27 | 1 | 2 | 5/16 | 2831 |
| 439 | R.ETANYLER.T |  | 1 | 0.8737 | 34.520 | 0.832 | 498.24 | 1 | 2 | 6/14 | 2832 |
| 440 | R.GALDTAGVENR.A |  | 1 | 0.1662 | 21.930 | 0.000 | 368.19 | 1 | 3 | 11/20 | 2848 |
| 441 | R.KEYETVFDDNKR.W |  | 1 | 0.7378 | 34.710 | 0.889 | 515.25 | 1 | 3 | 11/22 | 2853 |
| 442 | R.VIVSNVAGTTR.D |  | 1 | 0.3875 | 64.940 | 0.000 | 558.82 | 1 | 2 | 10/20 | 2854 |
| 443 | R.ALEEPIR.Q |  | 1 | 0.4417 | 31.150 | 0.000 | 414.23 | 0 | 2 | 4/12 | 2862 |
| 444 | R.IESIQPVNR.K |  | 1 | 0.4167 | 45.470 | 0.000 | 528.30 | 1 | 2 | 5/16 | 2866 |
| 445 | R.EVGNDYHATLNGQEAGK.T |  | 1 | 0.1477 | 32.370 | 0.000 | 601.61 | 0 | 3 | 13/32 | 2867 |
| 446 | R.IPAVQEAIKK.E |  | 1 | 0.1493 | 34.200 | 0.000 | 366.23 | 0 | 3 | 8/18 | 2868 |
| 447 | K.LQDDIYK.N |  | 1 | 0.9288 | 35.330 | 0.840 | 447.73 | 0 | 2 | 6/12 | 2873 |
| 448 | K.QVVENDYVK.N |  | 1 | 0.9155 | 31.050 | 0.984 | 547.28 | 0 | 2 | 6/16 | 2876 |
| 449 | K.VGNPTVEGATVTAK.V |  | 1 | 0.1641 | 72.040 | 0.000 | 672.36 | 0 | 2 | 12/26 | 2877 |
| 450 | K.LEEHGLLR.Q |  | 1 | 0.8616 | 28.660 | 0.625 | 322.85 | 0 | 3 | 6/14 | 2881 |
| 451 | K.VAPPFR.T |  | 1 | 0.2929 | 21.060 | 0.000 | 343.70 | 0 | 2 | 3/10 | 2885 |
| 452 | K.HFDESTVYR.V |  | 1 | 0.4190 | 36.560 | 0.000 | 577.27 | 0 | 2 | 6/16 | 2886 |
| 453 | K.SGEINQSEFK.Y |  | 1 | 0.3466 | 32.470 | 0.000 | 569.77 | 1 | 2 | 6/18 | 2890 |
| 454 | K.TLVTHQTGPDGQVIK.R |  | 2 | 0.9428 | 69.170 | 0.980 | 797.43 | 0 | 2 | 12/28 | 2892 |
| 455 | K.HVLVVGVEK.L |  | 1 | 0.2470 | 38.440 | 0.000 | 490.30 | 1 | 2 | 6/16 | 2893 |
| 456 | K.KVEEAYNFTK.N |  | 2 | 0.4665 | 74.640 | 0.000 | 614.81 | 0 | 2 | 8/18 | 2895 |
| 457 | R.VLVHSSIYDK.F |  | 1 | 0.2524 | 21.520 | 0.000 | 387.55 | 0 | 3 | 9/18 | 2896 |
| 458 | K.NQNTIEEINTK.Y |  | 1 | 0.6117 | 54.330 | 0.000 | 652.33 | 0 | 2 | 8/20 | 2901 |
| 459 | R.GTLTDHYDPTR.R |  | 1 | 0.4436 | 43.880 | 0.000 | 638.30 | 1 | 2 | 7/20 | 2915 |
| 460 | R.GLITEEER.Y |  | 1 | 0.4299 | 36.430 | 0.000 | 473.75 | 1 | 2 | 6/14 | 2917 |
| 461 | K.DAAVQSQEFEK.A |  | 1 | 0.9534 | 54.800 | 0.802 | 626.30 | 1 | 2 | 14/20 | 2918 |
| 462 | K.NKLEAPAPIDR.N |  | 1 | 0.6920 | 28.990 | 0.845 | 612.34 | 0 | 2 | 8/20 | 2919 |
| 463 | K.GTQYVSIAK.D |  | 1 | 0.2900 | 43.440 | 0.000 | 483.77 | 1 | 2 | 7/16 | 2928 |
| 464 | R.VATVEYDPNR.S |  | 1 | 0.2743 | 46.610 | 0.000 | 582.29 | 1 | 2 | 6/18 | 2929 |
| 465 | R.GGQGAILSAGDR.V |  | 1 | 0.1535 | 59.580 | 0.000 | 551.29 | 1 | 2 | 8/22 | 2930 |
| 466 | M.TGQLVQYGR.H |  | 1 | 0.5290 | 57.680 | 0.000 | 511.27 | 0 | 2 | 6/16 | 2939 |
| 467 | K.AQEVPEAIR.K |  | 1 | 0.2161 | 26.140 | 0.000 | 506.77 | 0 | 2 | 6/16 | 2946 |
| 468 | K.GLEPVEPR.E |  | 1 | 0.3975 | 30.520 | 0.000 | 448.75 | 0 | 2 | 5/14 | 2947 |
| 469 | R.DTVGTQYVEK.K |  | 1 | 0.2285 | 31.860 | 0.000 | 570.28 | 1 | 2 | 7/18 | 2948 |
| 470 | R.SQNHILSEQLK.E |  | 1 | 0.2438 | 21.110 | 0.000 | 432.90 | 0 | 3 | 7/20 | 2949 |
| 471 | K.STLASVPDCQR.I |  | 1 | 0.1818 | 24.520 | 0.000 | 617.30 | 1 | 2 | 7/20 | 2953 |
| 472 | K.VIGQVEVR.Q |  | 1 | 0.9307 | 40.510 | 0.690 | 450.27 | 0 | 2 | 7/14 | 2969 |
| 473 | K.TADGVDLSEAK.I |  | 1 | 0.7070 | 30.370 | 0.798 | 553.27 | 1 | 2 | 7/20 | 2974 |
| 474 | K.IQEVVSNVGR.V |  | 1 | 0.2190 | 37.000 | 0.000 | 550.81 | 1 | 2 | 7/18 | 2984 |
| 475 | K.VDSVSVQPVR.G |  | 1 | 0.3356 | 67.910 | 0.000 | 543.30 | 0 | 2 | 8/18 | 2988 |
| 476 | K.EGIAKEEAEELK.A |  | 1 | 0.2920 | 60.040 | 0.000 | 673.35 | 0 | 2 | 7/22 | 2989 |
| 477 | K.AHLYCDFHSDER.E |  | 1 | 0.5445 | 21.980 | 0.000 | 517.22 | 0 | 3 | 7/22 | 2998 |
| 478 | R.VDSTVNEVNAGGVAK.E |  | 1 | 0.2830 | 50.970 | 0.000 | 730.37 | 0 | 2 | 20/28 | 3001 |
| 479 | K.KVLLLNSR.D |  | 1 | 0.6281 | 21.440 | 0.831 | 471.81 | 0 | 2 | 4/14 | 3002 |
| 480 | K.VILTAPGK.N |  | 1 | 0.2143 | 31.530 | 0.000 | 399.76 | 0 | 2 | 5/14 | 3008 |
| 481 | R.QLVNHGHILVDGSR.V |  | 2 | 0.4017 | 42.060 | 0.000 | 515.61 | 1 | 3 | 14/26 | 3010 |
| 482 | K.FSFSDTHNR.L |  | 1 | 0.6298 | 45.660 | 0.000 | 555.75 | 0 | 2 | 7/16 | 3015 |
| 483 | R.GHGGYVGDDQIPR.L |  | 2 | 0.2025 | 60.440 | 0.000 | 685.83 | 1 | 2 | 7/24 | 3016 |
| 484 | K.CACGNEFETGSVK.E |  | 1 | 0.3494 | 72.020 | 0.000 | 729.80 | 1 | 2 | 10/24 | 3023 |
| 485 | K.ASIQSTEIAGTGEK.F |  | 1 | 0.1610 | 69.080 | 0.000 | 696.35 | 0 | 2 | 10/26 | 3033 |
| 486 | K.TQVTVEYDENNKPVR.I |  | 2 | 0.3032 | 77.650 | 0.000 | 896.45 | 0 | 2 | 11/28 | 3050 |
| 487 | R.NPLPNHAGNLVEK.A |  | 1 | 0.8236 | 45.970 | 0.833 | 701.88 | 0 | 2 | 9/24 | 3052 |
| 488 | K.TTAEEQSEIGGVNVHQR.L |  | 1 | 0.2543 | 38.750 | 0.000 | 618.97 | 1 | 3 | 12/32 | 3053 |
| 489 | K.AVAEALAEAK.D |  | 1 | 0.8026 | 35.440 | 0.803 | 486.77 | 0 | 2 | 8/18 | 3059 |
| 490 | R.EITDVVK.G |  | 1 | 0.9234 | 25.500 | 0.776 | 402.23 | 0 | 2 | 5/12 | 3060 |
| 491 | -.M#AEIRK.L |  | 1 | 0.6663 | 30.910 | 0.200 | 382.21 | 4 | 2 | 8/10 | 3062 |
| 492 | K.SINAVVEGAK.E |  | 1 | 0.9397 | 48.700 | 0.972 | 494.28 | 0 | 2 | 12/18 | 3070 |
| 493 | K.INVNTENQISSAK.A |  | 1 | 0.4144 | 70.890 | 0.000 | 709.37 | 0 | 2 | 11/24 | 3071 |
| 494 | K.VNYLHSEIK.T |  | 1 | 0.4844 | 23.410 | 0.000 | 368.20 | 0 | 3 | 9/16 | 3074 |
| 495 | K.DFIDKGDVK.F |  | 1 | 0.1625 | 25.730 | 0.000 | 346.18 | 0 | 3 | 5/16 | 3085 |
| 496 | K.QREDHIAGEITSAEEK.N |  | 3 | 0.7820 | 67.340 | 0.908 | 906.94 | 1 | 2 | 12/30 | 3087 |
| 497 | K.NNVDGVYNADPR.K |  | 1 | 0.4435 | 76.720 | 0.000 | 667.31 | 1 | 2 | 9/22 | 3094 |
| 498 | K.YHLSGEGVVK.T |  | 1 | 0.4746 | 46.450 | 0.000 | 363.53 | 0 | 3 | 7/18 | 3101 |
| 499 | K.EAQQLIEEQR.V |  | 1 | 0.4845 | 57.470 | 0.000 | 622.32 | 1 | 2 | 7/18 | 3106 |
| 500 | K.LSAIQQAEDR.D |  | 1 | 0.5745 | 61.430 | 0.000 | 565.79 | 0 | 2 | 8/18 | 3115 |
| 501 | K.IEDYVVENDRR.N |  | 1 | 0.1901 | 20.660 | 0.000 | 469.90 | 0 | 3 | 5/20 | 3116 |
| 502 | R.VGEEADALHEHGIR.Y |  | 1 | 0.3907 | 27.480 | 0.000 | 383.94 | 0 | 4 | 13/26 | 3119 |
| 503 | R.QVDAICHVVR.A |  | 1 | 0.4591 | 24.370 | 0.000 | 399.55 | 0 | 3 | 12/18 | 3132 |
| 504 | R.KPNVVVCTPSGSTAVER.R |  | 1 | 0.1971 | 27.440 | 0.000 | 600.98 | 1 | 3 | 20/32 | 3135 |
| 505 | R.FVIPSSHR.E |  | 1 | 0.3483 | 24.150 | 0.000 | 314.84 | 0 | 3 | 7/14 | 3136 |
| 506 | R.DAANSTPQFGDR.K |  | 1 | 0.2201 | 42.480 | 0.000 | 639.79 | 0 | 2 | 8/22 | 3140 |
| 507 | K.TTGANGTGTATFYK.V |  | 1 | 0.2141 | 66.790 | 0.000 | 695.34 | 1 | 2 | 11/26 | 3148 |
| 508 | R.LAEAGCQIVR.V |  | 1 | 0.3045 | 43.260 | 0.000 | 558.80 | 0 | 2 | 9/18 | 3158 |
| 509 | R.GAVVLPNGTGK.T |  | 1 | 0.0453 | 28.170 | 0.000 | 506.79 | 2 | 2 | 4/20 | 3160 |
| 510 | K.ELVDNTPKPLK.E |  | 1 | 0.6276 | 49.680 | 0.709 | 627.36 | 0 | 2 | 6/20 | 3162 |
| 511 | R.ADEGIVEVK.I |  | 1 | 0.8784 | 48.560 | 0.838 | 480.26 | 1 | 2 | 6/16 | 3163 |
| 512 | R.LGVDEADVK.L |  | 1 | 0.5109 | 41.980 | 0.000 | 473.25 | 0 | 2 | 6/16 | 3167 |
| 513 | R.VSVLGHIQR.G |  | 1 | 0.2185 | 29.480 | 0.000 | 336.87 | 0 | 3 | 5/16 | 3169 |
| 514 | K.NTFIEHNEINR.M |  | 2 | 0.7419 | 59.090 | 0.000 | 693.84 | 0 | 2 | 15/20 | 3171 |
| 515 | R.ACTELNIR.T |  | 1 | 0.9114 | 21.260 | 0.966 | 488.75 | 0 | 2 | 6/14 | 3172 |
| 516 | R.VTADLIQK.I |  | 1 | 0.8379 | 40.380 | 0.802 | 444.26 | 0 | 2 | 7/14 | 3173 |
| 517 | R.EAEFPGEER.I |  | 1 | 0.3450 | 20.020 | 0.529 | 532.24 | 0 | 2 | 6/16 | 3176 |
| 518 | K.LAAQGEENSGTQSIYK.L |  | 2 | 0.3589 | 99.910 | 0.000 | 848.41 | 0 | 2 | 12/30 | 3179 |
| 519 | R.DVFAQTNEAK.V |  | 1 | 0.2981 | 26.470 | 0.000 | 561.77 | 0 | 2 | 12/18 | 3182 |
| 520 | K.EEIEAYAEK.T |  | 1 | 0.3630 | 29.550 | 0.000 | 541.26 | 0 | 2 | 5/16 | 3195 |
| 521 | K.IAPNITVK.I |  | 1 | 0.1252 | 24.620 | 0.000 | 428.27 | 0 | 2 | 7/14 | 3198 |
| 522 | R.VGAIAANIR.A |  | 1 | 0.8885 | 53.810 | 0.795 | 442.77 | 0 | 2 | 8/16 | 3201 |
| 523 | K.EAICGCTTLSR.D |  | 1 | 0.4838 | 55.720 | 0.000 | 634.29 | 1 | 2 | 8/20 | 3202 |
| 524 | K.EIAPALM#TK.F |  | 1 | 0.3380 | 48.360 | 0.000 | 495.27 | 0 | 2 | 8/16 | 3210 |
| 525 | R.YILAPK.G |  | 1 | 0.3875 | 21.300 | 0.000 | 352.72 | 0 | 2 | 5/10 | 3214 |
| 526 | R.TSHITIVVSEK.K |  | 1 | 0.2605 | 41.380 | 0.000 | 607.34 | 0 | 2 | 8/20 | 3217 |
| 527 | K.TNVGELSVK.A |  | 1 | 0.7874 | 50.250 | 0.769 | 473.76 | 1 | 2 | 6/16 | 3220 |
| 528 | R.EAQIIEEAGQK.G |  | 1 | 0.1985 | 42.090 | 0.000 | 608.31 | 1 | 2 | 8/20 | 3222 |
| 529 | K.YGVSVPEGK.V |  | 1 | 0.8671 | 35.510 | 0.896 | 468.25 | 0 | 2 | 6/16 | 3234 |
| 530 | R.VSTEGNWPR.D |  | 1 | 0.9628 | 43.410 | 0.945 | 523.26 | 1 | 2 | 8/16 | 3235 |
| 531 | K.EGIAAGLSEK.D |  | 1 | 0.8021 | 36.160 | 1.000 | 487.76 | 1 | 2 | 8/18 | 3237 |
| 532 | K.AEIEGDM#GDSHVGLQAR.L |  | 2 | 0.1772 | 70.700 | 0.000 | 900.91 | 0 | 2 | 10/32 | 3248 |
| 533 | K.DLNVESTGDTSAATK.V |  | 1 | 0.7260 | 69.770 | 0.892 | 754.86 | 1 | 2 | 11/28 | 3258 |
| 534 | R.QVFETNNVDNCSR.Y |  | 1 | 0.5046 | 84.190 | 0.000 | 791.85 | 0 | 2 | 11/24 | 3259 |
| 535 | R.NIAIIAHVDHGK.T |  | 1 | 0.2032 | 22.390 | 0.000 | 322.68 | 1 | 4 | 8/22 | 3268 |
| 536 | R.VLSYIQK.G |  | 1 | 0.8514 | 24.420 | 0.978 | 425.76 | 0 | 2 | 5/12 | 3274 |
| 537 | R.LGEITSDSVAK.G |  | 1 | 0.2377 | 40.170 | 0.000 | 560.30 | 1 | 2 | 5/20 | 3275 |
| 538 | R.KAFDQGEWR.T |  | 1 | 0.4004 | 40.640 | 0.000 | 568.78 | 1 | 2 | 6/16 | 3277 |
| 539 | R.FEPELEEEKR.N |  | 1 | 0.3524 | 41.740 | 0.000 | 653.32 | 0 | 2 | 8/18 | 3279 |
| 540 | R.NTGHTEEDKLDVLK.S |  | 1 | 0.1855 | 32.570 | 0.000 | 533.61 | 0 | 3 | 11/26 | 3284 |
| 541 | K.LTELVNPK.K |  | 1 | 0.3638 | 24.560 | 0.000 | 457.27 | 0 | 2 | 5/14 | 3285 |
| 542 | R.HSEYGNDLLK.N |  | 1 | 0.4035 | 35.100 | 0.000 | 588.29 | 0 | 2 | 6/18 | 3287 |
| 543 | K.QSVDGGSYSEIQNSSAK.E |  | 1 | 0.1673 | 82.610 | 0.000 | 878.90 | 0 | 2 | 10/32 | 3292 |
| 544 | K.IDIPR.I |  | 1 | 0.9077 | 32.960 | 0.512 | 307.19 | 0 | 2 | 4/8 | 3297 |
| 545 | R.VVTPTNLTDK.Q |  | 1 | 0.0952 | 26.910 | 0.000 | 544.30 | 1 | 2 | 4/18 | 3298 |
| 546 | K.RKPDFGQFPR.F |  | 1 | 0.1055 | 39.060 | 0.000 | 416.56 | 0 | 3 | 8/18 | 3300 |
| 547 | K.TYENPDWK.A |  | 1 | 0.6859 | 30.100 | 0.000 | 526.74 | 0 | 2 | 9/14 | 3308 |
| 548 | R.VAFGNVNAR.D |  | 1 | 0.8262 | 35.400 | 0.996 | 474.26 | 1 | 2 | 7/16 | 3314 |
| 549 | R.VNSISAGPIR.T |  | 1 | 0.2833 | 42.330 | 0.000 | 507.29 | 0 | 2 | 7/18 | 3327 |
| 550 | K.APIAIIDKR.R |  | 2 | 0.3876 | 47.450 | 0.000 | 498.81 | 0 | 2 | 7/16 | 3329 |
| 551 | K.NAAAGLNLGGGK.T |  | 1 | 0.0960 | 42.060 | 0.000 | 521.79 | 0 | 2 | 9/22 | 3334 |
| 552 | K.INTGDFVKPK.A |  | 1 | 0.1461 | 25.900 | 0.000 | 373.54 | 1 | 3 | 11/18 | 3335 |
| 553 | K.TEIQPGDDVPR.A |  | 1 | 0.3344 | 34.350 | 0.000 | 613.80 | 1 | 2 | 9/20 | 3342 |
| 554 | K.LRPDVVIR.F |  | 1 | 0.2665 | 28.100 | 0.000 | 323.21 | 1 | 3 | 9/14 | 3347 |
| 555 | R.LEGTNVDLGK.K |  | 1 | 0.8339 | 46.030 | 0.828 | 523.28 | 0 | 2 | 8/18 | 3351 |
| 556 | K.VETLVASDNVK.G |  | 1 | 0.8409 | 55.490 | 0.965 | 587.82 | 1 | 2 | 6/20 | 3365 |
| 557 | R.VRDLFENAK.K |  | 1 | 0.1614 | 28.550 | 0.000 | 546.30 | 0 | 2 | 6/16 | 3372 |
| 558 | K.VNSPGGGVYESAEIHK.K |  | 1 | 0.1368 | 24.200 | 0.000 | 548.61 | 1 | 3 | 14/30 | 3374 |
| 559 | K.VAVIGQSGNLTPADKK.L |  | 1 | 0.6372 | 89.120 | 0.889 | 799.45 | 0 | 2 | 13/30 | 3378 |
| 560 | R.HKPSADYLFR.S |  | 1 | 0.0861 | 26.680 | 0.000 | 411.88 | 0 | 3 | 7/18 | 3383 |
| 561 | K.TQYGYHIIK.K |  | 1 | 0.3640 | 20.960 | 0.000 | 561.80 | 1 | 2 | 6/16 | 3385 |
| 562 | K.AFEVDKEAYAK.G |  | 2 | 0.8727 | 47.380 | 0.987 | 635.82 | 1 | 2 | 9/20 | 3394 |
| 563 | K.VEEGQTVYIEK.L |  | 1 | 0.9576 | 67.160 | 0.997 | 647.83 | 0 | 2 | 7/20 | 3395 |
| 564 | K.LAVGTIGASK.R |  | 1 | 0.0958 | 36.400 | 0.000 | 458.78 | 0 | 2 | 9/18 | 3413 |
| 565 | R.FLGHNELER.R |  | 1 | 0.9190 | 22.940 | 0.974 | 372.19 | 0 | 3 | 8/16 | 3414 |
| 566 | R.TPNPDVLCNK.E |  | 1 | 0.1987 | 31.550 | 0.000 | 579.28 | 1 | 2 | 8/18 | 3416 |
| 567 | K.EQVQEVLDK.L |  | 1 | 0.8803 | 30.440 | 0.807 | 544.28 | 0 | 2 | 5/16 | 3417 |
| 568 | R.IEIIR.D |  | 1 | 0.6935 | 35.450 | 0.000 | 322.21 | 0 | 2 | 4/8 | 3418 |
| 569 | K.FGAEPVNYSK.V |  | 1 | 0.9350 | 45.590 | 0.885 | 556.27 | 0 | 2 | 13/18 | 3423 |
| 570 | K.GEVVEELR.L |  | 1 | 0.9388 | 38.270 | 0.859 | 465.75 | 0 | 2 | 6/14 | 3426 |
| 571 | R.GTVNHFGLDGR.V |  | 1 | 0.1696 | 26.370 | 0.000 | 391.53 | 0 | 3 | 4/20 | 3428 |
| 572 | K.GGAEENYDELR.Y |  | 1 | 0.4342 | 53.440 | 0.000 | 626.78 | 0 | 2 | 7/20 | 3436 |
| 573 | R.LTERPQIIVANK.M |  | 1 | 0.0827 | 21.480 | 0.000 | 461.28 | 0 | 3 | 7/22 | 3445 |
| 574 | K.ALNDVLNHPK.V |  | 1 | 0.2276 | 22.920 | 0.000 | 374.21 | 1 | 3 | 6/18 | 3455 |
| 575 | R.LNHLSDIQVK.E |  | 1 | 0.2679 | 23.380 | 0.000 | 389.56 | 0 | 3 | 6/18 | 3456 |
| 576 | R.NKPLDETVNLK.S |  | 2 | 0.1163 | 29.930 | 0.000 | 424.24 | 0 | 3 | 6/20 | 3465 |
| 577 | K.LLIGER.T |  | 1 | 0.6175 | 20.040 | 0.444 | 350.72 | 0 | 2 | 4/10 | 3467 |
| 578 | K.ALDAAVHLAK.E |  | 2 | 0.7492 | 49.660 | 0.804 | 504.80 | 0 | 2 | 6/18 | 3477 |
| 579 | R.AYDIYSR.L |  | 1 | 0.5363 | 31.770 | 0.000 | 444.22 | 0 | 2 | 9/12 | 3488 |
| 580 | K.TSLNEAVVTGK.G |  | 1 | 0.3550 | 55.650 | 0.000 | 559.81 | 0 | 2 | 9/20 | 3491 |
| 581 | K.VFDEQDGIYKK.I |  | 1 | 0.8578 | 40.650 | 0.847 | 447.89 | 1 | 3 | 15/20 | 3496 |
| 582 | R.IAQLQDNIK.K |  | 1 | 0.8342 | 29.090 | 0.888 | 521.80 | 0 | 2 | 5/16 | 3503 |
| 583 | K.FVNATGLENK.D |  | 1 | 0.9123 | 47.100 | 0.989 | 546.79 | 0 | 2 | 7/18 | 3507 |
| 584 | K.TYAQNVISNAK.R |  | 1 | 0.9597 | 69.760 | 0.963 | 604.82 | 0 | 2 | 9/20 | 3509 |
| 585 | R.VHIQVGTLSK.A |  | 1 | 0.1699 | 25.860 | 0.000 | 541.32 | 0 | 2 | 8/18 | 3511 |
| 586 | R.AFSDDNITHVSGK.V |  | 2 | 0.4000 | 76.020 | 0.000 | 695.83 | 0 | 2 | 10/24 | 3513 |
| 587 | R.AGQWDYER.V |  | 1 | 0.6826 | 47.510 | 0.000 | 512.73 | 0 | 2 | 7/14 | 3514 |
| 588 | K.LFNGPVR.G |  | 1 | 0.9552 | 32.270 | 0.868 | 401.73 | 0 | 2 | 5/12 | 3515 |
| 589 | K.NICSWAK.N |  | 1 | 0.3636 | 24.020 | 0.000 | 439.71 | 0 | 2 | 6/12 | 3528 |
| 590 | K.NIIHGSDSLESAER.E |  | 1 | 0.1270 | 22.690 | 0.000 | 509.92 | 0 | 3 | 7/26 | 3531 |
| 591 | K.ITVFR.Y |  | 1 | 0.3004 | 20.440 | 0.000 | 318.20 | 0 | 2 | 3/8 | 3532 |
| 592 | R.AQVEETTSEFDR.E |  | 1 | 0.5039 | 79.350 | 0.000 | 706.32 | 0 | 2 | 8/22 | 3541 |
| 593 | K.LNTNENYTGLK.K |  | 1 | 0.3904 | 60.720 | 0.000 | 633.82 | 1 | 2 | 7/20 | 3543 |
| 594 | K.VGSDVFER.L |  | 1 | 0.7579 | 25.710 | 0.889 | 454.73 | 0 | 2 | 7/14 | 3549 |
| 595 | R.QTSGICEELR.E |  | 1 | 0.9375 | 46.140 | 0.924 | 596.78 | 0 | 2 | 9/18 | 3550 |
| 596 | K.DILQSDSAPQEK.V |  | 1 | 0.3418 | 39.900 | 0.000 | 665.83 | 0 | 2 | 11/22 | 3553 |
| 597 | K.VFAEISR.R |  | 1 | 0.4232 | 26.020 | 0.416 | 411.23 | 1 | 2 | 4/12 | 3554 |
| 598 | R.LFTSESVTEGHPDK.I |  | 1 | 0.3809 | 75.000 | 0.000 | 773.87 | 0 | 2 | 10/26 | 3557 |
| 599 | K.WVNNQYVK.K |  | 1 | 0.3688 | 22.560 | 0.000 | 525.77 | 0 | 2 | 4/14 | 3562 |
| 600 | K.HIGTPHEVLEEGQTVK.V |  | 1 | 0.4237 | 40.140 | 0.000 | 591.98 | 0 | 3 | 14/30 | 3578 |
| 601 | K.LAGGVAVIK.V |  | 1 | 0.1646 | 36.930 | 0.000 | 414.27 | 1 | 2 | 8/16 | 3581 |
| 602 | K.HIEVQVIGDK.Q |  | 1 | 0.4141 | 40.840 | 0.000 | 569.32 | 0 | 2 | 7/18 | 3585 |
| 603 | K.TYNIGDTVK.T |  | 1 | 0.2386 | 32.560 | 0.000 | 505.76 | 0 | 2 | 5/16 | 3592 |
| 604 | K.QFIESGTYK.H |  | 1 | 0.2424 | 20.060 | 0.000 | 536.77 | 0 | 2 | 7/16 | 3593 |
| 605 | R.HAGYAFDTDVK.V |  | 1 | 0.9779 | 73.970 | 0.934 | 612.29 | 0 | 2 | 15/20 | 3594 |
| 606 | R.GYAAEGIHGDLTQAK.R |  | 2 | 0.4651 | 90.780 | 0.000 | 765.88 | 0 | 2 | 21/28 | 3595 |
| 607 | K.IAFTGSTEIGKK.I |  | 2 | 0.1350 | 60.430 | 0.000 | 626.35 | 0 | 2 | 8/22 | 3597 |
| 608 | K.LIEVVR.N |  | 1 | 0.7710 | 29.390 | 0.474 | 364.74 | 0 | 2 | 4/10 | 3604 |
| 609 | R.YLNEGFSGGEK.K |  | 1 | 0.2875 | 44.070 | 0.000 | 600.78 | 0 | 2 | 7/20 | 3608 |
| 610 | R.VSSAELSDAVK.V |  | 1 | 0.7484 | 38.320 | 0.826 | 553.29 | 0 | 2 | 9/20 | 3611 |
| 611 | R.IQGIAAEGDVGSGR.A |  | 1 | 0.1820 | 70.490 | 0.000 | 665.34 | 0 | 2 | 12/26 | 3617 |
| 612 | K.AAENGVLYGTPTK.H |  | 1 | 0.2135 | 68.270 | 0.000 | 660.84 | 0 | 2 | 9/24 | 3618 |
| 613 | K.LAEEDPTFR.T |  | 1 | 0.4350 | 37.450 | 0.000 | 539.26 | 0 | 2 | 6/16 | 3624 |
| 614 | R.EDHIAGEITSAEEK.N |  | 1 | 0.0783 | 22.080 | 0.000 | 510.24 | 1 | 3 | 5/26 | 3643 |
| 615 | K.NLLQTNGNGDVR.H |  | 1 | 0.2628 | 45.980 | 0.000 | 650.83 | 1 | 2 | 6/22 | 3649 |
| 616 | K.YHTNAGSNYVGATIGEK.A |  | 1 | 0.0487 | 21.820 | 0.000 | 594.62 | 1 | 3 | 9/32 | 3657 |
| 617 | K.FSNDVIVK.I |  | 1 | 0.9007 | 34.790 | 0.984 | 461.26 | 1 | 2 | 6/14 | 3659 |
| 618 | K.ALAELPSR.E |  | 1 | 0.3434 | 28.480 | 0.000 | 428.75 | 1 | 2 | 5/14 | 3660 |
| 619 | R.EDQDAFAVR.S |  | 1 | 0.3773 | 40.440 | 0.000 | 525.75 | 1 | 2 | 8/16 | 3662 |
| 620 | K.ISSALITEK.F |  | 1 | 0.7997 | 27.300 | 0.984 | 481.28 | 0 | 2 | 7/16 | 3667 |
| 621 | K.TAEEVDEALTK.K |  | 1 | 0.9100 | 69.080 | 0.901 | 603.30 | 1 | 2 | 7/20 | 3669 |
| 622 | R.RLGISLSGTGK.E |  | 1 | 0.4914 | 34.490 | 0.915 | 544.82 | 0 | 2 | 6/20 | 3676 |
| 623 | K.CACGNEFETGSVKEEVR.V |  | 1 | 0.1740 | 25.390 | 0.000 | 657.96 | 0 | 3 | 11/32 | 3678 |
| 624 | R.TGEYVNTLAEK.Y |  | 1 | 0.3258 | 52.690 | 0.000 | 612.81 | 0 | 2 | 7/20 | 3679 |
| 625 | R.TQAVVVAIK.N |  | 1 | 0.2054 | 32.060 | 0.000 | 464.80 | 1 | 2 | 7/16 | 3681 |
| 626 | R.DLDEEDPKEIEASK.Y |  | 1 | 0.2895 | 63.370 | 0.000 | 809.38 | 0 | 2 | 15/26 | 3683 |
| 627 | K.FWGAQTQR.S |  | 1 | 0.9479 | 25.230 | 0.910 | 497.25 | 0 | 2 | 7/14 | 3688 |
| 628 | R.KESLELLK.T |  | 1 | 0.7251 | 42.380 | 0.572 | 320.53 | 0 | 3 | 6/14 | 3706 |
| 629 | K.ITNEENYVIQK.L |  | 1 | 0.6969 | 67.110 | 0.000 | 675.85 | 0 | 2 | 9/20 | 3714 |
| 630 | K.AIVNQDDETVVR.L |  | 1 | 0.4618 | 64.790 | 0.000 | 679.85 | 0 | 2 | 8/22 | 3728 |
| 631 | R.DNAEDNEIIAEQEKR.Q |  | 2 | 0.2296 | 94.080 | 0.000 | 887.42 | 0 | 2 | 10/28 | 3730 |
| 632 | R.LQDAEAVTIGK.L |  | 1 | 0.2339 | 44.720 | 0.000 | 572.81 | 0 | 2 | 9/20 | 3734 |
| 633 | K.HFIAGIVK.H |  | 1 | 0.7066 | 34.640 | 0.318 | 442.77 | 0 | 2 | 9/14 | 3745 |
| 634 | K.QIEVIGTAR.N |  | 1 | 0.8249 | 37.880 | 0.759 | 493.79 | 0 | 2 | 8/16 | 3748 |
| 635 | R.NLSIIK.E |  | 1 | 0.8675 | 29.050 | 0.667 | 344.22 | 1 | 2 | 5/10 | 3751 |
| 636 | K.GLGHAVWCAR.N |  | 1 | 0.2825 | 21.570 | 0.000 | 376.19 | 1 | 3 | 9/18 | 3752 |
| 637 | R.SLNVALR.Q |  | 1 | 0.7931 | 33.420 | 0.532 | 386.74 | 0 | 2 | 5/12 | 3757 |
| 638 | K.LIESIPK.M |  | 1 | 0.6284 | 20.640 | 0.632 | 400.25 | 0 | 2 | 4/12 | 3768 |
| 639 | K.ISDDIIR.H |  | 1 | 0.4109 | 22.920 | 0.000 | 416.23 | 1 | 2 | 9/12 | 3776 |
| 640 | R.SPSVYFSGK.V |  | 1 | 0.2104 | 34.820 | 0.000 | 486.25 | 0 | 2 | 5/16 | 3784 |
| 641 | K.QSYGSAEEIHTITR.K |  | 1 | 0.3345 | 33.920 | 0.000 | 531.26 | 0 | 3 | 13/26 | 3785 |
| 642 | K.ELGVHTITVK.A |  | 1 | 0.2088 | 31.870 | 0.000 | 548.82 | 0 | 2 | 8/18 | 3797 |
| 643 | K.ISDLVDESYKR.I |  | 2 | 0.3202 | 40.650 | 0.000 | 662.84 | 0 | 2 | 8/20 | 3807 |
| 644 | R.ELISNSSDAIDK.I |  | 1 | 0.1808 | 39.120 | 0.000 | 646.32 | 0 | 2 | 8/22 | 3808 |
| 645 | K.AVELGLK.V |  | 1 | 0.1721 | 21.940 | 0.018 | 365.23 | 1 | 2 | 6/12 | 3817 |
| 646 | K.SIFHALNQK.A |  | 1 | 0.3963 | 26.690 | 0.000 | 529.29 | 0 | 2 | 11/16 | 3819 |
| 647 | K.GYDIVAGER.R |  | 1 | 0.3962 | 48.360 | 0.000 | 490.25 | 0 | 2 | 7/16 | 3824 |
| 648 | R.EVSLNIK.R |  | 1 | 0.8957 | 26.910 | 0.835 | 401.74 | 1 | 2 | 4/12 | 3835 |
| 649 | K.EIVEEISR.M |  | 1 | 0.9015 | 23.120 | 0.979 | 487.76 | 1 | 2 | 4/14 | 3836 |
| 650 | R.SGGFEPLR.Y |  | 1 | 0.2650 | 22.710 | 0.102 | 431.72 | 1 | 2 | 7/14 | 3841 |
| 651 | K.TALDDAFKK.V |  | 1 | 0.1068 | 28.800 | 0.000 | 336.85 | 1 | 3 | 5/16 | 3842 |
| 652 | R.LATSGVSAHPQHEVYPEIR.R |  | 1 | 0.6815 | 50.370 | 0.000 | 697.69 | 1 | 3 | 19/36 | 3850 |
| 653 | R.AGDDAAGLAISEK.M |  | 1 | 0.2023 | 75.530 | 0.000 | 609.30 | 0 | 2 | 13/24 | 3851 |
| 654 | K.VEEAYNFTK.N |  | 1 | 0.4570 | 31.700 | 0.000 | 550.77 | 0 | 2 | 7/16 | 3858 |
| 655 | K.VAGHVLQIGAVK.A |  | 1 | 0.6619 | 37.050 | 0.686 | 397.91 | 0 | 3 | 13/22 | 3864 |
| 656 | R.WATHGEPSYLNAHPHQSALGR.F |  | 1 | 0.3179 | 44.390 | 0.000 | 583.04 | 0 | 4 | 12/40 | 3866 |
| 657 | R.AAELLGRPLK.D |  | 2 | 0.7550 | 29.930 | 0.973 | 534.33 | 0 | 2 | 6/18 | 3870 |
| 658 | K.AEVYVLSK.E |  | 1 | 0.8604 | 46.450 | 0.426 | 454.76 | 0 | 2 | 11/14 | 3877 |
| 659 | K.TGNLPADEDFK.A |  | 1 | 0.3582 | 38.860 | 0.000 | 603.79 | 0 | 2 | 10/20 | 3878 |
| 660 | R.LAQVSEEIAGTK.A |  | 1 | 0.2723 | 52.570 | 0.000 | 623.34 | 0 | 2 | 8/22 | 3881 |
| 661 | K.IQDAVLNEYHR.L |  | 1 | 0.3268 | 32.640 | 0.000 | 679.34 | 3 | 2 | 9/20 | 3886 |
| 662 | K.DGDLLVGK.V |  | 1 | 0.8797 | 29.220 | 0.894 | 408.73 | 0 | 2 | 5/14 | 3890 |
| 663 | K.NEEAFEYAR.R |  | 1 | 0.5383 | 28.790 | 0.000 | 564.75 | 0 | 2 | 6/16 | 3896 |
| 664 | K.DGTYSVVPR.M |  | 1 | 0.2328 | 20.270 | 0.000 | 497.25 | 0 | 2 | 6/16 | 3901 |
| 665 | R.FIINK.F |  | 1 | 0.7191 | 27.890 | 0.138 | 317.70 | 0 | 2 | 4/8 | 3905 |
| 666 | R.KAIIPAAGLGTR.F |  | 1 | 0.0489 | 26.050 | 0.000 | 389.91 | 0 | 3 | 7/22 | 3907 |
| 667 | R.AAVLFR.A |  | 1 | 0.8881 | 27.360 | 0.781 | 338.71 | 0 | 2 | 5/10 | 3912 |
| 668 | R.RIEEQQAELDEIRGEQK.E |  | 1 | 0.4038 | 44.800 | 0.000 | 691.02 | 1 | 3 | 18/32 | 3913 |
| 669 | K.IELLK.E |  | 1 | 0.3663 | 26.450 | 0.000 | 308.21 | 0 | 2 | 3/8 | 3918 |
| 670 | K.GVTICIK.E |  | 1 | 0.8778 | 22.270 | 0.956 | 395.73 | 0 | 2 | 6/12 | 3927 |
| 671 | R.IFEGTNEINR.L |  | 1 | 0.5270 | 54.250 | 0.000 | 596.80 | 0 | 2 | 7/18 | 3928 |
| 672 | K.TDPFENVK.K |  | 1 | 0.9202 | 33.660 | 0.907 | 475.23 | 0 | 2 | 6/14 | 3934 |
| 673 | K.GGGYYVEYR.T |  | 1 | 0.4674 | 40.730 | 0.000 | 532.25 | 0 | 2 | 7/16 | 3949 |
| 674 | R.GQVLTEGSIDPK.E |  | 1 | 0.8620 | 51.310 | 0.949 | 622.33 | 1 | 2 | 7/22 | 3951 |
| 675 | K.EAFIATEDAR.F |  | 1 | 0.3790 | 63.670 | 0.000 | 561.78 | 1 | 2 | 8/18 | 3954 |
| 676 | R.EFAQNAIDTDGR.S |  | 1 | 0.5469 | 80.620 | 0.000 | 668.81 | 0 | 2 | 10/22 | 3959 |
| 677 | K.SLSTYSDEYQSCEK.- |  | 1 | 0.4432 | 58.680 | 0.000 | 848.85 | 1 | 2 | 8/26 | 3967 |
| 678 | K.DKEDDVVEADIR.G |  | 2 | 0.6133 | 55.480 | 0.000 | 702.34 | 0 | 2 | 14/22 | 3974 |
| 679 | R.SPEVNAFATGPSK.R |  | 1 | 0.3189 | 57.270 | 0.000 | 652.83 | 1 | 2 | 10/24 | 3975 |
| 680 | K.LQLLR.A |  | 1 | 0.7416 | 35.050 | 0.303 | 321.72 | 0 | 2 | 4/8 | 3978 |
| 681 | K.FEEDAFK.I |  | 1 | 0.6455 | 33.530 | 0.355 | 443.20 | 0 | 2 | 5/12 | 3981 |
| 682 | R.YKADEAYLVGEGK.K |  | 1 | 0.2249 | 67.400 | 0.000 | 721.86 | 0 | 2 | 8/24 | 3983 |
| 683 | K.SYEVVGSSKPYGEEVAGK.Q |  | 1 | 0.0829 | 29.620 | 0.000 | 629.31 | 1 | 3 | 12/34 | 3985 |
| 684 | R.AAIDYAIEHGR.K |  | 2 | 0.4777 | 64.400 | 0.000 | 608.31 | 0 | 2 | 7/20 | 3990 |
| 685 | R.EIAEILKR.E |  | 2 | 0.6978 | 25.830 | 0.736 | 486.30 | 0 | 2 | 6/14 | 3991 |
| 686 | K.STPFAAQM#AAETAAK.G |  | 1 | 0.7549 | 73.800 | 0.940 | 755.86 | 1 | 2 | 9/28 | 3996 |
| 687 | K.AVLAIGADAK.N |  | 1 | 0.1595 | 43.380 | 0.000 | 464.78 | 0 | 2 | 7/18 | 4003 |
| 688 | K.LPLVDDQNK.L |  | 1 | 0.9198 | 34.820 | 0.773 | 521.28 | 0 | 2 | 11/16 | 4005 |
| 689 | K.VDYAFNEQR.G |  | 1 | 0.8857 | 35.370 | 0.841 | 571.27 | 0 | 2 | 5/16 | 4006 |
| 690 | K.VTHNLVNEFR.R |  | 1 | 0.6197 | 43.970 | 0.000 | 614.83 | 0 | 2 | 7/18 | 4013 |
| 691 | R.FVGVPDKETIAK.V |  | 1 | 0.0952 | 28.570 | 0.000 | 652.37 | 0 | 2 | 8/22 | 4015 |
| 692 | K.TNAVAVEDLR.E |  | 1 | 0.9283 | 44.980 | 0.939 | 544.29 | 1 | 2 | 8/18 | 4017 |
| 693 | K.ELVVTNSIK.L |  | 1 | 0.2727 | 30.170 | 0.000 | 501.80 | 0 | 2 | 7/16 | 4021 |
| 694 | R.NEVLEELRK.N |  | 1 | 0.8037 | 25.130 | 0.945 | 377.21 | 0 | 3 | 6/16 | 4022 |
| 695 | R.KHEFSALLVK.E |  | 1 | 0.6814 | 32.770 | 0.919 | 391.23 | 0 | 3 | 8/18 | 4029 |
| 696 | R.VASPNYIGVR.D |  | 1 | 0.8575 | 40.120 | 0.956 | 538.30 | 1 | 2 | 8/18 | 4033 |
| 697 | R.LLIEEGCDIKK.E |  | 1 | 0.1204 | 21.110 | 0.000 | 439.91 | 0 | 3 | 9/20 | 4034 |
| 698 | K.LGITPEASDEDGTTR.G |  | 1 | 0.1592 | 71.150 | 0.000 | 781.37 | 0 | 2 | 10/28 | 4038 |
| 699 | R.TPLIISGQAAK.S |  | 1 | 0.8150 | 40.200 | 0.993 | 549.83 | 0 | 2 | 5/20 | 4039 |
| 700 | R.DLTTAEIEQK.V |  | 1 | 0.9444 | 45.560 | 0.877 | 574.30 | 0 | 2 | 13/18 | 4042 |
| 701 | K.TDIPLNATGER.Q |  | 1 | 0.0589 | 20.420 | 0.000 | 593.81 | 1 | 2 | 8/20 | 4046 |
| 702 | R.SVIVVGPHLK.M |  | 2 | 0.3332 | 28.700 | 0.000 | 524.83 | 1 | 2 | 10/18 | 4047 |
| 703 | R.GNYTLGIK.E |  | 1 | 0.8762 | 34.640 | 0.860 | 433.24 | 0 | 2 | 6/14 | 4053 |
| 704 | K.TITVVVETYKK.H |  | 1 | 0.2299 | 53.990 | 0.000 | 640.88 | 0 | 2 | 8/20 | 4055 |
| 705 | K.AALETAEEIEK.L |  | 1 | 0.3003 | 58.540 | 0.000 | 602.31 | 0 | 2 | 9/20 | 4064 |
| 706 | K.VIEFPNSSK.D |  | 1 | 0.6173 | 23.060 | 0.644 | 510.77 | 0 | 2 | 5/16 | 4066 |
| 707 | K.VEANVGAPQVAYR.E |  | 2 | 0.2829 | 57.560 | 0.000 | 687.36 | 0 | 2 | 15/24 | 4075 |
| 708 | K.GEPTLELNDK.G |  | 1 | 0.7692 | 25.870 | 0.920 | 558.28 | 1 | 2 | 9/18 | 4076 |
| 709 | R.RQWAYELSK.C |  | 1 | 0.3172 | 27.470 | 0.000 | 590.81 | 0 | 2 | 7/16 | 4080 |
| 710 | K.LAAYALER.F |  | 1 | 0.9541 | 45.850 | 0.928 | 453.76 | 0 | 2 | 6/14 | 4084 |
| 711 | K.GTAM#AYDQIDGAPEER.E |  | 2 | 0.2762 | 85.930 | 0.000 | 870.38 | 0 | 2 | 10/30 | 4087 |
| 712 | K.VFSSAVANGGVVK.A |  | 1 | 0.1826 | 74.940 | 0.000 | 617.84 | 0 | 2 | 11/24 | 4093 |
| 713 | R.NAENISSQSVEIAR.M |  | 1 | 0.4579 | 70.160 | 0.000 | 759.38 | 1 | 2 | 11/26 | 4095 |
| 714 | R.VVDLIR.H |  | 1 | 0.6796 | 26.560 | 0.531 | 357.73 | 0 | 2 | 5/10 | 4098 |
| 715 | R.AAAENIIPTSTGAAK.A |  | 1 | 0.0515 | 34.390 | 0.000 | 707.88 | 0 | 2 | 10/28 | 4110 |
| 716 | K.ENVGQIDQITK.K |  | 1 | 0.2613 | 38.520 | 0.000 | 622.83 | 0 | 2 | 7/20 | 4131 |
| 717 | R.VLVFAK.G |  | 1 | 0.5114 | 20.410 | 0.290 | 338.72 | 0 | 2 | 6/10 | 4133 |
| 718 | K.IVTLLDKPSGR.K |  | 1 | 0.6680 | 30.590 | 0.889 | 400.24 | 0 | 3 | 6/20 | 4137 |
| 719 | K.YIEDFR.H |  | 1 | 0.6886 | 24.630 | 0.000 | 421.71 | 0 | 2 | 4/10 | 4139 |
| 720 | R.DNAEDNEIIAEQEK.R |  | 1 | 0.5610 | 84.060 | 0.000 | 809.37 | 0 | 2 | 16/26 | 4141 |
| 721 | R.VIGQSGVLDTAR.F |  | 1 | 0.2796 | 67.150 | 0.000 | 608.34 | 1 | 2 | 7/22 | 4144 |
| 722 | R.LSEPVYYGR.S |  | 1 | 0.4084 | 42.190 | 0.000 | 542.28 | 0 | 2 | 7/16 | 4149 |
| 723 | R.VGGLTEALK.I |  | 1 | 0.1000 | 23.060 | 0.000 | 444.26 | 0 | 2 | 7/16 | 4150 |
| 724 | K.VAINGFGR.I |  | 1 | 0.8893 | 40.950 | 0.874 | 417.24 | 0 | 2 | 7/14 | 4156 |
| 725 | R.LKAPIAIIDKR.R |  | 1 | 0.1157 | 32.990 | 0.000 | 310.21 | 1 | 4 | 9/20 | 4161 |
| 726 | R.IDLLK.N |  | 1 | 0.3818 | 26.880 | 0.000 | 301.20 | 0 | 2 | 4/8 | 4163 |
| 727 | K.RADQLIDNIK.T |  | 1 | 0.6196 | 39.730 | 0.587 | 593.33 | 0 | 2 | 5/18 | 4168 |
| 728 | R.EFNREDVVIATK.A |  | 1 | 0.6572 | 28.140 | 0.740 | 710.87 | 1 | 2 | 6/22 | 4171 |
| 729 | K.ILVVDDEAR.I |  | 1 | 0.7946 | 33.480 | 0.825 | 515.28 | 0 | 2 | 4/16 | 4175 |
| 730 | K.NCGYSEFYNK.E |  | 1 | 0.4741 | 45.490 | 0.000 | 641.26 | 1 | 2 | 6/18 | 4184 |
| 731 | K.EVVHFIPR.H |  | 1 | 0.5980 | 39.390 | 0.000 | 498.78 | 0 | 2 | 6/14 | 4190 |
| 732 | K.INQQLQLVSK.Q |  | 1 | 0.4448 | 39.900 | 0.000 | 585.85 | 0 | 2 | 7/18 | 4191 |
| 733 | K.GLITIK.D |  | 1 | 0.5781 | 20.190 | 0.450 | 322.72 | 0 | 2 | 5/10 | 4194 |
| 734 | K.IGSAALTVPEGK.M |  | 1 | 0.1059 | 74.500 | 0.000 | 571.82 | 0 | 2 | 8/22 | 4196 |
| 735 | K.QLLEAGVHFGHQTR.R |  | 1 | 0.3772 | 85.300 | 0.000 | 796.92 | 0 | 2 | 10/26 | 4203 |
| 736 | R.LGELLGK.E |  | 1 | 0.8241 | 42.400 | 0.457 | 365.23 | 0 | 2 | 5/12 | 4207 |
| 737 | R.QIVCIGTSTGGPR.A |  | 1 | 0.2041 | 68.570 | 0.000 | 673.35 | 0 | 2 | 10/24 | 4212 |
| 738 | K.VVFDVASK.I |  | 1 | 0.2023 | 25.380 | 0.000 | 432.74 | 0 | 2 | 7/14 | 4213 |
| 739 | K.VEQSELLCTATDSHR.L |  | 2 | 0.4561 | 90.050 | 0.000 | 873.41 | 1 | 2 | 10/28 | 4214 |
| 740 | R.KDYEIVLR.K |  | 1 | 0.2744 | 32.920 | 0.000 | 518.30 | 0 | 2 | 5/14 | 4216 |
| 741 | K.TTVTGVEM#FR.K |  | 1 | 0.8988 | 40.020 | 0.896 | 578.79 | 0 | 2 | 7/18 | 4219 |
| 742 | R.AEQELYLTNAK.M |  | 1 | 0.7141 | 53.320 | 0.000 | 640.33 | 0 | 2 | 15/20 | 4225 |
| 743 | K.VPAPTGDPEAPLK.A |  | 1 | 0.2173 | 61.310 | 0.000 | 646.35 | 0 | 2 | 8/24 | 4227 |
| 744 | R.ELIIGDR.Q |  | 1 | 0.2918 | 23.970 | 0.000 | 408.23 | 0 | 2 | 5/12 | 4229 |
| 745 | R.DVTYSAPLR.V |  | 1 | 0.3582 | 29.940 | 0.000 | 511.27 | 0 | 2 | 10/16 | 4237 |
| 746 | K.AADLEKEDLLSK.V |  | 2 | 0.2417 | 36.100 | 0.000 | 666.36 | 1 | 2 | 9/22 | 4238 |
| 747 | K.ELEHNELTETVER.M |  | 1 | 0.5835 | 64.060 | 0.000 | 799.89 | 0 | 2 | 10/24 | 4243 |
| 748 | R.DVEFVEDSK.Q |  | 1 | 0.4553 | 38.300 | 0.000 | 534.25 | 0 | 2 | 7/16 | 4248 |
| 749 | R.VVNALIAAQR.A |  | 1 | 0.9390 | 57.370 | 0.996 | 527.82 | 0 | 2 | 9/18 | 4249 |
| 750 | K.SFESIVNK.L |  | 1 | 0.8768 | 44.480 | 0.748 | 462.25 | 0 | 2 | 6/14 | 4251 |
| 751 | K.YYINQAIK.N |  | 1 | 0.4423 | 21.270 | 0.000 | 506.78 | 0 | 2 | 9/14 | 4253 |
| 752 | R.ETYADVIK.N |  | 1 | 0.1708 | 26.130 | 0.000 | 469.75 | 0 | 2 | 6/14 | 4254 |
| 753 | K.VGINGFGR.I |  | 1 | 0.4289 | 40.810 | 0.000 | 410.23 | 0 | 2 | 7/14 | 4259 |
| 754 | K.TGTVTFEVEK.A |  | 1 | 0.2191 | 57.130 | 0.000 | 555.79 | 0 | 2 | 8/18 | 4260 |
| 755 | K.ETEAVILER.V |  | 1 | 0.9559 | 43.280 | 0.887 | 530.29 | 0 | 2 | 7/16 | 4272 |
| 756 | K.GHFFDSETEVR.I |  | 1 | 0.2698 | 26.910 | 0.000 | 662.30 | 0 | 2 | 7/20 | 4273 |
| 757 | K.YASDEWNYK.R |  | 1 | 0.5530 | 32.910 | 0.000 | 588.25 | 0 | 2 | 7/16 | 4274 |
| 758 | R.RIDPSKLELEER.L |  | 1 | 0.6020 | 33.250 | 0.656 | 495.61 | 0 | 3 | 10/22 | 4278 |
| 759 | K.NTEVTDIPDHAVAVK.M |  | 2 | 0.4875 | 61.080 | 0.000 | 804.91 | 0 | 2 | 19/28 | 4294 |
| 760 | R.ISLSIK.D |  | 1 | 0.8098 | 21.990 | 0.721 | 330.72 | 0 | 2 | 5/10 | 4296 |
| 761 | K.APIAIIDK.R |  | 1 | 0.4582 | 42.830 | 0.000 | 420.76 | 0 | 2 | 7/14 | 4300 |
| 762 | R.IGLISDER.Y |  | 1 | 0.3019 | 30.780 | 0.254 | 451.75 | 0 | 2 | 7/14 | 4302 |
| 763 | R.VYTPGVADVCR.L |  | 1 | 0.0793 | 21.980 | 0.000 | 618.81 | 1 | 2 | 5/20 | 4303 |
| 764 | K.LCGDVEFEGAK.E |  | 1 | 0.3239 | 39.480 | 0.000 | 612.78 | 1 | 2 | 8/20 | 4304 |
| 765 | K.FEGCYHGHGDSLLIK.A |  | 1 | 0.4596 | 44.530 | 0.000 | 866.91 | 0 | 2 | 14/28 | 4306 |
| 766 | K.HKPTYTPHVDTGDHVIIINAEK.I |  | 1 | 0.5577 | 34.530 | 0.000 | 622.08 | 0 | 4 | 24/42 | 4308 |
| 767 | R.EPSVVALQTDTK.S |  | 1 | 0.1054 | 31.540 | 0.000 | 644.34 | 1 | 2 | 9/22 | 4320 |
| 768 | R.VPSVEIVR.M |  | 1 | 0.9000 | 30.740 | 0.977 | 449.77 | 0 | 2 | 5/14 | 4327 |
| 769 | R.YDLANVGR.Y |  | 1 | 0.9537 | 37.550 | 0.902 | 454.24 | 0 | 2 | 12/14 | 4329 |
| 770 | K.GAEIHPNDIVIK.E |  | 2 | 0.9051 | 46.460 | 0.926 | 653.36 | 0 | 2 | 14/22 | 4330 |
| 771 | R.ACGESIIPTTTGAAK.A |  | 1 | 0.1796 | 55.900 | 0.000 | 738.87 | 0 | 2 | 12/28 | 4338 |
| 772 | R.VYSGTLDSGSYVK.N |  | 1 | 0.8527 | 76.020 | 0.934 | 688.34 | 0 | 2 | 11/24 | 4340 |
| 773 | K.ATNVTGIALTK.L |  | 1 | 0.5673 | 21.960 | 0.953 | 544.82 | 0 | 2 | 6/20 | 4341 |
| 774 | R.QITVDRPDVIGR.E |  | 2 | 0.1348 | 24.760 | 0.000 | 456.92 | 0 | 3 | 11/22 | 4362 |
| 775 | K.VAVLTNGTLM#R.K |  | 1 | 0.2126 | 44.590 | 0.000 | 595.83 | 0 | 2 | 9/20 | 4367 |
| 776 | K.ITINRPEVHNAFTPK.T |  | 1 | 0.1337 | 24.000 | 0.000 | 579.65 | 0 | 3 | 12/28 | 4368 |
| 777 | R.NDDEWLK.T |  | 1 | 0.4599 | 21.270 | 0.000 | 460.21 | 0 | 2 | 9/12 | 4372 |
| 778 | R.DDRPDLIYR.T |  | 1 | 0.2484 | 21.410 | 0.000 | 388.20 | 0 | 3 | 6/16 | 4376 |
| 779 | K.SVITFESTK.V |  | 1 | 0.7776 | 25.780 | 0.724 | 506.27 | 1 | 2 | 9/16 | 4377 |
| 780 | R.NPQTGEEIEIPASK.V |  | 1 | 0.8200 | 57.860 | 0.727 | 756.88 | 0 | 2 | 9/26 | 4385 |
| 781 | R.DTNLQAFR.N |  | 1 | 0.6020 | 49.100 | 0.000 | 482.75 | 0 | 2 | 6/14 | 4386 |
| 782 | K.FGAGELVR.D |  | 1 | 0.9488 | 53.740 | 0.911 | 424.73 | 0 | 2 | 7/14 | 4393 |
| 783 | K.EPFSASIER.S |  | 1 | 0.3302 | 30.300 | 0.000 | 518.26 | 1 | 2 | 5/16 | 4396 |
| 784 | R.VVVPEEEETDIK.N |  | 1 | 0.1908 | 32.450 | 0.000 | 693.85 | 1 | 2 | 6/22 | 4399 |
| 785 | K.RLETFLQGTK.K |  | 1 | 0.2181 | 38.490 | 0.000 | 596.84 | 0 | 2 | 7/18 | 4400 |
| 786 | K.VATVFPDAR.H |  | 1 | 0.3884 | 41.250 | 0.000 | 488.27 | 0 | 2 | 7/16 | 4404 |
| 787 | R.SKTEELGIEPDFHEK.Y |  | 1 | 0.0991 | 21.070 | 0.000 | 586.96 | 1 | 3 | 9/28 | 4408 |
| 788 | K.HATSFTAVTNPTVNSYK.R |  | 1 | 0.4199 | 48.770 | 0.000 | 613.31 | 0 | 3 | 22/32 | 4415 |
| 789 | K.AIEVTIAGR.M |  | 1 | 0.1682 | 25.990 | 0.000 | 465.27 | 1 | 2 | 6/16 | 4418 |
| 790 | K.VAVIGQSGNLTPADK.K |  | 1 | 0.1788 | 76.450 | 0.000 | 735.40 | 0 | 2 | 11/28 | 4427 |
| 791 | R.VAEGLGLPIKK.D |  | 1 | 0.1281 | 45.180 | 0.000 | 375.57 | 0 | 3 | 14/20 | 4428 |
| 792 | K.GANEVLYPEHAQALR.E |  | 2 | 0.4287 | 46.060 | 0.000 | 834.43 | 1 | 2 | 15/28 | 4433 |
| 793 | K.TASGIVLPDSAK.E |  | 1 | 0.6671 | 46.090 | 0.865 | 579.82 | 0 | 2 | 6/22 | 4437 |
| 794 | K.LSEEELVQQR.Y |  | 1 | 0.9681 | 80.590 | 0.985 | 615.82 | 1 | 2 | 8/18 | 4438 |
| 795 | K.GELNVVYTSR.Y |  | 1 | 0.4698 | 59.330 | 0.000 | 569.30 | 0 | 2 | 7/18 | 4441 |
| 796 | K.NGVGNSILIK.V |  | 1 | 0.7753 | 33.020 | 0.988 | 507.80 | 1 | 2 | 6/18 | 4447 |
| 797 | K.AFENAQLYEQSK.A |  | 1 | 0.3332 | 41.050 | 0.000 | 714.34 | 0 | 2 | 7/22 | 4455 |
| 798 | K.IYAVEPSDSPVLSGGKPGPHK.I |  | 1 | 0.0972 | 33.920 | 0.000 | 534.53 | 0 | 4 | 16/40 | 4459 |
| 799 | R.ATLQGLSELKR.A |  | 2 | 0.8873 | 57.280 | 0.954 | 608.36 | 0 | 2 | 10/20 | 4463 |
| 800 | K.LIQAADYDVEK.A |  | 1 | 0.9701 | 55.240 | 0.924 | 632.82 | 0 | 2 | 16/20 | 4464 |
| 801 | R.IIEVYGPESSGK.T |  | 1 | 0.8265 | 38.500 | 0.960 | 639.83 | 0 | 2 | 7/22 | 4473 |
| 802 | K.LVGIITNR.D |  | 1 | 0.9520 | 38.030 | 0.988 | 443.28 | 0 | 2 | 6/14 | 4478 |
| 803 | R.AEPVGCTATILNK.M |  | 1 | 0.2624 | 83.380 | 0.000 | 687.36 | 0 | 2 | 10/24 | 4480 |
| 804 | R.IPLYVR.R |  | 1 | 0.9413 | 36.630 | 0.761 | 380.74 | 0 | 2 | 5/10 | 4484 |
| 805 | R.DNDLVVNVSK.M |  | 1 | 0.2942 | 51.500 | 0.000 | 551.79 | 0 | 2 | 6/18 | 4494 |
| 806 | R.QIAHNAGLEGSVIVER.L |  | 1 | 0.8558 | 55.720 | 0.997 | 564.97 | 0 | 3 | 10/30 | 4497 |
| 807 | K.AFDQGEWR.T |  | 1 | 0.9283 | 26.140 | 0.850 | 504.73 | 0 | 2 | 5/14 | 4504 |
| 808 | K.SGYAGQHASNLGGIIAR.E |  | 1 | 0.9242 | 103.190 | 0.935 | 836.43 | 0 | 2 | 23/32 | 4507 |
| 809 | K.IKEFGDPYPDQLHESAK.L |  | 1 | 0.3671 | 46.180 | 0.000 | 658.66 | 1 | 3 | 17/32 | 4524 |
| 810 | K.VDSLTEIETK.I |  | 1 | 0.3418 | 55.300 | 0.000 | 567.80 | 0 | 2 | 6/18 | 4527 |
| 811 | K.ILEGTVASVPPQGGR.K |  | 1 | 0.7828 | 74.020 | 0.972 | 740.91 | 1 | 2 | 10/28 | 4528 |
| 812 | R.LSVGATIVR.D |  | 1 | 0.2226 | 32.270 | 0.000 | 458.28 | 0 | 2 | 8/16 | 4529 |
| 813 | K.VGIGPGSICTTR.V |  | 1 | 0.2967 | 67.170 | 0.000 | 609.32 | 0 | 2 | 10/22 | 4533 |
| 814 | K.LIVAGASAYPR.T |  | 1 | 0.4329 | 74.500 | 0.000 | 559.32 | 1 | 2 | 9/20 | 4534 |
| 815 | R.YENDFGQIR.E |  | 1 | 0.9350 | 41.260 | 0.770 | 571.27 | 1 | 2 | 8/16 | 4540 |
| 816 | K.TDFYDYK.A |  | 1 | 0.6622 | 21.410 | 0.000 | 476.21 | 0 | 2 | 6/12 | 4546 |
| 817 | K.NELLVYK.F |  | 1 | 0.9265 | 41.410 | 0.673 | 439.75 | 0 | 2 | 6/12 | 4549 |
| 818 | R.LAYVGITR.A |  | 1 | 0.7832 | 21.510 | 0.909 | 446.77 | 0 | 2 | 6/14 | 4550 |
| 819 | R.ELAQEPEVLK.V |  | 1 | 0.2468 | 28.870 | 0.000 | 578.32 | 1 | 2 | 5/18 | 4557 |
| 820 | R.SAGGIWTEDASR.M |  | 1 | 0.1080 | 43.220 | 0.000 | 625.29 | 1 | 2 | 7/22 | 4561 |
| 821 | R.SYNVVIPGK.S |  | 1 | 0.1994 | 28.590 | 0.000 | 488.78 | 0 | 2 | 5/16 | 4566 |
| 822 | K.VYIADSTNFK.V |  | 1 | 0.2150 | 23.350 | 0.000 | 579.30 | 0 | 2 | 5/18 | 4567 |
| 823 | K.VLLLNSR.D |  | 1 | 0.8633 | 28.730 | 0.720 | 407.76 | 0 | 2 | 4/12 | 4569 |
| 824 | K.GLWSEEEEAK.V |  | 1 | 0.2331 | 37.060 | 0.000 | 589.27 | 0 | 2 | 5/18 | 4573 |
| 825 | K.GEPGTGNIVEAVR.H |  | 1 | 0.0634 | 32.750 | 0.000 | 649.84 | 1 | 2 | 10/24 | 4580 |
| 826 | R.YLVLTEGK.M |  | 1 | 0.2316 | 22.200 | 0.000 | 461.77 | 0 | 2 | 4/14 | 4582 |
| 827 | R.NVNVPGEQEFK.N |  | 1 | 0.2110 | 40.710 | 0.000 | 630.81 | 0 | 2 | 7/20 | 4587 |
| 828 | K.IDEELALK.W |  | 1 | 0.8962 | 33.750 | 0.803 | 465.76 | 0 | 2 | 7/14 | 4591 |
| 829 | K.TLTLLK.G |  | 1 | 0.7799 | 23.090 | 0.611 | 344.73 | 1 | 2 | 5/10 | 4594 |
| 830 | R.TVIAESFER.I |  | 1 | 0.4634 | 42.020 | 0.000 | 526.27 | 1 | 2 | 7/16 | 4599 |
| 831 | K.GPLTTPVGGGIR.S |  | 1 | 0.0345 | 21.640 | 0.000 | 562.82 | 1 | 2 | 6/22 | 4600 |
| 832 | R.VGNDGVITIEESK.G |  | 1 | 0.8903 | 72.280 | 0.970 | 680.85 | 0 | 2 | 11/24 | 4615 |
| 833 | K.LETSFITK.A |  | 1 | 0.8079 | 24.700 | 0.991 | 469.76 | 0 | 2 | 6/14 | 4616 |
| 834 | K.AFEEWR.Y |  | 1 | 0.5516 | 23.350 | 0.000 | 419.20 | 0 | 2 | 5/10 | 4619 |
| 835 | K.STIIVDYR.G |  | 1 | 0.5301 | 35.030 | 0.000 | 483.77 | 0 | 2 | 5/14 | 4627 |
| 836 | K.AAIEALGYEK.G |  | 1 | 0.4629 | 44.420 | 0.000 | 532.78 | 0 | 2 | 14/18 | 4630 |
| 837 | R.AFRPEFINR.I |  | 1 | 0.8788 | 41.720 | 0.861 | 383.88 | 0 | 3 | 6/16 | 4632 |
| 838 | K.NELTEPEEFSK.V |  | 1 | 0.4021 | 37.220 | 0.000 | 661.81 | 1 | 2 | 10/20 | 4639 |
| 839 | R.KHFDDEALAELK.E |  | 1 | 0.8573 | 30.380 | 0.957 | 472.58 | 1 | 3 | 11/22 | 4644 |
| 840 | K.WQEGSSPNVLENK.N |  | 1 | 0.3590 | 60.880 | 0.000 | 744.36 | 0 | 2 | 9/24 | 4648 |
| 841 | K.NLDDGKVDSVSVQPVR.G |  | 1 | 0.8466 | 100.300 | 0.964 | 864.45 | 0 | 2 | 12/30 | 4650 |
| 842 | R.TNTVVAPGSDAGVLR.I |  | 1 | 0.1108 | 50.730 | 0.000 | 728.89 | 0 | 2 | 12/28 | 4651 |
| 843 | R.IGVLTSGGDSPGM#NAAVR.A |  | 1 | 0.0896 | 30.000 | 0.000 | 573.29 | 1 | 3 | 12/34 | 4652 |
| 844 | K.DTPNFIANR.I |  | 1 | 0.9207 | 53.920 | 0.945 | 524.26 | 0 | 2 | 7/16 | 4656 |
| 845 | R.IALAHPEVSIR.L |  | 1 | 0.3149 | 32.350 | 0.000 | 402.57 | 0 | 3 | 6/20 | 4657 |
| 846 | K.VFGELGHLK.A |  | 1 | 0.6885 | 29.760 | 0.628 | 333.86 | 0 | 3 | 7/16 | 4663 |
| 847 | K.SNISLIGPTGSGK.T |  | 1 | 0.0473 | 23.640 | 0.000 | 615.84 | 0 | 2 | 7/24 | 4665 |
| 848 | K.ALEHIHLFK.G |  | 1 | 0.5236 | 34.990 | 0.000 | 369.88 | 0 | 3 | 8/16 | 4667 |
| 849 | K.TVITSPAFTGSEK.G |  | 1 | 0.5867 | 29.970 | 0.799 | 669.35 | 0 | 2 | 9/24 | 4668 |
| 850 | K.DTQIGPLVSK.E |  | 1 | 0.3066 | 29.320 | 0.000 | 529.30 | 0 | 2 | 11/18 | 4673 |
| 851 | R.ITHLPTNVVVTCQTER.S |  | 1 | 0.4129 | 34.740 | 0.000 | 623.33 | 0 | 3 | 14/30 | 4685 |
| 852 | K.ELYELAR.H |  | 1 | 0.3310 | 20.750 | 0.000 | 447.24 | 0 | 2 | 5/12 | 4692 |
| 853 | K.ITFLK.S |  | 1 | 0.3129 | 21.250 | 0.000 | 311.20 | 0 | 2 | 4/8 | 4703 |
| 854 | R.YFQTKPELLDK.I |  | 1 | 0.2903 | 47.010 | 0.000 | 691.37 | 0 | 2 | 7/20 | 4709 |
| 855 | K.SLVILCSHSGNTPETVK.A |  | 2 | 0.5004 | 82.900 | 0.000 | 921.48 | 0 | 2 | 18/32 | 4710 |
| 856 | R.LSNHFVDSLR.G |  | 1 | 0.8385 | 29.280 | 0.832 | 396.54 | 0 | 3 | 11/18 | 4711 |
| 857 | R.HITVSTSGIIPK.I |  | 1 | 0.7989 | 24.650 | 0.956 | 626.87 | 0 | 2 | 12/22 | 4714 |
| 858 | R.TKDTGQDSCLSCVV.- |  | 1 | 0.3477 | 51.750 | 0.000 | 785.35 | 1 | 2 | 12/26 | 4724 |
| 859 | R.VITLGAYNR.N |  | 1 | 0.4359 | 50.510 | 0.000 | 503.79 | 0 | 2 | 7/16 | 4730 |
| 860 | K.QNAIGILQNK.F |  | 1 | 0.2587 | 48.830 | 0.000 | 549.82 | 0 | 2 | 8/18 | 4737 |
| 861 | R.VLNSTGALALK.E |  | 1 | 0.2551 | 64.750 | 0.000 | 543.83 | 0 | 2 | 9/20 | 4740 |
| 862 | K.EVYACCTHPVLSGPAVER.I |  | 2 | 0.3514 | 72.880 | 0.000 | 1022.98 | 0 | 2 | 9/34 | 4743 |
| 863 | R.ELEGALIR.V |  | 1 | 0.7220 | 36.990 | 0.658 | 450.76 | 0 | 2 | 6/14 | 4744 |
| 864 | R.ADIDYATSEADTTYGK.L |  | 2 | 0.2429 | 82.710 | 0.000 | 860.88 | 1 | 2 | 11/30 | 4749 |
| 865 | K.VVTYDFAR.L |  | 1 | 0.5192 | 49.450 | 0.000 | 485.75 | 0 | 2 | 7/14 | 4750 |
| 866 | K.TPPAAVLLK.K |  | 1 | 0.8738 | 44.700 | 0.977 | 455.29 | 1 | 2 | 8/16 | 4752 |
| 867 | R.VDFNVPM#KDGEVTDDTR.I |  | 1 | 0.1768 | 51.690 | 0.000 | 651.97 | 0 | 3 | 8/32 | 4753 |
| 868 | R.NIIQESQTDAYIHK.D |  | 1 | 0.5347 | 85.150 | 0.000 | 830.42 | 0 | 2 | 11/26 | 4754 |
| 869 | R.IGDLSELK.L |  | 1 | 0.7359 | 30.220 | 0.612 | 437.75 | 0 | 2 | 6/14 | 4757 |
| 870 | K.DLNEIDGIGHR.V |  | 2 | 0.8809 | 39.060 | 0.834 | 619.81 | 1 | 2 | 6/20 | 4760 |
| 871 | K.IIAINAGSSSLK.F |  | 1 | 0.1510 | 50.000 | 0.000 | 587.35 | 0 | 2 | 10/22 | 4764 |
| 872 | R.VTDILSQAER.I |  | 1 | 0.8906 | 39.430 | 0.936 | 566.30 | 1 | 2 | 9/18 | 4765 |
| 873 | K.AYLQELGTR.V |  | 1 | 0.4366 | 28.640 | 0.000 | 525.78 | 0 | 2 | 5/16 | 4766 |
| 874 | R.GPDSFLER.K |  | 1 | 0.9714 | 42.250 | 0.956 | 460.73 | 1 | 2 | 6/14 | 4772 |
| 875 | K.ASNLADIHYIR.Q |  | 2 | 0.5805 | 52.510 | 0.000 | 636.84 | 1 | 2 | 8/20 | 4775 |
| 876 | R.LIEVLAK.G |  | 1 | 0.8665 | 24.080 | 0.823 | 393.26 | 0 | 2 | 9/12 | 4778 |
| 877 | R.VIVSQLVR.S |  | 1 | 0.8674 | 50.890 | 0.561 | 457.30 | 0 | 2 | 7/14 | 4784 |
| 878 | R.EITGLGLK.E |  | 1 | 0.7650 | 23.350 | 0.929 | 415.75 | 0 | 2 | 6/14 | 4786 |
| 879 | K.ATVELLER.L |  | 1 | 0.6160 | 59.870 | 0.000 | 465.77 | 0 | 2 | 7/14 | 4787 |
| 880 | R.SSDEVTVIAVTK.Y |  | 1 | 0.8798 | 81.370 | 0.929 | 624.84 | 0 | 2 | 9/22 | 4788 |
| 881 | R.EVIDFR.G |  | 1 | 0.9296 | 27.470 | 0.633 | 389.71 | 1 | 2 | 4/10 | 4789 |
| 882 | K.YVLNAEQIR.H |  | 1 | 0.9467 | 40.100 | 0.893 | 553.30 | 0 | 2 | 7/16 | 4796 |
| 883 | R.APEAITEALK.E |  | 1 | 0.4488 | 55.020 | 0.000 | 521.79 | 0 | 2 | 8/18 | 4800 |
| 884 | R.LGISLSGTGK.E |  | 1 | 0.1586 | 41.140 | 0.000 | 466.77 | 0 | 2 | 7/18 | 4804 |
| 885 | R.GAAEQILEDAKR.D |  | 2 | 0.2198 | 66.020 | 0.000 | 650.85 | 0 | 2 | 8/22 | 4809 |
| 886 | K.WTVIGR.G |  | 1 | 0.9448 | 31.340 | 0.731 | 366.21 | 0 | 2 | 4/10 | 4811 |
| 887 | K.SILVAQEAAEDVKR.N |  | 1 | 0.2665 | 78.870 | 0.000 | 764.92 | 1 | 2 | 11/26 | 4818 |
| 888 | R.TPGNIVAIRPLK.D |  | 1 | 0.1205 | 39.600 | 0.000 | 639.90 | 1 | 2 | 7/22 | 4819 |
| 889 | K.IVVEAANGPTTLEGTK.I |  | 1 | 0.8174 | 71.390 | 0.878 | 800.43 | 1 | 2 | 18/30 | 4820 |
| 890 | R.YEVAGLVTK.Y |  | 1 | 0.8114 | 28.060 | 0.961 | 490.28 | 0 | 2 | 4/16 | 4823 |
| 891 | R.AGINTVQELANK.T |  | 1 | 0.8862 | 61.000 | 0.904 | 629.34 | 0 | 2 | 7/22 | 4826 |
| 892 | K.ASVNLENADETIEK.E |  | 1 | 0.4372 | 72.510 | 0.000 | 766.88 | 0 | 2 | 10/26 | 4830 |
| 893 | K.FVEDVDLSK.V |  | 1 | 0.6874 | 21.910 | 0.770 | 526.27 | 1 | 2 | 7/16 | 4837 |
| 894 | K.EQEWNAQFAK.Y |  | 1 | 0.5193 | 35.290 | 0.000 | 625.79 | 0 | 2 | 7/18 | 4840 |
| 895 | K.EAEENPELVQEAPHTTIVK.R |  | 1 | 0.2908 | 29.650 | 0.000 | 712.03 | 0 | 3 | 13/36 | 4841 |
| 896 | R.IEDELDYPGHIK.V |  | 1 | 0.6044 | 37.600 | 0.000 | 476.91 | 0 | 3 | 11/22 | 4846 |
| 897 | R.LADENSADVYLK.L |  | 1 | 0.3842 | 81.320 | 0.000 | 669.33 | 0 | 2 | 9/22 | 4850 |
| 898 | K.TNYDSFEETIKR.L |  | 1 | 0.2017 | 31.710 | 0.000 | 501.58 | 0 | 3 | 6/22 | 4852 |
| 899 | K.DGLFCER.I |  | 1 | 0.3571 | 24.130 | 0.000 | 448.70 | 0 | 2 | 5/12 | 4855 |
| 900 | K.LEEISESYEGVEK.S |  | 1 | 0.5678 | 78.580 | 0.000 | 756.36 | 0 | 2 | 11/24 | 4857 |
| 901 | K.ENGLFIEK.I |  | 1 | 0.4602 | 24.870 | 0.366 | 475.25 | 0 | 2 | 6/14 | 4858 |
| 902 | K.GEAYFVDSNSVR.V |  | 1 | 0.1649 | 31.430 | 0.000 | 672.31 | 1 | 2 | 9/22 | 4866 |
| 903 | R.EIAEILK.R |  | 1 | 0.9458 | 28.910 | 0.980 | 408.25 | 0 | 2 | 5/12 | 4867 |
| 904 | R.ALDIAIK.D |  | 1 | 0.3057 | 32.940 | 0.000 | 372.24 | 0 | 2 | 9/12 | 4871 |
| 905 | R.QEITAAGYTELK.T |  | 1 | 0.2889 | 57.350 | 0.000 | 662.34 | 0 | 2 | 8/22 | 4873 |
| 906 | R.LDLASYR.E |  | 1 | 0.9210 | 30.380 | 0.782 | 419.23 | 0 | 2 | 6/12 | 4878 |
| 907 | R.VNITLACTECGER.N |  | 1 | 0.3588 | 55.950 | 0.000 | 761.85 | 0 | 2 | 10/24 | 4881 |
| 908 | K.EVSQGYECGITIK.K |  | 1 | 0.4834 | 50.860 | 0.000 | 742.36 | 0 | 2 | 9/24 | 4882 |
| 909 | K.VGNVIEFK.N |  | 1 | 0.9224 | 31.150 | 0.952 | 453.26 | 2 | 2 | 7/14 | 4883 |
| 910 | R.LIEDALTPNTK.C |  | 1 | 0.7679 | 23.910 | 0.929 | 607.84 | 0 | 2 | 9/20 | 4886 |
| 911 | K.LFSDIATR.Y |  | 1 | 0.4788 | 50.240 | 0.000 | 461.75 | 0 | 2 | 7/14 | 4889 |
| 912 | K.CTLCFPR.L |  | 1 | 0.7473 | 34.120 | 0.000 | 477.22 | 0 | 2 | 5/12 | 4891 |
| 913 | K.YDHLPEDAFR.L |  | 1 | 0.6889 | 50.150 | 0.000 | 421.53 | 1 | 3 | 8/18 | 4893 |
| 914 | R.VEICSECHPFYTGR.Q |  | 2 | 0.6988 | 80.460 | 0.000 | 877.89 | 1 | 2 | 11/26 | 4896 |
| 915 | K.VAELVLER.A |  | 1 | 0.5890 | 50.790 | 0.000 | 464.78 | 0 | 2 | 7/14 | 4899 |
| 916 | K.FKAEVYVLSK.E |  | 3 | 0.8920 | 47.620 | 0.929 | 395.23 | 0 | 3 | 8/18 | 4911 |
| 917 | K.TPDNTVTVFAGQDK.E |  | 1 | 0.3586 | 93.550 | 0.000 | 746.87 | 0 | 2 | 10/26 | 4913 |
| 918 | R.EISGDSWGVGR.T |  | 1 | 0.2383 | 45.800 | 0.000 | 581.78 | 1 | 2 | 9/20 | 4921 |
| 919 | K.QAVYFDTPDGR.M |  | 1 | 0.4564 | 54.560 | 0.000 | 634.80 | 1 | 2 | 7/20 | 4925 |
| 920 | K.VGVM#FGNPETTPGGR.A |  | 1 | 0.2002 | 66.580 | 0.000 | 767.87 | 1 | 2 | 11/28 | 4926 |
| 921 | K.LIEEDDTSVLK.A |  | 1 | 0.8640 | 28.670 | 0.993 | 631.33 | 1 | 2 | 9/20 | 4927 |
| 922 | K.CFSLISEETKR.M |  | 1 | 0.2921 | 20.500 | 0.000 | 457.23 | 2 | 3 | 11/20 | 4929 |
| 923 | K.QIGLGSSSGTIDTIHSTSATQSTGR.T |  | 1 | 0.1317 | 61.300 | 0.000 | 821.41 | 2 | 3 | 20/48 | 4936 |
| 924 | R.AIQISNTFTNK.D |  | 1 | 0.5042 | 52.860 | 0.000 | 618.83 | 1 | 2 | 12/20 | 4939 |
| 925 | R.GITISTAHVEYETETR.H |  | 13 | 0.4400 | 85.700 | 0.000 | 903.95 | 1 | 2 | 13/30 | 4941 |
| 926 | R.TGQPLEVIER.D |  | 1 | 0.3061 | 34.120 | 0.000 | 571.31 | 1 | 2 | 7/18 | 4943 |
| 927 | R.ESVAVIVQAAK.Q |  | 1 | 0.9413 | 63.760 | 0.970 | 557.83 | 1 | 2 | 18/20 | 4945 |
| 928 | R.IDPSKLELEER.L |  | 1 | 0.8727 | 47.320 | 0.988 | 664.86 | 1 | 2 | 9/20 | 4947 |
| 929 | R.RPGDIATCFADPAK.A |  | 1 | 0.1738 | 34.970 | 0.000 | 759.87 | 1 | 2 | 19/26 | 4949 |
| 930 | R.GANTCIISLK.G |  | 1 | 0.5861 | 21.740 | 0.833 | 538.79 | 1 | 2 | 10/18 | 4954 |
| 931 | K.YAESFGATGLR.V |  | 1 | 0.3278 | 66.140 | 0.000 | 586.29 | 0 | 2 | 9/20 | 4965 |
| 932 | K.ATSYGIQGIEDR.G |  | 1 | 0.3015 | 54.140 | 0.000 | 655.32 | 0 | 2 | 7/22 | 4967 |
| 933 | K.ELGLLNQQR.L |  | 1 | 0.2301 | 27.610 | 0.000 | 535.80 | 0 | 2 | 7/16 | 4971 |
| 934 | R.VITVVLNAK.G |  | 1 | 0.2021 | 27.750 | 0.000 | 478.81 | 0 | 2 | 7/16 | 4972 |
| 935 | K.TGLVVLAGK.D |  | 1 | 0.8718 | 50.230 | 0.927 | 429.28 | 1 | 2 | 7/16 | 4979 |
| 936 | K.QGNVVHLFER.D |  | 1 | 0.3598 | 50.300 | 0.000 | 599.82 | 0 | 2 | 8/18 | 4980 |
| 937 | K.AAQVSEEASTPQGFNTLK.D |  | 1 | 0.1389 | 74.570 | 0.000 | 939.47 | 0 | 2 | 12/34 | 4981 |
| 938 | K.IHEFNDIIR.K |  | 1 | 0.9488 | 22.880 | 0.971 | 386.21 | 0 | 3 | 7/16 | 4982 |
| 939 | R.TTHSIPDSYGIVVK.T |  | 1 | 0.4389 | 50.450 | 0.000 | 506.27 | 0 | 3 | 14/26 | 4988 |
| 940 | K.EIPEEYKEQAEELR.N |  | 1 | 0.3047 | 74.170 | 0.000 | 881.93 | 0 | 2 | 10/26 | 4992 |
| 941 | R.GVDALADAVK.V |  | 1 | 0.6379 | 24.710 | 0.839 | 479.76 | 0 | 2 | 6/18 | 4999 |
| 942 | R.EIEISGIR.K |  | 1 | 0.7121 | 22.640 | 0.777 | 458.76 | 0 | 2 | 5/14 | 5000 |
| 943 | R.VFAYDFEGKR.Y |  | 1 | 0.1524 | 23.870 | 0.000 | 411.21 | 0 | 3 | 5/18 | 5008 |
| 944 | R.FEELPEETVAK.F |  | 1 | 0.2830 | 39.710 | 0.000 | 646.32 | 0 | 2 | 9/20 | 5012 |
| 945 | R.IGSSLVGNETGGVQQ.- |  | 1 | 0.3755 | 25.620 | 0.551 | 723.37 | 0 | 2 | 10/28 | 5015 |
| 946 | K.LLDNAFR.Y |  | 1 | 0.9612 | 35.580 | 0.926 | 424.74 | 0 | 2 | 6/12 | 5018 |
| 947 | K.VVIPSTPYDAK.G |  | 1 | 0.1299 | 27.170 | 0.000 | 595.33 | 1 | 2 | 4/20 | 5022 |
| 948 | R.LLHAIFGEK.A |  | 1 | 0.9144 | 33.860 | 0.809 | 343.20 | 0 | 3 | 10/16 | 5024 |
| 949 | K.LSVDQEYLK.S |  | 1 | 0.3607 | 31.290 | 0.000 | 547.79 | 1 | 2 | 7/16 | 5030 |
| 950 | K.QFEEETEGGNYITAR.K |  | 1 | 0.4591 | 120.270 | 0.000 | 872.39 | 0 | 2 | 11/28 | 5031 |
| 951 | R.EPNEFEGGHILGAR.N |  | 1 | 0.2972 | 27.910 | 0.000 | 509.25 | 0 | 3 | 15/26 | 5037 |
| 952 | R.HVIISNASCTTNCLAPVVK.V |  | 2 | 0.2935 | 84.200 | 0.000 | 1042.53 | 1 | 2 | 10/36 | 5045 |
| 953 | R.EISDLVTSEEK.S |  | 1 | 0.2973 | 43.090 | 0.000 | 625.31 | 1 | 2 | 8/20 | 5051 |
| 954 | R.EANYDDSFYTENTR.A |  | 1 | 0.4995 | 90.000 | 0.000 | 862.86 | 0 | 2 | 10/26 | 5052 |
| 955 | K.SIIQYTGVSDCK.M |  | 1 | 0.9479 | 70.660 | 0.899 | 685.83 | 0 | 2 | 10/22 | 5059 |
| 956 | R.TPLFIR.F |  | 1 | 0.9587 | 33.360 | 0.781 | 373.73 | 0 | 2 | 4/10 | 5062 |
| 957 | R.IADLLDR.D |  | 1 | 0.7805 | 27.540 | 0.674 | 408.23 | 0 | 2 | 5/12 | 5075 |
| 958 | R.GGGISETFTVR.K |  | 1 | 0.2905 | 54.970 | 0.000 | 562.29 | 0 | 2 | 9/20 | 5083 |
| 959 | K.KQPVQSNTTNFDILK.R |  | 1 | 0.8455 | 39.850 | 0.871 | 578.31 | 0 | 3 | 13/28 | 5100 |
| 960 | K.LGGGVSLNLSK.L |  | 1 | 0.1621 | 45.110 | 0.000 | 522.81 | 0 | 2 | 9/20 | 5103 |
| 961 | R.SNQGTCYNQRPIVSVGDEVVK.G |  | 1 | 0.2253 | 27.500 | 0.000 | 784.05 | 1 | 3 | 17/40 | 5106 |
| 962 | K.M#LELAIK.G |  | 1 | 0.8329 | 26.820 | 0.737 | 417.24 | 0 | 2 | 6/12 | 5117 |
| 963 | K.FEVGSVYTGK.V |  | 1 | 0.4095 | 52.410 | 0.000 | 543.78 | 0 | 2 | 8/18 | 5135 |
| 964 | R.TEIVSLPHDIK.I |  | 1 | 0.4037 | 28.820 | 0.000 | 417.90 | 0 | 3 | 8/20 | 5141 |
| 965 | K.FGDSVTTDHISPAGAIGK.D |  | 1 | 0.6361 | 26.910 | 0.954 | 591.63 | 0 | 3 | 11/34 | 5144 |
| 966 | R.VPFLDK.V |  | 1 | 0.7482 | 20.230 | 0.700 | 359.71 | 0 | 2 | 5/10 | 5148 |
| 967 | K.EDSLDPVIGR.S |  | 1 | 0.1599 | 32.460 | 0.000 | 550.78 | 0 | 2 | 8/18 | 5149 |
| 968 | R.GDYENLSDDALK.H |  | 1 | 0.3547 | 52.930 | 0.000 | 670.30 | 0 | 2 | 8/22 | 5169 |
| 969 | K.GITTETAELLHK.T |  | 1 | 0.2691 | 23.440 | 0.000 | 438.24 | 0 | 3 | 11/22 | 5170 |
| 970 | K.GAQTDLTSLTDESQEK.T |  | 1 | 0.1717 | 94.780 | 0.000 | 861.91 | 0 | 2 | 10/30 | 5173 |
| 971 | K.KADILVVAVGR.A |  | 1 | 0.0944 | 20.740 | 0.000 | 380.91 | 0 | 3 | 9/20 | 5174 |
| 972 | K.EGDVIENLPK.V |  | 1 | 0.1404 | 33.450 | 0.000 | 557.29 | 0 | 2 | 6/18 | 5176 |
| 973 | K.SGSWYSYEEER.L |  | 1 | 0.4967 | 55.390 | 0.000 | 696.79 | 0 | 2 | 9/20 | 5180 |
| 974 | R.YKPPVQLIPR.Q |  | 1 | 0.4980 | 21.900 | 0.607 | 404.25 | 1 | 3 | 6/18 | 5183 |
| 975 | R.LISCHLGNGASIAAVEGGK.S |  | 1 | 0.0527 | 35.750 | 0.000 | 618.66 | 0 | 3 | 8/36 | 5189 |
| 976 | K.DQFFEQNEVK.L |  | 1 | 0.9561 | 53.370 | 0.720 | 642.30 | 0 | 2 | 8/18 | 5190 |
| 977 | K.FDVIYGGAQK.N |  | 1 | 0.6551 | 24.200 | 0.752 | 549.28 | 0 | 2 | 4/18 | 5191 |
| 978 | K.VGNGFIEAEVK.G |  | 1 | 0.2496 | 46.400 | 0.000 | 581.81 | 0 | 2 | 6/20 | 5192 |
| 979 | R.GSVFGLTR.G |  | 1 | 0.2062 | 32.460 | 0.000 | 418.73 | 0 | 2 | 6/14 | 5196 |
| 980 | K.GVYVVISTGR.T |  | 1 | 0.2491 | 41.610 | 0.000 | 525.80 | 0 | 2 | 7/18 | 5204 |
| 981 | K.NLTAAFDAHC.- |  | 1 | 0.6690 | 43.730 | 0.000 | 560.25 | 0 | 2 | 6/18 | 5210 |
| 982 | K.IIAEIFKR.F |  | 1 | 0.3301 | 31.960 | 0.000 | 330.54 | 0 | 3 | 6/14 | 5213 |
| 983 | R.GHIVPVVYLK.E |  | 1 | 0.1312 | 24.950 | 0.000 | 562.85 | 0 | 2 | 4/18 | 5222 |
| 984 | K.IAYAQANLLK.K |  | 1 | 0.8687 | 26.120 | 0.897 | 552.82 | 0 | 2 | 13/18 | 5224 |
| 985 | K.TILHNLLR.C |  | 1 | 0.2215 | 20.150 | 0.000 | 490.31 | 1 | 2 | 6/14 | 5225 |
| 986 | K.LIVADLR.K |  | 1 | 0.6068 | 26.240 | 0.349 | 400.26 | 0 | 2 | 5/12 | 5226 |
| 987 | K.IIVDTYGGYAR.H |  | 1 | 0.4641 | 53.940 | 0.000 | 614.32 | 0 | 2 | 9/20 | 5230 |
| 988 | R.IKEELEYFEK.I |  | 1 | 0.8260 | 29.610 | 0.852 | 443.23 | 1 | 3 | 8/18 | 5232 |
| 989 | K.LAGGKPVYVEGLEENHFK.I |  | 1 | 0.2005 | 52.570 | 0.000 | 663.01 | 0 | 3 | 12/34 | 5233 |
| 990 | K.GTVIFTAHGVSPEVR.R |  | 1 | 0.2339 | 27.930 | 0.000 | 523.95 | 0 | 3 | 14/28 | 5234 |
| 991 | R.IGYAAGSEDIIK.A |  | 1 | 0.6049 | 40.460 | 0.718 | 618.83 | 0 | 2 | 8/22 | 5241 |
| 992 | K.ETQYIDYDDVR.E |  | 1 | 0.4812 | 46.050 | 0.000 | 708.82 | 0 | 2 | 8/20 | 5255 |
| 993 | K.AVGESVAIPLK.Y |  | 1 | 0.1862 | 24.240 | 0.000 | 542.32 | 1 | 2 | 5/20 | 5258 |
| 994 | R.REGYELQVSKPEVIIK.E |  | 1 | 0.1600 | 39.960 | 0.000 | 630.02 | 0 | 3 | 13/30 | 5260 |
| 995 | K.RQEQVLLSGGVIQQETR.R |  | 1 | 0.2084 | 59.760 | 0.000 | 647.69 | 0 | 3 | 10/32 | 5263 |
| 996 | K.ATVADHPHVLEGIGSLK.T |  | 1 | 0.1781 | 25.190 | 0.000 | 436.74 | 0 | 4 | 13/32 | 5264 |
| 997 | K.IVGLLGGR.V |  | 1 | 0.1457 | 26.120 | 0.000 | 392.76 | 0 | 2 | 7/14 | 5266 |
| 998 | K.GTNLLGIR.T |  | 1 | 0.8928 | 38.440 | 0.923 | 422.26 | 0 | 2 | 6/14 | 5277 |
| 999 | R.M#VFAIPR.E |  | 1 | 0.6273 | 27.340 | 0.000 | 425.24 | 1 | 2 | 6/12 | 5279 |
| 1000 | R.FPETSGIGIKPVSEEGTSR.L |  | 1 | 0.1396 | 34.990 | 0.000 | 664.34 | 1 | 3 | 16/36 | 5287 |
| 1001 | K.TGQTADEVLNTLNKK.S |  | 2 | 0.7148 | 86.040 | 0.792 | 816.43 | 1 | 2 | 10/28 | 5291 |
| 1002 | K.LATDSLTHVSETTDIR.N |  | 2 | 0.3768 | 77.670 | 0.000 | 879.95 | 1 | 2 | 12/30 | 5292 |
| 1003 | R.GEEAFISYPHSPVK.V |  | 2 | 0.9120 | 46.920 | 0.952 | 780.89 | 1 | 2 | 10/26 | 5293 |
| 1004 | K.TYFTESSSYSDINK.M |  | 1 | 0.5052 | 85.000 | 0.000 | 821.37 | 0 | 2 | 11/26 | 5300 |
| 1005 | K.AFITPIER.E |  | 1 | 0.3012 | 20.530 | 0.000 | 473.77 | 0 | 2 | 6/14 | 5308 |
| 1006 | K.AYAEELLGK.T |  | 1 | 0.9371 | 49.140 | 0.760 | 497.27 | 0 | 2 | 13/16 | 5310 |
| 1007 | K.FSQYVSINQGVAR.I |  | 1 | 0.3207 | 63.370 | 0.000 | 734.88 | 0 | 2 | 8/24 | 5311 |
| 1008 | K.AIIPAAGLGTR.F |  | 1 | 0.1849 | 45.230 | 0.000 | 520.32 | 0 | 2 | 14/20 | 5318 |
| 1009 | K.ILSDLNPNIK.V |  | 1 | 0.3473 | 26.700 | 0.000 | 563.83 | 0 | 2 | 11/18 | 5319 |
| 1010 | K.FGPYELGK.T |  | 1 | 0.8828 | 30.970 | 0.960 | 455.74 | 0 | 2 | 7/14 | 5327 |
| 1011 | K.RVHINILEIK.R |  | 1 | 0.3272 | 45.120 | 0.000 | 412.26 | 0 | 3 | 10/18 | 5334 |
| 1012 | K.GSVSAEVISLK.A |  | 1 | 0.8051 | 55.150 | 0.772 | 545.31 | 0 | 2 | 7/20 | 5340 |
| 1013 | R.QWAYELSK.C |  | 1 | 0.4452 | 27.110 | 0.000 | 512.76 | 0 | 2 | 6/14 | 5341 |
| 1014 | R.GDNEFGEGQFVAGK.I |  | 1 | 0.1587 | 51.390 | 0.000 | 727.83 | 0 | 2 | 10/26 | 5342 |
| 1015 | K.HGYFVPQQFNNPSNPEIHR.Q |  | 1 | 0.5595 | 43.270 | 0.000 | 761.03 | 0 | 3 | 16/36 | 5343 |
| 1016 | R.NIEGGEQSQLNYLK.W |  | 1 | 0.3649 | 85.240 | 0.000 | 796.90 | 0 | 2 | 10/26 | 5345 |
| 1017 | R.SEYYSEGTVPLHTLR.A |  | 2 | 0.5093 | 85.620 | 0.000 | 876.43 | 0 | 2 | 11/28 | 5349 |
| 1018 | R.QLDEDYFK.K |  | 1 | 0.2781 | 21.080 | 0.000 | 529.25 | 0 | 2 | 5/14 | 5356 |
| 1019 | R.STHGVNCTGSCSWNIYVK.N |  | 1 | 0.2711 | 46.370 | 0.000 | 690.64 | 1 | 3 | 10/34 | 5358 |
| 1020 | K.TLLAQTLAR.I |  | 1 | 0.3968 | 36.830 | 0.000 | 493.80 | 0 | 2 | 8/16 | 5362 |
| 1021 | R.QLLQDLGR.E |  | 1 | 0.8140 | 20.340 | 0.864 | 471.77 | 0 | 2 | 4/14 | 5363 |
| 1022 | K.GADIPVDITDQK.V |  | 1 | 0.8440 | 56.850 | 0.690 | 636.33 | 0 | 2 | 15/22 | 5365 |
| 1023 | K.DTYSVIGGGDSAAAVEK.F |  | 1 | 0.0999 | 104.860 | 0.000 | 820.39 | 0 | 2 | 13/32 | 5367 |
| 1024 | K.EAAEIAHYLDEK.M |  | 1 | 0.2915 | 21.450 | 0.000 | 463.56 | 0 | 3 | 7/22 | 5368 |
| 1025 | K.HVLVVYDDLSK.Q |  | 1 | 0.6413 | 51.780 | 0.000 | 644.35 | 0 | 2 | 18/20 | 5373 |
| 1026 | K.QEETETDLNVLAK.T |  | 1 | 0.4770 | 79.880 | 0.000 | 745.37 | 0 | 2 | 11/24 | 5374 |
| 1027 | R.LEDDVDPNETTGQHLKPAEFYEK.M |  | 1 | 0.2884 | 31.810 | 0.000 | 669.57 | 0 | 4 | 13/44 | 5376 |
| 1028 | K.ILQLIK.Q |  | 1 | 0.6028 | 36.500 | 0.333 | 364.26 | 0 | 2 | 5/10 | 5378 |
| 1029 | R.ALTDLGIK.T |  | 1 | 0.7547 | 38.540 | 0.742 | 415.75 | 1 | 2 | 7/14 | 5384 |
| 1030 | R.FALIDESK.V |  | 1 | 0.3238 | 34.890 | 0.000 | 461.75 | 1 | 2 | 4/14 | 5386 |
| 1031 | R.LAVSAGADFVK.T |  | 1 | 0.6420 | 37.690 | 0.791 | 539.30 | 1 | 2 | 7/20 | 5388 |
| 1032 | K.SYAESYLGETVSK.A |  | 1 | 0.3574 | 73.800 | 0.000 | 717.34 | 0 | 2 | 9/24 | 5396 |
| 1033 | K.TYTFTIR.D |  | 1 | 0.3948 | 24.310 | 0.000 | 451.24 | 0 | 2 | 6/12 | 5399 |
| 1034 | R.NGLGLIAEDR.I |  | 1 | 0.3313 | 39.590 | 0.000 | 529.29 | 0 | 2 | 7/18 | 5404 |
| 1035 | R.LLIEEGCDIK.K |  | 1 | 0.2321 | 25.230 | 0.000 | 595.31 | 1 | 2 | 7/18 | 5408 |
| 1036 | R.QILTQQALQEGKPENIVAK.M |  | 1 | 0.0798 | 26.770 | 0.000 | 703.39 | 0 | 3 | 9/36 | 5409 |
| 1037 | K.TDVTAEEVNEAFKR.A |  | 2 | 0.2658 | 87.370 | 0.000 | 804.90 | 0 | 2 | 11/26 | 5411 |
| 1038 | K.LEITSTPNQDSPLSEGK.T |  | 1 | 0.2236 | 65.270 | 0.000 | 908.45 | 0 | 2 | 11/32 | 5412 |
| 1039 | R.LIEIGSYR.G |  | 1 | 0.9282 | 32.000 | 0.949 | 475.77 | 0 | 2 | 6/14 | 5417 |
| 1040 | K.TAYLVITSDR.G |  | 1 | 0.4219 | 51.400 | 0.000 | 569.81 | 1 | 2 | 6/18 | 5422 |
| 1041 | R.SILGTISSAK.N |  | 1 | 0.0721 | 28.500 | 0.000 | 488.79 | 0 | 2 | 6/18 | 5423 |
| 1042 | R.LKDFLE.- |  | 1 | 0.8437 | 22.610 | 0.664 | 382.71 | 0 | 2 | 4/10 | 5424 |
| 1043 | R.SAWVFK.N |  | 1 | 0.9579 | 30.270 | 0.969 | 369.20 | 0 | 2 | 5/10 | 5426 |
| 1044 | R.TLSIDAIEK.A |  | 1 | 0.2007 | 21.410 | 0.000 | 495.28 | 0 | 2 | 7/16 | 5428 |
| 1045 | K.VCTHIADLDFNK.I |  | 1 | 0.3901 | 20.920 | 0.000 | 478.23 | 0 | 3 | 9/22 | 5433 |
| 1046 | R.IDFIK.D |  | 1 | 0.5136 | 33.160 | 0.000 | 318.19 | 1 | 2 | 5/8 | 5450 |
| 1047 | R.FSTVAGELGSADTVRDPR.G |  | 1 | 0.0720 | 36.360 | 0.000 | 626.65 | 1 | 3 | 10/34 | 5455 |
| 1048 | K.VIDFINNEK.E |  | 1 | 0.8950 | 37.340 | 0.787 | 546.29 | 1 | 2 | 5/16 | 5456 |
| 1049 | K.APAIFVVQNNR.Y |  | 1 | 0.4227 | 86.770 | 0.000 | 614.84 | 0 | 2 | 8/20 | 5457 |
| 1050 | K.AVEEAGIEPVDRPEIDVEK.I |  | 1 | 0.0412 | 25.010 | 0.000 | 699.02 | 1 | 3 | 7/36 | 5459 |
| 1051 | K.TITVVVETYK.K |  | 1 | 0.2114 | 24.010 | 0.000 | 576.83 | 0 | 2 | 5/18 | 5460 |
| 1052 | K.IALPLHPEYR.T |  | 1 | 0.4104 | 30.390 | 0.000 | 403.56 | 0 | 3 | 11/18 | 5461 |
| 1053 | R.AGLTTIIAPK.D |  | 1 | 0.9109 | 75.110 | 0.904 | 492.81 | 0 | 2 | 8/18 | 5462 |
| 1054 | R.YATDQIVEYAK.E |  | 1 | 0.5012 | 56.490 | 0.000 | 650.82 | 0 | 2 | 7/20 | 5463 |
| 1055 | K.MAEHIVDLVR.D |  | 2 | 0.4563 | 41.130 | 0.000 | 394.88 | 0 | 3 | 6/18 | 5469 |
| 1056 | K.VIQNIQQIVR.E |  | 1 | 0.5770 | 48.660 | 0.000 | 605.87 | 0 | 2 | 9/18 | 5478 |
| 1057 | K.YYHPDEFAELK.E |  | 1 | 0.5345 | 34.290 | 0.000 | 471.22 | 0 | 3 | 8/20 | 5482 |
| 1058 | R.GYLTSCPTNVGTGLR.A |  | 1 | 0.2667 | 80.140 | 0.000 | 798.40 | 0 | 2 | 12/28 | 5483 |
| 1059 | K.EAVEITEIPAANEK.Q |  | 1 | 0.3797 | 71.050 | 0.000 | 757.39 | 0 | 2 | 17/26 | 5484 |
| 1060 | K.LLSDHGIFVR.V |  | 2 | 0.3128 | 49.070 | 0.000 | 578.83 | 0 | 2 | 8/18 | 5491 |
| 1061 | R.ILLLR.D |  | 1 | 0.6525 | 34.580 | 0.000 | 314.23 | 0 | 2 | 4/8 | 5494 |
| 1062 | K.DVEDLGGTLR.L |  | 1 | 0.3170 | 53.240 | 0.000 | 537.77 | 0 | 2 | 8/18 | 5499 |
| 1063 | R.ATLQGLSELK.R |  | 1 | 0.8848 | 57.660 | 0.900 | 530.31 | 0 | 2 | 8/18 | 5500 |
| 1064 | K.TLPAAGTYTFR.A |  | 1 | 0.1776 | 30.950 | 0.000 | 599.32 | 0 | 2 | 12/20 | 5502 |
| 1065 | R.EAIFHALVR.K |  | 1 | 0.2472 | 23.440 | 0.000 | 352.54 | 0 | 3 | 6/16 | 5506 |
| 1066 | R.SETLENYIR.R |  | 1 | 0.4492 | 32.350 | 0.000 | 562.78 | 0 | 2 | 5/16 | 5514 |
| 1067 | K.GDNVPEPGVPESFK.V |  | 1 | 0.0661 | 22.250 | 0.000 | 736.36 | 1 | 2 | 7/26 | 5516 |
| 1068 | M.PTINQLIR.K |  | 1 | 0.8077 | 61.090 | 0.000 | 477.79 | 0 | 2 | 7/14 | 5521 |
| 1069 | R.IDLIGVK.K |  | 1 | 0.8144 | 47.390 | 0.519 | 379.24 | 1 | 2 | 6/12 | 5530 |
| 1070 | K.QTTDLIFK.R |  | 1 | 0.3865 | 29.860 | 0.000 | 483.27 | 1 | 2 | 5/14 | 5531 |
| 1071 | K.SFFGVR.S |  | 1 | 0.4066 | 25.250 | 0.000 | 356.69 | 1 | 2 | 4/10 | 5532 |
| 1072 | K.DINLIVK.A |  | 1 | 0.9471 | 37.450 | 0.760 | 407.76 | 0 | 2 | 5/12 | 5542 |
| 1073 | K.LSEYGLQLQEK.Q |  | 2 | 0.5548 | 71.080 | 0.000 | 654.35 | 1 | 2 | 8/20 | 5550 |
| 1074 | R.WTSPVTIR.S |  | 1 | 0.9466 | 37.810 | 0.845 | 480.27 | 1 | 2 | 6/14 | 5555 |
| 1075 | R.YFNPFGAHPSGR.I |  | 2 | 0.3931 | 40.210 | 0.000 | 450.55 | 1 | 3 | 14/22 | 5556 |
| 1076 | R.NPNTVSEVQELSESR.F |  | 1 | 0.3467 | 83.830 | 0.000 | 844.91 | 3 | 2 | 10/28 | 5561 |
| 1077 | K.LRPFLLR.D |  | 1 | 0.3175 | 27.390 | 0.000 | 305.54 | 1 | 3 | 5/12 | 5566 |
| 1078 | K.AEHVSVEDVNTIIK.D |  | 2 | 0.5419 | 69.540 | 0.000 | 777.41 | 1 | 2 | 19/26 | 5573 |
| 1079 | K.SPNILLPDANLKK.A |  | 2 | 0.0631 | 34.720 | 0.000 | 711.92 | 1 | 2 | 5/24 | 5574 |
| 1080 | K.EITGDQSVNGITYVDR.E |  | 1 | 0.3837 | 92.170 | 0.000 | 883.93 | 1 | 2 | 13/30 | 5577 |
| 1081 | R.DWENVYR.N |  | 1 | 0.7973 | 35.410 | 0.000 | 491.22 | 1 | 2 | 6/12 | 5584 |
| 1082 | R.DPEHIDVVEAYCR.S |  | 1 | 0.5278 | 53.660 | 0.000 | 801.86 | 1 | 2 | 8/24 | 5587 |
| 1083 | R.LNALVNEISAK.K |  | 1 | 0.2783 | 48.100 | 0.000 | 586.34 | 0 | 2 | 7/20 | 5593 |
| 1084 | K.VIGGGLPVGAYGGK.A |  | 1 | 0.1075 | 55.100 | 0.000 | 622.85 | 1 | 2 | 10/26 | 5594 |
| 1085 | K.NIFYVSER.Y |  | 1 | 0.4667 | 20.090 | 0.000 | 514.26 | 0 | 2 | 6/14 | 5595 |
| 1086 | K.LFSIVPVKR.S |  | 1 | 0.7675 | 25.880 | 0.852 | 353.56 | 0 | 3 | 10/16 | 5604 |
| 1087 | K.NNPDILAGYTEK.S |  | 1 | 0.9054 | 46.450 | 0.990 | 667.83 | 0 | 2 | 6/22 | 5612 |
| 1088 | K.TNVTLYIPQK.E |  | 1 | 0.4743 | 35.360 | 0.000 | 588.83 | 0 | 2 | 5/18 | 5614 |
| 1089 | R.LIGPNCPGVITPEECK.I |  | 1 | 0.4076 | 69.950 | 0.000 | 892.44 | 0 | 2 | 12/30 | 5615 |
| 1090 | K.LIQDYLK.E |  | 1 | 0.8775 | 27.090 | 0.750 | 446.76 | 0 | 2 | 6/12 | 5617 |
| 1091 | K.DISIGLR.T |  | 1 | 0.9180 | 33.240 | 0.806 | 387.23 | 0 | 2 | 6/12 | 5618 |
| 1092 | K.TNYDSFEETIK.R |  | 1 | 0.6538 | 56.490 | 0.000 | 673.81 | 0 | 2 | 8/20 | 5629 |
| 1093 | K.ILDLVQNASSR.K |  | 1 | 0.4647 | 60.230 | 0.000 | 608.34 | 0 | 2 | 9/20 | 5632 |
| 1094 | K.YDIHIHVPEGAVPK.D |  | 1 | 0.4756 | 30.400 | 0.000 | 525.62 | 0 | 3 | 10/26 | 5638 |
| 1095 | K.GSGSDIPASEDGLQYVK.T |  | 1 | 0.0946 | 51.950 | 0.000 | 861.91 | 0 | 2 | 12/32 | 5641 |
| 1096 | K.VVANGAGAAGIAIIK.L |  | 1 | 0.0744 | 87.170 | 0.000 | 662.90 | 0 | 2 | 9/28 | 5647 |
| 1097 | R.SANIALINYADGEKR.Y |  | 2 | 0.1534 | 58.780 | 0.000 | 817.93 | 0 | 2 | 9/28 | 5650 |
| 1098 | R.IGLAM#IEAAEK.E |  | 1 | 0.1252 | 33.410 | 0.000 | 581.31 | 0 | 2 | 9/20 | 5654 |
| 1099 | K.ELPGVAALNDK.- |  | 1 | 0.8544 | 40.970 | 0.981 | 563.81 | 0 | 2 | 7/20 | 5665 |
| 1100 | K.GAVPEHLTSLIAK.M |  | 1 | 0.5747 | 40.150 | 0.711 | 668.39 | 0 | 2 | 10/24 | 5667 |
| 1101 | R.FFSEIVK.K |  | 1 | 0.2499 | 27.110 | 0.000 | 435.24 | 0 | 2 | 5/12 | 5668 |
| 1102 | K.ETAAALGIENISK.V |  | 1 | 0.5832 | 60.190 | 0.812 | 658.86 | 0 | 2 | 9/24 | 5676 |
| 1103 | K.ILTGLLR.K |  | 1 | 0.8301 | 45.880 | 0.665 | 393.27 | 0 | 2 | 6/12 | 5683 |
| 1104 | K.GTAPSNLAILGR.Y |  | 1 | 0.1910 | 48.670 | 0.000 | 585.34 | 0 | 2 | 10/22 | 5691 |
| 1105 | M.SNITIYDVAR.E |  | 1 | 0.1304 | 25.830 | 0.000 | 576.31 | 0 | 2 | 8/18 | 5695 |
| 1106 | R.TPEEAYAYLEK.L |  | 1 | 0.6451 | 72.410 | 0.000 | 657.32 | 0 | 2 | 9/20 | 5697 |
| 1107 | K.LVGYNTDGEGFVK.S |  | 1 | 0.3265 | 62.530 | 0.000 | 699.85 | 0 | 2 | 9/24 | 5698 |
| 1108 | R.VSTEQEAQEFIQK.I |  | 1 | 0.4246 | 60.730 | 0.000 | 768.88 | 0 | 2 | 9/24 | 5702 |
| 1109 | K.AHFLGTHFFNPAR.Y |  | 1 | 0.2748 | 25.830 | 0.000 | 505.59 | 0 | 3 | 6/24 | 5703 |
| 1110 | R.AYGDDEAIVGGIAK.F |  | 1 | 0.4476 | 80.420 | 0.000 | 689.85 | 0 | 2 | 19/26 | 5705 |
| 1111 | K.VIDIDQAPIGR.T |  | 1 | 0.4401 | 51.070 | 0.000 | 598.83 | 1 | 2 | 15/20 | 5706 |
| 1112 | K.HLPAQDEQVFNAIK.N |  | 2 | 0.7540 | 64.190 | 0.000 | 805.42 | 1 | 2 | 20/26 | 5710 |
| 1113 | R.FQLATGQLENTAR.I |  | 1 | 0.4004 | 61.210 | 0.000 | 724.88 | 1 | 2 | 9/24 | 5716 |
| 1114 | K.KGDSVVTIGGLHGTVDSIDESK.V |  | 3 | 0.0611 | 84.620 | 0.000 | 1107.57 | 0 | 2 | 17/42 | 5721 |
| 1115 | R.ALAPEIVGEEHYAVAR.E |  | 1 | 0.3395 | 76.140 | 0.000 | 862.95 | 0 | 2 | 12/30 | 5731 |
| 1116 | R.DQTIVINGLSK.S |  | 1 | 0.8186 | 34.720 | 0.888 | 594.34 | 0 | 2 | 8/20 | 5732 |
| 1117 | R.SFTFITK.T |  | 1 | 0.6092 | 48.130 | 0.000 | 422.23 | 0 | 2 | 6/12 | 5734 |
| 1118 | K.ILSELGIK.V |  | 1 | 0.5450 | 32.250 | 0.514 | 436.78 | 0 | 2 | 6/14 | 5736 |
| 1119 | K.QLVELYPESK.V |  | 1 | 0.9606 | 55.220 | 0.977 | 603.32 | 0 | 2 | 7/18 | 5738 |
| 1120 | K.ISTAGTTIPVSHISSNWK.E |  | 1 | 0.0864 | 23.440 | 0.000 | 633.67 | 0 | 3 | 7/34 | 5740 |
| 1121 | R.LDQDTSGAIVFAK.H |  | 1 | 0.0297 | 22.740 | 0.000 | 682.86 | 0 | 2 | 10/24 | 5750 |
| 1122 | K.NLWTENDEGIK.I |  | 1 | 0.2846 | 35.370 | 0.000 | 659.82 | 1 | 2 | 6/20 | 5754 |
| 1123 | K.YTEDTTFPEGDLR.N |  | 1 | 0.4593 | 39.000 | 0.000 | 772.35 | 1 | 2 | 16/24 | 5762 |
| 1124 | K.FPDFIHTQK.R |  | 1 | 0.9129 | 27.100 | 0.668 | 378.20 | 0 | 3 | 14/16 | 5763 |
| 1125 | K.IQAPVLLIHGEK.D |  | 1 | 0.8604 | 35.150 | 0.988 | 439.93 | 0 | 3 | 9/22 | 5764 |
| 1126 | K.NVGILLDTK.G |  | 1 | 0.5853 | 33.900 | 0.000 | 486.79 | 0 | 2 | 12/16 | 5769 |
| 1127 | K.DAVIDFM#SER.F |  | 1 | 0.3661 | 29.650 | 0.000 | 599.77 | 0 | 2 | 8/18 | 5772 |
| 1128 | R.EEDGIVLVDQNACR.S |  | 1 | 0.3202 | 67.360 | 0.000 | 809.38 | 0 | 2 | 11/26 | 5776 |
| 1129 | R.LLGACGSCPSSTITLK.A |  | 1 | 0.4378 | 77.880 | 0.000 | 832.92 | 0 | 2 | 18/30 | 5786 |
| 1130 | R.LVSNPDDDISFTR.I |  | 1 | 0.3299 | 63.350 | 0.000 | 739.86 | 1 | 2 | 10/24 | 5790 |
| 1131 | K.LPQAAYQIDVPAK.E |  | 1 | 0.3420 | 69.980 | 0.000 | 707.39 | 0 | 2 | 11/24 | 5796 |
| 1132 | K.SLLEEDKIELAK.S |  | 1 | 0.9179 | 74.570 | 0.903 | 694.39 | 0 | 2 | 10/22 | 5804 |
| 1133 | K.VGDEVEIIGLQEENKK.T |  | 2 | 0.8550 | 81.240 | 0.750 | 900.47 | 0 | 2 | 22/30 | 5807 |
| 1134 | K.LFDGSYHDVDSNEM#AFK.V |  | 1 | 0.4291 | 35.460 | 0.000 | 664.29 | 0 | 3 | 14/32 | 5811 |
| 1135 | K.IRFPETSGIGIKPVSEEGTSR.L |  | 2 | 0.6606 | 52.140 | 0.992 | 565.80 | 0 | 4 | 15/40 | 5814 |
| 1136 | K.NTQPYSSPCAGSIFR.N |  | 1 | 0.2810 | 64.410 | 0.000 | 842.89 | 0 | 2 | 9/28 | 5817 |
| 1137 | R.DQNIEYKPSQIIVCTGAK.H |  | 2 | 0.5846 | 73.180 | 0.000 | 1032.53 | 2 | 2 | 25/34 | 5819 |
| 1138 | K.INIAIEEYK.A |  | 1 | 0.3753 | 27.550 | 0.000 | 546.80 | 1 | 2 | 5/16 | 5820 |
| 1139 | R.VIPGFVSQGGCPHGTGTGGPGYTIK.C |  | 1 | 0.0397 | 27.220 | 0.000 | 815.41 | 0 | 3 | 11/48 | 5824 |
| 1140 | K.YNLGFVR.R |  | 1 | 0.8685 | 29.710 | 0.773 | 434.74 | 0 | 2 | 5/12 | 5829 |
| 1141 | R.SIAWGIAR.S |  | 1 | 0.8948 | 38.040 | 0.885 | 437.25 | 0 | 2 | 6/14 | 5830 |
| 1142 | R.TVGSGVVSTITE.- |  | 1 | 0.8164 | 46.580 | 0.900 | 575.30 | 1 | 2 | 7/22 | 5833 |
| 1143 | R.LEVEGTLITGSK.T |  | 1 | 0.1685 | 51.610 | 0.000 | 623.85 | 0 | 2 | 8/22 | 5836 |
| 1144 | R.TYDYTIGIR.A |  | 1 | 0.6088 | 53.020 | 0.000 | 551.28 | 0 | 2 | 6/16 | 5844 |
| 1145 | R.ISLSADPVEEVK.V |  | 1 | 0.7972 | 34.850 | 0.840 | 643.85 | 0 | 2 | 9/22 | 5846 |
| 1146 | R.KTANIGDVIVCTVK.Q |  | 1 | 0.2877 | 61.140 | 0.000 | 759.42 | 0 | 2 | 12/26 | 5853 |
| 1147 | K.AEVEESIFQTAK.S |  | 1 | 0.9322 | 59.120 | 0.925 | 676.34 | 0 | 2 | 7/22 | 5855 |
| 1148 | R.KLPVTVLLR.A |  | 2 | 0.1078 | 45.870 | 0.000 | 519.86 | 0 | 2 | 5/16 | 5858 |
| 1149 | R.VGESIHDIFR.K |  | 1 | 0.5432 | 31.120 | 0.000 | 391.54 | 0 | 3 | 11/18 | 5860 |
| 1150 | K.QEYLQDVELK.S |  | 1 | 0.8798 | 30.290 | 0.747 | 632.82 | 0 | 2 | 7/18 | 5871 |
| 1151 | K.EFADTLKEYETK.G |  | 1 | 0.7498 | 21.500 | 0.630 | 491.91 | 0 | 3 | 12/22 | 5872 |
| 1152 | K.AEGYEVLYDDR.A |  | 1 | 0.5284 | 58.710 | 0.000 | 665.30 | 0 | 2 | 7/20 | 5876 |
| 1153 | K.VEISLLK.D |  | 1 | 0.4915 | 20.160 | 0.419 | 401.26 | 0 | 2 | 6/12 | 5880 |
| 1154 | K.GLVAIDATCPDVTK.T |  | 1 | 0.3841 | 52.400 | 0.000 | 730.38 | 0 | 2 | 13/26 | 5894 |
| 1155 | R.NDYEYDLGHFR.G |  | 1 | 0.4471 | 29.100 | 0.000 | 476.88 | 0 | 3 | 8/20 | 5897 |
| 1156 | K.LPLYGQELTR.D |  | 1 | 0.8271 | 40.830 | 0.844 | 595.33 | 0 | 2 | 5/18 | 5902 |
| 1157 | K.QFDDTLLEK.I |  | 1 | 0.8623 | 55.440 | 0.557 | 554.78 | 0 | 2 | 7/16 | 5906 |
| 1158 | K.LTGGVAGLLK.G |  | 1 | 0.7674 | 41.060 | 0.686 | 464.79 | 0 | 2 | 8/18 | 5915 |
| 1159 | R.FSTVAGELGSADTVR.D |  | 1 | 0.2010 | 79.090 | 0.000 | 755.38 | 1 | 2 | 12/28 | 5916 |
| 1160 | R.NLLLIK.G |  | 1 | 0.3503 | 23.340 | 0.031 | 357.25 | 1 | 2 | 5/10 | 5918 |
| 1161 | K.TDVTAEEVNEAFK.R |  | 1 | 0.7177 | 82.630 | 0.000 | 726.85 | 1 | 2 | 19/24 | 5922 |
| 1162 | K.CSEFGEELIK.N |  | 1 | 0.4223 | 45.790 | 0.000 | 606.28 | 0 | 2 | 7/18 | 5935 |
| 1163 | R.NNFDLRPAGIIK.M |  | 1 | 0.7662 | 45.100 | 0.881 | 679.38 | 1 | 2 | 7/22 | 5939 |
| 1164 | K.VYIHGFLDGR.D |  | 2 | 0.4938 | 39.250 | 0.000 | 588.81 | 1 | 2 | 8/18 | 5940 |
| 1165 | R.EIFDVCVNQK.L |  | 1 | 0.9732 | 44.300 | 0.992 | 626.31 | 0 | 2 | 12/18 | 5945 |
| 1166 | K.NHILETLHEQGINISK.F |  | 1 | 0.9303 | 42.680 | 0.952 | 616.00 | 1 | 3 | 10/30 | 5946 |
| 1167 | R.GIELLHLK.N |  | 2 | 0.2585 | 31.220 | 0.000 | 461.79 | 0 | 2 | 6/14 | 5947 |
| 1168 | K.EDTVLGGEYPISK.G |  | 1 | 0.8398 | 37.520 | 0.962 | 704.35 | 0 | 2 | 9/24 | 5948 |
| 1169 | R.AGVGVDNIDIDEATK.H |  | 1 | 0.2268 | 72.420 | 0.000 | 758.88 | 1 | 2 | 11/28 | 5949 |
| 1170 | R.HDVDLPWER.T |  | 1 | 0.5320 | 43.170 | 0.000 | 583.78 | 0 | 2 | 5/16 | 5950 |
| 1171 | R.AGTGLGLAIVK.N |  | 1 | 0.6389 | 44.700 | 0.815 | 500.31 | 1 | 2 | 9/20 | 5951 |
| 1172 | K.QYGVESQEVLNQVDR.L |  | 2 | 0.5276 | 93.440 | 0.000 | 882.43 | 0 | 2 | 12/28 | 5952 |
| 1173 | R.SWSFGEVK.K |  | 1 | 0.3534 | 31.870 | 0.000 | 470.23 | 0 | 2 | 5/14 | 5956 |
| 1174 | R.EHANAFTELNDPIDQR.E |  | 1 | 0.3825 | 41.880 | 0.000 | 623.96 | 1 | 3 | 9/30 | 5959 |
| 1175 | R.EISSCSNFEAFQAR.R |  | 1 | 0.5313 | 91.730 | 0.000 | 823.37 | 0 | 2 | 10/26 | 5960 |
| 1176 | R.LVSPFGAVK.L |  | 1 | 0.2810 | 22.670 | 0.534 | 459.28 | 0 | 2 | 4/16 | 5964 |
| 1177 | R.TDLPAFRPGDTLR.V |  | 1 | 0.6111 | 22.420 | 0.948 | 729.89 | 0 | 2 | 8/24 | 5967 |
| 1178 | K.LISVSLPR.V |  | 1 | 0.3270 | 33.390 | 0.000 | 442.78 | 0 | 2 | 6/14 | 5968 |
| 1179 | K.TGQTADEVLNTLNK.K |  | 1 | 0.8860 | 93.110 | 0.974 | 752.39 | 0 | 2 | 10/26 | 5970 |
| 1180 | R.SLEFLR.R |  | 1 | 0.5584 | 33.030 | 0.000 | 382.72 | 0 | 2 | 4/10 | 5972 |
| 1181 | R.VSQVLGPVVDVR.F |  | 1 | 0.7031 | 55.210 | 0.770 | 634.37 | 0 | 2 | 6/22 | 5989 |
| 1182 | K.DLGVDYCVIGHSER.R |  | 1 | 0.5741 | 59.710 | 0.000 | 810.38 | 0 | 2 | 9/26 | 5992 |
| 1183 | K.HYGGEPANFLDVGGGATAEK.V |  | 2 | 0.5645 | 81.330 | 0.899 | 995.47 | 0 | 2 | 9/38 | 5997 |
| 1184 | K.IQPVSDAPVNAISTCVK.G |  | 1 | 0.0581 | 29.420 | 0.000 | 899.97 | 0 | 2 | 8/32 | 6001 |
| 1185 | K.VTVPVAIHLDHGSSFESCAK.A |  | 1 | 0.1739 | 33.730 | 0.000 | 718.69 | 0 | 3 | 10/38 | 6005 |
| 1186 | K.TVTEQDVNNYLDSK.K |  | 1 | 0.9426 | 105.430 | 0.962 | 813.39 | 0 | 2 | 11/26 | 6007 |
| 1187 | K.GADQIFNALKK.T |  | 1 | 0.8117 | 27.670 | 0.962 | 402.23 | 0 | 3 | 10/20 | 6008 |
| 1188 | K.ALILHADHELNASTFTAR.V |  | 1 | 0.3351 | 70.080 | 0.000 | 660.68 | 1 | 3 | 11/34 | 6013 |
| 1189 | R.FNHSLGVYEIVR.R |  | 1 | 0.2872 | 39.610 | 0.000 | 478.59 | 0 | 3 | 9/22 | 6014 |
| 1190 | R.DDCLYVGITK.K |  | 1 | 0.3799 | 34.950 | 0.000 | 592.29 | 1 | 2 | 8/18 | 6015 |
| 1191 | K.TDDVVAEIAER.T |  | 2 | 0.4673 | 65.130 | 0.000 | 609.30 | 0 | 2 | 9/20 | 6021 |
| 1192 | R.DPETLKPVEYYVK.L |  | 2 | 0.9428 | 37.540 | 0.978 | 790.91 | 0 | 2 | 16/24 | 6024 |
| 1193 | K.QETHLPVFVDVTHSTGR.R |  | 1 | 0.3282 | 64.960 | 0.000 | 641.66 | 1 | 3 | 12/32 | 6025 |
| 1194 | K.IGTIAGGYVTEGTITR.D |  | 1 | 0.1732 | 88.130 | 0.000 | 804.93 | 0 | 2 | 12/30 | 6027 |
| 1195 | K.DTDSALKENEDIFR.E |  | 2 | 0.3008 | 71.860 | 0.000 | 826.89 | 0 | 2 | 10/26 | 6028 |
| 1196 | R.YYGGCEHVDVVEDIAR.D |  | 1 | 0.4272 | 30.200 | 0.000 | 627.95 | 0 | 3 | 15/30 | 6033 |
| 1197 | K.ALEQNLNPVVVVNK.I |  | 1 | 0.4520 | 47.880 | 0.000 | 768.94 | 0 | 2 | 15/26 | 6035 |
| 1198 | K.LINVWGADHHGYIPR.M |  | 1 | 0.5291 | 25.470 | 0.000 | 437.73 | 0 | 4 | 13/28 | 6040 |
| 1199 | K.LTHTGDGYSLVDFNR.Q |  | 1 | 0.1418 | 20.350 | 0.000 | 565.61 | 0 | 3 | 8/28 | 6044 |
| 1200 | K.FSAFIVEK.E |  | 1 | 0.8122 | 31.340 | 0.704 | 470.76 | 0 | 2 | 5/14 | 6048 |
| 1201 | K.GLTIQGITGR.K |  | 1 | 0.2730 | 53.410 | 0.000 | 508.30 | 0 | 2 | 8/18 | 6051 |
| 1202 | R.DINDFLDTKK.I |  | 1 | 0.2191 | 21.670 | 0.000 | 403.54 | 0 | 3 | 7/18 | 6052 |
| 1203 | R.DIPNVGEDALR.N |  | 1 | 0.1826 | 44.390 | 0.000 | 599.81 | 0 | 2 | 10/20 | 6055 |
| 1204 | R.QPASFCGVVGLKPTYGR.V |  | 1 | 0.0946 | 34.070 | 0.000 | 612.99 | 0 | 3 | 9/32 | 6067 |
| 1205 | K.AVSNPDRPFTAIIGGAK.V |  | 2 | 0.1218 | 62.950 | 0.000 | 857.47 | 0 | 2 | 15/32 | 6074 |
| 1206 | K.YNLLIGER.T |  | 1 | 0.5421 | 34.330 | 0.000 | 489.27 | 0 | 2 | 5/14 | 6078 |
| 1207 | K.AAYVFYPHTIYK.E |  | 2 | 0.4999 | 40.740 | 0.000 | 736.88 | 0 | 2 | 8/22 | 6083 |
| 1208 | R.VAGAVTGLWR.K |  | 1 | 0.8868 | 60.880 | 0.965 | 515.30 | 0 | 2 | 8/18 | 6090 |
| 1209 | K.EFQLTSAIK.K |  | 1 | 0.3453 | 20.910 | 0.574 | 518.79 | 1 | 2 | 5/16 | 6093 |
| 1210 | K.WLVVDAAGK.T |  | 1 | 0.7178 | 30.740 | 0.789 | 479.77 | 0 | 2 | 5/16 | 6099 |
| 1211 | K.ALTITNVPGSTLSR.E |  | 1 | 0.1079 | 38.890 | 0.000 | 715.40 | 0 | 2 | 10/26 | 6100 |
| 1212 | K.LLHDAVVPLEQ.- |  | 1 | 0.8207 | 24.210 | 0.776 | 617.35 | 0 | 2 | 5/20 | 6101 |
| 1213 | R.ASIYNAVSLEDCEK.L |  | 1 | 0.2792 | 59.000 | 0.000 | 799.87 | 1 | 2 | 8/26 | 6104 |
| 1214 | R.GAWLEYETDAK.D |  | 1 | 0.9289 | 56.940 | 0.931 | 641.80 | 0 | 2 | 8/20 | 6110 |
| 1215 | K.VISWYDNESGYSNR.V |  | 3 | 0.6365 | 75.650 | 0.000 | 845.38 | 0 | 2 | 11/26 | 6111 |
| 1216 | K.SLGSNTPINMIR.A |  | 1 | 0.1849 | 34.860 | 0.000 | 651.85 | 0 | 2 | 8/22 | 6116 |
| 1217 | K.HPETGEVLVNENELIDEDK.A |  | 1 | 0.2930 | 61.930 | 0.000 | 727.35 | 0 | 3 | 9/36 | 6124 |
| 1218 | K.AEHVSVEDVNTIIKDNEK.V |  | 1 | 0.2326 | 49.780 | 0.000 | 680.68 | 0 | 3 | 16/34 | 6125 |
| 1219 | K.EIIVSTPHPTAAFVAAR.A |  | 1 | 0.3071 | 43.020 | 0.000 | 890.49 | 0 | 2 | 20/32 | 6126 |
| 1220 | K.VIVLGEIR.D |  | 1 | 0.3565 | 31.520 | 0.000 | 449.79 | 0 | 2 | 7/14 | 6130 |
| 1221 | K.LVLQGGNIISESR.S |  | 1 | 0.3512 | 70.950 | 0.000 | 693.39 | 0 | 2 | 10/24 | 6131 |
| 1222 | K.EQQAELSILHVGR.E |  | 2 | 0.3861 | 67.910 | 0.000 | 740.40 | 0 | 2 | 10/24 | 6132 |
| 1223 | K.IFEFDDR.L |  | 1 | 0.9717 | 30.640 | 0.967 | 471.22 | 1 | 2 | 6/12 | 6147 |
| 1224 | R.DQLPTEDEQFDAYK.T |  | 1 | 0.4399 | 59.260 | 0.000 | 849.88 | 0 | 2 | 10/26 | 6154 |
| 1225 | R.SNNDWSCPIVVR.A |  | 1 | 0.4497 | 55.820 | 0.000 | 723.84 | 1 | 2 | 7/22 | 6155 |
| 1226 | R.AIEDHFDYSPELER.N |  | 2 | 0.4776 | 80.130 | 0.000 | 860.89 | 0 | 2 | 11/26 | 6162 |
| 1227 | K.LGANAILGVSM#ACAR.A |  | 1 | 0.7087 | 45.950 | 0.992 | 760.39 | 0 | 2 | 11/28 | 6164 |
| 1228 | R.VAEGLGLPIK.K |  | 1 | 0.4175 | 39.960 | 0.000 | 498.81 | 0 | 2 | 13/18 | 6169 |
| 1229 | R.GITITSAATTAQWK.G |  | 2 | 0.2394 | 75.750 | 0.000 | 724.89 | 0 | 2 | 8/26 | 6170 |
| 1230 | K.IAAYALTEPGSGSDALGAK.T |  | 2 | 0.8001 | 79.740 | 0.984 | 896.46 | 0 | 2 | 24/36 | 6172 |
| 1231 | K.KIVNATGPWVDQLR.E |  | 2 | 0.2542 | 72.700 | 0.000 | 798.95 | 0 | 2 | 10/26 | 6174 |
| 1232 | K.QLVGVLSYR.D |  | 1 | 0.7930 | 33.670 | 0.785 | 517.80 | 0 | 2 | 5/16 | 6175 |
| 1233 | K.TVLIFGGSR.G |  | 1 | 0.9165 | 44.640 | 0.919 | 475.28 | 0 | 2 | 8/16 | 6176 |
| 1234 | K.RPDQLVTVFAGQDK.E |  | 1 | 0.4882 | 69.340 | 0.000 | 787.42 | 0 | 2 | 17/26 | 6183 |
| 1235 | R.VHINILEIK.R |  | 2 | 0.3001 | 29.940 | 0.000 | 539.83 | 0 | 2 | 6/16 | 6184 |
| 1236 | K.APAVNYYDLEVNK.T |  | 1 | 0.3758 | 52.340 | 0.000 | 748.38 | 0 | 2 | 7/24 | 6186 |
| 1237 | K.NLQDFIGK.R |  | 1 | 0.2599 | 20.480 | 0.000 | 467.75 | 1 | 2 | 5/14 | 6191 |
| 1238 | R.GVLTYEEIAER.M |  | 1 | 0.7151 | 42.280 | 0.000 | 640.33 | 1 | 2 | 13/20 | 6200 |
| 1239 | K.RADLDAQLVADNIAR.Q |  | 2 | 0.8706 | 78.210 | 0.914 | 820.94 | 0 | 2 | 12/28 | 6205 |
| 1240 | R.LVGSPPGYVGYDEGGQLTEK.V |  | 2 | 0.2286 | 77.570 | 0.000 | 1033.51 | 0 | 2 | 15/38 | 6207 |
| 1241 | K.EIENEWEQKDPLVR.F |  | 1 | 0.2581 | 26.730 | 0.000 | 595.63 | 0 | 3 | 8/26 | 6208 |
| 1242 | R.CDANISLRPIGQEEFGTK.T |  | 2 | 0.2850 | 65.330 | 0.000 | 1018.00 | 0 | 2 | 17/34 | 6210 |
| 1243 | R.EGYELQVSKPEVIIK.E |  | 1 | 0.1779 | 61.180 | 0.000 | 866.48 | 0 | 2 | 11/28 | 6211 |
| 1244 | K.AANQVDDWIEEK.V |  | 1 | 0.5689 | 34.170 | 0.000 | 709.33 | 0 | 2 | 14/22 | 6218 |
| 1245 | K.VAEHVLDEIGITSNK.N |  | 2 | 0.5950 | 89.550 | 0.000 | 812.93 | 0 | 2 | 21/28 | 6225 |
| 1246 | K.VLPIYGGQDIGR.Q |  | 1 | 0.7556 | 35.130 | 0.913 | 644.36 | 0 | 2 | 7/22 | 6229 |
| 1247 | K.ELEAAGAHILGIK.D |  | 1 | 0.1890 | 29.600 | 0.000 | 441.25 | 0 | 3 | 14/24 | 6231 |
| 1248 | R.QIVEAVGGAENIAAATHCVTR.L |  | 2 | 0.2100 | 59.960 | 0.000 | 1084.05 | 0 | 2 | 15/40 | 6243 |
| 1249 | R.VFAYDFEGK.R |  | 1 | 0.9573 | 43.980 | 0.956 | 538.26 | 1 | 2 | 12/16 | 6246 |
| 1250 | R.VVQDGNEHPIIGLDNEALSTR.N |  | 1 | 0.4200 | 42.620 | 0.000 | 759.72 | 0 | 3 | 16/40 | 6248 |
| 1251 | K.LSSITDWEDR.N |  | 1 | 0.5319 | 46.330 | 0.000 | 611.29 | 0 | 2 | 7/18 | 6250 |
| 1252 | K.AIVVTTPEISAVR.D |  | 1 | 0.0972 | 23.920 | 0.000 | 678.40 | 0 | 2 | 9/24 | 6255 |
| 1253 | K.NLFGNETDPIK.V |  | 1 | 0.9681 | 45.330 | 0.972 | 624.32 | 1 | 2 | 13/20 | 6257 |
| 1254 | K.GELEGINFGESAK.A |  | 1 | 0.2556 | 54.710 | 0.000 | 675.83 | 0 | 2 | 10/24 | 6258 |
| 1255 | R.LPVIASLEK.L |  | 1 | 0.1782 | 32.410 | 0.000 | 485.30 | 0 | 2 | 4/16 | 6260 |
| 1256 | K.IFSLNSNPELAK.E |  | 1 | 0.8010 | 68.850 | 0.716 | 666.86 | 0 | 2 | 9/22 | 6261 |
| 1257 | R.VSALTPSTTLAITAK.A |  | 1 | 0.0538 | 31.430 | 0.000 | 737.43 | 0 | 2 | 15/28 | 6262 |
| 1258 | R.LKEQDLSIELTDAAK.A |  | 1 | 0.7626 | 29.480 | 0.896 | 558.64 | 0 | 3 | 15/28 | 6265 |
| 1259 | K.TLGIVGLGR.I |  | 1 | 0.2715 | 32.660 | 0.000 | 443.28 | 0 | 2 | 8/16 | 6269 |
| 1260 | K.LTEFGIIK.D |  | 1 | 0.4689 | 28.660 | 0.000 | 460.78 | 0 | 2 | 7/14 | 6271 |
| 1261 | R.STGPYSLVTQQPLGGK.A |  | 1 | 0.8028 | 76.560 | 0.955 | 816.93 | 1 | 2 | 12/30 | 6275 |
| 1262 | K.DFNSWVK.K |  | 1 | 0.9370 | 25.980 | 0.772 | 448.22 | 0 | 2 | 8/12 | 6282 |
| 1263 | R.GELQCIGATTLDEYRK.Y |  | 1 | 0.0940 | 32.590 | 0.000 | 618.64 | 0 | 3 | 10/30 | 6283 |
| 1264 | K.LYSIFK.A |  | 1 | 0.8773 | 22.820 | 0.879 | 385.73 | 1 | 2 | 5/10 | 6287 |
| 1265 | M.PYIVDVYAR.E |  | 1 | 0.4422 | 37.300 | 0.000 | 548.30 | 0 | 2 | 5/16 | 6290 |
| 1266 | K.HYYPHGVEYGETEEFPVQVVTK.S |  | 1 | 0.5876 | 23.530 | 0.000 | 652.81 | 0 | 4 | 16/42 | 6298 |
| 1267 | K.LLEESPSPALDSEIR.E |  | 1 | 0.4114 | 81.470 | 0.000 | 828.43 | 0 | 2 | 13/28 | 6299 |
| 1268 | R.ILEYLAVQK.L |  | 1 | 0.3783 | 38.660 | 0.000 | 538.82 | 0 | 2 | 7/16 | 6301 |
| 1269 | K.ENLLGEIGK.G |  | 1 | 0.7389 | 29.900 | 0.766 | 486.77 | 0 | 2 | 6/16 | 6303 |
| 1270 | R.FAALVVVGDK.N |  | 1 | 0.4275 | 59.170 | 0.000 | 509.80 | 0 | 2 | 14/18 | 6304 |
| 1271 | K.EELVGEYLNTNR.D |  | 1 | 0.5441 | 70.060 | 0.000 | 718.85 | 1 | 2 | 10/22 | 6308 |
| 1272 | K.GEPLVTLYANR.E |  | 1 | 0.3022 | 52.960 | 0.000 | 616.84 | 0 | 2 | 8/20 | 6313 |
| 1273 | K.IYDWVDER.L |  | 1 | 0.6035 | 34.440 | 0.000 | 548.26 | 0 | 2 | 7/14 | 6314 |
| 1274 | R.VVIELVESEEK.T |  | 1 | 0.6512 | 49.370 | 0.000 | 637.35 | 0 | 2 | 16/20 | 6323 |
| 1275 | K.IGLFGGAGVGK.T |  | 1 | 0.1058 | 35.320 | 0.000 | 488.28 | 0 | 2 | 6/20 | 6326 |
| 1276 | K.VLIVDDHLVVR.E |  | 1 | 0.2175 | 23.840 | 0.000 | 426.59 | 0 | 3 | 9/20 | 6331 |
| 1277 | R.VEPTASPDAWVVSGR.G |  | 1 | 0.8350 | 63.710 | 0.971 | 785.90 | 0 | 2 | 11/28 | 6335 |
| 1278 | K.LIDLVVR.E |  | 1 | 0.9721 | 39.930 | 0.908 | 414.27 | 0 | 2 | 6/12 | 6337 |
| 1279 | R.LANEILDAANNTGAAVK.K |  | 1 | 0.0875 | 65.120 | 0.000 | 842.95 | 0 | 2 | 11/32 | 6343 |
| 1280 | K.QPVQSNTTNFDILK.R |  | 1 | 0.3838 | 62.940 | 0.000 | 802.92 | 0 | 2 | 11/26 | 6349 |
| 1281 | R.EYFIAIK.G |  | 1 | 0.9003 | 28.660 | 0.863 | 442.25 | 0 | 2 | 5/12 | 6351 |
| 1282 | R.LAYEINDFR.D |  | 1 | 0.6843 | 48.350 | 0.000 | 570.79 | 0 | 2 | 7/16 | 6354 |
| 1283 | K.VGSFDAGYGSGYLAR.I |  | 1 | 0.7070 | 110.660 | 0.947 | 760.36 | 0 | 2 | 11/28 | 6355 |
| 1284 | K.EGVNIGLIGR.T |  | 1 | 0.1885 | 23.290 | 0.000 | 514.30 | 0 | 2 | 6/18 | 6363 |
| 1285 | K.MQENGTFDVLPK.K |  | 2 | 0.9327 | 62.330 | 0.917 | 689.84 | 2 | 2 | 9/22 | 6370 |
| 1286 | K.IGEVTDDLTEIK.E |  | 1 | 0.9109 | 94.500 | 0.941 | 666.85 | 1 | 2 | 9/22 | 6380 |
| 1287 | K.ALLEIYER.L |  | 1 | 0.5972 | 33.940 | 0.000 | 503.78 | 1 | 2 | 6/14 | 6385 |
| 1288 | R.IGALLTIER.D |  | 1 | 0.9109 | 55.590 | 0.942 | 493.31 | 0 | 2 | 8/16 | 6389 |
| 1289 | R.HVLSVGEPLNPEVIR.W |  | 1 | 0.2431 | 31.080 | 0.000 | 553.65 | 0 | 3 | 8/28 | 6395 |
| 1290 | K.CDVIAEGVVEATR.Q |  | 2 | 0.5239 | 88.280 | 0.000 | 709.85 | 0 | 2 | 9/24 | 6397 |
| 1291 | R.IIELPIK.S |  | 1 | 0.3258 | 32.140 | 0.000 | 413.28 | 0 | 2 | 4/12 | 6400 |
| 1292 | K.LILADEPTGALDTK.T |  | 1 | 0.9087 | 49.950 | 0.914 | 728.90 | 0 | 2 | 17/26 | 6402 |
| 1293 | K.LNFDSNALYR.Q |  | 1 | 0.7141 | 89.530 | 0.000 | 606.80 | 0 | 2 | 8/18 | 6405 |
| 1294 | K.VVVTTPELLER.I |  | 1 | 0.2031 | 24.450 | 0.000 | 628.37 | 0 | 2 | 7/20 | 6406 |
| 1295 | K.NTDEALLAYQNGYVHLHTR.V |  | 1 | 0.7335 | 47.120 | 0.000 | 739.03 | 0 | 3 | 23/36 | 6407 |
| 1296 | R.FISTNGDIAIPTER.G |  | 1 | 0.3323 | 54.570 | 0.000 | 767.40 | 0 | 2 | 12/26 | 6409 |
| 1297 | K.CIHLSEEKEPQIFNAIR.F |  | 1 | 0.6195 | 32.760 | 0.829 | 521.77 | 0 | 4 | 5/32 | 6416 |
| 1298 | K.LNELLSDPEVVNDPK.K |  | 1 | 0.6183 | 68.480 | 0.000 | 841.43 | 1 | 2 | 19/28 | 6419 |
| 1299 | K.TVLGVDPAFR.T |  | 1 | 0.8929 | 31.830 | 0.994 | 537.80 | 0 | 2 | 5/18 | 6420 |
| 1300 | R.ALQAAGLEVTAIR.D |  | 2 | 0.3126 | 78.850 | 0.000 | 656.88 | 1 | 2 | 10/24 | 6425 |
| 1301 | R.LNVLDLQR.L |  | 1 | 0.9673 | 43.080 | 0.938 | 485.79 | 0 | 2 | 7/14 | 6426 |
| 1302 | R.NVTFEAIAAGR.T |  | 1 | 0.4908 | 43.010 | 0.000 | 574.81 | 1 | 2 | 13/20 | 6428 |
| 1303 | K.IHANVPVVYYGTGEENDFQAR.N |  | 1 | 0.4769 | 64.970 | 0.000 | 793.72 | 1 | 3 | 16/40 | 6439 |
| 1304 | K.INQVDAWYESEESR.S |  | 1 | 0.6025 | 85.080 | 0.000 | 863.39 | 0 | 2 | 11/26 | 6440 |
| 1305 | K.TTLLDSIR.K |  | 1 | 0.4579 | 45.900 | 0.000 | 459.77 | 0 | 2 | 7/14 | 6443 |
| 1306 | R.IYIIGCGTSYHAGLVGK.Q |  | 1 | 0.9280 | 82.100 | 0.987 | 904.97 | 0 | 2 | 13/32 | 6444 |
| 1307 | K.VISISPDFAESSK.F |  | 1 | 0.1528 | 41.370 | 0.000 | 690.36 | 0 | 2 | 9/24 | 6446 |
| 1308 | K.DFIVKPFQADR.V |  | 2 | 0.1574 | 28.200 | 0.000 | 668.36 | 1 | 2 | 7/20 | 6447 |
| 1309 | R.LYSVLEELKK.- |  | 1 | 0.3402 | 23.660 | 0.592 | 407.91 | 0 | 3 | 6/18 | 6452 |
| 1310 | K.TFVSISSVEDLNKK.V |  | 1 | 0.8487 | 59.050 | 0.959 | 783.92 | 0 | 2 | 7/26 | 6453 |
| 1311 | K.AAEVPIIVAVNK.I |  | 1 | 0.8767 | 67.170 | 0.821 | 612.37 | 0 | 2 | 13/22 | 6457 |
| 1312 | K.WTNAEGEGVWIK.Y |  | 1 | 0.5749 | 87.100 | 0.000 | 695.34 | 0 | 2 | 9/22 | 6472 |
| 1313 | K.GQLFETFR.G |  | 1 | 0.9359 | 29.360 | 0.983 | 499.26 | 1 | 2 | 5/14 | 6474 |
| 1314 | K.NNTLTCYNGIIADGCGECPACHLR.S |  | 1 | 0.6513 | 71.980 | 0.000 | 922.73 | 0 | 3 | 20/46 | 6478 |
| 1315 | K.GSLATVRPDDLGAICVK.E |  | 1 | 0.0966 | 29.700 | 0.000 | 591.32 | 0 | 3 | 14/32 | 6480 |
| 1316 | R.AVIGESIGTIVR.G |  | 1 | 0.9190 | 56.130 | 0.991 | 607.86 | 0 | 2 | 9/22 | 6481 |
| 1317 | R.DVLPDPIYNSK.L |  | 1 | 0.3079 | 28.120 | 0.000 | 630.83 | 0 | 2 | 13/20 | 6483 |
| 1318 | K.IAFITTDTYR.I |  | 1 | 0.9124 | 43.550 | 0.935 | 600.82 | 0 | 2 | 8/18 | 6490 |
| 1319 | R.EILNEYVIGQDQAK.K |  | 1 | 0.7307 | 75.160 | 0.000 | 810.42 | 0 | 2 | 19/26 | 6498 |
| 1320 | K.DGELPSTFVPGR.N |  | 1 | 0.2179 | 41.640 | 0.000 | 637.82 | 0 | 2 | 8/22 | 6500 |
| 1321 | R.SIIIGVPYK.D |  | 1 | 0.4367 | 33.300 | 0.000 | 495.31 | 0 | 2 | 7/16 | 6502 |
| 1322 | R.TTVGPWFR.E |  | 1 | 0.3161 | 24.580 | 0.000 | 482.26 | 1 | 2 | 5/14 | 6503 |
| 1323 | R.VLEFALK.Y |  | 1 | 0.6969 | 25.560 | 0.560 | 410.25 | 0 | 2 | 6/12 | 6506 |
| 1324 | K.ALDLSSYYK.N |  | 1 | 0.4833 | 46.040 | 0.000 | 530.27 | 0 | 2 | 7/16 | 6509 |
| 1325 | K.ELCELPSNWR.S |  | 1 | 0.2559 | 21.840 | 0.000 | 652.31 | 0 | 2 | 8/18 | 6514 |
| 1326 | K.GSIVDIDETVHSIR.K |  | 1 | 0.7714 | 21.790 | 0.863 | 514.27 | 0 | 3 | 6/26 | 6516 |
| 1327 | R.AIFNNVSFR.L |  | 1 | 0.3097 | 31.620 | 0.000 | 534.28 | 1 | 2 | 6/16 | 6521 |
| 1328 | R.LIFTVPSR.G |  | 1 | 0.6955 | 27.880 | 0.461 | 466.78 | 1 | 2 | 4/14 | 6523 |
| 1329 | K.FSGWLVK.Q |  | 1 | 0.7964 | 27.430 | 0.791 | 418.74 | 0 | 2 | 6/12 | 6525 |
| 1330 | R.MLICCPTNITSVEQK.A |  | 1 | 0.3767 | 83.240 | 0.000 | 897.43 | 1 | 2 | 11/28 | 6527 |
| 1331 | K.DLSLAYSPGVAEPCKDIHEDINK.V |  | 1 | 0.1732 | 23.510 | 0.000 | 643.57 | 0 | 4 | 15/44 | 6537 |
| 1332 | R.DLEDQYFGR.I |  | 1 | 0.5695 | 35.470 | 0.000 | 571.76 | 0 | 2 | 5/16 | 6548 |
| 1333 | R.TNLGIQVIEEVKK.Y |  | 1 | 0.0825 | 20.940 | 0.000 | 490.96 | 0 | 3 | 12/24 | 6549 |
| 1334 | R.NCTGAIVGYHPFGGFK.M |  | 1 | 0.2289 | 22.210 | 0.000 | 575.61 | 0 | 3 | 11/30 | 6550 |
| 1335 | R.GLAGAFNSSVLR.S |  | 1 | 0.2450 | 77.450 | 0.000 | 596.33 | 0 | 2 | 9/22 | 6559 |
| 1336 | K.NIGAILQTDQAEADKNIAQAK.A |  | 1 | 0.0690 | 26.180 | 0.000 | 738.06 | 0 | 3 | 14/40 | 6562 |
| 1337 | R.LIIRPGQDAVFLNNGR.V |  | 1 | 0.5552 | 23.820 | 0.944 | 595.00 | 0 | 3 | 9/30 | 6573 |
| 1338 | R.INFIGIR.L |  | 1 | 0.5949 | 47.910 | 0.000 | 416.76 | 1 | 2 | 6/12 | 6574 |
| 1339 | K.KGHVGVVSR.S |  | 1 | 0.1166 | 26.880 | 0.000 | 313.52 | 1 | 3 | 6/16 | 657 |
| 1340 | R.ELLEEHNAQDIQIIPK.I |  | 2 | 0.9645 | 70.690 | 0.905 | 945.50 | 1 | 2 | 15/30 | 6588 |
| 1341 | K.NGEFIDVTNEDLK.G |  | 1 | 0.4759 | 56.750 | 0.000 | 747.36 | 0 | 2 | 10/24 | 6589 |
| 1342 | K.SVAPALGAGNGVVLKPHEETPICGGTLIAK.I |  | 1 | 0.3478 | 56.820 | 0.921 | 986.20 | 0 | 3 | 14/58 | 6593 |
| 1343 | R.WNEIETTDEALYK.W |  | 1 | 0.5408 | 83.110 | 0.000 | 806.38 | 0 | 2 | 11/24 | 6597 |
| 1344 | K.ATFAAFIK.R |  | 1 | 0.2343 | 22.750 | 0.000 | 434.75 | 1 | 2 | 6/14 | 6600 |
| 1345 | R.QEVPEEEYTIELGK.A |  | 1 | 0.9171 | 49.650 | 0.983 | 832.41 | 0 | 2 | 8/26 | 6607 |
| 1346 | R.ISKPEEYPVDLSEDCWSNK.M |  | 1 | 0.3180 | 46.610 | 0.000 | 766.02 | 0 | 3 | 11/36 | 6608 |
| 1347 | K.QALFSDPSIVR.E |  | 1 | 0.2643 | 43.970 | 0.000 | 616.84 | 0 | 2 | 7/20 | 6611 |
| 1348 | R.VHEQQSVSYLFS.- |  | 1 | 0.4929 | 32.750 | 0.000 | 712.35 | 0 | 2 | 9/22 | 6613 |
| 1349 | K.LEGGEGVFESIR.A |  | 1 | 0.9189 | 61.020 | 0.952 | 646.83 | 0 | 2 | 10/22 | 6616 |
| 1350 | K.DSTGICFIGER.N |  | 1 | 0.3388 | 36.270 | 0.000 | 627.79 | 0 | 2 | 5/20 | 6619 |
| 1351 | K.GISDVVFDR.G |  | 1 | 0.8689 | 60.030 | 0.710 | 504.26 | 0 | 2 | 7/16 | 6625 |
| 1352 | R.TPPELSADIIDR.G |  | 1 | 0.4121 | 47.770 | 0.000 | 663.85 | 0 | 2 | 8/22 | 6628 |
| 1353 | K.VSQLSTCPVLIVR.- |  | 1 | 0.8835 | 52.270 | 0.910 | 736.42 | 0 | 2 | 10/24 | 6629 |
| 1354 | K.GDSVVTIGGLHGTVDSIDESK.V |  | 2 | 0.0999 | 62.550 | 0.000 | 1043.52 | 0 | 2 | 14/40 | 6632 |
| 1355 | K.LIAPSIAEGLR.R |  | 1 | 0.1191 | 20.570 | 0.000 | 570.34 | 0 | 2 | 8/20 | 6641 |
| 1356 | K.ALFGVLQK.I |  | 1 | 0.8487 | 44.140 | 0.850 | 438.27 | 0 | 2 | 7/14 | 6647 |
| 1357 | R.ENTEDIYAGIEYAK.G |  | 1 | 0.3003 | 83.920 | 0.000 | 808.38 | 0 | 2 | 9/26 | 6648 |
| 1358 | K.EKGETFDQYEGSTYATGEYFDK.Y |  | 1 | 0.3646 | 45.100 | 0.000 | 855.70 | 1 | 3 | 23/42 | 6649 |
| 1359 | K.LGAFIAEGALK.H |  | 1 | 0.6223 | 49.450 | 0.694 | 545.32 | 0 | 2 | 8/20 | 6660 |
| 1360 | K.HSIIIVAEGVGSGVEFGKR.I |  | 1 | 0.2355 | 69.080 | 0.000 | 652.36 | 0 | 3 | 23/36 | 6663 |
| 1361 | K.VFTWAQYDR.I |  | 1 | 0.5030 | 40.320 | 0.000 | 593.29 | 0 | 2 | 7/16 | 6668 |
| 1362 | R.VVVQGFGNAGSYLAK.F |  | 2 | 0.3125 | 90.250 | 0.000 | 755.41 | 0 | 2 | 12/28 | 6681 |
| 1363 | K.GELPLFEK.A |  | 1 | 0.2830 | 30.380 | 0.000 | 466.76 | 0 | 2 | 6/14 | 6683 |
| 1364 | K.YVPIEDPEAFR.E |  | 1 | 0.2255 | 21.080 | 0.000 | 668.33 | 1 | 2 | 6/20 | 6684 |
| 1365 | R.AIEEILSVAK.D |  | 1 | 0.9439 | 39.860 | 0.903 | 536.82 | 1 | 2 | 13/18 | 6685 |
| 1366 | K.TILGLVPR.A |  | 1 | 0.4584 | 40.370 | 0.108 | 434.78 | 0 | 2 | 6/14 | 6686 |
| 1367 | K.DTGQDSCLSCVV.- |  | 1 | 0.4989 | 40.630 | 0.000 | 670.78 | 0 | 2 | 9/22 | 6687 |
| 1368 | K.YDAANHDVISNASCTTNCLAPFAK.V |  | 1 | 0.2101 | 43.770 | 0.000 | 880.73 | 0 | 3 | 14/46 | 6688 |
| 1369 | K.GYIVPGLGDAGDR.M |  | 1 | 0.1829 | 36.650 | 0.000 | 645.33 | 0 | 2 | 10/24 | 6690 |
| 1370 | R.VIVQGITGSTALFHTK.Q |  | 2 | 0.9088 | 76.710 | 0.963 | 836.47 | 0 | 2 | 12/30 | 6695 |
| 1371 | K.LAIGLLR.N |  | 1 | 0.3147 | 33.630 | 0.000 | 378.26 | 0 | 2 | 6/12 | 6696 |
| 1372 | R.QGSGAAYLNIFHR.D |  | 2 | 0.2794 | 50.430 | 0.000 | 717.37 | 1 | 2 | 10/24 | 6697 |
| 1373 | K.NAIPYDPEKPFVTSGIR.L |  | 1 | 0.6256 | 68.890 | 0.975 | 952.50 | 0 | 2 | 8/32 | 6702 |
| 1374 | K.LEELGLGLR.K |  | 1 | 0.9301 | 46.050 | 0.921 | 500.30 | 0 | 2 | 8/16 | 6707 |
| 1375 | R.AGATYVSPFLGR.L |  | 1 | 0.7612 | 72.080 | 0.982 | 619.83 | 0 | 2 | 8/22 | 6708 |
| 1376 | R.GLELVGVGYR.A |  | 1 | 0.3924 | 69.730 | 0.000 | 531.80 | 0 | 2 | 8/18 | 6713 |
| 1377 | K.RLEVLEAFR.N |  | 2 | 0.6113 | 42.520 | 0.000 | 566.83 | 0 | 2 | 13/16 | 6714 |
| 1378 | K.GNLFVYVR.E |  | 1 | 0.9282 | 47.010 | 0.856 | 484.27 | 0 | 2 | 6/14 | 6724 |
| 1379 | K.NTITVPYNDLESVK.L |  | 1 | 0.8613 | 45.420 | 0.944 | 796.91 | 0 | 2 | 13/26 | 6728 |
| 1380 | K.IAAGADAIVLDVK.T |  | 1 | 0.2641 | 95.690 | 0.000 | 628.37 | 1 | 2 | 11/24 | 6736 |
| 1381 | K.KVPNELSNGGTVISVPEDQVDSLK.V |  | 1 | 0.0453 | 20.640 | 0.000 | 842.44 | 0 | 3 | 16/46 | 6742 |
| 1382 | R.DYIHVVDLAEGHVK.A |  | 1 | 0.5386 | 33.230 | 0.000 | 532.28 | 0 | 3 | 13/26 | 6756 |
| 1383 | R.ALVPSGASTGEYEAVELR.D |  | 2 | 0.8160 | 124.710 | 0.994 | 924.97 | 0 | 2 | 12/34 | 6757 |
| 1384 | K.AAGATDIYAVELSPER.Q |  | 2 | 0.4243 | 65.910 | 0.000 | 831.92 | 0 | 2 | 19/30 | 6759 |
| 1385 | K.YGINIVAIK.R |  | 1 | 0.1226 | 25.760 | 0.000 | 495.80 | 0 | 2 | 8/16 | 6760 |
| 1386 | K.INVTIPAGVDDGQQLR.L |  | 1 | 0.1743 | 49.620 | 0.000 | 848.45 | 0 | 2 | 15/30 | 6762 |
| 1387 | R.GPDSDGYFHDEHVGFGFR.R |  | 1 | 0.5309 | 21.960 | 0.000 | 510.47 | 0 | 4 | 15/34 | 6768 |
| 1388 | R.GYDSAGIAVANEQGIHVFK.E |  | 2 | 0.1295 | 59.090 | 0.000 | 988.50 | 0 | 2 | 11/36 | 6773 |
| 1389 | K.QTFTQQEAANIIEAR.V |  | 2 | 0.3285 | 101.640 | 0.000 | 860.44 | 1 | 2 | 11/28 | 6780 |
| 1390 | K.FDQTPALNQTCDIGEIAK.I |  | 2 | 0.2503 | 73.640 | 0.000 | 1010.99 | 0 | 2 | 9/34 | 6782 |
| 1391 | K.ADIVLIGVSR.T |  | 1 | 0.2879 | 61.360 | 0.000 | 521.82 | 0 | 2 | 8/18 | 6783 |
| 1392 | R.GVVTPVIDLR.K |  | 1 | 0.1713 | 32.130 | 0.000 | 534.82 | 1 | 2 | 7/18 | 6794 |
| 1393 | K.LYVPVEQIDQVQK.Y |  | 1 | 0.1851 | 30.490 | 0.000 | 779.93 | 0 | 2 | 5/24 | 6798 |
| 1394 | R.INILDTPGHADFGGEVER.I |  | 1 | 0.9099 | 51.240 | 0.999 | 647.32 | 1 | 3 | 18/34 | 6801 |
| 1395 | K.NIILGFSR.N |  | 1 | 0.4641 | 29.730 | 0.000 | 460.27 | 0 | 2 | 6/14 | 6806 |
| 1396 | R.VSITDDAIEAAVK.L |  | 1 | 0.2613 | 79.760 | 0.000 | 666.36 | 0 | 2 | 11/24 | 6807 |
| 1397 | R.VFVLGEDVGR.K |  | 1 | 0.6400 | 55.800 | 0.000 | 545.80 | 0 | 2 | 12/18 | 6817 |
| 1398 | R.IIALGGVGEIGK.N |  | 1 | 0.0884 | 34.680 | 0.000 | 563.85 | 0 | 2 | 6/22 | 6825 |
| 1399 | K.DTDVSIEQIVK.Q |  | 1 | 0.9297 | 36.100 | 0.905 | 623.83 | 0 | 2 | 14/20 | 6829 |
| 1400 | R.FQNLCVVGDSDQSIYR.W |  | 1 | 0.3399 | 67.170 | 0.000 | 950.95 | 0 | 2 | 9/30 | 6834 |
| 1401 | R.MYDFLDK.L |  | 1 | 0.2573 | 21.820 | 0.000 | 466.21 | 0 | 2 | 5/12 | 6849 |
| 1402 | K.LGVVPILR.A |  | 1 | 0.2778 | 37.030 | 0.000 | 433.79 | 0 | 2 | 4/14 | 6860 |
| 1403 | R.VLPHPVFQELTVR.Q |  | 1 | 0.4715 | 46.000 | 0.000 | 512.30 | 0 | 3 | 13/24 | 6864 |
| 1404 | K.LLQDIDLVK.E |  | 1 | 0.9124 | 49.500 | 0.799 | 528.82 | 0 | 2 | 7/16 | 6866 |
| 1405 | K.YSSEFYGPFR.D |  | 1 | 0.3355 | 33.110 | 0.000 | 626.79 | 0 | 2 | 7/18 | 6871 |
| 1406 | R.FATSDLNDLYR.R |  | 1 | 0.5797 | 54.380 | 0.000 | 657.82 | 0 | 2 | 9/20 | 6878 |
| 1407 | R.SDLLDAIK.E |  | 1 | 0.7833 | 29.400 | 0.695 | 437.75 | 0 | 2 | 6/14 | 6880 |
| 1408 | K.LDILGHDDPTVIR.M |  | 1 | 0.1794 | 29.230 | 0.000 | 488.60 | 1 | 3 | 10/24 | 6882 |
| 1409 | K.FDIHPIYITEK.G |  | 1 | 0.5256 | 27.030 | 0.000 | 459.25 | 0 | 3 | 10/20 | 6887 |
| 1410 | K.TLLVDLDGAAK.A |  | 1 | 0.6830 | 25.750 | 0.977 | 558.32 | 1 | 2 | 4/20 | 6892 |
| 1411 | K.SLKEELFNLR.F |  | 2 | 0.8468 | 36.770 | 0.897 | 624.85 | 0 | 2 | 7/18 | 6893 |
| 1412 | K.ILGTSLEDLDR.A |  | 1 | 0.8364 | 38.700 | 0.813 | 616.33 | 1 | 2 | 9/20 | 6899 |
| 1413 | R.NSDFAITACTWTNK.K |  | 1 | 0.4152 | 71.800 | 0.000 | 814.87 | 0 | 2 | 10/26 | 6904 |
| 1414 | R.SDIFLIEK.G |  | 1 | 0.6714 | 32.720 | 0.000 | 482.77 | 0 | 2 | 11/14 | 6905 |
| 1415 | K.LNIIGEDDIVK.F |  | 1 | 0.3781 | 48.090 | 0.000 | 614.84 | 1 | 2 | 9/20 | 6909 |
| 1416 | K.TSLGSIVCTGDFK.F |  | 1 | 0.4043 | 68.440 | 0.000 | 692.84 | 0 | 2 | 10/24 | 6911 |
| 1417 | K.NLEDIVIVSPDHGGVTR.A |  | 2 | 0.9478 | 76.300 | 0.927 | 910.98 | 0 | 2 | 22/32 | 6912 |
| 1418 | R.GTALLHILIK.T |  | 1 | 0.2278 | 32.460 | 0.000 | 360.24 | 0 | 3 | 6/18 | 6914 |
| 1419 | R.DGTGFIQGVVVK.A |  | 1 | 0.8625 | 49.150 | 0.987 | 610.34 | 1 | 2 | 7/22 | 6917 |
| 1420 | R.ATLEDCPPELSGDIVDR.G |  | 1 | 0.3457 | 82.810 | 0.000 | 943.94 | 0 | 2 | 13/32 | 6921 |
| 1421 | K.AFQLAVLK.G |  | 1 | 0.8818 | 33.260 | 0.873 | 445.28 | 0 | 2 | 6/14 | 6925 |
| 1422 | K.VLLESVLR.Q |  | 1 | 0.4268 | 36.390 | 0.000 | 464.79 | 0 | 2 | 7/14 | 6938 |
| 1423 | K.VQAFLALDEER.A |  | 1 | 0.9571 | 58.010 | 0.890 | 645.84 | 0 | 2 | 7/20 | 6941 |
| 1424 | K.LDAEVSVDGNNLVVNGK.T |  | 2 | 0.1940 | 55.510 | 0.000 | 871.95 | 0 | 2 | 16/32 | 6943 |
| 1425 | K.LANEGFYDGLTFHR.V |  | 1 | 0.3602 | 55.800 | 0.000 | 547.27 | 0 | 3 | 11/26 | 6949 |
| 1426 | K.VIVAINKDPEADIFK.I |  | 1 | 0.5392 | 28.240 | 0.734 | 557.98 | 0 | 3 | 8/28 | 6950 |
| 1427 | R.ADFYVADIGEGAR.E |  | 1 | 0.2136 | 56.930 | 0.000 | 692.33 | 1 | 2 | 7/24 | 6952 |
| 1428 | R.LEAEGYDWIK.Q |  | 1 | 0.5401 | 47.740 | 0.000 | 612.30 | 0 | 2 | 7/18 | 6960 |
| 1429 | K.VGDEVEIIGLQEENK.K |  | 1 | 0.3558 | 66.750 | 0.000 | 836.43 | 1 | 2 | 12/28 | 6962 |
| 1430 | K.LFNGFIK.T |  | 1 | 0.7067 | 30.380 | 0.425 | 419.75 | 1 | 2 | 8/12 | 6964 |
| 1431 | R.FALDPVLK.S |  | 1 | 0.8930 | 31.870 | 0.945 | 451.77 | 0 | 2 | 4/14 | 6969 |
| 1432 | K.TFTVSNLIK.E |  | 1 | 0.2961 | 21.170 | 0.000 | 511.80 | 0 | 2 | 11/16 | 6970 |
| 1433 | R.LTSYITALTGPK.L |  | 1 | 0.2465 | 63.670 | 0.000 | 632.86 | 0 | 2 | 9/22 | 6971 |
| 1434 | R.IM#EVPVGEELIGR.I |  | 1 | 0.7979 | 44.760 | 0.965 | 729.39 | 0 | 2 | 6/24 | 6974 |
| 1435 | R.GTVALPVASSWGEAK.T |  | 1 | 0.1552 | 50.310 | 0.000 | 736.89 | 0 | 2 | 15/28 | 6984 |
| 1436 | R.TLGSPFVLSSDR.K |  | 1 | 0.2876 | 40.400 | 0.000 | 639.84 | 0 | 2 | 8/22 | 6992 |
| 1437 | K.TGLPTELDLQEPER.V |  | 1 | 0.4945 | 59.780 | 0.000 | 799.41 | 0 | 2 | 9/26 | 6993 |
| 1438 | K.VPGLVAAFSR.I |  | 1 | 0.7679 | 43.980 | 0.640 | 508.80 | 0 | 2 | 8/18 | 6994 |
| 1439 | R.FDFSHFGQVTK.E |  | 1 | 0.4439 | 31.900 | 0.000 | 438.22 | 0 | 3 | 12/20 | 6998 |
| 1440 | R.HSSDEEPFSALAFK.V |  | 2 | 0.2937 | 69.850 | 0.000 | 782.87 | 0 | 2 | 8/26 | 7001 |
| 1441 | R.AINEDGNVTTFEAVVR.F |  | 2 | 0.3683 | 95.620 | 0.000 | 867.94 | 0 | 2 | 12/30 | 7016 |
| 1442 | K.LQDGQFSECDLTFK.E |  | 1 | 0.5201 | 57.230 | 0.000 | 844.39 | 0 | 2 | 11/26 | 7019 |
| 1443 | K.CAHCYLESSPEALGTVSIEQFKK.T |  | 1 | 0.7335 | 45.170 | 0.000 | 885.42 | 0 | 3 | 31/44 | 7030 |
| 1444 | R.LVHVFSLPLK.Y |  | 1 | 0.8957 | 29.490 | 0.647 | 384.91 | 0 | 3 | 13/18 | 7032 |
| 1445 | K.EQEEQLTELIASK.K |  | 1 | 0.3244 | 44.620 | 0.000 | 759.39 | 1 | 2 | 9/24 | 7037 |
| 1446 | R.LNLPEYEITDETR.I |  | 1 | 0.5230 | 56.980 | 0.000 | 796.89 | 0 | 2 | 10/24 | 7038 |
| 1447 | R.ILELLSER.E |  | 1 | 0.9498 | 50.700 | 0.797 | 486.79 | 0 | 2 | 6/14 | 7039 |
| 1448 | R.GIVLTGGGALLR.N |  | 1 | 0.1867 | 59.920 | 0.000 | 563.85 | 0 | 2 | 9/22 | 7042 |
| 1449 | K.CGIIDLPYGGGK.G |  | 1 | 0.8152 | 51.280 | 0.964 | 625.32 | 1 | 2 | 6/22 | 7044 |
| 1450 | K.DSVGDDQYEIFK.S |  | 1 | 0.5422 | 50.730 | 0.000 | 708.32 | 0 | 2 | 9/22 | 7048 |
| 1451 | K.LGGIIEIYEK.F |  | 1 | 0.5832 | 48.610 | 0.000 | 567.82 | 0 | 2 | 12/18 | 7053 |
| 1452 | R.QQYEQLVEELK.Q |  | 1 | 0.9630 | 58.840 | 0.933 | 703.86 | 0 | 2 | 8/20 | 7055 |
| 1453 | K.LGISFETIK.S |  | 1 | 0.9016 | 25.160 | 0.956 | 504.29 | 0 | 2 | 11/16 | 7060 |
| 1454 | R.ASYIAELDASDIEK.G |  | 1 | 0.8453 | 76.380 | 0.977 | 762.87 | 0 | 2 | 8/26 | 7065 |
| 1455 | K.FGSPLITNDGVTIAK.E |  | 1 | 0.8329 | 54.160 | 0.877 | 766.92 | 0 | 2 | 11/28 | 7067 |
| 1456 | R.LVDVAQDVIIR.E |  | 1 | 0.2624 | 32.050 | 0.000 | 620.87 | 0 | 2 | 6/20 | 7068 |
| 1457 | K.AFEVYQDEVVYER.H |  | 1 | 0.6545 | 84.150 | 0.000 | 823.89 | 0 | 2 | 9/24 | 7070 |
| 1458 | K.LAGEPVDILVNQR.I |  | 1 | 0.2968 | 71.230 | 0.000 | 712.40 | 0 | 2 | 9/24 | 7072 |
| 1459 | K.LELGSVGDIIHR.G |  | 2 | 0.4202 | 70.900 | 0.000 | 654.87 | 0 | 2 | 9/22 | 7073 |
| 1460 | K.EDFVLPGYR.D |  | 1 | 0.8978 | 23.120 | 0.978 | 548.28 | 0 | 2 | 7/16 | 7076 |
| 1461 | R.KLLDTWDSFK.A |  | 1 | 0.1933 | 28.180 | 0.000 | 418.22 | 0 | 3 | 8/18 | 7079 |
| 1462 | K.TANIGDVIVCTVK.Q |  | 2 | 0.2050 | 45.910 | 0.000 | 695.37 | 0 | 2 | 11/24 | 7081 |
| 1463 | K.DYPLIEVGR.M |  | 1 | 0.9672 | 45.280 | 0.948 | 531.28 | 0 | 2 | 11/16 | 7082 |
| 1464 | R.ILYLAAPIR.L |  | 1 | 0.3958 | 40.380 | 0.000 | 515.33 | 0 | 2 | 6/16 | 7086 |
| 1465 | R.GACDADYCDYALQIGQTGK.V |  | 2 | 0.2232 | 89.190 | 0.000 | 1053.45 | 1 | 2 | 10/36 | 7089 |
| 1466 | R.NANFTGEHFADQEFDVNAVTEK.D |  | 2 | 0.1799 | 79.050 | 0.000 | 1242.06 | 0 | 2 | 12/42 | 7096 |
| 1467 | K.AQVLYSCIDESNGFYK.G |  | 1 | 0.4858 | 91.310 | 0.000 | 947.44 | 0 | 2 | 10/30 | 7098 |
| 1468 | K.DGLTEILR.K |  | 1 | 0.9287 | 48.660 | 0.771 | 458.76 | 0 | 2 | 6/14 | 7101 |
| 1469 | K.AEEAAWDAYQYYK.D |  | 1 | 0.5251 | 65.370 | 0.000 | 804.35 | 0 | 2 | 10/24 | 7110 |
| 1470 | K.ALSLVLPHLK.G |  | 1 | 0.3765 | 38.790 | 0.000 | 545.85 | 0 | 2 | 11/18 | 7111 |
| 1471 | K.DITSILR.V |  | 1 | 0.7745 | 25.300 | 0.420 | 409.24 | 0 | 2 | 4/12 | 7112 |
| 1472 | K.LSDIAILYR.T |  | 1 | 0.5238 | 48.250 | 0.000 | 532.31 | 1 | 2 | 8/16 | 7114 |
| 1473 | R.AETALEVFASR.I |  | 1 | 0.3754 | 63.500 | 0.000 | 597.31 | 0 | 2 | 9/20 | 7115 |
| 1474 | K.LITAYDQPALGAVYK.L |  | 1 | 0.2210 | 74.470 | 0.000 | 811.94 | 1 | 2 | 9/28 | 7116 |
| 1475 | K.YDVIVGPTTPTPAFK.I |  | 1 | 0.3987 | 61.940 | 0.000 | 803.43 | 1 | 2 | 18/28 | 7117 |
| 1476 | K.LFSIVPVK.R |  | 1 | 0.1577 | 21.260 | 0.000 | 451.79 | 0 | 2 | 5/14 | 7119 |
| 1477 | R.LNAGCSLATLQDGLR.R |  | 2 | 0.2784 | 96.470 | 0.000 | 794.91 | 1 | 2 | 10/28 | 7125 |
| 1478 | K.IVTVWGVGYK.F |  | 1 | 0.9223 | 39.440 | 0.992 | 561.32 | 0 | 2 | 5/18 | 7127 |
| 1479 | R.VVIPIELR.R |  | 1 | 0.2281 | 24.620 | 0.000 | 469.81 | 0 | 2 | 6/14 | 7128 |
| 1480 | R.DLELDSLTVVNHK.N |  | 1 | 0.2906 | 43.850 | 0.000 | 741.89 | 0 | 2 | 9/24 | 7130 |
| 1481 | R.SEELIGEVLR.E |  | 1 | 0.2746 | 41.270 | 0.000 | 572.81 | 1 | 2 | 7/18 | 7136 |
| 1482 | K.DLHELIFEK.G |  | 1 | 0.3419 | 24.800 | 0.000 | 381.87 | 0 | 3 | 6/16 | 7138 |
| 1483 | R.SKEYEAVHQK.A |  | 1 | 0.8020 | 23.620 | 0.871 | 406.88 | 0 | 3 | 12/18 | 713 |
| 1484 | K.IVNATGPWVDQLR.E |  | 1 | 0.2914 | 56.800 | 0.000 | 734.90 | 0 | 2 | 9/24 | 7140 |
| 1485 | R.EIWYLCR.Q |  | 1 | 0.5745 | 20.770 | 0.000 | 520.26 | 1 | 2 | 4/12 | 7143 |
| 1486 | K.EDHVYVNSVDPLAQGGTAWSK.V |  | 1 | 0.3528 | 36.520 | 0.000 | 758.37 | 0 | 3 | 19/40 | 7144 |
| 1487 | K.VETLDYLPGDEAGIK.S |  | 1 | 0.1441 | 47.690 | 0.000 | 810.41 | 0 | 2 | 8/28 | 7152 |
| 1488 | K.TFDELFEK.T |  | 1 | 0.4576 | 33.060 | 0.000 | 514.75 | 0 | 2 | 5/14 | 7153 |
| 1489 | R.YEDQPISDLASPIAASSR.Y |  | 2 | 0.8381 | 98.980 | 0.962 | 960.47 | 0 | 2 | 11/34 | 7156 |
| 1490 | R.TGIDCLAPALGSVHGPYK.G |  | 1 | 0.2285 | 34.770 | 0.000 | 619.32 | 0 | 3 | 15/34 | 7163 |
| 1491 | K.DAIIVSGDSDQSPWVK.K |  | 1 | 0.3041 | 50.790 | 0.000 | 858.93 | 0 | 2 | 11/30 | 7164 |
| 1492 | K.DGDVLVLENVR.F |  | 1 | 0.3952 | 37.910 | 0.000 | 614.83 | 0 | 2 | 13/20 | 7168 |
| 1493 | K.LVTEALDNNPQSIEDFK.N |  | 1 | 0.3245 | 86.360 | 0.000 | 966.98 | 1 | 2 | 10/32 | 7173 |
| 1494 | K.SWADSHQIEYITSDYR.D |  | 1 | 0.3564 | 20.350 | 0.000 | 657.63 | 0 | 3 | 13/30 | 7177 |
| 1495 | R.RVDELAEALNLR.G |  | 1 | 0.8292 | 48.190 | 0.858 | 466.93 | 0 | 3 | 6/22 | 7184 |
| 1496 | K.EITLLGQNVNAYGK.D |  | 1 | 0.6561 | 75.720 | 0.000 | 760.41 | 0 | 2 | 20/26 | 7190 |
| 1497 | R.ANHSEYGLAAGLWTENVK.Q |  | 2 | 0.7543 | 90.890 | 0.000 | 980.48 | 0 | 2 | 28/34 | 7199 |
| 1498 | R.ADILDPALLRPGR.F |  | 2 | 0.0412 | 24.290 | 0.000 | 469.61 | 1 | 3 | 5/24 | 7201 |
| 1499 | R.LVEIIELK.D |  | 1 | 0.5555 | 29.730 | 0.000 | 478.81 | 0 | 2 | 12/14 | 7204 |
| 1500 | K.FVEVLEESGLPK.G |  | 1 | 0.9177 | 67.910 | 0.786 | 673.86 | 0 | 2 | 10/22 | 7208 |
| 1501 | K.QLLQYDDVLR.Q |  | 1 | 0.4843 | 50.070 | 0.000 | 631.84 | 1 | 2 | 6/18 | 7209 |
| 1502 | K.EFAVIGLGR.F |  | 1 | 0.8361 | 48.720 | 0.680 | 481.28 | 0 | 2 | 8/16 | 7210 |
| 1503 | R.SVAGDVVSSTFDEVPENHIK.V |  | 1 | 0.3583 | 44.470 | 0.000 | 710.68 | 0 | 3 | 19/38 | 7212 |
| 1504 | K.KTRPQGSGQQLTAK.K |  | 1 | 0.4808 | 53.220 | 0.853 | 500.61 | 0 | 3 | 11/26 | 721 |
| 1505 | R.VTLYDVNFDSAK.I |  | 1 | 0.2572 | 42.090 | 0.000 | 686.34 | 0 | 2 | 8/22 | 7224 |
| 1506 | K.GYLAPLVLNR.V |  | 2 | 0.3057 | 40.520 | 0.000 | 558.33 | 0 | 2 | 7/18 | 7226 |
| 1507 | R.FWLSQDKEELLK.V |  | 1 | 0.7224 | 29.510 | 0.782 | 512.61 | 0 | 3 | 7/22 | 7227 |
| 1508 | R.IANFETAEPLYYR.A |  | 1 | 0.5683 | 77.440 | 0.000 | 793.90 | 0 | 2 | 10/24 | 7228 |
| 1509 | K.IADVVDKYEIPLVK.M |  | 1 | 0.8445 | 35.460 | 0.830 | 534.64 | 1 | 3 | 12/26 | 7229 |
| 1510 | K.SAEEALEYGLIDK.I |  | 2 | 0.9747 | 79.390 | 0.957 | 719.36 | 0 | 2 | 17/24 | 7232 |
| 1511 | R.VEFFGDEIER.I |  | 1 | 0.3935 | 41.010 | 0.000 | 620.80 | 0 | 2 | 8/18 | 7234 |
| 1512 | R.FDPFDVTK.V |  | 1 | 0.8326 | 21.500 | 0.950 | 484.74 | 0 | 2 | 5/14 | 7236 |
| 1513 | R.ADLDAQLVADNIAR.Q |  | 1 | 0.8683 | 74.950 | 0.961 | 495.60 | 0 | 3 | 9/26 | 7237 |
| 1514 | R.GGIAGITELNLNEK.E |  | 1 | 0.1514 | 58.600 | 0.000 | 714.89 | 1 | 2 | 10/26 | 7241 |
| 1515 | K.NAIIATGSRPIELPNFK.Y |  | 2 | 0.1969 | 72.750 | 0.000 | 921.02 | 0 | 2 | 13/32 | 7242 |
| 1516 | K.GIVLNEPSVVALDK.N |  | 2 | 0.1790 | 64.560 | 0.000 | 727.42 | 1 | 2 | 9/26 | 7245 |
| 1517 | K.YEGTEYLILR.E |  | 1 | 0.7727 | 54.040 | 0.000 | 628.83 | 1 | 2 | 14/18 | 7246 |
| 1518 | K.GADQIFNALK.K |  | 1 | 0.8012 | 46.310 | 0.786 | 538.79 | 1 | 2 | 6/18 | 7247 |
| 1519 | R.DAFGFEGTPIK.I |  | 1 | 0.3921 | 55.620 | 0.000 | 591.30 | 1 | 2 | 8/20 | 7249 |
| 1520 | K.GIILNEPSVVAVDTTTK.A |  | 1 | 0.2583 | 50.140 | 0.000 | 878.99 | 1 | 2 | 17/32 | 7251 |
| 1521 | K.IGVVFQDFK.L |  | 1 | 0.5560 | 40.090 | 0.000 | 526.79 | 0 | 2 | 7/16 | 7254 |
| 1522 | R.YLDLIVNPDSK.H |  | 1 | 0.5663 | 48.690 | 0.000 | 638.84 | 0 | 2 | 13/20 | 7258 |
| 1523 | R.AIRDEFEPIGLNTLNNNGEK.A |  | 1 | 0.3505 | 44.470 | 0.000 | 748.71 | 0 | 3 | 21/38 | 7260 |
| 1524 | K.NSSVDAALELVK.S |  | 1 | 0.8736 | 51.860 | 0.871 | 623.34 | 0 | 2 | 10/22 | 7261 |
| 1525 | K.TDYPETDDLFYEGK.E |  | 1 | 0.4962 | 69.850 | 0.000 | 846.87 | 0 | 2 | 8/26 | 7265 |
| 1526 | R.ELSLLLR.R |  | 1 | 0.4658 | 27.120 | 0.000 | 422.27 | 0 | 2 | 5/12 | 7267 |
| 1527 | K.NDAYKQEELDQIVDDVK.N |  | 1 | 0.4380 | 43.810 | 0.000 | 674.66 | 1 | 3 | 16/32 | 7270 |
| 1528 | K.LGFPVPIR.H |  | 1 | 0.3313 | 36.260 | 0.000 | 449.78 | 0 | 2 | 5/14 | 7275 |
| 1529 | R.DAIKFPDFIHTQK.R |  | 2 | 0.2963 | 36.860 | 0.000 | 520.61 | 1 | 3 | 11/24 | 7284 |
| 1530 | K.NQGGDSSIVDDPSKLPQAAYQIDVPAK.E |  | 2 | 0.1967 | 55.050 | 0.000 | 938.47 | 1 | 3 | 22/52 | 7289 |
| 1531 | K.WILDNVEGAR.E |  | 1 | 0.2228 | 25.820 | 0.000 | 586.81 | 1 | 2 | 7/18 | 7290 |
| 1532 | K.FSDSVLLTDETIK.E |  | 1 | 0.2646 | 67.770 | 0.000 | 734.38 | 0 | 2 | 8/24 | 7291 |
| 1533 | K.QEELDQIVDDVK.N |  | 1 | 0.4171 | 52.170 | 0.000 | 715.85 | 0 | 2 | 9/22 | 7293 |
| 1534 | K.SIAEYAIECSLNK.V |  | 2 | 0.9244 | 61.610 | 0.834 | 499.91 | 0 | 3 | 10/24 | 7296 |
| 1535 | K.VQDYISLIDK.I |  | 1 | 0.9357 | 50.490 | 0.927 | 597.32 | 0 | 2 | 8/18 | 7297 |
| 1536 | R.GAAEIQAIGAGALNQAVK.A |  | 1 | 0.8157 | 76.240 | 0.946 | 841.47 | 0 | 2 | 20/34 | 7303 |
| 1537 | K.ATLGGVCLNVGCIPSK.A |  | 2 | 0.0915 | 43.480 | 0.000 | 823.42 | 3 | 2 | 9/30 | 7304 |
| 1538 | R.ILEIEHLLK.R |  | 1 | 0.8898 | 29.610 | 0.680 | 369.90 | 0 | 3 | 9/16 | 7307 |
| 1539 | R.AAVEEGIVSGGGTALVNVYNK.V |  | 2 | 0.5135 | 86.500 | 0.877 | 1024.54 | 0 | 2 | 10/40 | 7317 |
| 1540 | K.SAEHNVSLQTALAVIK.A |  | 1 | 0.7398 | 28.040 | 0.955 | 560.98 | 0 | 3 | 9/30 | 7322 |
| 1541 | K.LAVLNYLK.T |  | 1 | 0.8801 | 40.000 | 0.739 | 467.29 | 0 | 2 | 7/14 | 7324 |
| 1542 | K.DYKPILATVIAR.S |  | 1 | 0.0763 | 23.710 | 0.000 | 453.94 | 0 | 3 | 9/22 | 7330 |
| 1543 | R.EDGDELPPGVNQLVR.V |  | 1 | 0.1847 | 45.910 | 0.000 | 819.41 | 0 | 2 | 14/28 | 7331 |
| 1544 | K.VEELLYDAEENADKYR.F |  | 2 | 0.3884 | 109.670 | 0.000 | 978.96 | 1 | 2 | 11/30 | 7337 |
| 1545 | R.TPEGLLDFR.R |  | 1 | 0.9885 | 51.230 | 0.953 | 524.28 | 0 | 2 | 13/16 | 7338 |
| 1546 | K.LTLALLR.E |  | 1 | 0.2674 | 24.850 | 0.000 | 400.27 | 0 | 2 | 5/12 | 7339 |
| 1547 | R.VIAIEDGIIVR.D |  | 1 | 0.9682 | 68.330 | 0.792 | 599.36 | 0 | 2 | 17/20 | 7341 |
| 1548 | K.ITGQTIPVSGAYFNYTR.H |  | 2 | 0.1507 | 45.910 | 0.000 | 944.48 | 0 | 2 | 8/32 | 7343 |
| 1549 | K.AAAPSWSGLVSGTVQR.M |  | 1 | 0.1819 | 82.150 | 0.000 | 793.92 | 2 | 2 | 11/30 | 7348 |
| 1550 | K.GVADNQAEIIACVGNFHGR.T |  | 1 | 0.3335 | 52.060 | 0.000 | 676.66 | 0 | 3 | 13/36 | 7354 |
| 1551 | R.TAQYADVVLPATPSLEK.D |  | 1 | 0.8704 | 49.810 | 0.980 | 901.98 | 0 | 2 | 14/32 | 7357 |
| 1552 | R.TPELLPVLK.E |  | 1 | 0.2938 | 28.020 | 0.000 | 505.32 | 0 | 2 | 9/16 | 7359 |
| 1553 | R.IDEFNSPGFITGKPLVLGGSHGR.E |  | 2 | 0.0876 | 65.110 | 0.000 | 800.09 | 0 | 3 | 12/44 | 7365 |
| 1554 | R.GLGLSDIGLER.D |  | 1 | 0.1941 | 63.720 | 0.000 | 565.31 | 0 | 2 | 7/20 | 7367 |
| 1555 | K.TAGASVLTQLVQEK.V |  | 1 | 0.2021 | 70.160 | 0.000 | 722.90 | 0 | 2 | 10/26 | 7374 |
| 1556 | R.TDNADNLIDLYK.Q |  | 1 | 0.5670 | 91.880 | 0.000 | 697.84 | 0 | 2 | 9/22 | 7376 |
| 1557 | K.IDAVFDALQDK.G |  | 1 | 0.9279 | 59.420 | 0.840 | 617.82 | 1 | 2 | 9/20 | 7377 |
| 1558 | K.GIIYIDEIDK.V |  | 1 | 0.8608 | 36.500 | 0.848 | 589.82 | 0 | 2 | 5/18 | 7382 |
| 1559 | M.ALTAGIVGLPNVGK.S |  | 1 | 0.1131 | 57.740 | 0.000 | 655.40 | 0 | 2 | 8/26 | 7386 |
| 1560 | K.LEQLSVFGNDYPTK.D |  | 1 | 0.2419 | 43.170 | 0.000 | 805.91 | 0 | 2 | 6/26 | 7390 |
| 1561 | R.ASFYLYNTEEEIDK.L |  | 1 | 0.4095 | 40.470 | 0.000 | 861.40 | 0 | 2 | 11/26 | 7391 |
| 1562 | K.ADVQGSAEALTAALQK.I |  | 2 | 0.3039 | 77.880 | 0.000 | 786.92 | 0 | 2 | 13/30 | 7392 |
| 1563 | R.VESPDQLADVLR.Q |  | 1 | 0.4274 | 62.730 | 0.000 | 671.35 | 0 | 2 | 10/22 | 7396 |
| 1564 | K.DGDLWASIK.K |  | 1 | 0.4202 | 45.460 | 0.000 | 502.76 | 0 | 2 | 7/16 | 7408 |
| 1565 | R.FGFIETPYR.R |  | 1 | 0.4845 | 25.150 | 0.000 | 565.29 | 1 | 2 | 12/16 | 7411 |
| 1566 | R.GPTLIQDVHLLEK.L |  | 2 | 0.3355 | 58.610 | 0.000 | 731.92 | 0 | 2 | 9/24 | 7416 |
| 1567 | K.VAEAAICYTGDILDK.N |  | 1 | 0.9169 | 42.790 | 0.957 | 819.90 | 1 | 2 | 20/28 | 7421 |
| 1568 | K.IALFAAGLAAQK.Y |  | 1 | 0.0935 | 39.510 | 0.000 | 587.35 | 1 | 2 | 9/22 | 7427 |
| 1569 | R.FGAGNILLKPASEGTGVIAGGPVR.A |  | 2 | 0.0105 | 54.230 | 0.000 | 1141.14 | 0 | 2 | 14/46 | 7430 |
| 1570 | K.LAGLDTVPAIVR.E |  | 1 | 0.6822 | 51.960 | 0.754 | 612.87 | 0 | 2 | 10/22 | 7434 |
| 1571 | K.IALFCDIDTK.A |  | 1 | 0.3919 | 37.710 | 0.000 | 598.30 | 0 | 2 | 7/18 | 7440 |
| 1572 | K.GTPNLDWEGLAR.S |  | 1 | 0.3191 | 56.200 | 0.000 | 664.83 | 1 | 2 | 7/22 | 7444 |
| 1573 | K.DFEDVFEK.Y |  | 1 | 0.5912 | 25.240 | 0.000 | 514.73 | 0 | 2 | 8/14 | 7445 |
| 1574 | R.EVQAFLQDLK.G |  | 1 | 0.8999 | 52.320 | 0.715 | 595.82 | 0 | 2 | 7/18 | 7449 |
| 1575 | K.AYDVSEAVALVK.K |  | 1 | 0.6766 | 89.260 | 0.000 | 632.84 | 0 | 2 | 20/22 | 7450 |
| 1576 | R.DTVSTIVEAVK.S |  | 1 | 0.9565 | 56.630 | 0.915 | 581.32 | 0 | 2 | 14/20 | 7451 |
| 1577 | R.GSNLLVLDEGIR.G |  | 1 | 0.1771 | 51.100 | 0.000 | 643.36 | 1 | 2 | 7/22 | 7454 |
| 1578 | R.VQGFEAGTDDYIVKPFSPR.E |  | 1 | 0.2523 | 34.330 | 0.000 | 709.36 | 0 | 3 | 15/36 | 7462 |
| 1579 | K.EAAVPQEIIEHFK.S |  | 1 | 0.3556 | 53.270 | 0.000 | 755.90 | 0 | 2 | 10/24 | 7463 |
| 1580 | K.IAFLQLR.D |  | 1 | 0.9425 | 27.140 | 0.909 | 430.77 | 0 | 2 | 10/12 | 7465 |
| 1581 | K.ITQQEAATAFGNPGVYIEK.Y |  | 2 | 0.2035 | 64.530 | 0.000 | 1019.02 | 0 | 2 | 13/36 | 7467 |
| 1582 | K.NGFEVVAEAENGAQAVEK.Y |  | 2 | 0.9388 | 108.430 | 0.950 | 931.45 | 0 | 2 | 24/34 | 7471 |
| 1583 | R.EEECFTFEQITAQPK.T |  | 1 | 0.4646 | 48.290 | 0.000 | 928.92 | 1 | 2 | 9/28 | 7476 |
| 1584 | R.ILLNEVFNK.E |  | 1 | 0.6117 | 44.860 | 0.000 | 545.32 | 0 | 2 | 7/16 | 7479 |
| 1585 | K.GIPLTTSIAALNR.D |  | 2 | 0.9486 | 69.630 | 0.971 | 663.89 | 0 | 2 | 17/24 | 7483 |
| 1586 | R.DINDFLDTK.K |  | 1 | 0.7816 | 46.750 | 0.000 | 540.76 | 0 | 2 | 11/16 | 7490 |
| 1587 | R.DGGDCELVDVDEGIVK.L |  | 1 | 0.2601 | 63.120 | 0.000 | 860.39 | 1 | 2 | 11/30 | 7491 |
| 1588 | R.IEYAIDFVK.A |  | 1 | 0.3775 | 29.140 | 0.000 | 549.30 | 0 | 2 | 5/16 | 7502 |
| 1589 | R.VVDLAAYIAK.K |  | 3 | 0.5999 | 61.850 | 0.000 | 531.81 | 0 | 2 | 15/18 | 7505 |
| 1590 | K.DGFLYLGK.A |  | 1 | 0.2342 | 32.090 | 0.000 | 456.74 | 0 | 2 | 5/14 | 7509 |
| 1591 | K.GISDFNSILK.D |  | 1 | 0.3930 | 38.950 | 0.000 | 547.30 | 1 | 2 | 11/18 | 7510 |
| 1592 | R.FTQAGSEVSALLGR.M |  | 2 | 0.3981 | 74.310 | 0.000 | 718.38 | 1 | 2 | 10/26 | 7515 |
| 1593 | K.VAEYGTSFGAPTEVENELAK.L |  | 2 | 0.0921 | 36.300 | 0.000 | 1056.51 | 0 | 2 | 12/38 | 7528 |
| 1594 | K.DYADFLHEDLK.I |  | 2 | 0.9795 | 40.600 | 0.979 | 683.32 | 0 | 2 | 7/20 | 7529 |
| 1595 | K.EEGDKEIVTIEQPGSIVLQAR.F |  | 1 | 0.6826 | 51.600 | 0.966 | 771.08 | 0 | 3 | 9/40 | 7532 |
| 1596 | K.FAIEDPDNEVNFPR.N |  | 1 | 0.4808 | 79.370 | 0.000 | 831.89 | 0 | 2 | 9/26 | 7533 |
| 1597 | K.THIENVYEFTDELAK.Q |  | 1 | 0.5964 | 32.150 | 0.000 | 603.63 | 1 | 3 | 13/28 | 7542 |
| 1598 | K.ADELNLQILK.L |  | 1 | 0.7640 | 58.800 | 0.000 | 578.83 | 3 | 2 | 15/18 | 7543 |
| 1599 | K.SSDLGDLIGVTGK.V |  | 1 | 0.7407 | 82.840 | 0.845 | 631.34 | 0 | 2 | 10/24 | 7544 |
| 1600 | K.AGLVIIVGANPAEGHPVLATR.V |  | 1 | 0.0300 | 20.370 | 0.000 | 685.73 | 0 | 3 | 6/40 | 7549 |
| 1601 | K.FDFTPVGEPANLTK.M |  | 1 | 0.2492 | 55.990 | 0.000 | 768.39 | 0 | 2 | 9/26 | 7558 |
| 1602 | R.TIASFFGTNDVTK.W |  | 1 | 0.2148 | 63.060 | 0.000 | 700.86 | 1 | 2 | 11/24 | 7561 |
| 1603 | R.IDEIIVFHSLEK.K |  | 1 | 0.3926 | 35.230 | 0.000 | 481.60 | 0 | 3 | 8/22 | 7565 |
| 1604 | K.AFNVTLENNTALLK.G |  | 1 | 0.3511 | 37.980 | 0.000 | 774.42 | 0 | 2 | 9/26 | 7568 |
| 1605 | K.INPESPNIQAIVIAPTR.E |  | 1 | 0.3032 | 76.110 | 0.000 | 917.02 | 0 | 2 | 13/32 | 7574 |
| 1606 | K.LVNAGAVHLEPFGIYSK.T |  | 1 | 0.7498 | 29.440 | 0.873 | 605.66 | 1 | 3 | 12/32 | 7579 |
| 1607 | R.LLEDGNTVPFIAR.Y |  | 1 | 0.8509 | 46.590 | 0.897 | 722.89 | 2 | 2 | 9/24 | 7580 |
| 1608 | R.LEVLEAFR.N |  | 1 | 0.9522 | 42.070 | 0.703 | 488.78 | 0 | 2 | 6/14 | 7584 |
| 1609 | K.VIEVAPSVSLSPELR.D |  | 1 | 0.8210 | 87.230 | 0.999 | 798.45 | 0 | 2 | 10/28 | 7587 |
| 1610 | R.VYDFLAGVK.K |  | 1 | 0.5084 | 35.550 | 0.000 | 506.28 | 0 | 2 | 11/16 | 7588 |
| 1611 | K.NPDKAEEAAWDAYQYYK.D |  | 1 | 0.5057 | 44.550 | 0.000 | 687.98 | 0 | 3 | 17/32 | 7589 |
| 1612 | R.YPVEEGDKDNIIGVINIK.E |  | 1 | 0.8571 | 41.780 | 0.749 | 672.69 | 0 | 3 | 17/34 | 7590 |
| 1613 | K.VELLLLK.Q |  | 1 | 0.9297 | 44.140 | 0.754 | 414.28 | 0 | 2 | 6/12 | 7595 |
| 1614 | K.AGSGVATLGLPDSPGVPEGIAK.N |  | 2 | 0.0540 | 69.420 | 0.000 | 997.03 | 0 | 2 | 19/42 | 7600 |
| 1615 | M.PSVESFELDHNAVVAPYVR.H |  | 1 | 0.5811 | 38.050 | 0.000 | 710.36 | 0 | 3 | 16/36 | 7602 |
| 1616 | K.GILGYSEEPLVSGDYNGNK.N |  | 3 | 0.4752 | 86.550 | 0.000 | 1006.48 | 0 | 2 | 23/36 | 7604 |
| 1617 | R.VANISEFLDDK.M |  | 1 | 0.5333 | 67.710 | 0.000 | 625.82 | 0 | 2 | 7/20 | 7609 |
| 1618 | K.VIFEGDALAICR.A |  | 1 | 0.8706 | 54.200 | 0.849 | 682.36 | 0 | 2 | 8/22 | 7612 |
| 1619 | R.IDYLTADEEDNYVVAQANAR.L |  | 1 | 0.7015 | 48.010 | 0.000 | 757.36 | 1 | 3 | 21/38 | 7614 |
| 1620 | R.HTPFFSNYRPQFYFR.T |  | 1 | 0.3557 | 20.670 | 0.000 | 502.50 | 0 | 4 | 12/28 | 7621 |
| 1621 | R.VISEGILQGFR.N |  | 1 | 0.4299 | 82.770 | 0.000 | 609.85 | 0 | 2 | 8/20 | 7624 |
| 1622 | K.GYASFDYELIGYKPSK.L |  | 1 | 0.6960 | 20.460 | 0.920 | 613.31 | 0 | 3 | 9/30 | 7630 |
| 1623 | K.EAVDTLIVIPNDR.I |  | 1 | 0.2282 | 36.310 | 0.000 | 727.90 | 0 | 2 | 11/24 | 7631 |
| 1624 | R.HGLGIGGSGEPWFAVGK.D |  | 1 | 0.1187 | 22.780 | 0.000 | 556.95 | 1 | 3 | 14/32 | 7632 |
| 1625 | K.EIADIVGVQLGK.C |  | 2 | 0.1853 | 72.780 | 0.000 | 621.36 | 0 | 2 | 7/22 | 7642 |
| 1626 | R.TSAVDFGNIDIVK.Y |  | 1 | 0.9317 | 59.570 | 0.949 | 689.86 | 0 | 2 | 10/24 | 7644 |
| 1627 | R.IADELGPIFR.F |  | 1 | 0.9242 | 53.650 | 0.909 | 565.81 | 0 | 2 | 9/18 | 7659 |
| 1628 | R.KSVTLVHK.G |  | 1 | 0.1550 | 20.260 | 0.000 | 304.53 | 0 | 3 | 8/14 | 765 |
| 1629 | K.VFSFGPTFR.A |  | 1 | 0.9055 | 39.600 | 0.883 | 529.28 | 0 | 2 | 8/16 | 7664 |
| 1630 | K.SYDFAQSVATDEVLK.S |  | 1 | 0.2878 | 92.950 | 0.000 | 836.91 | 1 | 2 | 11/28 | 7667 |
| 1631 | R.NLFVWR.A |  | 1 | 0.9764 | 35.000 | 0.698 | 417.73 | 0 | 2 | 8/10 | 7669 |
| 1632 | K.AIDALIPIGR.G |  | 1 | 0.4295 | 38.130 | 0.000 | 519.82 | 0 | 2 | 12/18 | 7675 |
| 1633 | R.VPEVLEFYQR.K |  | 1 | 0.6661 | 30.780 | 0.000 | 640.34 | 1 | 2 | 8/18 | 7678 |
| 1634 | K.GIDLSQVPTGDPLGR.V |  | 1 | 0.2678 | 39.910 | 0.000 | 762.90 | 0 | 2 | 13/28 | 7684 |
| 1635 | K.DGKPWYFLEEK.Y |  | 1 | 0.7552 | 27.010 | 0.841 | 471.23 | 0 | 3 | 6/20 | 7686 |
| 1636 | R.LFYLAEAYR.R |  | 1 | 0.9390 | 30.990 | 0.858 | 573.30 | 0 | 2 | 7/16 | 7687 |
| 1637 | K.NQTFELTVFQK.G |  | 1 | 0.6343 | 49.630 | 0.000 | 677.85 | 0 | 2 | 7/20 | 7694 |
| 1638 | K.YTGLEGDVLGIDR.F |  | 1 | 0.3880 | 74.700 | 0.000 | 704.36 | 0 | 2 | 11/24 | 7695 |
| 1639 | R.SLELGEYFK.S |  | 1 | 0.3400 | 25.070 | 0.000 | 543.28 | 0 | 2 | 5/16 | 7696 |
| 1640 | K.QFETFQILNEK.G |  | 1 | 0.5261 | 46.870 | 0.000 | 698.86 | 1 | 2 | 7/20 | 7713 |
| 1641 | K.SSFINSLINR.K |  | 1 | 0.5953 | 56.060 | 0.000 | 575.81 | 0 | 2 | 8/18 | 7714 |
| 1642 | K.AVITVPAYFNDAER.Q |  | 2 | 0.6988 | 56.770 | 0.000 | 522.60 | 0 | 3 | 19/26 | 7715 |
| 1643 | K.DQENPLNDAFVVK.T |  | 1 | 0.3916 | 64.270 | 0.000 | 744.87 | 0 | 2 | 10/24 | 7716 |
| 1644 | K.LYYDGANLNAVLSK.A |  | 1 | 0.4010 | 62.890 | 0.000 | 770.90 | 0 | 2 | 12/26 | 7725 |
| 1645 | K.LYPNGGVVEDEVTLQSIK.I |  | 2 | 0.2638 | 87.710 | 0.000 | 981.01 | 0 | 2 | 11/34 | 7732 |
| 1646 | K.YLEGEEITIDELK.A |  | 2 | 0.9661 | 85.410 | 0.990 | 776.39 | 0 | 2 | 9/24 | 7740 |
| 1647 | R.LVPGYEAPCYVAWSAQNR.S |  | 2 | 0.4254 | 50.430 | 0.000 | 1041.00 | 0 | 2 | 13/34 | 7742 |
| 1648 | R.LAETLVDPETGEILAEK.G |  | 2 | 0.4853 | 55.430 | 0.000 | 914.48 | 0 | 2 | 24/32 | 7743 |
| 1649 | K.LWDGQCYVFDER.I |  | 1 | 0.7091 | 58.310 | 0.000 | 794.35 | 0 | 2 | 8/22 | 7757 |
| 1650 | K.VGLDYLTLSR.A |  | 1 | 0.8707 | 43.580 | 0.896 | 568.82 | 1 | 2 | 8/18 | 7758 |
| 1651 | R.FTLVHNGVIENYVQLK.Q |  | 1 | 0.4068 | 27.260 | 0.000 | 625.34 | 1 | 3 | 13/30 | 7764 |
| 1652 | R.KDEIWTSDSGLITIAGGK.L |  | 2 | 0.1033 | 106.620 | 0.000 | 945.99 | 0 | 2 | 13/34 | 7768 |
| 1653 | R.DIFEAYQGEAPYDR.F |  | 1 | 0.9784 | 56.100 | 0.881 | 837.38 | 0 | 2 | 18/26 | 7771 |
| 1654 | K.LQEAFEEYCEEV.- |  | 1 | 0.6835 | 69.500 | 0.000 | 773.32 | 0 | 2 | 9/22 | 7774 |
| 1655 | R.IEDQLAETAQYHGINSFYNLNK.- |  | 2 | 0.9259 | 42.790 | 0.926 | 856.75 | 0 | 3 | 10/42 | 7775 |
| 1656 | R.TGIVQVVFNPDVSK.E |  | 1 | 0.0961 | 23.540 | 0.000 | 751.91 | 0 | 2 | 9/26 | 7780 |
| 1657 | R.IANLFTDVPHLR.K |  | 1 | 0.3970 | 44.630 | 0.000 | 465.93 | 1 | 3 | 7/22 | 7789 |
| 1658 | R.IELTELAEDEVR.V |  | 1 | 0.1679 | 28.810 | 0.000 | 708.86 | 2 | 2 | 9/22 | 7790 |
| 1659 | R.YLGIHIASPVFDGAR.E |  | 1 | 0.2739 | 34.880 | 0.000 | 539.29 | 0 | 3 | 15/28 | 7794 |
| 1660 | K.EGFEAEFVHDGLEGYQR.F |  | 1 | 0.4361 | 26.360 | 0.000 | 661.63 | 0 | 3 | 14/32 | 7800 |
| 1661 | R.IINEPTAAALAYGLDK.T |  | 2 | 0.3374 | 86.140 | 0.000 | 830.45 | 1 | 2 | 13/30 | 7813 |
| 1662 | R.FVLVLVR.G |  | 1 | 0.5869 | 41.750 | 0.000 | 423.28 | 0 | 2 | 6/12 | 7814 |
| 1663 | K.GFTNQEIADALHLSK.R |  | 1 | 0.1190 | 25.180 | 0.000 | 548.62 | 0 | 3 | 9/28 | 7820 |
| 1664 | R.NELAVTTLFK.I |  | 1 | 0.9484 | 47.180 | 0.945 | 568.32 | 0 | 2 | 9/18 | 7837 |
| 1665 | K.CAHCYLESSPEALGTVSIEQFK.K |  | 2 | 0.8810 | 52.920 | 0.960 | 1263.59 | 1 | 2 | 11/42 | 7838 |
| 1666 | R.GAAGGEEAALFAGNLYR.M |  | 1 | 0.1302 | 92.640 | 0.000 | 833.91 | 0 | 2 | 13/32 | 7840 |
| 1667 | R.SGETEDSTIADIAVATNAGQIK.T |  | 2 | 0.2304 | 65.300 | 0.000 | 731.03 | 0 | 3 | 18/42 | 7846 |
| 1668 | K.FAEFLSLK.G |  | 1 | 0.3323 | 26.830 | 0.000 | 477.77 | 0 | 2 | 9/14 | 7859 |
| 1669 | R.ILVDAPCSGFGVIR.R |  | 1 | 0.1850 | 51.510 | 0.000 | 752.40 | 0 | 2 | 9/26 | 7860 |
| 1670 | K.ILGLEEYLHR.K |  | 2 | 0.6153 | 63.260 | 0.000 | 621.85 | 0 | 2 | 8/18 | 7865 |
| 1671 | R.SIGVSNFSLEQLK.E |  | 1 | 0.3886 | 66.140 | 0.000 | 711.38 | 1 | 2 | 10/24 | 7871 |
| 1672 | K.LSGEALAGEQGNGINPTVIQSIAK.Q |  | 2 | 0.0356 | 53.180 | 0.000 | 1184.13 | 1 | 2 | 12/46 | 7876 |
| 1673 | K.TTLTAAITTVLHK.K |  | 11 | 0.2645 | 64.950 | 0.000 | 685.41 | 0 | 2 | 10/24 | 7880 |
| 1674 | K.NILSLALQR.C |  | 1 | 0.4186 | 38.920 | 0.000 | 514.32 | 1 | 2 | 7/16 | 7881 |
| 1675 | R.VNIIDTPGHVDFTVEVER.S |  | 2 | 0.3707 | 78.320 | 0.000 | 1020.52 | 0 | 2 | 13/34 | 7887 |
| 1676 | K.VQLVGDDLFVTNTK.K |  | 1 | 0.3790 | 85.730 | 0.000 | 774.92 | 0 | 2 | 10/26 | 7888 |
| 1677 | K.DDAPVTVVEFGDYK.C |  | 1 | 0.3289 | 69.000 | 0.000 | 777.87 | 0 | 2 | 14/26 | 7891 |
| 1678 | K.DHPWFVASQFHPEFK.S |  | 1 | 0.4209 | 24.670 | 0.000 | 624.63 | 0 | 3 | 8/28 | 7895 |
| 1679 | R.SHPLAATGLPLEGIALER.E |  | 1 | 0.2423 | 43.170 | 0.000 | 615.68 | 0 | 3 | 14/34 | 7897 |
| 1680 | R.SSLWAVQTPQAFR.L |  | 1 | 0.1546 | 30.820 | 0.000 | 745.89 | 5 | 2 | 8/24 | 7904 |
| 1681 | R.IIGLLEQEENIEPPR.L |  | 1 | 0.7183 | 70.840 | 0.000 | 875.47 | 2 | 2 | 20/28 | 7908 |
| 1682 | K.TLAGQLYSEFK.E |  | 1 | 0.9150 | 52.740 | 0.800 | 628.83 | 0 | 2 | 8/20 | 7912 |
| 1683 | R.QGTPLVEIVSEPDIR.T |  | 2 | 0.3801 | 77.810 | 0.000 | 826.95 | 1 | 2 | 10/28 | 7915 |
| 1684 | K.TLDGWYALHDFR.T |  | 1 | 0.5787 | 25.620 | 0.000 | 498.58 | 0 | 3 | 14/22 | 7917 |
| 1685 | K.ITNWNFTNFAK.H |  | 1 | 0.4751 | 48.290 | 0.000 | 678.34 | 0 | 2 | 7/20 | 7918 |
| 1686 | K.QGFEDVAQLDGGIVTYGKDPEVQGK.L |  | 1 | 0.2193 | 87.570 | 0.000 | 884.10 | 1 | 3 | 27/48 | 7922 |
| 1687 | R.FLALLPSLKPR.Y |  | 1 | 0.0767 | 28.230 | 0.000 | 418.94 | 0 | 3 | 6/20 | 7933 |
| 1688 | K.KPLFIFLSQSGETADSR.A |  | 2 | 0.1217 | 78.850 | 0.000 | 948.50 | 0 | 2 | 10/32 | 7935 |
| 1689 | K.ELDQLVDSFGDSGK.S |  | 1 | 0.1858 | 64.220 | 0.000 | 755.36 | 0 | 2 | 9/26 | 7943 |
| 1690 | R.LDDEGAFIDDSIVAR.F |  | 2 | 0.8339 | 72.300 | 0.875 | 818.40 | 1 | 2 | 10/28 | 7944 |
| 1691 | K.VSNLSQTITIGLVGK.Y |  | 1 | 0.2453 | 44.570 | 0.000 | 765.45 | 0 | 2 | 16/28 | 7945 |
| 1692 | R.LSPSSLGLEPWK.F |  | 1 | 0.7306 | 28.840 | 0.975 | 657.36 | 0 | 2 | 7/22 | 7951 |
| 1693 | R.IINVSSIVGVSGNPGQANYVAAK.A |  | 2 | 0.3125 | 56.820 | 0.000 | 753.41 | 0 | 3 | 23/44 | 7955 |
| 1694 | K.TVEQPFTILSAPHFK.A |  | 2 | 0.4179 | 84.890 | 0.000 | 857.96 | 0 | 2 | 11/28 | 7959 |
| 1695 | K.GLVQISDEGVLLK.L |  | 1 | 0.6202 | 32.350 | 0.786 | 685.90 | 1 | 2 | 9/24 | 7964 |
| 1696 | R.GNQIVGAVLFGDSSEGNR.L |  | 2 | 0.0822 | 69.700 | 0.000 | 910.45 | 0 | 2 | 9/34 | 7965 |
| 1697 | K.GGTFGSFTTSIGLR.V |  | 2 | 0.2188 | 79.740 | 0.000 | 700.86 | 0 | 2 | 9/26 | 7973 |
| 1698 | K.ESVLQHGILQPLIVR.K |  | 1 | 0.8908 | 32.870 | 0.923 | 568.01 | 0 | 3 | 12/28 | 7977 |
| 1699 | R.EADFINCVTWR.R |  | 1 | 0.5691 | 54.550 | 0.000 | 705.83 | 0 | 2 | 8/20 | 7979 |
| 1700 | R.FGSVLENVVVDEDTR.E |  | 1 | 0.4322 | 81.930 | 0.000 | 839.92 | 0 | 2 | 9/28 | 7980 |
| 1701 | R.IGFLFAPK.D |  | 1 | 0.9194 | 48.250 | 0.867 | 446.77 | 0 | 2 | 7/14 | 7981 |
| 1702 | K.AVSLVLPELK.G |  | 3 | 0.3134 | 60.020 | 0.000 | 534.84 | 0 | 2 | 8/18 | 7984 |
| 1703 | R.IQIFEGVVIK.R |  | 1 | 0.3539 | 47.980 | 0.000 | 573.35 | 0 | 2 | 7/18 | 7985 |
| 1704 | K.EIADFVTKPVDEDGIAYAVK.E |  | 1 | 0.1901 | 42.130 | 0.000 | 727.38 | 0 | 3 | 15/38 | 7986 |
| 1705 | R.VGDGPFPTELKDEIGDQIR.E |  | 1 | 0.4459 | 33.500 | 0.000 | 696.02 | 1 | 3 | 21/36 | 7997 |
| 1706 | R.ATYEFFNNEGFVK.V |  | 1 | 0.5163 | 60.200 | 0.000 | 783.37 | 0 | 2 | 9/24 | 8011 |
| 1707 | K.DIGGDAFSHVTISGGNPALLK.Q |  | 1 | 0.1189 | 34.890 | 0.000 | 690.36 | 1 | 3 | 14/40 | 8019 |
| 1708 | R.ELYVDEVVNEVK.K |  | 1 | 0.4337 | 63.310 | 0.000 | 718.37 | 0 | 2 | 9/22 | 8031 |
| 1709 | K.LAAAVLSINAFK.G |  | 1 | 0.3720 | 54.660 | 0.000 | 609.37 | 0 | 2 | 16/22 | 8037 |
| 1710 | K.IATVGDAVNYIQNQQ.- |  | 2 | 0.9632 | 71.270 | 0.981 | 817.41 | 0 | 2 | 15/28 | 8038 |
| 1711 | K.QVAILYALTK.G |  | 1 | 0.2121 | 28.620 | 0.000 | 560.34 | 0 | 2 | 8/18 | 8039 |
| 1712 | K.CADLLPDHFSFVK.G |  | 1 | 0.4239 | 25.360 | 0.000 | 516.92 | 0 | 3 | 13/24 | 8050 |
| 1713 | K.LLEIIPIK.E |  | 1 | 0.3114 | 26.350 | 0.000 | 469.82 | 0 | 2 | 9/14 | 8051 |
| 1714 | R.SPFGGGVHTPELHADSLEGLVAQQPGIK.V |  | 1 | 0.1159 | 22.780 | 0.000 | 711.12 | 0 | 4 | 14/54 | 8056 |
| 1715 | K.SDWDIFK.A |  | 1 | 0.7575 | 47.150 | 0.000 | 455.72 | 0 | 2 | 5/12 | 8057 |
| 1716 | R.TLNTADIVSEWSQK.I |  | 1 | 0.2722 | 53.230 | 0.000 | 796.40 | 1 | 2 | 10/26 | 8076 |
| 1717 | K.SFGLASNAATLISCPTCGR.I |  | 1 | 0.7212 | 85.460 | 0.991 | 991.98 | 1 | 2 | 10/36 | 8077 |
| 1718 | R.EFALGATHEEVITSLVR.D |  | 1 | 0.3091 | 33.990 | 0.000 | 624.67 | 0 | 3 | 14/32 | 8081 |
| 1719 | K.VETPLQAYFR.I |  | 1 | 0.8656 | 30.860 | 0.828 | 612.32 | 0 | 2 | 8/18 | 8082 |
| 1720 | K.STSDTIPYNEILNDNISLEHEAK.V |  | 1 | 0.3566 | 31.620 | 0.000 | 868.42 | 1 | 3 | 17/44 | 8083 |
| 1721 | K.VAYVKPAADLTDLNNVIVVNR.D |  | 1 | 0.1168 | 30.410 | 0.000 | 762.09 | 0 | 3 | 16/40 | 8084 |
| 1722 | R.EIAFAINIPK.E |  | 1 | 0.9292 | 47.140 | 0.932 | 558.33 | 1 | 2 | 7/18 | 8085 |
| 1723 | K.AIDENVTSYTPNAGYLELR.Q |  | 1 | 0.0503 | 24.850 | 0.000 | 1063.52 | 1 | 2 | 7/36 | 8087 |
| 1724 | R.TYVSIDEIPDVVK.E |  | 1 | 0.3541 | 40.820 | 0.000 | 739.39 | 0 | 2 | 7/24 | 8094 |
| 1725 | R.EIALLENLQR.E |  | 1 | 0.4861 | 55.930 | 0.000 | 599.84 | 0 | 2 | 9/18 | 8103 |
| 1726 | K.ALGEHLFEHFIEAK.E |  | 1 | 0.4243 | 28.800 | 0.000 | 547.62 | 0 | 3 | 10/26 | 8104 |
| 1727 | K.GGFFVFPK.S |  | 1 | 0.9030 | 46.360 | 0.861 | 449.74 | 1 | 2 | 6/14 | 8108 |
| 1728 | K.VNIVIIDDHQLFR.E |  | 1 | 0.9633 | 62.970 | 0.912 | 791.44 | 0 | 2 | 11/24 | 8111 |
| 1729 | K.VIILSIHDDENYVTHALK.T |  | 1 | 0.1663 | 21.570 | 0.000 | 694.04 | 0 | 3 | 7/34 | 8117 |
| 1730 | K.QVGEAVSILNLTPR.A |  | 2 | 0.3845 | 71.580 | 0.000 | 748.93 | 0 | 2 | 12/26 | 8118 |
| 1731 | K.IDALSFIVHR.D |  | 1 | 0.9563 | 50.620 | 0.989 | 390.89 | 0 | 3 | 11/18 | 8120 |
| 1732 | R.GVILTGGGALLNGIK.E |  | 1 | 0.0963 | 57.530 | 0.000 | 691.92 | 0 | 2 | 10/28 | 8122 |
| 1733 | R.AESGDLGILPGHIPTVAPLK.I |  | 1 | 0.1901 | 49.790 | 0.000 | 662.37 | 0 | 3 | 16/38 | 8125 |
| 1734 | K.GEDLTDFDKFEALDDR.R |  | 1 | 0.1843 | 44.240 | 0.000 | 629.29 | 0 | 3 | 9/30 | 8126 |
| 1735 | R.NGEAQFVINTLTK.G |  | 1 | 0.9179 | 57.190 | 0.948 | 717.88 | 0 | 2 | 11/24 | 8128 |
| 1736 | R.DLTTDLIINER.I |  | 1 | 0.9532 | 46.530 | 0.966 | 651.85 | 0 | 2 | 7/20 | 8129 |
| 1737 | R.TLEWTVSSPPPEYNFK.Q |  | 1 | 0.1525 | 31.040 | 0.000 | 947.96 | 0 | 2 | 9/30 | 8131 |
| 1738 | R.SWTDIVSAVK.E |  | 1 | 0.9426 | 54.140 | 0.996 | 553.30 | 0 | 2 | 7/18 | 8134 |
| 1739 | K.LDVIGNNIANVNTVGFK.K |  | 2 | 0.8542 | 85.010 | 0.984 | 894.49 | 1 | 2 | 9/32 | 8139 |
| 1740 | K.DAVESIFGVK.V |  | 1 | 0.3718 | 42.250 | 0.000 | 532.78 | 0 | 2 | 8/18 | 8142 |
| 1741 | K.LTELVNILTK.S |  | 1 | 0.4710 | 49.710 | 0.000 | 572.35 | 0 | 2 | 8/18 | 8145 |
| 1742 | K.AFGLETVSENELAHSEEELR.I |  | 1 | 0.5954 | 46.000 | 0.000 | 754.03 | 0 | 3 | 19/38 | 8154 |
| 1743 | K.VGLQIAEATPLIGTQVK.T |  | 2 | 0.1323 | 65.690 | 0.000 | 869.51 | 0 | 2 | 13/32 | 8156 |
| 1744 | K.TVDADYVLITVGR.R |  | 2 | 0.2650 | 71.340 | 0.000 | 711.38 | 1 | 2 | 10/24 | 8157 |
| 1745 | R.QSCTALIDQLEQPIR.L |  | 2 | 0.5005 | 62.220 | 0.000 | 886.45 | 0 | 2 | 11/28 | 8175 |
| 1746 | K.TIAIFINQIR.E |  | 3 | 0.5334 | 52.020 | 0.000 | 594.86 | 0 | 2 | 8/18 | 8186 |
| 1747 | K.HGIVPIICVGETLEER.E |  | 1 | 0.3602 | 54.110 | 0.000 | 607.99 | 0 | 3 | 13/30 | 8187 |
| 1748 | K.VLFEISGVSEEVAR.E |  | 2 | 0.2497 | 57.930 | 0.000 | 767.91 | 0 | 2 | 11/26 | 8194 |
| 1749 | K.GVVNFVPGSGAEVGDYLVDHPK.T |  | 1 | 0.0349 | 27.530 | 0.000 | 752.72 | 1 | 3 | 7/42 | 8197 |
| 1750 | K.TELINAVAEASELSK.K |  | 1 | 0.9087 | 43.840 | 0.869 | 525.61 | 1 | 3 | 17/28 | 8198 |
| 1751 | K.QTAGSDPAAVIYDAVHAAK.A |  | 1 | 0.1569 | 31.650 | 0.000 | 628.99 | 1 | 3 | 16/36 | 8200 |
| 1752 | K.LAVITEEELFK.N |  | 1 | 0.3646 | 39.270 | 0.000 | 646.36 | 0 | 2 | 9/20 | 8201 |
| 1753 | K.TLGEAVSFVEEVK.S |  | 2 | 0.2537 | 61.900 | 0.000 | 704.37 | 1 | 2 | 10/24 | 8203 |
| 1754 | K.LVENFTTM#YDTILK.A |  | 1 | 0.9349 | 99.560 | 0.904 | 852.43 | 0 | 2 | 10/26 | 8204 |
| 1755 | R.NTLDISAVPILK.Q |  | 1 | 0.2984 | 55.220 | 0.000 | 642.38 | 0 | 2 | 8/22 | 8217 |
| 1756 | R.TLLDVISGAK.T |  | 1 | 0.8488 | 43.810 | 0.692 | 508.80 | 0 | 2 | 11/18 | 8218 |
| 1757 | R.VVYDITSKPPATIEWE.- |  | 1 | 0.4353 | 42.360 | 0.000 | 924.47 | 0 | 2 | 16/30 | 8221 |
| 1758 | K.EFEVTEVGVFTPK.A |  | 1 | 0.3895 | 71.270 | 0.000 | 741.38 | 0 | 2 | 8/24 | 8229 |
| 1759 | K.AWESLENITNLK.L |  | 1 | 0.9429 | 58.890 | 0.759 | 709.37 | 0 | 2 | 16/22 | 8230 |
| 1760 | R.FICDIYNPDGTPFEGDPR.N |  | 1 | 0.4549 | 27.350 | 0.000 | 1056.97 | 1 | 2 | 22/34 | 8231 |
| 1761 | K.GGYFDLAPTDLGENCR.R |  | 1 | 0.4582 | 74.230 | 0.000 | 892.90 | 0 | 2 | 20/30 | 8238 |
| 1762 | K.SSPNSVAGALAGVLR.E |  | 1 | 0.6698 | 79.630 | 0.899 | 699.89 | 0 | 2 | 11/28 | 8239 |
| 1763 | K.LIPGLENVEIVR.Y |  | 1 | 0.4419 | 36.170 | 0.000 | 676.40 | 0 | 2 | 12/22 | 8240 |
| 1764 | K.TLEGSALSIFK.M |  | 1 | 0.3661 | 55.110 | 0.000 | 583.33 | 0 | 2 | 8/20 | 8242 |
| 1765 | K.GYEIPIVHVNADDPEACLSAVK.F |  | 1 | 0.9778 | 59.210 | 0.931 | 799.73 | 1 | 3 | 26/42 | 8244 |
| 1766 | K.GVNVPGVSVNLPGITEK.D |  | 1 | 0.2340 | 59.080 | 0.000 | 840.47 | 0 | 2 | 12/32 | 8247 |
| 1767 | K.SFNEQLGLTGVVLTK.L |  | 1 | 0.3886 | 78.450 | 0.000 | 803.45 | 0 | 2 | 12/28 | 8250 |
| 1768 | K.SITTGAAGSWSLSNLAPR.M |  | 2 | 0.2237 | 79.280 | 0.000 | 894.97 | 0 | 2 | 12/34 | 8251 |
| 1769 | R.KGTEIVVSNLFFNTPAR.L |  | 1 | 0.0429 | 21.060 | 0.000 | 631.68 | 0 | 3 | 7/32 | 8257 |
| 1770 | K.YFEAVYIPPSLK.D |  | 1 | 0.5137 | 42.220 | 0.000 | 713.88 | 0 | 2 | 13/22 | 8264 |
| 1771 | K.AAVIGGGLLGLEAAR.G |  | 1 | 0.1145 | 96.480 | 0.000 | 684.40 | 0 | 2 | 12/28 | 8272 |
| 1772 | K.NIILDPGIGFAK.T |  | 1 | 0.7893 | 28.360 | 0.923 | 629.36 | 0 | 2 | 8/22 | 8274 |
| 1773 | K.WLEDYVDLK.G |  | 1 | 0.4692 | 32.130 | 0.000 | 590.80 | 1 | 2 | 7/16 | 8278 |
| 1774 | R.RGDITIEEDLIEEAAR.L |  | 1 | 0.3359 | 30.150 | 0.000 | 610.65 | 0 | 3 | 19/30 | 8280 |
| 1775 | R.GDLGVEIPAEEVPLVQK.E |  | 1 | 0.8527 | 73.580 | 0.986 | 896.99 | 0 | 2 | 12/32 | 8285 |
| 1776 | K.ELEITNSSPTNIEVNALGINK.A |  | 1 | 0.1466 | 67.110 | 0.000 | 1128.59 | 0 | 2 | 14/40 | 8294 |
| 1777 | K.DDQSLGEFFR.R |  | 1 | 0.4394 | 53.530 | 0.000 | 607.28 | 0 | 2 | 7/18 | 8296 |
| 1778 | K.EATVLELNDLVK.A |  | 2 | 0.9123 | 53.160 | 0.954 | 448.59 | 1 | 3 | 12/22 | 8307 |
| 1779 | R.LPVEQYGVDAAILYK.D |  | 1 | 0.3812 | 75.440 | 0.000 | 839.96 | 0 | 2 | 9/28 | 8318 |
| 1780 | K.IDTGFFFK.E |  | 1 | 0.3571 | 39.500 | 0.000 | 487.75 | 0 | 2 | 6/14 | 8321 |
| 1781 | R.LDVINLISK.D |  | 1 | 0.2795 | 38.490 | 0.000 | 507.81 | 0 | 2 | 6/16 | 8322 |
| 1782 | K.LGGFNGTIVEPTILK.D |  | 1 | 0.1313 | 55.610 | 0.000 | 779.95 | 0 | 2 | 8/28 | 8327 |
| 1783 | R.TQSTASLFATITGASK.T |  | 2 | 0.1586 | 121.500 | 0.000 | 792.42 | 0 | 2 | 11/30 | 8328 |
| 1784 | R.TLDVVGLDTFAHVAR.N |  | 2 | 0.3753 | 71.910 | 0.000 | 807.44 | 1 | 2 | 11/28 | 8335 |
| 1785 | K.TQRPSVCNAIESLLIHK.A |  | 2 | 0.4599 | 40.380 | 0.000 | 492.27 | 0 | 4 | 18/32 | 8341 |
| 1786 | R.TLLDHFDQYIK.I |  | 1 | 0.6203 | 26.260 | 0.000 | 464.91 | 0 | 3 | 13/20 | 8345 |
| 1787 | R.NTPGVTGFVGSAGSGSKPTPLLPGEAETILK.R |  | 1 | 0.0095 | 37.270 | 0.000 | 994.86 | 0 | 3 | 13/60 | 8349 |
| 1788 | K.IWLTTEFTGGR.H |  | 1 | 0.9051 | 46.470 | 0.904 | 640.84 | 0 | 2 | 6/20 | 8357 |
| 1789 | R.LPELLSEAYLSK.E |  | 1 | 0.9245 | 64.660 | 0.879 | 681.88 | 1 | 2 | 10/22 | 8358 |
| 1790 | R.SLLSLIEK.E |  | 1 | 0.9523 | 38.740 | 0.947 | 451.78 | 0 | 2 | 7/14 | 8362 |
| 1791 | K.LFGIVQFEK.E |  | 1 | 0.7158 | 23.980 | 0.499 | 540.81 | 0 | 2 | 11/16 | 8366 |
| 1792 | K.FFLTFDSK.R |  | 1 | 0.8787 | 26.610 | 0.716 | 502.76 | 0 | 2 | 4/14 | 8370 |
| 1793 | K.VLEGLGATWEEVSLPHSK.Y |  | 2 | 0.8964 | 105.820 | 0.996 | 976.51 | 0 | 2 | 15/34 | 8372 |
| 1794 | K.LFANLLETAGATR.V |  | 2 | 0.8770 | 86.180 | 0.917 | 688.88 | 0 | 2 | 11/24 | 8375 |
| 1795 | R.LLDLGAPSIIVQNEK.R |  | 2 | 0.4424 | 59.790 | 0.000 | 805.46 | 0 | 2 | 20/28 | 8376 |
| 1796 | R.ITLANNPSHLEFINPIVEGSTR.A |  | 1 | 0.5722 | 26.790 | 0.000 | 808.10 | 0 | 3 | 24/42 | 8379 |
| 1797 | R.LGIDPYDVLTK.I |  | 1 | 0.2611 | 36.840 | 0.000 | 617.34 | 0 | 2 | 8/20 | 8389 |
| 1798 | R.HGALLICDEVICGFGR.T |  | 1 | 0.4149 | 30.170 | 0.000 | 606.30 | 0 | 3 | 15/30 | 8390 |
| 1799 | K.TLGINVVGAPENVFK.N |  | 1 | 0.0969 | 58.660 | 0.000 | 779.44 | 0 | 2 | 7/28 | 8392 |
| 1800 | K.DSIEFFVDGDK.I |  | 1 | 0.8810 | 41.700 | 0.908 | 636.29 | 0 | 2 | 8/20 | 8401 |
| 1801 | K.VFQYFLDEYK.A |  | 1 | 0.7508 | 44.500 | 0.000 | 676.33 | 0 | 2 | 13/18 | 8409 |
| 1802 | K.LLPTLLLR.N |  | 1 | 0.8928 | 30.920 | 0.960 | 469.82 | 0 | 2 | 7/14 | 8413 |
| 1803 | R.DQAILDIIR.Y |  | 1 | 0.4423 | 34.540 | 0.000 | 528.81 | 0 | 2 | 11/16 | 8418 |
| 1804 | R.GIAYGLVAPLYEQAK.V |  | 1 | 0.2307 | 40.210 | 0.000 | 796.94 | 0 | 2 | 17/28 | 8419 |
| 1805 | R.AHEHSVENLHASK.V |  | 1 | 0.2917 | 21.720 | 0.000 | 365.43 | 1 | 4 | 10/24 | 841 |
| 1806 | K.SAGLSGAISGGAEQLFGK.Q |  | 2 | 0.4496 | 80.050 | 0.946 | 825.43 | 0 | 2 | 10/34 | 8429 |
| 1807 | K.QVWDGPLFVR.V |  | 1 | 0.4029 | 40.650 | 0.000 | 608.83 | 0 | 2 | 6/18 | 8432 |
| 1808 | K.AEAVVCAPALFLEK.L |  | 1 | 0.8883 | 41.690 | 0.984 | 506.61 | 1 | 3 | 12/26 | 8433 |
| 1809 | K.CVVLPYPSNPTGVTLSEEELK.S |  | 1 | 0.9363 | 60.750 | 0.942 | 778.06 | 0 | 3 | 19/40 | 8442 |
| 1810 | K.LAAFGYTEGLQPEQQGVFVK.A |  | 2 | 0.1725 | 40.800 | 0.000 | 1091.56 | 0 | 2 | 13/38 | 8444 |
| 1811 | R.LGIDLGIIK.G |  | 1 | 0.5219 | 50.150 | 0.000 | 471.31 | 0 | 2 | 13/16 | 8447 |
| 1812 | R.DDFESITPFVK.G |  | 1 | 0.4429 | 52.800 | 0.000 | 649.32 | 1 | 2 | 8/20 | 8453 |
| 1813 | K.QNAENLLAPYLER.G |  | 1 | 0.8696 | 56.720 | 0.896 | 765.90 | 0 | 2 | 9/24 | 8454 |
| 1814 | K.EIEIFEA.- |  | 1 | 0.6370 | 27.360 | 0.000 | 425.71 | 0 | 2 | 8/12 | 8458 |
| 1815 | K.WEEGGETLTPSLDLVGK.I |  | 1 | 0.1910 | 99.690 | 0.000 | 915.96 | 0 | 2 | 13/32 | 8479 |
| 1816 | R.LTEVEGFEQFLHR.T |  | 1 | 0.9803 | 30.270 | 0.985 | 535.61 | 0 | 3 | 17/24 | 8482 |
| 1817 | K.M#AGDKFDM#EILNYIK.R |  | 1 | 0.1781 | 27.630 | 0.000 | 607.29 | 0 | 3 | 9/28 | 8484 |
| 1818 | K.SPIQTTGQLVDLIK.D |  | 1 | 0.2959 | 55.000 | 0.000 | 756.94 | 0 | 2 | 10/26 | 8495 |
| 1819 | K.FGFDIEDDDIR.N |  | 1 | 0.9519 | 57.770 | 0.814 | 671.30 | 0 | 2 | 7/20 | 8501 |
| 1820 | K.GYLDDIPVADIR.R |  | 2 | 0.8978 | 44.920 | 0.963 | 673.85 | 0 | 2 | 8/22 | 8507 |
| 1821 | K.SLLGEIQPLEGSVER.G |  | 2 | 0.5206 | 68.870 | 0.000 | 813.94 | 0 | 2 | 20/28 | 8510 |
| 1822 | R.SVIINGPVQLQGAEVAATDLR.A |  | 2 | 0.1588 | 69.760 | 0.000 | 1076.09 | 0 | 2 | 15/40 | 8518 |
| 1823 | K.VVIFNDEDLEFAK.T |  | 1 | 0.3928 | 64.190 | 0.000 | 769.89 | 0 | 2 | 8/24 | 8521 |
| 1824 | R.LGIQAFEPTLVEGR.A |  | 1 | 0.2229 | 90.950 | 0.000 | 765.42 | 0 | 2 | 9/26 | 8528 |
| 1825 | K.VLPYLENGIGFR.K |  | 2 | 0.7100 | 53.370 | 0.000 | 689.38 | 1 | 2 | 16/22 | 8539 |
| 1826 | R.QVGLTLPLVVR.L |  | 2 | 0.2644 | 42.610 | 0.000 | 398.92 | 0 | 3 | 6/20 | 8542 |
| 1827 | K.LELIAQTLLK.V |  | 1 | 0.9083 | 35.960 | 0.917 | 571.36 | 0 | 2 | 6/18 | 8543 |
| 1828 | R.VCNQIGIPYYAVNFEK.Q |  | 1 | 0.5780 | 53.770 | 0.000 | 957.98 | 1 | 2 | 16/30 | 8544 |
| 1829 | R.WLGGTLTNFETIQK.R |  | 2 | 0.9523 | 73.740 | 0.995 | 804.42 | 0 | 2 | 10/26 | 8550 |
| 1830 | R.LVNDLLDLAR.M |  | 1 | 0.2706 | 37.400 | 0.000 | 571.33 | 1 | 2 | 8/18 | 8566 |
| 1831 | K.LINSETTQEQLEPIIEEIKK.T |  | 1 | 0.8807 | 41.550 | 0.992 | 785.76 | 0 | 3 | 13/38 | 8567 |
| 1832 | K.AEEEFDLNDLSVPPGVK.I |  | 1 | 0.8940 | 37.660 | 0.872 | 929.96 | 1 | 2 | 18/32 | 8568 |
| 1833 | K.TNPDYLFVIDR.G |  | 1 | 0.5077 | 52.660 | 0.000 | 676.85 | 0 | 2 | 8/20 | 8573 |
| 1834 | K.TGLLIDPYFSGTK.V |  | 1 | 0.1072 | 22.960 | 0.000 | 706.38 | 1 | 2 | 8/24 | 8581 |
| 1835 | R.GVLINLWR.E |  | 1 | 0.8497 | 27.750 | 0.774 | 485.80 | 0 | 2 | 5/14 | 8584 |
| 1836 | K.NLIEVPM#VGTTIPHEIIGR.F |  | 1 | 0.3337 | 55.680 | 0.000 | 1053.07 | 2 | 2 | 13/36 | 8586 |
| 1837 | K.LISFLQNELNVNK.I |  | 2 | 0.9684 | 55.620 | 0.903 | 766.43 | 1 | 2 | 10/24 | 8588 |
| 1838 | K.QVVSAATACIPFLENDDSNR.A |  | 1 | 0.2538 | 38.050 | 0.000 | 736.35 | 0 | 3 | 18/38 | 8594 |
| 1839 | K.DGTYTYLLPDIAYHK.D |  | 2 | 0.2772 | 62.550 | 0.000 | 885.44 | 0 | 2 | 9/28 | 8601 |
| 1840 | R.DELIEELVK.S |  | 1 | 0.3740 | 29.860 | 0.000 | 544.30 | 0 | 2 | 4/16 | 8604 |
| 1841 | K.LSTWALTFEQAAAK.M |  | 1 | 0.1910 | 67.770 | 0.000 | 768.91 | 0 | 2 | 8/26 | 8607 |
| 1842 | K.DSYLNVTNIVSVAK.L |  | 1 | 0.2202 | 57.980 | 0.000 | 761.91 | 0 | 2 | 8/26 | 8608 |
| 1843 | K.IGGPADVLVIPSSVDAVK.D |  | 1 | 0.1034 | 56.940 | 0.000 | 868.99 | 0 | 2 | 18/34 | 8610 |
| 1844 | K.DVLVSTVEALNR.R |  | 2 | 0.9644 | 59.110 | 0.806 | 439.25 | 0 | 3 | 15/22 | 8611 |
| 1845 | R.DTEKPFM#M#PVEDVFSITGR.G |  | 1 | 0.1993 | 58.510 | 0.000 | 744.35 | 0 | 3 | 10/36 | 8614 |
| 1846 | R.ISNETLEIFAPLAHR.L |  | 1 | 0.5698 | 48.940 | 0.000 | 570.98 | 1 | 3 | 15/28 | 8616 |
| 1847 | K.EQLIFPEIDYDK.V |  | 2 | 0.5393 | 25.550 | 0.000 | 503.92 | 0 | 3 | 12/22 | 8618 |
| 1848 | R.DVIDSNIFVVSR.R |  | 1 | 0.9384 | 56.420 | 0.979 | 682.37 | 1 | 2 | 9/22 | 8619 |
| 1849 | K.SYGVNFPVVLDTDR.Q |  | 1 | 0.3447 | 65.510 | 0.000 | 791.40 | 0 | 2 | 11/26 | 8626 |
| 1850 | K.ASLETDSFLSAASFQETTR.V |  | 2 | 0.1544 | 72.810 | 0.000 | 1030.99 | 0 | 2 | 10/36 | 8628 |
| 1851 | R.TLM#FNIYDLK.W |  | 1 | 0.4950 | 31.540 | 0.000 | 637.33 | 3 | 2 | 6/18 | 8631 |
| 1852 | K.IGVIESLLDK.V |  | 1 | 0.9118 | 59.190 | 0.858 | 543.82 | 1 | 2 | 8/18 | 8640 |
| 1853 | K.FADDWLSIR.Q |  | 1 | 0.6456 | 62.010 | 0.000 | 561.78 | 0 | 2 | 6/16 | 8645 |
| 1854 | K.TTLPLLVTAQQGLNEPR.Y |  | 2 | 0.8833 | 105.970 | 0.985 | 926.02 | 0 | 2 | 11/32 | 8649 |
| 1855 | R.TLILDQAEVPKENLLGEIGK.G |  | 1 | 0.6970 | 25.540 | 0.939 | 727.41 | 0 | 3 | 15/38 | 8658 |
| 1856 | K.LGPLQFGQSQGGQVFLGR.D |  | 2 | 0.5769 | 65.020 | 0.000 | 945.01 | 0 | 2 | 22/34 | 8662 |
| 1857 | R.QELDLFVCLRPVR.Y |  | 1 | 0.3234 | 30.950 | 0.000 | 548.97 | 1 | 3 | 7/24 | 8665 |
| 1858 | K.GHEVHALSR.T |  | 1 | 0.2432 | 30.210 | 0.000 | 335.85 | 0 | 3 | 6/16 | 866 |
| 1859 | R.VTAVSSALAAETYDLIKPEK.K |  | 1 | 0.0619 | 24.350 | 0.000 | 702.72 | 1 | 3 | 17/38 | 8674 |
| 1860 | K.LSEHLEELGFDLVR.F |  | 1 | 0.4731 | 31.390 | 0.000 | 552.96 | 0 | 3 | 15/26 | 8675 |
| 1861 | K.VLAWYDNEWGYSCR.V |  | 2 | 0.6681 | 70.550 | 0.000 | 909.90 | 0 | 2 | 9/26 | 8679 |
| 1862 | K.RDDQPIGVIPIDSIYTPVSR.V |  | 1 | 0.2133 | 39.890 | 0.000 | 747.73 | 0 | 3 | 16/38 | 8681 |
| 1863 | K.DVLDLIDHVK.K |  | 1 | 0.4895 | 29.550 | 0.000 | 389.55 | 0 | 3 | 13/18 | 8685 |
| 1864 | R.LEYYTQEELADIVTR.T |  | 1 | 0.4865 | 31.020 | 0.000 | 614.98 | 1 | 3 | 12/28 | 8704 |
| 1865 | K.ELAGLFEEAGLK.N |  | 1 | 0.8775 | 47.280 | 0.968 | 638.84 | 0 | 2 | 9/22 | 8713 |
| 1866 | R.ESYLQNYFGTTVNIK.R |  | 1 | 0.3275 | 40.340 | 0.000 | 888.94 | 0 | 2 | 7/28 | 8718 |
| 1867 | K.LVSPQSIAIAAAAVGQTGK.E |  | 2 | 0.0693 | 106.040 | 0.000 | 891.51 | 0 | 2 | 13/36 | 8722 |
| 1868 | K.DILAYLR.L |  | 1 | 0.8247 | 22.180 | 0.524 | 432.25 | 0 | 2 | 8/12 | 8726 |
| 1869 | K.NILYVDQGFHNPLLYSDK.I |  | 1 | 0.3930 | 43.920 | 0.000 | 1068.54 | 0 | 2 | 13/34 | 8729 |
| 1870 | K.YPIISIEDGLDENDWEGHK.L |  | 2 | 0.9875 | 71.060 | 0.976 | 744.01 | 0 | 3 | 23/36 | 8732 |
| 1871 | R.LLGLQEIIER.S |  | 1 | 0.9441 | 45.330 | 0.871 | 592.36 | 0 | 2 | 9/18 | 8734 |
| 1872 | R.QSIIHEFTGLLEK.W |  | 1 | 0.8887 | 40.440 | 0.971 | 757.91 | 0 | 2 | 10/24 | 8735 |
| 1873 | K.SEIVISAVKPEQYPEGGLPEIALAGR.S |  | 1 | 0.1420 | 62.900 | 0.000 | 908.49 | 0 | 3 | 21/50 | 8736 |
| 1874 | R.AGAALILAGLVAEGHTR.V |  | 2 | 0.1746 | 68.520 | 0.000 | 810.47 | 1 | 2 | 17/32 | 8737 |
| 1875 | R.SDFIIFSGGLGPTKDDLTK.E |  | 1 | 0.0541 | 31.510 | 0.000 | 671.02 | 1 | 3 | 10/36 | 8738 |
| 1876 | R.EADYTLLLHAGPEIAVASTK.A |  | 2 | 0.3788 | 60.730 | 0.000 | 1050.05 | 1 | 2 | 21/38 | 8756 |
| 1877 | R.VLLVDIDPQGNATSGLGIEK.A |  | 2 | 0.1879 | 93.230 | 0.000 | 1020.05 | 0 | 2 | 14/38 | 8758 |
| 1878 | R.TVAAILENYQQEDGSVVIPK.V |  | 1 | 0.0658 | 35.810 | 0.000 | 1087.57 | 0 | 2 | 9/38 | 8769 |
| 1879 | R.NLPYIGVLKPAVYES.- |  | 1 | 0.4471 | 39.250 | 0.000 | 831.96 | 0 | 2 | 14/28 | 8775 |
| 1880 | R.FIISELIR.E |  | 1 | 0.9839 | 46.680 | 0.983 | 495.80 | 0 | 2 | 7/14 | 8776 |
| 1881 | K.VVDLLAPYIK.G |  | 1 | 0.3967 | 47.790 | 0.000 | 565.84 | 0 | 2 | 9/18 | 8777 |
| 1882 | K.LTALHAALVNGGAFLYVPK.N |  | 1 | 0.0688 | 22.560 | 0.000 | 652.38 | 1 | 3 | 9/36 | 8779 |
| 1883 | K.LGAVQNR.L |  | 1 | 0.4813 | 41.210 | 0.000 | 379.22 | 1 | 2 | 6/12 | 877 |
| 1884 | K.TIYELDPVTLTYGER.T |  | 1 | 0.7458 | 65.310 | 0.000 | 885.45 | 1 | 2 | 22/28 | 8787 |
| 1885 | K.IQLIDGLLGK.Y |  | 1 | 0.8534 | 45.390 | 0.891 | 535.33 | 1 | 2 | 7/18 | 8789 |
| 1886 | K.NYDNILDALR.S |  | 1 | 0.9637 | 41.940 | 0.994 | 603.81 | 0 | 2 | 8/18 | 8799 |
| 1887 | K.FLIELENLK.S |  | 1 | 0.9625 | 48.770 | 0.851 | 559.83 | 0 | 2 | 7/16 | 8800 |
| 1888 | R.VGEFFYGYDDTSLVK.E |  | 1 | 0.3069 | 69.020 | 0.000 | 870.41 | 0 | 2 | 12/28 | 8805 |
| 1889 | K.ALEEIDAGLLSFEKEDR.E |  | 1 | 0.0597 | 27.520 | 0.000 | 645.66 | 1 | 3 | 7/32 | 8818 |
| 1890 | K.LTVYDLIK.K |  | 1 | 0.9181 | 29.570 | 0.927 | 482.79 | 1 | 2 | 6/14 | 8827 |
| 1891 | K.EGIISSAQDFNDYVIDAGYHK.E |  | 1 | 0.5812 | 41.850 | 0.000 | 781.37 | 0 | 3 | 21/40 | 8841 |
| 1892 | K.NDLLITSVLSGNR.N |  | 2 | 0.5811 | 95.470 | 0.000 | 701.39 | 1 | 2 | 19/24 | 8845 |
| 1893 | K.SGLLGISGFSSDLR.D |  | 1 | 0.1303 | 39.460 | 0.000 | 704.87 | 1 | 2 | 10/26 | 8852 |
| 1894 | K.IDAVFDALQDKGPEIIVAR.H |  | 1 | 0.1526 | 62.760 | 0.000 | 690.71 | 1 | 3 | 12/36 | 8853 |
| 1895 | K.FADSDLIGLPIR.I |  | 1 | 0.3697 | 49.440 | 0.000 | 658.86 | 1 | 2 | 9/22 | 8854 |
| 1896 | K.LEAPDIGQLFHSSLK.L |  | 1 | 0.8934 | 29.940 | 0.970 | 552.30 | 1 | 3 | 17/28 | 8858 |
| 1897 | R.DVENDDLLLSLK.V |  | 1 | 0.5252 | 60.810 | 0.000 | 687.36 | 0 | 2 | 7/22 | 8863 |
| 1898 | K.WSNGDPVTAQDFEYAWK.W |  | 1 | 0.3989 | 85.050 | 0.000 | 1007.45 | 0 | 2 | 10/32 | 8864 |
| 1899 | R.FEILQYIR.L |  | 1 | 0.5405 | 26.200 | 0.000 | 541.31 | 1 | 2 | 7/14 | 8869 |
| 1900 | K.TTTSANLGTALAILGK.R |  | 2 | 0.8605 | 95.780 | 0.958 | 766.44 | 0 | 2 | 13/30 | 8871 |
| 1901 | K.KTVPTAFEFTDIAGIVK.G |  | 1 | 0.1613 | 44.570 | 0.000 | 613.01 | 0 | 3 | 8/32 | 8873 |
| 1902 | R.GNSGVILSQLFR.G |  | 1 | 0.1440 | 25.790 | 0.000 | 645.86 | 2 | 2 | 8/22 | 8877 |
| 1903 | K.SVFEISDEINGLATK.A |  | 1 | 0.9330 | 76.070 | 0.969 | 811.92 | 0 | 2 | 12/28 | 8879 |
| 1904 | K.LLEIIPIKETDPDILK.F |  | 1 | 0.8192 | 36.280 | 0.953 | 617.37 | 0 | 3 | 15/30 | 8887 |
| 1905 | R.VANSITELIGNTPIVK.L |  | 2 | 0.2905 | 57.400 | 0.000 | 834.98 | 0 | 2 | 20/30 | 8890 |
| 1906 | K.TINIVIPYYGYAR.Q |  | 1 | 0.2335 | 30.190 | 0.000 | 514.95 | 0 | 3 | 5/24 | 8893 |
| 1907 | R.KSFLDQLFR.L |  | 1 | 0.2695 | 26.070 | 0.000 | 385.22 | 0 | 3 | 6/16 | 8895 |
| 1908 | K.TVLILNFPAEGHVNPTLGITK.A |  | 1 | 0.0768 | 28.430 | 0.000 | 745.42 | 1 | 3 | 7/40 | 8897 |
| 1909 | K.QEFDYVIIDCPAGIEQGYK.N |  | 2 | 0.6564 | 48.430 | 0.000 | 749.02 | 0 | 3 | 16/36 | 8898 |
| 1910 | K.STLFQLIPR.L |  | 1 | 0.6153 | 53.860 | 0.000 | 537.82 | 0 | 2 | 7/16 | 8902 |
| 1911 | K.NIDETAEYFVNFK.V |  | 2 | 0.5900 | 88.790 | 0.000 | 795.38 | 0 | 2 | 9/24 | 8906 |
| 1912 | K.KQAQDSVKEEAQR.S |  | 1 | 0.6505 | 42.420 | 0.908 | 506.26 | 0 | 3 | 11/24 | 891 |
| 1913 | R.LEDLPIIPEPLK.L |  | 1 | 0.4198 | 42.780 | 0.000 | 688.91 | 0 | 2 | 12/22 | 8926 |
| 1914 | R.AVGFGLQFLK.G |  | 1 | 0.0700 | 24.220 | 0.000 | 540.32 | 0 | 2 | 6/18 | 8931 |
| 1915 | K.AWIPIDGTYDLGPGPR.M |  | 2 | 0.9197 | 65.380 | 0.983 | 864.44 | 0 | 2 | 12/30 | 8935 |
| 1916 | K.FASVCVNPTWVELAAK.E |  | 1 | 0.2499 | 51.970 | 0.000 | 896.46 | 0 | 2 | 11/30 | 8936 |
| 1917 | K.VGDQAPDFTVLTNSLEEK.S |  | 2 | 0.2944 | 68.040 | 0.000 | 981.99 | 1 | 2 | 13/34 | 8940 |
| 1918 | K.LTAQEALTLIEK.L |  | 1 | 0.4313 | 83.640 | 0.000 | 665.39 | 1 | 2 | 9/22 | 8943 |
| 1919 | R.YLELFLPK.M |  | 1 | 0.4793 | 22.470 | 0.000 | 511.80 | 0 | 2 | 9/14 | 8946 |
| 1920 | K.DVEFQQYEPIPGWK.V |  | 2 | 0.7723 | 73.070 | 0.000 | 868.42 | 0 | 2 | 19/26 | 8956 |
| 1921 | K.AVSFGEVLQDDFK.T |  | 2 | 0.9085 | 80.980 | 0.945 | 727.86 | 0 | 2 | 10/24 | 8957 |
| 1922 | K.INPEIPVDLVIDHSVQVDK.A |  | 1 | 0.8472 | 24.970 | 0.911 | 710.72 | 0 | 3 | 17/36 | 8961 |
| 1923 | K.EETSTGFQLGDLIGDK.L |  | 1 | 0.2348 | 94.550 | 0.000 | 855.41 | 0 | 2 | 10/30 | 8963 |
| 1924 | R.EEDPVHYEESIDSYFISR.Y |  | 1 | 0.2455 | 35.520 | 0.000 | 739.00 | 0 | 3 | 7/34 | 8964 |
| 1925 | K.LTDDYPVFPISAVTR.E |  | 1 | 0.4723 | 80.020 | 0.000 | 847.44 | 0 | 2 | 20/28 | 8965 |
| 1926 | R.DLVHQDVYEFLEK.L |  | 1 | 0.5847 | 33.960 | 0.000 | 545.61 | 0 | 3 | 11/24 | 8967 |
| 1927 | K.DGFIVIDPNQIGSSVQR.L |  | 1 | 0.2069 | 51.240 | 0.000 | 922.98 | 0 | 2 | 9/32 | 8975 |
| 1928 | K.TYAAFEPALDYVVSK.I |  | 1 | 0.2602 | 55.980 | 0.000 | 837.42 | 0 | 2 | 12/28 | 8985 |
| 1929 | K.KVVVFDFGSLDTLDK.L |  | 1 | 0.1592 | 48.080 | 0.000 | 561.64 | 0 | 3 | 9/28 | 8987 |
| 1930 | K.VEIFSAGEIVDVTGVSK.G |  | 2 | 0.8567 | 98.920 | 0.986 | 875.47 | 1 | 2 | 10/32 | 8991 |
| 1931 | K.DLLLFYSSK.E |  | 1 | 0.4489 | 31.880 | 0.000 | 543.30 | 0 | 2 | 10/16 | 8995 |
| 1932 | K.NGVLFVGSTEQIFNPEK.F |  | 1 | 0.2960 | 80.860 | 0.000 | 939.98 | 0 | 2 | 11/32 | 8996 |
| 1933 | K.GDDAIFAATGVTDGELLK.G |  | 2 | 0.2109 | 64.680 | 0.000 | 896.95 | 1 | 2 | 19/34 | 9005 |
| 1934 | K.GYFIKPTIFADLDPK.A |  | 1 | 0.2339 | 36.400 | 0.000 | 575.65 | 0 | 3 | 12/28 | 9021 |
| 1935 | R.IIPAINQLGLVVR.G |  | 1 | 0.2750 | 64.990 | 0.000 | 703.45 | 0 | 2 | 9/24 | 9022 |
| 1936 | R.KLLDYAEAGDNIGALLR.G |  | 1 | 0.1613 | 50.830 | 0.000 | 611.34 | 0 | 3 | 17/32 | 9024 |
| 1937 | R.LAEPLLFLSK.Y |  | 1 | 0.9152 | 51.420 | 0.931 | 565.84 | 0 | 2 | 8/18 | 9036 |
| 1938 | R.EQIVITEIPFEVNK.A |  | 1 | 0.8197 | 34.130 | 0.942 | 829.95 | 1 | 2 | 7/26 | 9037 |
| 1939 | R.IGIADSVFTISVNGEK.N |  | 1 | 0.1662 | 81.710 | 0.000 | 825.44 | 0 | 2 | 9/30 | 9042 |
| 1940 | R.VVENAKPLPVVTYTEICNLAYQGAK.V |  | 2 | 0.3485 | 52.510 | 0.000 | 926.49 | 0 | 3 | 24/48 | 9044 |
| 1941 | R.QDDAAGTLYNIVGFQTHLK.W |  | 1 | 0.4190 | 61.650 | 0.000 | 697.69 | 1 | 3 | 23/36 | 9045 |
| 1942 | K.VFIALPPLYK.V |  | 1 | 0.9095 | 44.690 | 0.994 | 580.86 | 1 | 2 | 5/18 | 9049 |
| 1943 | R.LAPQFEVALFR.L |  | 1 | 0.6028 | 57.530 | 0.000 | 645.86 | 0 | 2 | 8/20 | 9053 |
| 1944 | K.EIEDISELAPLHNPANIVGIK.A |  | 1 | 0.1364 | 31.040 | 0.000 | 758.08 | 0 | 3 | 9/40 | 9066 |
| 1945 | K.TAQLADGAAQVTSGIQSLDSSLGK.F |  | 2 | 0.2140 | 64.590 | 0.000 | 773.40 | 1 | 3 | 23/46 | 9070 |
| 1946 | K.YYGIPVIVIATK.A |  | 1 | 0.1450 | 33.930 | 0.000 | 668.90 | 1 | 2 | 5/22 | 9072 |
| 1947 | R.YENWISGFNACPVLK.L |  | 2 | 0.5658 | 63.110 | 0.000 | 899.44 | 0 | 2 | 12/28 | 9078 |
| 1948 | K.WVSLFENLK.Y |  | 1 | 0.9404 | 26.660 | 0.950 | 568.31 | 0 | 2 | 6/16 | 9084 |
| 1949 | K.TYAELLQAPLEVCYTK.E |  | 2 | 0.6699 | 65.500 | 0.000 | 949.98 | 0 | 2 | 22/30 | 9087 |
| 1950 | K.IFTTQLTGIFSR.I |  | 1 | 0.4101 | 41.230 | 0.000 | 692.39 | 0 | 2 | 9/22 | 9089 |
| 1951 | R.DKFEQALGELGVPQPLGK.T |  | 1 | 0.0748 | 20.210 | 0.000 | 642.68 | 1 | 3 | 13/34 | 9090 |
| 1952 | K.AIYQAVSPSFLTVTPGIR.M |  | 1 | 0.0467 | 23.360 | 0.000 | 640.69 | 0 | 3 | 8/34 | 9092 |
| 1953 | R.AAIVPELTDEFIK.L |  | 2 | 0.4214 | 45.600 | 0.000 | 723.40 | 1 | 2 | 15/24 | 9097 |
| 1954 | K.AFGLTIPLVTK.A |  | 1 | 0.6224 | 28.900 | 0.837 | 580.36 | 1 | 2 | 4/20 | 9102 |
| 1955 | K.LSVPGDIAAAVAEADR.I |  | 2 | 0.1998 | 117.860 | 0.000 | 777.91 | 0 | 2 | 14/30 | 9108 |
| 1956 | K.NSLILFDEIGR.G |  | 1 | 0.4672 | 60.530 | 0.000 | 638.85 | 1 | 2 | 9/20 | 9111 |
| 1957 | K.FSLLLTEIAK.N |  | 1 | 0.4565 | 49.290 | 0.000 | 567.84 | 0 | 2 | 14/18 | 9119 |
| 1958 | K.LDEEALVAILTKPK.N |  | 2 | 0.8815 | 85.170 | 0.988 | 770.45 | 0 | 2 | 11/26 | 9121 |
| 1959 | R.AFDLTEGDTPIAEALK.K |  | 1 | 0.8890 | 49.340 | 0.920 | 845.93 | 0 | 2 | 18/30 | 9133 |
| 1960 | K.DKFSLLLTEIAK.N |  | 1 | 0.5631 | 30.870 | 0.676 | 459.94 | 0 | 3 | 13/22 | 9135 |
| 1961 | K.YDYIVGDWGYGQLR.L |  | 1 | 0.6235 | 78.600 | 0.000 | 852.90 | 0 | 2 | 12/26 | 9137 |
| 1962 | R.WAAPWHVIEIIK.S |  | 1 | 0.5731 | 27.410 | 0.000 | 488.28 | 2 | 3 | 15/22 | 9138 |
| 1963 | K.KLEHQLETK.M |  | 1 | 0.7860 | 25.890 | 0.845 | 375.88 | 1 | 3 | 5/16 | 913 |
| 1964 | R.VLQLLELPYR.V |  | 2 | 0.5999 | 61.460 | 0.000 | 622.37 | 0 | 2 | 8/18 | 9141 |
| 1965 | K.DLIPIIPYER.K |  | 1 | 0.8629 | 38.120 | 0.775 | 614.85 | 0 | 2 | 5/18 | 9142 |
| 1966 | R.ENILLPLALDK.V |  | 1 | 0.2031 | 45.430 | 0.000 | 619.87 | 0 | 2 | 7/20 | 9146 |
| 1967 | R.ALAIGGTAVGTGINAHPEFGELVSEEITK.L |  | 1 | 0.0446 | 57.470 | 0.000 | 961.17 | 0 | 3 | 17/56 | 9155 |
| 1968 | K.THFEDLFGTPTGLDFK.H |  | 1 | 0.1694 | 28.890 | 0.000 | 608.97 | 1 | 3 | 6/30 | 9156 |
| 1969 | K.NLPLELTLVEASPR.V |  | 2 | 0.9565 | 76.920 | 0.955 | 776.44 | 0 | 2 | 15/26 | 9163 |
| 1970 | K.GIHLVFDQSVFPLK.Q |  | 1 | 0.5916 | 46.460 | 0.000 | 800.45 | 0 | 2 | 17/26 | 9164 |
| 1971 | R.GIDENKDQTYFLNQLTEDTLSK.V |  | 1 | 0.1182 | 41.410 | 0.000 | 858.09 | 0 | 3 | 9/42 | 9165 |
| 1972 | K.DFFIPNVVGR.E |  | 1 | 0.2957 | 31.740 | 0.000 | 582.31 | 0 | 2 | 6/18 | 9170 |
| 1973 | K.IISNLFENALGNYK.E |  | 1 | 0.3903 | 88.550 | 0.000 | 798.43 | 0 | 2 | 9/26 | 9187 |
| 1974 | R.TTNFNAGPAALPLEVLQK.A |  | 1 | 0.3803 | 57.670 | 0.000 | 942.51 | 0 | 2 | 21/34 | 9191 |
| 1975 | K.ILPVLPDDLIER.A |  | 1 | 0.1353 | 25.350 | 0.000 | 696.91 | 0 | 2 | 4/22 | 9193 |
| 1976 | R.VIDAGDTDVLPGTLLDIHQFTEANKK.V |  | 1 | 0.1405 | 27.400 | 0.000 | 703.37 | 0 | 4 | 17/50 | 9196 |
| 1977 | M.SILDIHDVSVWYER.D |  | 1 | 0.2810 | 21.510 | 0.000 | 577.96 | 1 | 3 | 5/26 | 9197 |
| 1978 | K.LASEDKPFLYTVWGVGYK.F |  | 1 | 0.3373 | 29.180 | 0.000 | 691.69 | 0 | 3 | 19/34 | 9198 |
| 1979 | K.ELGLKPVDVFLVSAGR.G |  | 1 | 0.0523 | 27.360 | 0.000 | 567.33 | 0 | 3 | 10/30 | 9200 |
| 1980 | K.NDTTLYDIILK.A |  | 1 | 0.8884 | 50.730 | 0.806 | 654.86 | 1 | 2 | 9/20 | 9216 |
| 1981 | R.EANELGILAGVTTNPSLVAK.E |  | 1 | 0.0933 | 76.750 | 0.000 | 999.05 | 0 | 2 | 12/38 | 9219 |
| 1982 | K.AFIDWFNK.L |  | 1 | 0.9147 | 41.010 | 0.508 | 520.76 | 0 | 2 | 12/14 | 9220 |
| 1983 | K.AFSYNWVDDFGR.G |  | 2 | 0.5895 | 59.810 | 0.000 | 738.83 | 0 | 2 | 8/22 | 9221 |
| 1984 | R.ETYPELNIIAGNVATAEATR.A |  | 2 | 0.1653 | 54.570 | 0.000 | 1067.04 | 1 | 2 | 14/38 | 9225 |
| 1985 | R.VFNVLGENIDLNEPVPADAK.K |  | 2 | 0.1838 | 56.100 | 0.000 | 1077.56 | 0 | 2 | 13/38 | 9230 |
| 1986 | R.VDYNCAQGYFGVTPDLTCLGK.V |  | 2 | 0.2095 | 43.680 | 0.000 | 1189.54 | 0 | 2 | 13/40 | 9231 |
| 1987 | K.IWASIPVSIDLKPIAEAK.E |  | 1 | 0.0397 | 28.740 | 0.000 | 651.05 | 0 | 3 | 10/34 | 9234 |
| 1988 | K.IGADFLYSVGTLR.D |  | 1 | 0.9396 | 47.480 | 0.979 | 471.26 | 1 | 3 | 14/24 | 9236 |
| 1989 | K.SDTVGAITEILEPVK.E |  | 1 | 0.3688 | 29.660 | 0.598 | 786.43 | 1 | 2 | 10/28 | 9245 |
| 1990 | K.QGVTPGLAVILIGDDPASHSYVR.G |  | 1 | 0.2788 | 47.900 | 0.000 | 789.09 | 0 | 3 | 20/44 | 9249 |
| 1991 | K.TRPQGSGQQLTAK.K |  | 1 | 0.7220 | 77.740 | 0.940 | 457.92 | 0 | 3 | 9/24 | 924 |
| 1992 | K.DLDEIWSGIK.Q |  | 1 | 0.4856 | 28.210 | 0.000 | 588.30 | 0 | 2 | 10/18 | 9250 |
| 1993 | R.IINIVGNLWK.E |  | 1 | 0.2505 | 32.230 | 0.000 | 585.36 | 0 | 2 | 7/18 | 9256 |
| 1994 | R.YLSTPLYQFD.- |  | 1 | 0.6874 | 28.730 | 0.000 | 623.80 | 1 | 2 | 7/18 | 9259 |
| 1995 | K.AIDSAVEELTFIAGQKPVVTR.A |  | 3 | 0.1210 | 67.110 | 0.000 | 561.81 | 0 | 4 | 13/40 | 9283 |
| 1996 | K.IILTRPAVEAGESLGFLPGDLK.E |  | 1 | 0.1242 | 38.020 | 0.000 | 766.10 | 0 | 3 | 18/42 | 9285 |
| 1997 | R.IVNPLGQPVDGLGPILTSK.T |  | 2 | 0.1413 | 63.960 | 0.000 | 959.55 | 0 | 2 | 13/36 | 9287 |
| 1998 | K.NKDVEDVVATILNR.E |  | 1 | 0.8504 | 54.350 | 0.892 | 529.29 | 0 | 3 | 14/26 | 9288 |
| 1999 | K.QELVEVVEFLKDPR.K |  | 1 | 0.1389 | 25.560 | 0.000 | 850.96 | 0 | 2 | 9/26 | 9290 |
| 2000 | K.ILENFDPIYDATVVQR.L |  | 2 | 0.3930 | 74.650 | 0.000 | 946.99 | 0 | 2 | 9/30 | 9294 |
| 2001 | R.KGTLNVEFYPVLVGSAFK.N |  | 2 | 0.0960 | 123.660 | 0.000 | 985.04 | 0 | 2 | 15/34 | 9296 |
| 2002 | R.DKLELIAQTLLK.V |  | 2 | 0.2272 | 64.130 | 0.000 | 462.29 | 0 | 3 | 8/22 | 9297 |
| 2003 | R.FVIIDVPYIK.G |  | 1 | 0.4673 | 35.350 | 0.000 | 603.86 | 1 | 2 | 7/18 | 9305 |
| 2004 | K.TGM#LPTVDIIELAAK.T |  | 1 | 0.2484 | 33.390 | 0.000 | 794.44 | 1 | 2 | 17/28 | 9307 |
| 2005 | R.DLIEEVGLDAVR.Y |  | 1 | 0.2429 | 42.330 | 0.000 | 664.85 | 3 | 2 | 9/22 | 9312 |
| 2006 | R.DITPIEAGIGFAVK.H |  | 1 | 0.2169 | 44.660 | 0.000 | 715.90 | 1 | 2 | 14/26 | 9313 |
| 2007 | K.AVIECEDADNLYSIPLELQK.Q |  | 2 | 0.3116 | 43.290 | 0.000 | 774.05 | 1 | 3 | 11/38 | 9324 |
| 2008 | R.ILDINGLYDVPVEPVR.G |  | 1 | 0.3053 | 51.620 | 0.000 | 906.50 | 0 | 2 | 11/30 | 9332 |
| 2009 | K.ENVIIGK.L |  | 1 | 0.6495 | 25.040 | 0.614 | 386.73 | 1 | 2 | 4/12 | 9344 |
| 2010 | K.SIVISPFGEVRPCALFPK.E |  | 1 | 0.0905 | 32.530 | 0.000 | 1009.05 | 0 | 2 | 12/34 | 9349 |
| 2011 | R.VLDGAVAVLDAQSGVEPQTETVWR.Q |  | 3 | 0.0898 | 72.980 | 0.000 | 1270.65 | 1 | 2 | 14/46 | 9350 |
| 2012 | R.LPNTADFFNANLDNPDFAK.D |  | 1 | 0.1716 | 54.210 | 0.000 | 1062.50 | 1 | 2 | 13/36 | 9351 |
| 2013 | K.AYNPLFIYGGVGLGK.T |  | 1 | 0.2543 | 61.600 | 0.000 | 784.93 | 0 | 2 | 10/28 | 9353 |
| 2014 | K.GEFLFTFDAR.D |  | 1 | 0.3900 | 48.530 | 0.000 | 601.80 | 1 | 2 | 6/18 | 9366 |
| 2015 | K.KNAPCLIFIDEIDAVGR.Q |  | 1 | 0.3309 | 48.610 | 0.000 | 644.34 | 1 | 3 | 12/32 | 9367 |
| 2016 | R.LTLDTIGLCGFNYR.F |  | 1 | 0.3153 | 51.910 | 0.000 | 548.28 | 1 | 3 | 9/26 | 9371 |
| 2017 | K.IEETLIQPTFIYGHPVEISPLAK.K |  | 1 | 0.3538 | 34.200 | 0.000 | 865.81 | 1 | 3 | 17/44 | 9378 |
| 2018 | K.LPEDFQTAEFLLK.H |  | 1 | 0.9123 | 60.950 | 0.799 | 775.91 | 0 | 2 | 10/24 | 9386 |
| 2019 | K.STVIDAYDSFLKDDELK.R |  | 1 | 0.0977 | 21.410 | 0.000 | 653.66 | 0 | 3 | 10/32 | 9387 |
| 2020 | R.VFVTEVQAESVQFLEPK.N |  | 2 | 0.4164 | 69.920 | 0.000 | 975.51 | 0 | 2 | 10/32 | 9391 |
| 2021 | K.NFLPGADCLSTIDCFR.K |  | 1 | 0.9368 | 63.700 | 0.879 | 943.43 | 1 | 2 | 13/30 | 9392 |
| 2022 | R.TLGSPIALVVENNDWK.H |  | 2 | 0.8177 | 100.380 | 0.947 | 878.47 | 1 | 2 | 10/30 | 9393 |
| 2023 | R.LGFYAPTAGQEASQIATHFALEK.E |  | 1 | 0.7635 | 29.250 | 0.980 | 817.42 | 0 | 3 | 15/44 | 9397 |
| 2024 | K.SFAGVDAFPIALNTNDVDK.I |  | 2 | 0.1967 | 59.700 | 0.000 | 997.50 | 0 | 2 | 13/36 | 9401 |
| 2025 | K.ELIFAILK.A |  | 1 | 0.4133 | 36.600 | 0.000 | 473.80 | 0 | 2 | 6/14 | 9413 |
| 2026 | K.TNVTLIFNANQALLAAR.A |  | 1 | 0.9600 | 76.590 | 0.923 | 915.52 | 0 | 2 | 23/32 | 9415 |
| 2027 | R.ELEAFAQFGSDLDQATQAK.L |  | 2 | 0.8915 | 108.830 | 0.978 | 1034.99 | 2 | 2 | 14/36 | 9417 |
| 2028 | K.ALSAFDKPVILLAGGLDR.G |  | 1 | 0.1078 | 39.160 | 0.000 | 619.36 | 0 | 3 | 15/34 | 9420 |
| 2029 | K.LLDYAEAGDNIGALLR.G |  | 7 | 0.2169 | 106.950 | 0.000 | 852.45 | 0 | 2 | 12/30 | 9421 |
| 2030 | K.QVPADLIEATEAFGSTTAQR.L |  | 1 | 0.2447 | 53.700 | 0.000 | 702.35 | 1 | 3 | 18/38 | 9428 |
| 2031 | R.ESASIFDLIEK.L |  | 1 | 0.8304 | 34.520 | 0.867 | 626.33 | 0 | 2 | 7/20 | 9432 |
| 2032 | R.HDVIDAVLESSELEPYSALHK.A |  | 1 | 0.6474 | 68.280 | 0.000 | 784.73 | 0 | 3 | 26/40 | 9434 |
| 2033 | K.GDVALFFGLSGTGK.T |  | 1 | 0.1189 | 60.110 | 0.000 | 684.86 | 0 | 2 | 10/26 | 9435 |
| 2034 | K.LPEYLGFFSGR.R |  | 1 | 0.4091 | 41.350 | 0.000 | 643.33 | 0 | 2 | 8/20 | 9437 |
| 2035 | R.VLDELWDEYPGLPEK.E |  | 1 | 0.9206 | 76.640 | 0.914 | 901.95 | 0 | 2 | 9/28 | 9447 |
| 2036 | K.GFDNFTVLLESEGK.Q |  | 1 | 0.4177 | 55.330 | 0.000 | 778.39 | 1 | 2 | 15/26 | 9455 |
| 2037 | K.KLTVLDAASYDFTAIDDSVK.G |  | 1 | 0.0866 | 84.630 | 0.000 | 1086.56 | 1 | 2 | 15/38 | 9462 |
| 2038 | K.LFDWIDSAVK.K |  | 1 | 0.8901 | 37.010 | 0.893 | 597.31 | 0 | 2 | 5/18 | 9466 |
| 2039 | K.AVLEEEQVPEVLSTFAAK.L |  | 2 | 0.5616 | 54.890 | 0.000 | 980.52 | 1 | 2 | 24/34 | 9473 |
| 2040 | R.SYLEALAEGAWENK.D |  | 1 | 0.3083 | 61.510 | 0.000 | 790.88 | 1 | 2 | 12/26 | 9474 |
| 2041 | R.LGANSLLSAIYGGM#VAGPNAVK.Y |  | 2 | 0.0834 | 46.400 | 0.000 | 707.05 | 0 | 3 | 12/42 | 9483 |
| 2042 | R.IDLYPDINEETLFQK.M |  | 2 | 0.5173 | 37.060 | 0.000 | 919.46 | 0 | 2 | 18/28 | 9485 |
| 2043 | K.TGIPIFAGGFIR.T |  | 1 | 0.7986 | 36.010 | 0.961 | 624.86 | 0 | 2 | 7/22 | 9487 |
| 2044 | R.NLGLDAYFAIPR.T |  | 1 | 0.4539 | 65.170 | 0.000 | 675.36 | 0 | 2 | 8/22 | 9490 |
| 2045 | K.NAAFIQDQLQFLER.T |  | 1 | 0.5911 | 63.990 | 0.000 | 564.96 | 0 | 3 | 10/26 | 9501 |
| 2046 | K.TGEWLPAETLDVIR.E |  | 2 | 0.2642 | 52.280 | 0.000 | 800.42 | 1 | 2 | 7/26 | 9507 |
| 2047 | R.NIPGVTVVEANGINVLDVVNHEK.L |  | 3 | 0.1760 | 68.620 | 0.000 | 1215.65 | 0 | 2 | 16/44 | 9509 |
| 2048 | R.DYFEGYPPSSPSFAILK.D |  | 1 | 0.2193 | 48.160 | 0.000 | 959.47 | 0 | 2 | 11/32 | 9511 |
| 2049 | K.DYETFHQEIEELQQENLQLK.K |  | 1 | 0.8359 | 44.670 | 0.000 | 845.41 | 1 | 3 | 20/38 | 9518 |
| 2050 | R.NWLFVLQR.V |  | 1 | 0.8826 | 31.310 | 0.614 | 538.31 | 1 | 2 | 6/14 | 9521 |
| 2051 | K.VTDLTTVQEYLLHEVQK.V |  | 1 | 0.4504 | 28.450 | 0.000 | 672.69 | 0 | 3 | 16/32 | 9529 |
| 2052 | K.EQGIPAEWIEAAQQSPIYK.M |  | 1 | 0.5469 | 45.400 | 0.000 | 720.03 | 1 | 3 | 18/36 | 9532 |
| 2053 | R.DNVSISDFEYLDLNK.T |  | 4 | 0.4866 | 86.910 | 0.000 | 886.42 | 1 | 2 | 12/28 | 9544 |
| 2054 | R.FAIIGTNWITDR.F |  | 1 | 0.3312 | 33.730 | 0.000 | 703.87 | 1 | 2 | 8/22 | 9545 |
| 2055 | R.GYDEDEVNEFLAQVR.K |  | 2 | 0.6927 | 70.640 | 0.000 | 892.41 | 0 | 2 | 18/28 | 9550 |
| 2056 | K.AFAELADVYVNDAFGAAHR.A |  | 3 | 0.1699 | 102.620 | 0.000 | 1018.99 | 1 | 2 | 11/36 | 9560 |
| 2057 | R.ISTVPSGSLALDTALGIGGYPR.G |  | 2 | 0.0420 | 61.560 | 0.000 | 1073.08 | 1 | 2 | 12/42 | 9561 |
| 2058 | R.QTLNIDPEDELILFSSETKK.G |  | 1 | 0.3660 | 31.370 | 0.000 | 774.07 | 0 | 3 | 21/38 | 9562 |
| 2059 | K.DVLGLTLLEK.T |  | 1 | 0.8714 | 61.270 | 0.823 | 550.83 | 0 | 2 | 8/18 | 9564 |
| 2060 | R.ELQVPVIALSQLSR.G |  | 1 | 0.2729 | 45.610 | 0.000 | 776.96 | 1 | 2 | 10/26 | 9577 |
| 2061 | R.FLASICEPYLEPFR.K |  | 1 | 0.9644 | 82.270 | 0.949 | 871.43 | 0 | 2 | 9/26 | 9586 |
| 2062 | K.GVIFTDILTAAR.E |  | 1 | 0.3731 | 84.660 | 0.000 | 638.87 | 0 | 2 | 9/22 | 9588 |
| 2063 | R.TAYAGATTGQQLLYALDEQVR.R |  | 2 | 0.1586 | 134.070 | 0.000 | 1135.08 | 0 | 2 | 14/40 | 9593 |
| 2064 | K.LGYPEEVYELLK.E |  | 1 | 0.9515 | 63.550 | 0.923 | 726.88 | 0 | 2 | 9/22 | 9600 |
| 2065 | R.DIFPAVLSLM#K.E |  | 2 | 0.9369 | 38.130 | 0.950 | 625.35 | 0 | 2 | 13/20 | 9602 |
| 2066 | R.LCIPQIDEEQVLEGLK.Q |  | 1 | 0.3749 | 73.590 | 0.000 | 942.49 | 0 | 2 | 9/30 | 9604 |
| 2067 | K.KPDSLPVLAELAAQEIR.S |  | 1 | 0.6064 | 25.310 | 0.961 | 617.35 | 1 | 3 | 11/32 | 9606 |
| 2068 | K.QPDTEQEWVTPGLLGDLAK.S |  | 1 | 0.1762 | 48.710 | 0.000 | 1049.03 | 0 | 2 | 14/36 | 9609 |
| 2069 | K.WTQDLDPNLNSILK.N |  | 1 | 0.0813 | 20.370 | 0.000 | 828.93 | 0 | 2 | 4/26 | 9612 |
| 2070 | K.QELVEVVEFLK.D |  | 1 | 0.5747 | 62.430 | 0.000 | 666.87 | 0 | 2 | 8/20 | 9614 |
| 2071 | R.NIGALAGLPYTVPAITVNR.Y |  | 2 | 0.7996 | 62.110 | 0.978 | 970.55 | 0 | 2 | 18/36 | 9618 |
| 2072 | R.CSYATSIEQLQEALVR.M |  | 1 | 0.4744 | 87.590 | 0.000 | 934.46 | 0 | 2 | 10/30 | 9621 |
| 2073 | K.IQLIGFGNFEVR.E |  | 2 | 0.4612 | 54.210 | 0.000 | 696.89 | 0 | 2 | 9/22 | 9624 |
| 2074 | R.TNVPNIYAIGDIIEGPPLAHK.A |  | 1 | 0.2628 | 31.630 | 0.000 | 744.74 | 0 | 3 | 20/40 | 9626 |
| 2075 | R.SVSLLPTTYIAIVPK.S |  | 1 | 0.0954 | 32.390 | 0.000 | 801.48 | 0 | 2 | 11/28 | 9627 |
| 2076 | R.IGGDQLDEDIVSFVR.K |  | 2 | 0.4013 | 90.460 | 0.000 | 831.92 | 0 | 2 | 13/28 | 9635 |
| 2077 | R.LNLIVAPENIVFDK.G |  | 1 | 0.2949 | 40.220 | 0.000 | 792.95 | 1 | 2 | 10/26 | 9644 |
| 2078 | K.HIYAQIIDDVNGVTLASASTLDK.D |  | 1 | 0.3466 | 68.440 | 0.000 | 815.43 | 0 | 3 | 20/44 | 9647 |
| 2079 | K.SLLSAIPLPDPDYER.N |  | 1 | 0.1352 | 35.890 | 0.000 | 843.44 | 2 | 2 | 6/28 | 9651 |
| 2080 | K.RDESIIQFIEQYK.E |  | 1 | 0.8936 | 34.630 | 0.738 | 556.96 | 1 | 3 | 11/24 | 9662 |
| 2081 | K.AEELGAIIVDPSKTDDVVAEIAER.T |  | 2 | 0.1508 | 38.410 | 0.000 | 847.44 | 0 | 3 | 19/46 | 9669 |
| 2082 | R.YSYAGGIPLETLIPK.E |  | 1 | 0.1403 | 41.820 | 0.000 | 811.45 | 1 | 2 | 8/28 | 9670 |
| 2083 | K.QVLCALSGGVDSSVVAVLIHK.A |  | 1 | 0.3699 | 53.660 | 0.000 | 718.07 | 1 | 3 | 24/40 | 9673 |
| 2084 | K.LNVLDSIEFVQSER.K |  | 2 | 0.9612 | 96.420 | 0.973 | 824.93 | 0 | 2 | 10/26 | 9681 |
| 2085 | K.DPTDLQLVHDIFR.A |  | 2 | 0.8349 | 63.430 | 0.000 | 784.91 | 0 | 2 | 17/24 | 9691 |
| 2086 | R.AINGEGPTLIETLTFR.Y |  | 2 | 0.2477 | 80.390 | 0.000 | 866.47 | 1 | 2 | 10/30 | 9695 |
| 2087 | R.VVAGVGVPQITAIYDCATEAR.K |  | 2 | 0.1601 | 84.980 | 0.000 | 1095.56 | 0 | 2 | 15/40 | 9713 |
| 2088 | K.LEDILDEYYEYGQQIK.K |  | 1 | 0.6426 | 72.010 | 0.000 | 1009.98 | 0 | 2 | 13/30 | 9723 |
| 2089 | R.AESIGQLSTDIFNIQTSDR.M |  | 1 | 0.3304 | 72.970 | 0.000 | 1048.02 | 1 | 2 | 12/36 | 9725 |
| 2090 | K.DLTFYEADLLDR.E |  | 2 | 0.6135 | 64.310 | 0.000 | 735.86 | 0 | 2 | 9/22 | 9728 |
| 2091 | R.ESGDLINDAQELISNHLQK.V |  | 1 | 0.7470 | 20.840 | 0.777 | 708.69 | 0 | 3 | 15/36 | 9730 |
| 2092 | R.FGSGWAWLVVNNGK.L |  | 2 | 0.1851 | 76.430 | 0.000 | 767.89 | 0 | 2 | 8/26 | 9734 |
| 2093 | K.TTVALHAIAEVQQQGGQAAFIDAEHALDPVYAQK.L |  | 1 | 0.1761 | 22.010 | 0.000 | 898.46 | 0 | 4 | 13/66 | 9739 |
| 2094 | K.ALIFDSLYDAYR.G |  | 1 | 0.5946 | 76.210 | 0.000 | 723.87 | 1 | 2 | 9/22 | 9740 |
| 2095 | R.LTADQLEM#QLR.L |  | 1 | 0.2711 | 21.710 | 0.000 | 667.35 | 6 | 2 | 5/20 | 9741 |
| 2096 | R.IIYDLVDVVEGR.C |  | 2 | 0.5474 | 87.120 | 0.000 | 695.88 | 1 | 2 | 10/22 | 9748 |
| 2097 | K.EGLTFDDVLLVPAK.S |  | 1 | 0.8333 | 56.160 | 0.927 | 758.92 | 0 | 2 | 8/26 | 9749 |
| 2098 | K.KPAALIILDGFGLR.N |  | 1 | 0.0712 | 30.110 | 0.000 | 495.30 | 0 | 3 | 5/26 | 9754 |
| 2099 | K.INLLDPDSVYDLGNGYK.V |  | 1 | 0.2319 | 61.410 | 0.000 | 948.47 | 0 | 2 | 10/32 | 9757 |
| 2100 | K.DINADLAAEALKDIIPSSKPK.V |  | 1 | 0.0268 | 27.980 | 0.000 | 553.06 | 0 | 4 | 7/40 | 9763 |
| 2101 | R.AYLDFLLR.L |  | 1 | 0.7702 | 53.790 | 0.000 | 505.79 | 0 | 2 | 7/14 | 9770 |
| 2102 | K.TFNIAGLQASAIIIPDR.L |  | 2 | 0.2550 | 71.740 | 0.000 | 900.50 | 0 | 2 | 12/32 | 9773 |
| 2103 | R.VLNGLGIAIISTSQGVLTDKEAR.A |  | 1 | 0.0366 | 47.620 | 0.000 | 785.78 | 0 | 3 | 11/44 | 9777 |
| 2104 | R.FGLIPEFIGR.L |  | 1 | 0.6707 | 32.510 | 0.624 | 574.83 | 0 | 2 | 7/18 | 9778 |
| 2105 | K.VLFIATANNLATIPGPLR.D |  | 1 | 0.0631 | 32.890 | 0.000 | 941.05 | 1 | 2 | 9/34 | 9793 |
| 2106 | R.TALFNYLFAR.N |  | 1 | 0.5760 | 49.760 | 0.000 | 608.33 | 0 | 2 | 8/18 | 9804 |
| 2107 | K.VFFDLGFVK.I |  | 1 | 0.9054 | 39.890 | 0.681 | 536.30 | 0 | 2 | 10/16 | 9806 |
| 2108 | K.VITTHLFDWFK.M |  | 1 | 0.9677 | 24.500 | 0.982 | 469.59 | 1 | 3 | 8/20 | 9808 |
| 2109 | R.LPFENGYAVFAGLEK.A |  | 1 | 0.9491 | 77.170 | 0.975 | 827.93 | 0 | 2 | 13/28 | 9811 |
| 2110 | R.DDQPIGVIPIDSIYTPVSR.V |  | 2 | 0.4364 | 60.170 | 0.000 | 1043.05 | 0 | 2 | 22/36 | 9817 |
| 2111 | R.ASHILVADKK.T |  | 1 | 0.2613 | 53.300 | 0.000 | 361.22 | 0 | 3 | 7/18 | 981 |
| 2112 | K.LVFNEILPESFPYM#NEPTK.S |  | 1 | 0.4344 | 21.190 | 0.000 | 762.04 | 1 | 3 | 14/36 | 9821 |
| 2113 | R.LDLPPFTLVGATTR.V |  | 1 | 0.8509 | 63.470 | 0.972 | 750.92 | 0 | 2 | 9/26 | 9827 |
| 2114 | K.VNQIGTLTETFDAIEM#AK.R |  | 2 | 0.3299 | 86.060 | 0.000 | 999.00 | 0 | 2 | 12/34 | 9828 |
| 2115 | R.AGIEITDICLQPLAAGSAALSK.D |  | 2 | 0.3367 | 63.970 | 0.000 | 733.73 | 0 | 3 | 22/42 | 9833 |
| 2116 | K.ESVEVNNFVPEYLTFDAEK.L |  | 2 | 0.3168 | 40.380 | 0.000 | 744.02 | 0 | 3 | 11/36 | 9834 |
| 2117 | R.QTGESTEISVDELSAFISK.Q |  | 1 | 0.0842 | 38.790 | 0.000 | 1021.00 | 0 | 2 | 8/36 | 9837 |
| 2118 | K.TLTSTLPVYLNALTGK.G |  | 2 | 0.1093 | 66.210 | 0.000 | 846.48 | 0 | 2 | 9/30 | 9841 |
| 2119 | R.ALGTELGIPDEIVWR.Q |  | 1 | 0.9210 | 46.290 | 0.987 | 834.95 | 0 | 2 | 16/28 | 9845 |
| 2120 | K.IYNAGTPEDVFTQPFFR.E |  | 1 | 0.3066 | 38.260 | 0.000 | 1001.49 | 1 | 2 | 10/32 | 9846 |
| 2121 | K.LADIAAPSILLGQAIGR.W |  | 2 | 0.2223 | 39.820 | 0.000 | 840.00 | 0 | 2 | 23/32 | 9848 |
| 2122 | R.VAAPDTVFPFSQAESVWLPNHK.D |  | 1 | 0.4702 | 27.340 | 0.000 | 814.08 | 0 | 3 | 21/42 | 9853 |
| 2123 | R.GASFSWYIYSPLR.V |  | 1 | 0.1827 | 44.270 | 0.000 | 773.89 | 0 | 2 | 6/24 | 9855 |
| 2124 | K.DLASILNAGALPVK.L |  | 2 | 0.2320 | 83.270 | 0.000 | 691.41 | 0 | 2 | 11/26 | 9864 |
| 2125 | K.AGELGLLGADVPEEYGGLGLDK.I |  | 1 | 0.0369 | 36.830 | 0.000 | 1087.05 | 0 | 2 | 10/42 | 9865 |
| 2126 | R.ETPVELEFTQIDKL.- |  | 1 | 0.7600 | 33.710 | 0.869 | 554.63 | 1 | 3 | 7/26 | 9871 |
| 2127 | K.TTLVDQLLHQAGTFR.A |  | 1 | 0.5805 | 43.250 | 0.000 | 567.31 | 1 | 3 | 16/28 | 9874 |
| 2128 | K.ALQYPILADPLSNLR.N |  | 1 | 0.5329 | 55.760 | 0.000 | 842.48 | 0 | 2 | 16/28 | 9878 |
| 2129 | R.GLIGYSTEFLSLTR.G |  | 1 | 0.4330 | 78.680 | 0.000 | 778.92 | 0 | 2 | 11/26 | 9886 |
| 2130 | R.WIYILSFK.D |  | 1 | 0.5059 | 32.960 | 0.000 | 535.31 | 0 | 2 | 5/14 | 9898 |
| 2131 | K.ICDQISDSILDEILKK.D |  | 1 | 0.9426 | 30.440 | 0.971 | 630.67 | 1 | 3 | 20/30 | 9900 |
| 2132 | R.LQFTDILGTIK.N |  | 1 | 0.5753 | 59.680 | 0.000 | 624.86 | 0 | 2 | 9/20 | 9902 |
| 2133 | K.AFGIDNLFDVK.H |  | 1 | 0.9024 | 50.550 | 0.948 | 619.82 | 0 | 2 | 6/20 | 9903 |
| 2134 | K.IDVQDLDCDFFALSSHK.M |  | 1 | 0.5393 | 56.320 | 0.000 | 670.65 | 0 | 3 | 11/32 | 9908 |
| 2135 | K.SLQYLLAQPAVASVITGASK.I |  | 2 | 0.0871 | 79.590 | 0.000 | 1009.07 | 1 | 2 | 14/38 | 9910 |
| 2136 | R.LVDNGDTVLVIEHNLDIIK.T |  | 1 | 0.2364 | 36.830 | 0.000 | 707.39 | 0 | 3 | 6/36 | 9920 |
| 2137 | K.IITTSDEILQELVNLKR.- |  | 1 | 0.1563 | 36.960 | 0.000 | 662.38 | 0 | 3 | 15/32 | 9932 |
| 2138 | K.NGIVYFVGAGPGDPGLLTIK.G |  | 1 | 0.0758 | 57.400 | 0.000 | 994.55 | 0 | 2 | 11/38 | 9938 |
| 2139 | K.NNEIAAAACYLPLSESPFISK.E |  | 2 | 0.4625 | 66.170 | 0.000 | 765.72 | 0 | 3 | 22/40 | 9943 |
| 2140 | R.NFSILDTADQLSVIK.G |  | 2 | 0.9610 | 100.300 | 0.923 | 832.45 | 0 | 2 | 20/28 | 9947 |
| 2141 | R.AASSAVVCDIVYNPIQTALLK.E |  | 2 | 0.3329 | 40.570 | 0.000 | 745.07 | 0 | 3 | 18/40 | 9948 |
| 2142 | R.ILGPSIFSPLPGTISVER.G |  | 1 | 0.1705 | 60.810 | 0.000 | 942.04 | 1 | 2 | 12/34 | 9951 |
| 2143 | R.LDTGNSFQGAVITPYYDSLLVK.L |  | 2 | 0.5420 | 33.880 | 0.000 | 801.08 | 0 | 3 | 24/42 | 9957 |
| 2144 | R.TVEDNAFLLQAISGVDK.M |  | 2 | 0.8190 | 111.500 | 0.945 | 910.48 | 0 | 2 | 11/32 | 9958 |
| 2145 | K.QGLISIIIPSYNEGYNVK.L |  | 1 | 0.7712 | 41.330 | 0.999 | 1004.54 | 1 | 2 | 11/34 | 9965 |
| 2146 | K.FSILDPVNTFTVPK.N |  | 1 | 0.9073 | 40.630 | 0.971 | 789.43 | 0 | 2 | 15/26 | 9968 |
| 2147 | K.NLLAYSHLYGELYDSLEK.D |  | 1 | 0.6996 | 46.070 | 0.000 | 710.02 | 0 | 3 | 16/34 | 9975 |
| 2148 | K.NGEPIQTGDLLLEIEKA.- |  | 1 | 0.2413 | 55.320 | 0.000 | 920.49 | 1 | 2 | 11/32 | 9977 |
| 2149 | K.GIEQFLADWNK.- |  | 2 | 0.4845 | 66.450 | 0.000 | 660.83 | 0 | 2 | 8/20 | 9983 |
| 2150 | K.FLADWFQLSK.E |  | 1 | 0.5259 | 25.860 | 0.000 | 627.83 | 0 | 2 | 12/18 | 9986 |
| 2151 | R.ESLLHILEAIR.A |  | 2 | 0.3483 | 49.360 | 0.000 | 647.38 | 0 | 2 | 8/20 | 9991 |

*#* Serial (sequential) number for peptide.

*Sequence* Assigned peptide sequence. Periods (".") delimit the sequence for internal peptides.

*PTM Site* Location of the post-translational modification (PTM) in the peptide.

*Nr. Scans* The number of MS/MS scans that were matched to this peptide.

*Logistic Score* The logistic probability score of this peptide/spectrum match.

*MOWSE Score* Score given to this peptide/spectrum match by Mascot.

*Delta Cn* Normalized gap between the highest and next-highest peptide matches.

*Isolated Mass* "Mass" in the Orbitrap analyzer.

*Delta Mass* Difference between the observed and theoretical masses, in ppm.

*Charge State* Inferred ionic state of the peptide.

*Matched Ions* Number of matched MS/MS ions, as reported by Mascot.

*Scan Nr.* MS/MS scan number in the original Xcalibur RAW file.
