# Supplementary material for: Data set of proteomic analysis of food borne pathogens after treatment with the disinfectants based on pyridoxal oxime derivatives
Source: Data Brief. 2017 Sep 29;15:738–41. doi: 10.1016/j.dib.2017.09.060 (PMC5671409; doi:10.1016/j.dib.2017.09.060)
Supplement: Supplementary file 3 — Supplementary material [file mmc3.docx]

RI NSF/EPSCoR Proteomics Facility

Mass Spectrometry Report

Protein Groups and Peptide-Spectrum Matches (PSMs)

Sample: Ecoli 11

Researcher: Djuro Josic

Results filtering: Unique PSMs; MOWSE Score > 0; 5 Proteins/Group max; Proteins from NCBI database

Input PSMs: 1727 'Target'; 0 'Decoy'; 0.0% FDR

Output PSMs: 1727

| **Protein IDs** | | | | | | | |
| --- | --- | --- | --- | --- | --- | --- | --- |
| *Grp Nr.* | *Accession Number* | *Protein Name* | *Protein Score* | *Unique PSMs* | *PSM Serial Nrs.* | *Other Grp.* | *Score (other)* |
| 1 | [91209071](http://www.ncbi.nlm.nih.gov/entrez/query.fcgi?cmd=Search&db=Protein&term=91209071&doptcmdl=GenPept) | chaperone Hsp70; DNA biosynthesis; autoregulated heat shock proteins [Escherichia coli UTI89] | 2256.25 | 39 | 6 75 112 117 190 232 242 243 255 259 264 270 297 305 326 358 465 482 488 578 650 876 916 1043 1052 1076 1150 1155 1164 1183 1254 1260 1268 1371 1393 1415 1420 1633 1712 |  |  |
|  | [91070645](http://www.ncbi.nlm.nih.gov/entrez/query.fcgi?cmd=Search&db=Protein&term=91070645&doptcmdl=GenPept) | chaperone Hsp70; DNA biosynthesis; autoregulated heat shock proteins [Escherichia coli UTI89] | 2256.25 | 39 | 6 75 112 117 190 232 242 243 255 259 264 270 297 305 326 358 465 482 488 578 650 876 916 1043 1052 1076 1150 1155 1164 1183 1254 1260 1268 1371 1393 1415 1420 1633 1712 |  |  |
|  | [90110254](http://www.ncbi.nlm.nih.gov/entrez/query.fcgi?cmd=Search&db=Protein&term=90110254&doptcmdl=GenPept) | Chaperone protein dnaK (Heat shock protein 70) (Heat shock 70 kDa protein) (HSP70) | 2256.25 | 39 | 6 75 112 117 190 232 242 243 255 259 264 270 297 305 326 358 465 482 488 578 650 876 916 1043 1052 1076 1150 1155 1164 1183 1254 1260 1268 1371 1393 1415 1420 1633 1712 |  |  |
|  | [90110253](http://www.ncbi.nlm.nih.gov/entrez/query.fcgi?cmd=Search&db=Protein&term=90110253&doptcmdl=GenPept) | Chaperone protein dnaK (Heat shock protein 70) (Heat shock 70 kDa protein) (HSP70) | 2256.25 | 39 | 6 75 112 117 190 232 242 243 255 259 264 270 297 305 326 358 465 482 488 578 650 876 916 1043 1052 1076 1150 1155 1164 1183 1254 1260 1268 1371 1393 1415 1420 1633 1712 |  |  |
|  | [89106898](http://www.ncbi.nlm.nih.gov/entrez/query.fcgi?cmd=Search&db=Protein&term=89106898&doptcmdl=GenPept) | chaperone Hsp70, co-chaperone with DnaJ [Escherichia coli W3110] | 2256.25 | 39 | 6 75 112 117 190 232 242 243 255 259 264 270 297 305 326 358 465 482 488 578 650 876 916 1043 1052 1076 1150 1155 1164 1183 1254 1260 1268 1371 1393 1415 1420 1633 1712 |  |  |
| 2 | [91211926](http://www.ncbi.nlm.nih.gov/entrez/query.fcgi?cmd=Search&db=Protein&term=91211926&doptcmdl=GenPept) | protein disaggregation chaperone [Escherichia coli UTI89] | 1329.57 | 24 | 165 206 309 439 485 494 612 664 682 736 786 859 894 963 985 1134 1232 1313 1358 1449 1580 1605 1652 1688 |  |  |
|  | [91073500](http://www.ncbi.nlm.nih.gov/entrez/query.fcgi?cmd=Search&db=Protein&term=91073500&doptcmdl=GenPept) | heat shock protein [Escherichia coli UTI89] | 1329.57 | 24 | 165 206 309 439 485 494 612 664 682 736 786 859 894 963 985 1134 1232 1313 1358 1449 1580 1605 1652 1688 |  |  |
|  | [89109394](http://www.ncbi.nlm.nih.gov/entrez/query.fcgi?cmd=Search&db=Protein&term=89109394&doptcmdl=GenPept) | protein disaggregation chaperone [Escherichia coli W3110] | 1329.57 | 24 | 165 206 309 439 485 494 612 664 682 736 786 859 894 963 985 1134 1232 1313 1358 1449 1580 1605 1652 1688 |  |  |
|  | [83585276](http://www.ncbi.nlm.nih.gov/entrez/query.fcgi?cmd=Search&db=Protein&term=83585276&doptcmdl=GenPept) | COG0542: ATPases with chaperone activity, ATP-binding subunit [Escherichia coli 101-1] | 1329.57 | 24 | 165 206 309 439 485 494 612 664 682 736 786 859 894 963 985 1134 1232 1313 1358 1449 1580 1605 1652 1688 |  |  |
|  | [82545045](http://www.ncbi.nlm.nih.gov/entrez/query.fcgi?cmd=Search&db=Protein&term=82545045&doptcmdl=GenPept) | protein disaggregation chaperone [Shigella boydii Sb227] | 1329.57 | 24 | 165 206 309 439 485 494 612 664 682 736 786 859 894 963 985 1134 1232 1313 1358 1449 1580 1605 1652 1688 |  |  |
| 3 | [RS1_ECOLI](http://us.expasy.org/uniprot/RS1_ECOLI) | 30S ribosomal protein S1 | 1290.79 | 23 | 162 217 241 280 313 378 523 531 571 589 797 822 884 1042 1108 1212 1220 1258 1286 1316 1436 1493 1687 |  |  |
|  | [91210012](http://www.ncbi.nlm.nih.gov/entrez/query.fcgi?cmd=Search&db=Protein&term=91210012&doptcmdl=GenPept) | 30S ribosomal protein S1 [Escherichia coli UTI89] | 1226.89 | 22 | 162 217 241 280 313 378 523 531 571 589 797 822 884 1042 1108 1212 1220 1258 1286 1316 1436 1493 |  |  |
| 4 | [91212806](http://www.ncbi.nlm.nih.gov/entrez/query.fcgi?cmd=Search&db=Protein&term=91212806&doptcmdl=GenPept) | DNA-directed RNA polymerase subunit beta [Escherichia coli UTI89] | 1274.71 | 23 | 70 81 88 134 189 336 424 506 596 603 670 681 698 800 805 842 1099 1321 1336 1353 1413 1453 1603 |  |  |
|  | [91074380](http://www.ncbi.nlm.nih.gov/entrez/query.fcgi?cmd=Search&db=Protein&term=91074380&doptcmdl=GenPept) | RNA polymerase beta subunit [Escherichia coli UTI89] | 1274.71 | 23 | 70 81 88 134 189 336 424 506 596 603 670 681 698 800 805 842 1099 1321 1336 1353 1413 1453 1603 |  |  |
|  | [89110052](http://www.ncbi.nlm.nih.gov/entrez/query.fcgi?cmd=Search&db=Protein&term=89110052&doptcmdl=GenPept) | RNA polymerase, beta subunit [Escherichia coli W3110] | 1274.71 | 23 | 70 81 88 134 189 336 424 506 596 603 670 681 698 800 805 842 1099 1321 1336 1353 1413 1453 1603 |  |  |
|  | [88909639](http://www.ncbi.nlm.nih.gov/entrez/query.fcgi?cmd=Search&db=Protein&term=88909639&doptcmdl=GenPept) | DNA-directed RNA polymerase subunit beta (RNAP subunit beta) (Transcriptase subunit beta) (RNA polymerase subunit beta) | 1274.71 | 23 | 70 81 88 134 189 336 424 506 596 603 670 681 698 800 805 842 1099 1321 1336 1353 1413 1453 1603 |  |  |
|  | [88909637](http://www.ncbi.nlm.nih.gov/entrez/query.fcgi?cmd=Search&db=Protein&term=88909637&doptcmdl=GenPept) | DNA-directed RNA polymerase subunit beta (RNAP subunit beta) (Transcriptase subunit beta) (RNA polymerase subunit beta) | 1274.71 | 23 | 70 81 88 134 189 336 424 506 596 603 670 681 698 800 805 842 1099 1321 1336 1353 1413 1453 1603 |  |  |
| 5 | [91209547](http://www.ncbi.nlm.nih.gov/entrez/query.fcgi?cmd=Search&db=Protein&term=91209547&doptcmdl=GenPept) | heat shock protein 90 [Escherichia coli UTI89] | 1164.96 | 20 | 35 188 353 392 484 613 617 632 707 775 827 886 924 926 928 935 1003 1322 1326 1361 |  |  |
|  | [91071121](http://www.ncbi.nlm.nih.gov/entrez/query.fcgi?cmd=Search&db=Protein&term=91071121&doptcmdl=GenPept) | chaperone Hsp90, heat shock protein C 62.5 [Escherichia coli UTI89] | 1164.96 | 20 | 35 188 353 392 484 613 617 632 707 775 827 886 924 926 928 935 1003 1322 1326 1361 |  |  |
|  | [89107342](http://www.ncbi.nlm.nih.gov/entrez/query.fcgi?cmd=Search&db=Protein&term=89107342&doptcmdl=GenPept) | molecular chaperone HSP90 family [Escherichia coli W3110] | 1164.96 | 20 | 35 188 353 392 484 613 617 632 707 775 827 886 924 926 928 935 1003 1322 1326 1361 |  |  |
|  | [88909153](http://www.ncbi.nlm.nih.gov/entrez/query.fcgi?cmd=Search&db=Protein&term=88909153&doptcmdl=GenPept) | Chaperone protein htpG (Heat shock protein htpG) (High temperature protein G) | 1164.96 | 20 | 35 188 353 392 484 613 617 632 707 775 827 886 924 926 928 935 1003 1322 1326 1361 |  |  |
|  | [88909151](http://www.ncbi.nlm.nih.gov/entrez/query.fcgi?cmd=Search&db=Protein&term=88909151&doptcmdl=GenPept) | Chaperone protein htpG (Heat shock protein htpG) (High temperature protein G) | 1164.96 | 20 | 35 188 353 392 484 613 617 632 707 775 827 886 924 926 928 935 1003 1322 1326 1361 |  |  |
| 6 | [91210463](http://www.ncbi.nlm.nih.gov/entrez/query.fcgi?cmd=Search&db=Protein&term=91210463&doptcmdl=GenPept) | CoA-linked acetaldehyde dehydrogenase [Escherichia coli UTI89] | 1077.96 | 20 | 236 400 401 481 546 599 621 660 782 955 1057 1080 1124 1156 1180 1308 1363 1460 1568 1644 |  |  |
|  | [91072037](http://www.ncbi.nlm.nih.gov/entrez/query.fcgi?cmd=Search&db=Protein&term=91072037&doptcmdl=GenPept) | CoA-linked acetaldehyde dehydrogenase [Escherichia coli UTI89] | 1077.96 | 20 | 236 400 401 481 546 599 621 660 782 955 1057 1080 1124 1156 1180 1308 1363 1460 1568 1644 |  |  |
|  | [89108085](http://www.ncbi.nlm.nih.gov/entrez/query.fcgi?cmd=Search&db=Protein&term=89108085&doptcmdl=GenPept) | fused acetaldehyde-CoA dehydrogenase and iron-dependent alcohol dehydrogenase and pyruvate-formate lyase deactivase [Escherichia coli W3110] | 1077.96 | 20 | 236 400 401 481 546 599 621 660 782 955 1057 1080 1124 1156 1180 1308 1363 1460 1568 1644 |  |  |
|  | [83587379](http://www.ncbi.nlm.nih.gov/entrez/query.fcgi?cmd=Search&db=Protein&term=83587379&doptcmdl=GenPept) | COG1012: NAD-dependent aldehyde dehydrogenases [Escherichia coli 101-1] | 1077.96 | 20 | 236 400 401 481 546 599 621 660 782 955 1057 1080 1124 1156 1180 1308 1363 1460 1568 1644 |  |  |
|  | [82776584](http://www.ncbi.nlm.nih.gov/entrez/query.fcgi?cmd=Search&db=Protein&term=82776584&doptcmdl=GenPept) | CoA-linked acetaldehyde dehydrogenase and iron-dependent alcohol dehydrogenase/pyruvate-formate-lyase deactivase [Shigella dysenteriae Sd197] | 1077.96 | 20 | 236 400 401 481 546 599 621 660 782 955 1057 1080 1124 1156 1180 1308 1363 1460 1568 1644 |  |  |
| 7 | [91210004](http://www.ncbi.nlm.nih.gov/entrez/query.fcgi?cmd=Search&db=Protein&term=91210004&doptcmdl=GenPept) | formate acetyltransferase 1 [Escherichia coli UTI89] | 1071.04 | 19 | 8 129 258 541 597 667 686 717 742 1048 1207 1211 1319 1376 1533 1626 1629 1646 1668 |  |  |
|  | [91071578](http://www.ncbi.nlm.nih.gov/entrez/query.fcgi?cmd=Search&db=Protein&term=91071578&doptcmdl=GenPept) | formate acetyltransferase 1 [Escherichia coli UTI89] | 1071.04 | 19 | 8 129 258 541 597 667 686 717 742 1048 1207 1211 1319 1376 1533 1626 1629 1646 1668 |  |  |
|  | [89107753](http://www.ncbi.nlm.nih.gov/entrez/query.fcgi?cmd=Search&db=Protein&term=89107753&doptcmdl=GenPept) | pyruvate formate lyase I [Escherichia coli W3110] | 1071.04 | 19 | 8 129 258 541 597 667 686 717 742 1048 1207 1211 1319 1376 1533 1626 1629 1646 1668 |  |  |
|  | [8569374](http://www.ncbi.nlm.nih.gov/entrez/query.fcgi?cmd=Search&db=Protein&term=8569374&doptcmdl=GenPept) | Chain B, Crystal Structure Of Pfl From E.Coli In Complex With Substrate Analogue Oxamate | 1071.04 | 19 | 8 129 258 541 597 667 686 717 742 1048 1207 1211 1319 1376 1533 1626 1629 1646 1668 |  |  |
|  | [8569373](http://www.ncbi.nlm.nih.gov/entrez/query.fcgi?cmd=Search&db=Protein&term=8569373&doptcmdl=GenPept) | Chain A, Crystal Structure Of Pfl From E.Coli In Complex With Substrate Analogue Oxamate | 1071.04 | 19 | 8 129 258 541 597 667 686 717 742 1048 1207 1211 1319 1376 1533 1626 1629 1646 1668 |  |  |
| 8 | [91212816](http://www.ncbi.nlm.nih.gov/entrez/query.fcgi?cmd=Search&db=Protein&term=91212816&doptcmdl=GenPept) | elongation factor G [Escherichia coli UTI89] | 1014.31 | 15 | 92 693 696 771 960 991 1035 1079 1125 1167 1206 1394 1418 1444 1674 |  |  |
|  | [91074390](http://www.ncbi.nlm.nih.gov/entrez/query.fcgi?cmd=Search&db=Protein&term=91074390&doptcmdl=GenPept) | translation elongation factor EF-G GTP-binding protein chain [Escherichia coli UTI89] | 1014.31 | 15 | 92 693 696 771 960 991 1035 1079 1125 1167 1206 1394 1418 1444 1674 |  |  |
|  | [90110712](http://www.ncbi.nlm.nih.gov/entrez/query.fcgi?cmd=Search&db=Protein&term=90110712&doptcmdl=GenPept) | Elongation factor G (EF-G) | 1014.31 | 15 | 92 693 696 771 960 991 1035 1079 1125 1167 1206 1394 1418 1444 1674 |  |  |
|  | [90110711](http://www.ncbi.nlm.nih.gov/entrez/query.fcgi?cmd=Search&db=Protein&term=90110711&doptcmdl=GenPept) | Elongation factor G (EF-G) | 1014.31 | 15 | 92 693 696 771 960 991 1035 1079 1125 1167 1206 1394 1418 1444 1674 |  |  |
|  | [90110710](http://www.ncbi.nlm.nih.gov/entrez/query.fcgi?cmd=Search&db=Protein&term=90110710&doptcmdl=GenPept) | Elongation factor G (EF-G) | 1014.31 | 15 | 92 693 696 771 960 991 1035 1079 1125 1167 1206 1394 1418 1444 1674 |  |  |
| 9 | [99031714](http://www.ncbi.nlm.nih.gov/entrez/query.fcgi?cmd=Search&db=Protein&term=99031714&doptcmdl=GenPept) | Chain A, Crystal Structure Of The E. Coli Phosphoglycerate Kinase | 999.68 | 14 | 53 84 114 246 475 737 794 883 947 1069 1314 1366 1512 1708 |  |  |
|  | [91212302](http://www.ncbi.nlm.nih.gov/entrez/query.fcgi?cmd=Search&db=Protein&term=91212302&doptcmdl=GenPept) | phosphoglycerate kinase [Escherichia coli UTI89] | 999.68 | 14 | 53 84 114 246 475 737 794 883 947 1069 1314 1366 1512 1708 |  |  |
|  | [91073876](http://www.ncbi.nlm.nih.gov/entrez/query.fcgi?cmd=Search&db=Protein&term=91073876&doptcmdl=GenPept) | phosphoglycerate kinase [Escherichia coli UTI89] [MASS=43297] | 999.68 | 14 | 53 84 114 246 475 737 794 883 947 1069 1314 1366 1512 1708 |  |  |
|  | [89109704](http://www.ncbi.nlm.nih.gov/entrez/query.fcgi?cmd=Search&db=Protein&term=89109704&doptcmdl=GenPept) | phosphoglycerate kinase [Escherichia coli W3110] | 999.68 | 14 | 53 84 114 246 475 737 794 883 947 1069 1314 1366 1512 1708 |  |  |
|  | [882455](http://www.ncbi.nlm.nih.gov/entrez/query.fcgi?cmd=Search&db=Protein&term=882455&doptcmdl=GenPept) | phosphoglycerate kinase | 999.68 | 14 | 53 84 114 246 475 737 794 883 947 1069 1314 1366 1512 1708 |  |  |
| 10 | [91209847](http://www.ncbi.nlm.nih.gov/entrez/query.fcgi?cmd=Search&db=Protein&term=91209847&doptcmdl=GenPept) | DNA starvation/stationary phase protection protein Dps [Escherichia coli UTI89] | 997.30 | 15 | 40 201 245 250 267 365 402 521 533 684 702 766 870 879 1123 |  |  |
|  | [91071421](http://www.ncbi.nlm.nih.gov/entrez/query.fcgi?cmd=Search&db=Protein&term=91071421&doptcmdl=GenPept) | stationary phase nucleoid protein Dps [Escherichia coli UTI89] | 997.30 | 15 | 40 201 245 250 267 365 402 521 533 684 702 766 870 879 1123 |  |  |
|  | [89107663](http://www.ncbi.nlm.nih.gov/entrez/query.fcgi?cmd=Search&db=Protein&term=89107663&doptcmdl=GenPept) | Fe-binding and storage protein [Escherichia coli W3110] | 997.30 | 15 | 40 201 245 250 267 365 402 521 533 684 702 766 870 879 1123 |  |  |
|  | [83586115](http://www.ncbi.nlm.nih.gov/entrez/query.fcgi?cmd=Search&db=Protein&term=83586115&doptcmdl=GenPept) | COG0783: DNA-binding ferritin-like protein (oxidative damage protectant) [Escherichia coli 101-1] | 997.30 | 15 | 40 201 245 250 267 365 402 521 533 684 702 766 870 879 1123 |  |  |
|  | [82776108](http://www.ncbi.nlm.nih.gov/entrez/query.fcgi?cmd=Search&db=Protein&term=82776108&doptcmdl=GenPept) | DNA starvation/stationary phase protection protein Dps [Shigella dysenteriae Sd197] | 997.30 | 15 | 40 201 245 250 267 365 402 521 533 684 702 766 870 879 1123 |  |  |
| 11 | [91213693](http://www.ncbi.nlm.nih.gov/entrez/query.fcgi?cmd=Search&db=Protein&term=91213693&doptcmdl=GenPept) | chaperonin GroEL [Escherichia coli UTI89] | 937.03 | 15 | 48 247 263 269 315 357 404 487 516 550 770 1006 1247 1252 1682 |  |  |
|  | [91075267](http://www.ncbi.nlm.nih.gov/entrez/query.fcgi?cmd=Search&db=Protein&term=91075267&doptcmdl=GenPept) | GroEL (chaperone Hsp60) [Escherichia coli UTI89] | 937.03 | 15 | 48 247 263 269 315 357 404 487 516 550 770 1006 1247 1252 1682 |  |  |
|  | [89110864](http://www.ncbi.nlm.nih.gov/entrez/query.fcgi?cmd=Search&db=Protein&term=89110864&doptcmdl=GenPept) | Cpn60 chaperonin GroEL, large subunit of GroESL [Escherichia coli W3110] | 937.03 | 15 | 48 247 263 269 315 357 404 487 516 550 770 1006 1247 1252 1682 |  |  |
|  | [85676895](http://www.ncbi.nlm.nih.gov/entrez/query.fcgi?cmd=Search&db=Protein&term=85676895&doptcmdl=GenPept) | Cpn60 chaperonin GroEL, large subunit of GroESL [Escherichia coli W3110] | 937.03 | 15 | 48 247 263 269 315 357 404 487 516 550 770 1006 1247 1252 1682 |  |  |
|  | [83587266](http://www.ncbi.nlm.nih.gov/entrez/query.fcgi?cmd=Search&db=Protein&term=83587266&doptcmdl=GenPept) | COG0459: Chaperonin GroEL (HSP60 family) [Escherichia coli 101-1] | 937.03 | 15 | 48 247 263 269 315 357 404 487 516 550 770 1006 1247 1252 1682 |  |  |
| 12 | [93279787](http://www.ncbi.nlm.nih.gov/entrez/query.fcgi?cmd=Search&db=Protein&term=93279787&doptcmdl=GenPept) | Chain A, Crystal Structure Determination Of E. Coli Elongation Factor, Tu Using A Twinned Data Set | 811.54 | 16 | 56 143 252 278 298 549 639 768 932 1039 1161 1179 1310 1427 1525 1723 |  |  |
|  | [91212777](http://www.ncbi.nlm.nih.gov/entrez/query.fcgi?cmd=Search&db=Protein&term=91212777&doptcmdl=GenPept) | elongation factor Tu [Escherichia coli UTI89] | 811.54 | 16 | 56 143 252 278 298 549 639 768 932 1039 1161 1179 1310 1427 1525 1723 |  |  |
|  | [91074389](http://www.ncbi.nlm.nih.gov/entrez/query.fcgi?cmd=Search&db=Protein&term=91074389&doptcmdl=GenPept) | translation elongation factor EF-Tu [Escherichia coli UTI89] [MASS=44880] | 811.54 | 16 | 56 143 252 278 298 549 639 768 932 1039 1161 1179 1310 1427 1525 1723 |  |  |
|  | [91074351](http://www.ncbi.nlm.nih.gov/entrez/query.fcgi?cmd=Search&db=Protein&term=91074351&doptcmdl=GenPept) | protein chain elongation factor EF-Tu (duplicate of tufB) [Escherichia coli UTI89] | 811.54 | 16 | 56 143 252 278 298 549 639 768 932 1039 1161 1179 1310 1427 1525 1723 |  |  |
|  | [89110671](http://www.ncbi.nlm.nih.gov/entrez/query.fcgi?cmd=Search&db=Protein&term=89110671&doptcmdl=GenPept) | protein chain elongation factor EF-Tu [Escherichia coli W3110] | 811.54 | 16 | 56 143 252 278 298 549 639 768 932 1039 1161 1179 1310 1427 1525 1723 |  |  |
| 13 | [91210996](http://www.ncbi.nlm.nih.gov/entrez/query.fcgi?cmd=Search&db=Protein&term=91210996&doptcmdl=GenPept) | glyceraldehyde-3-phosphate dehydrogenase A [Escherichia coli UTI89] | 804.33 | 14 | 256 257 261 279 308 420 440 505 579 921 1031 1175 1215 1514 |  |  |
|  | [91072570](http://www.ncbi.nlm.nih.gov/entrez/query.fcgi?cmd=Search&db=Protein&term=91072570&doptcmdl=GenPept) | glyceraldehyde-3-phosphate dehydrogenase A [Escherichia coli UTI89] | 804.33 | 14 | 256 257 261 279 308 420 440 505 579 921 1031 1175 1215 1514 |  |  |
|  | [89108618](http://www.ncbi.nlm.nih.gov/entrez/query.fcgi?cmd=Search&db=Protein&term=89108618&doptcmdl=GenPept) | glyceraldehyde-3-phosphate dehydrogenase A [Escherichia coli W3110] | 804.33 | 14 | 256 257 261 279 308 420 440 505 579 921 1031 1175 1215 1514 |  |  |
|  | [83586079](http://www.ncbi.nlm.nih.gov/entrez/query.fcgi?cmd=Search&db=Protein&term=83586079&doptcmdl=GenPept) | COG0057: Glyceraldehyde-3-phosphate dehydrogenase/erythrose-4-phosphate dehydrogenase [Escherichia coli 101-1] | 804.33 | 14 | 256 257 261 279 308 420 440 505 579 921 1031 1175 1215 1514 |  |  |
|  | [83569614](http://www.ncbi.nlm.nih.gov/entrez/query.fcgi?cmd=Search&db=Protein&term=83569614&doptcmdl=GenPept) | COG0057: Glyceraldehyde-3-phosphate dehydrogenase/erythrose-4-phosphate dehydrogenase [Shigella dysenteriae 1012] | 804.33 | 14 | 256 257 261 279 308 420 440 505 579 921 1031 1175 1215 1514 |  |  |
| 14 | [91213688](http://www.ncbi.nlm.nih.gov/entrez/query.fcgi?cmd=Search&db=Protein&term=91213688&doptcmdl=GenPept) | aspartate ammonia-lyase [Escherichia coli UTI89] | 797.81 | 14 | 28 125 512 544 756 757 814 867 901 1025 1102 1186 1257 1543 |  |  |
|  | [91075262](http://www.ncbi.nlm.nih.gov/entrez/query.fcgi?cmd=Search&db=Protein&term=91075262&doptcmdl=GenPept) | aspartate ammonia-lyase (aspartase) [Escherichia coli UTI89] | 797.81 | 14 | 28 125 512 544 756 757 814 867 901 1025 1102 1186 1257 1543 |  |  |
|  | [90111690](http://www.ncbi.nlm.nih.gov/entrez/query.fcgi?cmd=Search&db=Protein&term=90111690&doptcmdl=GenPept) | aspartate ammonia-lyase [Escherichia coli str. K-12 substr. MG1655] | 797.81 | 14 | 28 125 512 544 756 757 814 867 901 1025 1102 1186 1257 1543 |  |  |
|  | [89110860](http://www.ncbi.nlm.nih.gov/entrez/query.fcgi?cmd=Search&db=Protein&term=89110860&doptcmdl=GenPept) | aspartate ammonia-lyase [Escherichia coli W3110] | 797.81 | 14 | 28 125 512 544 756 757 814 867 901 1025 1102 1186 1257 1543 |  |  |
|  | [87082375](http://www.ncbi.nlm.nih.gov/entrez/query.fcgi?cmd=Search&db=Protein&term=87082375&doptcmdl=GenPept) | aspartate ammonia-lyase [Escherichia coli str. K-12 substr. MG1655] | 797.81 | 14 | 28 125 512 544 756 757 814 867 901 1025 1102 1186 1257 1543 |  |  |
| 15 | [91209509](http://www.ncbi.nlm.nih.gov/entrez/query.fcgi?cmd=Search&db=Protein&term=91209509&doptcmdl=GenPept) | trigger factor [Escherichia coli UTI89] | 772.54 | 15 | 57 176 479 534 760 774 903 1302 1385 1432 1434 1468 1553 1676 1721 |  |  |
|  | [91071083](http://www.ncbi.nlm.nih.gov/entrez/query.fcgi?cmd=Search&db=Protein&term=91071083&doptcmdl=GenPept) | trigger factor [Escherichia coli UTI89] | 772.54 | 15 | 57 176 479 534 760 774 903 1302 1385 1432 1434 1468 1553 1676 1721 |  |  |
|  | [89107306](http://www.ncbi.nlm.nih.gov/entrez/query.fcgi?cmd=Search&db=Protein&term=89107306&doptcmdl=GenPept) | peptidyl-prolyl cis/trans isomerase [Escherichia coli W3110] | 772.54 | 15 | 57 176 479 534 760 774 903 1302 1385 1432 1434 1468 1553 1676 1721 |  |  |
|  | [85674576](http://www.ncbi.nlm.nih.gov/entrez/query.fcgi?cmd=Search&db=Protein&term=85674576&doptcmdl=GenPept) | peptidyl-prolyl cis/trans isomerase [Escherichia coli W3110] | 772.54 | 15 | 57 176 479 534 760 774 903 1302 1385 1432 1434 1468 1553 1676 1721 |  |  |
|  | [83584465](http://www.ncbi.nlm.nih.gov/entrez/query.fcgi?cmd=Search&db=Protein&term=83584465&doptcmdl=GenPept) | COG0544: FKBP-type peptidyl-prolyl cis-trans isomerase (trigger factor) [Escherichia coli 101-1] | 772.54 | 15 | 57 176 479 534 760 774 903 1302 1385 1432 1434 1468 1553 1676 1721 |  |  |
| 16 | [91213257](http://www.ncbi.nlm.nih.gov/entrez/query.fcgi?cmd=Search&db=Protein&term=91213257&doptcmdl=GenPept) | F0F1 ATP synthase subunit beta [Escherichia coli UTI89] | 759.28 | 12 | 33 96 606 837 845 869 1066 1275 1338 1448 1486 1702 |  |  |
|  | [91074831](http://www.ncbi.nlm.nih.gov/entrez/query.fcgi?cmd=Search&db=Protein&term=91074831&doptcmdl=GenPept) | membrane-bound ATP synthase F1 sector beta-subunit [Escherichia coli UTI89] | 759.28 | 12 | 33 96 606 837 845 869 1066 1275 1338 1448 1486 1702 |  |  |
|  | [899257](http://www.ncbi.nlm.nih.gov/entrez/query.fcgi?cmd=Search&db=Protein&term=899257&doptcmdl=GenPept) | unnamed protein product [Escherichia coli] | 759.28 | 12 | 33 96 606 837 845 869 1066 1275 1338 1448 1486 1702 |  |  |
|  | [89110275](http://www.ncbi.nlm.nih.gov/entrez/query.fcgi?cmd=Search&db=Protein&term=89110275&doptcmdl=GenPept) | F1 sector of membrane-bound ATP synthase, beta subunit [Escherichia coli W3110] | 759.28 | 12 | 33 96 606 837 845 869 1066 1275 1338 1448 1486 1702 |  |  |
|  | [85676306](http://www.ncbi.nlm.nih.gov/entrez/query.fcgi?cmd=Search&db=Protein&term=85676306&doptcmdl=GenPept) | F1 sector of membrane-bound ATP synthase, beta subunit [Escherichia coli W3110] | 759.28 | 12 | 33 96 606 837 845 869 1066 1275 1338 1448 1486 1702 |  |  |
| 17 | [91212591](http://www.ncbi.nlm.nih.gov/entrez/query.fcgi?cmd=Search&db=Protein&term=91212591&doptcmdl=GenPept) | transcription elongation factor NusA [Escherichia coli UTI89] | 733.73 | 13 | 118 339 375 454 497 582 627 1208 1357 1502 1592 1624 1689 |  |  |
|  | [91074165](http://www.ncbi.nlm.nih.gov/entrez/query.fcgi?cmd=Search&db=Protein&term=91074165&doptcmdl=GenPept) | transcription pausing; L factor [Escherichia coli UTI89] | 733.73 | 13 | 118 339 375 454 497 582 627 1208 1357 1502 1592 1624 1689 |  |  |
|  | [89109934](http://www.ncbi.nlm.nih.gov/entrez/query.fcgi?cmd=Search&db=Protein&term=89109934&doptcmdl=GenPept) | transcription termination/antitermination L factor [Escherichia coli W3110] | 733.73 | 13 | 118 339 375 454 497 582 627 1208 1357 1502 1592 1624 1689 |  |  |
|  | [85675965](http://www.ncbi.nlm.nih.gov/entrez/query.fcgi?cmd=Search&db=Protein&term=85675965&doptcmdl=GenPept) | transcription termination/antitermination L factor [Escherichia coli W3110] | 733.73 | 13 | 118 339 375 454 497 582 627 1208 1357 1502 1592 1624 1689 |  |  |
|  | [84028776](http://www.ncbi.nlm.nih.gov/entrez/query.fcgi?cmd=Search&db=Protein&term=84028776&doptcmdl=GenPept) | Transcription elongation protein nusA (N utilization substance protein A) (L factor) | 733.73 | 13 | 118 339 375 454 497 582 627 1208 1357 1502 1592 1624 1689 |  |  |
| 18 | [91075253](http://www.ncbi.nlm.nih.gov/entrez/query.fcgi?cmd=Search&db=Protein&term=91075253&doptcmdl=GenPept) | lysyl-tRNA synthetase, heat inducible [Escherichia coli UTI89] [MASS=58831] | 706.90 | 16 | 115 372 381 492 588 665 888 915 979 995 1053 1127 1354 1368 1425 1647 |  |  |
|  | [89110851](http://www.ncbi.nlm.nih.gov/entrez/query.fcgi?cmd=Search&db=Protein&term=89110851&doptcmdl=GenPept) | lysine tRNA synthetase, inducible [Escherichia coli W3110] | 706.90 | 16 | 115 372 381 492 588 665 888 915 979 995 1053 1127 1354 1368 1425 1647 |  |  |
|  | [85676882](http://www.ncbi.nlm.nih.gov/entrez/query.fcgi?cmd=Search&db=Protein&term=85676882&doptcmdl=GenPept) | lysine tRNA synthetase, inducible [Escherichia coli W3110] | 706.90 | 16 | 115 372 381 492 588 665 888 915 979 995 1053 1127 1354 1368 1425 1647 |  |  |
|  | [83587253](http://www.ncbi.nlm.nih.gov/entrez/query.fcgi?cmd=Search&db=Protein&term=83587253&doptcmdl=GenPept) | COG1190: Lysyl-tRNA synthetase (class II) [Escherichia coli 101-1] [MASS=57840] | 706.90 | 16 | 115 372 381 492 588 665 888 915 979 995 1053 1127 1354 1368 1425 1647 |  |  |
|  | [75212057](http://www.ncbi.nlm.nih.gov/entrez/query.fcgi?cmd=Search&db=Protein&term=75212057&doptcmdl=GenPept) | COG1190: Lysyl-tRNA synthetase (class II) [Escherichia coli B171] | 706.90 | 16 | 115 372 381 492 588 665 888 915 979 995 1053 1127 1354 1368 1425 1647 |  |  |
| 19 | [91212145](http://www.ncbi.nlm.nih.gov/entrez/query.fcgi?cmd=Search&db=Protein&term=91212145&doptcmdl=GenPept) | phosphopyruvate hydratase [Escherichia coli UTI89] | 649.83 | 9 | 24 66 495 839 1256 1372 1426 1528 1597 |  |  |
|  | [91073719](http://www.ncbi.nlm.nih.gov/entrez/query.fcgi?cmd=Search&db=Protein&term=91073719&doptcmdl=GenPept) | enolase [Escherichia coli UTI89] | 649.83 | 9 | 24 66 495 839 1256 1372 1426 1528 1597 |  |  |
|  | [90109739](http://www.ncbi.nlm.nih.gov/entrez/query.fcgi?cmd=Search&db=Protein&term=90109739&doptcmdl=GenPept) | Chain F, Crystal Structure Of E. Coli Enolase Complexed With The Minimal Binding Segment Of Rnase E [MASS=45523] | 649.83 | 9 | 24 66 495 839 1256 1372 1426 1528 1597 |  |  |
|  | [90109737](http://www.ncbi.nlm.nih.gov/entrez/query.fcgi?cmd=Search&db=Protein&term=90109737&doptcmdl=GenPept) | Chain D, Crystal Structure Of E. Coli Enolase Complexed With The Minimal Binding Segment Of Rnase E. | 649.83 | 9 | 24 66 495 839 1256 1372 1426 1528 1597 |  |  |
|  | [90109736](http://www.ncbi.nlm.nih.gov/entrez/query.fcgi?cmd=Search&db=Protein&term=90109736&doptcmdl=GenPept) | Chain C, Crystal Structure Of E. Coli Enolase Complexed With The Minimal Binding Segment Of Rnase E. | 649.83 | 9 | 24 66 495 839 1256 1372 1426 1528 1597 |  |  |
| 20 | [91212810](http://www.ncbi.nlm.nih.gov/entrez/query.fcgi?cmd=Search&db=Protein&term=91212810&doptcmdl=GenPept) | 50S ribosomal protein L1 [Escherichia coli UTI89] | 645.39 | 11 | 139 186 376 500 545 873 1303 1416 1494 1576 1648 |  |  |
|  | [91207379](http://www.ncbi.nlm.nih.gov/entrez/query.fcgi?cmd=Search&db=Protein&term=91207379&doptcmdl=GenPept) | 50S ribosomal protein L1 | 645.39 | 11 | 139 186 376 500 545 873 1303 1416 1494 1576 1648 |  |  |
|  | [91207378](http://www.ncbi.nlm.nih.gov/entrez/query.fcgi?cmd=Search&db=Protein&term=91207378&doptcmdl=GenPept) | 50S ribosomal protein L1 | 645.39 | 11 | 139 186 376 500 545 873 1303 1416 1494 1576 1648 |  |  |
|  | [91207377](http://www.ncbi.nlm.nih.gov/entrez/query.fcgi?cmd=Search&db=Protein&term=91207377&doptcmdl=GenPept) | 50S ribosomal protein L1 | 645.39 | 11 | 139 186 376 500 545 873 1303 1416 1494 1576 1648 |  |  |
|  | [91074384](http://www.ncbi.nlm.nih.gov/entrez/query.fcgi?cmd=Search&db=Protein&term=91074384&doptcmdl=GenPept) | 50S ribosomal subunit protein L1 [Escherichia coli UTI89] | 645.39 | 11 | 139 186 376 500 545 873 1303 1416 1494 1576 1648 |  |  |
| 21 | [91211593](http://www.ncbi.nlm.nih.gov/entrez/query.fcgi?cmd=Search&db=Protein&term=91211593&doptcmdl=GenPept) | phosphate acetyltransferase [Escherichia coli UTI89] | 643.68 | 12 | 148 238 323 407 616 645 703 741 1236 1296 1536 1557 |  |  |
|  | [91073167](http://www.ncbi.nlm.nih.gov/entrez/query.fcgi?cmd=Search&db=Protein&term=91073167&doptcmdl=GenPept) | phosphate acetyltransferase [Escherichia coli UTI89] [MASS=77213] | 643.68 | 12 | 148 238 323 407 616 645 703 741 1236 1296 1536 1557 |  |  |
|  | [89109117](http://www.ncbi.nlm.nih.gov/entrez/query.fcgi?cmd=Search&db=Protein&term=89109117&doptcmdl=GenPept) | phosphate acetyltransferase [Escherichia coli W3110] | 643.68 | 12 | 148 238 323 407 616 645 703 741 1236 1296 1536 1557 |  |  |
|  | [85675350](http://www.ncbi.nlm.nih.gov/entrez/query.fcgi?cmd=Search&db=Protein&term=85675350&doptcmdl=GenPept) | phosphate acetyltransferase [Escherichia coli W3110] | 643.68 | 12 | 148 238 323 407 616 645 703 741 1236 1296 1536 1557 |  |  |
|  | [83588280](http://www.ncbi.nlm.nih.gov/entrez/query.fcgi?cmd=Search&db=Protein&term=83588280&doptcmdl=GenPept) | COG0857: BioD-like N-terminal domain of phosphotransacetylase [Escherichia coli 101-1] [MASS=76571] | 643.68 | 12 | 148 238 323 407 616 645 703 741 1236 1296 1536 1557 |  |  |
| 22 | [91213259](http://www.ncbi.nlm.nih.gov/entrez/query.fcgi?cmd=Search&db=Protein&term=91213259&doptcmdl=GenPept) | F0F1 ATP synthase subunit alpha [Escherichia coli UTI89] | 633.89 | 10 | 34 608 727 914 1007 1292 1300 1365 1506 1637 |  |  |
|  | [91074833](http://www.ncbi.nlm.nih.gov/entrez/query.fcgi?cmd=Search&db=Protein&term=91074833&doptcmdl=GenPept) | membrane-bound ATP synthase F1 sector alpha-subunit [Escherichia coli UTI89] | 633.89 | 10 | 34 608 727 914 1007 1292 1300 1365 1506 1637 |  |  |
|  | [89110273](http://www.ncbi.nlm.nih.gov/entrez/query.fcgi?cmd=Search&db=Protein&term=89110273&doptcmdl=GenPept) | F1 sector of membrane-bound ATP synthase, alpha subunit [Escherichia coli W3110] | 633.89 | 10 | 34 608 727 914 1007 1292 1300 1365 1506 1637 |  |  |
|  | [85676304](http://www.ncbi.nlm.nih.gov/entrez/query.fcgi?cmd=Search&db=Protein&term=85676304&doptcmdl=GenPept) | F1 sector of membrane-bound ATP synthase, alpha subunit [Escherichia coli W3110] | 633.89 | 10 | 34 608 727 914 1007 1292 1300 1365 1506 1637 |  |  |
|  | [83588401](http://www.ncbi.nlm.nih.gov/entrez/query.fcgi?cmd=Search&db=Protein&term=83588401&doptcmdl=GenPept) | COG0056: F0F1-type ATP synthase, alpha subunit [Escherichia coli 101-1] | 633.89 | 10 | 34 608 727 914 1007 1292 1300 1365 1506 1637 |  |  |
| 23 | [91211831](http://www.ncbi.nlm.nih.gov/entrez/query.fcgi?cmd=Search&db=Protein&term=91211831&doptcmdl=GenPept) | inositol-5-monophosphate dehydrogenase [Escherichia coli UTI89] | 619.28 | 13 | 77 202 262 275 477 502 773 937 1016 1129 1332 1627 1722 |  |  |
|  | [91073405](http://www.ncbi.nlm.nih.gov/entrez/query.fcgi?cmd=Search&db=Protein&term=91073405&doptcmdl=GenPept) | inosine-5'-monophosphate dehydrogenase [Escherichia coli UTI89] | 619.28 | 13 | 77 202 262 275 477 502 773 937 1016 1129 1332 1627 1722 |  |  |
|  | [89109314](http://www.ncbi.nlm.nih.gov/entrez/query.fcgi?cmd=Search&db=Protein&term=89109314&doptcmdl=GenPept) | IMP dehydrogenase [Escherichia coli W3110] | 619.28 | 13 | 77 202 262 275 477 502 773 937 1016 1129 1332 1627 1722 |  |  |
|  | [85675431](http://www.ncbi.nlm.nih.gov/entrez/query.fcgi?cmd=Search&db=Protein&term=85675431&doptcmdl=GenPept) | IMP dehydrogenase [Escherichia coli W3110] | 619.28 | 13 | 77 202 262 275 477 502 773 937 1016 1129 1332 1627 1722 |  |  |
|  | [83586842](http://www.ncbi.nlm.nih.gov/entrez/query.fcgi?cmd=Search&db=Protein&term=83586842&doptcmdl=GenPept) | COG0516: IMP dehydrogenase/GMP reductase [Escherichia coli 101-1] | 619.28 | 13 | 77 202 262 275 477 502 773 937 1016 1129 1332 1627 1722 |  |  |
| 24 | [91209116](http://www.ncbi.nlm.nih.gov/entrez/query.fcgi?cmd=Search&db=Protein&term=91209116&doptcmdl=GenPept) | organic solvent tolerance protein [Escherichia coli UTI89] | 610.34 | 10 | 21 356 382 405 605 815 969 980 986 1435 |  |  |
|  | [91070690](http://www.ncbi.nlm.nih.gov/entrez/query.fcgi?cmd=Search&db=Protein&term=91070690&doptcmdl=GenPept) | organic solvent tolerance protein precursor [Escherichia coli UTI89] | 610.34 | 10 | 21 356 382 405 605 815 969 980 986 1435 |  |  |
|  | [89106938](http://www.ncbi.nlm.nih.gov/entrez/query.fcgi?cmd=Search&db=Protein&term=89106938&doptcmdl=GenPept) | exported protein required for envelope biosynthesis and integrity [Escherichia coli W3110] | 610.34 | 10 | 21 356 382 405 605 815 969 980 986 1435 |  |  |
|  | [3834368](http://www.ncbi.nlm.nih.gov/entrez/query.fcgi?cmd=Search&db=Protein&term=3834368&doptcmdl=GenPept) | ostA/imp [Escherichia coli] | 610.34 | 10 | 21 356 382 405 605 815 969 980 986 1435 |  |  |
|  | [29336638](http://www.ncbi.nlm.nih.gov/entrez/query.fcgi?cmd=Search&db=Protein&term=29336638&doptcmdl=GenPept) | LPS-assembly protein precursor (Organic solvent tolerance protein) | 610.34 | 10 | 21 356 382 405 605 815 969 980 986 1435 |  |  |

| **Protein IDs*, cont.*** | | | | | | | |
| --- | --- | --- | --- | --- | --- | --- | --- |
| *Grp Nr.* | *Accession Number* | *Protein Name* | *Protein Score* | *Unique PSMs* | *PSM Serial Nrs.* | *Other Grp.* | *Score (other)* |
| 25 | [PTNAB_SHIFL](http://us.expasy.org/uniprot/PTNAB_SHIFL) | PTS system mannose-specific EIIAB component (EIIAB-Man) [Includes: Mannose-specific phosphotransferase enzyme IIA component (EC 2.7.1.-) (PTS system mannose-specific EIIA component) (EIII-Man); Mannose-specific phosphotransferase enz | 604.70 | 9 | 50 145 229 317 953 964 977 1190 1473 |  |  |
|  | [PTNAB_ECOLI](http://us.expasy.org/uniprot/PTNAB_ECOLI) | PTS system mannose-specific EIIAB component (EIIAB-Man) [Includes: Mannose-specific phosphotransferase enzyme IIA component (EC 2.7.1.-) (PTS system mannose-specific EIIA component) (EIII-Man); Mannose-specific phosphotransferase enz | 604.70 | 9 | 50 145 229 317 953 964 977 1190 1473 |  |  |
|  | [PTNAB_ECOL6](http://us.expasy.org/uniprot/PTNAB_ECOL6) | PTS system mannose-specific EIIAB component (EIIAB-Man) [Includes: Mannose-specific phosphotransferase enzyme IIA component (EC 2.7.1.-) (PTS system mannose-specific EIIA component) (EIII-Man); Mannose-specific phosphotransferase enz | 604.70 | 9 | 50 145 229 317 953 964 977 1190 1473 |  |  |
|  | [PTNAB_ECO57](http://us.expasy.org/uniprot/PTNAB_ECO57) | PTS system mannose-specific EIIAB component (EIIAB-Man) [Includes: Mannose-specific phosphotransferase enzyme IIA component (EC 2.7.1.-) (PTS system mannose-specific EIIA component) (EIII-Man); Mannose-specific phosphotransferase enz | 604.70 | 9 | 50 145 229 317 953 964 977 1190 1473 |  |  |
|  | [91211035](http://www.ncbi.nlm.nih.gov/entrez/query.fcgi?cmd=Search&db=Protein&term=91211035&doptcmdl=GenPept) | PTS system, mannose-specific IIAB component [Escherichia coli UTI89] | 533.66 | 8 | 50 145 229 317 953 964 977 1190 |  |  |
| 26 | [91213304](http://www.ncbi.nlm.nih.gov/entrez/query.fcgi?cmd=Search&db=Protein&term=91213304&doptcmdl=GenPept) | transcription termination factor Rho [Escherichia coli UTI89] | 595.40 | 12 | 1 223 396 434 535 570 648 1056 1261 1267 1374 1600 |  |  |
|  | [91074878](http://www.ncbi.nlm.nih.gov/entrez/query.fcgi?cmd=Search&db=Protein&term=91074878&doptcmdl=GenPept) | transcription termination factor rho [Escherichia coli UTI89] | 595.40 | 12 | 1 223 396 434 535 570 648 1056 1261 1267 1374 1600 |  |  |
|  | [89110234](http://www.ncbi.nlm.nih.gov/entrez/query.fcgi?cmd=Search&db=Protein&term=89110234&doptcmdl=GenPept) | transcription termination factor [Escherichia coli W3110] | 595.40 | 12 | 1 223 396 434 535 570 648 1056 1261 1267 1374 1600 |  |  |
|  | [85676265](http://www.ncbi.nlm.nih.gov/entrez/query.fcgi?cmd=Search&db=Protein&term=85676265&doptcmdl=GenPept) | transcription termination factor [Escherichia coli W3110] | 595.40 | 12 | 1 223 396 434 535 570 648 1056 1261 1267 1374 1600 |  |  |
|  | [84027887](http://www.ncbi.nlm.nih.gov/entrez/query.fcgi?cmd=Search&db=Protein&term=84027887&doptcmdl=GenPept) | Transcription termination factor rho (ATP-dependent helicase rho) | 595.40 | 12 | 1 223 396 434 535 570 648 1056 1261 1267 1374 1600 |  |  |
| 27 | [91211755](http://www.ncbi.nlm.nih.gov/entrez/query.fcgi?cmd=Search&db=Protein&term=91211755&doptcmdl=GenPept) | phosphoenolpyruvate-protein phosphotransferase [Escherichia coli UTI89] | 588.25 | 10 | 177 208 214 464 553 610 1058 1281 1344 1664 |  |  |
|  | [91073329](http://www.ncbi.nlm.nih.gov/entrez/query.fcgi?cmd=Search&db=Protein&term=91073329&doptcmdl=GenPept) | phosphoenolpyruvate-protein phosphotransferase system enzyme I [Escherichia coli UTI89] | 588.25 | 10 | 177 208 214 464 553 610 1058 1281 1344 1664 |  |  |
|  | [89109230](http://www.ncbi.nlm.nih.gov/entrez/query.fcgi?cmd=Search&db=Protein&term=89109230&doptcmdl=GenPept) | PEP-protein phosphotransferase of PTS system [Escherichia coli W3110] | 588.25 | 10 | 177 208 214 464 553 610 1058 1281 1344 1664 |  |  |
|  | [83586926](http://www.ncbi.nlm.nih.gov/entrez/query.fcgi?cmd=Search&db=Protein&term=83586926&doptcmdl=GenPept) | COG1080: Phosphoenolpyruvate-protein kinase (PTS system EI component in bacteria) [Escherichia coli 101-1] | 588.25 | 10 | 177 208 214 464 553 610 1058 1281 1344 1664 |  |  |
|  | [83570637](http://www.ncbi.nlm.nih.gov/entrez/query.fcgi?cmd=Search&db=Protein&term=83570637&doptcmdl=GenPept) | COG1080: Phosphoenolpyruvate-protein kinase (PTS system EI component in bacteria) [Shigella dysenteriae 1012] [MASS=63591] | 588.25 | 10 | 177 208 214 464 553 610 1058 1281 1344 1664 |  |  |
| 28 | [91213668](http://www.ncbi.nlm.nih.gov/entrez/query.fcgi?cmd=Search&db=Protein&term=91213668&doptcmdl=GenPept) | fumarate hydratase class I, anaerobic [Escherichia coli UTI89] | 586.24 | 10 | 47 123 426 585 1051 1203 1221 1239 1458 1462 |  |  |
|  | [91075242](http://www.ncbi.nlm.nih.gov/entrez/query.fcgi?cmd=Search&db=Protein&term=91075242&doptcmdl=GenPept) | fumarate hydratase class I, anaerobic [Escherichia coli UTI89] | 586.24 | 10 | 47 123 426 585 1051 1203 1221 1239 1458 1462 |  |  |
|  | [89110843](http://www.ncbi.nlm.nih.gov/entrez/query.fcgi?cmd=Search&db=Protein&term=89110843&doptcmdl=GenPept) | anaerobic class I fumarate hydratase [Escherichia coli W3110] | 586.24 | 10 | 47 123 426 585 1051 1203 1221 1239 1458 1462 |  |  |
|  | [85676874](http://www.ncbi.nlm.nih.gov/entrez/query.fcgi?cmd=Search&db=Protein&term=85676874&doptcmdl=GenPept) | anaerobic class I fumarate hydratase [Escherichia coli W3110] | 586.24 | 10 | 47 123 426 585 1051 1203 1221 1239 1458 1462 |  |  |
|  | [83587247](http://www.ncbi.nlm.nih.gov/entrez/query.fcgi?cmd=Search&db=Protein&term=83587247&doptcmdl=GenPept) | COG1951: Tartrate dehydratase alpha subunit/Fumarate hydratase class I, N-terminal domain [Escherichia coli 101-1] [MASS=60133] | 586.24 | 10 | 47 123 426 585 1051 1203 1221 1239 1458 1462 |  |  |
| 29 | [91213416](http://www.ncbi.nlm.nih.gov/entrez/query.fcgi?cmd=Search&db=Protein&term=91213416&doptcmdl=GenPept) | glutamine synthetase [Escherichia coli UTI89] | 574.18 | 12 | 69 108 120 172 577 750 897 981 1117 1229 1386 1693 |  |  |
|  | [91074990](http://www.ncbi.nlm.nih.gov/entrez/query.fcgi?cmd=Search&db=Protein&term=91074990&doptcmdl=GenPept) | glutamine synthetase [Escherichia coli UTI89] | 574.18 | 12 | 69 108 120 172 577 750 897 981 1117 1229 1386 1693 |  |  |
|  | [89110158](http://www.ncbi.nlm.nih.gov/entrez/query.fcgi?cmd=Search&db=Protein&term=89110158&doptcmdl=GenPept) | glutamine synthetase [Escherichia coli W3110] | 574.18 | 12 | 69 108 120 172 577 750 897 981 1117 1229 1386 1693 |  |  |
|  | [85676189](http://www.ncbi.nlm.nih.gov/entrez/query.fcgi?cmd=Search&db=Protein&term=85676189&doptcmdl=GenPept) | glutamine synthetase [Escherichia coli W3110] | 574.18 | 12 | 69 108 120 172 577 750 897 981 1117 1229 1386 1693 |  |  |
|  | [83586281](http://www.ncbi.nlm.nih.gov/entrez/query.fcgi?cmd=Search&db=Protein&term=83586281&doptcmdl=GenPept) | COG0174: Glutamine synthetase [Escherichia coli 101-1] | 574.18 | 12 | 69 108 120 172 577 750 897 981 1117 1229 1386 1693 |  |  |
| 30 | [91212301](http://www.ncbi.nlm.nih.gov/entrez/query.fcgi?cmd=Search&db=Protein&term=91212301&doptcmdl=GenPept) | fructose-bisphosphate aldolase [Escherichia coli UTI89] | 569.73 | 10 | 80 196 463 635 1288 1350 1454 1671 1703 1726 |  |  |
|  | [91073875](http://www.ncbi.nlm.nih.gov/entrez/query.fcgi?cmd=Search&db=Protein&term=91073875&doptcmdl=GenPept) | fructose-bisphosphate aldolase class II [Escherichia coli UTI89] [MASS=42711] | 569.73 | 10 | 80 196 463 635 1288 1350 1454 1671 1703 1726 |  |  |
|  | [89109703](http://www.ncbi.nlm.nih.gov/entrez/query.fcgi?cmd=Search&db=Protein&term=89109703&doptcmdl=GenPept) | fructose-bisphosphate aldolase, class II [Escherichia coli W3110] | 569.73 | 10 | 80 196 463 635 1288 1350 1454 1671 1703 1726 |  |  |
|  | [882454](http://www.ncbi.nlm.nih.gov/entrez/query.fcgi?cmd=Search&db=Protein&term=882454&doptcmdl=GenPept) | fructose 1,6-bisphosphate aldolase | 569.73 | 10 | 80 196 463 635 1288 1350 1454 1671 1703 1726 |  |  |
|  | [85675736](http://www.ncbi.nlm.nih.gov/entrez/query.fcgi?cmd=Search&db=Protein&term=85675736&doptcmdl=GenPept) | fructose-bisphosphate aldolase, class II [Escherichia coli W3110] | 569.73 | 10 | 80 196 463 635 1288 1350 1454 1671 1703 1726 |  |  |
| 31 | [91211752](http://www.ncbi.nlm.nih.gov/entrez/query.fcgi?cmd=Search&db=Protein&term=91211752&doptcmdl=GenPept) | cysteine synthase A [Escherichia coli UTI89] | 552.81 | 9 | 67 327 377 799 1019 1131 1241 1467 1541 |  |  |
|  | [91073326](http://www.ncbi.nlm.nih.gov/entrez/query.fcgi?cmd=Search&db=Protein&term=91073326&doptcmdl=GenPept) | O-acetylserine sulfhydrolase A [Escherichia coli UTI89] | 552.81 | 9 | 67 327 377 799 1019 1131 1241 1467 1541 |  |  |
|  | [89109228](http://www.ncbi.nlm.nih.gov/entrez/query.fcgi?cmd=Search&db=Protein&term=89109228&doptcmdl=GenPept) | cysteine synthase A, O-acetylserine sulfhydrolase A subunit [Escherichia coli W3110] | 552.81 | 9 | 67 327 377 799 1019 1131 1241 1467 1541 |  |  |
|  | [85675398](http://www.ncbi.nlm.nih.gov/entrez/query.fcgi?cmd=Search&db=Protein&term=85675398&doptcmdl=GenPept) | cysteine synthase A, O-acetylserine sulfhydrolase A subunit [Escherichia coli W3110] | 552.81 | 9 | 67 327 377 799 1019 1131 1241 1467 1541 |  |  |
|  | [83586928](http://www.ncbi.nlm.nih.gov/entrez/query.fcgi?cmd=Search&db=Protein&term=83586928&doptcmdl=GenPept) | COG0031: Cysteine synthase [Escherichia coli 101-1] | 552.81 | 9 | 67 327 377 799 1019 1131 1241 1467 1541 |  |  |
| 32 | [91213473](http://www.ncbi.nlm.nih.gov/entrez/query.fcgi?cmd=Search&db=Protein&term=91213473&doptcmdl=GenPept) | ATP-dependent protease ATP-binding subunit [Escherichia coli UTI89] | 542.89 | 11 | 54 294 370 398 525 602 807 910 946 1170 1214 |  |  |
|  | [91075047](http://www.ncbi.nlm.nih.gov/entrez/query.fcgi?cmd=Search&db=Protein&term=91075047&doptcmdl=GenPept) | ATP-dependent hslVU protease ATP-binding subunit hslU [Escherichia coli UTI89] | 542.89 | 11 | 54 294 370 398 525 602 807 910 946 1170 1214 |  |  |
|  | [89110098](http://www.ncbi.nlm.nih.gov/entrez/query.fcgi?cmd=Search&db=Protein&term=89110098&doptcmdl=GenPept) | molecular chaperone and ATPase component of HslUV protease [Escherichia coli W3110] | 542.89 | 11 | 54 294 370 398 525 602 807 910 946 1170 1214 |  |  |
|  | [85676129](http://www.ncbi.nlm.nih.gov/entrez/query.fcgi?cmd=Search&db=Protein&term=85676129&doptcmdl=GenPept) | molecular chaperone and ATPase component of HslUV protease [Escherichia coli W3110] | 542.89 | 11 | 54 294 370 398 525 602 807 910 946 1170 1214 |  |  |
|  | [83586225](http://www.ncbi.nlm.nih.gov/entrez/query.fcgi?cmd=Search&db=Protein&term=83586225&doptcmdl=GenPept) | COG1220: ATP-dependent protease HslVU (ClpYQ), ATPase subunit [Escherichia coli 101-1] | 542.89 | 11 | 54 294 370 398 525 602 807 910 946 1170 1214 |  |  |
| 33 | [91213489](http://www.ncbi.nlm.nih.gov/entrez/query.fcgi?cmd=Search&db=Protein&term=91213489&doptcmdl=GenPept) | bifunctional catalase HPI/peroxidase HPI [Escherichia coli UTI89] | 536.60 | 10 | 36 122 304 618 1139 1166 1199 1325 1382 1560 |  |  |
|  | [91075063](http://www.ncbi.nlm.nih.gov/entrez/query.fcgi?cmd=Search&db=Protein&term=91075063&doptcmdl=GenPept) | bifunctional catalase HPI/peroxidase HPI [Escherichia coli UTI89] [MASS=81422] | 536.60 | 10 | 36 122 304 618 1139 1166 1199 1325 1382 1560 |  |  |
|  | [89110087](http://www.ncbi.nlm.nih.gov/entrez/query.fcgi?cmd=Search&db=Protein&term=89110087&doptcmdl=GenPept) | catalase/hydroperoxidase HPI(I) [Escherichia coli W3110] | 536.60 | 10 | 36 122 304 618 1139 1166 1199 1325 1382 1560 |  |  |
|  | [85676118](http://www.ncbi.nlm.nih.gov/entrez/query.fcgi?cmd=Search&db=Protein&term=85676118&doptcmdl=GenPept) | catalase/hydroperoxidase HPI(I) [Escherichia coli W3110] | 536.60 | 10 | 36 122 304 618 1139 1166 1199 1325 1382 1560 |  |  |
|  | [83586213](http://www.ncbi.nlm.nih.gov/entrez/query.fcgi?cmd=Search&db=Protein&term=83586213&doptcmdl=GenPept) | COG0376: Catalase (peroxidase I) [Escherichia coli 101-1] | 536.60 | 10 | 36 122 304 618 1139 1166 1199 1325 1382 1560 |  |  |
| 34 | [91213023](http://www.ncbi.nlm.nih.gov/entrez/query.fcgi?cmd=Search&db=Protein&term=91213023&doptcmdl=GenPept) | glutamate decarboxylase alpha [Escherichia coli UTI89] | 532.63 | 8 | 62 248 751 1070 1072 1140 1489 1570 |  |  |
|  | [91074597](http://www.ncbi.nlm.nih.gov/entrez/query.fcgi?cmd=Search&db=Protein&term=91074597&doptcmdl=GenPept) | glutamate decarboxylase alpha [Escherichia coli UTI89] [MASS=55201] | 532.63 | 8 | 62 248 751 1070 1072 1140 1489 1570 |  |  |
|  | [89110496](http://www.ncbi.nlm.nih.gov/entrez/query.fcgi?cmd=Search&db=Protein&term=89110496&doptcmdl=GenPept) | glutamate decarboxylase A, PLP-dependent [Escherichia coli W3110] | 532.63 | 8 | 62 248 751 1070 1072 1140 1489 1570 |  |  |
|  | [85676527](http://www.ncbi.nlm.nih.gov/entrez/query.fcgi?cmd=Search&db=Protein&term=85676527&doptcmdl=GenPept) | glutamate decarboxylase A, PLP-dependent [Escherichia coli W3110] | 532.63 | 8 | 62 248 751 1070 1072 1140 1489 1570 |  |  |
|  | [83588628](http://www.ncbi.nlm.nih.gov/entrez/query.fcgi?cmd=Search&db=Protein&term=83588628&doptcmdl=GenPept) | COG0076: Glutamate decarboxylase and related PLP-dependent proteins [Escherichia coli 101-1] | 532.63 | 8 | 62 248 751 1070 1072 1140 1489 1570 |  |  |
| 35 | [91209235](http://www.ncbi.nlm.nih.gov/entrez/query.fcgi?cmd=Search&db=Protein&term=91209235&doptcmdl=GenPept) | 2,3,4,5-tetrahydropyridine-2-carboxylate N-succinyltransferase [Escherichia coli UTI89] | 521.00 | 8 | 2 349 456 551 654 978 1024 1159 |  |  |
|  | [91070809](http://www.ncbi.nlm.nih.gov/entrez/query.fcgi?cmd=Search&db=Protein&term=91070809&doptcmdl=GenPept) | 2,3,4,5-tetrahydropyridine-2-carboxylate N-succinyltransferase [Escherichia coli UTI89] | 521.00 | 8 | 2 349 456 551 654 978 1024 1159 |  |  |
|  | [89107046](http://www.ncbi.nlm.nih.gov/entrez/query.fcgi?cmd=Search&db=Protein&term=89107046&doptcmdl=GenPept) | 2,3,4,5-tetrahydropyridine-2-carboxylate N-succinyltransferase [Escherichia coli W3110] | 521.00 | 8 | 2 349 456 551 654 978 1024 1159 |  |  |
|  | [83585482](http://www.ncbi.nlm.nih.gov/entrez/query.fcgi?cmd=Search&db=Protein&term=83585482&doptcmdl=GenPept) | COG2171: Tetrahydrodipicolinate N-succinyltransferase [Escherichia coli 101-1] | 521.00 | 8 | 2 349 456 551 654 978 1024 1159 |  |  |
|  | [83569748](http://www.ncbi.nlm.nih.gov/entrez/query.fcgi?cmd=Search&db=Protein&term=83569748&doptcmdl=GenPept) | COG2171: Tetrahydrodipicolinate N-succinyltransferase [Shigella dysenteriae 1012] | 521.00 | 8 | 2 349 456 551 654 978 1024 1159 |  |  |
| 36 | [91211591](http://www.ncbi.nlm.nih.gov/entrez/query.fcgi?cmd=Search&db=Protein&term=91211591&doptcmdl=GenPept) | acetate kinase [Escherichia coli UTI89] | 520.28 | 9 | 91 126 319 366 1119 1201 1279 1289 1403 |  |  |
|  | [91073165](http://www.ncbi.nlm.nih.gov/entrez/query.fcgi?cmd=Search&db=Protein&term=91073165&doptcmdl=GenPept) | acetate kinase [Escherichia coli UTI89] | 520.28 | 9 | 91 126 319 366 1119 1201 1279 1289 1403 |  |  |
|  | [89109116](http://www.ncbi.nlm.nih.gov/entrez/query.fcgi?cmd=Search&db=Protein&term=89109116&doptcmdl=GenPept) | acetate kinase A and propionate kinase 2 [Escherichia coli W3110] | 520.28 | 9 | 91 126 319 366 1119 1201 1279 1289 1403 |  |  |
|  | [83588279](http://www.ncbi.nlm.nih.gov/entrez/query.fcgi?cmd=Search&db=Protein&term=83588279&doptcmdl=GenPept) | COG0282: Acetate kinase [Escherichia coli 101-1] | 520.28 | 9 | 91 126 319 366 1119 1201 1279 1289 1403 |  |  |
|  | [82544775](http://www.ncbi.nlm.nih.gov/entrez/query.fcgi?cmd=Search&db=Protein&term=82544775&doptcmdl=GenPept) | acetate kinase [Shigella boydii Sb227] | 520.28 | 9 | 91 126 319 366 1119 1201 1279 1289 1403 |  |  |
| 37 | [7546190](http://www.ncbi.nlm.nih.gov/entrez/query.fcgi?cmd=Search&db=Protein&term=7546190&doptcmdl=GenPept) | Chain A, Structure Of Adenylosuccinate Synthetase From E. Coli Complexed With Gdp, Imp, Hadacidin, And No3 | 510.39 | 10 | 301 922 951 1106 1147 1192 1249 1408 1495 1555 |  |  |
|  | [6730454](http://www.ncbi.nlm.nih.gov/entrez/query.fcgi?cmd=Search&db=Protein&term=6730454&doptcmdl=GenPept) | Chain A, Design, Synthesis, And X-Ray Crystal Structure Of An Enzyme Bound Bisubstrate Hybrid Inhibitor Of Adenylosuccinate Synthetase | 510.39 | 10 | 301 922 951 1106 1147 1192 1249 1408 1495 1555 |  |  |
|  | [6730453](http://www.ncbi.nlm.nih.gov/entrez/query.fcgi?cmd=Search&db=Protein&term=6730453&doptcmdl=GenPept) | Chain A, Design, Synthesis, And X-Ray Crystal Structure Of An Enzyme Bound Bisubstrate Hybrid Inhibitor Of Adenylosuccinate Synthetase | 510.39 | 10 | 301 922 951 1106 1147 1192 1249 1408 1495 1555 |  |  |
|  | [6730168](http://www.ncbi.nlm.nih.gov/entrez/query.fcgi?cmd=Search&db=Protein&term=6730168&doptcmdl=GenPept) | Chain A, Structure Of Adenylosuccinate Synthetase From E. Coli Complexed With A Stringent Effector, Ppg2':3'p | 510.39 | 10 | 301 922 951 1106 1147 1192 1249 1408 1495 1555 |  |  |
|  | [5542175](http://www.ncbi.nlm.nih.gov/entrez/query.fcgi?cmd=Search&db=Protein&term=5542175&doptcmdl=GenPept) | Chain A, Structure Of The Mutant (R303l) Of Adenylosuccinate Synthetase From E. Coli Complexed With, Gdp, 6-Phosphoryl- Imp, And Mg2+ [MASS=47170] | 510.39 | 10 | 301 922 951 1106 1147 1192 1249 1408 1495 1555 |  |  |
| 38 | [91070758](http://www.ncbi.nlm.nih.gov/entrez/query.fcgi?cmd=Search&db=Protein&term=91070758&doptcmdl=GenPept) | dihydrolipoamide dehydrogenase [Escherichia coli UTI89] | 509.07 | 8 | 303 347 630 724 1128 1253 1377 1667 |  |  |
|  | [89106997](http://www.ncbi.nlm.nih.gov/entrez/query.fcgi?cmd=Search&db=Protein&term=89106997&doptcmdl=GenPept) | lipoamide dehydrogenase, E3 component is part of three enzyme complexes [Escherichia coli W3110] | 509.07 | 8 | 303 347 630 724 1128 1253 1377 1667 |  |  |
|  | [85674337](http://www.ncbi.nlm.nih.gov/entrez/query.fcgi?cmd=Search&db=Protein&term=85674337&doptcmdl=GenPept) | lipoamide dehydrogenase, E3 component is part of three enzyme complexes [Escherichia coli W3110] | 509.07 | 8 | 303 347 630 724 1128 1253 1377 1667 |  |  |
|  | [83584558](http://www.ncbi.nlm.nih.gov/entrez/query.fcgi?cmd=Search&db=Protein&term=83584558&doptcmdl=GenPept) | COG1249: Pyruvate/2-oxoglutarate dehydrogenase complex, dihydrolipoamide dehydrogenase (E3) component, and related enzymes [Escherichia coli 101-1] | 509.07 | 8 | 303 347 630 724 1128 1253 1377 1667 |  |  |
|  | [83569696](http://www.ncbi.nlm.nih.gov/entrez/query.fcgi?cmd=Search&db=Protein&term=83569696&doptcmdl=GenPept) | COG1249: Pyruvate/2-oxoglutarate dehydrogenase complex, dihydrolipoamide dehydrogenase (E3) component, and related enzymes [Shigella dysenteriae 1012] | 509.07 | 8 | 303 347 630 724 1128 1253 1377 1667 |  |  |
| 39 | [89108850](http://www.ncbi.nlm.nih.gov/entrez/query.fcgi?cmd=Search&db=Protein&term=89108850&doptcmdl=GenPept) | gluconate-6-phosphate dehydrogenase, decarboxylating [Escherichia coli W3110] | 504.61 | 10 | 163 624 759 850 992 1034 1270 1335 1497 1697 |  |  |
|  | [56123318](http://www.ncbi.nlm.nih.gov/entrez/query.fcgi?cmd=Search&db=Protein&term=56123318&doptcmdl=GenPept) | Gnd [Escherichia coli] [MASS=51613] | 504.61 | 10 | 163 624 759 850 992 1034 1270 1335 1497 1697 |  |  |
|  | [4867927](http://www.ncbi.nlm.nih.gov/entrez/query.fcgi?cmd=Search&db=Protein&term=4867927&doptcmdl=GenPept) | 6-phosphogluconate dehydrogenase [Escherichia coli] [MASS=50449] | 504.61 | 10 | 163 624 759 850 992 1034 1270 1335 1497 1697 |  |  |
|  | [37788093](http://www.ncbi.nlm.nih.gov/entrez/query.fcgi?cmd=Search&db=Protein&term=37788093&doptcmdl=GenPept) | gluconate-6-phosphate dehydrogenase [Escherichia coli] | 504.61 | 10 | 163 624 759 850 992 1034 1270 1335 1497 1697 |  |  |
|  | [26248404](http://www.ncbi.nlm.nih.gov/entrez/query.fcgi?cmd=Search&db=Protein&term=26248404&doptcmdl=GenPept) | 6-phosphogluconate dehydrogenase [Escherichia coli CFT073] | 504.61 | 10 | 163 624 759 850 992 1034 1270 1335 1497 1697 |  |  |
| 40 | [91212722](http://www.ncbi.nlm.nih.gov/entrez/query.fcgi?cmd=Search&db=Protein&term=91212722&doptcmdl=GenPept) | DNA-directed RNA polymerase subunit alpha [Escherichia coli UTI89] | 491.84 | 10 | 107 195 251 526 539 565 998 999 1347 1617 |  |  |
|  | [91074296](http://www.ncbi.nlm.nih.gov/entrez/query.fcgi?cmd=Search&db=Protein&term=91074296&doptcmdl=GenPept) | RNA polymerase, alpha subunit [Escherichia coli UTI89] | 491.84 | 10 | 107 195 251 526 539 565 998 999 1347 1617 |  |  |
|  | [90110867](http://www.ncbi.nlm.nih.gov/entrez/query.fcgi?cmd=Search&db=Protein&term=90110867&doptcmdl=GenPept) | DNA-directed RNA polymerase subunit alpha (RNAP subunit alpha) (Transcriptase subunit alpha) (RNA polymerase subunit alpha) | 491.84 | 10 | 107 195 251 526 539 565 998 999 1347 1617 |  |  |
|  | [90110865](http://www.ncbi.nlm.nih.gov/entrez/query.fcgi?cmd=Search&db=Protein&term=90110865&doptcmdl=GenPept) | DNA-directed RNA polymerase subunit alpha (RNAP subunit alpha) (Transcriptase subunit alpha) (RNA polymerase subunit alpha) | 491.84 | 10 | 107 195 251 526 539 565 998 999 1347 1617 |  |  |
|  | [89110715](http://www.ncbi.nlm.nih.gov/entrez/query.fcgi?cmd=Search&db=Protein&term=89110715&doptcmdl=GenPept) | RNA polymerase, alpha subunit [Escherichia coli W3110] | 491.84 | 10 | 107 195 251 526 539 565 998 999 1347 1617 |  |  |
| 41 | [91209665](http://www.ncbi.nlm.nih.gov/entrez/query.fcgi?cmd=Search&db=Protein&term=91209665&doptcmdl=GenPept) | citrate lyase beta chain (acyl lyase subunit) [Escherichia coli UTI89] | 486.26 | 6 | 289 905 1086 1609 1673 1700 |  |  |
|  | [91071239](http://www.ncbi.nlm.nih.gov/entrez/query.fcgi?cmd=Search&db=Protein&term=91071239&doptcmdl=GenPept) | citrate lyase beta chain (acyl lyase subunit) [Escherichia coli UTI89] | 486.26 | 6 | 289 905 1086 1609 1673 1700 |  |  |
|  | [90111153](http://www.ncbi.nlm.nih.gov/entrez/query.fcgi?cmd=Search&db=Protein&term=90111153&doptcmdl=GenPept) | citrate lyase, citryl-ACP lyase (beta) subunit [Escherichia coli str. K-12 substr. MG1655] | 486.26 | 6 | 289 905 1086 1609 1673 1700 |  |  |
|  | [89107483](http://www.ncbi.nlm.nih.gov/entrez/query.fcgi?cmd=Search&db=Protein&term=89107483&doptcmdl=GenPept) | citrate lyase, citryl-ACP lyase (beta) subunit [Escherichia coli W3110] | 486.26 | 6 | 289 905 1086 1609 1673 1700 |  |  |
|  | [87081764](http://www.ncbi.nlm.nih.gov/entrez/query.fcgi?cmd=Search&db=Protein&term=87081764&doptcmdl=GenPept) | citrate lyase, citryl-ACP lyase (beta) subunit [Escherichia coli str. K-12 substr. MG1655] | 486.26 | 6 | 289 905 1086 1609 1673 1700 |  |  |
| 42 | [91209761](http://www.ncbi.nlm.nih.gov/entrez/query.fcgi?cmd=Search&db=Protein&term=91209761&doptcmdl=GenPept) | succinyl-CoA synthetase subunit beta [Escherichia coli UTI89] | 483.45 | 7 | 39 833 933 1114 1623 1659 1694 |  |  |
|  | [91071335](http://www.ncbi.nlm.nih.gov/entrez/query.fcgi?cmd=Search&db=Protein&term=91071335&doptcmdl=GenPept) | succinyl-CoA synthetase, beta subunit [Escherichia coli UTI89] | 483.45 | 7 | 39 833 933 1114 1623 1659 1694 |  |  |
|  | [89107585](http://www.ncbi.nlm.nih.gov/entrez/query.fcgi?cmd=Search&db=Protein&term=89107585&doptcmdl=GenPept) | succinyl-CoA synthetase, beta subunit [Escherichia coli W3110] | 483.45 | 7 | 39 833 933 1114 1623 1659 1694 |  |  |
|  | [83585341](http://www.ncbi.nlm.nih.gov/entrez/query.fcgi?cmd=Search&db=Protein&term=83585341&doptcmdl=GenPept) | COG0045: Succinyl-CoA synthetase, beta subunit [Escherichia coli 101-1] | 483.45 | 7 | 39 833 933 1114 1623 1659 1694 |  |  |
|  | [75209704](http://www.ncbi.nlm.nih.gov/entrez/query.fcgi?cmd=Search&db=Protein&term=75209704&doptcmdl=GenPept) | COG0045: Succinyl-CoA synthetase, beta subunit [Escherichia coli B171] | 483.45 | 7 | 39 833 933 1114 1623 1659 1694 |  |  |
| 43 | [91213263](http://www.ncbi.nlm.nih.gov/entrez/query.fcgi?cmd=Search&db=Protein&term=91213263&doptcmdl=GenPept) | F0F1 ATP synthase subunit B [Escherichia coli UTI89] | 482.73 | 8 | 64 265 412 416 559 598 898 1706 |  |  |
|  | [91074837](http://www.ncbi.nlm.nih.gov/entrez/query.fcgi?cmd=Search&db=Protein&term=91074837&doptcmdl=GenPept) | membrane-bound ATP synthase F0 sector subunit b [Escherichia coli UTI89] | 482.73 | 8 | 64 265 412 416 559 598 898 1706 |  |  |
|  | [89110271](http://www.ncbi.nlm.nih.gov/entrez/query.fcgi?cmd=Search&db=Protein&term=89110271&doptcmdl=GenPept) | F0 sector of membrane-bound ATP synthase, subunit b [Escherichia coli W3110] | 482.73 | 8 | 64 265 412 416 559 598 898 1706 |  |  |
|  | [85676302](http://www.ncbi.nlm.nih.gov/entrez/query.fcgi?cmd=Search&db=Protein&term=85676302&doptcmdl=GenPept) | F0 sector of membrane-bound ATP synthase, subunit b [Escherichia coli W3110] | 482.73 | 8 | 64 265 412 416 559 598 898 1706 |  |  |
|  | [83588399](http://www.ncbi.nlm.nih.gov/entrez/query.fcgi?cmd=Search&db=Protein&term=83588399&doptcmdl=GenPept) | COG0711: F0F1-type ATP synthase, subunit b [Escherichia coli 101-1] | 482.73 | 8 | 64 265 412 416 559 598 898 1706 |  |  |
| 44 | [91209183](http://www.ncbi.nlm.nih.gov/entrez/query.fcgi?cmd=Search&db=Protein&term=91209183&doptcmdl=GenPept) | dihydrolipoamide acetyltransferase [Escherichia coli UTI89] | 479.97 | 9 | 9 14 87 180 362 988 1103 1228 1491 |  |  |
|  | [91070757](http://www.ncbi.nlm.nih.gov/entrez/query.fcgi?cmd=Search&db=Protein&term=91070757&doptcmdl=GenPept) | pyruvate dehydrogenase [Escherichia coli UTI89] | 479.97 | 9 | 9 14 87 180 362 988 1103 1228 1491 |  |  |
|  | [89106996](http://www.ncbi.nlm.nih.gov/entrez/query.fcgi?cmd=Search&db=Protein&term=89106996&doptcmdl=GenPept) | pyruvate dehydrogenase, dihydrolipoyltransacetylase component E2 [Escherichia coli W3110] | 479.97 | 9 | 9 14 87 180 362 988 1103 1228 1491 |  |  |
|  | [83584559](http://www.ncbi.nlm.nih.gov/entrez/query.fcgi?cmd=Search&db=Protein&term=83584559&doptcmdl=GenPept) | COG0508: Pyruvate/2-oxoglutarate dehydrogenase complex, dihydrolipoamide acyltransferase (E2) component, and related enzymes [Escherichia coli 101-1] | 479.97 | 9 | 9 14 87 180 362 988 1103 1228 1491 |  |  |
|  | [75210442](http://www.ncbi.nlm.nih.gov/entrez/query.fcgi?cmd=Search&db=Protein&term=75210442&doptcmdl=GenPept) | COG0508: Pyruvate/2-oxoglutarate dehydrogenase complex, dihydrolipoamide acyltransferase (E2) component, and related enzymes [Escherichia coli B171] | 479.97 | 9 | 9 14 87 180 362 988 1103 1228 1491 |  |  |
| 45 | [91211531](http://www.ncbi.nlm.nih.gov/entrez/query.fcgi?cmd=Search&db=Protein&term=91211531&doptcmdl=GenPept) | glycerophosphodiester phosphodiesterase [Escherichia coli UTI89] | 476.28 | 10 | 446 473 548 625 846 892 973 1324 1370 1604 |  |  |
|  | [91073105](http://www.ncbi.nlm.nih.gov/entrez/query.fcgi?cmd=Search&db=Protein&term=91073105&doptcmdl=GenPept) | glycerophosphodiester phosphodiesterase, periplasmic [Escherichia coli UTI89] [MASS=40838] | 476.28 | 10 | 446 473 548 625 846 892 973 1324 1370 1604 |  |  |
|  | [89109056](http://www.ncbi.nlm.nih.gov/entrez/query.fcgi?cmd=Search&db=Protein&term=89109056&doptcmdl=GenPept) | periplasmic glycerophosphodiester phosphodiesterase [Escherichia coli W3110] | 476.28 | 10 | 446 473 548 625 846 892 973 1324 1370 1604 |  |  |
|  | [83588225](http://www.ncbi.nlm.nih.gov/entrez/query.fcgi?cmd=Search&db=Protein&term=83588225&doptcmdl=GenPept) | COG0584: Glycerophosphoryl diester phosphodiesterase [Escherichia coli 101-1] [MASS=42245] | 476.28 | 10 | 446 473 548 625 846 892 973 1324 1370 1604 |  |  |
|  | [75211650](http://www.ncbi.nlm.nih.gov/entrez/query.fcgi?cmd=Search&db=Protein&term=75211650&doptcmdl=GenPept) | COG0584: Glycerophosphoryl diester phosphodiesterase [Escherichia coli B171] [MASS=42295] | 476.28 | 10 | 446 473 548 625 846 892 973 1324 1370 1604 |  |  |
| 46 | [91212808](http://www.ncbi.nlm.nih.gov/entrez/query.fcgi?cmd=Search&db=Protein&term=91212808&doptcmdl=GenPept) | 50S ribosomal protein L7/L12 [Escherichia coli UTI89] | 474.07 | 7 | 185 233 272 299 730 819 1148 |  |  |
|  | [91074382](http://www.ncbi.nlm.nih.gov/entrez/query.fcgi?cmd=Search&db=Protein&term=91074382&doptcmdl=GenPept) | 50S ribosomal subunit protein L7/L12 [Escherichia coli UTI89] | 474.07 | 7 | 185 233 272 299 730 819 1148 |  |  |
|  | [89110053](http://www.ncbi.nlm.nih.gov/entrez/query.fcgi?cmd=Search&db=Protein&term=89110053&doptcmdl=GenPept) | 50S ribosomal subunit protein L7/L12 [Escherichia coli W3110] | 474.07 | 7 | 185 233 272 299 730 819 1148 |  |  |
|  | [85676084](http://www.ncbi.nlm.nih.gov/entrez/query.fcgi?cmd=Search&db=Protein&term=85676084&doptcmdl=GenPept) | 50S ribosomal subunit protein L7/L12 [Escherichia coli W3110] | 474.07 | 7 | 185 233 272 299 730 819 1148 |  |  |
|  | [83584816](http://www.ncbi.nlm.nih.gov/entrez/query.fcgi?cmd=Search&db=Protein&term=83584816&doptcmdl=GenPept) | COG0222: Ribosomal protein L7/L12 [Escherichia coli 101-1] | 474.07 | 7 | 185 233 272 299 730 819 1148 |  |  |
| 47 | [91071493](http://www.ncbi.nlm.nih.gov/entrez/query.fcgi?cmd=Search&db=Protein&term=91071493&doptcmdl=GenPept) | ATP-dependent clp protease ATP-binding subunit clpA [Escherichia coli UTI89] [MASS=84720] | 472.64 | 9 | 15 173 274 896 929 1184 1216 1266 1503 |  |  |
|  | [89107733](http://www.ncbi.nlm.nih.gov/entrez/query.fcgi?cmd=Search&db=Protein&term=89107733&doptcmdl=GenPept) | ATPase and specificity subunit of ClpA-ClpP ATP-dependent serine protease, chaperone activity [Escherichia coli W3110] | 472.64 | 9 | 15 173 274 896 929 1184 1216 1266 1503 |  |  |
|  | [83585826](http://www.ncbi.nlm.nih.gov/entrez/query.fcgi?cmd=Search&db=Protein&term=83585826&doptcmdl=GenPept) | COG0542: ATPases with chaperone activity, ATP-binding subunit [Escherichia coli 101-1] [MASS=84210] | 472.64 | 9 | 15 173 274 896 929 1184 1216 1266 1503 |  |  |
|  | [78099957](http://www.ncbi.nlm.nih.gov/entrez/query.fcgi?cmd=Search&db=Protein&term=78099957&doptcmdl=GenPept) | ATP-dependent Clp protease ATP-binding subunit clpA | 472.64 | 9 | 15 173 274 896 929 1184 1216 1266 1503 |  |  |
|  | [78099956](http://www.ncbi.nlm.nih.gov/entrez/query.fcgi?cmd=Search&db=Protein&term=78099956&doptcmdl=GenPept) | ATP-dependent Clp protease ATP-binding subunit clpA | 472.64 | 9 | 15 173 274 896 929 1184 1216 1266 1503 |  |  |
| 48 | [91070812](http://www.ncbi.nlm.nih.gov/entrez/query.fcgi?cmd=Search&db=Protein&term=91070812&doptcmdl=GenPept) | 30S ribosomal protein S2 [Escherichia coli UTI89] [MASS=34426] | 472.38 | 7 | 476 478 552 776 976 1431 1446 |  |  |
|  | [89107049](http://www.ncbi.nlm.nih.gov/entrez/query.fcgi?cmd=Search&db=Protein&term=89107049&doptcmdl=GenPept) | 30S ribosomal subunit protein S2 [Escherichia coli W3110] | 472.38 | 7 | 476 478 552 776 976 1431 1446 |  |  |
|  | [83754115](http://www.ncbi.nlm.nih.gov/entrez/query.fcgi?cmd=Search&db=Protein&term=83754115&doptcmdl=GenPept) | Chain B, Crystal Structure Of The Bacterial Ribosome From Escherichia Coli At 3.5 A Resolution. This File Contains The 30s Subunit Of The Second 70s Ribosome. The Entire Crystal Structure Contains Two 70s Ribosomes And Is Described In Remark 400. | 472.38 | 7 | 476 478 552 776 976 1431 1446 |  |  |
|  | [83754059](http://www.ncbi.nlm.nih.gov/entrez/query.fcgi?cmd=Search&db=Protein&term=83754059&doptcmdl=GenPept) | Chain B, Crystal Structure Of The Bacterial Ribosome From Escherichia Coli At 3.5 A Resolution. This File Contains The 30s Subunit Of One 70s Ribosome. The Entire Crystal Structure Contains Two 70s Ribosomes And Is Described In Remark 400. | 472.38 | 7 | 476 478 552 776 976 1431 1446 |  |  |
|  | [83585485](http://www.ncbi.nlm.nih.gov/entrez/query.fcgi?cmd=Search&db=Protein&term=83585485&doptcmdl=GenPept) | COG0052: Ribosomal protein S2 [Escherichia coli 101-1] | 472.38 | 7 | 476 478 552 776 976 1431 1446 |  |  |

| **Protein IDs*, cont.*** | | | | | | | |
| --- | --- | --- | --- | --- | --- | --- | --- |
| *Grp Nr.* | *Accession Number* | *Protein Name* | *Protein Score* | *Unique PSMs* | *PSM Serial Nrs.* | *Other Grp.* | *Score (other)* |
| 49 | [91209247](http://www.ncbi.nlm.nih.gov/entrez/query.fcgi?cmd=Search&db=Protein&term=91209247&doptcmdl=GenPept) | outer membrane protein assembly factor YaeT [Escherichia coli UTI89] | 466.46 | 9 | 687 706 716 728 745 1157 1274 1440 1566 |  |  |
|  | [91070821](http://www.ncbi.nlm.nih.gov/entrez/query.fcgi?cmd=Search&db=Protein&term=91070821&doptcmdl=GenPept) | protein with possible extracytoplasmic function [Escherichia coli UTI89] | 466.46 | 9 | 687 706 716 728 745 1157 1274 1440 1566 |  |  |
|  | [89107057](http://www.ncbi.nlm.nih.gov/entrez/query.fcgi?cmd=Search&db=Protein&term=89107057&doptcmdl=GenPept) | hypothetical protein [Escherichia coli W3110] | 466.46 | 9 | 687 706 716 728 745 1157 1274 1440 1566 |  |  |
|  | [85674369](http://www.ncbi.nlm.nih.gov/entrez/query.fcgi?cmd=Search&db=Protein&term=85674369&doptcmdl=GenPept) | conserved hypothetical protein [Escherichia coli W3110] | 466.46 | 9 | 687 706 716 728 745 1157 1274 1440 1566 |  |  |
|  | [84027742](http://www.ncbi.nlm.nih.gov/entrez/query.fcgi?cmd=Search&db=Protein&term=84027742&doptcmdl=GenPept) | Outer membrane protein assembly factor yaeT precursor | 466.46 | 9 | 687 706 716 728 745 1157 1274 1440 1566 |  |  |
| 50 | [SYK1_ECOLI](http://us.expasy.org/uniprot/SYK1_ECOLI) | Lysyl-tRNA synthetase (EC 6.1.1.6) (Lysine--tRNA ligase) (LysRS) | 465.98 | 8 | 130 157 574 831 1009 1010 1050 1686 | *18* | *230.09* |
|  | [SYK1_ECOL6](http://us.expasy.org/uniprot/SYK1_ECOL6) | Lysyl-tRNA synthetase (EC 6.1.1.6) (Lysine--tRNA ligase) (LysRS) | 465.98 | 8 | 130 157 574 831 1009 1010 1050 1686 | *18* | *230.09* |
|  | [SYK1_ECO57](http://us.expasy.org/uniprot/SYK1_ECO57) | Lysyl-tRNA synthetase (EC 6.1.1.6) (Lysine--tRNA ligase) (LysRS) | 465.98 | 8 | 130 157 574 831 1009 1010 1050 1686 | *18* | *230.09* |
|  | [SYK1_SHIFL](http://us.expasy.org/uniprot/SYK1_SHIFL) | Lysyl-tRNA synthetase (EC 6.1.1.6) (Lysine--tRNA ligase) (LysRS) | 465.98 | 8 | 130 157 574 831 1009 1010 1050 1686 | *18* | *171.92* |
|  | [91212268](http://www.ncbi.nlm.nih.gov/entrez/query.fcgi?cmd=Search&db=Protein&term=91212268&doptcmdl=GenPept) | lysyl-tRNA synthetase [Escherichia coli UTI89] | 412.95 | 7 | 130 157 574 1009 1010 1050 1686 | *18* | *230.09* |
| 51 | [91212656](http://www.ncbi.nlm.nih.gov/entrez/query.fcgi?cmd=Search&db=Protein&term=91212656&doptcmdl=GenPept) | malate dehydrogenase [Escherichia coli UTI89] | 459.88 | 8 | 155 307 351 1017 1153 1295 1591 1720 |  |  |
|  | [91074230](http://www.ncbi.nlm.nih.gov/entrez/query.fcgi?cmd=Search&db=Protein&term=91074230&doptcmdl=GenPept) | malate dehydrogenase [Escherichia coli UTI89] [MASS=35057] | 459.88 | 8 | 155 307 351 1017 1153 1295 1591 1720 |  |  |
|  | [89109998](http://www.ncbi.nlm.nih.gov/entrez/query.fcgi?cmd=Search&db=Protein&term=89109998&doptcmdl=GenPept) | malate dehydrogenase, NAD(P)-binding [Escherichia coli W3110] | 459.88 | 8 | 155 307 351 1017 1153 1295 1591 1720 |  |  |
|  | [85676029](http://www.ncbi.nlm.nih.gov/entrez/query.fcgi?cmd=Search&db=Protein&term=85676029&doptcmdl=GenPept) | malate dehydrogenase, NAD(P)-binding [Escherichia coli W3110] | 459.88 | 8 | 155 307 351 1017 1153 1295 1591 1720 |  |  |
|  | [83585393](http://www.ncbi.nlm.nih.gov/entrez/query.fcgi?cmd=Search&db=Protein&term=83585393&doptcmdl=GenPept) | COG0039: Malate/lactate dehydrogenases [Escherichia coli 101-1] | 459.88 | 8 | 155 307 351 1017 1153 1295 1591 1720 |  |  |
| 52 | [91211079](http://www.ncbi.nlm.nih.gov/entrez/query.fcgi?cmd=Search&db=Protein&term=91211079&doptcmdl=GenPept) | pyruvate kinase [Escherichia coli UTI89] | 450.69 | 8 | 393 864 968 1146 1152 1534 1692 1696 |  |  |
|  | [91072653](http://www.ncbi.nlm.nih.gov/entrez/query.fcgi?cmd=Search&db=Protein&term=91072653&doptcmdl=GenPept) | pyruvate kinase II [Escherichia coli UTI89] | 450.69 | 8 | 393 864 968 1146 1152 1534 1692 1696 |  |  |
|  | [89108694](http://www.ncbi.nlm.nih.gov/entrez/query.fcgi?cmd=Search&db=Protein&term=89108694&doptcmdl=GenPept) | pyruvate kinase II [Escherichia coli W3110] | 450.69 | 8 | 393 864 968 1146 1152 1534 1692 1696 |  |  |
|  | [83585545](http://www.ncbi.nlm.nih.gov/entrez/query.fcgi?cmd=Search&db=Protein&term=83585545&doptcmdl=GenPept) | COG0469: Pyruvate kinase [Escherichia coli 101-1] | 450.69 | 8 | 393 864 968 1146 1152 1534 1692 1696 |  |  |
|  | [82543678](http://www.ncbi.nlm.nih.gov/entrez/query.fcgi?cmd=Search&db=Protein&term=82543678&doptcmdl=GenPept) | pyruvate kinase [Shigella boydii Sb227] | 450.69 | 8 | 393 864 968 1146 1152 1534 1692 1696 |  |  |
| 53 | [91070760](http://www.ncbi.nlm.nih.gov/entrez/query.fcgi?cmd=Search&db=Protein&term=91070760&doptcmdl=GenPept) | aconitate hydratase 2 [Escherichia coli UTI89] | 450.37 | 7 | 79 199 352 853 865 919 1441 |  |  |
|  | [89106999](http://www.ncbi.nlm.nih.gov/entrez/query.fcgi?cmd=Search&db=Protein&term=89106999&doptcmdl=GenPept) | bifunctional aconitate hydratase 2 and 2-methylisocitrate dehydratase [Escherichia coli W3110] | 450.37 | 7 | 79 199 352 853 865 919 1441 |  |  |
|  | [85674339](http://www.ncbi.nlm.nih.gov/entrez/query.fcgi?cmd=Search&db=Protein&term=85674339&doptcmdl=GenPept) | bifunctional aconitate hydratase 2 and 2-methylisocitrate dehydratase [Escherichia coli W3110] | 450.37 | 7 | 79 199 352 853 865 919 1441 |  |  |
|  | [83584556](http://www.ncbi.nlm.nih.gov/entrez/query.fcgi?cmd=Search&db=Protein&term=83584556&doptcmdl=GenPept) | COG1049: Aconitase B [Escherichia coli 101-1] | 450.37 | 7 | 79 199 352 853 865 919 1441 |  |  |
|  | [82542721](http://www.ncbi.nlm.nih.gov/entrez/query.fcgi?cmd=Search&db=Protein&term=82542721&doptcmdl=GenPept) | bifunctional aconitate hydratase 2/2-methylisocitrate dehydratase [Shigella boydii Sb227] | 450.37 | 7 | 79 199 352 853 865 919 1441 |  |  |
| 54 | [91209653](http://www.ncbi.nlm.nih.gov/entrez/query.fcgi?cmd=Search&db=Protein&term=91209653&doptcmdl=GenPept) | alkyl hydroperoxide reductase subunit C [Escherichia coli UTI89] | 433.04 | 9 | 224 285 328 642 818 843 889 1231 1550 |  |  |
|  | [91071227](http://www.ncbi.nlm.nih.gov/entrez/query.fcgi?cmd=Search&db=Protein&term=91071227&doptcmdl=GenPept) | alkyl hydroperoxide reductase, C22 subunit; detoxification of hydroperoxides [Escherichia coli UTI89] | 433.04 | 9 | 224 285 328 642 818 843 889 1231 1550 |  |  |
|  | [89107473](http://www.ncbi.nlm.nih.gov/entrez/query.fcgi?cmd=Search&db=Protein&term=89107473&doptcmdl=GenPept) | alkyl hydroperoxide reductase, C22 subunit [Escherichia coli W3110] | 433.04 | 9 | 224 285 328 642 818 843 889 1231 1550 |  |  |
|  | [83586959](http://www.ncbi.nlm.nih.gov/entrez/query.fcgi?cmd=Search&db=Protein&term=83586959&doptcmdl=GenPept) | COG0450: Peroxiredoxin [Escherichia coli 101-1] | 433.04 | 9 | 224 285 328 642 818 843 889 1231 1550 |  |  |
|  | [83570434](http://www.ncbi.nlm.nih.gov/entrez/query.fcgi?cmd=Search&db=Protein&term=83570434&doptcmdl=GenPept) | COG0450: Peroxiredoxin [Shigella dysenteriae 1012] | 433.04 | 9 | 224 285 328 642 818 843 889 1231 1550 |  |  |
| 55 | [2781228](http://www.ncbi.nlm.nih.gov/entrez/query.fcgi?cmd=Search&db=Protein&term=2781228&doptcmdl=GenPept) | Chain B, Structure Of Inorganic Pyrophosphatase | 430.36 | 9 | 146 183 655 662 675 729 890 957 1205 |  |  |
|  | [2781227](http://www.ncbi.nlm.nih.gov/entrez/query.fcgi?cmd=Search&db=Protein&term=2781227&doptcmdl=GenPept) | Chain A, Structure Of Inorganic Pyrophosphatase | 430.36 | 9 | 146 183 655 662 675 729 890 957 1205 |  |  |
|  | [2554848](http://www.ncbi.nlm.nih.gov/entrez/query.fcgi?cmd=Search&db=Protein&term=2554848&doptcmdl=GenPept) | Chain C, Structure Of Inorganic Pyrophosphatase | 430.36 | 9 | 146 183 655 662 675 729 890 957 1205 |  |  |
|  | [2554847](http://www.ncbi.nlm.nih.gov/entrez/query.fcgi?cmd=Search&db=Protein&term=2554847&doptcmdl=GenPept) | Chain B, Structure Of Inorganic Pyrophosphatase | 430.36 | 9 | 146 183 655 662 675 729 890 957 1205 |  |  |
|  | [2554846](http://www.ncbi.nlm.nih.gov/entrez/query.fcgi?cmd=Search&db=Protein&term=2554846&doptcmdl=GenPept) | Chain A, Structure Of Inorganic Pyrophosphatase | 430.36 | 9 | 146 183 655 662 675 729 890 957 1205 |  |  |
| 56 | [91212589](http://www.ncbi.nlm.nih.gov/entrez/query.fcgi?cmd=Search&db=Protein&term=91212589&doptcmdl=GenPept) | translation initiation factor IF-2 [Escherichia coli UTI89] | 429.14 | 10 | 150 204 230 235 445 779 1422 1439 1513 1547 |  |  |
|  | [91074163](http://www.ncbi.nlm.nih.gov/entrez/query.fcgi?cmd=Search&db=Protein&term=91074163&doptcmdl=GenPept) | translation initiation factor IF2-alpha [Escherichia coli UTI89] | 429.14 | 10 | 150 204 230 235 445 779 1422 1439 1513 1547 |  |  |
|  | [89109933](http://www.ncbi.nlm.nih.gov/entrez/query.fcgi?cmd=Search&db=Protein&term=89109933&doptcmdl=GenPept) | fused protein chain initiation factor 2, IF2 [Escherichia coli W3110] | 429.14 | 10 | 150 204 230 235 445 779 1422 1439 1513 1547 |  |  |
|  | [85675964](http://www.ncbi.nlm.nih.gov/entrez/query.fcgi?cmd=Search&db=Protein&term=85675964&doptcmdl=GenPept) | fused protein chain initiation factor 2, IF2 [Escherichia coli W3110] | 429.14 | 10 | 150 204 230 235 445 779 1422 1439 1513 1547 |  |  |
|  | [83585809](http://www.ncbi.nlm.nih.gov/entrez/query.fcgi?cmd=Search&db=Protein&term=83585809&doptcmdl=GenPept) | COG0532: Translation initiation factor 2 (IF-2; GTPase) [Escherichia coli 101-1] | 429.14 | 10 | 150 204 230 235 445 779 1422 1439 1513 1547 |  |  |
| 57 | [91213467](http://www.ncbi.nlm.nih.gov/entrez/query.fcgi?cmd=Search&db=Protein&term=91213467&doptcmdl=GenPept) | glycerol kinase [Escherichia coli UTI89] | 428.42 | 7 | 331 388 443 743 1055 1143 1240 |  |  |
|  | [91075041](http://www.ncbi.nlm.nih.gov/entrez/query.fcgi?cmd=Search&db=Protein&term=91075041&doptcmdl=GenPept) | glycerol kinase [Escherichia coli UTI89] [MASS=60282] | 428.42 | 7 | 331 388 443 743 1055 1143 1240 |  |  |
|  | [89110103](http://www.ncbi.nlm.nih.gov/entrez/query.fcgi?cmd=Search&db=Protein&term=89110103&doptcmdl=GenPept) | glycerol kinase [Escherichia coli W3110] | 428.42 | 7 | 331 388 443 743 1055 1143 1240 |  |  |
|  | [85676134](http://www.ncbi.nlm.nih.gov/entrez/query.fcgi?cmd=Search&db=Protein&term=85676134&doptcmdl=GenPept) | glycerol kinase [Escherichia coli W3110] | 428.42 | 7 | 331 388 443 743 1055 1143 1240 |  |  |
|  | [83586230](http://www.ncbi.nlm.nih.gov/entrez/query.fcgi?cmd=Search&db=Protein&term=83586230&doptcmdl=GenPept) | COG0554: Glycerol kinase [Escherichia coli 101-1] | 428.42 | 7 | 331 388 443 743 1055 1143 1240 |  |  |
| 58 | [91213493](http://www.ncbi.nlm.nih.gov/entrez/query.fcgi?cmd=Search&db=Protein&term=91213493&doptcmdl=GenPept) | glycerol dehydrogenase [Escherichia coli UTI89] | 427.26 | 7 | 147 240 556 767 1096 1169 1202 |  |  |
|  | [91075067](http://www.ncbi.nlm.nih.gov/entrez/query.fcgi?cmd=Search&db=Protein&term=91075067&doptcmdl=GenPept) | glycerol dehydrogenase [Escherichia coli UTI89] | 427.26 | 7 | 147 240 556 767 1096 1169 1202 |  |  |
|  | [90111668](http://www.ncbi.nlm.nih.gov/entrez/query.fcgi?cmd=Search&db=Protein&term=90111668&doptcmdl=GenPept) | glycerol dehydrogenase, NAD [Escherichia coli str. K-12 substr. MG1655] | 427.26 | 7 | 147 240 556 767 1096 1169 1202 |  |  |
|  | [89110084](http://www.ncbi.nlm.nih.gov/entrez/query.fcgi?cmd=Search&db=Protein&term=89110084&doptcmdl=GenPept) | glycerol dehydrogenase, NAD [Escherichia coli W3110] | 427.26 | 7 | 147 240 556 767 1096 1169 1202 |  |  |
|  | [87082352](http://www.ncbi.nlm.nih.gov/entrez/query.fcgi?cmd=Search&db=Protein&term=87082352&doptcmdl=GenPept) | glycerol dehydrogenase, NAD [Escherichia coli str. K-12 substr. MG1655] | 427.26 | 7 | 147 240 556 767 1096 1169 1202 |  |  |
| 59 | [91213752](http://www.ncbi.nlm.nih.gov/entrez/query.fcgi?cmd=Search&db=Protein&term=91213752&doptcmdl=GenPept) | 50S ribosomal protein L9 [Escherichia coli UTI89] | 426.42 | 8 | 266 462 623 663 680 844 936 1262 |  |  |
|  | [91075326](http://www.ncbi.nlm.nih.gov/entrez/query.fcgi?cmd=Search&db=Protein&term=91075326&doptcmdl=GenPept) | 50S ribosomal subunit protein L9 [Escherichia coli UTI89] | 426.42 | 8 | 266 462 623 663 680 844 936 1262 |  |  |
|  | [89110923](http://www.ncbi.nlm.nih.gov/entrez/query.fcgi?cmd=Search&db=Protein&term=89110923&doptcmdl=GenPept) | 50S ribosomal subunit protein L9 [Escherichia coli W3110] | 426.42 | 8 | 266 462 623 663 680 844 936 1262 |  |  |
|  | [85676954](http://www.ncbi.nlm.nih.gov/entrez/query.fcgi?cmd=Search&db=Protein&term=85676954&doptcmdl=GenPept) | 50S ribosomal subunit protein L9 [Escherichia coli W3110] | 426.42 | 8 | 266 462 623 663 680 844 936 1262 |  |  |
|  | [83754125](http://www.ncbi.nlm.nih.gov/entrez/query.fcgi?cmd=Search&db=Protein&term=83754125&doptcmdl=GenPept) | Chain H, Crystal Structure Of The Bacterial Ribosome From Escherichia Coli At 3.5 A Resolution. This File Contains The 50s Subunit Of The Second 70s Ribosome. The Entire Crystal Structure Contains Two 70s Ribosomes And Is Described In Remark 400. | 426.42 | 8 | 266 462 623 663 680 844 936 1262 |  |  |
| 60 | [91209515](http://www.ncbi.nlm.nih.gov/entrez/query.fcgi?cmd=Search&db=Protein&term=91209515&doptcmdl=GenPept) | peptidyl-prolyl cis-trans isomerase (rotamase D) [Escherichia coli UTI89] | 423.01 | 7 | 467 826 835 1136 1273 1329 1563 |  |  |
|  | [91071089](http://www.ncbi.nlm.nih.gov/entrez/query.fcgi?cmd=Search&db=Protein&term=91071089&doptcmdl=GenPept) | peptidyl-prolyl cis-trans isomerase D [Escherichia coli UTI89] | 423.01 | 7 | 467 826 835 1136 1273 1329 1563 |  |  |
|  | [89107311](http://www.ncbi.nlm.nih.gov/entrez/query.fcgi?cmd=Search&db=Protein&term=89107311&doptcmdl=GenPept) | peptidyl-prolyl cis-trans isomerase [Escherichia coli W3110] | 423.01 | 7 | 467 826 835 1136 1273 1329 1563 |  |  |
|  | [85674581](http://www.ncbi.nlm.nih.gov/entrez/query.fcgi?cmd=Search&db=Protein&term=85674581&doptcmdl=GenPept) | peptidyl-prolyl cis-trans isomerase [Escherichia coli W3110] | 423.01 | 7 | 467 826 835 1136 1273 1329 1563 |  |  |
|  | [83587103](http://www.ncbi.nlm.nih.gov/entrez/query.fcgi?cmd=Search&db=Protein&term=83587103&doptcmdl=GenPept) | COG0760: Parvulin-like peptidyl-prolyl isomerase [Escherichia coli 101-1] [MASS=68135] | 423.01 | 7 | 467 826 835 1136 1273 1329 1563 |  |  |
| 61 | [91212755](http://www.ncbi.nlm.nih.gov/entrez/query.fcgi?cmd=Search&db=Protein&term=91212755&doptcmdl=GenPept) | 50S ribosomal protein L4 [Escherichia coli UTI89] | 418.44 | 6 | 295 444 1098 1282 1638 1675 |  |  |
|  | [91074329](http://www.ncbi.nlm.nih.gov/entrez/query.fcgi?cmd=Search&db=Protein&term=91074329&doptcmdl=GenPept) | 50S ribosomal subunit protein L4 [Escherichia coli UTI89] | 418.44 | 6 | 295 444 1098 1282 1638 1675 |  |  |
|  | [89110691](http://www.ncbi.nlm.nih.gov/entrez/query.fcgi?cmd=Search&db=Protein&term=89110691&doptcmdl=GenPept) | 50S ribosomal subunit protein L4 [Escherichia coli W3110] | 418.44 | 6 | 295 444 1098 1282 1638 1675 |  |  |
|  | [85676722](http://www.ncbi.nlm.nih.gov/entrez/query.fcgi?cmd=Search&db=Protein&term=85676722&doptcmdl=GenPept) | 50S ribosomal subunit protein L4 [Escherichia coli W3110] | 418.44 | 6 | 295 444 1098 1282 1638 1675 |  |  |
|  | [83754122](http://www.ncbi.nlm.nih.gov/entrez/query.fcgi?cmd=Search&db=Protein&term=83754122&doptcmdl=GenPept) | Chain E, Crystal Structure Of The Bacterial Ribosome From Escherichia Coli At 3.5 A Resolution. This File Contains The 50s Subunit Of The Second 70s Ribosome. The Entire Crystal Structure Contains Two 70s Ribosomes And Is Described In Remark 400. | 418.44 | 6 | 295 444 1098 1282 1638 1675 |  |  |
| 62 | [91210459](http://www.ncbi.nlm.nih.gov/entrez/query.fcgi?cmd=Search&db=Protein&term=91210459&doptcmdl=GenPept) | global DNA-binding transcriptional dual regulator H-NS [Escherichia coli UTI89] | 410.08 | 7 | 85 324 359 403 489 1323 1340 |  |  |
|  | [91072033](http://www.ncbi.nlm.nih.gov/entrez/query.fcgi?cmd=Search&db=Protein&term=91072033&doptcmdl=GenPept) | Hns transcriptional dual regulator [Escherichia coli UTI89] | 410.08 | 7 | 85 324 359 403 489 1323 1340 |  |  |
|  | [89108083](http://www.ncbi.nlm.nih.gov/entrez/query.fcgi?cmd=Search&db=Protein&term=89108083&doptcmdl=GenPept) | global DNA-binding transcriptional dual regulator H-NS [Escherichia coli W3110] | 410.08 | 7 | 85 324 359 403 489 1323 1340 |  |  |
|  | [83587377](http://www.ncbi.nlm.nih.gov/entrez/query.fcgi?cmd=Search&db=Protein&term=83587377&doptcmdl=GenPept) | COG2916: DNA-binding protein H-NS [Escherichia coli 101-1] | 410.08 | 7 | 85 324 359 403 489 1323 1340 |  |  |
|  | [83569291](http://www.ncbi.nlm.nih.gov/entrez/query.fcgi?cmd=Search&db=Protein&term=83569291&doptcmdl=GenPept) | COG2916: DNA-binding protein H-NS [Shigella dysenteriae 1012] | 410.08 | 7 | 85 324 359 403 489 1323 1340 |  |  |
| 63 | [91213072](http://www.ncbi.nlm.nih.gov/entrez/query.fcgi?cmd=Search&db=Protein&term=91213072&doptcmdl=GenPept) | glycyl-tRNA synthetase subunit beta [Escherichia coli UTI89] | 410.01 | 8 | 71 234 367 442 1348 1519 1608 1724 |  |  |
|  | [91074646](http://www.ncbi.nlm.nih.gov/entrez/query.fcgi?cmd=Search&db=Protein&term=91074646&doptcmdl=GenPept) | glycine tRNA synthetase beta subunit [Escherichia coli UTI89] | 410.01 | 8 | 71 234 367 442 1348 1519 1608 1724 |  |  |
|  | [89110454](http://www.ncbi.nlm.nih.gov/entrez/query.fcgi?cmd=Search&db=Protein&term=89110454&doptcmdl=GenPept) | glycine tRNA synthetase, beta subunit [Escherichia coli W3110] | 410.01 | 8 | 71 234 367 442 1348 1519 1608 1724 |  |  |
|  | [85676485](http://www.ncbi.nlm.nih.gov/entrez/query.fcgi?cmd=Search&db=Protein&term=85676485&doptcmdl=GenPept) | glycine tRNA synthetase, beta subunit [Escherichia coli W3110] | 410.01 | 8 | 71 234 367 442 1348 1519 1608 1724 |  |  |
|  | [83588588](http://www.ncbi.nlm.nih.gov/entrez/query.fcgi?cmd=Search&db=Protein&term=83588588&doptcmdl=GenPept) | COG0751: Glycyl-tRNA synthetase, beta subunit [Escherichia coli 101-1] | 410.01 | 8 | 71 234 367 442 1348 1519 1608 1724 |  |  |
| 64 | [91211457](http://www.ncbi.nlm.nih.gov/entrez/query.fcgi?cmd=Search&db=Protein&term=91211457&doptcmdl=GenPept) | fructose-specific PTS IIA/HPr components [Escherichia coli UTI89] | 408.46 | 7 | 543 790 834 1015 1074 1402 1595 |  |  |
|  | [91073031](http://www.ncbi.nlm.nih.gov/entrez/query.fcgi?cmd=Search&db=Protein&term=91073031&doptcmdl=GenPept) | fructose-specific PTS system IIA component [Escherichia coli UTI89] | 408.46 | 7 | 543 790 834 1015 1074 1402 1595 |  |  |
|  | [89108986](http://www.ncbi.nlm.nih.gov/entrez/query.fcgi?cmd=Search&db=Protein&term=89108986&doptcmdl=GenPept) | fused fructose-specific PTS enzyme IIA component and HPr component [Escherichia coli W3110] | 408.46 | 7 | 543 790 834 1015 1074 1402 1595 |  |  |
|  | [85675282](http://www.ncbi.nlm.nih.gov/entrez/query.fcgi?cmd=Search&db=Protein&term=85675282&doptcmdl=GenPept) | fused fructose-specific PTS enzyme IIA component and HPr component [Escherichia coli W3110] | 408.46 | 7 | 543 790 834 1015 1074 1402 1595 |  |  |
|  | [83588150](http://www.ncbi.nlm.nih.gov/entrez/query.fcgi?cmd=Search&db=Protein&term=83588150&doptcmdl=GenPept) | COG4668: Mannitol/fructose-specific phosphotransferase system, IIA domain [Escherichia coli 101-1] [MASS=39678] | 408.46 | 7 | 543 790 834 1015 1074 1402 1595 |  |  |
| 65 | [91212773](http://www.ncbi.nlm.nih.gov/entrez/query.fcgi?cmd=Search&db=Protein&term=91212773&doptcmdl=GenPept) | bacterioferritin, iron storage and detoxification protein [Escherichia coli UTI89] | 405.22 | 5 | 29 60 796 902 1690 |  |  |
|  | [91074347](http://www.ncbi.nlm.nih.gov/entrez/query.fcgi?cmd=Search&db=Protein&term=91074347&doptcmdl=GenPept) | bacterioferrin [Escherichia coli UTI89] | 405.22 | 5 | 29 60 796 902 1690 |  |  |
|  | [89110674](http://www.ncbi.nlm.nih.gov/entrez/query.fcgi?cmd=Search&db=Protein&term=89110674&doptcmdl=GenPept) | bacterioferritin, iron storage and detoxification protein [Escherichia coli W3110] | 405.22 | 5 | 29 60 796 902 1690 |  |  |
|  | [85676705](http://www.ncbi.nlm.nih.gov/entrez/query.fcgi?cmd=Search&db=Protein&term=85676705&doptcmdl=GenPept) | bacterioferritin, iron storage and detoxification protein [Escherichia coli W3110] | 405.22 | 5 | 29 60 796 902 1690 |  |  |
|  | [809205](http://www.ncbi.nlm.nih.gov/entrez/query.fcgi?cmd=Search&db=Protein&term=809205&doptcmdl=GenPept) | Chain B, The Structure Of A Unique, Two-Fold Symmetric, Haem-Binding Site | 405.22 | 5 | 29 60 796 902 1690 |  |  |
| 66 | [91209512](http://www.ncbi.nlm.nih.gov/entrez/query.fcgi?cmd=Search&db=Protein&term=91209512&doptcmdl=GenPept) | ATP-dependent protease ATP-binding subunit [Escherichia coli UTI89] | 398.22 | 9 | 200 411 423 651 923 1309 1466 1616 1658 |  |  |
|  | [91071086](http://www.ncbi.nlm.nih.gov/entrez/query.fcgi?cmd=Search&db=Protein&term=91071086&doptcmdl=GenPept) | ATP-dependent specificity component of ClpP serine protease, chaperone [Escherichia coli UTI89] | 398.22 | 9 | 200 411 423 651 923 1309 1466 1616 1658 |  |  |
|  | [89107308](http://www.ncbi.nlm.nih.gov/entrez/query.fcgi?cmd=Search&db=Protein&term=89107308&doptcmdl=GenPept) | ATPase and specificity subunit of ClpX-ClpP ATP-dependent serine protease [Escherichia coli W3110] | 398.22 | 9 | 200 411 423 651 923 1309 1466 1616 1658 |  |  |
|  | [85674578](http://www.ncbi.nlm.nih.gov/entrez/query.fcgi?cmd=Search&db=Protein&term=85674578&doptcmdl=GenPept) | ATPase and specificity subunit of ClpX-ClpP ATP-dependent serine protease [Escherichia coli W3110] | 398.22 | 9 | 200 411 423 651 923 1309 1466 1616 1658 |  |  |
|  | [82775655](http://www.ncbi.nlm.nih.gov/entrez/query.fcgi?cmd=Search&db=Protein&term=82775655&doptcmdl=GenPept) | ATP-dependent protease ATP-binding subunit [Shigella dysenteriae Sd197] | 398.22 | 9 | 200 411 423 651 923 1309 1466 1616 1658 |  |  |
| 67 | [91210096](http://www.ncbi.nlm.nih.gov/entrez/query.fcgi?cmd=Search&db=Protein&term=91210096&doptcmdl=GenPept) | TrpR binding protein WrbA [Escherichia coli UTI89] | 395.59 | 6 | 380 503 504 802 1635 1678 |  |  |
|  | [91071670](http://www.ncbi.nlm.nih.gov/entrez/query.fcgi?cmd=Search&db=Protein&term=91071670&doptcmdl=GenPept) | trp repressor binding protein [Escherichia coli UTI89] | 395.59 | 6 | 380 503 504 802 1635 1678 |  |  |
|  | [89107855](http://www.ncbi.nlm.nih.gov/entrez/query.fcgi?cmd=Search&db=Protein&term=89107855&doptcmdl=GenPept) | predicted flavoprotein in Trp regulation [Escherichia coli W3110] | 395.59 | 6 | 380 503 504 802 1635 1678 |  |  |
|  | [83586452](http://www.ncbi.nlm.nih.gov/entrez/query.fcgi?cmd=Search&db=Protein&term=83586452&doptcmdl=GenPept) | COG0655: Multimeric flavodoxin WrbA [Escherichia coli 101-1] | 395.59 | 6 | 380 503 504 802 1635 1678 |  |  |
|  | [75210283](http://www.ncbi.nlm.nih.gov/entrez/query.fcgi?cmd=Search&db=Protein&term=75210283&doptcmdl=GenPept) | COG0655: Multimeric flavodoxin WrbA [Escherichia coli B171] | 395.59 | 6 | 380 503 504 802 1635 1678 |  |  |
| 68 | [91212805](http://www.ncbi.nlm.nih.gov/entrez/query.fcgi?cmd=Search&db=Protein&term=91212805&doptcmdl=GenPept) | DNA-directed RNA polymerase subunit beta' [Escherichia coli UTI89] | 395.26 | 8 | 167 956 962 1036 1271 1488 1612 1622 |  |  |
|  | [91074379](http://www.ncbi.nlm.nih.gov/entrez/query.fcgi?cmd=Search&db=Protein&term=91074379&doptcmdl=GenPept) | RNA polymerase, beta prime subunit [Escherichia coli UTI89] | 395.26 | 8 | 167 956 962 1036 1271 1488 1612 1622 |  |  |
|  | [90111032](http://www.ncbi.nlm.nih.gov/entrez/query.fcgi?cmd=Search&db=Protein&term=90111032&doptcmdl=GenPept) | DNA-directed RNA polymerase subunit beta' (RNAP subunit beta') (Transcriptase subunit beta') (RNA polymerase subunit beta') | 395.26 | 8 | 167 956 962 1036 1271 1488 1612 1622 |  |  |
|  | [89110051](http://www.ncbi.nlm.nih.gov/entrez/query.fcgi?cmd=Search&db=Protein&term=89110051&doptcmdl=GenPept) | RNA polymerase, beta prime subunit [Escherichia coli W3110] | 395.26 | 8 | 167 956 962 1036 1271 1488 1612 1622 |  |  |
|  | [85676082](http://www.ncbi.nlm.nih.gov/entrez/query.fcgi?cmd=Search&db=Protein&term=85676082&doptcmdl=GenPept) | RNA polymerase, beta prime subunit [Escherichia coli W3110] | 395.26 | 8 | 167 956 962 1036 1271 1488 1612 1622 |  |  |
| 69 | [91212739](http://www.ncbi.nlm.nih.gov/entrez/query.fcgi?cmd=Search&db=Protein&term=91212739&doptcmdl=GenPept) | 50S ribosomal protein L5 [Escherichia coli UTI89] | 390.55 | 9 | 13 271 310 384 913 1298 1379 1438 1509 |  |  |
|  | [91074313](http://www.ncbi.nlm.nih.gov/entrez/query.fcgi?cmd=Search&db=Protein&term=91074313&doptcmdl=GenPept) | 50S ribosomal subunit protein L5 [Escherichia coli UTI89] | 390.55 | 9 | 13 271 310 384 913 1298 1379 1438 1509 |  |  |
|  | [89110702](http://www.ncbi.nlm.nih.gov/entrez/query.fcgi?cmd=Search&db=Protein&term=89110702&doptcmdl=GenPept) | 50S ribosomal subunit protein L5 [Escherichia coli W3110] | 390.55 | 9 | 13 271 310 384 913 1298 1379 1438 1509 |  |  |
|  | [85676733](http://www.ncbi.nlm.nih.gov/entrez/query.fcgi?cmd=Search&db=Protein&term=85676733&doptcmdl=GenPept) | 50S ribosomal subunit protein L5 [Escherichia coli W3110] | 390.55 | 9 | 13 271 310 384 913 1298 1379 1438 1509 |  |  |
|  | [83754123](http://www.ncbi.nlm.nih.gov/entrez/query.fcgi?cmd=Search&db=Protein&term=83754123&doptcmdl=GenPept) | Chain F, Crystal Structure Of The Bacterial Ribosome From Escherichia Coli At 3.5 A Resolution. This File Contains The 50s Subunit Of The Second 70s Ribosome. The Entire Crystal Structure Contains Two 70s Ribosomes And Is Described In Remark 400. | 390.55 | 9 | 13 271 310 384 913 1298 1379 1438 1509 |  |  |
| 70 | [91073898](http://www.ncbi.nlm.nih.gov/entrez/query.fcgi?cmd=Search&db=Protein&term=91073898&doptcmdl=GenPept) | S-adenosylmethionine synthetase [Escherichia coli UTI89] [MASS=42423] | 388.16 | 6 | 65 321 361 609 1142 1219 |  |  |
|  | [89109719](http://www.ncbi.nlm.nih.gov/entrez/query.fcgi?cmd=Search&db=Protein&term=89109719&doptcmdl=GenPept) | methionine adenosyltransferase 1 [Escherichia coli W3110] | 388.16 | 6 | 65 321 361 609 1142 1219 |  |  |
|  | [882471](http://www.ncbi.nlm.nih.gov/entrez/query.fcgi?cmd=Search&db=Protein&term=882471&doptcmdl=GenPept) | CG Site No. 507 | 388.16 | 6 | 65 321 361 609 1142 1219 |  |  |
|  | [85675752](http://www.ncbi.nlm.nih.gov/entrez/query.fcgi?cmd=Search&db=Protein&term=85675752&doptcmdl=GenPept) | methionine adenosyltransferase 1 [Escherichia coli W3110] | 388.16 | 6 | 65 321 361 609 1142 1219 |  |  |
|  | [83587660](http://www.ncbi.nlm.nih.gov/entrez/query.fcgi?cmd=Search&db=Protein&term=83587660&doptcmdl=GenPept) | COG0192: S-adenosylmethionine synthetase [Escherichia coli 101-1] | 388.16 | 6 | 65 321 361 609 1142 1219 |  |  |
| 71 | [91212585](http://www.ncbi.nlm.nih.gov/entrez/query.fcgi?cmd=Search&db=Protein&term=91212585&doptcmdl=GenPept) | polynucleotide phosphorylase/polyadenylase [Escherichia coli UTI89] | 383.24 | 8 | 193 320 394 511 692 715 1255 1387 |  |  |
|  | [91074159](http://www.ncbi.nlm.nih.gov/entrez/query.fcgi?cmd=Search&db=Protein&term=91074159&doptcmdl=GenPept) | polyribonucleotide nucleotidyltransferase [Escherichia coli UTI89] [MASS=80548] | 383.24 | 8 | 193 320 394 511 692 715 1255 1387 |  |  |
|  | [89109929](http://www.ncbi.nlm.nih.gov/entrez/query.fcgi?cmd=Search&db=Protein&term=89109929&doptcmdl=GenPept) | polynucleotide phosphorylase/polyadenylase [Escherichia coli W3110] | 383.24 | 8 | 193 320 394 511 692 715 1255 1387 |  |  |
|  | [85675960](http://www.ncbi.nlm.nih.gov/entrez/query.fcgi?cmd=Search&db=Protein&term=85675960&doptcmdl=GenPept) | polynucleotide phosphorylase/polyadenylase [Escherichia coli W3110] | 383.24 | 8 | 193 320 394 511 692 715 1255 1387 |  |  |
|  | [83585805](http://www.ncbi.nlm.nih.gov/entrez/query.fcgi?cmd=Search&db=Protein&term=83585805&doptcmdl=GenPept) | COG1185: Polyribonucleotide nucleotidyltransferase (polynucleotide phosphorylase) [Escherichia coli 101-1] | 383.24 | 8 | 193 320 394 511 692 715 1255 1387 |  |  |
| 72 | [91212167](http://www.ncbi.nlm.nih.gov/entrez/query.fcgi?cmd=Search&db=Protein&term=91212167&doptcmdl=GenPept) | L-1,2-propanediol oxidoreductase [Escherichia coli UTI89] | 378.85 | 7 | 98 105 600 1118 1182 1306 1660 |  |  |
|  | [91073741](http://www.ncbi.nlm.nih.gov/entrez/query.fcgi?cmd=Search&db=Protein&term=91073741&doptcmdl=GenPept) | lactaldehyde reductase [Escherichia coli UTI89] | 378.85 | 7 | 98 105 600 1118 1182 1306 1660 |  |  |
|  | [89109585](http://www.ncbi.nlm.nih.gov/entrez/query.fcgi?cmd=Search&db=Protein&term=89109585&doptcmdl=GenPept) | L-1,2-propanediol oxidoreductase [Escherichia coli W3110] | 378.85 | 7 | 98 105 600 1118 1182 1306 1660 |  |  |
|  | [882694](http://www.ncbi.nlm.nih.gov/entrez/query.fcgi?cmd=Search&db=Protein&term=882694&doptcmdl=GenPept) | 1,2-propanediol oxidoreductase (lactaldehyde reductase | 378.85 | 7 | 98 105 600 1118 1182 1306 1660 |  |  |
|  | [85675618](http://www.ncbi.nlm.nih.gov/entrez/query.fcgi?cmd=Search&db=Protein&term=85675618&doptcmdl=GenPept) | L-1,2-propanediol oxidoreductase [Escherichia coli W3110] | 378.85 | 7 | 98 105 600 1118 1182 1306 1660 |  |  |

| **Protein IDs*, cont.*** | | | | | | | |
| --- | --- | --- | --- | --- | --- | --- | --- |
| *Grp Nr.* | *Accession Number* | *Protein Name* | *Protein Score* | *Unique PSMs* | *PSM Serial Nrs.* | *Other Grp.* | *Score (other)* |
| 73 | [91213137](http://www.ncbi.nlm.nih.gov/entrez/query.fcgi?cmd=Search&db=Protein&term=91213137&doptcmdl=GenPept) | ADP-L-glycero-D-mannoheptose-6-epimerase [Escherichia coli UTI89] | 377.24 | 6 | 643 1121 1173 1185 1414 1421 |  |  |
|  | [91074711](http://www.ncbi.nlm.nih.gov/entrez/query.fcgi?cmd=Search&db=Protein&term=91074711&doptcmdl=GenPept) | ADP-L-glycero-D-mannoheptose-6-epimerase [Escherichia coli UTI89] | 377.24 | 6 | 643 1121 1173 1185 1414 1421 |  |  |
|  | [89110392](http://www.ncbi.nlm.nih.gov/entrez/query.fcgi?cmd=Search&db=Protein&term=89110392&doptcmdl=GenPept) | ADP-L-glycero-D-mannoheptose-6-epimerase, NAD(P)-binding [Escherichia coli W3110] | 377.24 | 6 | 643 1121 1173 1185 1414 1421 |  |  |
|  | [85676423](http://www.ncbi.nlm.nih.gov/entrez/query.fcgi?cmd=Search&db=Protein&term=85676423&doptcmdl=GenPept) | ADP-L-glycero-D-mannoheptose-6-epimerase, NAD(P)-binding [Escherichia coli W3110] | 377.24 | 6 | 643 1121 1173 1185 1414 1421 |  |  |
|  | [83588528](http://www.ncbi.nlm.nih.gov/entrez/query.fcgi?cmd=Search&db=Protein&term=83588528&doptcmdl=GenPept) | COG0451: Nucleoside-diphosphate-sugar epimerases [Escherichia coli 101-1] | 377.24 | 6 | 643 1121 1173 1185 1414 1421 |  |  |
| 74 | [89110189](http://www.ncbi.nlm.nih.gov/entrez/query.fcgi?cmd=Search&db=Protein&term=89110189&doptcmdl=GenPept) | uridine phosphorylase [Escherichia coli W3110] | 374.53 | 6 | 7 268 961 1054 1191 1337 |  |  |
|  | [85676220](http://www.ncbi.nlm.nih.gov/entrez/query.fcgi?cmd=Search&db=Protein&term=85676220&doptcmdl=GenPept) | uridine phosphorylase [Escherichia coli W3110] | 374.53 | 6 | 7 268 961 1054 1191 1337 |  |  |
|  | [83585914](http://www.ncbi.nlm.nih.gov/entrez/query.fcgi?cmd=Search&db=Protein&term=83585914&doptcmdl=GenPept) | COG2820: Uridine phosphorylase [Escherichia coli 101-1] | 374.53 | 6 | 7 268 961 1054 1191 1337 |  |  |
|  | [82778988](http://www.ncbi.nlm.nih.gov/entrez/query.fcgi?cmd=Search&db=Protein&term=82778988&doptcmdl=GenPept) | uridine phosphorylase [Shigella dysenteriae Sd197] | 374.53 | 6 | 7 268 961 1054 1191 1337 |  |  |
|  | [82546184](http://www.ncbi.nlm.nih.gov/entrez/query.fcgi?cmd=Search&db=Protein&term=82546184&doptcmdl=GenPept) | uridine phosphorylase [Shigella boydii Sb227] | 374.53 | 6 | 7 268 961 1054 1191 1337 |  |  |
| 75 | [91209159](http://www.ncbi.nlm.nih.gov/entrez/query.fcgi?cmd=Search&db=Protein&term=91209159&doptcmdl=GenPept) | cell division protein FtsZ [Escherichia coli UTI89] | 373.35 | 7 | 10 41 281 368 451 1002 1698 |  |  |
|  | [91070733](http://www.ncbi.nlm.nih.gov/entrez/query.fcgi?cmd=Search&db=Protein&term=91070733&doptcmdl=GenPept) | cell division; forms circumferential ring; tubulin-like GTP-binding protein and GTPase [Escherichia coli UTI89] | 373.35 | 7 | 10 41 281 368 451 1002 1698 |  |  |
|  | [89106978](http://www.ncbi.nlm.nih.gov/entrez/query.fcgi?cmd=Search&db=Protein&term=89106978&doptcmdl=GenPept) | GTP-binding tubulin-like cell division protein [Escherichia coli W3110] | 373.35 | 7 | 10 41 281 368 451 1002 1698 |  |  |
|  | [85674323](http://www.ncbi.nlm.nih.gov/entrez/query.fcgi?cmd=Search&db=Protein&term=85674323&doptcmdl=GenPept) | GTP-binding tubulin-like cell division protein [Escherichia coli W3110] | 373.35 | 7 | 10 41 281 368 451 1002 1698 |  |  |
|  | [82775502](http://www.ncbi.nlm.nih.gov/entrez/query.fcgi?cmd=Search&db=Protein&term=82775502&doptcmdl=GenPept) | cell division protein FtsZ [Shigella dysenteriae Sd197] | 373.35 | 7 | 10 41 281 368 451 1002 1698 |  |  |
| 76 | [91212757](http://www.ncbi.nlm.nih.gov/entrez/query.fcgi?cmd=Search&db=Protein&term=91212757&doptcmdl=GenPept) | 50S ribosomal protein L3 [Escherichia coli UTI89] | 365.93 | 7 | 225 227 708 726 804 1330 1650 |  |  |
|  | [91074331](http://www.ncbi.nlm.nih.gov/entrez/query.fcgi?cmd=Search&db=Protein&term=91074331&doptcmdl=GenPept) | 50S ribosomal subunit protein L3 [Escherichia coli UTI89] | 365.93 | 7 | 225 227 708 726 804 1330 1650 |  |  |
|  | [89110690](http://www.ncbi.nlm.nih.gov/entrez/query.fcgi?cmd=Search&db=Protein&term=89110690&doptcmdl=GenPept) | 50S ribosomal subunit protein L3 [Escherichia coli W3110] | 365.93 | 7 | 225 227 708 726 804 1330 1650 |  |  |
|  | [85676721](http://www.ncbi.nlm.nih.gov/entrez/query.fcgi?cmd=Search&db=Protein&term=85676721&doptcmdl=GenPept) | 50S ribosomal subunit protein L3 [Escherichia coli W3110] | 365.93 | 7 | 225 227 708 726 804 1330 1650 |  |  |
|  | [83754121](http://www.ncbi.nlm.nih.gov/entrez/query.fcgi?cmd=Search&db=Protein&term=83754121&doptcmdl=GenPept) | Chain D, Crystal Structure Of The Bacterial Ribosome From Escherichia Coli At 3.5 A Resolution. This File Contains The 50s Subunit Of The Second 70s Ribosome. The Entire Crystal Structure Contains Two 70s Ribosomes And Is Described In Remark 400. | 365.93 | 7 | 225 227 708 726 804 1330 1650 |  |  |
| 77 | [ALF1_ECOLI](http://us.expasy.org/uniprot/ALF1_ECOLI) | Fructose-bisphosphate aldolase class I (EC 4.1.2.13) (FBP aldolase) | 362.03 | 6 | 461 965 1005 1060 1105 1165 |  |  |
|  | [ALF1_ECOL6](http://us.expasy.org/uniprot/ALF1_ECOL6) | Fructose-bisphosphate aldolase class I (EC 4.1.2.13) (FBP aldolase) | 362.03 | 6 | 461 965 1005 1060 1105 1165 |  |  |
|  | [91072958](http://www.ncbi.nlm.nih.gov/entrez/query.fcgi?cmd=Search&db=Protein&term=91072958&doptcmdl=GenPept) | hypothetical protein UTI89_C2371 [Escherichia coli UTI89] [MASS=40906] | 302.58 | 5 | 461 965 1005 1060 1105 |  |  |
| 78 | [91212423](http://www.ncbi.nlm.nih.gov/entrez/query.fcgi?cmd=Search&db=Protein&term=91212423&doptcmdl=GenPept) | hypothetical oxidoreductase YqhD [Escherichia coli UTI89] | 357.46 | 6 | 38 100 641 780 1507 1620 |  |  |
|  | [91073997](http://www.ncbi.nlm.nih.gov/entrez/query.fcgi?cmd=Search&db=Protein&term=91073997&doptcmdl=GenPept) | hypothetical oxidoreductase YqhD [Escherichia coli UTI89] | 357.46 | 6 | 38 100 641 780 1507 1620 |  |  |
|  | [89109782](http://www.ncbi.nlm.nih.gov/entrez/query.fcgi?cmd=Search&db=Protein&term=89109782&doptcmdl=GenPept) | alcohol dehydrogenase, NAD(P)-dependent [Escherichia coli W3110] | 357.46 | 6 | 38 100 641 780 1507 1620 |  |  |
|  | [882540](http://www.ncbi.nlm.nih.gov/entrez/query.fcgi?cmd=Search&db=Protein&term=882540&doptcmdl=GenPept) | ORF_o387 | 357.46 | 6 | 38 100 641 780 1507 1620 |  |  |
|  | [85675815](http://www.ncbi.nlm.nih.gov/entrez/query.fcgi?cmd=Search&db=Protein&term=85675815&doptcmdl=GenPept) | alcohol dehydrogenase, NAD(P)-dependent [Escherichia coli W3110] | 357.46 | 6 | 38 100 641 780 1507 1620 |  |  |
| 79 | [99032369](http://www.ncbi.nlm.nih.gov/entrez/query.fcgi?cmd=Search&db=Protein&term=99032369&doptcmdl=GenPept) | Chain B, E. Coli Pyruvate Dehydrogenase H407a Variant Phosphonolactylthiamin Diphosphate Complex | 351.53 | 7 | 89 158 538 626 719 764 1222 |  |  |
|  | [99032368](http://www.ncbi.nlm.nih.gov/entrez/query.fcgi?cmd=Search&db=Protein&term=99032368&doptcmdl=GenPept) | Chain A, E. Coli Pyruvate Dehydrogenase H407a Variant Phosphonolactylthiamin Diphosphate Complex | 351.53 | 7 | 89 158 538 626 719 764 1222 |  |  |
|  | [99032367](http://www.ncbi.nlm.nih.gov/entrez/query.fcgi?cmd=Search&db=Protein&term=99032367&doptcmdl=GenPept) | Chain B, E. Coli Pyruvate Dehydrogenase Phosphonolactylthiamin Diphosphate Complex | 351.53 | 7 | 89 158 538 626 719 764 1222 |  |  |
|  | [99032366](http://www.ncbi.nlm.nih.gov/entrez/query.fcgi?cmd=Search&db=Protein&term=99032366&doptcmdl=GenPept) | Chain A, E. Coli Pyruvate Dehydrogenase Phosphonolactylthiamin Diphosphate Complex | 351.53 | 7 | 89 158 538 626 719 764 1222 |  |  |
|  | [91209182](http://www.ncbi.nlm.nih.gov/entrez/query.fcgi?cmd=Search&db=Protein&term=91209182&doptcmdl=GenPept) | pyruvate dehydrogenase subunit E1 [Escherichia coli UTI89] | 351.53 | 7 | 89 158 538 626 719 764 1222 |  |  |
| 80 | [91211429](http://www.ncbi.nlm.nih.gov/entrez/query.fcgi?cmd=Search&db=Protein&term=91211429&doptcmdl=GenPept) | cytidine deaminase [Escherichia coli UTI89] | 345.68 | 5 | 179 192 1158 1369 1437 |  |  |
|  | [91073003](http://www.ncbi.nlm.nih.gov/entrez/query.fcgi?cmd=Search&db=Protein&term=91073003&doptcmdl=GenPept) | cytidine deaminase [Escherichia coli UTI89] | 345.68 | 5 | 179 192 1158 1369 1437 |  |  |
|  | [89108960](http://www.ncbi.nlm.nih.gov/entrez/query.fcgi?cmd=Search&db=Protein&term=89108960&doptcmdl=GenPept) | cytidine/deoxycytidine deaminase [Escherichia coli W3110] | 345.68 | 5 | 179 192 1158 1369 1437 |  |  |
|  | [85681029](http://www.ncbi.nlm.nih.gov/entrez/query.fcgi?cmd=Search&db=Protein&term=85681029&doptcmdl=GenPept) | Cytidine deaminase (Cytidine aminohydrolase) (CDA) | 345.68 | 5 | 179 192 1158 1369 1437 |  |  |
|  | [85675257](http://www.ncbi.nlm.nih.gov/entrez/query.fcgi?cmd=Search&db=Protein&term=85675257&doptcmdl=GenPept) | cytidine/deoxycytidine deaminase [Escherichia coli W3110] | 345.68 | 5 | 179 192 1158 1369 1437 |  |  |
| 81 | [91212735](http://www.ncbi.nlm.nih.gov/entrez/query.fcgi?cmd=Search&db=Protein&term=91212735&doptcmdl=GenPept) | 50S ribosomal protein L6 [Escherichia coli UTI89] | 338.66 | 7 | 409 569 661 755 803 811 885 |  |  |
|  | [91074309](http://www.ncbi.nlm.nih.gov/entrez/query.fcgi?cmd=Search&db=Protein&term=91074309&doptcmdl=GenPept) | 50S ribosomal subunit protein L6 [Escherichia coli UTI89] | 338.66 | 7 | 409 569 661 755 803 811 885 |  |  |
|  | [89110705](http://www.ncbi.nlm.nih.gov/entrez/query.fcgi?cmd=Search&db=Protein&term=89110705&doptcmdl=GenPept) | 50S ribosomal subunit protein L6 [Escherichia coli W3110] | 338.66 | 7 | 409 569 661 755 803 811 885 |  |  |
|  | [85676736](http://www.ncbi.nlm.nih.gov/entrez/query.fcgi?cmd=Search&db=Protein&term=85676736&doptcmdl=GenPept) | 50S ribosomal subunit protein L6 [Escherichia coli W3110] | 338.66 | 7 | 409 569 661 755 803 811 885 |  |  |
|  | [84028084](http://www.ncbi.nlm.nih.gov/entrez/query.fcgi?cmd=Search&db=Protein&term=84028084&doptcmdl=GenPept) | 50S ribosomal protein L6 | 338.66 | 7 | 409 569 661 755 803 811 885 |  |  |
| 82 | [91213324](http://www.ncbi.nlm.nih.gov/entrez/query.fcgi?cmd=Search&db=Protein&term=91213324&doptcmdl=GenPept) | putative uroporphyrinogen III C-methyltransferase [Escherichia coli UTI89] | 338.02 | 5 | 316 341 672 1504 1545 |  |  |
|  | [91074898](http://www.ncbi.nlm.nih.gov/entrez/query.fcgi?cmd=Search&db=Protein&term=91074898&doptcmdl=GenPept) | putative uroporphyrin-III C-methyltransferase [Escherichia coli UTI89] | 338.02 | 5 | 316 341 672 1504 1545 |  |  |
|  | [89110217](http://www.ncbi.nlm.nih.gov/entrez/query.fcgi?cmd=Search&db=Protein&term=89110217&doptcmdl=GenPept) | uroporphyrinogen III methylase [Escherichia coli W3110] | 338.02 | 5 | 316 341 672 1504 1545 |  |  |
|  | [85676248](http://www.ncbi.nlm.nih.gov/entrez/query.fcgi?cmd=Search&db=Protein&term=85676248&doptcmdl=GenPept) | uroporphyrinogen III methylase [Escherichia coli W3110] [MASS=42963] | 338.02 | 5 | 316 341 672 1504 1545 |  |  |
|  | [83585948](http://www.ncbi.nlm.nih.gov/entrez/query.fcgi?cmd=Search&db=Protein&term=83585948&doptcmdl=GenPept) | COG2959: Uncharacterized enzyme of heme biosynthesis [Escherichia coli 101-1] [MASS=43957] | 338.02 | 5 | 316 341 672 1504 1545 |  |  |
| 83 | [91211850](http://www.ncbi.nlm.nih.gov/entrez/query.fcgi?cmd=Search&db=Protein&term=91211850&doptcmdl=GenPept) | aminopeptidase B [Escherichia coli UTI89] | 336.06 | 6 | 470 646 798 917 1028 1176 |  |  |
|  | [91073424](http://www.ncbi.nlm.nih.gov/entrez/query.fcgi?cmd=Search&db=Protein&term=91073424&doptcmdl=GenPept) | aminopeptidase B [Escherichia coli UTI89] | 336.06 | 6 | 470 646 798 917 1028 1176 |  |  |
|  | [90111453](http://www.ncbi.nlm.nih.gov/entrez/query.fcgi?cmd=Search&db=Protein&term=90111453&doptcmdl=GenPept) | aminopeptidase B [Escherichia coli str. K-12 substr. MG1655] | 336.06 | 6 | 470 646 798 917 1028 1176 |  |  |
|  | [89109329](http://www.ncbi.nlm.nih.gov/entrez/query.fcgi?cmd=Search&db=Protein&term=89109329&doptcmdl=GenPept) | aminopeptidase B [Escherichia coli W3110] | 336.06 | 6 | 470 646 798 917 1028 1176 |  |  |
|  | [87082123](http://www.ncbi.nlm.nih.gov/entrez/query.fcgi?cmd=Search&db=Protein&term=87082123&doptcmdl=GenPept) | aminopeptidase B [Escherichia coli str. K-12 substr. MG1655] | 336.06 | 6 | 470 646 798 917 1028 1176 |  |  |
| 84 | [640119](http://www.ncbi.nlm.nih.gov/entrez/query.fcgi?cmd=Search&db=Protein&term=640119&doptcmdl=GenPept) | Chain B, Structure-Function In E. Coli Iron Superoxide Dismutase: Comparisons With The Manganese Enzyme From T. Thermophilus [MASS=21134] | 334.89 | 6 | 469 714 942 1389 1569 1594 |  |  |
|  | [640118](http://www.ncbi.nlm.nih.gov/entrez/query.fcgi?cmd=Search&db=Protein&term=640118&doptcmdl=GenPept) | Chain A, Structure-Function In E. Coli Iron Superoxide Dismutase: Comparisons With The Manganese Enzyme From T. Thermophilus | 334.89 | 6 | 469 714 942 1389 1569 1594 |  |  |
|  | [640117](http://www.ncbi.nlm.nih.gov/entrez/query.fcgi?cmd=Search&db=Protein&term=640117&doptcmdl=GenPept) | Chain B, Structure-Function In E. Coli Iron Superoxide Dismutase: Comparisons With The Manganese Enzyme From T. Thermophilus | 334.89 | 6 | 469 714 942 1389 1569 1594 |  |  |
|  | [640116](http://www.ncbi.nlm.nih.gov/entrez/query.fcgi?cmd=Search&db=Protein&term=640116&doptcmdl=GenPept) | Chain A, Structure-Function In E. Coli Iron Superoxide Dismutase: Comparisons With The Manganese Enzyme From T. Thermophilus | 334.89 | 6 | 469 714 942 1389 1569 1594 |  |  |
|  | [640115](http://www.ncbi.nlm.nih.gov/entrez/query.fcgi?cmd=Search&db=Protein&term=640115&doptcmdl=GenPept) | Chain B, Structure-Function In E. Coli Iron Superoxide Dismutase: Comparisons With The Manganese Enzyme From T. Thermophilus | 334.89 | 6 | 469 714 942 1389 1569 1594 |  |  |
| 85 | [91210916](http://www.ncbi.nlm.nih.gov/entrez/query.fcgi?cmd=Search&db=Protein&term=91210916&doptcmdl=GenPept) | phosphoenolpyruvate synthase [Escherichia coli UTI89] | 332.44 | 5 | 12 1012 1381 1419 1464 |  |  |
|  | [91072490](http://www.ncbi.nlm.nih.gov/entrez/query.fcgi?cmd=Search&db=Protein&term=91072490&doptcmdl=GenPept) | phosphoenolpyruvate synthase [Escherichia coli UTI89] | 332.44 | 5 | 12 1012 1381 1419 1464 |  |  |
|  | [89108542](http://www.ncbi.nlm.nih.gov/entrez/query.fcgi?cmd=Search&db=Protein&term=89108542&doptcmdl=GenPept) | phosphoenolpyruvate synthase [Escherichia coli W3110] | 332.44 | 5 | 12 1012 1381 1419 1464 |  |  |
|  | [83586006](http://www.ncbi.nlm.nih.gov/entrez/query.fcgi?cmd=Search&db=Protein&term=83586006&doptcmdl=GenPept) | COG0574: Phosphoenolpyruvate synthase/pyruvate phosphate dikinase [Escherichia coli 101-1] [MASS=87379] | 332.44 | 5 | 12 1012 1381 1419 1464 |  |  |
|  | [75209254](http://www.ncbi.nlm.nih.gov/entrez/query.fcgi?cmd=Search&db=Protein&term=75209254&doptcmdl=GenPept) | COG0574: Phosphoenolpyruvate synthase/pyruvate phosphate dikinase [Escherichia coli B171] | 332.44 | 5 | 12 1012 1381 1419 1464 |  |  |
| 86 | [1942724](http://www.ncbi.nlm.nih.gov/entrez/query.fcgi?cmd=Search&db=Protein&term=1942724&doptcmdl=GenPept) | Chain D, Elongation Factor Complex Ef-TuEF-Ts From Escherichia Coli [MASS=30292] | 330.29 | 6 | 537 547 580 636 840 841 |  |  |
|  | [1942722](http://www.ncbi.nlm.nih.gov/entrez/query.fcgi?cmd=Search&db=Protein&term=1942722&doptcmdl=GenPept) | Chain B, Elongation Factor Complex Ef-TuEF-Ts From Escherichia Coli | 330.29 | 6 | 537 547 580 636 840 841 |  |  |
| 87 | [7427908](http://www.ncbi.nlm.nih.gov/entrez/query.fcgi?cmd=Search&db=Protein&term=7427908&doptcmdl=GenPept) | dihydrolipoamide S-succinyltransferase (EC 2.3.1.61) [validated] - Escherichia coli (strain K-12) | 327.01 | 6 | 536 620 754 761 1341 1709 |  |  |
|  | [43022](http://www.ncbi.nlm.nih.gov/entrez/query.fcgi?cmd=Search&db=Protein&term=43022&doptcmdl=GenPept) | E20 component [Escherichia coli] | 327.01 | 6 | 536 620 754 761 1341 1709 |  |  |
|  | [26246694](http://www.ncbi.nlm.nih.gov/entrez/query.fcgi?cmd=Search&db=Protein&term=26246694&doptcmdl=GenPept) | Dihydrolipoamide succinyltransferase component of 2-oxoglutarate dehydrogenase complex [Escherichia coli CFT073] | 327.01 | 6 | 536 620 754 761 1341 1709 |  |  |
|  | [26107093](http://www.ncbi.nlm.nih.gov/entrez/query.fcgi?cmd=Search&db=Protein&term=26107093&doptcmdl=GenPept) | Dihydrolipoamide succinyltransferase component of 2-oxoglutarate dehydrogenase complex [Escherichia coli CFT073] | 327.01 | 6 | 536 620 754 761 1341 1709 |  |  |
|  | [25286453](http://www.ncbi.nlm.nih.gov/entrez/query.fcgi?cmd=Search&db=Protein&term=25286453&doptcmdl=GenPept) | dihydrolipoamide S-succinyltransferase (EC 2.3.1.61) [similarity] - Escherichia coli (strain O157:H7, substrain RIMD 0509952) | 327.01 | 6 | 536 620 754 761 1341 1709 |  |  |
| 88 | [91212339](http://www.ncbi.nlm.nih.gov/entrez/query.fcgi?cmd=Search&db=Protein&term=91212339&doptcmdl=GenPept) | L-asparaginase II [Escherichia coli UTI89] | 317.44 | 4 | 346 417 1004 1233 |  |  |
|  | [91073913](http://www.ncbi.nlm.nih.gov/entrez/query.fcgi?cmd=Search&db=Protein&term=91073913&doptcmdl=GenPept) | L-asparaginase II precursor [Escherichia coli UTI89] | 317.44 | 4 | 346 417 1004 1233 |  |  |
|  | [89109734](http://www.ncbi.nlm.nih.gov/entrez/query.fcgi?cmd=Search&db=Protein&term=89109734&doptcmdl=GenPept) | periplasmic L-asparaginase II [Escherichia coli W3110] | 317.44 | 4 | 346 417 1004 1233 |  |  |
|  | [882486](http://www.ncbi.nlm.nih.gov/entrez/query.fcgi?cmd=Search&db=Protein&term=882486&doptcmdl=GenPept) | L-asparaginase | 317.44 | 4 | 346 417 1004 1233 |  |  |
|  | [85675767](http://www.ncbi.nlm.nih.gov/entrez/query.fcgi?cmd=Search&db=Protein&term=85675767&doptcmdl=GenPept) | periplasmic L-asparaginase II [Escherichia coli W3110] | 317.44 | 4 | 346 417 1004 1233 |  |  |
| 89 | [91209938](http://www.ncbi.nlm.nih.gov/entrez/query.fcgi?cmd=Search&db=Protein&term=91209938&doptcmdl=GenPept) | seryl-tRNA synthetase [Escherichia coli UTI89] | 316.80 | 7 | 212 436 1073 1188 1450 1478 1524 |  |  |
|  | [91071512](http://www.ncbi.nlm.nih.gov/entrez/query.fcgi?cmd=Search&db=Protein&term=91071512&doptcmdl=GenPept) | seryl-tRNA synthetase [Escherichia coli UTI89] | 316.80 | 7 | 212 436 1073 1188 1450 1478 1524 |  |  |
|  | [89107743](http://www.ncbi.nlm.nih.gov/entrez/query.fcgi?cmd=Search&db=Protein&term=89107743&doptcmdl=GenPept) | seryl-tRNA synthetase, also charges selenocysteinyl-tRNA with serine [Escherichia coli W3110] | 316.80 | 7 | 212 436 1073 1188 1450 1478 1524 |  |  |
|  | [83585836](http://www.ncbi.nlm.nih.gov/entrez/query.fcgi?cmd=Search&db=Protein&term=83585836&doptcmdl=GenPept) | COG0172: Seryl-tRNA synthetase [Escherichia coli 101-1] | 316.80 | 7 | 212 436 1073 1188 1450 1478 1524 |  |  |
|  | [82543379](http://www.ncbi.nlm.nih.gov/entrez/query.fcgi?cmd=Search&db=Protein&term=82543379&doptcmdl=GenPept) | seryl-tRNA synthetase [Shigella boydii Sb227] | 316.80 | 7 | 212 436 1073 1188 1450 1478 1524 |  |  |
| 90 | [91214099](http://www.ncbi.nlm.nih.gov/entrez/query.fcgi?cmd=Search&db=Protein&term=91214099&doptcmdl=GenPept) | phosphopentomutase [Escherichia coli UTI89] | 316.45 | 6 | 103 801 820 1163 1606 1625 |  |  |
|  | [91075673](http://www.ncbi.nlm.nih.gov/entrez/query.fcgi?cmd=Search&db=Protein&term=91075673&doptcmdl=GenPept) | phosphopentomutase [Escherichia coli UTI89] | 316.45 | 6 | 103 801 820 1163 1606 1625 |  |  |
|  | [89111091](http://www.ncbi.nlm.nih.gov/entrez/query.fcgi?cmd=Search&db=Protein&term=89111091&doptcmdl=GenPept) | phosphopentomutase [Escherichia coli W3110] | 316.45 | 6 | 103 801 820 1163 1606 1625 |  |  |
|  | [85677122](http://www.ncbi.nlm.nih.gov/entrez/query.fcgi?cmd=Search&db=Protein&term=85677122&doptcmdl=GenPept) | phosphopentomutase [Escherichia coli W3110] | 316.45 | 6 | 103 801 820 1163 1606 1625 |  |  |
|  | [83585291](http://www.ncbi.nlm.nih.gov/entrez/query.fcgi?cmd=Search&db=Protein&term=83585291&doptcmdl=GenPept) | COG1015: Phosphopentomutase [Escherichia coli 101-1] | 316.45 | 6 | 103 801 820 1163 1606 1625 |  |  |
| 91 | [91208150](http://www.ncbi.nlm.nih.gov/entrez/query.fcgi?cmd=Search&db=Protein&term=91208150&doptcmdl=GenPept) | Transaldolase 1 | 302.27 | 6 | 406 414 997 1085 1532 1584 |  |  |
|  | [91208019](http://www.ncbi.nlm.nih.gov/entrez/query.fcgi?cmd=Search&db=Protein&term=91208019&doptcmdl=GenPept) | Transaldolase | 302.27 | 6 | 406 414 997 1085 1532 1584 |  |  |
|  | [91070638](http://www.ncbi.nlm.nih.gov/entrez/query.fcgi?cmd=Search&db=Protein&term=91070638&doptcmdl=GenPept) | transaldolase B [Escherichia coli UTI89] | 302.27 | 6 | 406 414 997 1085 1532 1584 |  |  |
|  | [89106892](http://www.ncbi.nlm.nih.gov/entrez/query.fcgi?cmd=Search&db=Protein&term=89106892&doptcmdl=GenPept) | transaldolase B [Escherichia coli W3110] | 302.27 | 6 | 406 414 997 1085 1532 1584 |  |  |
|  | [82775390](http://www.ncbi.nlm.nih.gov/entrez/query.fcgi?cmd=Search&db=Protein&term=82775390&doptcmdl=GenPept) | transaldolase B [Shigella dysenteriae Sd197] | 302.27 | 6 | 406 414 997 1085 1532 1584 |  |  |
| 92 | [91210428](http://www.ncbi.nlm.nih.gov/entrez/query.fcgi?cmd=Search&db=Protein&term=91210428&doptcmdl=GenPept) | ribose-phosphate pyrophosphokinase [Escherichia coli UTI89] | 300.67 | 5 | 778 783 1011 1375 1429 |  |  |
|  | [91072002](http://www.ncbi.nlm.nih.gov/entrez/query.fcgi?cmd=Search&db=Protein&term=91072002&doptcmdl=GenPept) | ribose-phosphate pyrophosphokinase [Escherichia coli UTI89] | 300.67 | 5 | 778 783 1011 1375 1429 |  |  |
|  | [89108052](http://www.ncbi.nlm.nih.gov/entrez/query.fcgi?cmd=Search&db=Protein&term=89108052&doptcmdl=GenPept) | phosphoribosylpyrophosphate synthase [Escherichia coli W3110] | 300.67 | 5 | 778 783 1011 1375 1429 |  |  |
|  | [86517028](http://www.ncbi.nlm.nih.gov/entrez/query.fcgi?cmd=Search&db=Protein&term=86517028&doptcmdl=GenPept) | PrsA [Escherichia coli] | 300.67 | 5 | 778 783 1011 1375 1429 |  |  |
|  | [86517026](http://www.ncbi.nlm.nih.gov/entrez/query.fcgi?cmd=Search&db=Protein&term=86517026&doptcmdl=GenPept) | PrsA [Escherichia coli] [MASS=34278] | 300.67 | 5 | 778 783 1011 1375 1429 |  |  |
| 93 | [91212021](http://www.ncbi.nlm.nih.gov/entrez/query.fcgi?cmd=Search&db=Protein&term=91212021&doptcmdl=GenPept) | hypothetical protein UTI89_C3020 [Escherichia coli UTI89] | 297.98 | 5 | 337 460 471 725 882 |  |  |
|  | [91073595](http://www.ncbi.nlm.nih.gov/entrez/query.fcgi?cmd=Search&db=Protein&term=91073595&doptcmdl=GenPept) | hypothetical protein UTI89_C3020 [Escherichia coli UTI89] | 297.98 | 5 | 337 460 471 725 882 |  |  |
|  | [89109458](http://www.ncbi.nlm.nih.gov/entrez/query.fcgi?cmd=Search&db=Protein&term=89109458&doptcmdl=GenPept) | hypothetical protein [Escherichia coli W3110] | 297.98 | 5 | 337 460 471 725 882 |  |  |
|  | [85675514](http://www.ncbi.nlm.nih.gov/entrez/query.fcgi?cmd=Search&db=Protein&term=85675514&doptcmdl=GenPept) | hypothetical protein [Escherichia coli W3110] | 297.98 | 5 | 337 460 471 725 882 |  |  |
|  | [83585168](http://www.ncbi.nlm.nih.gov/entrez/query.fcgi?cmd=Search&db=Protein&term=83585168&doptcmdl=GenPept) | COG1652: Uncharacterized protein containing LysM domain [Escherichia coli 101-1] | 297.98 | 5 | 337 460 471 725 882 |  |  |
| 94 | [91209514](http://www.ncbi.nlm.nih.gov/entrez/query.fcgi?cmd=Search&db=Protein&term=91209514&doptcmdl=GenPept) | transcriptional regulator HU subunit beta [Escherichia coli UTI89] | 297.37 | 5 | 44 221 239 287 1589 |  |  |
|  | [91071088](http://www.ncbi.nlm.nih.gov/entrez/query.fcgi?cmd=Search&db=Protein&term=91071088&doptcmdl=GenPept) | DNA-binding protein HU-beta, NS1 (HU-1) [Escherichia coli UTI89] | 297.37 | 5 | 44 221 239 287 1589 |  |  |
|  | [89107310](http://www.ncbi.nlm.nih.gov/entrez/query.fcgi?cmd=Search&db=Protein&term=89107310&doptcmdl=GenPept) | HU, DNA-binding transcriptional regulator, beta subunit [Escherichia coli W3110] | 297.37 | 5 | 44 221 239 287 1589 |  |  |
|  | [85674580](http://www.ncbi.nlm.nih.gov/entrez/query.fcgi?cmd=Search&db=Protein&term=85674580&doptcmdl=GenPept) | HU, DNA-binding transcriptional regulator, beta subunit [Escherichia coli W3110] | 297.37 | 5 | 44 221 239 287 1589 |  |  |
|  | [83587104](http://www.ncbi.nlm.nih.gov/entrez/query.fcgi?cmd=Search&db=Protein&term=83587104&doptcmdl=GenPept) | COG0776: Bacterial nucleoid DNA-binding protein [Escherichia coli 101-1] | 297.37 | 5 | 44 221 239 287 1589 |  |  |
| 95 | [91070839](http://www.ncbi.nlm.nih.gov/entrez/query.fcgi?cmd=Search&db=Protein&term=91070839&doptcmdl=GenPept) | prolyl-tRNA synthetase [Escherichia coli UTI89] [MASS=65710] | 297.14 | 7 | 27 527 633 895 1137 1475 1526 |  |  |
|  | [89107075](http://www.ncbi.nlm.nih.gov/entrez/query.fcgi?cmd=Search&db=Protein&term=89107075&doptcmdl=GenPept) | prolyl-tRNA synthetase [Escherichia coli W3110] | 297.14 | 7 | 27 527 633 895 1137 1475 1526 |  |  |
|  | [85674379](http://www.ncbi.nlm.nih.gov/entrez/query.fcgi?cmd=Search&db=Protein&term=85674379&doptcmdl=GenPept) | prolyl-tRNA synthetase [Escherichia coli W3110] | 297.14 | 7 | 27 527 633 895 1137 1475 1526 |  |  |
|  | [83584732](http://www.ncbi.nlm.nih.gov/entrez/query.fcgi?cmd=Search&db=Protein&term=83584732&doptcmdl=GenPept) | COG0442: Prolyl-tRNA synthetase [Escherichia coli 101-1] | 297.14 | 7 | 27 527 633 895 1137 1475 1526 |  |  |
|  | [82542793](http://www.ncbi.nlm.nih.gov/entrez/query.fcgi?cmd=Search&db=Protein&term=82542793&doptcmdl=GenPept) | prolyl-tRNA synthetase [Shigella boydii Sb227] | 297.14 | 7 | 27 527 633 895 1137 1475 1526 |  |  |
| 96 | [PEPD_ECOLI](http://us.expasy.org/uniprot/PEPD_ECOLI) | Aminoacyl-histidine dipeptidase (EC 3.4.13.3) (Xaa-His dipeptidase) (X-His dipeptidase) (Beta-alanyl-histidine dipeptidase) (Carnosinase) (Peptidase D) | 294.06 | 6 | 59 318 808 918 993 1349 |  |  |
|  | [91209325](http://www.ncbi.nlm.nih.gov/entrez/query.fcgi?cmd=Search&db=Protein&term=91209325&doptcmdl=GenPept) | aminoacyl-histidine dipeptidase (peptidase D) [Escherichia coli UTI89] | 210.12 | 5 | 318 808 918 993 1349 |  |  |
| 97 | [91213253](http://www.ncbi.nlm.nih.gov/entrez/query.fcgi?cmd=Search&db=Protein&term=91213253&doptcmdl=GenPept) | D-fructose-6-phosphate amidotransferase [Escherichia coli UTI89] | 293.25 | 6 | 42 501 540 1189 1284 1642 |  |  |
|  | [91074827](http://www.ncbi.nlm.nih.gov/entrez/query.fcgi?cmd=Search&db=Protein&term=91074827&doptcmdl=GenPept) | L-glutamine:D-fructose-6-phosphate aminotransferase [Escherichia coli UTI89] | 293.25 | 6 | 42 501 540 1189 1284 1642 |  |  |
|  | [89110278](http://www.ncbi.nlm.nih.gov/entrez/query.fcgi?cmd=Search&db=Protein&term=89110278&doptcmdl=GenPept) | L-glutamine:D-fructose-6-phosphate aminotransferase [Escherichia coli W3110] | 293.25 | 6 | 42 501 540 1189 1284 1642 |  |  |
|  | [85676309](http://www.ncbi.nlm.nih.gov/entrez/query.fcgi?cmd=Search&db=Protein&term=85676309&doptcmdl=GenPept) | L-glutamine:D-fructose-6-phosphate aminotransferase [Escherichia coli W3110] | 293.25 | 6 | 42 501 540 1189 1284 1642 |  |  |
|  | [83588406](http://www.ncbi.nlm.nih.gov/entrez/query.fcgi?cmd=Search&db=Protein&term=83588406&doptcmdl=GenPept) | COG0449: Glucosamine 6-phosphate synthetase, contains amidotransferase and phosphosugar isomerase domains [Escherichia coli 101-1] | 293.25 | 6 | 42 501 540 1189 1284 1642 |  |  |

| **Protein IDs*, cont.*** | | | | | | | |
| --- | --- | --- | --- | --- | --- | --- | --- |
| *Grp Nr.* | *Accession Number* | *Protein Name* | *Protein Score* | *Unique PSMs* | *PSM Serial Nrs.* | *Other Grp.* | *Score (other)* |
| 98 | [91074704](http://www.ncbi.nlm.nih.gov/entrez/query.fcgi?cmd=Search&db=Protein&term=91074704&doptcmdl=GenPept) | 2,3-bisphosphoglycerate-independent phosphoglycerate mutase [Escherichia coli UTI89] [MASS=57204] | 292.44 | 6 | 116 419 560 1049 1250 1312 |  |  |
|  | [89110399](http://www.ncbi.nlm.nih.gov/entrez/query.fcgi?cmd=Search&db=Protein&term=89110399&doptcmdl=GenPept) | phosphoglycero mutase III, cofactor-independent [Escherichia coli W3110] | 292.44 | 6 | 116 419 560 1049 1250 1312 |  |  |
|  | [85676430](http://www.ncbi.nlm.nih.gov/entrez/query.fcgi?cmd=Search&db=Protein&term=85676430&doptcmdl=GenPept) | phosphoglycero mutase III, cofactor-independent [Escherichia coli W3110] | 292.44 | 6 | 116 419 560 1049 1250 1312 |  |  |
|  | [85541658](http://www.ncbi.nlm.nih.gov/entrez/query.fcgi?cmd=Search&db=Protein&term=85541658&doptcmdl=GenPept) | 2,3-bisphosphoglycerate-independent phosphoglycerate mutase (Phosphoglyceromutase) (BPG-independent PGAM) (iPGM) | 292.44 | 6 | 116 419 560 1049 1250 1312 |  |  |
|  | [85541656](http://www.ncbi.nlm.nih.gov/entrez/query.fcgi?cmd=Search&db=Protein&term=85541656&doptcmdl=GenPept) | 2,3-bisphosphoglycerate-independent phosphoglycerate mutase (Phosphoglyceromutase) (BPG-independent PGAM) (iPGM) | 292.44 | 6 | 116 419 560 1049 1250 1312 |  |  |
| 99 | [91209731](http://www.ncbi.nlm.nih.gov/entrez/query.fcgi?cmd=Search&db=Protein&term=91209731&doptcmdl=GenPept) | phosphoglucomutase [Escherichia coli UTI89] | 288.42 | 6 | 386 631 1508 1522 1665 1713 |  |  |
|  | [91071305](http://www.ncbi.nlm.nih.gov/entrez/query.fcgi?cmd=Search&db=Protein&term=91071305&doptcmdl=GenPept) | phosphoglucomutase [Escherichia coli UTI89] [MASS=60380] | 288.42 | 6 | 386 631 1508 1522 1665 1713 |  |  |
|  | [89107546](http://www.ncbi.nlm.nih.gov/entrez/query.fcgi?cmd=Search&db=Protein&term=89107546&doptcmdl=GenPept) | phosphoglucomutase [Escherichia coli W3110] | 288.42 | 6 | 386 631 1508 1522 1665 1713 |  |  |
|  | [83585375](http://www.ncbi.nlm.nih.gov/entrez/query.fcgi?cmd=Search&db=Protein&term=83585375&doptcmdl=GenPept) | COG0033: Phosphoglucomutase [Escherichia coli 101-1] | 288.42 | 6 | 386 631 1508 1522 1665 1713 |  |  |
|  | [82543122](http://www.ncbi.nlm.nih.gov/entrez/query.fcgi?cmd=Search&db=Protein&term=82543122&doptcmdl=GenPept) | phosphoglucomutase [Shigella boydii Sb227] | 288.42 | 6 | 386 631 1508 1522 1665 1713 |  |  |
| 100 | [SYN_SHIFL](http://us.expasy.org/uniprot/SYN_SHIFL) | Asparaginyl-tRNA synthetase (EC 6.1.1.22) (Asparagine--tRNA ligase) (AsnRS) | 286.23 | 5 | 688 967 1112 1122 1132 |  |  |
|  | [SYN_ECOLI](http://us.expasy.org/uniprot/SYN_ECOLI) | Asparaginyl-tRNA synthetase (EC 6.1.1.22) (Asparagine--tRNA ligase) (AsnRS) | 286.23 | 5 | 688 967 1112 1122 1132 |  |  |
|  | [SYN_ECOL6](http://us.expasy.org/uniprot/SYN_ECOL6) | Asparaginyl-tRNA synthetase (EC 6.1.1.22) (Asparagine--tRNA ligase) (AsnRS) | 286.23 | 5 | 688 967 1112 1122 1132 |  |  |
|  | [91210032](http://www.ncbi.nlm.nih.gov/entrez/query.fcgi?cmd=Search&db=Protein&term=91210032&doptcmdl=GenPept) | asparaginyl-tRNA synthetase [Escherichia coli UTI89] | 228.66 | 4 | 688 967 1112 1122 |  |  |
| 101 | [91211588](http://www.ncbi.nlm.nih.gov/entrez/query.fcgi?cmd=Search&db=Protein&term=91211588&doptcmdl=GenPept) | hypothetical protein UTI89_C2576 [Escherichia coli UTI89] | 284.40 | 8 | 387 472 480 520 752 1115 1318 1339 |  |  |
|  | [91073162](http://www.ncbi.nlm.nih.gov/entrez/query.fcgi?cmd=Search&db=Protein&term=91073162&doptcmdl=GenPept) | protein YfbU [Escherichia coli UTI89] | 284.40 | 8 | 387 472 480 520 752 1115 1318 1339 |  |  |
|  | [90111415](http://www.ncbi.nlm.nih.gov/entrez/query.fcgi?cmd=Search&db=Protein&term=90111415&doptcmdl=GenPept) | conserved protein [Escherichia coli str. K-12 substr. MG1655] | 284.40 | 8 | 387 472 480 520 752 1115 1318 1339 |  |  |
|  | [89109114](http://www.ncbi.nlm.nih.gov/entrez/query.fcgi?cmd=Search&db=Protein&term=89109114&doptcmdl=GenPept) | hypothetical protein [Escherichia coli W3110] | 284.40 | 8 | 387 472 480 520 752 1115 1318 1339 |  |  |
|  | [87082081](http://www.ncbi.nlm.nih.gov/entrez/query.fcgi?cmd=Search&db=Protein&term=87082081&doptcmdl=GenPept) | conserved protein [Escherichia coli str. K-12 substr. MG1655] | 284.40 | 8 | 387 472 480 520 752 1115 1318 1339 |  |  |
| 102 | [91210093](http://www.ncbi.nlm.nih.gov/entrez/query.fcgi?cmd=Search&db=Protein&term=91210093&doptcmdl=GenPept) | curved DNA-binding protein CbpA [Escherichia coli UTI89] | 282.55 | 6 | 249 290 652 943 1126 1301 |  |  |
|  | [91071667](http://www.ncbi.nlm.nih.gov/entrez/query.fcgi?cmd=Search&db=Protein&term=91071667&doptcmdl=GenPept) | curved DNA-binding protein [Escherichia coli UTI89] | 282.55 | 6 | 249 290 652 943 1126 1301 |  |  |
|  | [89107851](http://www.ncbi.nlm.nih.gov/entrez/query.fcgi?cmd=Search&db=Protein&term=89107851&doptcmdl=GenPept) | curved DNA-binding protein, DnaJ homologue that functions as a co-chaperone of DnaK [Escherichia coli W3110] | 282.55 | 6 | 249 290 652 943 1126 1301 |  |  |
|  | [83586455](http://www.ncbi.nlm.nih.gov/entrez/query.fcgi?cmd=Search&db=Protein&term=83586455&doptcmdl=GenPept) | COG2214: DnaJ-class molecular chaperone [Escherichia coli 101-1] | 282.55 | 6 | 249 290 652 943 1126 1301 |  |  |
|  | [83570599](http://www.ncbi.nlm.nih.gov/entrez/query.fcgi?cmd=Search&db=Protein&term=83570599&doptcmdl=GenPept) | COG2214: DnaJ-class molecular chaperone [Shigella dysenteriae 1012] | 282.55 | 6 | 249 290 652 943 1126 1301 |  |  |
| 103 | [9955374](http://www.ncbi.nlm.nih.gov/entrez/query.fcgi?cmd=Search&db=Protein&term=9955374&doptcmdl=GenPept) | Chain D, Structure R271l Mutant Of E. Coli Pyruvate Kinase [MASS=50689] | 282.20 | 6 | 3 296 350 676 1327 1607 |  |  |
|  | [9955373](http://www.ncbi.nlm.nih.gov/entrez/query.fcgi?cmd=Search&db=Protein&term=9955373&doptcmdl=GenPept) | Chain C, Structure R271l Mutant Of E. Coli Pyruvate Kinase | 282.20 | 6 | 3 296 350 676 1327 1607 |  |  |
|  | [9955372](http://www.ncbi.nlm.nih.gov/entrez/query.fcgi?cmd=Search&db=Protein&term=9955372&doptcmdl=GenPept) | Chain B, Structure R271l Mutant Of E. Coli Pyruvate Kinase | 282.20 | 6 | 3 296 350 676 1327 1607 |  |  |
|  | [9955371](http://www.ncbi.nlm.nih.gov/entrez/query.fcgi?cmd=Search&db=Protein&term=9955371&doptcmdl=GenPept) | Chain A, Structure R271l Mutant Of E. Coli Pyruvate Kinase | 282.20 | 6 | 3 296 350 676 1327 1607 |  |  |
|  | [9955370](http://www.ncbi.nlm.nih.gov/entrez/query.fcgi?cmd=Search&db=Protein&term=9955370&doptcmdl=GenPept) | Chain D, R292d Mutant Of E. Coli Pyruvate Kinase [MASS=50691] | 282.20 | 6 | 3 296 350 676 1327 1607 |  |  |
| 104 | [91210387](http://www.ncbi.nlm.nih.gov/entrez/query.fcgi?cmd=Search&db=Protein&term=91210387&doptcmdl=GenPept) | cell division inhibitor MinD [Escherichia coli UTI89] | 278.56 | 5 | 522 904 994 1087 1120 |  |  |
|  | [91071961](http://www.ncbi.nlm.nih.gov/entrez/query.fcgi?cmd=Search&db=Protein&term=91071961&doptcmdl=GenPept) | cell division inhibitor, membrane ATPase MinD [Escherichia coli UTI89] | 278.56 | 5 | 522 904 994 1087 1120 |  |  |
|  | [89108020](http://www.ncbi.nlm.nih.gov/entrez/query.fcgi?cmd=Search&db=Protein&term=89108020&doptcmdl=GenPept) | membrane ATPase of the MinC-MinD-MinE system [Escherichia coli W3110] | 278.56 | 5 | 522 904 994 1087 1120 |  |  |
|  | [85376575](http://www.ncbi.nlm.nih.gov/entrez/query.fcgi?cmd=Search&db=Protein&term=85376575&doptcmdl=GenPept) | MinD [Escherichia coli] | 278.56 | 5 | 522 904 994 1087 1120 |  |  |
|  | [84028098](http://www.ncbi.nlm.nih.gov/entrez/query.fcgi?cmd=Search&db=Protein&term=84028098&doptcmdl=GenPept) | Septum site-determining protein minD (Cell division inhibitor minD) | 278.56 | 5 | 522 904 994 1087 1120 |  |  |
| 105 | [93115446](http://www.ncbi.nlm.nih.gov/entrez/query.fcgi?cmd=Search&db=Protein&term=93115446&doptcmdl=GenPept) | dTDP-glucose 4,6-dehydratase [Escherichia coli] [MASS=33825] | 274.32 | 5 | 86 197 330 711 1092 |  |  |
|  | [91211325](http://www.ncbi.nlm.nih.gov/entrez/query.fcgi?cmd=Search&db=Protein&term=91211325&doptcmdl=GenPept) | dTDP-glucose 4,6 dehydratase [Escherichia coli UTI89] | 274.32 | 5 | 86 197 330 711 1092 |  |  |
|  | [91072899](http://www.ncbi.nlm.nih.gov/entrez/query.fcgi?cmd=Search&db=Protein&term=91072899&doptcmdl=GenPept) | RfbB, subunit of dTDP-glucose 4,6-dehydratase [Escherichia coli UTI89] [MASS=40516] | 274.32 | 5 | 86 197 330 711 1092 |  |  |
|  | [89108861](http://www.ncbi.nlm.nih.gov/entrez/query.fcgi?cmd=Search&db=Protein&term=89108861&doptcmdl=GenPept) | dTDP-glucose 4,6 dehydratase, NAD(P)-binding [Escherichia coli W3110] | 274.32 | 5 | 86 197 330 711 1092 |  |  |
|  | [799232](http://www.ncbi.nlm.nih.gov/entrez/query.fcgi?cmd=Search&db=Protein&term=799232&doptcmdl=GenPept) | RmlB [Escherichia coli] | 274.32 | 5 | 86 197 330 711 1092 |  |  |
| 106 | [91212723](http://www.ncbi.nlm.nih.gov/entrez/query.fcgi?cmd=Search&db=Protein&term=91212723&doptcmdl=GenPept) | 30S ribosomal protein S4 [Escherichia coli UTI89] | 273.89 | 6 | 668 718 753 1294 1505 1679 |  |  |
|  | [91074297](http://www.ncbi.nlm.nih.gov/entrez/query.fcgi?cmd=Search&db=Protein&term=91074297&doptcmdl=GenPept) | 30S ribosomal subunit protein S4 [Escherichia coli UTI89] | 273.89 | 6 | 668 718 753 1294 1505 1679 |  |  |
|  | [90101726](http://www.ncbi.nlm.nih.gov/entrez/query.fcgi?cmd=Search&db=Protein&term=90101726&doptcmdl=GenPept) | 30S ribosomal protein S4 | 273.89 | 6 | 668 718 753 1294 1505 1679 |  |  |
|  | [90101716](http://www.ncbi.nlm.nih.gov/entrez/query.fcgi?cmd=Search&db=Protein&term=90101716&doptcmdl=GenPept) | 30S ribosomal protein S4 | 273.89 | 6 | 668 718 753 1294 1505 1679 |  |  |
|  | [90101715](http://www.ncbi.nlm.nih.gov/entrez/query.fcgi?cmd=Search&db=Protein&term=90101715&doptcmdl=GenPept) | 30S ribosomal protein S4 | 273.89 | 6 | 668 718 753 1294 1505 1679 |  |  |
| 107 | [91073480](http://www.ncbi.nlm.nih.gov/entrez/query.fcgi?cmd=Search&db=Protein&term=91073480&doptcmdl=GenPept) | protein YfiD [Escherichia coli UTI89] [MASS=14984] | 272.95 | 5 | 63 468 493 601 1669 | *7* | *29.58* |
|  | [89109385](http://www.ncbi.nlm.nih.gov/entrez/query.fcgi?cmd=Search&db=Protein&term=89109385&doptcmdl=GenPept) | pyruvate formate lyase subunit [Escherichia coli W3110] | 272.95 | 5 | 63 468 493 601 1669 | *7* | *29.58* |
|  | [83586769](http://www.ncbi.nlm.nih.gov/entrez/query.fcgi?cmd=Search&db=Protein&term=83586769&doptcmdl=GenPept) | COG3445: Acid-induced glycyl radical enzyme [Escherichia coli 101-1] | 272.95 | 5 | 63 468 493 601 1669 | *7* | *29.58* |
|  | [83570816](http://www.ncbi.nlm.nih.gov/entrez/query.fcgi?cmd=Search&db=Protein&term=83570816&doptcmdl=GenPept) | COG3445: Acid-induced glycyl radical enzyme [Shigella dysenteriae 1012] | 272.95 | 5 | 63 468 493 601 1669 | *7* | *29.58* |
|  | [82545034](http://www.ncbi.nlm.nih.gov/entrez/query.fcgi?cmd=Search&db=Protein&term=82545034&doptcmdl=GenPept) | autonomous glycyl radical cofactor GrcA [Shigella boydii Sb227] | 272.95 | 5 | 63 468 493 601 1669 | *7* | *29.58* |
| 108 | [91212793](http://www.ncbi.nlm.nih.gov/entrez/query.fcgi?cmd=Search&db=Protein&term=91212793&doptcmdl=GenPept) | transcriptional regulator HU subunit alpha [Escherichia coli UTI89] | 272.86 | 5 | 156 871 1084 1407 1716 |  |  |
|  | [91074367](http://www.ncbi.nlm.nih.gov/entrez/query.fcgi?cmd=Search&db=Protein&term=91074367&doptcmdl=GenPept) | DNA-binding protein HU-alpha (HU-2) [Escherichia coli UTI89] | 272.86 | 5 | 156 871 1084 1407 1716 |  |  |
|  | [89110038](http://www.ncbi.nlm.nih.gov/entrez/query.fcgi?cmd=Search&db=Protein&term=89110038&doptcmdl=GenPept) | HU, DNA-binding transcriptional regulator, alpha subunit [Escherichia coli W3110] | 272.86 | 5 | 156 871 1084 1407 1716 |  |  |
|  | [85676069](http://www.ncbi.nlm.nih.gov/entrez/query.fcgi?cmd=Search&db=Protein&term=85676069&doptcmdl=GenPept) | HU, DNA-binding transcriptional regulator, alpha subunit [Escherichia coli W3110] | 272.86 | 5 | 156 871 1084 1407 1716 |  |  |
|  | [83584830](http://www.ncbi.nlm.nih.gov/entrez/query.fcgi?cmd=Search&db=Protein&term=83584830&doptcmdl=GenPept) | COG0776: Bacterial nucleoid DNA-binding protein [Escherichia coli 101-1] | 272.86 | 5 | 156 871 1084 1407 1716 |  |  |
| 109 | [91212823](http://www.ncbi.nlm.nih.gov/entrez/query.fcgi?cmd=Search&db=Protein&term=91212823&doptcmdl=GenPept) | FKBP-type peptidyl-prolyl cis-trans isomerase (rotamase) [Escherichia coli UTI89] | 270.60 | 4 | 734 1101 1395 1681 |  |  |
|  | [91074397](http://www.ncbi.nlm.nih.gov/entrez/query.fcgi?cmd=Search&db=Protein&term=91074397&doptcmdl=GenPept) | FKBP-type peptidyl-prolyl cis-trans isomerase [Escherichia coli UTI89] | 270.60 | 4 | 734 1101 1395 1681 |  |  |
|  | [89110663](http://www.ncbi.nlm.nih.gov/entrez/query.fcgi?cmd=Search&db=Protein&term=89110663&doptcmdl=GenPept) | FKBP-type peptidyl-prolyl cis-trans isomerase [Escherichia coli W3110] | 270.60 | 4 | 734 1101 1395 1681 |  |  |
|  | [862300](http://www.ncbi.nlm.nih.gov/entrez/query.fcgi?cmd=Search&db=Protein&term=862300&doptcmdl=GenPept) | fkpA gene product | 270.60 | 4 | 734 1101 1395 1681 |  |  |
|  | [85676694](http://www.ncbi.nlm.nih.gov/entrez/query.fcgi?cmd=Search&db=Protein&term=85676694&doptcmdl=GenPept) | FKBP-type peptidyl-prolyl cis-trans isomerase [Escherichia coli W3110] | 270.60 | 4 | 734 1101 1395 1681 |  |  |
| 110 | [91075339](http://www.ncbi.nlm.nih.gov/entrez/query.fcgi?cmd=Search&db=Protein&term=91075339&doptcmdl=GenPept) | FKBP-type 22KD peptidyl-prolyl cis-trans isomerase (a rotamase) [Escherichia coli UTI89] | 265.24 | 5 | 389 391 697 1090 1559 |  |  |
|  | [90111705](http://www.ncbi.nlm.nih.gov/entrez/query.fcgi?cmd=Search&db=Protein&term=90111705&doptcmdl=GenPept) | FKBP-type peptidyl-prolyl cis-trans isomerase (rotamase) [Escherichia coli str. K-12 substr. MG1655] | 265.24 | 5 | 389 391 697 1090 1559 |  |  |
|  | [89110927](http://www.ncbi.nlm.nih.gov/entrez/query.fcgi?cmd=Search&db=Protein&term=89110927&doptcmdl=GenPept) | FKBP-type peptidyl-prolyl cis-trans isomerase [Escherichia coli W3110] | 265.24 | 5 | 389 391 697 1090 1559 |  |  |
|  | [87082390](http://www.ncbi.nlm.nih.gov/entrez/query.fcgi?cmd=Search&db=Protein&term=87082390&doptcmdl=GenPept) | FKBP-type peptidyl-prolyl cis-trans isomerase (rotamase) [Escherichia coli str. K-12 substr. MG1655] | 265.24 | 5 | 389 391 697 1090 1559 |  |  |
|  | [85676958](http://www.ncbi.nlm.nih.gov/entrez/query.fcgi?cmd=Search&db=Protein&term=85676958&doptcmdl=GenPept) | FKBP-type peptidyl-prolyl cis-trans isomerase [Escherichia coli W3110] | 265.24 | 5 | 389 391 697 1090 1559 |  |  |
| 111 | [984578](http://www.ncbi.nlm.nih.gov/entrez/query.fcgi?cmd=Search&db=Protein&term=984578&doptcmdl=GenPept) | YafI [Escherichia coli W3110] | 263.37 | 4 | 78 457 1000 1237 |  |  |
|  | [91070884](http://www.ncbi.nlm.nih.gov/entrez/query.fcgi?cmd=Search&db=Protein&term=91070884&doptcmdl=GenPept) | phosphoheptose isomerase [Escherichia coli UTI89] | 263.37 | 4 | 78 457 1000 1237 |  |  |
|  | [89107097](http://www.ncbi.nlm.nih.gov/entrez/query.fcgi?cmd=Search&db=Protein&term=89107097&doptcmdl=GenPept) | D-sedoheptulose 7-phosphate isomerase [Escherichia coli W3110] | 263.37 | 4 | 78 457 1000 1237 |  |  |
|  | [83586303](http://www.ncbi.nlm.nih.gov/entrez/query.fcgi?cmd=Search&db=Protein&term=83586303&doptcmdl=GenPept) | COG0279: Phosphoheptose isomerase [Escherichia coli 101-1] | 263.37 | 4 | 78 457 1000 1237 |  |  |
|  | [83570263](http://www.ncbi.nlm.nih.gov/entrez/query.fcgi?cmd=Search&db=Protein&term=83570263&doptcmdl=GenPept) | COG0279: Phosphoheptose isomerase [Shigella dysenteriae 1012] | 263.37 | 4 | 78 457 1000 1237 |  |  |
| 112 | [9257158](http://www.ncbi.nlm.nih.gov/entrez/query.fcgi?cmd=Search&db=Protein&term=9257158&doptcmdl=GenPept) | Chain A, Aspartate Aminotransferase From Escherichia Coli, C191w Mutation, With Bound Maleate [MASS=43656] | 262.14 | 6 | 677 862 866 1391 1430 1479 |  |  |
|  | [9257157](http://www.ncbi.nlm.nih.gov/entrez/query.fcgi?cmd=Search&db=Protein&term=9257157&doptcmdl=GenPept) | Chain A, Aspartate Aminotransferase From Escherichia Coli, C191f Mutation, With Bound Maleate [MASS=43617] | 262.14 | 6 | 677 862 866 1391 1430 1479 |  |  |
|  | [91210030](http://www.ncbi.nlm.nih.gov/entrez/query.fcgi?cmd=Search&db=Protein&term=91210030&doptcmdl=GenPept) | aromatic amino acid aminotransferase [Escherichia coli UTI89] | 262.14 | 6 | 677 862 866 1391 1430 1479 |  |  |
|  | [91071604](http://www.ncbi.nlm.nih.gov/entrez/query.fcgi?cmd=Search&db=Protein&term=91071604&doptcmdl=GenPept) | aspartate transaminase [Escherichia coli UTI89] | 262.14 | 6 | 677 862 866 1391 1430 1479 |  |  |
|  | [89107778](http://www.ncbi.nlm.nih.gov/entrez/query.fcgi?cmd=Search&db=Protein&term=89107778&doptcmdl=GenPept) | aspartate aminotransferase, PLP-dependent [Escherichia coli W3110] | 262.14 | 6 | 677 862 866 1391 1430 1479 |  |  |
| 113 | [91211810](http://www.ncbi.nlm.nih.gov/entrez/query.fcgi?cmd=Search&db=Protein&term=91211810&doptcmdl=GenPept) | dihydrodipicolinate synthase [Escherichia coli UTI89] | 261.24 | 5 | 329 1032 1071 1225 1331 |  |  |
|  | [91073384](http://www.ncbi.nlm.nih.gov/entrez/query.fcgi?cmd=Search&db=Protein&term=91073384&doptcmdl=GenPept) | dihydrodipicolinate synthase [Escherichia coli UTI89] [MASS=32049] | 261.24 | 5 | 329 1032 1071 1225 1331 |  |  |
|  | [89109284](http://www.ncbi.nlm.nih.gov/entrez/query.fcgi?cmd=Search&db=Protein&term=89109284&doptcmdl=GenPept) | dihydrodipicolinate synthase [Escherichia coli W3110] | 261.24 | 5 | 329 1032 1071 1225 1331 |  |  |
|  | [83586873](http://www.ncbi.nlm.nih.gov/entrez/query.fcgi?cmd=Search&db=Protein&term=83586873&doptcmdl=GenPept) | COG0329: Dihydrodipicolinate synthase/N-acetylneuraminate lyase [Escherichia coli 101-1] | 261.24 | 5 | 329 1032 1071 1225 1331 |  |  |
|  | [83570694](http://www.ncbi.nlm.nih.gov/entrez/query.fcgi?cmd=Search&db=Protein&term=83570694&doptcmdl=GenPept) | COG0329: Dihydrodipicolinate synthase/N-acetylneuraminate lyase [Shigella dysenteriae 1012] | 261.24 | 5 | 329 1032 1071 1225 1331 |  |  |
| 114 | [91212298](http://www.ncbi.nlm.nih.gov/entrez/query.fcgi?cmd=Search&db=Protein&term=91212298&doptcmdl=GenPept) | hypothetical protein UTI89_C3305 [Escherichia coli UTI89] | 261.10 | 4 | 572 1063 1461 1598 |  |  |
|  | [91073872](http://www.ncbi.nlm.nih.gov/entrez/query.fcgi?cmd=Search&db=Protein&term=91073872&doptcmdl=GenPept) | hypothetical protein YggE [Escherichia coli UTI89] [MASS=26627] | 261.10 | 4 | 572 1063 1461 1598 |  |  |
|  | [89109700](http://www.ncbi.nlm.nih.gov/entrez/query.fcgi?cmd=Search&db=Protein&term=89109700&doptcmdl=GenPept) | hypothetical protein [Escherichia coli W3110] | 261.10 | 4 | 572 1063 1461 1598 |  |  |
|  | [882451](http://www.ncbi.nlm.nih.gov/entrez/query.fcgi?cmd=Search&db=Protein&term=882451&doptcmdl=GenPept) | ORF_f246; alternate name yggE'; orf6 of X14436 | 261.10 | 4 | 572 1063 1461 1598 |  |  |
|  | [85675733](http://www.ncbi.nlm.nih.gov/entrez/query.fcgi?cmd=Search&db=Protein&term=85675733&doptcmdl=GenPept) | conserved hypothetical protein [Escherichia coli W3110] | 261.10 | 4 | 572 1063 1461 1598 |  |  |
| 115 | [91211857](http://www.ncbi.nlm.nih.gov/entrez/query.fcgi?cmd=Search&db=Protein&term=91211857&doptcmdl=GenPept) | cysteine desulfurase [Escherichia coli UTI89] | 259.59 | 4 | 723 823 945 1383 |  |  |
|  | [91073431](http://www.ncbi.nlm.nih.gov/entrez/query.fcgi?cmd=Search&db=Protein&term=91073431&doptcmdl=GenPept) | cysteine desulfurase [Escherichia coli UTI89] | 259.59 | 4 | 723 823 945 1383 |  |  |
|  | [89109336](http://www.ncbi.nlm.nih.gov/entrez/query.fcgi?cmd=Search&db=Protein&term=89109336&doptcmdl=GenPept) | cysteine desulfurase (tRNA sulfurtransferase), PLP-dependent [Escherichia coli W3110] | 259.59 | 4 | 723 823 945 1383 |  |  |
|  | [83586820](http://www.ncbi.nlm.nih.gov/entrez/query.fcgi?cmd=Search&db=Protein&term=83586820&doptcmdl=GenPept) | COG1104: Cysteine sulfinate desulfinase/cysteine desulfurase and related enzymes [Escherichia coli 101-1] | 259.59 | 4 | 723 823 945 1383 |  |  |
|  | [82777913](http://www.ncbi.nlm.nih.gov/entrez/query.fcgi?cmd=Search&db=Protein&term=82777913&doptcmdl=GenPept) | putative aminotransferase [Shigella dysenteriae Sd197] | 259.59 | 4 | 723 823 945 1383 |  |  |
| 116 | [9664352](http://www.ncbi.nlm.nih.gov/entrez/query.fcgi?cmd=Search&db=Protein&term=9664352&doptcmdl=GenPept) | isocitrate dehydrogenase [Escherichia coli] | 256.58 | 6 | 51 371 673 704 971 1572 |  |  |
|  | [9664350](http://www.ncbi.nlm.nih.gov/entrez/query.fcgi?cmd=Search&db=Protein&term=9664350&doptcmdl=GenPept) | isocitrate dehydrogenase [Escherichia coli] | 256.58 | 6 | 51 371 673 704 971 1572 |  |  |
|  | [9664348](http://www.ncbi.nlm.nih.gov/entrez/query.fcgi?cmd=Search&db=Protein&term=9664348&doptcmdl=GenPept) | isocitrate dehydrogenase [Escherichia coli] | 256.58 | 6 | 51 371 673 704 971 1572 |  |  |
|  | [9664346](http://www.ncbi.nlm.nih.gov/entrez/query.fcgi?cmd=Search&db=Protein&term=9664346&doptcmdl=GenPept) | isocitrate dehydrogenase [Escherichia coli] | 256.58 | 6 | 51 371 673 704 971 1572 |  |  |
|  | [9664344](http://www.ncbi.nlm.nih.gov/entrez/query.fcgi?cmd=Search&db=Protein&term=9664344&doptcmdl=GenPept) | isocitrate dehydrogenase [Escherichia coli] | 256.58 | 6 | 51 371 673 704 971 1572 |  |  |
| 117 | [91213460](http://www.ncbi.nlm.nih.gov/entrez/query.fcgi?cmd=Search&db=Protein&term=91213460&doptcmdl=GenPept) | triosephosphate isomerase [Escherichia coli UTI89] | 252.87 | 5 | 82 369 925 1027 1715 |  |  |
|  | [91075034](http://www.ncbi.nlm.nih.gov/entrez/query.fcgi?cmd=Search&db=Protein&term=91075034&doptcmdl=GenPept) | triosephosphate isomerase [Escherichia coli UTI89] | 252.87 | 5 | 82 369 925 1027 1715 |  |  |
|  | [89110110](http://www.ncbi.nlm.nih.gov/entrez/query.fcgi?cmd=Search&db=Protein&term=89110110&doptcmdl=GenPept) | triosephosphate isomerase [Escherichia coli W3110] | 252.87 | 5 | 82 369 925 1027 1715 |  |  |
|  | [85676141](http://www.ncbi.nlm.nih.gov/entrez/query.fcgi?cmd=Search&db=Protein&term=85676141&doptcmdl=GenPept) | triosephosphate isomerase [Escherichia coli W3110] | 252.87 | 5 | 82 369 925 1027 1715 |  |  |
|  | [83586237](http://www.ncbi.nlm.nih.gov/entrez/query.fcgi?cmd=Search&db=Protein&term=83586237&doptcmdl=GenPept) | COG0149: Triosephosphate isomerase [Escherichia coli 101-1] [MASS=26238] | 252.87 | 5 | 82 369 925 1027 1715 |  |  |
| 118 | [91212732](http://www.ncbi.nlm.nih.gov/entrez/query.fcgi?cmd=Search&db=Protein&term=91212732&doptcmdl=GenPept) | 30S ribosomal protein S5 [Escherichia coli UTI89] | 249.49 | 4 | 674 858 1578 1579 |  |  |
|  | [91207888](http://www.ncbi.nlm.nih.gov/entrez/query.fcgi?cmd=Search&db=Protein&term=91207888&doptcmdl=GenPept) | 30S ribosomal protein S5 | 249.49 | 4 | 674 858 1578 1579 |  |  |
|  | [91207886](http://www.ncbi.nlm.nih.gov/entrez/query.fcgi?cmd=Search&db=Protein&term=91207886&doptcmdl=GenPept) | 30S ribosomal protein S5 | 249.49 | 4 | 674 858 1578 1579 |  |  |
|  | [91074306](http://www.ncbi.nlm.nih.gov/entrez/query.fcgi?cmd=Search&db=Protein&term=91074306&doptcmdl=GenPept) | 30S ribosomal subunit protein S5 [Escherichia coli UTI89] | 249.49 | 4 | 674 858 1578 1579 |  |  |
|  | [89110707](http://www.ncbi.nlm.nih.gov/entrez/query.fcgi?cmd=Search&db=Protein&term=89110707&doptcmdl=GenPept) | 30S ribosomal subunit protein S5 [Escherichia coli W3110] | 249.49 | 4 | 674 858 1578 1579 |  |  |
| 119 | [91210248](http://www.ncbi.nlm.nih.gov/entrez/query.fcgi?cmd=Search&db=Protein&term=91210248&doptcmdl=GenPept) | 3-oxoacyl-(acyl carrier protein) synthase II [Escherichia coli UTI89] | 247.29 | 5 | 322 345 515 855 1500 |  |  |
|  | [91071822](http://www.ncbi.nlm.nih.gov/entrez/query.fcgi?cmd=Search&db=Protein&term=91071822&doptcmdl=GenPept) | 3-oxoacyl-[acyl-carrier-protein] synthase II [Escherichia coli UTI89] [MASS=43104] | 247.29 | 5 | 322 345 515 855 1500 |  |  |
|  | [89107941](http://www.ncbi.nlm.nih.gov/entrez/query.fcgi?cmd=Search&db=Protein&term=89107941&doptcmdl=GenPept) | 3-oxoacyl-[acyl-carrier-protein] synthase II [Escherichia coli W3110] | 247.29 | 5 | 322 345 515 855 1500 |  |  |
|  | [83569583](http://www.ncbi.nlm.nih.gov/entrez/query.fcgi?cmd=Search&db=Protein&term=83569583&doptcmdl=GenPept) | COG0304: 3-oxoacyl-(acyl-carrier-protein) synthase [Shigella dysenteriae 1012] | 247.29 | 5 | 322 345 515 855 1500 |  |  |
|  | [77416728](http://www.ncbi.nlm.nih.gov/entrez/query.fcgi?cmd=Search&db=Protein&term=77416728&doptcmdl=GenPept) | 3-oxoacyl-[acyl-carrier-protein] synthase 2 (3-oxoacyl-[acyl-carrier-protein] synthase II) (Beta-ketoacyl-ACP synthase II) (KAS II) | 247.29 | 5 | 322 345 515 855 1500 |  |  |
| 120 | [49258635](http://www.ncbi.nlm.nih.gov/entrez/query.fcgi?cmd=Search&db=Protein&term=49258635&doptcmdl=GenPept) | Chain A, Crystal Structure Of Moab From Escherichia Coli [MASS=18498] | 246.86 | 5 | 131 276 758 785 1447 |  |  |
|  | [49258634](http://www.ncbi.nlm.nih.gov/entrez/query.fcgi?cmd=Search&db=Protein&term=49258634&doptcmdl=GenPept) | Chain B, Crystal Structure Of Moab From Escherichia Coli | 246.86 | 5 | 131 276 758 785 1447 |  |  |
| 121 | [91213681](http://www.ncbi.nlm.nih.gov/entrez/query.fcgi?cmd=Search&db=Protein&term=91213681&doptcmdl=GenPept) | CadA, subunit of lysine decarboxylase [Escherichia coli UTI89] | 243.14 | 5 | 344 1265 1356 1472 1539 |  |  |
|  | [91075255](http://www.ncbi.nlm.nih.gov/entrez/query.fcgi?cmd=Search&db=Protein&term=91075255&doptcmdl=GenPept) | CadA, subunit of lysine decarboxylase [Escherichia coli UTI89] [MASS=81478] | 243.14 | 5 | 344 1265 1356 1472 1539 |  |  |
|  | [89110853](http://www.ncbi.nlm.nih.gov/entrez/query.fcgi?cmd=Search&db=Protein&term=89110853&doptcmdl=GenPept) | lysine decarboxylase 1 [Escherichia coli W3110] | 243.14 | 5 | 344 1265 1356 1472 1539 |  |  |
|  | [85676884](http://www.ncbi.nlm.nih.gov/entrez/query.fcgi?cmd=Search&db=Protein&term=85676884&doptcmdl=GenPept) | lysine decarboxylase 1 [Escherichia coli W3110] | 243.14 | 5 | 344 1265 1356 1472 1539 |  |  |
|  | [83587255](http://www.ncbi.nlm.nih.gov/entrez/query.fcgi?cmd=Search&db=Protein&term=83587255&doptcmdl=GenPept) | COG1982: Arginine/lysine/ornithine decarboxylases [Escherichia coli 101-1] [MASS=80253] | 243.14 | 5 | 344 1265 1356 1472 1539 |  |  |

| **Protein IDs*, cont.*** | | | | | | | |
| --- | --- | --- | --- | --- | --- | --- | --- |
| *Grp Nr.* | *Accession Number* | *Protein Name* | *Protein Score* | *Unique PSMs* | *PSM Serial Nrs.* | *Other Grp.* | *Score (other)* |
| 122 | [91211819](http://www.ncbi.nlm.nih.gov/entrez/query.fcgi?cmd=Search&db=Protein&term=91211819&doptcmdl=GenPept) | uracil phosphoribosyltransferase [Escherichia coli UTI89] | 242.47 | 4 | 1094 1110 1442 1548 |  |  |
|  | [91073393](http://www.ncbi.nlm.nih.gov/entrez/query.fcgi?cmd=Search&db=Protein&term=91073393&doptcmdl=GenPept) | uracil phosphoribosyltransferase [Escherichia coli UTI89] [MASS=23559] | 242.47 | 4 | 1094 1110 1442 1548 |  |  |
|  | [90111448](http://www.ncbi.nlm.nih.gov/entrez/query.fcgi?cmd=Search&db=Protein&term=90111448&doptcmdl=GenPept) | uracil phosphoribosyltransferase [Escherichia coli str. K-12 substr. MG1655] | 242.47 | 4 | 1094 1110 1442 1548 |  |  |
|  | [89109304](http://www.ncbi.nlm.nih.gov/entrez/query.fcgi?cmd=Search&db=Protein&term=89109304&doptcmdl=GenPept) | uracil phosphoribosyltransferase [Escherichia coli W3110] | 242.47 | 4 | 1094 1110 1442 1548 |  |  |
|  | [87082118](http://www.ncbi.nlm.nih.gov/entrez/query.fcgi?cmd=Search&db=Protein&term=87082118&doptcmdl=GenPept) | uracil phosphoribosyltransferase [Escherichia coli str. K-12 substr. MG1655] | 242.47 | 4 | 1094 1110 1442 1548 |  |  |
| 123 | [91211127](http://www.ncbi.nlm.nih.gov/entrez/query.fcgi?cmd=Search&db=Protein&term=91211127&doptcmdl=GenPept) | ferritin [Escherichia coli UTI89] | 242.01 | 5 | 26 76 1041 1417 1717 |  |  |
|  | [91072701](http://www.ncbi.nlm.nih.gov/entrez/query.fcgi?cmd=Search&db=Protein&term=91072701&doptcmdl=GenPept) | cytoplasmic ferritin (an iron storage protein) [Escherichia coli UTI89] [MASS=20042] | 242.01 | 5 | 26 76 1041 1417 1717 |  |  |
|  | [89108743](http://www.ncbi.nlm.nih.gov/entrez/query.fcgi?cmd=Search&db=Protein&term=89108743&doptcmdl=GenPept) | cytoplasmic ferritin iron storage protein [Escherichia coli W3110] | 242.01 | 5 | 26 76 1041 1417 1717 |  |  |
|  | [83585498](http://www.ncbi.nlm.nih.gov/entrez/query.fcgi?cmd=Search&db=Protein&term=83585498&doptcmdl=GenPept) | COG1528: Ferritin-like protein [Escherichia coli 101-1] | 242.01 | 5 | 26 76 1041 1417 1717 |  |  |
|  | [82776412](http://www.ncbi.nlm.nih.gov/entrez/query.fcgi?cmd=Search&db=Protein&term=82776412&doptcmdl=GenPept) | ferritin [Shigella dysenteriae Sd197] | 242.01 | 5 | 26 76 1041 1417 1717 |  |  |
| 124 | [91213706](http://www.ncbi.nlm.nih.gov/entrez/query.fcgi?cmd=Search&db=Protein&term=91213706&doptcmdl=GenPept) | fumarate reductase flavoprotein subunit [Escherichia coli UTI89] | 240.32 | 5 | 138 292 666 721 1602 |  |  |
|  | [91075280](http://www.ncbi.nlm.nih.gov/entrez/query.fcgi?cmd=Search&db=Protein&term=91075280&doptcmdl=GenPept) | flavoprotein subunit of fumarate reductase [Escherichia coli UTI89] | 240.32 | 5 | 138 292 666 721 1602 |  |  |
|  | [90108810](http://www.ncbi.nlm.nih.gov/entrez/query.fcgi?cmd=Search&db=Protein&term=90108810&doptcmdl=GenPept) | Chain M, E. Coli Quinol Fumarate Reductase Frda E49q Mutation [MASS=65970] | 240.32 | 5 | 138 292 666 721 1602 |  |  |
|  | [90108806](http://www.ncbi.nlm.nih.gov/entrez/query.fcgi?cmd=Search&db=Protein&term=90108806&doptcmdl=GenPept) | Chain A, E. Coli Quinol Fumarate Reductase Frda E49q Mutation | 240.32 | 5 | 138 292 666 721 1602 |  |  |
|  | [89110877](http://www.ncbi.nlm.nih.gov/entrez/query.fcgi?cmd=Search&db=Protein&term=89110877&doptcmdl=GenPept) | fumarate reductase (anaerobic) catalytic and NAD/flavoprotein subunit [Escherichia coli W3110] | 240.32 | 5 | 138 292 666 721 1602 |  |  |
| 125 | [91073890](http://www.ncbi.nlm.nih.gov/entrez/query.fcgi?cmd=Search&db=Protein&term=91073890&doptcmdl=GenPept) | transketolase 1 [Escherichia coli UTI89] [MASS=73326] | 238.74 | 6 | 99 529 656 810 990 1727 |  |  |
|  | [89109712](http://www.ncbi.nlm.nih.gov/entrez/query.fcgi?cmd=Search&db=Protein&term=89109712&doptcmdl=GenPept) | transketolase 1, thiamin-binding [Escherichia coli W3110] | 238.74 | 6 | 99 529 656 810 990 1727 |  |  |
|  | [882464](http://www.ncbi.nlm.nih.gov/entrez/query.fcgi?cmd=Search&db=Protein&term=882464&doptcmdl=GenPept) | transketolase [MASS=72264] | 238.74 | 6 | 99 529 656 810 990 1727 |  |  |
|  | [85675745](http://www.ncbi.nlm.nih.gov/entrez/query.fcgi?cmd=Search&db=Protein&term=85675745&doptcmdl=GenPept) | transketolase 1, thiamin-binding [Escherichia coli W3110] | 238.74 | 6 | 99 529 656 810 990 1727 |  |  |
|  | [83587665](http://www.ncbi.nlm.nih.gov/entrez/query.fcgi?cmd=Search&db=Protein&term=83587665&doptcmdl=GenPept) | COG0021: Transketolase [Escherichia coli 101-1] | 238.74 | 6 | 99 529 656 810 990 1727 |  |  |
| 126 | [91212055](http://www.ncbi.nlm.nih.gov/entrez/query.fcgi?cmd=Search&db=Protein&term=91212055&doptcmdl=GenPept) | alanyl-tRNA synthetase [Escherichia coli UTI89] | 231.48 | 5 | 286 453 868 1100 1342 |  |  |
|  | [91073629](http://www.ncbi.nlm.nih.gov/entrez/query.fcgi?cmd=Search&db=Protein&term=91073629&doptcmdl=GenPept) | alanyl-tRNA synthetase [Escherichia coli UTI89] | 231.48 | 5 | 286 453 868 1100 1342 |  |  |
|  | [89109484](http://www.ncbi.nlm.nih.gov/entrez/query.fcgi?cmd=Search&db=Protein&term=89109484&doptcmdl=GenPept) | alanyl-tRNA synthetase [Escherichia coli W3110] | 231.48 | 5 | 286 453 868 1100 1342 |  |  |
|  | [83585644](http://www.ncbi.nlm.nih.gov/entrez/query.fcgi?cmd=Search&db=Protein&term=83585644&doptcmdl=GenPept) | COG0013: Alanyl-tRNA synthetase [Escherichia coli 101-1] | 231.48 | 5 | 286 453 868 1100 1342 |  |  |
|  | [83569947](http://www.ncbi.nlm.nih.gov/entrez/query.fcgi?cmd=Search&db=Protein&term=83569947&doptcmdl=GenPept) | COG0013: Alanyl-tRNA synthetase [Shigella dysenteriae 1012] | 231.48 | 5 | 286 453 868 1100 1342 |  |  |
| 127 | [ACCA_SHIFL](http://us.expasy.org/uniprot/ACCA_SHIFL) | Acetyl-coenzyme A carboxylase carboxyl transferase subunit alpha (EC 6.4.1.2) | 228.56 | 3 | 58 1498 1707 |  |  |
|  | [ACCA_ECOLI](http://us.expasy.org/uniprot/ACCA_ECOLI) | Acetyl-coenzyme A carboxylase carboxyl transferase subunit alpha (EC 6.4.1.2) | 228.56 | 3 | 58 1498 1707 |  |  |
|  | [ACCA_ECO57](http://us.expasy.org/uniprot/ACCA_ECO57) | Acetyl-coenzyme A carboxylase carboxyl transferase subunit alpha (EC 6.4.1.2) | 228.56 | 3 | 58 1498 1707 |  |  |
|  | [91209255](http://www.ncbi.nlm.nih.gov/entrez/query.fcgi?cmd=Search&db=Protein&term=91209255&doptcmdl=GenPept) | acetyl-CoA carboxylase carboxyltransferase subunit alpha [Escherichia coli UTI89] | 145.41 | 2 | 1498 1707 |  |  |
| 128 | [91209654](http://www.ncbi.nlm.nih.gov/entrez/query.fcgi?cmd=Search&db=Protein&term=91209654&doptcmdl=GenPept) | alkyl hydroperoxide reductase, F52a subunit; detoxification of hydroperoxides [Escherichia coli UTI89] | 226.58 | 5 | 498 622 1518 1531 1567 |  |  |
|  | [91071228](http://www.ncbi.nlm.nih.gov/entrez/query.fcgi?cmd=Search&db=Protein&term=91071228&doptcmdl=GenPept) | alkyl hydroperoxide reductase, F52a subunit; detoxification of hydroperoxides [Escherichia coli UTI89] | 226.58 | 5 | 498 622 1518 1531 1567 |  |  |
|  | [90111152](http://www.ncbi.nlm.nih.gov/entrez/query.fcgi?cmd=Search&db=Protein&term=90111152&doptcmdl=GenPept) | alkyl hydroperoxide reductase, F52a subunit, FAD/NAD(P)-binding [Escherichia coli str. K-12 substr. MG1655] | 226.58 | 5 | 498 622 1518 1531 1567 |  |  |
|  | [89107474](http://www.ncbi.nlm.nih.gov/entrez/query.fcgi?cmd=Search&db=Protein&term=89107474&doptcmdl=GenPept) | alkyl hydroperoxide reductase, F52a subunit, FAD/NAD(P)-binding [Escherichia coli W3110] | 226.58 | 5 | 498 622 1518 1531 1567 |  |  |
|  | [87081763](http://www.ncbi.nlm.nih.gov/entrez/query.fcgi?cmd=Search&db=Protein&term=87081763&doptcmdl=GenPept) | alkyl hydroperoxide reductase, F52a subunit, FAD/NAD(P)-binding [Escherichia coli str. K-12 substr. MG1655] | 226.58 | 5 | 498 622 1518 1531 1567 |  |  |
| 129 | [91209679](http://www.ncbi.nlm.nih.gov/entrez/query.fcgi?cmd=Search&db=Protein&term=91209679&doptcmdl=GenPept) | D-alanyl-D-alanine carboxypeptidase fraction A [Escherichia coli UTI89] | 222.18 | 5 | 857 1104 1360 1496 1610 |  |  |
|  | [91071253](http://www.ncbi.nlm.nih.gov/entrez/query.fcgi?cmd=Search&db=Protein&term=91071253&doptcmdl=GenPept) | D-alanyl-D-alanine carboxypeptidase, fraction A; penicillin-binding protein 5 [Escherichia coli UTI89] | 222.18 | 5 | 857 1104 1360 1496 1610 |  |  |
|  | [89107501](http://www.ncbi.nlm.nih.gov/entrez/query.fcgi?cmd=Search&db=Protein&term=89107501&doptcmdl=GenPept) | D-alanyl-D-alanine carboxypeptidase [Escherichia coli W3110] | 222.18 | 5 | 857 1104 1360 1496 1610 |  |  |
|  | [83586934](http://www.ncbi.nlm.nih.gov/entrez/query.fcgi?cmd=Search&db=Protein&term=83586934&doptcmdl=GenPept) | COG1686: D-alanyl-D-alanine carboxypeptidase [Escherichia coli 101-1] | 222.18 | 5 | 857 1104 1360 1496 1610 |  |  |
|  | [83288472](http://www.ncbi.nlm.nih.gov/entrez/query.fcgi?cmd=Search&db=Protein&term=83288472&doptcmdl=GenPept) | D-alanyl-D-alanine carboxypeptidase dacA precursor (DD-carboxypeptidase) (DD-peptidase) (Beta-lactamase) (Penicillin-binding protein 5) (PBP-5) | 222.18 | 5 | 857 1104 1360 1496 1610 |  |  |
| 130 | [91211756](http://www.ncbi.nlm.nih.gov/entrez/query.fcgi?cmd=Search&db=Protein&term=91211756&doptcmdl=GenPept) | glucose-specific PTS system component [Escherichia coli UTI89] | 217.70 | 4 | 31 187 496 816 |  |  |
|  | [91073330](http://www.ncbi.nlm.nih.gov/entrez/query.fcgi?cmd=Search&db=Protein&term=91073330&doptcmdl=GenPept) | PTS system, glucose-specific IIA component [Escherichia coli UTI89] | 217.70 | 4 | 31 187 496 816 |  |  |
|  | [89109231](http://www.ncbi.nlm.nih.gov/entrez/query.fcgi?cmd=Search&db=Protein&term=89109231&doptcmdl=GenPept) | glucose-specific enzyme IIA component of PTS [Escherichia coli W3110] | 217.70 | 4 | 31 187 496 816 |  |  |
|  | [85692736](http://www.ncbi.nlm.nih.gov/entrez/query.fcgi?cmd=Search&db=Protein&term=85692736&doptcmdl=GenPept) | Glucose-specific phosphotransferase enzyme IIA component (PTS system glucose-specific EIIA component) (EIIA-Glc) (EIII-Glc) | 217.70 | 4 | 31 187 496 816 |  |  |
|  | [83586925](http://www.ncbi.nlm.nih.gov/entrez/query.fcgi?cmd=Search&db=Protein&term=83586925&doptcmdl=GenPept) | COG2190: Phosphotransferase system IIA components [Escherichia coli 101-1] | 217.70 | 4 | 31 187 496 816 |  |  |
| 131 | [91211838](http://www.ncbi.nlm.nih.gov/entrez/query.fcgi?cmd=Search&db=Protein&term=91211838&doptcmdl=GenPept) | outer membrane protein assembly complex subunit YfgL [Escherichia coli UTI89] | 211.97 | 3 | 311 566 1209 |  |  |
|  | [91073412](http://www.ncbi.nlm.nih.gov/entrez/query.fcgi?cmd=Search&db=Protein&term=91073412&doptcmdl=GenPept) | hypothetical protein YfgL [Escherichia coli UTI89] | 211.97 | 3 | 311 566 1209 |  |  |
|  | [89109318](http://www.ncbi.nlm.nih.gov/entrez/query.fcgi?cmd=Search&db=Protein&term=89109318&doptcmdl=GenPept) | protein assembly complex, lipoprotein component [Escherichia coli W3110] | 211.97 | 3 | 311 566 1209 |  |  |
|  | [83586839](http://www.ncbi.nlm.nih.gov/entrez/query.fcgi?cmd=Search&db=Protein&term=83586839&doptcmdl=GenPept) | COG1520: FOG: WD40-like repeat [Escherichia coli 101-1] | 211.97 | 3 | 311 566 1209 |  |  |
|  | [82777897](http://www.ncbi.nlm.nih.gov/entrez/query.fcgi?cmd=Search&db=Protein&term=82777897&doptcmdl=GenPept) | outer membrane protein assembly complex subunit YfgL [Shigella dysenteriae Sd197] | 211.97 | 3 | 311 566 1209 |  |  |
| 132 | [91210640](http://www.ncbi.nlm.nih.gov/entrez/query.fcgi?cmd=Search&db=Protein&term=91210640&doptcmdl=GenPept) | hypothetical protein UTI89_C1617 [Escherichia coli UTI89] | 211.75 | 4 | 220 517 747 1515 |  |  |
|  | [91072214](http://www.ncbi.nlm.nih.gov/entrez/query.fcgi?cmd=Search&db=Protein&term=91072214&doptcmdl=GenPept) | hypothetical protein UTI89_C1617 [Escherichia coli UTI89] | 211.75 | 4 | 220 517 747 1515 |  |  |
|  | [90111262](http://www.ncbi.nlm.nih.gov/entrez/query.fcgi?cmd=Search&db=Protein&term=90111262&doptcmdl=GenPept) | stress-induced protein, ATP-binding protein [Escherichia coli str. K-12 substr. MG1655] | 211.75 | 4 | 220 517 747 1515 |  |  |
|  | [89108222](http://www.ncbi.nlm.nih.gov/entrez/query.fcgi?cmd=Search&db=Protein&term=89108222&doptcmdl=GenPept) | stress-induced protein, ATP-binding protein [Escherichia coli W3110] | 211.75 | 4 | 220 517 747 1515 |  |  |
|  | [87081893](http://www.ncbi.nlm.nih.gov/entrez/query.fcgi?cmd=Search&db=Protein&term=87081893&doptcmdl=GenPept) | stress-induced protein, ATP-binding protein [Escherichia coli str. K-12 substr. MG1655] | 211.75 | 4 | 220 517 747 1515 |  |  |
| 133 | [91211840](http://www.ncbi.nlm.nih.gov/entrez/query.fcgi?cmd=Search&db=Protein&term=91211840&doptcmdl=GenPept) | histidyl-tRNA synthetase [Escherichia coli UTI89] | 211.20 | 4 | 561 931 1276 1474 |  |  |
|  | [91073414](http://www.ncbi.nlm.nih.gov/entrez/query.fcgi?cmd=Search&db=Protein&term=91073414&doptcmdl=GenPept) | histidine tRNA synthetase [Escherichia coli UTI89] | 211.20 | 4 | 561 931 1276 1474 |  |  |
|  | [89109320](http://www.ncbi.nlm.nih.gov/entrez/query.fcgi?cmd=Search&db=Protein&term=89109320&doptcmdl=GenPept) | histidyl tRNA synthetase [Escherichia coli W3110] | 211.20 | 4 | 561 931 1276 1474 |  |  |
|  | [83586837](http://www.ncbi.nlm.nih.gov/entrez/query.fcgi?cmd=Search&db=Protein&term=83586837&doptcmdl=GenPept) | COG0124: Histidyl-tRNA synthetase [Escherichia coli 101-1] | 211.20 | 4 | 561 931 1276 1474 |  |  |
|  | [75211480](http://www.ncbi.nlm.nih.gov/entrez/query.fcgi?cmd=Search&db=Protein&term=75211480&doptcmdl=GenPept) | COG0124: Histidyl-tRNA synthetase [Escherichia coli B171] | 211.20 | 4 | 561 931 1276 1474 |  |  |
| 134 | [91212998](http://www.ncbi.nlm.nih.gov/entrez/query.fcgi?cmd=Search&db=Protein&term=91212998&doptcmdl=GenPept) | starvation induced outer membrane protein [Escherichia coli UTI89] | 209.01 | 4 | 119 735 1040 1390 |  |  |
|  | [91074572](http://www.ncbi.nlm.nih.gov/entrez/query.fcgi?cmd=Search&db=Protein&term=91074572&doptcmdl=GenPept) | starvation induced outer membrane protein [Escherichia coli UTI89] | 209.01 | 4 | 119 735 1040 1390 |  |  |
|  | [90111603](http://www.ncbi.nlm.nih.gov/entrez/query.fcgi?cmd=Search&db=Protein&term=90111603&doptcmdl=GenPept) | outer membrane lipoprotein [Escherichia coli str. K-12 substr. MG1655] | 209.01 | 4 | 119 735 1040 1390 |  |  |
|  | [89110507](http://www.ncbi.nlm.nih.gov/entrez/query.fcgi?cmd=Search&db=Protein&term=89110507&doptcmdl=GenPept) | outer membrane lipoprotein [Escherichia coli W3110] | 209.01 | 4 | 119 735 1040 1390 |  |  |
|  | [87082278](http://www.ncbi.nlm.nih.gov/entrez/query.fcgi?cmd=Search&db=Protein&term=87082278&doptcmdl=GenPept) | outer membrane lipoprotein [Escherichia coli str. K-12 substr. MG1655] | 209.01 | 4 | 119 735 1040 1390 |  |  |
| 135 | [91209548](http://www.ncbi.nlm.nih.gov/entrez/query.fcgi?cmd=Search&db=Protein&term=91209548&doptcmdl=GenPept) | adenylate kinase [Escherichia coli UTI89] | 208.72 | 3 | 408 900 1666 |  |  |
|  | [91071122](http://www.ncbi.nlm.nih.gov/entrez/query.fcgi?cmd=Search&db=Protein&term=91071122&doptcmdl=GenPept) | adenylate kinase [Escherichia coli UTI89] [MASS=25805] | 208.72 | 3 | 408 900 1666 |  |  |
|  | [89107343](http://www.ncbi.nlm.nih.gov/entrez/query.fcgi?cmd=Search&db=Protein&term=89107343&doptcmdl=GenPept) | adenylate kinase [Escherichia coli W3110] | 208.72 | 3 | 408 900 1666 |  |  |
|  | [85674613](http://www.ncbi.nlm.nih.gov/entrez/query.fcgi?cmd=Search&db=Protein&term=85674613&doptcmdl=GenPept) | adenylate kinase [Escherichia coli W3110] | 208.72 | 3 | 408 900 1666 |  |  |
|  | [83587071](http://www.ncbi.nlm.nih.gov/entrez/query.fcgi?cmd=Search&db=Protein&term=83587071&doptcmdl=GenPept) | COG0563: Adenylate kinase and related kinases [Escherichia coli 101-1] | 208.72 | 3 | 408 900 1666 |  |  |
| 136 | [91209757](http://www.ncbi.nlm.nih.gov/entrez/query.fcgi?cmd=Search&db=Protein&term=91209757&doptcmdl=GenPept) | succinate dehydrogenase flavoprotein subunit [Escherichia coli UTI89] | 207.59 | 3 | 72 466 1636 |  |  |
|  | [91071331](http://www.ncbi.nlm.nih.gov/entrez/query.fcgi?cmd=Search&db=Protein&term=91071331&doptcmdl=GenPept) | succinate dehydrogenase flavoprotein subunit [Escherichia coli UTI89] | 207.59 | 3 | 72 466 1636 |  |  |
|  | [89107581](http://www.ncbi.nlm.nih.gov/entrez/query.fcgi?cmd=Search&db=Protein&term=89107581&doptcmdl=GenPept) | succinate dehydrogenase, flavoprotein subunit [Escherichia coli W3110] | 207.59 | 3 | 72 466 1636 |  |  |
|  | [85544084](http://www.ncbi.nlm.nih.gov/entrez/query.fcgi?cmd=Search&db=Protein&term=85544084&doptcmdl=GenPept) | Chain A, Complex Ii (Succinate Dehydrogenase) From E. Coli With Atpenin A5 Inhibitor Co-Crystallized At The Ubiquinone Binding Site | 207.59 | 3 | 72 466 1636 |  |  |
|  | [83585345](http://www.ncbi.nlm.nih.gov/entrez/query.fcgi?cmd=Search&db=Protein&term=83585345&doptcmdl=GenPept) | COG1053: Succinate dehydrogenase/fumarate reductase, flavoprotein subunit [Escherichia coli 101-1] | 207.59 | 3 | 72 466 1636 |  |  |
| 137 | [91209777](http://www.ncbi.nlm.nih.gov/entrez/query.fcgi?cmd=Search&db=Protein&term=91209777&doptcmdl=GenPept) | hypothetical protein UTI89_C0739 [Escherichia coli UTI89] | 206.85 | 3 | 829 1480 1691 |  |  |
|  | [91071351](http://www.ncbi.nlm.nih.gov/entrez/query.fcgi?cmd=Search&db=Protein&term=91071351&doptcmdl=GenPept) | hypothetical protein UTI89_C0739 [Escherichia coli UTI89] | 206.85 | 3 | 829 1480 1691 |  |  |
|  | [89107600](http://www.ncbi.nlm.nih.gov/entrez/query.fcgi?cmd=Search&db=Protein&term=89107600&doptcmdl=GenPept) | hypothetical protein [Escherichia coli W3110] | 206.85 | 3 | 829 1480 1691 |  |  |
|  | [83584447](http://www.ncbi.nlm.nih.gov/entrez/query.fcgi?cmd=Search&db=Protein&term=83584447&doptcmdl=GenPept) | COG1729: Uncharacterized protein conserved in bacteria [Escherichia coli 101-1] | 206.85 | 3 | 829 1480 1691 |  |  |
|  | [75207819](http://www.ncbi.nlm.nih.gov/entrez/query.fcgi?cmd=Search&db=Protein&term=75207819&doptcmdl=GenPept) | COG1729: Uncharacterized protein conserved in bacteria [Escherichia coli B171] | 206.85 | 3 | 829 1480 1691 |  |  |
| 138 | [91211860](http://www.ncbi.nlm.nih.gov/entrez/query.fcgi?cmd=Search&db=Protein&term=91211860&doptcmdl=GenPept) | inositol monophosphatase [Escherichia coli UTI89] | 206.52 | 4 | 17 428 514 1210 |  |  |
|  | [91073434](http://www.ncbi.nlm.nih.gov/entrez/query.fcgi?cmd=Search&db=Protein&term=91073434&doptcmdl=GenPept) | inositol monophosphatase [Escherichia coli UTI89] | 206.52 | 4 | 17 428 514 1210 |  |  |
|  | [89109339](http://www.ncbi.nlm.nih.gov/entrez/query.fcgi?cmd=Search&db=Protein&term=89109339&doptcmdl=GenPept) | inositol monophosphatase [Escherichia coli W3110] | 206.52 | 4 | 17 428 514 1210 |  |  |
|  | [83586817](http://www.ncbi.nlm.nih.gov/entrez/query.fcgi?cmd=Search&db=Protein&term=83586817&doptcmdl=GenPept) | COG0483: Archaeal fructose-1,6-bisphosphatase and related enzymes of inositol monophosphatase family [Escherichia coli 101-1] | 206.52 | 4 | 17 428 514 1210 |  |  |
|  | [83570753](http://www.ncbi.nlm.nih.gov/entrez/query.fcgi?cmd=Search&db=Protein&term=83570753&doptcmdl=GenPept) | COG0483: Archaeal fructose-1,6-bisphosphatase and related enzymes of inositol monophosphatase family [Shigella dysenteriae 1012] | 206.52 | 4 | 17 428 514 1210 |  |  |
| 139 | [91213723](http://www.ncbi.nlm.nih.gov/entrez/query.fcgi?cmd=Search&db=Protein&term=91213723&doptcmdl=GenPept) | FtsH protease regulator HflK [Escherichia coli UTI89] | 206.24 | 3 | 418 694 1537 |  |  |
|  | [91075297](http://www.ncbi.nlm.nih.gov/entrez/query.fcgi?cmd=Search&db=Protein&term=91075297&doptcmdl=GenPept) | HflK protein regulator of FtsH protease, subunit of HflK-HflC complex [Escherichia coli UTI89] | 206.24 | 3 | 418 694 1537 |  |  |
|  | [89110894](http://www.ncbi.nlm.nih.gov/entrez/query.fcgi?cmd=Search&db=Protein&term=89110894&doptcmdl=GenPept) | modulator for HflB protease specific for phage lambda cII repressor [Escherichia coli W3110] | 206.24 | 3 | 418 694 1537 |  |  |
|  | [85676925](http://www.ncbi.nlm.nih.gov/entrez/query.fcgi?cmd=Search&db=Protein&term=85676925&doptcmdl=GenPept) | modulator for HflB protease specific for phage lambda cII repressor [Escherichia coli W3110] | 206.24 | 3 | 418 694 1537 |  |  |
|  | [83585088](http://www.ncbi.nlm.nih.gov/entrez/query.fcgi?cmd=Search&db=Protein&term=83585088&doptcmdl=GenPept) | COG0330: Membrane protease subunits, stomatin/prohibitin homologs [Escherichia coli 101-1] | 206.24 | 3 | 418 694 1537 |  |  |
| 140 | [91212817](http://www.ncbi.nlm.nih.gov/entrez/query.fcgi?cmd=Search&db=Protein&term=91212817&doptcmdl=GenPept) | 30S ribosomal protein S7 [Escherichia coli UTI89] | 204.38 | 3 | 104 450 695 |  |  |
|  | [91074391](http://www.ncbi.nlm.nih.gov/entrez/query.fcgi?cmd=Search&db=Protein&term=91074391&doptcmdl=GenPept) | 30S ribosomal subunit protein S7 [Escherichia coli UTI89] | 204.38 | 3 | 104 450 695 |  |  |
|  | [90120558](http://www.ncbi.nlm.nih.gov/entrez/query.fcgi?cmd=Search&db=Protein&term=90120558&doptcmdl=GenPept) | 30S ribosomal protein S7 | 204.38 | 3 | 104 450 695 |  |  |
|  | [90120547](http://www.ncbi.nlm.nih.gov/entrez/query.fcgi?cmd=Search&db=Protein&term=90120547&doptcmdl=GenPept) | 30S ribosomal protein S7 | 204.38 | 3 | 104 450 695 |  |  |
|  | [89110669](http://www.ncbi.nlm.nih.gov/entrez/query.fcgi?cmd=Search&db=Protein&term=89110669&doptcmdl=GenPept) | 30S ribosomal subunit protein S7 [Escherichia coli W3110] | 204.38 | 3 | 104 450 695 |  |  |
| 141 | [91209843](http://www.ncbi.nlm.nih.gov/entrez/query.fcgi?cmd=Search&db=Protein&term=91209843&doptcmdl=GenPept) | glutamine ABC transporter ATP-binding protein [Escherichia coli UTI89] | 202.61 | 3 | 273 806 1406 |  |  |
|  | [91071417](http://www.ncbi.nlm.nih.gov/entrez/query.fcgi?cmd=Search&db=Protein&term=91071417&doptcmdl=GenPept) | GlnQ protein, subunit of glutamine ABC transporter [Escherichia coli UTI89] | 202.61 | 3 | 273 806 1406 |  |  |
|  | [89107660](http://www.ncbi.nlm.nih.gov/entrez/query.fcgi?cmd=Search&db=Protein&term=89107660&doptcmdl=GenPept) | glutamine transporter subunit [Escherichia coli W3110] | 202.61 | 3 | 273 806 1406 |  |  |
|  | [83586112](http://www.ncbi.nlm.nih.gov/entrez/query.fcgi?cmd=Search&db=Protein&term=83586112&doptcmdl=GenPept) | COG1126: ABC-type polar amino acid transport system, ATPase component [Escherichia coli 101-1] | 202.61 | 3 | 273 806 1406 |  |  |
|  | [82543255](http://www.ncbi.nlm.nih.gov/entrez/query.fcgi?cmd=Search&db=Protein&term=82543255&doptcmdl=GenPept) | glutamine ABC transporter ATP-binding protein [Shigella boydii Sb227] | 202.61 | 3 | 273 806 1406 |  |  |
| 142 | [91212726](http://www.ncbi.nlm.nih.gov/entrez/query.fcgi?cmd=Search&db=Protein&term=91212726&doptcmdl=GenPept) | 30S ribosomal protein S13 [Escherichia coli UTI89] | 202.21 | 4 | 611 1022 1030 1116 |  |  |
|  | [91207726](http://www.ncbi.nlm.nih.gov/entrez/query.fcgi?cmd=Search&db=Protein&term=91207726&doptcmdl=GenPept) | 30S ribosomal protein S13 | 202.21 | 4 | 611 1022 1030 1116 |  |  |
|  | [91207725](http://www.ncbi.nlm.nih.gov/entrez/query.fcgi?cmd=Search&db=Protein&term=91207725&doptcmdl=GenPept) | 30S ribosomal protein S13 | 202.21 | 4 | 611 1022 1030 1116 |  |  |
|  | [91207724](http://www.ncbi.nlm.nih.gov/entrez/query.fcgi?cmd=Search&db=Protein&term=91207724&doptcmdl=GenPept) | 30S ribosomal protein S13 | 202.21 | 4 | 611 1022 1030 1116 |  |  |
|  | [91074300](http://www.ncbi.nlm.nih.gov/entrez/query.fcgi?cmd=Search&db=Protein&term=91074300&doptcmdl=GenPept) | 30S ribosomal subunit protein S13 [Escherichia coli UTI89] | 202.21 | 4 | 611 1022 1030 1116 |  |  |
| 143 | [91209776](http://www.ncbi.nlm.nih.gov/entrez/query.fcgi?cmd=Search&db=Protein&term=91209776&doptcmdl=GenPept) | peptidoglycan-associated outer membrane lipoprotein [Escherichia coli UTI89] | 199.83 | 3 | 121 792 949 |  |  |
|  | [91071350](http://www.ncbi.nlm.nih.gov/entrez/query.fcgi?cmd=Search&db=Protein&term=91071350&doptcmdl=GenPept) | peptidoglycan-associated lipoprotein precursor [Escherichia coli UTI89] [MASS=19681] | 199.83 | 3 | 121 792 949 |  |  |
|  | [89107599](http://www.ncbi.nlm.nih.gov/entrez/query.fcgi?cmd=Search&db=Protein&term=89107599&doptcmdl=GenPept) | peptidoglycan-associated outer membrane lipoprotein [Escherichia coli W3110] | 199.83 | 3 | 121 792 949 |  |  |
|  | [83584446](http://www.ncbi.nlm.nih.gov/entrez/query.fcgi?cmd=Search&db=Protein&term=83584446&doptcmdl=GenPept) | COG2885: Outer membrane protein and related peptidoglycan-associated (lipo)proteins [Escherichia coli 101-1] | 199.83 | 3 | 121 792 949 |  |  |
|  | [82543168](http://www.ncbi.nlm.nih.gov/entrez/query.fcgi?cmd=Search&db=Protein&term=82543168&doptcmdl=GenPept) | peptidoglycan-associated outer membrane lipoprotein [Shigella boydii Sb227] | 199.83 | 3 | 121 792 949 |  |  |
| 144 | [91212527](http://www.ncbi.nlm.nih.gov/entrez/query.fcgi?cmd=Search&db=Protein&term=91212527&doptcmdl=GenPept) | hypothetical protein UTI89_C3536 [Escherichia coli UTI89] | 199.04 | 3 | 205 325 1663 |  |  |
|  | [91074101](http://www.ncbi.nlm.nih.gov/entrez/query.fcgi?cmd=Search&db=Protein&term=91074101&doptcmdl=GenPept) | hypothetical protein UTI89_C3536 [Escherichia coli UTI89] | 199.04 | 3 | 205 325 1663 |  |  |
|  | [89109867](http://www.ncbi.nlm.nih.gov/entrez/query.fcgi?cmd=Search&db=Protein&term=89109867&doptcmdl=GenPept) | hypothetical protein [Escherichia coli W3110] | 199.04 | 3 | 205 325 1663 |  |  |
|  | [85675898](http://www.ncbi.nlm.nih.gov/entrez/query.fcgi?cmd=Search&db=Protein&term=85675898&doptcmdl=GenPept) | conserved hypothetical protein [Escherichia coli W3110] | 199.04 | 3 | 205 325 1663 |  |  |
|  | [83585742](http://www.ncbi.nlm.nih.gov/entrez/query.fcgi?cmd=Search&db=Protein&term=83585742&doptcmdl=GenPept) | COG4575: Uncharacterized conserved protein [Escherichia coli 101-1] | 199.04 | 3 | 205 325 1663 |  |  |
| 145 | [968934](http://www.ncbi.nlm.nih.gov/entrez/query.fcgi?cmd=Search&db=Protein&term=968934&doptcmdl=GenPept) | 3-deoxy-D-manno-octulosonic acid 8-phosphate synthetase | 198.48 | 6 | 68 364 542 595 1277 1380 |  |  |
|  | [940498](http://www.ncbi.nlm.nih.gov/entrez/query.fcgi?cmd=Search&db=Protein&term=940498&doptcmdl=GenPept) | 3-deoxy-D-manno-octulosonic acid 8-phosphate synthetase [Escherichia coli] | 198.48 | 6 | 68 364 542 595 1277 1380 |  |  |
|  | [91210436](http://www.ncbi.nlm.nih.gov/entrez/query.fcgi?cmd=Search&db=Protein&term=91210436&doptcmdl=GenPept) | 2-dehydro-3-deoxyphosphooctonate aldolase [Escherichia coli UTI89] | 198.48 | 6 | 68 364 542 595 1277 1380 |  |  |
|  | [91072010](http://www.ncbi.nlm.nih.gov/entrez/query.fcgi?cmd=Search&db=Protein&term=91072010&doptcmdl=GenPept) | 2-dehydro-3-deoxyphosphooctulonate aldolase [Escherichia coli UTI89] | 198.48 | 6 | 68 364 542 595 1277 1380 |  |  |
|  | [89108060](http://www.ncbi.nlm.nih.gov/entrez/query.fcgi?cmd=Search&db=Protein&term=89108060&doptcmdl=GenPept) | 3-deoxy-D-manno-octulosonate 8-phosphate synthase [Escherichia coli W3110] | 198.48 | 6 | 68 364 542 595 1277 1380 |  |  |

| **Protein IDs*, cont.*** | | | | | | | |
| --- | --- | --- | --- | --- | --- | --- | --- |
| *Grp Nr.* | *Accession Number* | *Protein Name* | *Protein Score* | *Unique PSMs* | *PSM Serial Nrs.* | *Other Grp.* | *Score (other)* |
| 146 | [91213254](http://www.ncbi.nlm.nih.gov/entrez/query.fcgi?cmd=Search&db=Protein&term=91213254&doptcmdl=GenPept) | bifunctional N-acetylglucosamine-1-phosphate uridyltransferase/glucosamine-1-phosphate acetyltransferase [Escherichia coli UTI89] | 195.76 | 3 | 90 343 410 |  |  |
|  | [91074828](http://www.ncbi.nlm.nih.gov/entrez/query.fcgi?cmd=Search&db=Protein&term=91074828&doptcmdl=GenPept) | bifunctional N-acetyl glucosamine-1-phosphate uridyltransferase/glucosamine-1-phosphate acetyltransferase [Escherichia coli UTI89] | 195.76 | 3 | 90 343 410 |  |  |
|  | [89110277](http://www.ncbi.nlm.nih.gov/entrez/query.fcgi?cmd=Search&db=Protein&term=89110277&doptcmdl=GenPept) | fused N-acetyl glucosamine-1-phosphate uridyltransferase and glucosamine-1-phosphate acetyl transferase [Escherichia coli W3110] | 195.76 | 3 | 90 343 410 |  |  |
|  | [85676308](http://www.ncbi.nlm.nih.gov/entrez/query.fcgi?cmd=Search&db=Protein&term=85676308&doptcmdl=GenPept) | fused N-acetyl glucosamine-1-phosphate uridyltransferase and glucosamine-1-phosphate acetyl transferase [Escherichia coli W3110] | 195.76 | 3 | 90 343 410 |  |  |
|  | [83588405](http://www.ncbi.nlm.nih.gov/entrez/query.fcgi?cmd=Search&db=Protein&term=83588405&doptcmdl=GenPept) | COG1207: N-acetylglucosamine-1-phosphate uridyltransferase (contains nucleotidyltransferase and I-patch acetyltransferase domains) [Escherichia coli 101-1] [MASS=48646] | 195.76 | 3 | 90 343 410 |  |  |
| 147 | [91210646](http://www.ncbi.nlm.nih.gov/entrez/query.fcgi?cmd=Search&db=Protein&term=91210646&doptcmdl=GenPept) | fermentative D-lactate dehydrogenase, NAD-dependent [Escherichia coli UTI89] | 195.39 | 3 | 763 989 1238 |  |  |
|  | [91072220](http://www.ncbi.nlm.nih.gov/entrez/query.fcgi?cmd=Search&db=Protein&term=91072220&doptcmdl=GenPept) | fermentative D-lactate dehydrogenase, NAD-dependent [Escherichia coli UTI89] | 195.39 | 3 | 763 989 1238 |  |  |
|  | [89108227](http://www.ncbi.nlm.nih.gov/entrez/query.fcgi?cmd=Search&db=Protein&term=89108227&doptcmdl=GenPept) | fermentative D-lactate dehydrogenase, NAD-dependent [Escherichia coli W3110] | 195.39 | 3 | 763 989 1238 |  |  |
|  | [83587753](http://www.ncbi.nlm.nih.gov/entrez/query.fcgi?cmd=Search&db=Protein&term=83587753&doptcmdl=GenPept) | COG1052: Lactate dehydrogenase and related dehydrogenases [Escherichia coli 101-1] | 195.39 | 3 | 763 989 1238 |  |  |
|  | [83571037](http://www.ncbi.nlm.nih.gov/entrez/query.fcgi?cmd=Search&db=Protein&term=83571037&doptcmdl=GenPept) | COG1052: Lactate dehydrogenase and related dehydrogenases [Shigella dysenteriae 1012] | 195.39 | 3 | 763 989 1238 |  |  |
| 148 | [91212812](http://www.ncbi.nlm.nih.gov/entrez/query.fcgi?cmd=Search&db=Protein&term=91212812&doptcmdl=GenPept) | 50S ribosomal protein L11 [Escherichia coli UTI89] | 194.96 | 3 | 415 671 1645 |  |  |
|  | [91074386](http://www.ncbi.nlm.nih.gov/entrez/query.fcgi?cmd=Search&db=Protein&term=91074386&doptcmdl=GenPept) | 50S ribosomal subunit protein L11 [Escherichia coli UTI89] | 194.96 | 3 | 415 671 1645 |  |  |
|  | [89110056](http://www.ncbi.nlm.nih.gov/entrez/query.fcgi?cmd=Search&db=Protein&term=89110056&doptcmdl=GenPept) | 50S ribosomal subunit protein L11 [Escherichia coli W3110] | 194.96 | 3 | 415 671 1645 |  |  |
|  | [85676087](http://www.ncbi.nlm.nih.gov/entrez/query.fcgi?cmd=Search&db=Protein&term=85676087&doptcmdl=GenPept) | 50S ribosomal subunit protein L11 [Escherichia coli W3110] | 194.96 | 3 | 415 671 1645 |  |  |
|  | [83754147](http://www.ncbi.nlm.nih.gov/entrez/query.fcgi?cmd=Search&db=Protein&term=83754147&doptcmdl=GenPept) | Chain I, Crystal Structure Of The Bacterial Ribosome From Escherichia Coli At 3.5 A Resolution. This File Contains The 50s Subunit Of The Second 70s Ribosome. The Entire Crystal Structure Contains Two 70s Ribosomes And Is Described In Remark 400. | 194.96 | 3 | 415 671 1645 |  |  |
| 149 | [91210457](http://www.ncbi.nlm.nih.gov/entrez/query.fcgi?cmd=Search&db=Protein&term=91210457&doptcmdl=GenPept) | UTP--glucose-1-phosphate uridylyltransferase subunit GalU [Escherichia coli UTI89] | 194.44 | 3 | 16 856 878 |  |  |
|  | [91072031](http://www.ncbi.nlm.nih.gov/entrez/query.fcgi?cmd=Search&db=Protein&term=91072031&doptcmdl=GenPept) | glucose-1-phosphate uridylyltransferase [Escherichia coli UTI89] | 194.44 | 3 | 16 856 878 |  |  |
|  | [89108082](http://www.ncbi.nlm.nih.gov/entrez/query.fcgi?cmd=Search&db=Protein&term=89108082&doptcmdl=GenPept) | glucose-1-phosphate uridylyltransferase [Escherichia coli W3110] | 194.44 | 3 | 16 856 878 |  |  |
|  | [84028328](http://www.ncbi.nlm.nih.gov/entrez/query.fcgi?cmd=Search&db=Protein&term=84028328&doptcmdl=GenPept) | UTP--glucose-1-phosphate uridylyltransferase (UDP-glucose pyrophosphorylase) (UDPGP) (Alpha-D-glucosyl-1-phosphate uridylyltransferase) (Uridine diphosphoglucose pyrophosphorylase) | 194.44 | 3 | 16 856 878 |  |  |
|  | [84028327](http://www.ncbi.nlm.nih.gov/entrez/query.fcgi?cmd=Search&db=Protein&term=84028327&doptcmdl=GenPept) | UTP--glucose-1-phosphate uridylyltransferase (UDP-glucose pyrophosphorylase) (UDPGP) (Alpha-D-glucosyl-1-phosphate uridylyltransferase) (Uridine diphosphoglucose pyrophosphorylase) | 194.44 | 3 | 16 856 878 |  |  |
| 150 | [91209091](http://www.ncbi.nlm.nih.gov/entrez/query.fcgi?cmd=Search&db=Protein&term=91209091&doptcmdl=GenPept) | carbamoyl phosphate synthase small subunit [Escherichia coli UTI89] | 192.11 | 4 | 168 191 1078 1089 |  |  |
|  | [91070665](http://www.ncbi.nlm.nih.gov/entrez/query.fcgi?cmd=Search&db=Protein&term=91070665&doptcmdl=GenPept) | carbamoyl-phosphate synthase small chain [Escherichia coli UTI89] | 192.11 | 4 | 168 191 1078 1089 |  |  |
|  | [89106916](http://www.ncbi.nlm.nih.gov/entrez/query.fcgi?cmd=Search&db=Protein&term=89106916&doptcmdl=GenPept) | carbamoyl phosphate synthetase small subunit, glutamine amidotransferase [Escherichia coli W3110] | 192.11 | 4 | 168 191 1078 1089 |  |  |
|  | [83584924](http://www.ncbi.nlm.nih.gov/entrez/query.fcgi?cmd=Search&db=Protein&term=83584924&doptcmdl=GenPept) | COG0505: Carbamoylphosphate synthase small subunit [Escherichia coli 101-1] [MASS=42409] | 192.11 | 4 | 168 191 1078 1089 |  |  |
|  | [82542648](http://www.ncbi.nlm.nih.gov/entrez/query.fcgi?cmd=Search&db=Protein&term=82542648&doptcmdl=GenPept) | carbamoyl phosphate synthase small subunit [Shigella boydii Sb227] | 192.11 | 4 | 168 191 1078 1089 |  |  |
| 151 | [91209663](http://www.ncbi.nlm.nih.gov/entrez/query.fcgi?cmd=Search&db=Protein&term=91209663&doptcmdl=GenPept) | citrate lyase alpha chain [Escherichia coli UTI89] | 189.21 | 4 | 1113 1130 1200 1248 |  |  |
|  | [91071237](http://www.ncbi.nlm.nih.gov/entrez/query.fcgi?cmd=Search&db=Protein&term=91071237&doptcmdl=GenPept) | citrate lyase alpha chain [Escherichia coli UTI89] | 189.21 | 4 | 1113 1130 1200 1248 |  |  |
|  | [89107482](http://www.ncbi.nlm.nih.gov/entrez/query.fcgi?cmd=Search&db=Protein&term=89107482&doptcmdl=GenPept) | citrate lyase, citrate-ACP transferase (alpha) subunit [Escherichia coli W3110] | 189.21 | 4 | 1113 1130 1200 1248 |  |  |
|  | [85674724](http://www.ncbi.nlm.nih.gov/entrez/query.fcgi?cmd=Search&db=Protein&term=85674724&doptcmdl=GenPept) | citrate lyase, citrate-ACP transferase (alpha) subunit [Escherichia coli W3110] | 189.21 | 4 | 1113 1130 1200 1248 |  |  |
|  | [83586950](http://www.ncbi.nlm.nih.gov/entrez/query.fcgi?cmd=Search&db=Protein&term=83586950&doptcmdl=GenPept) | COG3051: Citrate lyase, alpha subunit [Escherichia coli 101-1] [MASS=55171] | 189.21 | 4 | 1113 1130 1200 1248 |  |  |
| 152 | [91213136](http://www.ncbi.nlm.nih.gov/entrez/query.fcgi?cmd=Search&db=Protein&term=91213136&doptcmdl=GenPept) | 2-amino-3-ketobutyrate coenzyme A ligase [Escherichia coli UTI89] | 185.94 | 3 | 958 1411 1677 |  |  |
|  | [91074710](http://www.ncbi.nlm.nih.gov/entrez/query.fcgi?cmd=Search&db=Protein&term=91074710&doptcmdl=GenPept) | 2-amino-3-ketobutyrate coenzyme A ligase [Escherichia coli UTI89] [MASS=43133] | 185.94 | 3 | 958 1411 1677 |  |  |
|  | [89110394](http://www.ncbi.nlm.nih.gov/entrez/query.fcgi?cmd=Search&db=Protein&term=89110394&doptcmdl=GenPept) | glycine C-acetyltransferase [Escherichia coli W3110] | 185.94 | 3 | 958 1411 1677 |  |  |
|  | [85676425](http://www.ncbi.nlm.nih.gov/entrez/query.fcgi?cmd=Search&db=Protein&term=85676425&doptcmdl=GenPept) | glycine C-acetyltransferase [Escherichia coli W3110] | 185.94 | 3 | 958 1411 1677 |  |  |
|  | [83588529](http://www.ncbi.nlm.nih.gov/entrez/query.fcgi?cmd=Search&db=Protein&term=83588529&doptcmdl=GenPept) | COG0156: 7-keto-8-aminopelargonate synthetase and related enzymes [Escherichia coli 101-1] | 185.94 | 3 | 958 1411 1677 |  |  |
| 153 | [91214097](http://www.ncbi.nlm.nih.gov/entrez/query.fcgi?cmd=Search&db=Protein&term=91214097&doptcmdl=GenPept) | deoxyribose-phosphate aldolase [Escherichia coli UTI89] | 182.38 | 2 | 74 1719 |  |  |
|  | [91075671](http://www.ncbi.nlm.nih.gov/entrez/query.fcgi?cmd=Search&db=Protein&term=91075671&doptcmdl=GenPept) | deoxyribose-phosphate aldolase [Escherichia coli UTI89] [MASS=28651] | 182.38 | 2 | 74 1719 |  |  |
|  | [89111089](http://www.ncbi.nlm.nih.gov/entrez/query.fcgi?cmd=Search&db=Protein&term=89111089&doptcmdl=GenPept) | 2-deoxyribose-5-phosphate aldolase, NAD(P)-linked [Escherichia coli W3110] | 182.38 | 2 | 74 1719 |  |  |
|  | [85677120](http://www.ncbi.nlm.nih.gov/entrez/query.fcgi?cmd=Search&db=Protein&term=85677120&doptcmdl=GenPept) | 2-deoxyribose-5-phosphate aldolase, NAD(P)-linked [Escherichia coli W3110] | 182.38 | 2 | 74 1719 |  |  |
|  | [83585289](http://www.ncbi.nlm.nih.gov/entrez/query.fcgi?cmd=Search&db=Protein&term=83585289&doptcmdl=GenPept) | COG0274: Deoxyribose-phosphate aldolase [Escherichia coli 101-1] | 182.38 | 2 | 74 1719 |  |  |
| 154 | [97051296](http://www.ncbi.nlm.nih.gov/entrez/query.fcgi?cmd=Search&db=Protein&term=97051296&doptcmdl=GenPept) | Serine hydroxymethyltransferase (Serine methylase) (SHMT) | 178.38 | 4 | 97 175 583 1463 |  |  |
|  | [91211875](http://www.ncbi.nlm.nih.gov/entrez/query.fcgi?cmd=Search&db=Protein&term=91211875&doptcmdl=GenPept) | serine hydroxymethyltransferase [Escherichia coli UTI89] | 178.38 | 4 | 97 175 583 1463 |  |  |
|  | [91073449](http://www.ncbi.nlm.nih.gov/entrez/query.fcgi?cmd=Search&db=Protein&term=91073449&doptcmdl=GenPept) | serine hydroxymethyltransferase [Escherichia coli UTI89] | 178.38 | 4 | 97 175 583 1463 |  |  |
|  | [89109357](http://www.ncbi.nlm.nih.gov/entrez/query.fcgi?cmd=Search&db=Protein&term=89109357&doptcmdl=GenPept) | serine hydroxymethyltransferase [Escherichia coli W3110] | 178.38 | 4 | 97 175 583 1463 |  |  |
|  | [83586799](http://www.ncbi.nlm.nih.gov/entrez/query.fcgi?cmd=Search&db=Protein&term=83586799&doptcmdl=GenPept) | COG0112: Glycine/serine hydroxymethyltransferase [Escherichia coli 101-1] | 178.38 | 4 | 97 175 583 1463 |  |  |
| 155 | [91213705](http://www.ncbi.nlm.nih.gov/entrez/query.fcgi?cmd=Search&db=Protein&term=91213705&doptcmdl=GenPept) | fumarate reductase iron-sulfur subunit [Escherichia coli UTI89] | 177.86 | 4 | 354 852 1107 1160 |  |  |
|  | [91075279](http://www.ncbi.nlm.nih.gov/entrez/query.fcgi?cmd=Search&db=Protein&term=91075279&doptcmdl=GenPept) | iron-sulfur protein subunit of fumarate reductase [Escherichia coli UTI89] [MASS=27107] | 177.86 | 4 | 354 852 1107 1160 |  |  |
|  | [90108811](http://www.ncbi.nlm.nih.gov/entrez/query.fcgi?cmd=Search&db=Protein&term=90108811&doptcmdl=GenPept) | Chain N, E. Coli Quinol Fumarate Reductase Frda E49q Mutation | 177.86 | 4 | 354 852 1107 1160 |  |  |
|  | [90108807](http://www.ncbi.nlm.nih.gov/entrez/query.fcgi?cmd=Search&db=Protein&term=90108807&doptcmdl=GenPept) | Chain B, E. Coli Quinol Fumarate Reductase Frda E49q Mutation | 177.86 | 4 | 354 852 1107 1160 |  |  |
|  | [89110876](http://www.ncbi.nlm.nih.gov/entrez/query.fcgi?cmd=Search&db=Protein&term=89110876&doptcmdl=GenPept) | fumarate reductase (anaerobic), Fe-S subunit [Escherichia coli W3110] | 177.86 | 4 | 354 852 1107 1160 |  |  |
| 156 | [93278614](http://www.ncbi.nlm.nih.gov/entrez/query.fcgi?cmd=Search&db=Protein&term=93278614&doptcmdl=GenPept) | Chain B, 4 Crystal Structures Of Cap-Dna With All Base-Pair Substitutions At Position 6, Cap-[6c;17g]icap38 Dna | 177.41 | 4 | 592 938 1021 1346 |  |  |
|  | [93278613](http://www.ncbi.nlm.nih.gov/entrez/query.fcgi?cmd=Search&db=Protein&term=93278613&doptcmdl=GenPept) | Chain A, 4 Crystal Structures Of Cap-Dna With All Base-Pair Substitutions At Position 6, Cap-[6c;17g]icap38 Dna | 177.41 | 4 | 592 938 1021 1346 |  |  |
|  | [93278608](http://www.ncbi.nlm.nih.gov/entrez/query.fcgi?cmd=Search&db=Protein&term=93278608&doptcmdl=GenPept) | Chain B, 4 Crystal Structures Of Cap-Dna With All Base-Pair Substitutions At Position 6, Cap-[6g;17c]icap38 Dna | 177.41 | 4 | 592 938 1021 1346 |  |  |
|  | [93278607](http://www.ncbi.nlm.nih.gov/entrez/query.fcgi?cmd=Search&db=Protein&term=93278607&doptcmdl=GenPept) | Chain A, 4 Crystal Structures Of Cap-Dna With All Base-Pair Substitutions At Position 6, Cap-[6g;17c]icap38 Dna | 177.41 | 4 | 592 938 1021 1346 |  |  |
|  | [93278602](http://www.ncbi.nlm.nih.gov/entrez/query.fcgi?cmd=Search&db=Protein&term=93278602&doptcmdl=GenPept) | Chain B, 4 Crystal Structures Of Cap-Dna With All Base-Pair Substitutions At Position 6, Cap-[6a;17t]icap38 Dna | 177.41 | 4 | 592 938 1021 1346 |  |  |
| 157 | [89108558](http://www.ncbi.nlm.nih.gov/entrez/query.fcgi?cmd=Search&db=Protein&term=89108558&doptcmdl=GenPept) | protein chain initiation factor IF-3 [Escherichia coli W3110] | 175.90 | 3 | 429 1272 1399 |  |  |
|  | [67466075](http://www.ncbi.nlm.nih.gov/entrez/query.fcgi?cmd=Search&db=Protein&term=67466075&doptcmdl=GenPept) | Translation initiation factor IF-3 | 175.90 | 3 | 429 1272 1399 |  |  |
|  | [67466074](http://www.ncbi.nlm.nih.gov/entrez/query.fcgi?cmd=Search&db=Protein&term=67466074&doptcmdl=GenPept) | Translation initiation factor IF-3 | 175.90 | 3 | 429 1272 1399 |  |  |
|  | [67466073](http://www.ncbi.nlm.nih.gov/entrez/query.fcgi?cmd=Search&db=Protein&term=67466073&doptcmdl=GenPept) | Translation initiation factor IF-3 | 175.90 | 3 | 429 1272 1399 |  |  |
|  | [43067](http://www.ncbi.nlm.nih.gov/entrez/query.fcgi?cmd=Search&db=Protein&term=43067&doptcmdl=GenPept) | unnamed protein product [Escherichia coli] | 175.90 | 3 | 429 1272 1399 |  |  |
| 158 | [91209758](http://www.ncbi.nlm.nih.gov/entrez/query.fcgi?cmd=Search&db=Protein&term=91209758&doptcmdl=GenPept) | succinate dehydrogenase iron-sulfur subunit [Escherichia coli UTI89] | 174.67 | 4 | 373 1059 1311 1628 |  |  |
|  | [91071332](http://www.ncbi.nlm.nih.gov/entrez/query.fcgi?cmd=Search&db=Protein&term=91071332&doptcmdl=GenPept) | succinate dehydrogenase, iron sulfur protein [Escherichia coli UTI89] | 174.67 | 4 | 373 1059 1311 1628 |  |  |
|  | [89107582](http://www.ncbi.nlm.nih.gov/entrez/query.fcgi?cmd=Search&db=Protein&term=89107582&doptcmdl=GenPept) | succinate dehydrogenase, FeS subunit [Escherichia coli W3110] | 174.67 | 4 | 373 1059 1311 1628 |  |  |
|  | [85544085](http://www.ncbi.nlm.nih.gov/entrez/query.fcgi?cmd=Search&db=Protein&term=85544085&doptcmdl=GenPept) | Chain B, Complex Ii (Succinate Dehydrogenase) From E. Coli With Atpenin A5 Inhibitor Co-Crystallized At The Ubiquinone Binding Site | 174.67 | 4 | 373 1059 1311 1628 |  |  |
|  | [83585344](http://www.ncbi.nlm.nih.gov/entrez/query.fcgi?cmd=Search&db=Protein&term=83585344&doptcmdl=GenPept) | COG0479: Succinate dehydrogenase/fumarate reductase, Fe-S protein subunit [Escherichia coli 101-1] | 174.67 | 4 | 373 1059 1311 1628 |  |  |
| 159 | [91213692](http://www.ncbi.nlm.nih.gov/entrez/query.fcgi?cmd=Search&db=Protein&term=91213692&doptcmdl=GenPept) | co-chaperonin GroES [Escherichia coli UTI89] | 174.53 | 3 | 509 659 1293 |  |  |
|  | [91075266](http://www.ncbi.nlm.nih.gov/entrez/query.fcgi?cmd=Search&db=Protein&term=91075266&doptcmdl=GenPept) | GroES (10 Kd chaperone protein) [Escherichia coli UTI89] | 174.53 | 3 | 509 659 1293 |  |  |
|  | [89110863](http://www.ncbi.nlm.nih.gov/entrez/query.fcgi?cmd=Search&db=Protein&term=89110863&doptcmdl=GenPept) | Cpn10 chaperonin GroES, small subunit of GroESL [Escherichia coli W3110] | 174.53 | 3 | 509 659 1293 |  |  |
|  | [88192627](http://www.ncbi.nlm.nih.gov/entrez/query.fcgi?cmd=Search&db=Protein&term=88192627&doptcmdl=GenPept) | Chain U, Fitted Coordinates For Groel-Adp7-Groes Cryo-Em Complex (Emd-1181) | 174.53 | 3 | 509 659 1293 |  |  |
|  | [88192626](http://www.ncbi.nlm.nih.gov/entrez/query.fcgi?cmd=Search&db=Protein&term=88192626&doptcmdl=GenPept) | Chain T, Fitted Coordinates For Groel-Adp7-Groes Cryo-Em Complex (Emd-1181) | 174.53 | 3 | 509 659 1293 |  |  |
| 160 | [9955254](http://www.ncbi.nlm.nih.gov/entrez/query.fcgi?cmd=Search&db=Protein&term=9955254&doptcmdl=GenPept) | Chain B, A Minor Fmn-Dependent Nitroreductase From Escherichia Coli B [MASS=23887] | 172.64 | 3 | 332 395 1511 |  |  |
|  | [9955253](http://www.ncbi.nlm.nih.gov/entrez/query.fcgi?cmd=Search&db=Protein&term=9955253&doptcmdl=GenPept) | Chain A, A Minor Fmn-Dependent Nitroreductase From Escherichia Coli B | 172.64 | 3 | 332 395 1511 |  |  |
|  | [91209623](http://www.ncbi.nlm.nih.gov/entrez/query.fcgi?cmd=Search&db=Protein&term=91209623&doptcmdl=GenPept) | dihydropteridine reductase [Escherichia coli UTI89] | 172.64 | 3 | 332 395 1511 |  |  |
|  | [91071197](http://www.ncbi.nlm.nih.gov/entrez/query.fcgi?cmd=Search&db=Protein&term=91071197&doptcmdl=GenPept) | oxygen-insensitive NAD(P)H nitroreductase [Escherichia coli UTI89] | 172.64 | 3 | 332 395 1511 |  |  |
|  | [89107443](http://www.ncbi.nlm.nih.gov/entrez/query.fcgi?cmd=Search&db=Protein&term=89107443&doptcmdl=GenPept) | dihydropteridine reductase, NAD(P)H-dependent, oxygen-insensitive [Escherichia coli W3110] | 172.64 | 3 | 332 395 1511 |  |  |
| 161 | [91212480](http://www.ncbi.nlm.nih.gov/entrez/query.fcgi?cmd=Search&db=Protein&term=91212480&doptcmdl=GenPept) | bifunctional heptose 7-phosphate kinase/heptose 1-phosphate adenyltransferase [Escherichia coli UTI89] | 168.78 | 3 | 987 1680 1695 |  |  |
|  | [91074054](http://www.ncbi.nlm.nih.gov/entrez/query.fcgi?cmd=Search&db=Protein&term=91074054&doptcmdl=GenPept) | ADP-heptose synthase [Escherichia coli UTI89] | 168.78 | 3 | 987 1680 1695 |  |  |
|  | [89109822](http://www.ncbi.nlm.nih.gov/entrez/query.fcgi?cmd=Search&db=Protein&term=89109822&doptcmdl=GenPept) | fused heptose 7-phosphate kinase and heptose 1-phosphate adenyltransferase [Escherichia coli W3110] | 168.78 | 3 | 987 1680 1695 |  |  |
|  | [85675853](http://www.ncbi.nlm.nih.gov/entrez/query.fcgi?cmd=Search&db=Protein&term=85675853&doptcmdl=GenPept) | fused heptose 7-phosphate kinase and heptose 1-phosphate adenyltransferase [Escherichia coli W3110] | 168.78 | 3 | 987 1680 1695 |  |  |
|  | [83587510](http://www.ncbi.nlm.nih.gov/entrez/query.fcgi?cmd=Search&db=Protein&term=83587510&doptcmdl=GenPept) | COG2870: ADP-heptose synthase, bifunctional sugar kinase/adenylyltransferase [Escherichia coli 101-1] | 168.78 | 3 | 987 1680 1695 |  |  |
| 162 | [91209536](http://www.ncbi.nlm.nih.gov/entrez/query.fcgi?cmd=Search&db=Protein&term=91209536&doptcmdl=GenPept) | acriflavine resistance protein A precursor [Escherichia coli UTI89] | 167.17 | 3 | 282 283 1038 |  |  |
|  | [91071110](http://www.ncbi.nlm.nih.gov/entrez/query.fcgi?cmd=Search&db=Protein&term=91071110&doptcmdl=GenPept) | acriflavine resistance protein A precursor [Escherichia coli UTI89] | 167.17 | 3 | 282 283 1038 |  |  |
|  | [89107332](http://www.ncbi.nlm.nih.gov/entrez/query.fcgi?cmd=Search&db=Protein&term=89107332&doptcmdl=GenPept) | multidrug efflux system [Escherichia coli W3110] | 167.17 | 3 | 282 283 1038 |  |  |
|  | [85674602](http://www.ncbi.nlm.nih.gov/entrez/query.fcgi?cmd=Search&db=Protein&term=85674602&doptcmdl=GenPept) | multidrug efflux system [Escherichia coli W3110] | 167.17 | 3 | 282 283 1038 |  |  |
|  | [83587082](http://www.ncbi.nlm.nih.gov/entrez/query.fcgi?cmd=Search&db=Protein&term=83587082&doptcmdl=GenPept) | COG0845: Membrane-fusion protein [Escherichia coli 101-1] | 167.17 | 3 | 282 283 1038 |  |  |
| 163 | [91213959](http://www.ncbi.nlm.nih.gov/entrez/query.fcgi?cmd=Search&db=Protein&term=91213959&doptcmdl=GenPept) | type 1 fimbriae major subunit FimA [Escherichia coli UTI89] | 167.02 | 2 | 838 1181 |  |  |
|  | [91075533](http://www.ncbi.nlm.nih.gov/entrez/query.fcgi?cmd=Search&db=Protein&term=91075533&doptcmdl=GenPept) | type 1 fimbriae major subunit FimA [Escherichia coli UTI89] [MASS=18553] | 167.02 | 2 | 838 1181 |  |  |
|  | [89111026](http://www.ncbi.nlm.nih.gov/entrez/query.fcgi?cmd=Search&db=Protein&term=89111026&doptcmdl=GenPept) | major type 1 subunit fimbrin [Escherichia coli W3110] | 167.02 | 2 | 838 1181 |  |  |
|  | [85677057](http://www.ncbi.nlm.nih.gov/entrez/query.fcgi?cmd=Search&db=Protein&term=85677057&doptcmdl=GenPept) | major type 1 subunit fimbrin [Escherichia coli W3110] [MASS=18111] | 167.02 | 2 | 838 1181 |  |  |
|  | [732683](http://www.ncbi.nlm.nih.gov/entrez/query.fcgi?cmd=Search&db=Protein&term=732683&doptcmdl=GenPept) | FimA precursor [Escherichia coli] | 167.02 | 2 | 838 1181 |  |  |
| 164 | [91211575](http://www.ncbi.nlm.nih.gov/entrez/query.fcgi?cmd=Search&db=Protein&term=91211575&doptcmdl=GenPept) | NADH dehydrogenase subunit G [Escherichia coli UTI89] | 166.57 | 3 | 1299 1481 1484 |  |  |
|  | [91073149](http://www.ncbi.nlm.nih.gov/entrez/query.fcgi?cmd=Search&db=Protein&term=91073149&doptcmdl=GenPept) | NADH dehydrogenase I chain G [Escherichia coli UTI89] | 166.57 | 3 | 1299 1481 1484 |  |  |
|  | [89109101](http://www.ncbi.nlm.nih.gov/entrez/query.fcgi?cmd=Search&db=Protein&term=89109101&doptcmdl=GenPept) | NADH:ubiquinone oxidoreductase, chain G [Escherichia coli W3110] | 166.57 | 3 | 1299 1481 1484 |  |  |
|  | [85675343](http://www.ncbi.nlm.nih.gov/entrez/query.fcgi?cmd=Search&db=Protein&term=85675343&doptcmdl=GenPept) | NADH:ubiquinone oxidoreductase, chain G [Escherichia coli W3110] | 166.57 | 3 | 1299 1481 1484 |  |  |
|  | [83588266](http://www.ncbi.nlm.nih.gov/entrez/query.fcgi?cmd=Search&db=Protein&term=83588266&doptcmdl=GenPept) | COG1034: NADH dehydrogenase/NADH:ubiquinone oxidoreductase 75 kD subunit (chain G) [Escherichia coli 101-1] | 166.57 | 3 | 1299 1481 1484 |  |  |
| 165 | [91213258](http://www.ncbi.nlm.nih.gov/entrez/query.fcgi?cmd=Search&db=Protein&term=91213258&doptcmdl=GenPept) | F0F1 ATP synthase subunit gamma [Escherichia coli UTI89] | 166.34 | 3 | 769 1373 1433 |  |  |
|  | [91074832](http://www.ncbi.nlm.nih.gov/entrez/query.fcgi?cmd=Search&db=Protein&term=91074832&doptcmdl=GenPept) | membrane-bound ATP synthase F1 sector gamma-subunit [Escherichia coli UTI89] | 166.34 | 3 | 769 1373 1433 |  |  |
|  | [89110274](http://www.ncbi.nlm.nih.gov/entrez/query.fcgi?cmd=Search&db=Protein&term=89110274&doptcmdl=GenPept) | F1 sector of membrane-bound ATP synthase, gamma subunit [Escherichia coli W3110] | 166.34 | 3 | 769 1373 1433 |  |  |
|  | [85676305](http://www.ncbi.nlm.nih.gov/entrez/query.fcgi?cmd=Search&db=Protein&term=85676305&doptcmdl=GenPept) | F1 sector of membrane-bound ATP synthase, gamma subunit [Escherichia coli W3110] | 166.34 | 3 | 769 1373 1433 |  |  |
|  | [83588402](http://www.ncbi.nlm.nih.gov/entrez/query.fcgi?cmd=Search&db=Protein&term=83588402&doptcmdl=GenPept) | COG0224: F0F1-type ATP synthase, gamma subunit [Escherichia coli 101-1] | 166.34 | 3 | 769 1373 1433 |  |  |
| 166 | [91211890](http://www.ncbi.nlm.nih.gov/entrez/query.fcgi?cmd=Search&db=Protein&term=91211890&doptcmdl=GenPept) | pyridoxal phosphate biosynthetic protein PdxJ [Escherichia coli UTI89] | 165.07 | 2 | 363 593 |  |  |
|  | [91073464](http://www.ncbi.nlm.nih.gov/entrez/query.fcgi?cmd=Search&db=Protein&term=91073464&doptcmdl=GenPept) | pyridoxal phosphate biosynthetic protein PdxJ [Escherichia coli UTI89] | 165.07 | 2 | 363 593 |  |  |
|  | [89109370](http://www.ncbi.nlm.nih.gov/entrez/query.fcgi?cmd=Search&db=Protein&term=89109370&doptcmdl=GenPept) | pyridoxine 5'-phosphate synthase [Escherichia coli W3110] | 165.07 | 2 | 363 593 |  |  |
|  | [85675455](http://www.ncbi.nlm.nih.gov/entrez/query.fcgi?cmd=Search&db=Protein&term=85675455&doptcmdl=GenPept) | pyridoxine 5'-phosphate synthase [Escherichia coli W3110] | 165.07 | 2 | 363 593 |  |  |
|  | [83586784](http://www.ncbi.nlm.nih.gov/entrez/query.fcgi?cmd=Search&db=Protein&term=83586784&doptcmdl=GenPept) | COG0854: Pyridoxal phosphate biosynthesis protein [Escherichia coli 101-1] [MASS=27015] | 165.07 | 2 | 363 593 |  |  |
| 167 | [91212277](http://www.ncbi.nlm.nih.gov/entrez/query.fcgi?cmd=Search&db=Protein&term=91212277&doptcmdl=GenPept) | putative global regulator [Escherichia coli UTI89] | 163.55 | 3 | 836 1133 1345 |  |  |
|  | [91073851](http://www.ncbi.nlm.nih.gov/entrez/query.fcgi?cmd=Search&db=Protein&term=91073851&doptcmdl=GenPept) | 2D-phage unknown protein [Escherichia coli UTI89] | 163.55 | 3 | 836 1133 1345 |  |  |
|  | [89109677](http://www.ncbi.nlm.nih.gov/entrez/query.fcgi?cmd=Search&db=Protein&term=89109677&doptcmdl=GenPept) | predicted folate-dependent regulatory protein [Escherichia coli W3110] | 163.55 | 3 | 836 1133 1345 |  |  |
|  | [887848](http://www.ncbi.nlm.nih.gov/entrez/query.fcgi?cmd=Search&db=Protein&term=887848&doptcmdl=GenPept) | ORF_o326 | 163.55 | 3 | 836 1133 1345 |  |  |
|  | [85675710](http://www.ncbi.nlm.nih.gov/entrez/query.fcgi?cmd=Search&db=Protein&term=85675710&doptcmdl=GenPept) | predicted folate-dependent regulatory protein [Escherichia coli W3110] | 163.55 | 3 | 836 1133 1345 |  |  |
| 168 | [91075674](http://www.ncbi.nlm.nih.gov/entrez/query.fcgi?cmd=Search&db=Protein&term=91075674&doptcmdl=GenPept) | purine nucleoside phosphorylase [Escherichia coli UTI89] | 162.50 | 3 | 433 1234 1409 |  |  |
|  | [89111092](http://www.ncbi.nlm.nih.gov/entrez/query.fcgi?cmd=Search&db=Protein&term=89111092&doptcmdl=GenPept) | purine-nucleoside phosphorylase [Escherichia coli W3110] | 162.50 | 3 | 433 1234 1409 |  |  |
|  | [85677123](http://www.ncbi.nlm.nih.gov/entrez/query.fcgi?cmd=Search&db=Protein&term=85677123&doptcmdl=GenPept) | purine-nucleoside phosphorylase [Escherichia coli W3110] | 162.50 | 3 | 433 1234 1409 |  |  |
|  | [85541683](http://www.ncbi.nlm.nih.gov/entrez/query.fcgi?cmd=Search&db=Protein&term=85541683&doptcmdl=GenPept) | Purine nucleoside phosphorylase deoD-type (PNP) | 162.50 | 3 | 433 1234 1409 |  |  |
|  | [85541681](http://www.ncbi.nlm.nih.gov/entrez/query.fcgi?cmd=Search&db=Protein&term=85541681&doptcmdl=GenPept) | Purine nucleoside phosphorylase deoD-type (PNP) | 162.50 | 3 | 433 1234 1409 |  |  |

| **Protein IDs*, cont.*** | | | | | | | |
| --- | --- | --- | --- | --- | --- | --- | --- |
| *Grp Nr.* | *Accession Number* | *Protein Name* | *Protein Score* | *Unique PSMs* | *PSM Serial Nrs.* | *Other Grp.* | *Score (other)* |
| 169 | [91209248](http://www.ncbi.nlm.nih.gov/entrez/query.fcgi?cmd=Search&db=Protein&term=91209248&doptcmdl=GenPept) | periplasmic chaperone [Escherichia coli UTI89] | 161.14 | 3 | 260 824 872 |  |  |
|  | [91070822](http://www.ncbi.nlm.nih.gov/entrez/query.fcgi?cmd=Search&db=Protein&term=91070822&doptcmdl=GenPept) | histone-like protein, located in outer membrane or nucleoid [Escherichia coli UTI89] | 161.14 | 3 | 260 824 872 |  |  |
|  | [90101755](http://www.ncbi.nlm.nih.gov/entrez/query.fcgi?cmd=Search&db=Protein&term=90101755&doptcmdl=GenPept) | Chaperone protein skp precursor | 161.14 | 3 | 260 824 872 |  |  |
|  | [90101754](http://www.ncbi.nlm.nih.gov/entrez/query.fcgi?cmd=Search&db=Protein&term=90101754&doptcmdl=GenPept) | Chaperone protein skp precursor | 161.14 | 3 | 260 824 872 |  |  |
|  | [90101753](http://www.ncbi.nlm.nih.gov/entrez/query.fcgi?cmd=Search&db=Protein&term=90101753&doptcmdl=GenPept) | Chaperone protein skp precursor | 161.14 | 3 | 260 824 872 |  |  |
| 170 | [91213067](http://www.ncbi.nlm.nih.gov/entrez/query.fcgi?cmd=Search&db=Protein&term=91213067&doptcmdl=GenPept) | hypothetical protein UTI89_C4094 [Escherichia coli UTI89] | 160.68 | 4 | 508 722 1065 1499 |  |  |
|  | [91074641](http://www.ncbi.nlm.nih.gov/entrez/query.fcgi?cmd=Search&db=Protein&term=91074641&doptcmdl=GenPept) | hypothetical protein UTI89_C4094 [Escherichia coli UTI89] | 160.68 | 4 | 508 722 1065 1499 |  |  |
|  | [90111615](http://www.ncbi.nlm.nih.gov/entrez/query.fcgi?cmd=Search&db=Protein&term=90111615&doptcmdl=GenPept) | conserved protein [Escherichia coli str. K-12 substr. MG1655] | 160.68 | 4 | 508 722 1065 1499 |  |  |
|  | [89110460](http://www.ncbi.nlm.nih.gov/entrez/query.fcgi?cmd=Search&db=Protein&term=89110460&doptcmdl=GenPept) | hypothetical protein [Escherichia coli W3110] | 160.68 | 4 | 508 722 1065 1499 |  |  |
|  | [87082290](http://www.ncbi.nlm.nih.gov/entrez/query.fcgi?cmd=Search&db=Protein&term=87082290&doptcmdl=GenPept) | conserved protein [Escherichia coli str. K-12 substr. MG1655] | 160.68 | 4 | 508 722 1065 1499 |  |  |
| 171 | [91209251](http://www.ncbi.nlm.nih.gov/entrez/query.fcgi?cmd=Search&db=Protein&term=91209251&doptcmdl=GenPept) | UDP-N-acetylglucosamine acyltransferase [Escherichia coli UTI89] | 160.05 | 3 | 1343 1396 1544 |  |  |
|  | [91070825](http://www.ncbi.nlm.nih.gov/entrez/query.fcgi?cmd=Search&db=Protein&term=91070825&doptcmdl=GenPept) | UDP-N-acetylglucosamine acetyltransferase [Escherichia coli UTI89] | 160.05 | 3 | 1343 1396 1544 |  |  |
|  | [89107061](http://www.ncbi.nlm.nih.gov/entrez/query.fcgi?cmd=Search&db=Protein&term=89107061&doptcmdl=GenPept) | UDP-N-acetylglucosamine acetyltransferase [Escherichia coli W3110] | 160.05 | 3 | 1343 1396 1544 |  |  |
|  | [85674370](http://www.ncbi.nlm.nih.gov/entrez/query.fcgi?cmd=Search&db=Protein&term=85674370&doptcmdl=GenPept) | UDP-N-acetylglucosamine acetyltransferase [Escherichia coli W3110] | 160.05 | 3 | 1343 1396 1544 |  |  |
|  | [83584718](http://www.ncbi.nlm.nih.gov/entrez/query.fcgi?cmd=Search&db=Protein&term=83584718&doptcmdl=GenPept) | COG1043: Acyl-[acyl carrier protein]--UDP-N-acetylglucosamine O-acyltransferase [Escherichia coli 101-1] | 160.05 | 3 | 1343 1396 1544 |  |  |
| 172 | [91213572](http://www.ncbi.nlm.nih.gov/entrez/query.fcgi?cmd=Search&db=Protein&term=91213572&doptcmdl=GenPept) | quinone oxidoreductase, NADPH-dependent [Escherichia coli UTI89] | 157.64 | 3 | 149 152 1618 |  |  |
|  | [91075146](http://www.ncbi.nlm.nih.gov/entrez/query.fcgi?cmd=Search&db=Protein&term=91075146&doptcmdl=GenPept) | Qor subunit of quinone oxidoreductase [Escherichia coli UTI89] | 157.64 | 3 | 149 152 1618 |  |  |
|  | [89110772](http://www.ncbi.nlm.nih.gov/entrez/query.fcgi?cmd=Search&db=Protein&term=89110772&doptcmdl=GenPept) | quinone oxidoreductase, NADPH-dependent [Escherichia coli W3110] | 157.64 | 3 | 149 152 1618 |  |  |
|  | [85676803](http://www.ncbi.nlm.nih.gov/entrez/query.fcgi?cmd=Search&db=Protein&term=85676803&doptcmdl=GenPept) | quinone oxidoreductase, NADPH-dependent [Escherichia coli W3110] | 157.64 | 3 | 149 152 1618 |  |  |
|  | [83587174](http://www.ncbi.nlm.nih.gov/entrez/query.fcgi?cmd=Search&db=Protein&term=83587174&doptcmdl=GenPept) | COG0604: NADPH:quinone reductase and related Zn-dependent oxidoreductases [Escherichia coli 101-1] | 157.64 | 3 | 149 152 1618 |  |  |
| 173 | [91212877](http://www.ncbi.nlm.nih.gov/entrez/query.fcgi?cmd=Search&db=Protein&term=91212877&doptcmdl=GenPept) | phosphoenolpyruvate carboxykinase [Escherichia coli UTI89] | 156.05 | 5 | 18 590 1193 1423 1718 |  |  |
|  | [91074451](http://www.ncbi.nlm.nih.gov/entrez/query.fcgi?cmd=Search&db=Protein&term=91074451&doptcmdl=GenPept) | ATP phosphoenolpyruvate carboxykinase [Escherichia coli UTI89] [MASS=62417] | 156.05 | 5 | 18 590 1193 1423 1718 |  |  |
|  | [89110607](http://www.ncbi.nlm.nih.gov/entrez/query.fcgi?cmd=Search&db=Protein&term=89110607&doptcmdl=GenPept) | phosphoenolpyruvate carboxykinase [Escherichia coli W3110] | 156.05 | 5 | 18 590 1193 1423 1718 |  |  |
|  | [85676638](http://www.ncbi.nlm.nih.gov/entrez/query.fcgi?cmd=Search&db=Protein&term=85676638&doptcmdl=GenPept) | phosphoenolpyruvate carboxykinase [Escherichia coli W3110] | 156.05 | 5 | 18 590 1193 1423 1718 |  |  |
|  | [83588732](http://www.ncbi.nlm.nih.gov/entrez/query.fcgi?cmd=Search&db=Protein&term=83588732&doptcmdl=GenPept) | COG1866: Phosphoenolpyruvate carboxykinase (ATP) [Escherichia coli 101-1] | 156.05 | 5 | 18 590 1193 1423 1718 |  |  |
| 174 | [91210245](http://www.ncbi.nlm.nih.gov/entrez/query.fcgi?cmd=Search&db=Protein&term=91210245&doptcmdl=GenPept) | 3-ketoacyl-(acyl-carrier-protein) reductase [Escherichia coli UTI89] | 155.56 | 3 | 474 709 1487 |  |  |
|  | [91071819](http://www.ncbi.nlm.nih.gov/entrez/query.fcgi?cmd=Search&db=Protein&term=91071819&doptcmdl=GenPept) | 3-oxoacyl-[acyl-carrier-protein] reductase [Escherichia coli UTI89] | 155.56 | 3 | 474 709 1487 |  |  |
|  | [89107939](http://www.ncbi.nlm.nih.gov/entrez/query.fcgi?cmd=Search&db=Protein&term=89107939&doptcmdl=GenPept) | 3-oxoacyl-[acyl-carrier-protein] reductase [Escherichia coli W3110] | 155.56 | 3 | 474 709 1487 |  |  |
|  | [84028082](http://www.ncbi.nlm.nih.gov/entrez/query.fcgi?cmd=Search&db=Protein&term=84028082&doptcmdl=GenPept) | 3-oxoacyl-[acyl-carrier-protein] reductase (3-ketoacyl-acyl carrier protein reductase) | 155.56 | 3 | 474 709 1487 |  |  |
|  | [84028081](http://www.ncbi.nlm.nih.gov/entrez/query.fcgi?cmd=Search&db=Protein&term=84028081&doptcmdl=GenPept) | 3-oxoacyl-[acyl-carrier-protein] reductase (3-ketoacyl-acyl carrier protein reductase) | 155.56 | 3 | 474 709 1487 |  |  |
| 175 | [91212758](http://www.ncbi.nlm.nih.gov/entrez/query.fcgi?cmd=Search&db=Protein&term=91212758&doptcmdl=GenPept) | 30S ribosomal protein S10 [Escherichia coli UTI89] | 151.76 | 4 | 254 700 762 1037 |  |  |
|  | [91074332](http://www.ncbi.nlm.nih.gov/entrez/query.fcgi?cmd=Search&db=Protein&term=91074332&doptcmdl=GenPept) | 30S ribosomal subunit protein S10 [Escherichia coli UTI89] | 151.76 | 4 | 254 700 762 1037 |  |  |
|  | [89110689](http://www.ncbi.nlm.nih.gov/entrez/query.fcgi?cmd=Search&db=Protein&term=89110689&doptcmdl=GenPept) | 30S ribosomal subunit protein S10 [Escherichia coli W3110] | 151.76 | 4 | 254 700 762 1037 |  |  |
|  | [85676720](http://www.ncbi.nlm.nih.gov/entrez/query.fcgi?cmd=Search&db=Protein&term=85676720&doptcmdl=GenPept) | 30S ribosomal subunit protein S10 [Escherichia coli W3110] | 151.76 | 4 | 254 700 762 1037 |  |  |
|  | [83754104](http://www.ncbi.nlm.nih.gov/entrez/query.fcgi?cmd=Search&db=Protein&term=83754104&doptcmdl=GenPept) | Chain J, Crystal Structure Of The Bacterial Ribosome From Escherichia Coli At 3.5 A Resolution. This File Contains The 30s Subunit Of The Second 70s Ribosome. The Entire Crystal Structure Contains Two 70s Ribosomes And Is Described In Remark 400. | 151.76 | 4 | 254 700 762 1037 |  |  |
| 176 | [91212058](http://www.ncbi.nlm.nih.gov/entrez/query.fcgi?cmd=Search&db=Protein&term=91212058&doptcmdl=GenPept) | recombinase A [Escherichia coli UTI89] | 151.07 | 3 | 4 658 1392 |  |  |
|  | [91073632](http://www.ncbi.nlm.nih.gov/entrez/query.fcgi?cmd=Search&db=Protein&term=91073632&doptcmdl=GenPept) | RecA protein [Escherichia coli UTI89] | 151.07 | 3 | 4 658 1392 |  |  |
|  | [89109486](http://www.ncbi.nlm.nih.gov/entrez/query.fcgi?cmd=Search&db=Protein&term=89109486&doptcmdl=GenPept) | DNA strand exchange and recombination protein with protease and nuclease activity [Escherichia coli W3110] | 151.07 | 3 | 4 658 1392 |  |  |
|  | [85675524](http://www.ncbi.nlm.nih.gov/entrez/query.fcgi?cmd=Search&db=Protein&term=85675524&doptcmdl=GenPept) | DNA strand exchange and recombination protein with protease and nuclease activity [Escherichia coli W3110] | 151.07 | 3 | 4 658 1392 |  |  |
|  | [83585642](http://www.ncbi.nlm.nih.gov/entrez/query.fcgi?cmd=Search&db=Protein&term=83585642&doptcmdl=GenPept) | COG0468: RecA/RadA recombinase [Escherichia coli 101-1] | 151.07 | 3 | 4 658 1392 |  |  |
| 177 | [91212146](http://www.ncbi.nlm.nih.gov/entrez/query.fcgi?cmd=Search&db=Protein&term=91212146&doptcmdl=GenPept) | CTP synthetase [Escherichia coli UTI89] | 149.91 | 5 | 422 614 787 1093 1577 |  |  |
|  | [91073720](http://www.ncbi.nlm.nih.gov/entrez/query.fcgi?cmd=Search&db=Protein&term=91073720&doptcmdl=GenPept) | CTP synthetase [Escherichia coli UTI89] | 149.91 | 5 | 422 614 787 1093 1577 |  |  |
|  | [89109566](http://www.ncbi.nlm.nih.gov/entrez/query.fcgi?cmd=Search&db=Protein&term=89109566&doptcmdl=GenPept) | CTP synthetase [Escherichia coli W3110] | 149.91 | 5 | 422 614 787 1093 1577 |  |  |
|  | [882674](http://www.ncbi.nlm.nih.gov/entrez/query.fcgi?cmd=Search&db=Protein&term=882674&doptcmdl=GenPept) | CTP synthetase [Escherichia coli] | 149.91 | 5 | 422 614 787 1093 1577 |  |  |
|  | [85675599](http://www.ncbi.nlm.nih.gov/entrez/query.fcgi?cmd=Search&db=Protein&term=85675599&doptcmdl=GenPept) | CTP synthetase [Escherichia coli W3110] | 149.91 | 5 | 422 614 787 1093 1577 |  |  |
| 178 | [91210052](http://www.ncbi.nlm.nih.gov/entrez/query.fcgi?cmd=Search&db=Protein&term=91210052&doptcmdl=GenPept) | outer membrane protein A [Escherichia coli UTI89] | 148.21 | 4 | 881 1251 1400 1634 |  |  |
|  | [91071626](http://www.ncbi.nlm.nih.gov/entrez/query.fcgi?cmd=Search&db=Protein&term=91071626&doptcmdl=GenPept) | outer membrane protein A precursor [Escherichia coli UTI89] [MASS=40720] | 148.21 | 4 | 881 1251 1400 1634 |  |  |
|  | [89107807](http://www.ncbi.nlm.nih.gov/entrez/query.fcgi?cmd=Search&db=Protein&term=89107807&doptcmdl=GenPept) | outer membrane protein A (3a;II*;G;d) [Escherichia coli W3110] | 148.21 | 4 | 881 1251 1400 1634 |  |  |
|  | [74311514](http://www.ncbi.nlm.nih.gov/entrez/query.fcgi?cmd=Search&db=Protein&term=74311514&doptcmdl=GenPept) | outer membrane protein A [Shigella sonnei Ss046] | 148.21 | 4 | 881 1251 1400 1634 |  |  |
|  | [73854991](http://www.ncbi.nlm.nih.gov/entrez/query.fcgi?cmd=Search&db=Protein&term=73854991&doptcmdl=GenPept) | outer membrane protein 3a (II*;G;d) [Shigella sonnei Ss046] | 148.21 | 4 | 881 1251 1400 1634 |  |  |
| 179 | [93115448](http://www.ncbi.nlm.nih.gov/entrez/query.fcgi?cmd=Search&db=Protein&term=93115448&doptcmdl=GenPept) | glucose-1-phosphate thymidylyltransferase [Escherichia coli] [MASS=32444] | 148.11 | 2 | 1218 1574 |  |  |
|  | [89108859](http://www.ncbi.nlm.nih.gov/entrez/query.fcgi?cmd=Search&db=Protein&term=89108859&doptcmdl=GenPept) | glucose-1-phosphate thymidylyltransferase [Escherichia coli W3110] | 148.11 | 2 | 1218 1574 |  |  |
|  | [82543417](http://www.ncbi.nlm.nih.gov/entrez/query.fcgi?cmd=Search&db=Protein&term=82543417&doptcmdl=GenPept) | glucose-1-phosphate thymidylyltransferase [Shigella boydii Sb227] | 148.11 | 2 | 1218 1574 |  |  |
|  | [81244828](http://www.ncbi.nlm.nih.gov/entrez/query.fcgi?cmd=Search&db=Protein&term=81244828&doptcmdl=GenPept) | glucose-1-phosphate thymidylyltransferase [Shigella boydii Sb227] | 148.11 | 2 | 1218 1574 |  |  |
|  | [799234](http://www.ncbi.nlm.nih.gov/entrez/query.fcgi?cmd=Search&db=Protein&term=799234&doptcmdl=GenPept) | RmlA [Escherichia coli] [MASS=32609] | 148.11 | 2 | 1218 1574 |  |  |
| 180 | [91209115](http://www.ncbi.nlm.nih.gov/entrez/query.fcgi?cmd=Search&db=Protein&term=91209115&doptcmdl=GenPept) | peptidyl-prolyl cis-trans isomerase SurA [Escherichia coli UTI89] | 147.97 | 2 | 948 1613 |  |  |
|  | [91070689](http://www.ncbi.nlm.nih.gov/entrez/query.fcgi?cmd=Search&db=Protein&term=91070689&doptcmdl=GenPept) | peptidyl-prolyl cis-trans isomerase [Escherichia coli UTI89] | 147.97 | 2 | 948 1613 |  |  |
|  | [89106937](http://www.ncbi.nlm.nih.gov/entrez/query.fcgi?cmd=Search&db=Protein&term=89106937&doptcmdl=GenPept) | peptidyl-prolyl cis-trans isomerase [Escherichia coli W3110] | 147.97 | 2 | 948 1613 |  |  |
|  | [85674304](http://www.ncbi.nlm.nih.gov/entrez/query.fcgi?cmd=Search&db=Protein&term=85674304&doptcmdl=GenPept) | peptidyl-prolyl cis-trans isomerase [Escherichia coli W3110] | 147.97 | 2 | 948 1613 |  |  |
|  | [83584902](http://www.ncbi.nlm.nih.gov/entrez/query.fcgi?cmd=Search&db=Protein&term=83584902&doptcmdl=GenPept) | COG0760: Parvulin-like peptidyl-prolyl isomerase [Escherichia coli 101-1] | 147.97 | 2 | 948 1613 |  |  |
| 181 | [91212856](http://www.ncbi.nlm.nih.gov/entrez/query.fcgi?cmd=Search&db=Protein&term=91212856&doptcmdl=GenPept) | tryptophanyl-tRNA synthetase [Escherichia coli UTI89] | 147.09 | 3 | 198 795 1149 |  |  |
|  | [91074430](http://www.ncbi.nlm.nih.gov/entrez/query.fcgi?cmd=Search&db=Protein&term=91074430&doptcmdl=GenPept) | tryptophan tRNA synthetase [Escherichia coli UTI89] | 147.09 | 3 | 198 795 1149 |  |  |
|  | [89110626](http://www.ncbi.nlm.nih.gov/entrez/query.fcgi?cmd=Search&db=Protein&term=89110626&doptcmdl=GenPept) | tryptophanyl-tRNA synthetase [Escherichia coli W3110] | 147.09 | 3 | 198 795 1149 |  |  |
|  | [85676657](http://www.ncbi.nlm.nih.gov/entrez/query.fcgi?cmd=Search&db=Protein&term=85676657&doptcmdl=GenPept) | tryptophanyl-tRNA synthetase [Escherichia coli W3110] | 147.09 | 3 | 198 795 1149 |  |  |
|  | [83585105](http://www.ncbi.nlm.nih.gov/entrez/query.fcgi?cmd=Search&db=Protein&term=83585105&doptcmdl=GenPept) | COG0180: Tryptophanyl-tRNA synthetase [Escherichia coli 101-1] | 147.09 | 3 | 198 795 1149 |  |  |
| 182 | [9664552](http://www.ncbi.nlm.nih.gov/entrez/query.fcgi?cmd=Search&db=Protein&term=9664552&doptcmdl=GenPept) | aerobic regulator [Escherichia coli] [MASS=21580] | 145.53 | 3 | 619 1269 1540 |  |  |
|  | [9664550](http://www.ncbi.nlm.nih.gov/entrez/query.fcgi?cmd=Search&db=Protein&term=9664550&doptcmdl=GenPept) | aerobic regulator [Escherichia coli] | 145.53 | 3 | 619 1269 1540 |  |  |
|  | [9664548](http://www.ncbi.nlm.nih.gov/entrez/query.fcgi?cmd=Search&db=Protein&term=9664548&doptcmdl=GenPept) | aerobic regulator [Escherichia coli] | 145.53 | 3 | 619 1269 1540 |  |  |
|  | [9664546](http://www.ncbi.nlm.nih.gov/entrez/query.fcgi?cmd=Search&db=Protein&term=9664546&doptcmdl=GenPept) | aerobic regulator [Escherichia coli] | 145.53 | 3 | 619 1269 1540 |  |  |
|  | [9664544](http://www.ncbi.nlm.nih.gov/entrez/query.fcgi?cmd=Search&db=Protein&term=9664544&doptcmdl=GenPept) | aerobic regulator [Escherichia coli] [MASS=21606] | 145.53 | 3 | 619 1269 1540 |  |  |
| 183 | [91212647](http://www.ncbi.nlm.nih.gov/entrez/query.fcgi?cmd=Search&db=Protein&term=91212647&doptcmdl=GenPept) | stringent starvation protein A [Escherichia coli UTI89] | 141.74 | 2 | 1243 1476 |  |  |
|  | [91074221](http://www.ncbi.nlm.nih.gov/entrez/query.fcgi?cmd=Search&db=Protein&term=91074221&doptcmdl=GenPept) | stringent starvation protein A [Escherichia coli UTI89] | 141.74 | 2 | 1243 1476 |  |  |
|  | [89109991](http://www.ncbi.nlm.nih.gov/entrez/query.fcgi?cmd=Search&db=Protein&term=89109991&doptcmdl=GenPept) | stringent starvation protein A [Escherichia coli W3110] | 141.74 | 2 | 1243 1476 |  |  |
|  | [85676022](http://www.ncbi.nlm.nih.gov/entrez/query.fcgi?cmd=Search&db=Protein&term=85676022&doptcmdl=GenPept) | stringent starvation protein A [Escherichia coli W3110] | 141.74 | 2 | 1243 1476 |  |  |
|  | [83585400](http://www.ncbi.nlm.nih.gov/entrez/query.fcgi?cmd=Search&db=Protein&term=83585400&doptcmdl=GenPept) | COG0625: Glutathione S-transferase [Escherichia coli 101-1] | 141.74 | 2 | 1243 1476 |  |  |
| 184 | [91211562](http://www.ncbi.nlm.nih.gov/entrez/query.fcgi?cmd=Search&db=Protein&term=91211562&doptcmdl=GenPept) | hypothetical protein UTI89_C2550 [Escherichia coli UTI89] | 140.58 | 2 | 210 431 |  |  |
|  | [91073136](http://www.ncbi.nlm.nih.gov/entrez/query.fcgi?cmd=Search&db=Protein&term=91073136&doptcmdl=GenPept) | hypothetical protein UTI89_C2550 [Escherichia coli UTI89] | 140.58 | 2 | 210 431 |  |  |
|  | [89109084](http://www.ncbi.nlm.nih.gov/entrez/query.fcgi?cmd=Search&db=Protein&term=89109084&doptcmdl=GenPept) | hypothetical protein [Escherichia coli W3110] | 140.58 | 2 | 210 431 |  |  |
|  | [83588253](http://www.ncbi.nlm.nih.gov/entrez/query.fcgi?cmd=Search&db=Protein&term=83588253&doptcmdl=GenPept) | COG4575: Uncharacterized conserved protein [Escherichia coli 101-1] | 140.58 | 2 | 210 431 |  |  |
|  | [83309000](http://www.ncbi.nlm.nih.gov/entrez/query.fcgi?cmd=Search&db=Protein&term=83309000&doptcmdl=GenPept) | Protein elaB | 140.58 | 2 | 210 431 |  |  |
| 185 | [91209759](http://www.ncbi.nlm.nih.gov/entrez/query.fcgi?cmd=Search&db=Protein&term=91209759&doptcmdl=GenPept) | alpha-ketoglutarate decarboxylase [Escherichia coli UTI89] | 139.64 | 3 | 435 1297 1304 |  |  |
|  | [91071333](http://www.ncbi.nlm.nih.gov/entrez/query.fcgi?cmd=Search&db=Protein&term=91071333&doptcmdl=GenPept) | 2-oxoglutarate dehydrogenase decarboxylase component [Escherichia coli UTI89] | 139.64 | 3 | 435 1297 1304 |  |  |
|  | [89107583](http://www.ncbi.nlm.nih.gov/entrez/query.fcgi?cmd=Search&db=Protein&term=89107583&doptcmdl=GenPept) | 2-oxoglutarate decarboxylase, thiamin-requiring [Escherichia coli W3110] | 139.64 | 3 | 435 1297 1304 |  |  |
|  | [84027822](http://www.ncbi.nlm.nih.gov/entrez/query.fcgi?cmd=Search&db=Protein&term=84027822&doptcmdl=GenPept) | 2-oxoglutarate dehydrogenase E1 component (Alpha-ketoglutarate dehydrogenase) | 139.64 | 3 | 435 1297 1304 |  |  |
|  | [84027821](http://www.ncbi.nlm.nih.gov/entrez/query.fcgi?cmd=Search&db=Protein&term=84027821&doptcmdl=GenPept) | 2-oxoglutarate dehydrogenase E1 component (Alpha-ketoglutarate dehydrogenase) | 139.64 | 3 | 435 1297 1304 |  |  |
| 186 | [91209573](http://www.ncbi.nlm.nih.gov/entrez/query.fcgi?cmd=Search&db=Protein&term=91209573&doptcmdl=GenPept) | putative thioredoxin-like protein [Escherichia coli UTI89] | 138.04 | 2 | 222 1611 |  |  |
|  | [91071147](http://www.ncbi.nlm.nih.gov/entrez/query.fcgi?cmd=Search&db=Protein&term=91071147&doptcmdl=GenPept) | putative thioredoxin-like protein [Escherichia coli UTI89] | 138.04 | 2 | 222 1611 |  |  |
|  | [90111142](http://www.ncbi.nlm.nih.gov/entrez/query.fcgi?cmd=Search&db=Protein&term=90111142&doptcmdl=GenPept) | predicted thioredoxin domain-containing protein [Escherichia coli str. K-12 substr. MG1655] | 138.04 | 2 | 222 1611 |  |  |
|  | [89107361](http://www.ncbi.nlm.nih.gov/entrez/query.fcgi?cmd=Search&db=Protein&term=89107361&doptcmdl=GenPept) | predicted thioredoxin domain-containing protein [Escherichia coli W3110] | 138.04 | 2 | 222 1611 |  |  |
|  | [87081747](http://www.ncbi.nlm.nih.gov/entrez/query.fcgi?cmd=Search&db=Protein&term=87081747&doptcmdl=GenPept) | predicted thioredoxin domain-containing protein [Escherichia coli str. K-12 substr. MG1655] | 138.04 | 2 | 222 1611 |  |  |
| 187 | [91208213](http://www.ncbi.nlm.nih.gov/entrez/query.fcgi?cmd=Search&db=Protein&term=91208213&doptcmdl=GenPept) | Phenylalanyl-tRNA synthetase alpha chain (Phenylalanine--tRNA ligase alpha chain) (PheRS) | 137.71 | 3 | 390 934 1334 |  |  |
|  | [91072502](http://www.ncbi.nlm.nih.gov/entrez/query.fcgi?cmd=Search&db=Protein&term=91072502&doptcmdl=GenPept) | phenylalanyl-tRNA synthetase alpha chain [Escherichia coli UTI89] | 137.71 | 3 | 390 934 1334 |  |  |
|  | [89108554](http://www.ncbi.nlm.nih.gov/entrez/query.fcgi?cmd=Search&db=Protein&term=89108554&doptcmdl=GenPept) | phenylalanine tRNA synthetase, alpha subunit [Escherichia coli W3110] | 137.71 | 3 | 390 934 1334 |  |  |
|  | [85675086](http://www.ncbi.nlm.nih.gov/entrez/query.fcgi?cmd=Search&db=Protein&term=85675086&doptcmdl=GenPept) | phenylalanine tRNA synthetase, alpha subunit [Escherichia coli W3110] | 137.71 | 3 | 390 934 1334 |  |  |
|  | [83586017](http://www.ncbi.nlm.nih.gov/entrez/query.fcgi?cmd=Search&db=Protein&term=83586017&doptcmdl=GenPept) | COG0016: Phenylalanyl-tRNA synthetase alpha subunit [Escherichia coli 101-1] | 137.71 | 3 | 390 934 1334 |  |  |
| 188 | [91210243](http://www.ncbi.nlm.nih.gov/entrez/query.fcgi?cmd=Search&db=Protein&term=91210243&doptcmdl=GenPept) | 3-oxoacyl-(acyl carrier protein) synthase III [Escherichia coli UTI89] | 137.07 | 3 | 30 952 1242 |  |  |
|  | [91071817](http://www.ncbi.nlm.nih.gov/entrez/query.fcgi?cmd=Search&db=Protein&term=91071817&doptcmdl=GenPept) | 3-oxoacyl-[acyl-carrier-protein] synthase III; acetylCoA ACP transacylase [Escherichia coli UTI89] | 137.07 | 3 | 30 952 1242 |  |  |
|  | [89107937](http://www.ncbi.nlm.nih.gov/entrez/query.fcgi?cmd=Search&db=Protein&term=89107937&doptcmdl=GenPept) | 3-oxoacyl-[acyl-carrier-protein] synthase III [Escherichia coli W3110] | 137.07 | 3 | 30 952 1242 |  |  |
|  | [85674835](http://www.ncbi.nlm.nih.gov/entrez/query.fcgi?cmd=Search&db=Protein&term=85674835&doptcmdl=GenPept) | 3-oxoacyl-[acyl-carrier-protein] synthase III [Escherichia coli W3110] | 137.07 | 3 | 30 952 1242 |  |  |
|  | [84028083](http://www.ncbi.nlm.nih.gov/entrez/query.fcgi?cmd=Search&db=Protein&term=84028083&doptcmdl=GenPept) | 3-oxoacyl-[acyl-carrier-protein] synthase 3 (3-oxoacyl-[acyl-carrier-protein] synthase III) (Beta-ketoacyl-ACP synthase III) (KAS III) (EcFabH) | 137.07 | 3 | 30 952 1242 |  |  |
| 189 | [91209655](http://www.ncbi.nlm.nih.gov/entrez/query.fcgi?cmd=Search&db=Protein&term=91209655&doptcmdl=GenPept) | universal stress protein UspG [Escherichia coli UTI89] | 136.83 | 2 | 877 1535 |  |  |
|  | [91071229](http://www.ncbi.nlm.nih.gov/entrez/query.fcgi?cmd=Search&db=Protein&term=91071229&doptcmdl=GenPept) | universal stress protein UspG [Escherichia coli UTI89] | 136.83 | 2 | 877 1535 |  |  |
|  | [89107475](http://www.ncbi.nlm.nih.gov/entrez/query.fcgi?cmd=Search&db=Protein&term=89107475&doptcmdl=GenPept) | universal stress protein UP12 [Escherichia coli W3110] | 136.83 | 2 | 877 1535 |  |  |
|  | [83586957](http://www.ncbi.nlm.nih.gov/entrez/query.fcgi?cmd=Search&db=Protein&term=83586957&doptcmdl=GenPept) | COG0589: Universal stress protein UspA and related nucleotide-binding proteins [Escherichia coli 101-1] [MASS=15185] | 136.83 | 2 | 877 1535 |  |  |
|  | [83570436](http://www.ncbi.nlm.nih.gov/entrez/query.fcgi?cmd=Search&db=Protein&term=83570436&doptcmdl=GenPept) | COG0589: Universal stress protein UspA and related nucleotide-binding proteins [Shigella dysenteriae 1012] [MASS=15186] | 136.83 | 2 | 877 1535 |  |  |
| 190 | [91210236](http://www.ncbi.nlm.nih.gov/entrez/query.fcgi?cmd=Search&db=Protein&term=91210236&doptcmdl=GenPept) | ribonuclease E [Escherichia coli UTI89] | 136.68 | 3 | 218 832 1154 |  |  |
|  | [91071810](http://www.ncbi.nlm.nih.gov/entrez/query.fcgi?cmd=Search&db=Protein&term=91071810&doptcmdl=GenPept) | RNase E [Escherichia coli UTI89] | 136.68 | 3 | 218 832 1154 |  |  |
|  | [89107930](http://www.ncbi.nlm.nih.gov/entrez/query.fcgi?cmd=Search&db=Protein&term=89107930&doptcmdl=GenPept) | fused ribonucleaseE endoribonuclease and scaffold for formation of degradosome [Escherichia coli W3110] | 136.68 | 3 | 218 832 1154 |  |  |
|  | [83586382](http://www.ncbi.nlm.nih.gov/entrez/query.fcgi?cmd=Search&db=Protein&term=83586382&doptcmdl=GenPept) | COG1530: Ribonucleases G and E [Escherichia coli 101-1] [MASS=117870] | 136.68 | 3 | 218 832 1154 |  |  |
|  | [75210355](http://www.ncbi.nlm.nih.gov/entrez/query.fcgi?cmd=Search&db=Protein&term=75210355&doptcmdl=GenPept) | COG1530: Ribonucleases G and E [Escherichia coli B171] [MASS=117870] | 136.68 | 3 | 218 832 1154 |  |  |
| 191 | [91209225](http://www.ncbi.nlm.nih.gov/entrez/query.fcgi?cmd=Search&db=Protein&term=91209225&doptcmdl=GenPept) | glutamate-1-semialdehyde aminotransferase [Escherichia coli UTI89] | 136.08 | 2 | 809 1285 |  |  |
|  | [91070799](http://www.ncbi.nlm.nih.gov/entrez/query.fcgi?cmd=Search&db=Protein&term=91070799&doptcmdl=GenPept) | glutamate-1-semialdehyde 2,1-aminomutase [Escherichia coli UTI89] | 136.08 | 2 | 809 1285 |  |  |
|  | [89107035](http://www.ncbi.nlm.nih.gov/entrez/query.fcgi?cmd=Search&db=Protein&term=89107035&doptcmdl=GenPept) | glutamate-1-semialdehyde aminotransferase [Escherichia coli W3110] | 136.08 | 2 | 809 1285 |  |  |
|  | [85674360](http://www.ncbi.nlm.nih.gov/entrez/query.fcgi?cmd=Search&db=Protein&term=85674360&doptcmdl=GenPept) | glutamate-1-semialdehyde aminotransferase [Escherichia coli W3110] | 136.08 | 2 | 809 1285 |  |  |
|  | [83585471](http://www.ncbi.nlm.nih.gov/entrez/query.fcgi?cmd=Search&db=Protein&term=83585471&doptcmdl=GenPept) | COG0001: Glutamate-1-semialdehyde aminotransferase [Escherichia coli 101-1] | 136.08 | 2 | 809 1285 |  |  |

| **Protein IDs*, cont.*** | | | | | | | |
| --- | --- | --- | --- | --- | --- | --- | --- |
| *Grp Nr.* | *Accession Number* | *Protein Name* | *Protein Score* | *Unique PSMs* | *PSM Serial Nrs.* | *Other Grp.* | *Score (other)* |
| 192 | [91211620](http://www.ncbi.nlm.nih.gov/entrez/query.fcgi?cmd=Search&db=Protein&term=91211620&doptcmdl=GenPept) | 3-oxoacyl-(acyl carrier protein) synthase I [Escherichia coli UTI89] | 136.07 | 2 | 448 1704 |  |  |
|  | [91073194](http://www.ncbi.nlm.nih.gov/entrez/query.fcgi?cmd=Search&db=Protein&term=91073194&doptcmdl=GenPept) | bifunctional beta-ketoacyl-ACP synthase I/malonyl-ACP decarboxylase [Escherichia coli UTI89] | 136.07 | 2 | 448 1704 |  |  |
|  | [90108927](http://www.ncbi.nlm.nih.gov/entrez/query.fcgi?cmd=Search&db=Protein&term=90108927&doptcmdl=GenPept) | Chain D, Structure Of E. Coli Kas I H298q Mutant [MASS=44003] | 136.07 | 2 | 448 1704 |  |  |
|  | [90108926](http://www.ncbi.nlm.nih.gov/entrez/query.fcgi?cmd=Search&db=Protein&term=90108926&doptcmdl=GenPept) | Chain C, Structure Of E. Coli Kas I H298q Mutant | 136.07 | 2 | 448 1704 |  |  |
|  | [90108925](http://www.ncbi.nlm.nih.gov/entrez/query.fcgi?cmd=Search&db=Protein&term=90108925&doptcmdl=GenPept) | Chain B, Structure Of E. Coli Kas I H298q Mutant | 136.07 | 2 | 448 1704 |  |  |
| 193 | [91210424](http://www.ncbi.nlm.nih.gov/entrez/query.fcgi?cmd=Search&db=Protein&term=91210424&doptcmdl=GenPept) | translation-associated GTPase [Escherichia coli UTI89] | 134.26 | 3 | 491 793 1171 |  |  |
|  | [91071998](http://www.ncbi.nlm.nih.gov/entrez/query.fcgi?cmd=Search&db=Protein&term=91071998&doptcmdl=GenPept) | probable GTP-binding protein YchF [Escherichia coli UTI89] | 134.26 | 3 | 491 793 1171 |  |  |
|  | [89108048](http://www.ncbi.nlm.nih.gov/entrez/query.fcgi?cmd=Search&db=Protein&term=89108048&doptcmdl=GenPept) | predicted GTP-binding protein [Escherichia coli W3110] | 134.26 | 3 | 491 793 1171 |  |  |
|  | [83587347](http://www.ncbi.nlm.nih.gov/entrez/query.fcgi?cmd=Search&db=Protein&term=83587347&doptcmdl=GenPept) | COG0012: Predicted GTPase, probable translation factor [Escherichia coli 101-1] | 134.26 | 3 | 491 793 1171 |  |  |
|  | [82776543](http://www.ncbi.nlm.nih.gov/entrez/query.fcgi?cmd=Search&db=Protein&term=82776543&doptcmdl=GenPept) | translation-associated GTPase [Shigella dysenteriae Sd197] | 134.26 | 3 | 491 793 1171 |  |  |
| 194 | [91210855](http://www.ncbi.nlm.nih.gov/entrez/query.fcgi?cmd=Search&db=Protein&term=91210855&doptcmdl=GenPept) | outer membrane lipoprotein SlyB precursor [Escherichia coli UTI89] | 134.25 | 2 | 644 685 |  |  |
|  | [91072429](http://www.ncbi.nlm.nih.gov/entrez/query.fcgi?cmd=Search&db=Protein&term=91072429&doptcmdl=GenPept) | outer membrane lipoprotein SlyB precursor [Escherichia coli UTI89] | 134.25 | 2 | 644 685 |  |  |
|  | [89108483](http://www.ncbi.nlm.nih.gov/entrez/query.fcgi?cmd=Search&db=Protein&term=89108483&doptcmdl=GenPept) | outer membrane lipoprotein [Escherichia coli W3110] | 134.25 | 2 | 644 685 |  |  |
|  | [83587976](http://www.ncbi.nlm.nih.gov/entrez/query.fcgi?cmd=Search&db=Protein&term=83587976&doptcmdl=GenPept) | COG3133: Outer membrane lipoprotein [Escherichia coli 101-1] | 134.25 | 2 | 644 685 |  |  |
|  | [82777115](http://www.ncbi.nlm.nih.gov/entrez/query.fcgi?cmd=Search&db=Protein&term=82777115&doptcmdl=GenPept) | putative outer membrane protein [Shigella dysenteriae Sd197] | 134.25 | 2 | 644 685 |  |  |
| 195 | [91211841](http://www.ncbi.nlm.nih.gov/entrez/query.fcgi?cmd=Search&db=Protein&term=91211841&doptcmdl=GenPept) | 4-hydroxy-3-methylbut-2-en-1-yl diphosphate synthase [Escherichia coli UTI89] | 132.49 | 3 | 678 912 927 |  |  |
|  | [91073415](http://www.ncbi.nlm.nih.gov/entrez/query.fcgi?cmd=Search&db=Protein&term=91073415&doptcmdl=GenPept) | 1-hydroxy-2-methyl-2-(E)-butenyl 4-diphosphate synthase [Escherichia coli UTI89] | 132.49 | 3 | 678 912 927 |  |  |
|  | [89109321](http://www.ncbi.nlm.nih.gov/entrez/query.fcgi?cmd=Search&db=Protein&term=89109321&doptcmdl=GenPept) | 1-hydroxy-2-methyl-2-(E)-butenyl 4-diphosphate synthase [Escherichia coli W3110] | 132.49 | 3 | 678 912 927 |  |  |
|  | [85675435](http://www.ncbi.nlm.nih.gov/entrez/query.fcgi?cmd=Search&db=Protein&term=85675435&doptcmdl=GenPept) | 1-hydroxy-2-methyl-2-(E)-butenyl 4-diphosphate synthase [Escherichia coli W3110] | 132.49 | 3 | 678 912 927 |  |  |
|  | [83586836](http://www.ncbi.nlm.nih.gov/entrez/query.fcgi?cmd=Search&db=Protein&term=83586836&doptcmdl=GenPept) | COG0821: Enzyme involved in the deoxyxylulose pathway of isoprenoid biosynthesis [Escherichia coli 101-1] [MASS=38464] | 132.49 | 3 | 678 912 927 |  |  |
| 196 | [91210008](http://www.ncbi.nlm.nih.gov/entrez/query.fcgi?cmd=Search&db=Protein&term=91210008&doptcmdl=GenPept) | phosphoserine aminotransferase [Escherichia coli UTI89] | 132.07 | 2 | 984 1451 |  |  |
|  | [91071582](http://www.ncbi.nlm.nih.gov/entrez/query.fcgi?cmd=Search&db=Protein&term=91071582&doptcmdl=GenPept) | phosphoserine aminotransferase [Escherichia coli UTI89] | 132.07 | 2 | 984 1451 |  |  |
|  | [89107757](http://www.ncbi.nlm.nih.gov/entrez/query.fcgi?cmd=Search&db=Protein&term=89107757&doptcmdl=GenPept) | 3-phosphoserine/phosphohydroxythreonine aminotransferase [Escherichia coli W3110] | 132.07 | 2 | 984 1451 |  |  |
|  | [83585850](http://www.ncbi.nlm.nih.gov/entrez/query.fcgi?cmd=Search&db=Protein&term=83585850&doptcmdl=GenPept) | COG1932: Phosphoserine aminotransferase [Escherichia coli 101-1] | 132.07 | 2 | 984 1451 |  |  |
|  | [83569146](http://www.ncbi.nlm.nih.gov/entrez/query.fcgi?cmd=Search&db=Protein&term=83569146&doptcmdl=GenPept) | COG1932: Phosphoserine aminotransferase [Shigella dysenteriae 1012] | 132.07 | 2 | 984 1451 |  |  |
| 197 | [91073517](http://www.ncbi.nlm.nih.gov/entrez/query.fcgi?cmd=Search&db=Protein&term=91073517&doptcmdl=GenPept) | 30S ribosomal protein S16 [Escherichia coli UTI89] | 130.69 | 3 | 586 594 966 |  |  |
|  | [89109410](http://www.ncbi.nlm.nih.gov/entrez/query.fcgi?cmd=Search&db=Protein&term=89109410&doptcmdl=GenPept) | 30S ribosomal subunit protein S16 [Escherichia coli W3110] | 130.69 | 3 | 586 594 966 |  |  |
|  | [85675480](http://www.ncbi.nlm.nih.gov/entrez/query.fcgi?cmd=Search&db=Protein&term=85675480&doptcmdl=GenPept) | 30S ribosomal subunit protein S16 [Escherichia coli W3110] | 130.69 | 3 | 586 594 966 |  |  |
|  | [83754110](http://www.ncbi.nlm.nih.gov/entrez/query.fcgi?cmd=Search&db=Protein&term=83754110&doptcmdl=GenPept) | Chain P, Crystal Structure Of The Bacterial Ribosome From Escherichia Coli At 3.5 A Resolution. This File Contains The 30s Subunit Of The Second 70s Ribosome. The Entire Crystal Structure Contains Two 70s Ribosomes And Is Described In Remark 400. | 130.69 | 3 | 586 594 966 |  |  |
|  | [83754054](http://www.ncbi.nlm.nih.gov/entrez/query.fcgi?cmd=Search&db=Protein&term=83754054&doptcmdl=GenPept) | Chain P, Crystal Structure Of The Bacterial Ribosome From Escherichia Coli At 3.5 A Resolution. This File Contains The 30s Subunit Of One 70s Ribosome. The Entire Crystal Structure Contains Two 70s Ribosomes And Is Described In Remark 400. | 130.69 | 3 | 586 594 966 |  |  |
| 198 | [91210247](http://www.ncbi.nlm.nih.gov/entrez/query.fcgi?cmd=Search&db=Protein&term=91210247&doptcmdl=GenPept) | acyl carrier protein [Escherichia coli UTI89] | 129.87 | 2 | 628 1529 |  |  |
|  | [91071821](http://www.ncbi.nlm.nih.gov/entrez/query.fcgi?cmd=Search&db=Protein&term=91071821&doptcmdl=GenPept) | acyl carrier protein [Escherichia coli UTI89] | 129.87 | 2 | 628 1529 |  |  |
|  | [89107940](http://www.ncbi.nlm.nih.gov/entrez/query.fcgi?cmd=Search&db=Protein&term=89107940&doptcmdl=GenPept) | acyl carrier protein [Escherichia coli W3110] | 129.87 | 2 | 628 1529 |  |  |
|  | [82777293](http://www.ncbi.nlm.nih.gov/entrez/query.fcgi?cmd=Search&db=Protein&term=82777293&doptcmdl=GenPept) | acyl carrier protein [Shigella dysenteriae Sd197] | 129.87 | 2 | 628 1529 |  |  |
|  | [82544440](http://www.ncbi.nlm.nih.gov/entrez/query.fcgi?cmd=Search&db=Protein&term=82544440&doptcmdl=GenPept) | acyl carrier protein [Shigella boydii Sb227] | 129.87 | 2 | 628 1529 |  |  |
| 199 | [USG_ECOLI](http://us.expasy.org/uniprot/USG_ECOLI) | USG-1 protein | 129.33 | 3 | 184 209 1655 |  |  |
|  | [91211616](http://www.ncbi.nlm.nih.gov/entrez/query.fcgi?cmd=Search&db=Protein&term=91211616&doptcmdl=GenPept) | hypothetical protein UTI89_C2604 [Escherichia coli UTI89] | 77.57 | 2 | 184 1655 |  |  |
| 200 | [91212993](http://www.ncbi.nlm.nih.gov/entrez/query.fcgi?cmd=Search&db=Protein&term=91212993&doptcmdl=GenPept) | glutathione reductase [Escherichia coli UTI89] | 129.06 | 2 | 739 1549 |  |  |
|  | [91074567](http://www.ncbi.nlm.nih.gov/entrez/query.fcgi?cmd=Search&db=Protein&term=91074567&doptcmdl=GenPept) | glutathione reductase [Escherichia coli UTI89] | 129.06 | 2 | 739 1549 |  |  |
|  | [89110513](http://www.ncbi.nlm.nih.gov/entrez/query.fcgi?cmd=Search&db=Protein&term=89110513&doptcmdl=GenPept) | glutathione oxidoreductase [Escherichia coli W3110] | 129.06 | 2 | 739 1549 |  |  |
|  | [85676544](http://www.ncbi.nlm.nih.gov/entrez/query.fcgi?cmd=Search&db=Protein&term=85676544&doptcmdl=GenPept) | glutathione oxidoreductase [Escherichia coli W3110] | 129.06 | 2 | 739 1549 |  |  |
|  | [83588642](http://www.ncbi.nlm.nih.gov/entrez/query.fcgi?cmd=Search&db=Protein&term=83588642&doptcmdl=GenPept) | COG1249: Pyruvate/2-oxoglutarate dehydrogenase complex, dihydrolipoamide dehydrogenase (E3) component, and related enzymes [Escherichia coli 101-1] | 129.06 | 2 | 739 1549 |  |  |
| 201 | [97181965](http://www.ncbi.nlm.nih.gov/entrez/query.fcgi?cmd=Search&db=Protein&term=97181965&doptcmdl=GenPept) | 50S ribosomal protein L10 | 128.86 | 3 | 647 1075 1187 |  |  |
|  | [97181955](http://www.ncbi.nlm.nih.gov/entrez/query.fcgi?cmd=Search&db=Protein&term=97181955&doptcmdl=GenPept) | 50S ribosomal protein L10 | 128.86 | 3 | 647 1075 1187 |  |  |
|  | [97181947](http://www.ncbi.nlm.nih.gov/entrez/query.fcgi?cmd=Search&db=Protein&term=97181947&doptcmdl=GenPept) | 50S ribosomal protein L10 | 128.86 | 3 | 647 1075 1187 |  |  |
|  | [91212809](http://www.ncbi.nlm.nih.gov/entrez/query.fcgi?cmd=Search&db=Protein&term=91212809&doptcmdl=GenPept) | 50S ribosomal protein L10 [Escherichia coli UTI89] | 128.86 | 3 | 647 1075 1187 |  |  |
|  | [91074383](http://www.ncbi.nlm.nih.gov/entrez/query.fcgi?cmd=Search&db=Protein&term=91074383&doptcmdl=GenPept) | 50S ribosomal subunit protein L10 [Escherichia coli UTI89] | 128.86 | 3 | 647 1075 1187 |  |  |
| 202 | [91209193](http://www.ncbi.nlm.nih.gov/entrez/query.fcgi?cmd=Search&db=Protein&term=91209193&doptcmdl=GenPept) | hypoxanthine phosphoribosyltransferase [Escherichia coli UTI89] | 128.77 | 1 | 45 |  |  |
|  | [91070767](http://www.ncbi.nlm.nih.gov/entrez/query.fcgi?cmd=Search&db=Protein&term=91070767&doptcmdl=GenPept) | hypoxanthine phosphoribosyltransferase [Escherichia coli UTI89] | 128.77 | 1 | 45 |  |  |
|  | [90111088](http://www.ncbi.nlm.nih.gov/entrez/query.fcgi?cmd=Search&db=Protein&term=90111088&doptcmdl=GenPept) | hypoxanthine phosphoribosyltransferase [Escherichia coli str. K-12 substr. MG1655] | 128.77 | 1 | 45 |  |  |
|  | [89107006](http://www.ncbi.nlm.nih.gov/entrez/query.fcgi?cmd=Search&db=Protein&term=89107006&doptcmdl=GenPept) | hypoxanthine phosphoribosyltransferase [Escherichia coli W3110] | 128.77 | 1 | 45 |  |  |
|  | [87081689](http://www.ncbi.nlm.nih.gov/entrez/query.fcgi?cmd=Search&db=Protein&term=87081689&doptcmdl=GenPept) | hypoxanthine phosphoribosyltransferase [Escherichia coli str. K-12 substr. MG1655] | 128.77 | 1 | 45 |  |  |
| 203 | [9954966](http://www.ncbi.nlm.nih.gov/entrez/query.fcgi?cmd=Search&db=Protein&term=9954966&doptcmdl=GenPept) | Chain D, Crystal Structure Of Chymotryptic Fragment Of E. Coli Ssb Bound To Two 35-Mer Single Strand Dnas [MASS=12891] | 128.77 | 3 | 637 861 1111 |  |  |
|  | [9954965](http://www.ncbi.nlm.nih.gov/entrez/query.fcgi?cmd=Search&db=Protein&term=9954965&doptcmdl=GenPept) | Chain C, Crystal Structure Of Chymotryptic Fragment Of E. Coli Ssb Bound To Two 35-Mer Single Strand Dnas | 128.77 | 3 | 637 861 1111 |  |  |
|  | [9954964](http://www.ncbi.nlm.nih.gov/entrez/query.fcgi?cmd=Search&db=Protein&term=9954964&doptcmdl=GenPept) | Chain B, Crystal Structure Of Chymotryptic Fragment Of E. Coli Ssb Bound To Two 35-Mer Single Strand Dnas | 128.77 | 3 | 637 861 1111 |  |  |
|  | [9954963](http://www.ncbi.nlm.nih.gov/entrez/query.fcgi?cmd=Search&db=Protein&term=9954963&doptcmdl=GenPept) | Chain A, Crystal Structure Of Chymotryptic Fragment Of E. Coli Ssb Bound To Two 35-Mer Single Strand Dnas | 128.77 | 3 | 637 861 1111 |  |  |
|  | [91075172](http://www.ncbi.nlm.nih.gov/entrez/query.fcgi?cmd=Search&db=Protein&term=91075172&doptcmdl=GenPept) | single strand DNA-binding protein [Escherichia coli UTI89] [MASS=21475] | 128.77 | 3 | 637 861 1111 |  |  |
| 204 | [91212612](http://www.ncbi.nlm.nih.gov/entrez/query.fcgi?cmd=Search&db=Protein&term=91212612&doptcmdl=GenPept) | UDP-N-acetylglucosamine 1-carboxyvinyltransferase [Escherichia coli UTI89] | 128.11 | 3 | 181 940 1552 |  |  |
|  | [91207307](http://www.ncbi.nlm.nih.gov/entrez/query.fcgi?cmd=Search&db=Protein&term=91207307&doptcmdl=GenPept) | UDP-N-acetylglucosamine 1-carboxyvinyltransferase (Enoylpyruvate transferase) (UDP-N-acetylglucosamine enolpyruvyl transferase) (EPT) | 128.11 | 3 | 181 940 1552 |  |  |
|  | [91074186](http://www.ncbi.nlm.nih.gov/entrez/query.fcgi?cmd=Search&db=Protein&term=91074186&doptcmdl=GenPept) | UDP-N-glucosamine 1-carboxyvinyltransferase [Escherichia coli UTI89] | 128.11 | 3 | 181 940 1552 |  |  |
|  | [89109952](http://www.ncbi.nlm.nih.gov/entrez/query.fcgi?cmd=Search&db=Protein&term=89109952&doptcmdl=GenPept) | UDP-N-acetylglucosamine 1-carboxyvinyltransferase [Escherichia coli W3110] | 128.11 | 3 | 181 940 1552 |  |  |
|  | [85675983](http://www.ncbi.nlm.nih.gov/entrez/query.fcgi?cmd=Search&db=Protein&term=85675983&doptcmdl=GenPept) | UDP-N-acetylglucosamine 1-carboxyvinyltransferase [Escherichia coli W3110] | 128.11 | 3 | 181 940 1552 |  |  |
| 205 | [91213469](http://www.ncbi.nlm.nih.gov/entrez/query.fcgi?cmd=Search&db=Protein&term=91213469&doptcmdl=GenPept) | hypothetical protein UTI89_C4512 [Escherichia coli UTI89] | 128.05 | 2 | 427 568 |  |  |
|  | [91075043](http://www.ncbi.nlm.nih.gov/entrez/query.fcgi?cmd=Search&db=Protein&term=91075043&doptcmdl=GenPept) | conserved hypothetical protein [Escherichia coli UTI89] [MASS=10411] | 128.05 | 2 | 427 568 |  |  |
|  | [89110101](http://www.ncbi.nlm.nih.gov/entrez/query.fcgi?cmd=Search&db=Protein&term=89110101&doptcmdl=GenPept) | hypothetical protein [Escherichia coli W3110] | 128.05 | 2 | 427 568 |  |  |
|  | [85676132](http://www.ncbi.nlm.nih.gov/entrez/query.fcgi?cmd=Search&db=Protein&term=85676132&doptcmdl=GenPept) | conserved hypothetical protein [Escherichia coli W3110] | 128.05 | 2 | 427 568 |  |  |
|  | [84028029](http://www.ncbi.nlm.nih.gov/entrez/query.fcgi?cmd=Search&db=Protein&term=84028029&doptcmdl=GenPept) | Cell division protein zapB | 128.05 | 2 | 427 568 |  |  |
| 206 | [97196865](http://www.ncbi.nlm.nih.gov/entrez/query.fcgi?cmd=Search&db=Protein&term=97196865&doptcmdl=GenPept) | Aspartyl-tRNA synthetase (Aspartate--tRNA ligase) (AspRS) | 125.97 | 3 | 576 710 1649 |  |  |
|  | [9256906](http://www.ncbi.nlm.nih.gov/entrez/query.fcgi?cmd=Search&db=Protein&term=9256906&doptcmdl=GenPept) | Chain C, Crystal Structure Of Free Aspartyl-Trna Synthetase From Escherichia Coli | 125.97 | 3 | 576 710 1649 |  |  |
|  | [9256905](http://www.ncbi.nlm.nih.gov/entrez/query.fcgi?cmd=Search&db=Protein&term=9256905&doptcmdl=GenPept) | Chain B, Crystal Structure Of Free Aspartyl-Trna Synthetase From Escherichia Coli | 125.97 | 3 | 576 710 1649 |  |  |
|  | [9256904](http://www.ncbi.nlm.nih.gov/entrez/query.fcgi?cmd=Search&db=Protein&term=9256904&doptcmdl=GenPept) | Chain A, Crystal Structure Of Free Aspartyl-Trna Synthetase From Escherichia Coli | 125.97 | 3 | 576 710 1649 |  |  |
|  | [91211091](http://www.ncbi.nlm.nih.gov/entrez/query.fcgi?cmd=Search&db=Protein&term=91211091&doptcmdl=GenPept) | aspartyl-tRNA synthetase [Escherichia coli UTI89] | 125.97 | 3 | 576 710 1649 |  |  |
| 207 | [91210933](http://www.ncbi.nlm.nih.gov/entrez/query.fcgi?cmd=Search&db=Protein&term=91210933&doptcmdl=GenPept) | threonyl-tRNA synthetase [Escherichia coli UTI89] | 124.49 | 2 | 421 830 |  |  |
|  | [91072507](http://www.ncbi.nlm.nih.gov/entrez/query.fcgi?cmd=Search&db=Protein&term=91072507&doptcmdl=GenPept) | threonyl-tRNA synthetase [Escherichia coli UTI89] | 124.49 | 2 | 421 830 |  |  |
|  | [89108559](http://www.ncbi.nlm.nih.gov/entrez/query.fcgi?cmd=Search&db=Protein&term=89108559&doptcmdl=GenPept) | threonyl-tRNA synthetase [Escherichia coli W3110] | 124.49 | 2 | 421 830 |  |  |
|  | [83586021](http://www.ncbi.nlm.nih.gov/entrez/query.fcgi?cmd=Search&db=Protein&term=83586021&doptcmdl=GenPept) | COG0441: Threonyl-tRNA synthetase [Escherichia coli 101-1] [MASS=71811] | 124.49 | 2 | 421 830 |  |  |
|  | [75210206](http://www.ncbi.nlm.nih.gov/entrez/query.fcgi?cmd=Search&db=Protein&term=75210206&doptcmdl=GenPept) | COG0441: Threonyl-tRNA synthetase [Escherichia coli B171] [MASS=71797] | 124.49 | 2 | 421 830 |  |  |
| 208 | [92090398](http://www.ncbi.nlm.nih.gov/entrez/query.fcgi?cmd=Search&db=Protein&term=92090398&doptcmdl=GenPept) | Nucleoside diphosphate kinase (NDK) (NDP kinase) (Nucleoside-2-P kinase) | 123.06 | 2 | 657 1538 |  |  |
|  | [91211845](http://www.ncbi.nlm.nih.gov/entrez/query.fcgi?cmd=Search&db=Protein&term=91211845&doptcmdl=GenPept) | nucleoside diphosphate kinase [Escherichia coli UTI89] | 123.06 | 2 | 657 1538 |  |  |
|  | [91073419](http://www.ncbi.nlm.nih.gov/entrez/query.fcgi?cmd=Search&db=Protein&term=91073419&doptcmdl=GenPept) | nucleoside diphosphate kinase [Escherichia coli UTI89] | 123.06 | 2 | 657 1538 |  |  |
|  | [90110374](http://www.ncbi.nlm.nih.gov/entrez/query.fcgi?cmd=Search&db=Protein&term=90110374&doptcmdl=GenPept) | Nucleoside diphosphate kinase (NDK) (NDP kinase) (Nucleoside-2-P kinase) | 123.06 | 2 | 657 1538 |  |  |
|  | [90110373](http://www.ncbi.nlm.nih.gov/entrez/query.fcgi?cmd=Search&db=Protein&term=90110373&doptcmdl=GenPept) | Nucleoside diphosphate kinase (NDK) (NDP kinase) (Nucleoside-2-P kinase) | 123.06 | 2 | 657 1538 |  |  |
| 209 | [91214109](http://www.ncbi.nlm.nih.gov/entrez/query.fcgi?cmd=Search&db=Protein&term=91214109&doptcmdl=GenPept) | putative ABC transporter ATP-binding protein [Escherichia coli UTI89] | 122.87 | 2 | 166 1244 |  |  |
|  | [91075683](http://www.ncbi.nlm.nih.gov/entrez/query.fcgi?cmd=Search&db=Protein&term=91075683&doptcmdl=GenPept) | ABC transporter ATP-binding protein YjjK [Escherichia coli UTI89] | 122.87 | 2 | 166 1244 |  |  |
|  | [89111099](http://www.ncbi.nlm.nih.gov/entrez/query.fcgi?cmd=Search&db=Protein&term=89111099&doptcmdl=GenPept) | fused predicted transporter subunits and ATP-binding components of ABC superfamily [Escherichia coli W3110] | 122.87 | 2 | 166 1244 |  |  |
|  | [85677130](http://www.ncbi.nlm.nih.gov/entrez/query.fcgi?cmd=Search&db=Protein&term=85677130&doptcmdl=GenPept) | fused predicted transporter subunits and ATP-binding components of ABC superfamily [Escherichia coli W3110] | 122.87 | 2 | 166 1244 |  |  |
|  | [83585298](http://www.ncbi.nlm.nih.gov/entrez/query.fcgi?cmd=Search&db=Protein&term=83585298&doptcmdl=GenPept) | COG0488: ATPase components of ABC transporters with duplicated ATPase domains [Escherichia coli 101-1] [MASS=61601] | 122.87 | 2 | 166 1244 |  |  |
| 210 | [91213800](http://www.ncbi.nlm.nih.gov/entrez/query.fcgi?cmd=Search&db=Protein&term=91213800&doptcmdl=GenPept) | aspartate carbamoyltransferase catalytic subunit [Escherichia coli UTI89] | 122.65 | 2 | 1014 1483 |  |  |
|  | [91075374](http://www.ncbi.nlm.nih.gov/entrez/query.fcgi?cmd=Search&db=Protein&term=91075374&doptcmdl=GenPept) | PyrB, subunit of aspartatetranscarbamylase, catalytic subunit and aspartate-carbamoyltransferase [Escherichia coli UTI89] | 122.65 | 2 | 1014 1483 |  |  |
|  | [89110963](http://www.ncbi.nlm.nih.gov/entrez/query.fcgi?cmd=Search&db=Protein&term=89110963&doptcmdl=GenPept) | aspartate carbamoyltransferase, catalytic subunit [Escherichia coli W3110] | 122.65 | 2 | 1014 1483 |  |  |
|  | [88192068](http://www.ncbi.nlm.nih.gov/entrez/query.fcgi?cmd=Search&db=Protein&term=88192068&doptcmdl=GenPept) | Chain G, T-State Active Site Of Aspartate Transcarbamylase:crystal Structure Of The Carbamyl Phosphate And L-Alanosine Ligated Enzyme | 122.65 | 2 | 1014 1483 |  |  |
|  | [88192066](http://www.ncbi.nlm.nih.gov/entrez/query.fcgi?cmd=Search&db=Protein&term=88192066&doptcmdl=GenPept) | Chain A, T-State Active Site Of Aspartate Transcarbamylase:crystal Structure Of The Carbamyl Phosphate And L-Alanosine Ligated Enzyme | 122.65 | 2 | 1014 1483 |  |  |
| 211 | [91213722](http://www.ncbi.nlm.nih.gov/entrez/query.fcgi?cmd=Search&db=Protein&term=91213722&doptcmdl=GenPept) | putative GTPase HflX [Escherichia coli UTI89] | 122.43 | 2 | 124 182 |  |  |
|  | [91075296](http://www.ncbi.nlm.nih.gov/entrez/query.fcgi?cmd=Search&db=Protein&term=91075296&doptcmdl=GenPept) | GTP-binding protein HflX [Escherichia coli UTI89] | 122.43 | 2 | 124 182 |  |  |
|  | [89110893](http://www.ncbi.nlm.nih.gov/entrez/query.fcgi?cmd=Search&db=Protein&term=89110893&doptcmdl=GenPept) | predicted GTPase [Escherichia coli W3110] | 122.43 | 2 | 124 182 |  |  |
|  | [85676924](http://www.ncbi.nlm.nih.gov/entrez/query.fcgi?cmd=Search&db=Protein&term=85676924&doptcmdl=GenPept) | predicted GTPase [Escherichia coli W3110] | 122.43 | 2 | 124 182 |  |  |
|  | [83585089](http://www.ncbi.nlm.nih.gov/entrez/query.fcgi?cmd=Search&db=Protein&term=83585089&doptcmdl=GenPept) | COG2262: GTPases [Escherichia coli 101-1] | 122.43 | 2 | 124 182 |  |  |
| 212 | [91212873](http://www.ncbi.nlm.nih.gov/entrez/query.fcgi?cmd=Search&db=Protein&term=91212873&doptcmdl=GenPept) | Hsp33-like chaperonin [Escherichia coli UTI89] | 121.43 | 2 | 231 1630 |  |  |
|  | [91074447](http://www.ncbi.nlm.nih.gov/entrez/query.fcgi?cmd=Search&db=Protein&term=91074447&doptcmdl=GenPept) | heat shock protein 33 [Escherichia coli UTI89] | 121.43 | 2 | 231 1630 |  |  |
|  | [90111586](http://www.ncbi.nlm.nih.gov/entrez/query.fcgi?cmd=Search&db=Protein&term=90111586&doptcmdl=GenPept) | heat shock protein Hsp33 [Escherichia coli str. K-12 substr. MG1655] | 121.43 | 2 | 231 1630 |  |  |
|  | [89110609](http://www.ncbi.nlm.nih.gov/entrez/query.fcgi?cmd=Search&db=Protein&term=89110609&doptcmdl=GenPept) | heat shock protein Hsp33 [Escherichia coli W3110] | 121.43 | 2 | 231 1630 |  |  |
|  | [87082260](http://www.ncbi.nlm.nih.gov/entrez/query.fcgi?cmd=Search&db=Protein&term=87082260&doptcmdl=GenPept) | heat shock protein Hsp33 [Escherichia coli str. K-12 substr. MG1655] | 121.43 | 2 | 231 1630 |  |  |
| 213 | [91211456](http://www.ncbi.nlm.nih.gov/entrez/query.fcgi?cmd=Search&db=Protein&term=91211456&doptcmdl=GenPept) | 1-phosphofructokinase [Escherichia coli UTI89] | 121.21 | 2 | 95 1033 |  |  |
|  | [91073030](http://www.ncbi.nlm.nih.gov/entrez/query.fcgi?cmd=Search&db=Protein&term=91073030&doptcmdl=GenPept) | fructose-1-phosphate kinase [Escherichia coli UTI89] | 121.21 | 2 | 95 1033 |  |  |
|  | [89108985](http://www.ncbi.nlm.nih.gov/entrez/query.fcgi?cmd=Search&db=Protein&term=89108985&doptcmdl=GenPept) | fructose-1-phosphate kinase [Escherichia coli W3110] | 121.21 | 2 | 95 1033 |  |  |
|  | [84029477](http://www.ncbi.nlm.nih.gov/entrez/query.fcgi?cmd=Search&db=Protein&term=84029477&doptcmdl=GenPept) | 1-phosphofructokinase (Fructose 1-phosphate kinase) | 121.21 | 2 | 95 1033 |  |  |
|  | [84029476](http://www.ncbi.nlm.nih.gov/entrez/query.fcgi?cmd=Search&db=Protein&term=84029476&doptcmdl=GenPept) | 1-phosphofructokinase (Fructose 1-phosphate kinase) | 121.21 | 2 | 95 1033 |  |  |
| 214 | [91209495](http://www.ncbi.nlm.nih.gov/entrez/query.fcgi?cmd=Search&db=Protein&term=91209495&doptcmdl=GenPept) | nucleotide-binding protein [Escherichia coli UTI89] | 120.23 | 2 | 83 441 |  |  |
|  | [91071069](http://www.ncbi.nlm.nih.gov/entrez/query.fcgi?cmd=Search&db=Protein&term=91071069&doptcmdl=GenPept) | hypothetical protein UTI89_C0449 [Escherichia coli UTI89] [MASS=24624] | 120.23 | 2 | 83 441 |  |  |
|  | [90111132](http://www.ncbi.nlm.nih.gov/entrez/query.fcgi?cmd=Search&db=Protein&term=90111132&doptcmdl=GenPept) | predicted nucleotide binding protein [Escherichia coli str. K-12 substr. MG1655] | 120.23 | 2 | 83 441 |  |  |
|  | [89107296](http://www.ncbi.nlm.nih.gov/entrez/query.fcgi?cmd=Search&db=Protein&term=89107296&doptcmdl=GenPept) | predicted nucleotide binding protein [Escherichia coli W3110] | 120.23 | 2 | 83 441 |  |  |
|  | [87081737](http://www.ncbi.nlm.nih.gov/entrez/query.fcgi?cmd=Search&db=Protein&term=87081737&doptcmdl=GenPept) | predicted nucleotide binding protein [Escherichia coli str. K-12 substr. MG1655] | 120.23 | 2 | 83 441 |  |  |
| 215 | [91211101](http://www.ncbi.nlm.nih.gov/entrez/query.fcgi?cmd=Search&db=Protein&term=91211101&doptcmdl=GenPept) | arginyl-tRNA synthetase [Escherichia coli UTI89] | 120.21 | 3 | 567 825 1452 |  |  |
|  | [91072675](http://www.ncbi.nlm.nih.gov/entrez/query.fcgi?cmd=Search&db=Protein&term=91072675&doptcmdl=GenPept) | arginine tRNA synthetase [Escherichia coli UTI89] | 120.21 | 3 | 567 825 1452 |  |  |
|  | [89108716](http://www.ncbi.nlm.nih.gov/entrez/query.fcgi?cmd=Search&db=Protein&term=89108716&doptcmdl=GenPept) | arginyl-tRNA synthetase [Escherichia coli W3110] | 120.21 | 3 | 567 825 1452 |  |  |
|  | [83585523](http://www.ncbi.nlm.nih.gov/entrez/query.fcgi?cmd=Search&db=Protein&term=83585523&doptcmdl=GenPept) | COG0018: Arginyl-tRNA synthetase [Escherichia coli 101-1] [MASS=63099] | 120.21 | 3 | 567 825 1452 |  |  |
|  | [75209446](http://www.ncbi.nlm.nih.gov/entrez/query.fcgi?cmd=Search&db=Protein&term=75209446&doptcmdl=GenPept) | COG0018: Arginyl-tRNA synthetase [Escherichia coli B171] [MASS=63131] | 120.21 | 3 | 567 825 1452 |  |  |

| **Protein IDs*, cont.*** | | | | | | | |
| --- | --- | --- | --- | --- | --- | --- | --- |
| *Grp Nr.* | *Accession Number* | *Protein Name* | *Protein Score* | *Unique PSMs* | *PSM Serial Nrs.* | *Other Grp.* | *Score (other)* |
| 216 | [91207530](http://www.ncbi.nlm.nih.gov/entrez/query.fcgi?cmd=Search&db=Protein&term=91207530&doptcmdl=GenPept) | Glutamate 5-kinase (Gamma-glutamyl kinase) (GK) | 119.27 | 2 | 32 519 |  |  |
|  | [91207529](http://www.ncbi.nlm.nih.gov/entrez/query.fcgi?cmd=Search&db=Protein&term=91207529&doptcmdl=GenPept) | Glutamate 5-kinase (Gamma-glutamyl kinase) (GK) | 119.27 | 2 | 32 519 |  |  |
|  | [91207528](http://www.ncbi.nlm.nih.gov/entrez/query.fcgi?cmd=Search&db=Protein&term=91207528&doptcmdl=GenPept) | Glutamate 5-kinase (Gamma-glutamyl kinase) (GK) | 119.27 | 2 | 32 519 |  |  |
|  | [91070905](http://www.ncbi.nlm.nih.gov/entrez/query.fcgi?cmd=Search&db=Protein&term=91070905&doptcmdl=GenPept) | glutamate 5-kinase [Escherichia coli UTI89] [MASS=40733] | 119.27 | 2 | 32 519 |  |  |
|  | [89107115](http://www.ncbi.nlm.nih.gov/entrez/query.fcgi?cmd=Search&db=Protein&term=89107115&doptcmdl=GenPept) | gamma-glutamate kinase [Escherichia coli W3110] | 119.27 | 2 | 32 519 |  |  |
| 217 | [89108860](http://www.ncbi.nlm.nih.gov/entrez/query.fcgi?cmd=Search&db=Protein&term=89108860&doptcmdl=GenPept) | dTDP-4-dehydrorhamnose reductase subunit, NAD(P)-binding, of dTDP-L-rhamnose synthase [Escherichia coli W3110] | 118.54 | 2 | 699 1705 |  |  |
|  | [799233](http://www.ncbi.nlm.nih.gov/entrez/query.fcgi?cmd=Search&db=Protein&term=799233&doptcmdl=GenPept) | RmlD [Escherichia coli] [MASS=32866] | 118.54 | 2 | 699 1705 |  |  |
|  | [73809590](http://www.ncbi.nlm.nih.gov/entrez/query.fcgi?cmd=Search&db=Protein&term=73809590&doptcmdl=GenPept) | dTDP-6-deoxy-D-glucose-3,5 epimerase [Escherichia coli] [MASS=32637] | 118.54 | 2 | 699 1705 |  |  |
|  | [73809578](http://www.ncbi.nlm.nih.gov/entrez/query.fcgi?cmd=Search&db=Protein&term=73809578&doptcmdl=GenPept) | dTDP-6-deoxy-L-mannose-dehydrogenase [Escherichia coli] [MASS=32617] | 118.54 | 2 | 699 1705 |  |  |
|  | [63033902](http://www.ncbi.nlm.nih.gov/entrez/query.fcgi?cmd=Search&db=Protein&term=63033902&doptcmdl=GenPept) | RmlD [Escherichia coli] [MASS=32705] | 118.54 | 2 | 699 1705 |  |  |
| 218 | [91210955](http://www.ncbi.nlm.nih.gov/entrez/query.fcgi?cmd=Search&db=Protein&term=91210955&doptcmdl=GenPept) | NAD synthetase [Escherichia coli UTI89] | 117.10 | 2 | 893 1351 |  |  |
|  | [91072529](http://www.ncbi.nlm.nih.gov/entrez/query.fcgi?cmd=Search&db=Protein&term=91072529&doptcmdl=GenPept) | NH(3)-dependent NAD(+) synthetase [Escherichia coli UTI89] | 117.10 | 2 | 893 1351 |  |  |
|  | [89108579](http://www.ncbi.nlm.nih.gov/entrez/query.fcgi?cmd=Search&db=Protein&term=89108579&doptcmdl=GenPept) | NAD synthetase, NH3/glutamine-dependent [Escherichia coli W3110] | 117.10 | 2 | 893 1351 |  |  |
|  | [83586039](http://www.ncbi.nlm.nih.gov/entrez/query.fcgi?cmd=Search&db=Protein&term=83586039&doptcmdl=GenPept) | COG0171: NAD synthase [Escherichia coli 101-1] [MASS=30666] | 117.10 | 2 | 893 1351 |  |  |
|  | [75210227](http://www.ncbi.nlm.nih.gov/entrez/query.fcgi?cmd=Search&db=Protein&term=75210227&doptcmdl=GenPept) | COG0171: NAD synthase [Escherichia coli B171] | 117.10 | 2 | 893 1351 |  |  |
| 219 | [91209762](http://www.ncbi.nlm.nih.gov/entrez/query.fcgi?cmd=Search&db=Protein&term=91209762&doptcmdl=GenPept) | succinyl-CoA synthetase subunit alpha [Escherichia coli UTI89] | 116.16 | 3 | 449 733 1586 |  |  |
|  | [91071336](http://www.ncbi.nlm.nih.gov/entrez/query.fcgi?cmd=Search&db=Protein&term=91071336&doptcmdl=GenPept) | succinyl-CoA synthetase, alpha subunit [Escherichia coli UTI89] | 116.16 | 3 | 449 733 1586 |  |  |
|  | [89107586](http://www.ncbi.nlm.nih.gov/entrez/query.fcgi?cmd=Search&db=Protein&term=89107586&doptcmdl=GenPept) | succinyl-CoA synthetase, NAD(P)-binding, alpha subunit [Escherichia coli W3110] | 116.16 | 3 | 449 733 1586 |  |  |
|  | [84027802](http://www.ncbi.nlm.nih.gov/entrez/query.fcgi?cmd=Search&db=Protein&term=84027802&doptcmdl=GenPept) | Succinyl-CoA ligase [ADP-forming] subunit alpha (Succinyl-CoA synthetase subunit alpha) (SCS-alpha) | 116.16 | 3 | 449 733 1586 |  |  |
|  | [84027801](http://www.ncbi.nlm.nih.gov/entrez/query.fcgi?cmd=Search&db=Protein&term=84027801&doptcmdl=GenPept) | Succinyl-CoA ligase [ADP-forming] subunit alpha (Succinyl-CoA synthetase subunit alpha) (SCS-alpha) | 116.16 | 3 | 449 733 1586 |  |  |
| 220 | [91073049](http://www.ncbi.nlm.nih.gov/entrez/query.fcgi?cmd=Search&db=Protein&term=91073049&doptcmdl=GenPept) | 50S ribosomal protein L25 [Escherichia coli UTI89] [MASS=14423] | 115.53 | 3 | 101 791 996 |  |  |
|  | [89109003](http://www.ncbi.nlm.nih.gov/entrez/query.fcgi?cmd=Search&db=Protein&term=89109003&doptcmdl=GenPept) | 50S ribosomal subunit protein L25 [Escherichia coli W3110] | 115.53 | 3 | 101 791 996 |  |  |
|  | [85675294](http://www.ncbi.nlm.nih.gov/entrez/query.fcgi?cmd=Search&db=Protein&term=85675294&doptcmdl=GenPept) | 50S ribosomal subunit protein L25 [Escherichia coli W3110] | 115.53 | 3 | 101 791 996 |  |  |
|  | [83754119](http://www.ncbi.nlm.nih.gov/entrez/query.fcgi?cmd=Search&db=Protein&term=83754119&doptcmdl=GenPept) | Chain V, Crystal Structure Of The Bacterial Ribosome From Escherichia Coli At 3.5 A Resolution. This File Contains The 50s Subunit Of The Second 70s Ribosome. The Entire Crystal Structure Contains Two 70s Ribosomes And Is Described In Remark 400. | 115.53 | 3 | 101 791 996 |  |  |
|  | [83754063](http://www.ncbi.nlm.nih.gov/entrez/query.fcgi?cmd=Search&db=Protein&term=83754063&doptcmdl=GenPept) | Chain V, Crystal Structure Of The Bacterial Ribosome From Escherichia Coli At 3.5 A Resolution. This File Contains The 50s Subunit Of One 70s Ribosome. The Entire Crystal Structure Contains Two 70s Ribosomes And Is Described In Remark 400. | 115.53 | 3 | 101 791 996 |  |  |
| 221 | [91211769](http://www.ncbi.nlm.nih.gov/entrez/query.fcgi?cmd=Search&db=Protein&term=91211769&doptcmdl=GenPept) | hypothetical protein YfeX [Escherichia coli UTI89] | 115.11 | 3 | 127 338 1013 |  |  |
|  | [91073343](http://www.ncbi.nlm.nih.gov/entrez/query.fcgi?cmd=Search&db=Protein&term=91073343&doptcmdl=GenPept) | hypothetical protein YfeX [Escherichia coli UTI89] [MASS=33962] | 115.11 | 3 | 127 338 1013 |  |  |
|  | [90111433](http://www.ncbi.nlm.nih.gov/entrez/query.fcgi?cmd=Search&db=Protein&term=90111433&doptcmdl=GenPept) | conserved protein [Escherichia coli str. K-12 substr. MG1655] | 115.11 | 3 | 127 338 1013 |  |  |
|  | [89109245](http://www.ncbi.nlm.nih.gov/entrez/query.fcgi?cmd=Search&db=Protein&term=89109245&doptcmdl=GenPept) | hypothetical protein [Escherichia coli W3110] | 115.11 | 3 | 127 338 1013 |  |  |
|  | [87082102](http://www.ncbi.nlm.nih.gov/entrez/query.fcgi?cmd=Search&db=Protein&term=87082102&doptcmdl=GenPept) | conserved protein [Escherichia coli str. K-12 substr. MG1655] | 115.11 | 3 | 127 338 1013 |  |  |
| 222 | [91209900](http://www.ncbi.nlm.nih.gov/entrez/query.fcgi?cmd=Search&db=Protein&term=91209900&doptcmdl=GenPept) | hypothetical protein UTI89_C0869 [Escherichia coli UTI89] | 114.64 | 2 | 1023 1197 |  |  |
|  | [91071474](http://www.ncbi.nlm.nih.gov/entrez/query.fcgi?cmd=Search&db=Protein&term=91071474&doptcmdl=GenPept) | hypothetical protein UTI89_C0869 [Escherichia coli UTI89] | 114.64 | 2 | 1023 1197 |  |  |
|  | [90101804](http://www.ncbi.nlm.nih.gov/entrez/query.fcgi?cmd=Search&db=Protein&term=90101804&doptcmdl=GenPept) | UPF0145 protein ybjQ | 114.64 | 2 | 1023 1197 |  |  |
|  | [90101803](http://www.ncbi.nlm.nih.gov/entrez/query.fcgi?cmd=Search&db=Protein&term=90101803&doptcmdl=GenPept) | UPF0145 protein ybjQ | 114.64 | 2 | 1023 1197 |  |  |
|  | [89107717](http://www.ncbi.nlm.nih.gov/entrez/query.fcgi?cmd=Search&db=Protein&term=89107717&doptcmdl=GenPept) | hypothetical protein [Escherichia coli W3110] | 114.64 | 2 | 1023 1197 |  |  |
| 223 | [91209144](http://www.ncbi.nlm.nih.gov/entrez/query.fcgi?cmd=Search&db=Protein&term=91209144&doptcmdl=GenPept) | DNA-binding transcriptional regulator FruR [Escherichia coli UTI89] | 113.20 | 3 | 342 1516 1593 |  |  |
|  | [91070718](http://www.ncbi.nlm.nih.gov/entrez/query.fcgi?cmd=Search&db=Protein&term=91070718&doptcmdl=GenPept) | transcriptional repressor of fru operon and others [Escherichia coli UTI89] | 113.20 | 3 | 342 1516 1593 |  |  |
|  | [89106963](http://www.ncbi.nlm.nih.gov/entrez/query.fcgi?cmd=Search&db=Protein&term=89106963&doptcmdl=GenPept) | DNA-binding transcriptional dual regulator [Escherichia coli W3110] | 113.20 | 3 | 342 1516 1593 |  |  |
|  | [83585198](http://www.ncbi.nlm.nih.gov/entrez/query.fcgi?cmd=Search&db=Protein&term=83585198&doptcmdl=GenPept) | COG1609: Transcriptional regulators [Escherichia coli 101-1] | 113.20 | 3 | 342 1516 1593 |  |  |
|  | [82592592](http://www.ncbi.nlm.nih.gov/entrez/query.fcgi?cmd=Search&db=Protein&term=82592592&doptcmdl=GenPept) | Fructose repressor (Catabolite repressor/activator) | 113.20 | 3 | 342 1516 1593 |  |  |
| 224 | [91210720](http://www.ncbi.nlm.nih.gov/entrez/query.fcgi?cmd=Search&db=Protein&term=91210720&doptcmdl=GenPept) | NAD-dependent malic enzyme [Escherichia coli UTI89] | 112.91 | 2 | 226 974 |  |  |
|  | [91072294](http://www.ncbi.nlm.nih.gov/entrez/query.fcgi?cmd=Search&db=Protein&term=91072294&doptcmdl=GenPept) | NAD-dependent malic enzyme [Escherichia coli UTI89] | 112.91 | 2 | 226 974 |  |  |
|  | [90111281](http://www.ncbi.nlm.nih.gov/entrez/query.fcgi?cmd=Search&db=Protein&term=90111281&doptcmdl=GenPept) | malate dehydrogenase, (decarboxylating, NAD-requiring) (malic enzyme) [Escherichia coli str. K-12 substr. MG1655] | 112.91 | 2 | 226 974 |  |  |
|  | [89108322](http://www.ncbi.nlm.nih.gov/entrez/query.fcgi?cmd=Search&db=Protein&term=89108322&doptcmdl=GenPept) | malate dehydrogenase, NAD-requiring [Escherichia coli W3110] | 112.91 | 2 | 226 974 |  |  |
|  | [87081919](http://www.ncbi.nlm.nih.gov/entrez/query.fcgi?cmd=Search&db=Protein&term=87081919&doptcmdl=GenPept) | malate dehydrogenase, (decarboxylating, NAD-requiring) (malic enzyme) [Escherichia coli str. K-12 substr. MG1655] | 112.91 | 2 | 226 974 |  |  |
| 225 | [91211812](http://www.ncbi.nlm.nih.gov/entrez/query.fcgi?cmd=Search&db=Protein&term=91211812&doptcmdl=GenPept) | thioredoxin-dependent thiol peroxidase [Escherichia coli UTI89] | 112.89 | 3 | 781 821 1388 |  |  |
|  | [91073386](http://www.ncbi.nlm.nih.gov/entrez/query.fcgi?cmd=Search&db=Protein&term=91073386&doptcmdl=GenPept) | thiol peroxidase [Escherichia coli UTI89] | 112.89 | 3 | 781 821 1388 |  |  |
|  | [89109286](http://www.ncbi.nlm.nih.gov/entrez/query.fcgi?cmd=Search&db=Protein&term=89109286&doptcmdl=GenPept) | thiol peroxidase, thioredoxin-dependent [Escherichia coli W3110] | 112.89 | 3 | 781 821 1388 |  |  |
|  | [83586871](http://www.ncbi.nlm.nih.gov/entrez/query.fcgi?cmd=Search&db=Protein&term=83586871&doptcmdl=GenPept) | COG1225: Peroxiredoxin [Escherichia coli 101-1] | 112.89 | 3 | 781 821 1388 |  |  |
|  | [83288399](http://www.ncbi.nlm.nih.gov/entrez/query.fcgi?cmd=Search&db=Protein&term=83288399&doptcmdl=GenPept) | Putative peroxiredoxin bcp (Thioredoxin reductase) (Bacterioferritin comigratory protein) | 112.89 | 3 | 781 821 1388 |  |  |
| 226 | [91075672](http://www.ncbi.nlm.nih.gov/entrez/query.fcgi?cmd=Search&db=Protein&term=91075672&doptcmdl=GenPept) | thymidine phosphorylase [Escherichia coli UTI89] [MASS=49857] | 112.76 | 2 | 159 1046 |  |  |
|  | [89111090](http://www.ncbi.nlm.nih.gov/entrez/query.fcgi?cmd=Search&db=Protein&term=89111090&doptcmdl=GenPept) | thymidine phosphorylase [Escherichia coli W3110] | 112.76 | 2 | 159 1046 |  |  |
|  | [85677121](http://www.ncbi.nlm.nih.gov/entrez/query.fcgi?cmd=Search&db=Protein&term=85677121&doptcmdl=GenPept) | thymidine phosphorylase [Escherichia coli W3110] | 112.76 | 2 | 159 1046 |  |  |
|  | [85541979](http://www.ncbi.nlm.nih.gov/entrez/query.fcgi?cmd=Search&db=Protein&term=85541979&doptcmdl=GenPept) | Thymidine phosphorylase (TdRPase) | 112.76 | 2 | 159 1046 |  |  |
|  | [85541974](http://www.ncbi.nlm.nih.gov/entrez/query.fcgi?cmd=Search&db=Protein&term=85541974&doptcmdl=GenPept) | Thymidine phosphorylase (TdRPase) | 112.76 | 2 | 159 1046 |  |  |
| 227 | [91212050](http://www.ncbi.nlm.nih.gov/entrez/query.fcgi?cmd=Search&db=Protein&term=91212050&doptcmdl=GenPept) | S-ribosylhomocysteinase [Escherichia coli UTI89] | 111.63 | 2 | 701 738 |  |  |
|  | [91073624](http://www.ncbi.nlm.nih.gov/entrez/query.fcgi?cmd=Search&db=Protein&term=91073624&doptcmdl=GenPept) | S-ribosylhomocysteinase [Escherichia coli UTI89] | 111.63 | 2 | 701 738 |  |  |
|  | [89109479](http://www.ncbi.nlm.nih.gov/entrez/query.fcgi?cmd=Search&db=Protein&term=89109479&doptcmdl=GenPept) | S-ribosylhomocysteinase [Escherichia coli W3110] | 111.63 | 2 | 701 738 |  |  |
|  | [83585147](http://www.ncbi.nlm.nih.gov/entrez/query.fcgi?cmd=Search&db=Protein&term=83585147&doptcmdl=GenPept) | COG1854: LuxS protein involved in autoinducer AI2 synthesis [Escherichia coli 101-1] | 111.63 | 2 | 701 738 |  |  |
|  | [83569952](http://www.ncbi.nlm.nih.gov/entrez/query.fcgi?cmd=Search&db=Protein&term=83569952&doptcmdl=GenPept) | COG1854: LuxS protein involved in autoinducer AI2 synthesis [Shigella dysenteriae 1012] | 111.63 | 2 | 701 738 |  |  |
| 228 | [91212597](http://www.ncbi.nlm.nih.gov/entrez/query.fcgi?cmd=Search&db=Protein&term=91212597&doptcmdl=GenPept) | phosphoglucosamine mutase [Escherichia coli UTI89] | 110.27 | 3 | 432 1283 1307 |  |  |
|  | [91074171](http://www.ncbi.nlm.nih.gov/entrez/query.fcgi?cmd=Search&db=Protein&term=91074171&doptcmdl=GenPept) | protein MrsA [Escherichia coli UTI89] | 110.27 | 3 | 432 1283 1307 |  |  |
|  | [89109939](http://www.ncbi.nlm.nih.gov/entrez/query.fcgi?cmd=Search&db=Protein&term=89109939&doptcmdl=GenPept) | phosphoglucosamine mutase [Escherichia coli W3110] | 110.27 | 3 | 432 1283 1307 |  |  |
|  | [85675970](http://www.ncbi.nlm.nih.gov/entrez/query.fcgi?cmd=Search&db=Protein&term=85675970&doptcmdl=GenPept) | phosphoglucosamine mutase [Escherichia coli W3110] | 110.27 | 3 | 432 1283 1307 |  |  |
|  | [84029254](http://www.ncbi.nlm.nih.gov/entrez/query.fcgi?cmd=Search&db=Protein&term=84029254&doptcmdl=GenPept) | Phosphoglucosamine mutase | 110.27 | 3 | 432 1283 1307 |  |  |
| 229 | [9655703](http://www.ncbi.nlm.nih.gov/entrez/query.fcgi?cmd=Search&db=Protein&term=9655703&doptcmdl=GenPept) | integration host factor, alpha subunit [Vibrio cholerae] | 109.85 | 2 | 518 1235 |  |  |
|  | [91210926](http://www.ncbi.nlm.nih.gov/entrez/query.fcgi?cmd=Search&db=Protein&term=91210926&doptcmdl=GenPept) | integration host factor subunit alpha [Escherichia coli UTI89] | 109.85 | 2 | 518 1235 |  |  |
|  | [91072500](http://www.ncbi.nlm.nih.gov/entrez/query.fcgi?cmd=Search&db=Protein&term=91072500&doptcmdl=GenPept) | integration host factor (IHF), alpha subunit [Escherichia coli UTI89] | 109.85 | 2 | 518 1235 |  |  |
|  | [89108552](http://www.ncbi.nlm.nih.gov/entrez/query.fcgi?cmd=Search&db=Protein&term=89108552&doptcmdl=GenPept) | integration host factor (IHF), DNA-binding protein, alpha subunit [Escherichia coli W3110] | 109.85 | 2 | 518 1235 |  |  |
|  | [83586015](http://www.ncbi.nlm.nih.gov/entrez/query.fcgi?cmd=Search&db=Protein&term=83586015&doptcmdl=GenPept) | COG0776: Bacterial nucleoid DNA-binding protein [Escherichia coli 101-1] | 109.85 | 2 | 518 1235 |  |  |
| 230 | [91211740](http://www.ncbi.nlm.nih.gov/entrez/query.fcgi?cmd=Search&db=Protein&term=91211740&doptcmdl=GenPept) | glutamyl-tRNA synthetase [Escherichia coli UTI89] | 109.80 | 2 | 160 1639 |  |  |
|  | [91073314](http://www.ncbi.nlm.nih.gov/entrez/query.fcgi?cmd=Search&db=Protein&term=91073314&doptcmdl=GenPept) | glutamyl-tRNA synthetase [Escherichia coli UTI89] | 109.80 | 2 | 160 1639 |  |  |
|  | [89109217](http://www.ncbi.nlm.nih.gov/entrez/query.fcgi?cmd=Search&db=Protein&term=89109217&doptcmdl=GenPept) | glutamyl-tRNA synthetase [Escherichia coli W3110] | 109.80 | 2 | 160 1639 |  |  |
|  | [83584575](http://www.ncbi.nlm.nih.gov/entrez/query.fcgi?cmd=Search&db=Protein&term=83584575&doptcmdl=GenPept) | COG0008: Glutamyl- and glutaminyl-tRNA synthetases [Escherichia coli 101-1] [MASS=53214] | 109.80 | 2 | 160 1639 |  |  |
|  | [75210186](http://www.ncbi.nlm.nih.gov/entrez/query.fcgi?cmd=Search&db=Protein&term=75210186&doptcmdl=GenPept) | COG0008: Glutamyl- and glutaminyl-tRNA synthetases [Escherichia coli B171] | 109.80 | 2 | 160 1639 |  |  |
| 231 | [91211764](http://www.ncbi.nlm.nih.gov/entrez/query.fcgi?cmd=Search&db=Protein&term=91211764&doptcmdl=GenPept) | short chain dehydrogenase [Escherichia coli UTI89] | 109.28 | 1 | 1471 |  |  |
|  | [91073338](http://www.ncbi.nlm.nih.gov/entrez/query.fcgi?cmd=Search&db=Protein&term=91073338&doptcmdl=GenPept) | oxidoreductase UcpA [Escherichia coli UTI89] [MASS=30595] | 109.28 | 1 | 1471 |  |  |
|  | [90111431](http://www.ncbi.nlm.nih.gov/entrez/query.fcgi?cmd=Search&db=Protein&term=90111431&doptcmdl=GenPept) | predicted oxidoredutase, sulfate metabolism protein [Escherichia coli str. K-12 substr. MG1655] | 109.28 | 1 | 1471 |  |  |
|  | [89109240](http://www.ncbi.nlm.nih.gov/entrez/query.fcgi?cmd=Search&db=Protein&term=89109240&doptcmdl=GenPept) | predicted oxidoredutase, sulfate metabolism protein [Escherichia coli W3110] | 109.28 | 1 | 1471 |  |  |
|  | [87082100](http://www.ncbi.nlm.nih.gov/entrez/query.fcgi?cmd=Search&db=Protein&term=87082100&doptcmdl=GenPept) | predicted oxidoredutase, sulfate metabolism protein [Escherichia coli str. K-12 substr. MG1655] | 109.28 | 1 | 1471 |  |  |
| 232 | [91209165](http://www.ncbi.nlm.nih.gov/entrez/query.fcgi?cmd=Search&db=Protein&term=91209165&doptcmdl=GenPept) | hypothetical protein UTI89_C0110 [Escherichia coli UTI89] | 108.53 | 2 | 587 1641 |  |  |
|  | [91070739](http://www.ncbi.nlm.nih.gov/entrez/query.fcgi?cmd=Search&db=Protein&term=91070739&doptcmdl=GenPept) | hypothetical protein YacF [Escherichia coli UTI89] | 108.53 | 2 | 587 1641 |  |  |
|  | [89106984](http://www.ncbi.nlm.nih.gov/entrez/query.fcgi?cmd=Search&db=Protein&term=89106984&doptcmdl=GenPept) | hypothetical protein [Escherichia coli W3110] | 108.53 | 2 | 587 1641 |  |  |
|  | [85674327](http://www.ncbi.nlm.nih.gov/entrez/query.fcgi?cmd=Search&db=Protein&term=85674327&doptcmdl=GenPept) | conserved hypothetical protein [Escherichia coli W3110] | 108.53 | 2 | 587 1641 |  |  |
|  | [83584474](http://www.ncbi.nlm.nih.gov/entrez/query.fcgi?cmd=Search&db=Protein&term=83584474&doptcmdl=GenPept) | COG4582: Uncharacterized protein conserved in bacteria [Escherichia coli 101-1] | 108.53 | 2 | 587 1641 |  |  |
| 233 | [91213699](http://www.ncbi.nlm.nih.gov/entrez/query.fcgi?cmd=Search&db=Protein&term=91213699&doptcmdl=GenPept) | entericidin B membrane lipoprotein [Escherichia coli UTI89] | 108.35 | 1 | 746 |  |  |
|  | [91075273](http://www.ncbi.nlm.nih.gov/entrez/query.fcgi?cmd=Search&db=Protein&term=91075273&doptcmdl=GenPept) | putative toxin of osmotically regulated toxin-antitoxin system associated with programmed cell death [Escherichia coli UTI89] | 108.35 | 1 | 746 |  |  |
|  | [89110870](http://www.ncbi.nlm.nih.gov/entrez/query.fcgi?cmd=Search&db=Protein&term=89110870&doptcmdl=GenPept) | entericidin B membrane lipoprotein [Escherichia coli W3110] | 108.35 | 1 | 746 |  |  |
|  | [85676901](http://www.ncbi.nlm.nih.gov/entrez/query.fcgi?cmd=Search&db=Protein&term=85676901&doptcmdl=GenPept) | entericidin B membrane lipoprotein [Escherichia coli W3110] | 108.35 | 1 | 746 |  |  |
|  | [83308963](http://www.ncbi.nlm.nih.gov/entrez/query.fcgi?cmd=Search&db=Protein&term=83308963&doptcmdl=GenPept) | Entericidin B precursor | 108.35 | 1 | 746 |  |  |
| 234 | [96788](http://www.ncbi.nlm.nih.gov/entrez/query.fcgi?cmd=Search&db=Protein&term=96788&doptcmdl=GenPept) | probable (3R)-hydroxymyristoyl-[acyl carrier protein] dehydratase (EC 4.2.1.-) - Salmonella typhimurium (fragment) | 108.31 | 2 | 1510 1573 |  |  |
|  | [91209250](http://www.ncbi.nlm.nih.gov/entrez/query.fcgi?cmd=Search&db=Protein&term=91209250&doptcmdl=GenPept) | (3R)-hydroxymyristoyl-(acyl carrier protein) dehydratase [Escherichia coli UTI89] | 108.31 | 2 | 1510 1573 |  |  |
|  | [91206651](http://www.ncbi.nlm.nih.gov/entrez/query.fcgi?cmd=Search&db=Protein&term=91206651&doptcmdl=GenPept) | (3R)-hydroxymyristoyl-[acyl-carrier-protein] dehydratase ((3R)-hydroxymyristoyl ACP dehydrase) | 108.31 | 2 | 1510 1573 |  |  |
|  | [91206650](http://www.ncbi.nlm.nih.gov/entrez/query.fcgi?cmd=Search&db=Protein&term=91206650&doptcmdl=GenPept) | (3R)-hydroxymyristoyl-[acyl-carrier-protein] dehydratase ((3R)-hydroxymyristoyl ACP dehydrase) | 108.31 | 2 | 1510 1573 |  |  |
|  | [91206649](http://www.ncbi.nlm.nih.gov/entrez/query.fcgi?cmd=Search&db=Protein&term=91206649&doptcmdl=GenPept) | (3R)-hydroxymyristoyl-[acyl-carrier-protein] dehydratase ((3R)-hydroxymyristoyl ACP dehydrase) | 108.31 | 2 | 1510 1573 |  |  |
| 235 | [91211327](http://www.ncbi.nlm.nih.gov/entrez/query.fcgi?cmd=Search&db=Protein&term=91211327&doptcmdl=GenPept) | UTP--glucose-1-phosphate uridylyltransferase subunit GalF [Escherichia coli UTI89] | 107.25 | 2 | 1575 1601 |  |  |
|  | [91072901](http://www.ncbi.nlm.nih.gov/entrez/query.fcgi?cmd=Search&db=Protein&term=91072901&doptcmdl=GenPept) | putative UTP--glucose-1-phosphate uridylyltransferase [Escherichia coli UTI89] [MASS=33302] | 107.25 | 2 | 1575 1601 |  |  |
|  | [90183189](http://www.ncbi.nlm.nih.gov/entrez/query.fcgi?cmd=Search&db=Protein&term=90183189&doptcmdl=GenPept) | UTP--glucose-1-phosphate uridylyltransferase (UDP-glucose pyrophosphorylase) (UDPGP) (Alpha-D-glucosyl-1-phosphate uridylyltransferase) (Uridine diphosphoglucose pyrophosphorylase) | 107.25 | 2 | 1575 1601 |  |  |
|  | [90183188](http://www.ncbi.nlm.nih.gov/entrez/query.fcgi?cmd=Search&db=Protein&term=90183188&doptcmdl=GenPept) | UTP--glucose-1-phosphate uridylyltransferase (UDP-glucose pyrophosphorylase) (UDPGP) (Alpha-D-glucosyl-1-phosphate uridylyltransferase) (Uridine diphosphoglucose pyrophosphorylase) | 107.25 | 2 | 1575 1601 |  |  |
|  | [89108862](http://www.ncbi.nlm.nih.gov/entrez/query.fcgi?cmd=Search&db=Protein&term=89108862&doptcmdl=GenPept) | predicted subunit with GalU [Escherichia coli W3110] | 107.25 | 2 | 1575 1601 |  |  |
| 236 | [91213157](http://www.ncbi.nlm.nih.gov/entrez/query.fcgi?cmd=Search&db=Protein&term=91213157&doptcmdl=GenPept) | deoxyuridine 5'-triphosphate nucleotidohydrolase [Escherichia coli UTI89] | 107.18 | 1 | 1465 |  |  |
|  | [91206612](http://www.ncbi.nlm.nih.gov/entrez/query.fcgi?cmd=Search&db=Protein&term=91206612&doptcmdl=GenPept) | Deoxyuridine 5'-triphosphate nucleotidohydrolase (dUTPase) (dUTP pyrophosphatase) | 107.18 | 1 | 1465 |  |  |
|  | [91206611](http://www.ncbi.nlm.nih.gov/entrez/query.fcgi?cmd=Search&db=Protein&term=91206611&doptcmdl=GenPept) | Deoxyuridine 5'-triphosphate nucleotidohydrolase (dUTPase) (dUTP pyrophosphatase) | 107.18 | 1 | 1465 |  |  |
|  | [91074731](http://www.ncbi.nlm.nih.gov/entrez/query.fcgi?cmd=Search&db=Protein&term=91074731&doptcmdl=GenPept) | deoxyuridine 5'-triphosphate nucleotidohydrolase [Escherichia coli UTI89] | 107.18 | 1 | 1465 |  |  |
|  | [89110371](http://www.ncbi.nlm.nih.gov/entrez/query.fcgi?cmd=Search&db=Protein&term=89110371&doptcmdl=GenPept) | deoxyuridinetriphosphatase [Escherichia coli W3110] | 107.18 | 1 | 1465 |  |  |
| 237 | [91209072](http://www.ncbi.nlm.nih.gov/entrez/query.fcgi?cmd=Search&db=Protein&term=91209072&doptcmdl=GenPept) | chaperone protein DnaJ [Escherichia coli UTI89] | 105.66 | 2 | 982 1305 |  |  |
|  | [91070646](http://www.ncbi.nlm.nih.gov/entrez/query.fcgi?cmd=Search&db=Protein&term=91070646&doptcmdl=GenPept) | chaperone with DnaK; heat shock protein [Escherichia coli UTI89] | 105.66 | 2 | 982 1305 |  |  |
|  | [89106899](http://www.ncbi.nlm.nih.gov/entrez/query.fcgi?cmd=Search&db=Protein&term=89106899&doptcmdl=GenPept) | chaperone Hsp40, co-chaperone with DnaK [Escherichia coli W3110] | 105.66 | 2 | 982 1305 |  |  |
|  | [83585322](http://www.ncbi.nlm.nih.gov/entrez/query.fcgi?cmd=Search&db=Protein&term=83585322&doptcmdl=GenPept) | COG0484: DnaJ-class molecular chaperone with C-terminal Zn finger domain [Escherichia coli 101-1] [MASS=41025] | 105.66 | 2 | 982 1305 |  |  |
|  | [75209619](http://www.ncbi.nlm.nih.gov/entrez/query.fcgi?cmd=Search&db=Protein&term=75209619&doptcmdl=GenPept) | COG0484: DnaJ-class molecular chaperone with C-terminal Zn finger domain [Escherichia coli B171] [MASS=41074] | 105.66 | 2 | 982 1305 |  |  |
| 238 | [91209703](http://www.ncbi.nlm.nih.gov/entrez/query.fcgi?cmd=Search&db=Protein&term=91209703&doptcmdl=GenPept) | putative ATP-binding protein in pho regulon [Escherichia coli UTI89] | 104.75 | 2 | 581 607 |  |  |
|  | [91071277](http://www.ncbi.nlm.nih.gov/entrez/query.fcgi?cmd=Search&db=Protein&term=91071277&doptcmdl=GenPept) | putative ATP-binding protein in pho regulon [Escherichia coli UTI89] | 104.75 | 2 | 581 607 |  |  |
|  | [89107529](http://www.ncbi.nlm.nih.gov/entrez/query.fcgi?cmd=Search&db=Protein&term=89107529&doptcmdl=GenPept) | hypothetical protein with nucleoside triphosphate hydrolase domain [Escherichia coli W3110] | 104.75 | 2 | 581 607 |  |  |
|  | [83584742](http://www.ncbi.nlm.nih.gov/entrez/query.fcgi?cmd=Search&db=Protein&term=83584742&doptcmdl=GenPept) | COG1702: Phosphate starvation-inducible protein PhoH, predicted ATPase [Escherichia coli 101-1] | 104.75 | 2 | 581 607 |  |  |
|  | [83569120](http://www.ncbi.nlm.nih.gov/entrez/query.fcgi?cmd=Search&db=Protein&term=83569120&doptcmdl=GenPept) | COG1702: Phosphate starvation-inducible protein PhoH, predicted ATPase [Shigella dysenteriae 1012] | 104.75 | 2 | 581 607 |  |  |

| **Protein IDs*, cont.*** | | | | | | | |
| --- | --- | --- | --- | --- | --- | --- | --- |
| *Grp Nr.* | *Accession Number* | *Protein Name* | *Protein Score* | *Unique PSMs* | *PSM Serial Nrs.* | *Other Grp.* | *Score (other)* |
| 239 | [91212879](http://www.ncbi.nlm.nih.gov/entrez/query.fcgi?cmd=Search&db=Protein&term=91212879&doptcmdl=GenPept) | osmolarity response regulator [Escherichia coli UTI89] | 103.85 | 2 | 1223 1482 |  |  |
|  | [91074453](http://www.ncbi.nlm.nih.gov/entrez/query.fcgi?cmd=Search&db=Protein&term=91074453&doptcmdl=GenPept) | transcriptional regulatory protein OmpR [Escherichia coli UTI89] | 103.85 | 2 | 1223 1482 |  |  |
|  | [89110605](http://www.ncbi.nlm.nih.gov/entrez/query.fcgi?cmd=Search&db=Protein&term=89110605&doptcmdl=GenPept) | DNA-binding response regulator in two-component regulatory system with EnvZ [Escherichia coli W3110] | 103.85 | 2 | 1223 1482 |  |  |
|  | [85692721](http://www.ncbi.nlm.nih.gov/entrez/query.fcgi?cmd=Search&db=Protein&term=85692721&doptcmdl=GenPept) | Transcriptional regulatory protein ompR | 103.85 | 2 | 1223 1482 |  |  |
|  | [85692720](http://www.ncbi.nlm.nih.gov/entrez/query.fcgi?cmd=Search&db=Protein&term=85692720&doptcmdl=GenPept) | Transcriptional regulatory protein ompR | 103.85 | 2 | 1223 1482 |  |  |
| 240 | [91212664](http://www.ncbi.nlm.nih.gov/entrez/query.fcgi?cmd=Search&db=Protein&term=91212664&doptcmdl=GenPept) | csrA activity inhibitor TldD [Escherichia coli UTI89] | 103.51 | 2 | 848 1699 |  |  |
|  | [91074238](http://www.ncbi.nlm.nih.gov/entrez/query.fcgi?cmd=Search&db=Protein&term=91074238&doptcmdl=GenPept) | csrA activity inhibitor TldD [Escherichia coli UTI89] | 103.51 | 2 | 848 1699 |  |  |
|  | [89110006](http://www.ncbi.nlm.nih.gov/entrez/query.fcgi?cmd=Search&db=Protein&term=89110006&doptcmdl=GenPept) | predicted peptidase [Escherichia coli W3110] | 103.51 | 2 | 848 1699 |  |  |
|  | [85676037](http://www.ncbi.nlm.nih.gov/entrez/query.fcgi?cmd=Search&db=Protein&term=85676037&doptcmdl=GenPept) | predicted peptidase [Escherichia coli W3110] | 103.51 | 2 | 848 1699 |  |  |
|  | [84028915](http://www.ncbi.nlm.nih.gov/entrez/query.fcgi?cmd=Search&db=Protein&term=84028915&doptcmdl=GenPept) | Protein tldD | 103.51 | 2 | 848 1699 |  |  |
| 241 | [91212721](http://www.ncbi.nlm.nih.gov/entrez/query.fcgi?cmd=Search&db=Protein&term=91212721&doptcmdl=GenPept) | 50S ribosomal protein L17 [Escherichia coli UTI89] | 103.11 | 3 | 777 812 1088 |  |  |
|  | [91074295](http://www.ncbi.nlm.nih.gov/entrez/query.fcgi?cmd=Search&db=Protein&term=91074295&doptcmdl=GenPept) | 50S ribosomal subunit protein L17 [Escherichia coli UTI89] | 103.11 | 3 | 777 812 1088 |  |  |
|  | [89110716](http://www.ncbi.nlm.nih.gov/entrez/query.fcgi?cmd=Search&db=Protein&term=89110716&doptcmdl=GenPept) | 50S ribosomal subunit protein L17 [Escherichia coli W3110] | 103.11 | 3 | 777 812 1088 |  |  |
|  | [85676747](http://www.ncbi.nlm.nih.gov/entrez/query.fcgi?cmd=Search&db=Protein&term=85676747&doptcmdl=GenPept) | 50S ribosomal subunit protein L17 [Escherichia coli W3110] | 103.11 | 3 | 777 812 1088 |  |  |
|  | [84027991](http://www.ncbi.nlm.nih.gov/entrez/query.fcgi?cmd=Search&db=Protein&term=84027991&doptcmdl=GenPept) | 50S ribosomal protein L17 | 103.11 | 3 | 777 812 1088 |  |  |
| 242 | [91209783](http://www.ncbi.nlm.nih.gov/entrez/query.fcgi?cmd=Search&db=Protein&term=91209783&doptcmdl=GenPept) | phosphoglycerate mutase 1 [Escherichia coli UTI89] | 102.28 | 2 | 1459 1590 |  |  |
|  | [91206785](http://www.ncbi.nlm.nih.gov/entrez/query.fcgi?cmd=Search&db=Protein&term=91206785&doptcmdl=GenPept) | 2,3-bisphosphoglycerate-dependent phosphoglycerate mutase (Phosphoglyceromutase) (PGAM) (BPG-dependent PGAM) (dPGM) | 102.28 | 2 | 1459 1590 |  |  |
|  | [91206783](http://www.ncbi.nlm.nih.gov/entrez/query.fcgi?cmd=Search&db=Protein&term=91206783&doptcmdl=GenPept) | 2,3-bisphosphoglycerate-dependent phosphoglycerate mutase (Phosphoglyceromutase) (PGAM) (BPG-dependent PGAM) (dPGM) | 102.28 | 2 | 1459 1590 |  |  |
|  | [91071357](http://www.ncbi.nlm.nih.gov/entrez/query.fcgi?cmd=Search&db=Protein&term=91071357&doptcmdl=GenPept) | phosphoglycerate mutase 1 [Escherichia coli UTI89] [MASS=29222] | 102.28 | 2 | 1459 1590 |  |  |
|  | [89107606](http://www.ncbi.nlm.nih.gov/entrez/query.fcgi?cmd=Search&db=Protein&term=89107606&doptcmdl=GenPept) | phosphoglyceromutase 1 [Escherichia coli W3110] | 102.28 | 2 | 1459 1590 |  |  |
| 243 | [2781033](http://www.ncbi.nlm.nih.gov/entrez/query.fcgi?cmd=Search&db=Protein&term=2781033&doptcmdl=GenPept) | Chain B, Flavodoxins That Are Required For Enzyme Activation: The Structure Of Oxidized Flavodoxin From Escherichia Coli At 1.8 Angstroms Resolution. | 101.09 | 1 | 1362 |  |  |
|  | [2781032](http://www.ncbi.nlm.nih.gov/entrez/query.fcgi?cmd=Search&db=Protein&term=2781032&doptcmdl=GenPept) | Chain A, Flavodoxins That Are Required For Enzyme Activation: The Structure Of Oxidized Flavodoxin From Escherichia Coli At 1.8 Angstroms Resolution. | 101.09 | 1 | 1362 |  |  |
|  | [157829885](http://www.ncbi.nlm.nih.gov/entrez/query.fcgi?cmd=Search&db=Protein&term=157829885&doptcmdl=GenPept) | Chain A, E. Coli Flavodoxin At 2.6 Angstroms Resolution [MASS=19606] | 101.09 | 1 | 1362 |  |  |
| 244 | [91213122](http://www.ncbi.nlm.nih.gov/entrez/query.fcgi?cmd=Search&db=Protein&term=91213122&doptcmdl=GenPept) | NAD(P)H-dependent glycerol-3-phosphate dehydrogenase [Escherichia coli UTI89] | 100.22 | 2 | 1001 1614 |  |  |
|  | [91074696](http://www.ncbi.nlm.nih.gov/entrez/query.fcgi?cmd=Search&db=Protein&term=91074696&doptcmdl=GenPept) | glycerol-3-phosphate dehydrogenase (NAD+) [Escherichia coli UTI89] | 100.22 | 2 | 1001 1614 |  |  |
|  | [89110403](http://www.ncbi.nlm.nih.gov/entrez/query.fcgi?cmd=Search&db=Protein&term=89110403&doptcmdl=GenPept) | glycerol-3-phosphate dehydrogenase (NAD+) [Escherichia coli W3110] | 100.22 | 2 | 1001 1614 |  |  |
|  | [85676434](http://www.ncbi.nlm.nih.gov/entrez/query.fcgi?cmd=Search&db=Protein&term=85676434&doptcmdl=GenPept) | glycerol-3-phosphate dehydrogenase (NAD+) [Escherichia coli W3110] | 100.22 | 2 | 1001 1614 |  |  |
|  | [83588538](http://www.ncbi.nlm.nih.gov/entrez/query.fcgi?cmd=Search&db=Protein&term=83588538&doptcmdl=GenPept) | COG0240: Glycerol-3-phosphate dehydrogenase [Escherichia coli 101-1] | 100.22 | 2 | 1001 1614 |  |  |
| 245 | [EFP_SHIFL](http://us.expasy.org/uniprot/EFP_SHIFL) | Elongation factor P (EF-P) | 99.43 | 2 | 399 530 |  |  |
|  | [EFP_SALTY](http://us.expasy.org/uniprot/EFP_SALTY) | Elongation factor P (EF-P) | 99.43 | 2 | 399 530 |  |  |
|  | [EFP_SALTI](http://us.expasy.org/uniprot/EFP_SALTI) | Elongation factor P (EF-P) | 99.43 | 2 | 399 530 |  |  |
|  | [EFP_SALPA](http://us.expasy.org/uniprot/EFP_SALPA) | Elongation factor P (EF-P) | 99.43 | 2 | 399 530 |  |  |
|  | [91213697](http://www.ncbi.nlm.nih.gov/entrez/query.fcgi?cmd=Search&db=Protein&term=91213697&doptcmdl=GenPept) | elongation factor P [Escherichia coli UTI89] | 66.58 | 1 | 399 |  |  |
| 246 | [9955170](http://www.ncbi.nlm.nih.gov/entrez/query.fcgi?cmd=Search&db=Protein&term=9955170&doptcmdl=GenPept) | Chain B, Crystal Structure Of E Coli Enoyl Reductase-Nad+-Triclosan Complex | 99.32 | 2 | 455 1554 |  |  |
|  | [9955169](http://www.ncbi.nlm.nih.gov/entrez/query.fcgi?cmd=Search&db=Protein&term=9955169&doptcmdl=GenPept) | Chain A, Crystal Structure Of E Coli Enoyl Reductase-Nad+-Triclosan Complex | 99.32 | 2 | 455 1554 |  |  |
|  | [91210584](http://www.ncbi.nlm.nih.gov/entrez/query.fcgi?cmd=Search&db=Protein&term=91210584&doptcmdl=GenPept) | enoyl-(acyl carrier protein) reductase [Escherichia coli UTI89] | 99.32 | 2 | 455 1554 |  |  |
|  | [91072158](http://www.ncbi.nlm.nih.gov/entrez/query.fcgi?cmd=Search&db=Protein&term=91072158&doptcmdl=GenPept) | enoyl-[acyl-carrier-protein] reductase (NADH) [Escherichia coli UTI89] | 99.32 | 2 | 455 1554 |  |  |
|  | [89108134](http://www.ncbi.nlm.nih.gov/entrez/query.fcgi?cmd=Search&db=Protein&term=89108134&doptcmdl=GenPept) | enoyl-[acyl-carrier-protein] reductase, NADH-dependent [Escherichia coli W3110] | 99.32 | 2 | 455 1554 |  |  |
| 247 | [91210825](http://www.ncbi.nlm.nih.gov/entrez/query.fcgi?cmd=Search&db=Protein&term=91210825&doptcmdl=GenPept) | hypothetical protein YdgA [Escherichia coli UTI89] | 97.87 | 2 | 360 874 |  |  |
|  | [91072399](http://www.ncbi.nlm.nih.gov/entrez/query.fcgi?cmd=Search&db=Protein&term=91072399&doptcmdl=GenPept) | hypothetical protein YdgA [Escherichia coli UTI89] | 97.87 | 2 | 360 874 |  |  |
|  | [89108455](http://www.ncbi.nlm.nih.gov/entrez/query.fcgi?cmd=Search&db=Protein&term=89108455&doptcmdl=GenPept) | hypothetical protein [Escherichia coli W3110] | 97.87 | 2 | 360 874 |  |  |
|  | [83587949](http://www.ncbi.nlm.nih.gov/entrez/query.fcgi?cmd=Search&db=Protein&term=83587949&doptcmdl=GenPept) | COG5339: Uncharacterized protein conserved in bacteria [Escherichia coli 101-1] | 97.87 | 2 | 360 874 |  |  |
|  | [83571004](http://www.ncbi.nlm.nih.gov/entrez/query.fcgi?cmd=Search&db=Protein&term=83571004&doptcmdl=GenPept) | COG5339: Uncharacterized protein conserved in bacteria [Shigella dysenteriae 1012] [MASS=54688] | 97.87 | 2 | 360 874 |  |  |
| 248 | [91213161](http://www.ncbi.nlm.nih.gov/entrez/query.fcgi?cmd=Search&db=Protein&term=91213161&doptcmdl=GenPept) | hypothetical protein UTI89_C4188 [Escherichia coli UTI89] | 97.19 | 2 | 584 887 |  |  |
|  | [91074735](http://www.ncbi.nlm.nih.gov/entrez/query.fcgi?cmd=Search&db=Protein&term=91074735&doptcmdl=GenPept) | putative alpha helix protein [Escherichia coli UTI89] | 97.19 | 2 | 584 887 |  |  |
|  | [89110368](http://www.ncbi.nlm.nih.gov/entrez/query.fcgi?cmd=Search&db=Protein&term=89110368&doptcmdl=GenPept) | hypothetical protein [Escherichia coli W3110] | 97.19 | 2 | 584 887 |  |  |
|  | [85676399](http://www.ncbi.nlm.nih.gov/entrez/query.fcgi?cmd=Search&db=Protein&term=85676399&doptcmdl=GenPept) | conserved hypothetical protein [Escherichia coli W3110] | 97.19 | 2 | 584 887 |  |  |
|  | [83588509](http://www.ncbi.nlm.nih.gov/entrez/query.fcgi?cmd=Search&db=Protein&term=83588509&doptcmdl=GenPept) | COG1561: Uncharacterized stress-induced protein [Escherichia coli 101-1] | 97.19 | 2 | 584 887 |  |  |
| 249 | [91212670](http://www.ncbi.nlm.nih.gov/entrez/query.fcgi?cmd=Search&db=Protein&term=91212670&doptcmdl=GenPept) | regulator of FtsI [Escherichia coli UTI89] | 97.08 | 2 | 891 1404 |  |  |
|  | [91074244](http://www.ncbi.nlm.nih.gov/entrez/query.fcgi?cmd=Search&db=Protein&term=91074244&doptcmdl=GenPept) | regulator of FtsI [Escherichia coli UTI89] [MASS=39541] | 97.08 | 2 | 891 1404 |  |  |
|  | [90111564](http://www.ncbi.nlm.nih.gov/entrez/query.fcgi?cmd=Search&db=Protein&term=90111564&doptcmdl=GenPept) | cell wall structural complex MreBCD, actin-like component MreB [Escherichia coli str. K-12 substr. MG1655] | 97.08 | 2 | 891 1404 |  |  |
|  | [90103506](http://www.ncbi.nlm.nih.gov/entrez/query.fcgi?cmd=Search&db=Protein&term=90103506&doptcmdl=GenPept) | Rod shape-determining protein mreB | 97.08 | 2 | 891 1404 |  |  |
|  | [90103505](http://www.ncbi.nlm.nih.gov/entrez/query.fcgi?cmd=Search&db=Protein&term=90103505&doptcmdl=GenPept) | Rod shape-determining protein mreB | 97.08 | 2 | 891 1404 |  |  |
| 250 | [91212599](http://www.ncbi.nlm.nih.gov/entrez/query.fcgi?cmd=Search&db=Protein&term=91212599&doptcmdl=GenPept) | ATP-dependent metalloprotease [Escherichia coli UTI89] | 96.48 | 2 | 144 1656 |  |  |
|  | [91074173](http://www.ncbi.nlm.nih.gov/entrez/query.fcgi?cmd=Search&db=Protein&term=91074173&doptcmdl=GenPept) | ATP-binding protein [Escherichia coli UTI89] | 96.48 | 2 | 144 1656 |  |  |
|  | [89109941](http://www.ncbi.nlm.nih.gov/entrez/query.fcgi?cmd=Search&db=Protein&term=89109941&doptcmdl=GenPept) | protease, ATP-dependent zinc-metallo [Escherichia coli W3110] | 96.48 | 2 | 144 1656 |  |  |
|  | [85675972](http://www.ncbi.nlm.nih.gov/entrez/query.fcgi?cmd=Search&db=Protein&term=85675972&doptcmdl=GenPept) | protease, ATP-dependent zinc-metallo [Escherichia coli W3110] | 96.48 | 2 | 144 1656 |  |  |
|  | [83585817](http://www.ncbi.nlm.nih.gov/entrez/query.fcgi?cmd=Search&db=Protein&term=83585817&doptcmdl=GenPept) | COG0465: ATP-dependent Zn proteases [Escherichia coli 101-1] [MASS=53892] | 96.48 | 2 | 144 1656 |  |  |
| 251 | [91212586](http://www.ncbi.nlm.nih.gov/entrez/query.fcgi?cmd=Search&db=Protein&term=91212586&doptcmdl=GenPept) | 30S ribosomal protein S15 [Escherichia coli UTI89] | 96.20 | 2 | 1410 1517 |  |  |
|  | [91074160](http://www.ncbi.nlm.nih.gov/entrez/query.fcgi?cmd=Search&db=Protein&term=91074160&doptcmdl=GenPept) | 30S ribosomal subunit protein S15 [Escherichia coli UTI89] | 96.20 | 2 | 1410 1517 |  |  |
|  | [89109930](http://www.ncbi.nlm.nih.gov/entrez/query.fcgi?cmd=Search&db=Protein&term=89109930&doptcmdl=GenPept) | 30S ribosomal subunit protein S15 [Escherichia coli W3110] | 96.20 | 2 | 1410 1517 |  |  |
|  | [85701261](http://www.ncbi.nlm.nih.gov/entrez/query.fcgi?cmd=Search&db=Protein&term=85701261&doptcmdl=GenPept) | 30S ribosomal protein S15 | 96.20 | 2 | 1410 1517 |  |  |
|  | [85701260](http://www.ncbi.nlm.nih.gov/entrez/query.fcgi?cmd=Search&db=Protein&term=85701260&doptcmdl=GenPept) | 30S ribosomal protein S15 | 96.20 | 2 | 1410 1517 |  |  |
| 252 | [91212026](http://www.ncbi.nlm.nih.gov/entrez/query.fcgi?cmd=Search&db=Protein&term=91212026&doptcmdl=GenPept) | DNA binding protein, nucleoid-associated [Escherichia coli UTI89] | 95.96 | 2 | 19 499 |  |  |
|  | [91073600](http://www.ncbi.nlm.nih.gov/entrez/query.fcgi?cmd=Search&db=Protein&term=91073600&doptcmdl=GenPept) | DNA-binding protein StpA [Escherichia coli UTI89] | 95.96 | 2 | 19 499 |  |  |
|  | [89109462](http://www.ncbi.nlm.nih.gov/entrez/query.fcgi?cmd=Search&db=Protein&term=89109462&doptcmdl=GenPept) | DNA binding protein, nucleoid-associated [Escherichia coli W3110] | 95.96 | 2 | 19 499 |  |  |
|  | [83585164](http://www.ncbi.nlm.nih.gov/entrez/query.fcgi?cmd=Search&db=Protein&term=83585164&doptcmdl=GenPept) | COG2916: DNA-binding protein H-NS [Escherichia coli 101-1] [MASS=13518] | 95.96 | 2 | 19 499 |  |  |
|  | [82778038](http://www.ncbi.nlm.nih.gov/entrez/query.fcgi?cmd=Search&db=Protein&term=82778038&doptcmdl=GenPept) | DNA binding protein, nucleoid-associated [Shigella dysenteriae Sd197] | 95.96 | 2 | 19 499 |  |  |
| 253 | [YEGP_SHIFL](http://us.expasy.org/uniprot/YEGP_SHIFL) | Hypothetical UPF0339 protein yegP | 95.14 | 2 | 1067 1144 |  |  |
|  | [YEGP_ECOLI](http://us.expasy.org/uniprot/YEGP_ECOLI) | Hypothetical UPF0339 protein yegP | 95.14 | 2 | 1067 1144 |  |  |
|  | [YEGP_ECOL6](http://us.expasy.org/uniprot/YEGP_ECOL6) | Hypothetical UPF0339 protein yegP | 95.14 | 2 | 1067 1144 |  |  |
|  | [YEGP_ECO57](http://us.expasy.org/uniprot/YEGP_ECO57) | Hypothetical UPF0339 protein yegP | 95.14 | 2 | 1067 1144 |  |  |
|  | [91211368](http://www.ncbi.nlm.nih.gov/entrez/query.fcgi?cmd=Search&db=Protein&term=91211368&doptcmdl=GenPept) | hypothetical protein YegP [Escherichia coli UTI89] | 58.49 | 1 | 1067 |  |  |
| 254 | [9256992](http://www.ncbi.nlm.nih.gov/entrez/query.fcgi?cmd=Search&db=Protein&term=9256992&doptcmdl=GenPept) | Chain A, Core Structure Of The Outer Membrane Lipoprotein From Escherichia Coli At 1.9 Angstrom Resolution [MASS=6154] | 94.56 | 1 | 817 |  |  |
|  | [92058700](http://www.ncbi.nlm.nih.gov/entrez/query.fcgi?cmd=Search&db=Protein&term=92058700&doptcmdl=GenPept) | Major outer membrane lipoprotein precursor (Murein-lipoprotein) | 94.56 | 1 | 817 |  |  |
|  | [91210890](http://www.ncbi.nlm.nih.gov/entrez/query.fcgi?cmd=Search&db=Protein&term=91210890&doptcmdl=GenPept) | murein lipoprotein [Escherichia coli UTI89] | 94.56 | 1 | 817 |  |  |
|  | [91072464](http://www.ncbi.nlm.nih.gov/entrez/query.fcgi?cmd=Search&db=Protein&term=91072464&doptcmdl=GenPept) | murein lipoprotein [Escherichia coli UTI89] | 94.56 | 1 | 817 |  |  |
|  | [89108517](http://www.ncbi.nlm.nih.gov/entrez/query.fcgi?cmd=Search&db=Protein&term=89108517&doptcmdl=GenPept) | murein lipoprotein [Escherichia coli W3110] | 94.56 | 1 | 817 |  |  |
| 255 | [91212568](http://www.ncbi.nlm.nih.gov/entrez/query.fcgi?cmd=Search&db=Protein&term=91212568&doptcmdl=GenPept) | hypothetical protein UTI89_C3577 [Escherichia coli UTI89] | 94.26 | 2 | 854 920 |  |  |
|  | [91074142](http://www.ncbi.nlm.nih.gov/entrez/query.fcgi?cmd=Search&db=Protein&term=91074142&doptcmdl=GenPept) | putative periplasmic protein [Escherichia coli UTI89] | 94.26 | 2 | 854 920 |  |  |
|  | [89109915](http://www.ncbi.nlm.nih.gov/entrez/query.fcgi?cmd=Search&db=Protein&term=89109915&doptcmdl=GenPept) | hypothetical protein [Escherichia coli W3110] | 94.26 | 2 | 854 920 |  |  |
|  | [85675946](http://www.ncbi.nlm.nih.gov/entrez/query.fcgi?cmd=Search&db=Protein&term=85675946&doptcmdl=GenPept) | hypothetical protein [Escherichia coli W3110] | 94.26 | 2 | 854 920 |  |  |
|  | [83585791](http://www.ncbi.nlm.nih.gov/entrez/query.fcgi?cmd=Search&db=Protein&term=83585791&doptcmdl=GenPept) | COG2823: Predicted periplasmic or secreted lipoprotein [Escherichia coli 101-1] | 94.26 | 2 | 854 920 |  |  |
| 256 | [91212858](http://www.ncbi.nlm.nih.gov/entrez/query.fcgi?cmd=Search&db=Protein&term=91212858&doptcmdl=GenPept) | ribulose-phosphate 3-epimerase [Escherichia coli UTI89] | 94.20 | 2 | 335 1621 |  |  |
|  | [91074432](http://www.ncbi.nlm.nih.gov/entrez/query.fcgi?cmd=Search&db=Protein&term=91074432&doptcmdl=GenPept) | D-ribulose-5-phosphate 3-epimerase [Escherichia coli UTI89] | 94.20 | 2 | 335 1621 |  |  |
|  | [89110624](http://www.ncbi.nlm.nih.gov/entrez/query.fcgi?cmd=Search&db=Protein&term=89110624&doptcmdl=GenPept) | D-ribulose-5-phosphate 3-epimerase [Escherichia coli W3110] | 94.20 | 2 | 335 1621 |  |  |
|  | [85676655](http://www.ncbi.nlm.nih.gov/entrez/query.fcgi?cmd=Search&db=Protein&term=85676655&doptcmdl=GenPept) | D-ribulose-5-phosphate 3-epimerase [Escherichia coli W3110] | 94.20 | 2 | 335 1621 |  |  |
|  | [84028158](http://www.ncbi.nlm.nih.gov/entrez/query.fcgi?cmd=Search&db=Protein&term=84028158&doptcmdl=GenPept) | Ribulose-phosphate 3-epimerase (Pentose-5-phosphate 3-epimerase) (PPE) (R5P3E) | 94.20 | 2 | 335 1621 |  |  |
| 257 | [91209162](http://www.ncbi.nlm.nih.gov/entrez/query.fcgi?cmd=Search&db=Protein&term=91209162&doptcmdl=GenPept) | preprotein translocase subunit SecA [Escherichia coli UTI89] | 94.09 | 2 | 136 1615 |  |  |
|  | [91070736](http://www.ncbi.nlm.nih.gov/entrez/query.fcgi?cmd=Search&db=Protein&term=91070736&doptcmdl=GenPept) | preprotein translocase; secretion protein [Escherichia coli UTI89] | 94.09 | 2 | 136 1615 |  |  |
|  | [89106981](http://www.ncbi.nlm.nih.gov/entrez/query.fcgi?cmd=Search&db=Protein&term=89106981&doptcmdl=GenPept) | preprotein translocase subunit, ATPase that targets protein precursors to the SecYE core translocon [Escherichia coli W3110] | 94.09 | 2 | 136 1615 |  |  |
|  | [85674325](http://www.ncbi.nlm.nih.gov/entrez/query.fcgi?cmd=Search&db=Protein&term=85674325&doptcmdl=GenPept) | preprotein translocase subunit, ATPase that targets protein precursors to the SecYE core translocon [Escherichia coli W3110] | 94.09 | 2 | 136 1615 |  |  |
|  | [82542702](http://www.ncbi.nlm.nih.gov/entrez/query.fcgi?cmd=Search&db=Protein&term=82542702&doptcmdl=GenPept) | preprotein translocase subunit SecA [Shigella boydii Sb227] | 94.09 | 2 | 136 1615 |  |  |
| 258 | [91209444](http://www.ncbi.nlm.nih.gov/entrez/query.fcgi?cmd=Search&db=Protein&term=91209444&doptcmdl=GenPept) | D-alanyl-alanine synthetase A [Escherichia coli UTI89] | 93.74 | 3 | 1047 1068 1168 |  |  |
|  | [91071018](http://www.ncbi.nlm.nih.gov/entrez/query.fcgi?cmd=Search&db=Protein&term=91071018&doptcmdl=GenPept) | D-alanine--D-alanine ligase A [Escherichia coli UTI89] | 93.74 | 3 | 1047 1068 1168 |  |  |
|  | [89107252](http://www.ncbi.nlm.nih.gov/entrez/query.fcgi?cmd=Search&db=Protein&term=89107252&doptcmdl=GenPept) | D-alanine-D-alanine ligase A [Escherichia coli W3110] | 93.74 | 3 | 1047 1068 1168 |  |  |
|  | [85674522](http://www.ncbi.nlm.nih.gov/entrez/query.fcgi?cmd=Search&db=Protein&term=85674522&doptcmdl=GenPept) | D-alanine-D-alanine ligase A [Escherichia coli W3110] | 93.74 | 3 | 1047 1068 1168 |  |  |
|  | [83585699](http://www.ncbi.nlm.nih.gov/entrez/query.fcgi?cmd=Search&db=Protein&term=83585699&doptcmdl=GenPept) | COG1181: D-alanine-D-alanine ligase and related ATP-grasp enzymes [Escherichia coli 101-1] | 93.74 | 3 | 1047 1068 1168 |  |  |
| 259 | [91210283](http://www.ncbi.nlm.nih.gov/entrez/query.fcgi?cmd=Search&db=Protein&term=91210283&doptcmdl=GenPept) | peptidase T [Escherichia coli UTI89] | 93.23 | 2 | 638 683 |  |  |
|  | [91071857](http://www.ncbi.nlm.nih.gov/entrez/query.fcgi?cmd=Search&db=Protein&term=91071857&doptcmdl=GenPept) | peptidase T [Escherichia coli UTI89] | 93.23 | 2 | 638 683 |  |  |
|  | [89107973](http://www.ncbi.nlm.nih.gov/entrez/query.fcgi?cmd=Search&db=Protein&term=89107973&doptcmdl=GenPept) | peptidase T [Escherichia coli W3110] | 93.23 | 2 | 638 683 |  |  |
|  | [83587299](http://www.ncbi.nlm.nih.gov/entrez/query.fcgi?cmd=Search&db=Protein&term=83587299&doptcmdl=GenPept) | COG2195: Di- and tripeptidases [Escherichia coli 101-1] | 93.23 | 2 | 638 683 |  |  |
|  | [83569552](http://www.ncbi.nlm.nih.gov/entrez/query.fcgi?cmd=Search&db=Protein&term=83569552&doptcmdl=GenPept) | COG2195: Di- and tripeptidases [Shigella dysenteriae 1012] | 93.23 | 2 | 638 683 |  |  |
| 260 | [91209527](http://www.ncbi.nlm.nih.gov/entrez/query.fcgi?cmd=Search&db=Protein&term=91209527&doptcmdl=GenPept) | hypothetical protein YbaY [Escherichia coli UTI89] | 91.32 | 1 | 1352 |  |  |
|  | [91071101](http://www.ncbi.nlm.nih.gov/entrez/query.fcgi?cmd=Search&db=Protein&term=91071101&doptcmdl=GenPept) | hypothetical protein YbaY [Escherichia coli UTI89] | 91.32 | 1 | 1352 |  |  |
|  | [89107323](http://www.ncbi.nlm.nih.gov/entrez/query.fcgi?cmd=Search&db=Protein&term=89107323&doptcmdl=GenPept) | predicted outer membrane lipoprotein [Escherichia coli W3110] | 91.32 | 1 | 1352 |  |  |
|  | [85674593](http://www.ncbi.nlm.nih.gov/entrez/query.fcgi?cmd=Search&db=Protein&term=85674593&doptcmdl=GenPept) | predicted outer membrane lipoprotein [Escherichia coli W3110] | 91.32 | 1 | 1352 |  |  |
|  | [49176025](http://www.ncbi.nlm.nih.gov/entrez/query.fcgi?cmd=Search&db=Protein&term=49176025&doptcmdl=GenPept) | predicted outer membrane lipoprotein [Escherichia coli str. K-12 substr. MG1655] | 91.32 | 1 | 1352 |  |  |
| 261 | [91210940](http://www.ncbi.nlm.nih.gov/entrez/query.fcgi?cmd=Search&db=Protein&term=91210940&doptcmdl=GenPept) | hypothetical protein YniA [Escherichia coli UTI89] | 91.17 | 1 | 22 |  |  |
|  | [91072514](http://www.ncbi.nlm.nih.gov/entrez/query.fcgi?cmd=Search&db=Protein&term=91072514&doptcmdl=GenPept) | hypothetical protein YniA [Escherichia coli UTI89] | 91.17 | 1 | 22 |  |  |
|  | [89108564](http://www.ncbi.nlm.nih.gov/entrez/query.fcgi?cmd=Search&db=Protein&term=89108564&doptcmdl=GenPept) | predicted phosphotransferase/kinase [Escherichia coli W3110] | 91.17 | 1 | 22 |  |  |
|  | [83586025](http://www.ncbi.nlm.nih.gov/entrez/query.fcgi?cmd=Search&db=Protein&term=83586025&doptcmdl=GenPept) | COG3001: Fructosamine-3-kinase [Escherichia coli 101-1] | 91.17 | 1 | 22 |  |  |
|  | [82777071](http://www.ncbi.nlm.nih.gov/entrez/query.fcgi?cmd=Search&db=Protein&term=82777071&doptcmdl=GenPept) | hypothetical protein SDY_1818 [Shigella dysenteriae Sd197] | 91.17 | 1 | 22 |  |  |
| 262 | [91213382](http://www.ncbi.nlm.nih.gov/entrez/query.fcgi?cmd=Search&db=Protein&term=91213382&doptcmdl=GenPept) | ubiquinone/menaquinone biosynthesis methyltransferase [Escherichia coli UTI89] | 90.61 | 2 | 691 1562 |  |  |
|  | [91074956](http://www.ncbi.nlm.nih.gov/entrez/query.fcgi?cmd=Search&db=Protein&term=91074956&doptcmdl=GenPept) | ubiquinone/menaquinone biosynthesis methyltransferase ubiE [Escherichia coli UTI89] | 90.61 | 2 | 691 1562 |  |  |
|  | [89110187](http://www.ncbi.nlm.nih.gov/entrez/query.fcgi?cmd=Search&db=Protein&term=89110187&doptcmdl=GenPept) | bifunctional 2-octaprenyl-6-methoxy-1,4-benzoquinone methylase and S-adenosylmethionine:2-DMK methyltransferase [Escherichia coli W3110] | 90.61 | 2 | 691 1562 |  |  |
|  | [85676218](http://www.ncbi.nlm.nih.gov/entrez/query.fcgi?cmd=Search&db=Protein&term=85676218&doptcmdl=GenPept) | bifunctional 2-octaprenyl-6-methoxy-1,4-benzoquinone methylase and S-adenosylmethionine:2-DMK methyltransferase [Escherichia coli W3110] | 90.61 | 2 | 691 1562 |  |  |
|  | [83585906](http://www.ncbi.nlm.nih.gov/entrez/query.fcgi?cmd=Search&db=Protein&term=83585906&doptcmdl=GenPept) | COG2226: Methylase involved in ubiquinone/menaquinone biosynthesis [Escherichia coli 101-1] | 90.61 | 2 | 691 1562 |  |  |

| **Protein IDs*, cont.*** | | | | | | | |
| --- | --- | --- | --- | --- | --- | --- | --- |
| *Grp Nr.* | *Accession Number* | *Protein Name* | *Protein Score* | *Unique PSMs* | *PSM Serial Nrs.* | *Other Grp.* | *Score (other)* |
| 263 | [89107281](http://www.ncbi.nlm.nih.gov/entrez/query.fcgi?cmd=Search&db=Protein&term=89107281&doptcmdl=GenPept) | nucleoside channel, receptor of phage T6 and colicin K [Escherichia coli W3110] | 90.47 | 2 | 483 1290 |  |  |
|  | [85674551](http://www.ncbi.nlm.nih.gov/entrez/query.fcgi?cmd=Search&db=Protein&term=85674551&doptcmdl=GenPept) | nucleoside channel, receptor of phage T6 and colicin K [Escherichia coli W3110] | 90.47 | 2 | 483 1290 |  |  |
|  | [83585670](http://www.ncbi.nlm.nih.gov/entrez/query.fcgi?cmd=Search&db=Protein&term=83585670&doptcmdl=GenPept) | COG3248: Nucleoside-binding outer membrane protein [Escherichia coli 101-1] | 90.47 | 2 | 483 1290 |  |  |
|  | [82775684](http://www.ncbi.nlm.nih.gov/entrez/query.fcgi?cmd=Search&db=Protein&term=82775684&doptcmdl=GenPept) | nucleoside channel [Shigella dysenteriae Sd197] | 90.47 | 2 | 483 1290 |  |  |
|  | [81239832](http://www.ncbi.nlm.nih.gov/entrez/query.fcgi?cmd=Search&db=Protein&term=81239832&doptcmdl=GenPept) | nucleoside channel [Shigella dysenteriae Sd197] | 90.47 | 2 | 483 1290 |  |  |
| 264 | [91213470](http://www.ncbi.nlm.nih.gov/entrez/query.fcgi?cmd=Search&db=Protein&term=91213470&doptcmdl=GenPept) | ribonuclease activity regulator protein RraA [Escherichia coli UTI89] | 89.48 | 3 | 486 513 1662 |  |  |
|  | [91075044](http://www.ncbi.nlm.nih.gov/entrez/query.fcgi?cmd=Search&db=Protein&term=91075044&doptcmdl=GenPept) | ribonuclease E inhibitor subunit [Escherichia coli UTI89] | 89.48 | 3 | 486 513 1662 |  |  |
|  | [89110100](http://www.ncbi.nlm.nih.gov/entrez/query.fcgi?cmd=Search&db=Protein&term=89110100&doptcmdl=GenPept) | ribonuclease E (RNase E) inhibitor protein [Escherichia coli W3110] | 89.48 | 3 | 486 513 1662 |  |  |
|  | [85676131](http://www.ncbi.nlm.nih.gov/entrez/query.fcgi?cmd=Search&db=Protein&term=85676131&doptcmdl=GenPept) | ribonuclease E (RNase E) inhibitor protein [Escherichia coli W3110] | 89.48 | 3 | 486 513 1662 |  |  |
|  | [83586227](http://www.ncbi.nlm.nih.gov/entrez/query.fcgi?cmd=Search&db=Protein&term=83586227&doptcmdl=GenPept) | COG0684: Demethylmenaquinone methyltransferase [Escherichia coli 101-1] | 89.48 | 3 | 486 513 1662 |  |  |
| 265 | [91071057](http://www.ncbi.nlm.nih.gov/entrez/query.fcgi?cmd=Search&db=Protein&term=91071057&doptcmdl=GenPept) | riboflavin synthase beta chain [Escherichia coli UTI89] | 89.45 | 2 | 111 490 |  |  |
|  | [89107285](http://www.ncbi.nlm.nih.gov/entrez/query.fcgi?cmd=Search&db=Protein&term=89107285&doptcmdl=GenPept) | riboflavin synthase beta chain [Escherichia coli W3110] | 89.45 | 2 | 111 490 |  |  |
|  | [85674555](http://www.ncbi.nlm.nih.gov/entrez/query.fcgi?cmd=Search&db=Protein&term=85674555&doptcmdl=GenPept) | riboflavin synthase beta chain [Escherichia coli W3110] | 89.45 | 2 | 111 490 |  |  |
|  | [83585666](http://www.ncbi.nlm.nih.gov/entrez/query.fcgi?cmd=Search&db=Protein&term=83585666&doptcmdl=GenPept) | COG0054: Riboflavin synthase beta-chain [Escherichia coli 101-1] | 89.45 | 2 | 111 490 |  |  |
|  | [83570317](http://www.ncbi.nlm.nih.gov/entrez/query.fcgi?cmd=Search&db=Protein&term=83570317&doptcmdl=GenPept) | COG0054: Riboflavin synthase beta-chain [Shigella dysenteriae 1012] | 89.45 | 2 | 111 490 |  |  |
| 266 | [91209242](http://www.ncbi.nlm.nih.gov/entrez/query.fcgi?cmd=Search&db=Protein&term=91209242&doptcmdl=GenPept) | ribosome recycling factor [Escherichia coli UTI89] | 89.15 | 2 | 615 1714 |  |  |
|  | [91070816](http://www.ncbi.nlm.nih.gov/entrez/query.fcgi?cmd=Search&db=Protein&term=91070816&doptcmdl=GenPept) | ribosome recycling factor [Escherichia coli UTI89] | 89.15 | 2 | 615 1714 |  |  |
|  | [89107052](http://www.ncbi.nlm.nih.gov/entrez/query.fcgi?cmd=Search&db=Protein&term=89107052&doptcmdl=GenPept) | ribosome recycling factor [Escherichia coli W3110] | 89.15 | 2 | 615 1714 |  |  |
|  | [83585488](http://www.ncbi.nlm.nih.gov/entrez/query.fcgi?cmd=Search&db=Protein&term=83585488&doptcmdl=GenPept) | COG0233: Ribosome recycling factor [Escherichia coli 101-1] | 89.15 | 2 | 615 1714 |  |  |
|  | [83569754](http://www.ncbi.nlm.nih.gov/entrez/query.fcgi?cmd=Search&db=Protein&term=83569754&doptcmdl=GenPept) | COG0233: Ribosome recycling factor [Shigella dysenteriae 1012] | 89.15 | 2 | 615 1714 |  |  |
| 267 | [91213409](http://www.ncbi.nlm.nih.gov/entrez/query.fcgi?cmd=Search&db=Protein&term=91213409&doptcmdl=GenPept) | DNA polymerase I [Escherichia coli UTI89] | 88.78 | 2 | 244 1405 |  |  |
|  | [91074983](http://www.ncbi.nlm.nih.gov/entrez/query.fcgi?cmd=Search&db=Protein&term=91074983&doptcmdl=GenPept) | DNA polymerase I [Escherichia coli UTI89] | 88.78 | 2 | 244 1405 |  |  |
|  | [89110164](http://www.ncbi.nlm.nih.gov/entrez/query.fcgi?cmd=Search&db=Protein&term=89110164&doptcmdl=GenPept) | fused DNA polymerase I 5'->3' exonuclease, 3'->5' polymerase and 3'->5' exonuclease [Escherichia coli W3110] | 88.78 | 2 | 244 1405 |  |  |
|  | [85676195](http://www.ncbi.nlm.nih.gov/entrez/query.fcgi?cmd=Search&db=Protein&term=85676195&doptcmdl=GenPept) | fused DNA polymerase I 5'->3' exonuclease, 3'->5' polymerase and 3'->5' exonuclease [Escherichia coli W3110] | 88.78 | 2 | 244 1405 |  |  |
|  | [83584518](http://www.ncbi.nlm.nih.gov/entrez/query.fcgi?cmd=Search&db=Protein&term=83584518&doptcmdl=GenPept) | COG0749: DNA polymerase I - 3'-5' exonuclease and polymerase domains [Escherichia coli 101-1] | 88.78 | 2 | 244 1405 |  |  |
| 268 | [93278469](http://www.ncbi.nlm.nih.gov/entrez/query.fcgi?cmd=Search&db=Protein&term=93278469&doptcmdl=GenPept) | Chain N, Clpp | 88.56 | 2 | 37 705 |  |  |
|  | [93278468](http://www.ncbi.nlm.nih.gov/entrez/query.fcgi?cmd=Search&db=Protein&term=93278468&doptcmdl=GenPept) | Chain M, Clpp | 88.56 | 2 | 37 705 |  |  |
|  | [93278467](http://www.ncbi.nlm.nih.gov/entrez/query.fcgi?cmd=Search&db=Protein&term=93278467&doptcmdl=GenPept) | Chain L, Clpp | 88.56 | 2 | 37 705 |  |  |
|  | [93278466](http://www.ncbi.nlm.nih.gov/entrez/query.fcgi?cmd=Search&db=Protein&term=93278466&doptcmdl=GenPept) | Chain K, Clpp | 88.56 | 2 | 37 705 |  |  |
|  | [93278465](http://www.ncbi.nlm.nih.gov/entrez/query.fcgi?cmd=Search&db=Protein&term=93278465&doptcmdl=GenPept) | Chain J, Clpp | 88.56 | 2 | 37 705 |  |  |
| 269 | [91213453](http://www.ncbi.nlm.nih.gov/entrez/query.fcgi?cmd=Search&db=Protein&term=91213453&doptcmdl=GenPept) | DNA-binding response regulator in two-component regulatory system with CpxA [Escherichia coli UTI89] | 87.82 | 3 | 413 1546 1583 |  |  |
|  | [91075027](http://www.ncbi.nlm.nih.gov/entrez/query.fcgi?cmd=Search&db=Protein&term=91075027&doptcmdl=GenPept) | transcriptional regulatory protein CpxR [Escherichia coli UTI89] | 87.82 | 3 | 413 1546 1583 |  |  |
|  | [89110116](http://www.ncbi.nlm.nih.gov/entrez/query.fcgi?cmd=Search&db=Protein&term=89110116&doptcmdl=GenPept) | DNA-binding response regulator in two-component regulatory system with CpxA [Escherichia coli W3110] | 87.82 | 3 | 413 1546 1583 |  |  |
|  | [85676147](http://www.ncbi.nlm.nih.gov/entrez/query.fcgi?cmd=Search&db=Protein&term=85676147&doptcmdl=GenPept) | DNA-binding response regulator in two-component regulatory system with CpxA [Escherichia coli W3110] | 87.82 | 3 | 413 1546 1583 |  |  |
|  | [83586243](http://www.ncbi.nlm.nih.gov/entrez/query.fcgi?cmd=Search&db=Protein&term=83586243&doptcmdl=GenPept) | COG0745: Response regulators consisting of a CheY-like receiver domain and a winged-helix DNA-binding domain [Escherichia coli 101-1] | 87.82 | 3 | 413 1546 1583 |  |  |
| 270 | [91071266](http://www.ncbi.nlm.nih.gov/entrez/query.fcgi?cmd=Search&db=Protein&term=91071266&doptcmdl=GenPept) | hypothetical protein YbeK [Escherichia coli UTI89] [MASS=35113] | 87.70 | 1 | 1398 |  |  |
|  | [89107520](http://www.ncbi.nlm.nih.gov/entrez/query.fcgi?cmd=Search&db=Protein&term=89107520&doptcmdl=GenPept) | ribonucleoside hydrolase 1 [Escherichia coli W3110] | 87.70 | 1 | 1398 |  |  |
|  | [88191852](http://www.ncbi.nlm.nih.gov/entrez/query.fcgi?cmd=Search&db=Protein&term=88191852&doptcmdl=GenPept) | Chain A, Crystal Structure Of A The E. Coli Pyrimidine Nucleoside Hydrolase Ybek With Bound Ribose [MASS=35090] | 87.70 | 1 | 1398 |  |  |
|  | [85541757](http://www.ncbi.nlm.nih.gov/entrez/query.fcgi?cmd=Search&db=Protein&term=85541757&doptcmdl=GenPept) | RecName: Full=Pyrimidine-specific ribonucleoside hydrolase rihA; AltName: Full=Cytidine/uridine-specific hydrolase | 87.70 | 1 | 1398 |  |  |
|  | [85540732](http://www.ncbi.nlm.nih.gov/entrez/query.fcgi?cmd=Search&db=Protein&term=85540732&doptcmdl=GenPept) | Pyrimidine-specific ribonucleoside hydrolase rihA (Cytidine/uridine-specific hydrolase) | 87.70 | 1 | 1398 |  |  |
| 271 | [91212991](http://www.ncbi.nlm.nih.gov/entrez/query.fcgi?cmd=Search&db=Protein&term=91212991&doptcmdl=GenPept) | oligopeptidase A [Escherichia coli UTI89] | 87.25 | 2 | 1565 1725 |  |  |
|  | [91074565](http://www.ncbi.nlm.nih.gov/entrez/query.fcgi?cmd=Search&db=Protein&term=91074565&doptcmdl=GenPept) | oligopeptidase A [Escherichia coli UTI89] [MASS=78515] | 87.25 | 2 | 1565 1725 |  |  |
|  | [89110515](http://www.ncbi.nlm.nih.gov/entrez/query.fcgi?cmd=Search&db=Protein&term=89110515&doptcmdl=GenPept) | oligopeptidase A [Escherichia coli W3110] | 87.25 | 2 | 1565 1725 |  |  |
|  | [85676546](http://www.ncbi.nlm.nih.gov/entrez/query.fcgi?cmd=Search&db=Protein&term=85676546&doptcmdl=GenPept) | oligopeptidase A [Escherichia coli W3110] | 87.25 | 2 | 1565 1725 |  |  |
|  | [83588644](http://www.ncbi.nlm.nih.gov/entrez/query.fcgi?cmd=Search&db=Protein&term=83588644&doptcmdl=GenPept) | COG0339: Zn-dependent oligopeptidases [Escherichia coli 101-1] | 87.25 | 2 | 1565 1725 |  |  |
| 272 | [91212724](http://www.ncbi.nlm.nih.gov/entrez/query.fcgi?cmd=Search&db=Protein&term=91212724&doptcmdl=GenPept) | 30S ribosomal protein S11 [Escherichia coli UTI89] | 87.03 | 1 | 1077 |  |  |
|  | [91207679](http://www.ncbi.nlm.nih.gov/entrez/query.fcgi?cmd=Search&db=Protein&term=91207679&doptcmdl=GenPept) | 30S ribosomal protein S11 | 87.03 | 1 | 1077 |  |  |
|  | [91207678](http://www.ncbi.nlm.nih.gov/entrez/query.fcgi?cmd=Search&db=Protein&term=91207678&doptcmdl=GenPept) | 30S ribosomal protein S11 | 87.03 | 1 | 1077 |  |  |
|  | [91207677](http://www.ncbi.nlm.nih.gov/entrez/query.fcgi?cmd=Search&db=Protein&term=91207677&doptcmdl=GenPept) | 30S ribosomal protein S11 | 87.03 | 1 | 1077 |  |  |
|  | [91074298](http://www.ncbi.nlm.nih.gov/entrez/query.fcgi?cmd=Search&db=Protein&term=91074298&doptcmdl=GenPept) | 30S ribosomal subunit protein S11 [Escherichia coli UTI89] | 87.03 | 1 | 1077 |  |  |
| 273 | [91209689](http://www.ncbi.nlm.nih.gov/entrez/query.fcgi?cmd=Search&db=Protein&term=91209689&doptcmdl=GenPept) | LPS-assembly lipoprotein RplB [Escherichia coli UTI89] | 86.63 | 1 | 314 |  |  |
|  | [91071263](http://www.ncbi.nlm.nih.gov/entrez/query.fcgi?cmd=Search&db=Protein&term=91071263&doptcmdl=GenPept) | RlpB [Escherichia coli UTI89] | 86.63 | 1 | 314 |  |  |
|  | [89107510](http://www.ncbi.nlm.nih.gov/entrez/query.fcgi?cmd=Search&db=Protein&term=89107510&doptcmdl=GenPept) | minor lipoprotein [Escherichia coli W3110] | 86.63 | 1 | 314 |  |  |
|  | [83584762](http://www.ncbi.nlm.nih.gov/entrez/query.fcgi?cmd=Search&db=Protein&term=83584762&doptcmdl=GenPept) | COG2980: Rare lipoprotein B [Escherichia coli 101-1] | 86.63 | 1 | 314 |  |  |
|  | [83287919](http://www.ncbi.nlm.nih.gov/entrez/query.fcgi?cmd=Search&db=Protein&term=83287919&doptcmdl=GenPept) | LPS-assembly lipoprotein rlpB precursor (Rare lipoprotein B) | 86.63 | 1 | 314 |  |  |
| 274 | [91210823](http://www.ncbi.nlm.nih.gov/entrez/query.fcgi?cmd=Search&db=Protein&term=91210823&doptcmdl=GenPept) | fumarase A [Escherichia coli UTI89] | 86.06 | 1 | 558 | *28* | *365.79* |
|  | [91072397](http://www.ncbi.nlm.nih.gov/entrez/query.fcgi?cmd=Search&db=Protein&term=91072397&doptcmdl=GenPept) | fumarase A [Escherichia coli UTI89] | 86.06 | 1 | 558 | *28* | *365.79* |
|  | [89108453](http://www.ncbi.nlm.nih.gov/entrez/query.fcgi?cmd=Search&db=Protein&term=89108453&doptcmdl=GenPept) | aerobic Class I fumarate hydratase [Escherichia coli W3110] | 86.06 | 1 | 558 | *28* | *365.79* |
|  | [83587947](http://www.ncbi.nlm.nih.gov/entrez/query.fcgi?cmd=Search&db=Protein&term=83587947&doptcmdl=GenPept) | COG1951: Tartrate dehydratase alpha subunit/Fumarate hydratase class I, N-terminal domain [Escherichia coli 101-1] | 86.06 | 1 | 558 | *28* | *365.79* |
|  | [82544021](http://www.ncbi.nlm.nih.gov/entrez/query.fcgi?cmd=Search&db=Protein&term=82544021&doptcmdl=GenPept) | fumarase A [Shigella boydii Sb227] | 86.06 | 1 | 558 | *28* | *365.79* |
| 275 | [91212543](http://www.ncbi.nlm.nih.gov/entrez/query.fcgi?cmd=Search&db=Protein&term=91212543&doptcmdl=GenPept) | threonine dehydratase [Escherichia coli UTI89] | 85.46 | 1 | 1701 |  |  |
|  | [91074117](http://www.ncbi.nlm.nih.gov/entrez/query.fcgi?cmd=Search&db=Protein&term=91074117&doptcmdl=GenPept) | threonine dehydratase, catabolic [Escherichia coli UTI89] | 85.46 | 1 | 1701 |  |  |
|  | [89109885](http://www.ncbi.nlm.nih.gov/entrez/query.fcgi?cmd=Search&db=Protein&term=89109885&doptcmdl=GenPept) | catabolic threonine dehydratase, PLP-dependent [Escherichia coli W3110] | 85.46 | 1 | 1701 |  |  |
|  | [85675916](http://www.ncbi.nlm.nih.gov/entrez/query.fcgi?cmd=Search&db=Protein&term=85675916&doptcmdl=GenPept) | catabolic threonine dehydratase, PLP-dependent [Escherichia coli W3110] | 85.46 | 1 | 1701 |  |  |
|  | [84027808](http://www.ncbi.nlm.nih.gov/entrez/query.fcgi?cmd=Search&db=Protein&term=84027808&doptcmdl=GenPept) | Threonine dehydratase catabolic (Threonine deaminase) | 85.46 | 1 | 1701 |  |  |
| 276 | [91212609](http://www.ncbi.nlm.nih.gov/entrez/query.fcgi?cmd=Search&db=Protein&term=91212609&doptcmdl=GenPept) | 50S ribosomal protein L21 [Escherichia coli UTI89] | 85.20 | 2 | 1333 1530 |  |  |
|  | [91074183](http://www.ncbi.nlm.nih.gov/entrez/query.fcgi?cmd=Search&db=Protein&term=91074183&doptcmdl=GenPept) | 50S ribosomal subunit protein L21 [Escherichia coli UTI89] | 85.20 | 2 | 1333 1530 |  |  |
|  | [89109949](http://www.ncbi.nlm.nih.gov/entrez/query.fcgi?cmd=Search&db=Protein&term=89109949&doptcmdl=GenPept) | 50S ribosomal subunit protein L21 [Escherichia coli W3110] | 85.20 | 2 | 1333 1530 |  |  |
|  | [85675980](http://www.ncbi.nlm.nih.gov/entrez/query.fcgi?cmd=Search&db=Protein&term=85675980&doptcmdl=GenPept) | 50S ribosomal subunit protein L21 [Escherichia coli W3110] | 85.20 | 2 | 1333 1530 |  |  |
|  | [84027994](http://www.ncbi.nlm.nih.gov/entrez/query.fcgi?cmd=Search&db=Protein&term=84027994&doptcmdl=GenPept) | 50S ribosomal protein L21 | 85.20 | 2 | 1333 1530 |  |  |
| 277 | [91212729](http://www.ncbi.nlm.nih.gov/entrez/query.fcgi?cmd=Search&db=Protein&term=91212729&doptcmdl=GenPept) | 50S ribosomal protein L15 [Escherichia coli UTI89] | 85.19 | 1 | 1026 |  |  |
|  | [91074303](http://www.ncbi.nlm.nih.gov/entrez/query.fcgi?cmd=Search&db=Protein&term=91074303&doptcmdl=GenPept) | 50S ribosomal subunit protein L15 [Escherichia coli UTI89] | 85.19 | 1 | 1026 |  |  |
|  | [89110709](http://www.ncbi.nlm.nih.gov/entrez/query.fcgi?cmd=Search&db=Protein&term=89110709&doptcmdl=GenPept) | 50S ribosomal subunit protein L15 [Escherichia coli W3110] | 85.19 | 1 | 1026 |  |  |
|  | [85676740](http://www.ncbi.nlm.nih.gov/entrez/query.fcgi?cmd=Search&db=Protein&term=85676740&doptcmdl=GenPept) | 50S ribosomal subunit protein L15 [Escherichia coli W3110] | 85.19 | 1 | 1026 |  |  |
|  | [83754128](http://www.ncbi.nlm.nih.gov/entrez/query.fcgi?cmd=Search&db=Protein&term=83754128&doptcmdl=GenPept) | Chain L, Crystal Structure Of The Bacterial Ribosome From Escherichia Coli At 3.5 A Resolution. This File Contains The 50s Subunit Of The Second 70s Ribosome. The Entire Crystal Structure Contains Two 70s Ribosomes And Is Described In Remark 400. | 85.19 | 1 | 1026 |  |  |
| 278 | [91213797](http://www.ncbi.nlm.nih.gov/entrez/query.fcgi?cmd=Search&db=Protein&term=91213797&doptcmdl=GenPept) | hypothetical protein UTI89_C4848 [Escherichia coli UTI89] | 84.87 | 2 | 43 983 |  |  |
|  | [91075371](http://www.ncbi.nlm.nih.gov/entrez/query.fcgi?cmd=Search&db=Protein&term=91075371&doptcmdl=GenPept) | conserved hypothetical protein [Escherichia coli UTI89] [MASS=16520] | 84.87 | 2 | 43 983 |  |  |
|  | [90111711](http://www.ncbi.nlm.nih.gov/entrez/query.fcgi?cmd=Search&db=Protein&term=90111711&doptcmdl=GenPept) | ketoacid-binding protein [Escherichia coli str. K-12 substr. MG1655] | 84.87 | 2 | 43 983 |  |  |
|  | [89110961](http://www.ncbi.nlm.nih.gov/entrez/query.fcgi?cmd=Search&db=Protein&term=89110961&doptcmdl=GenPept) | ketoacid-binding protein [Escherichia coli W3110] | 84.87 | 2 | 43 983 |  |  |
|  | [87082396](http://www.ncbi.nlm.nih.gov/entrez/query.fcgi?cmd=Search&db=Protein&term=87082396&doptcmdl=GenPept) | ketoacid-binding protein [Escherichia coli str. K-12 substr. MG1655] | 84.87 | 2 | 43 983 |  |  |
| 279 | [91212673](http://www.ncbi.nlm.nih.gov/entrez/query.fcgi?cmd=Search&db=Protein&term=91212673&doptcmdl=GenPept) | protein YhdH [Escherichia coli UTI89] | 84.50 | 2 | 907 1264 |  |  |
|  | [91074247](http://www.ncbi.nlm.nih.gov/entrez/query.fcgi?cmd=Search&db=Protein&term=91074247&doptcmdl=GenPept) | protein YhdH [Escherichia coli UTI89] | 84.50 | 2 | 907 1264 |  |  |
|  | [89110014](http://www.ncbi.nlm.nih.gov/entrez/query.fcgi?cmd=Search&db=Protein&term=89110014&doptcmdl=GenPept) | predicted oxidoreductase, Zn-dependent and NAD(P)-binding [Escherichia coli W3110] | 84.50 | 2 | 907 1264 |  |  |
|  | [85676045](http://www.ncbi.nlm.nih.gov/entrez/query.fcgi?cmd=Search&db=Protein&term=85676045&doptcmdl=GenPept) | predicted oxidoreductase, Zn-dependent and NAD(P)-binding [Escherichia coli W3110] | 84.50 | 2 | 907 1264 |  |  |
|  | [83584498](http://www.ncbi.nlm.nih.gov/entrez/query.fcgi?cmd=Search&db=Protein&term=83584498&doptcmdl=GenPept) | COG0604: NADPH:quinone reductase and related Zn-dependent oxidoreductases [Escherichia coli 101-1] | 84.50 | 2 | 907 1264 |  |  |
| 280 | [91212746](http://www.ncbi.nlm.nih.gov/entrez/query.fcgi?cmd=Search&db=Protein&term=91212746&doptcmdl=GenPept) | 50S ribosomal protein L16 [Escherichia coli UTI89] | 84.19 | 1 | 1367 |  |  |
|  | [91074320](http://www.ncbi.nlm.nih.gov/entrez/query.fcgi?cmd=Search&db=Protein&term=91074320&doptcmdl=GenPept) | 50S ribosomal subunit protein L16 [Escherichia coli UTI89] | 84.19 | 1 | 1367 |  |  |
|  | [89110697](http://www.ncbi.nlm.nih.gov/entrez/query.fcgi?cmd=Search&db=Protein&term=89110697&doptcmdl=GenPept) | 50S ribosomal subunit protein L16 [Escherichia coli W3110] | 84.19 | 1 | 1367 |  |  |
|  | [85701223](http://www.ncbi.nlm.nih.gov/entrez/query.fcgi?cmd=Search&db=Protein&term=85701223&doptcmdl=GenPept) | 50S ribosomal protein L16 | 84.19 | 1 | 1367 |  |  |
|  | [85701222](http://www.ncbi.nlm.nih.gov/entrez/query.fcgi?cmd=Search&db=Protein&term=85701222&doptcmdl=GenPept) | 50S ribosomal protein L16 | 84.19 | 1 | 1367 |  |  |
| 281 | [91210419](http://www.ncbi.nlm.nih.gov/entrez/query.fcgi?cmd=Search&db=Protein&term=91210419&doptcmdl=GenPept) | dihydroxyacetone kinase ADP-binding subunit [Escherichia coli UTI89] | 84.17 | 2 | 1172 1364 |  |  |
|  | [91071993](http://www.ncbi.nlm.nih.gov/entrez/query.fcgi?cmd=Search&db=Protein&term=91071993&doptcmdl=GenPept) | protein YcgS [Escherichia coli UTI89] [MASS=25552] | 84.17 | 2 | 1172 1364 |  |  |
|  | [89108044](http://www.ncbi.nlm.nih.gov/entrez/query.fcgi?cmd=Search&db=Protein&term=89108044&doptcmdl=GenPept) | dihydroxyacetone kinase, C-terminal domain [Escherichia coli W3110] | 84.17 | 2 | 1172 1364 |  |  |
|  | [85674873](http://www.ncbi.nlm.nih.gov/entrez/query.fcgi?cmd=Search&db=Protein&term=85674873&doptcmdl=GenPept) | dihydroxyacetone kinase, C-terminal domain [Escherichia coli W3110] | 84.17 | 2 | 1172 1364 |  |  |
|  | [83587343](http://www.ncbi.nlm.nih.gov/entrez/query.fcgi?cmd=Search&db=Protein&term=83587343&doptcmdl=GenPept) | COG2376: Dihydroxyacetone kinase [Escherichia coli 101-1] | 84.17 | 2 | 1172 1364 |  |  |
| 282 | [91212293](http://www.ncbi.nlm.nih.gov/entrez/query.fcgi?cmd=Search&db=Protein&term=91212293&doptcmdl=GenPept) | ribose-5-phosphate isomerase A [Escherichia coli UTI89] | 83.90 | 1 | 333 |  |  |
|  | [91073867](http://www.ncbi.nlm.nih.gov/entrez/query.fcgi?cmd=Search&db=Protein&term=91073867&doptcmdl=GenPept) | ribose-5-phosphate isomerase, constitutive [Escherichia coli UTI89] [MASS=25601] | 83.90 | 1 | 333 |  |  |
|  | [89109692](http://www.ncbi.nlm.nih.gov/entrez/query.fcgi?cmd=Search&db=Protein&term=89109692&doptcmdl=GenPept) | ribosephosphate isomerase, constitutive [Escherichia coli W3110] | 83.90 | 1 | 333 |  |  |
|  | [882443](http://www.ncbi.nlm.nih.gov/entrez/query.fcgi?cmd=Search&db=Protein&term=882443&doptcmdl=GenPept) | ribose 5-phosphate isomerase | 83.90 | 1 | 333 |  |  |
|  | [85675725](http://www.ncbi.nlm.nih.gov/entrez/query.fcgi?cmd=Search&db=Protein&term=85675725&doptcmdl=GenPept) | ribosephosphate isomerase, constitutive [Escherichia coli W3110] | 83.90 | 1 | 333 |  |  |
| 283 | [91210219](http://www.ncbi.nlm.nih.gov/entrez/query.fcgi?cmd=Search&db=Protein&term=91210219&doptcmdl=GenPept) | hypothetical protein UTI89_C1192 [Escherichia coli UTI89] | 83.56 | 1 | 174 |  |  |
|  | [91071793](http://www.ncbi.nlm.nih.gov/entrez/query.fcgi?cmd=Search&db=Protein&term=91071793&doptcmdl=GenPept) | hypothetical protein YceH [Escherichia coli UTI89] | 83.56 | 1 | 174 |  |  |
|  | [89107913](http://www.ncbi.nlm.nih.gov/entrez/query.fcgi?cmd=Search&db=Protein&term=89107913&doptcmdl=GenPept) | hypothetical protein [Escherichia coli W3110] | 83.56 | 1 | 174 |  |  |
|  | [83586399](http://www.ncbi.nlm.nih.gov/entrez/query.fcgi?cmd=Search&db=Protein&term=83586399&doptcmdl=GenPept) | COG3132: Uncharacterized protein conserved in bacteria [Escherichia coli 101-1] [MASS=24159] | 83.56 | 1 | 174 |  |  |
|  | [83570486](http://www.ncbi.nlm.nih.gov/entrez/query.fcgi?cmd=Search&db=Protein&term=83570486&doptcmdl=GenPept) | COG3132: Uncharacterized protein conserved in bacteria [Shigella dysenteriae 1012] [MASS=24233] | 83.56 | 1 | 174 |  |  |
| 284 | [91211302](http://www.ncbi.nlm.nih.gov/entrez/query.fcgi?cmd=Search&db=Protein&term=91211302&doptcmdl=GenPept) | protein YeeZ precursor [Escherichia coli UTI89] | 83.49 | 1 | 161 |  |  |
|  | [91072876](http://www.ncbi.nlm.nih.gov/entrez/query.fcgi?cmd=Search&db=Protein&term=91072876&doptcmdl=GenPept) | protein YeeZ precursor [Escherichia coli UTI89] | 83.49 | 1 | 161 |  |  |
|  | [89108836](http://www.ncbi.nlm.nih.gov/entrez/query.fcgi?cmd=Search&db=Protein&term=89108836&doptcmdl=GenPept) | predicted epimerase, with NAD(P)-binding Rossmann-fold domain [Escherichia coli W3110] | 83.49 | 1 | 161 |  |  |
|  | [85675192](http://www.ncbi.nlm.nih.gov/entrez/query.fcgi?cmd=Search&db=Protein&term=85675192&doptcmdl=GenPept) | predicted epimerase, with NAD(P)-binding Rossmann-fold domain [Escherichia coli W3110] | 83.49 | 1 | 161 |  |  |
|  | [83588018](http://www.ncbi.nlm.nih.gov/entrez/query.fcgi?cmd=Search&db=Protein&term=83588018&doptcmdl=GenPept) | COG0451: Nucleoside-diphosphate-sugar epimerases [Escherichia coli 101-1] | 83.49 | 1 | 161 |  |  |
| 285 | [91210199](http://www.ncbi.nlm.nih.gov/entrez/query.fcgi?cmd=Search&db=Protein&term=91210199&doptcmdl=GenPept) | glucan biosynthesis protein G [Escherichia coli UTI89] | 83.37 | 2 | 1018 1520 |  |  |
|  | [91071773](http://www.ncbi.nlm.nih.gov/entrez/query.fcgi?cmd=Search&db=Protein&term=91071773&doptcmdl=GenPept) | periplasmic glucans biosynthesis protein MdoG precursor [Escherichia coli UTI89] | 83.37 | 2 | 1018 1520 |  |  |
|  | [89107895](http://www.ncbi.nlm.nih.gov/entrez/query.fcgi?cmd=Search&db=Protein&term=89107895&doptcmdl=GenPept) | glucan biosynthesis protein, periplasmic [Escherichia coli W3110] | 83.37 | 2 | 1018 1520 |  |  |
|  | [83586416](http://www.ncbi.nlm.nih.gov/entrez/query.fcgi?cmd=Search&db=Protein&term=83586416&doptcmdl=GenPept) | COG3131: Periplasmic glucans biosynthesis protein [Escherichia coli 101-1] | 83.37 | 2 | 1018 1520 |  |  |
|  | [83570506](http://www.ncbi.nlm.nih.gov/entrez/query.fcgi?cmd=Search&db=Protein&term=83570506&doptcmdl=GenPept) | COG3131: Periplasmic glucans biosynthesis protein [Shigella dysenteriae 1012] | 83.37 | 2 | 1018 1520 |  |  |

| **Protein IDs*, cont.*** | | | | | | | |
| --- | --- | --- | --- | --- | --- | --- | --- |
| *Grp Nr.* | *Accession Number* | *Protein Name* | *Protein Score* | *Unique PSMs* | *PSM Serial Nrs.* | *Other Grp.* | *Score (other)* |
| 286 | [91209785](http://www.ncbi.nlm.nih.gov/entrez/query.fcgi?cmd=Search&db=Protein&term=91209785&doptcmdl=GenPept) | galactokinase [Escherichia coli UTI89] | 82.94 | 2 | 554 1287 |  |  |
|  | [91071359](http://www.ncbi.nlm.nih.gov/entrez/query.fcgi?cmd=Search&db=Protein&term=91071359&doptcmdl=GenPept) | galactokinase [Escherichia coli UTI89] | 82.94 | 2 | 554 1287 |  |  |
|  | [89107608](http://www.ncbi.nlm.nih.gov/entrez/query.fcgi?cmd=Search&db=Protein&term=89107608&doptcmdl=GenPept) | galactokinase [Escherichia coli W3110] | 82.94 | 2 | 554 1287 |  |  |
|  | [86516692](http://www.ncbi.nlm.nih.gov/entrez/query.fcgi?cmd=Search&db=Protein&term=86516692&doptcmdl=GenPept) | GalK [Escherichia coli] [MASS=41456] | 82.94 | 2 | 554 1287 |  |  |
|  | [86516690](http://www.ncbi.nlm.nih.gov/entrez/query.fcgi?cmd=Search&db=Protein&term=86516690&doptcmdl=GenPept) | GalK [Escherichia coli] | 82.94 | 2 | 554 1287 |  |  |
| 287 | [91210461](http://www.ncbi.nlm.nih.gov/entrez/query.fcgi?cmd=Search&db=Protein&term=91210461&doptcmdl=GenPept) | thymidine kinase [Escherichia coli UTI89] | 82.62 | 1 | 1384 |  |  |
|  | [91072035](http://www.ncbi.nlm.nih.gov/entrez/query.fcgi?cmd=Search&db=Protein&term=91072035&doptcmdl=GenPept) | thymidine kinase [Escherichia coli UTI89] | 82.62 | 1 | 1384 |  |  |
|  | [89108084](http://www.ncbi.nlm.nih.gov/entrez/query.fcgi?cmd=Search&db=Protein&term=89108084&doptcmdl=GenPept) | thymidine kinase/deoxyuridine kinase [Escherichia coli W3110] | 82.62 | 1 | 1384 |  |  |
|  | [87312671](http://www.ncbi.nlm.nih.gov/entrez/query.fcgi?cmd=Search&db=Protein&term=87312671&doptcmdl=GenPept) | thymidine kinase [Escherichia coli K12] | 82.62 | 1 | 1384 |  |  |
|  | [83587378](http://www.ncbi.nlm.nih.gov/entrez/query.fcgi?cmd=Search&db=Protein&term=83587378&doptcmdl=GenPept) | COG1435: Thymidine kinase [Escherichia coli 101-1] | 82.62 | 1 | 1384 |  |  |
| 288 | [94541093](http://www.ncbi.nlm.nih.gov/entrez/query.fcgi?cmd=Search&db=Protein&term=94541093&doptcmdl=GenPept) | predicted protein [Escherichia coli str. K-12 substr. MG1655] | 82.62 | 1 | 73 |  |  |
|  | [91211124](http://www.ncbi.nlm.nih.gov/entrez/query.fcgi?cmd=Search&db=Protein&term=91211124&doptcmdl=GenPept) | hypothetical protein UTI89_C2103 [Escherichia coli UTI89] | 82.62 | 1 | 73 |  |  |
|  | [91072698](http://www.ncbi.nlm.nih.gov/entrez/query.fcgi?cmd=Search&db=Protein&term=91072698&doptcmdl=GenPept) | hypothetical protein UTI89_C2103 [Escherichia coli UTI89] | 82.62 | 1 | 73 |  |  |
|  | [89108741](http://www.ncbi.nlm.nih.gov/entrez/query.fcgi?cmd=Search&db=Protein&term=89108741&doptcmdl=GenPept) | hypothetical protein [Escherichia coli W3110] | 82.62 | 1 | 73 |  |  |
|  | [87081998](http://www.ncbi.nlm.nih.gov/entrez/query.fcgi?cmd=Search&db=Protein&term=87081998&doptcmdl=GenPept) | predicted protein [Escherichia coli str. K-12 substr. MG1655] | 82.62 | 1 | 73 |  |  |
| 289 | [91212813](http://www.ncbi.nlm.nih.gov/entrez/query.fcgi?cmd=Search&db=Protein&term=91212813&doptcmdl=GenPept) | transcription antitermination protein NusG [Escherichia coli UTI89] | 81.93 | 1 | 1141 |  |  |
|  | [91074387](http://www.ncbi.nlm.nih.gov/entrez/query.fcgi?cmd=Search&db=Protein&term=91074387&doptcmdl=GenPept) | transcription antitermination protein [Escherichia coli UTI89] | 81.93 | 1 | 1141 |  |  |
|  | [90110823](http://www.ncbi.nlm.nih.gov/entrez/query.fcgi?cmd=Search&db=Protein&term=90110823&doptcmdl=GenPept) | Transcription antitermination protein nusG | 81.93 | 1 | 1141 |  |  |
|  | [90110822](http://www.ncbi.nlm.nih.gov/entrez/query.fcgi?cmd=Search&db=Protein&term=90110822&doptcmdl=GenPept) | Transcription antitermination protein nusG | 81.93 | 1 | 1141 |  |  |
|  | [89110057](http://www.ncbi.nlm.nih.gov/entrez/query.fcgi?cmd=Search&db=Protein&term=89110057&doptcmdl=GenPept) | transcription termination factor [Escherichia coli W3110] | 81.93 | 1 | 1141 |  |  |
| 290 | [91209203](http://www.ncbi.nlm.nih.gov/entrez/query.fcgi?cmd=Search&db=Protein&term=91209203&doptcmdl=GenPept) | 3-methyl-2-oxobutanoate hydroxymethyltransferase [Escherichia coli UTI89] | 81.46 | 2 | 863 1246 |  |  |
|  | [91070777](http://www.ncbi.nlm.nih.gov/entrez/query.fcgi?cmd=Search&db=Protein&term=91070777&doptcmdl=GenPept) | 3-methyl-2-oxobutanoate hydroxymethyltransferase [Escherichia coli UTI89] | 81.46 | 2 | 863 1246 |  |  |
|  | [89107015](http://www.ncbi.nlm.nih.gov/entrez/query.fcgi?cmd=Search&db=Protein&term=89107015&doptcmdl=GenPept) | 3-methyl-2-oxobutanoate hydroxymethyltransferase [Escherichia coli W3110] | 81.46 | 2 | 863 1246 |  |  |
|  | [83585453](http://www.ncbi.nlm.nih.gov/entrez/query.fcgi?cmd=Search&db=Protein&term=83585453&doptcmdl=GenPept) | COG0413: Ketopantoate hydroxymethyltransferase [Escherichia coli 101-1] [MASS=28121] | 81.46 | 2 | 863 1246 |  |  |
|  | [75210461](http://www.ncbi.nlm.nih.gov/entrez/query.fcgi?cmd=Search&db=Protein&term=75210461&doptcmdl=GenPept) | COG0413: Ketopantoate hydroxymethyltransferase [Escherichia coli B171] | 81.46 | 2 | 863 1246 |  |  |
| 291 | [9257124](http://www.ncbi.nlm.nih.gov/entrez/query.fcgi?cmd=Search&db=Protein&term=9257124&doptcmdl=GenPept) | Chain B, Structure Of Biotin Carboxylase (Apo) | 81.22 | 2 | 906 1619 |  |  |
|  | [9257123](http://www.ncbi.nlm.nih.gov/entrez/query.fcgi?cmd=Search&db=Protein&term=9257123&doptcmdl=GenPept) | Chain A, Structure Of Biotin Carboxylase (Apo) | 81.22 | 2 | 906 1619 |  |  |
|  | [91074252](http://www.ncbi.nlm.nih.gov/entrez/query.fcgi?cmd=Search&db=Protein&term=91074252&doptcmdl=GenPept) | acetyl CoA carboxylase, biotin carboxylase subunit [Escherichia coli UTI89] [MASS=50789] | 81.22 | 2 | 906 1619 |  |  |
|  | [89110016](http://www.ncbi.nlm.nih.gov/entrez/query.fcgi?cmd=Search&db=Protein&term=89110016&doptcmdl=GenPept) | acetyl-CoA carboxylase, biotin carboxylase subunit [Escherichia coli W3110] | 81.22 | 2 | 906 1619 |  |  |
|  | [85676047](http://www.ncbi.nlm.nih.gov/entrez/query.fcgi?cmd=Search&db=Protein&term=85676047&doptcmdl=GenPept) | acetyl-CoA carboxylase, biotin carboxylase subunit [Escherichia coli W3110] | 81.22 | 2 | 906 1619 |  |  |
| 292 | [91212030](http://www.ncbi.nlm.nih.gov/entrez/query.fcgi?cmd=Search&db=Protein&term=91212030&doptcmdl=GenPept) | hypothetical protein UTI89_C3029 [Escherichia coli UTI89] | 81.10 | 1 | 211 |  |  |
|  | [91073604](http://www.ncbi.nlm.nih.gov/entrez/query.fcgi?cmd=Search&db=Protein&term=91073604&doptcmdl=GenPept) | hypothetical protein UTI89_C3029 [Escherichia coli UTI89] | 81.10 | 1 | 211 |  |  |
|  | [89109465](http://www.ncbi.nlm.nih.gov/entrez/query.fcgi?cmd=Search&db=Protein&term=89109465&doptcmdl=GenPept) | hypothetical protein [Escherichia coli W3110] | 81.10 | 1 | 211 |  |  |
|  | [83585161](http://www.ncbi.nlm.nih.gov/entrez/query.fcgi?cmd=Search&db=Protein&term=83585161&doptcmdl=GenPept) | COG4575: Uncharacterized conserved protein [Escherichia coli 101-1] | 81.10 | 1 | 211 |  |  |
|  | [83287976](http://www.ncbi.nlm.nih.gov/entrez/query.fcgi?cmd=Search&db=Protein&term=83287976&doptcmdl=GenPept) | Uncharacterized protein ygaM | 81.10 | 1 | 211 |  |  |
| 293 | [91212742](http://www.ncbi.nlm.nih.gov/entrez/query.fcgi?cmd=Search&db=Protein&term=91212742&doptcmdl=GenPept) | 50S ribosomal protein L14 [Escherichia coli UTI89] | 80.94 | 2 | 510 1081 |  |  |
|  | [91074316](http://www.ncbi.nlm.nih.gov/entrez/query.fcgi?cmd=Search&db=Protein&term=91074316&doptcmdl=GenPept) | 50S ribosomal subunit protein L14 [Escherichia coli UTI89] | 80.94 | 2 | 510 1081 |  |  |
|  | [89110700](http://www.ncbi.nlm.nih.gov/entrez/query.fcgi?cmd=Search&db=Protein&term=89110700&doptcmdl=GenPept) | 50S ribosomal subunit protein L14 [Escherichia coli W3110] | 80.94 | 2 | 510 1081 |  |  |
|  | [85676731](http://www.ncbi.nlm.nih.gov/entrez/query.fcgi?cmd=Search&db=Protein&term=85676731&doptcmdl=GenPept) | 50S ribosomal subunit protein L14 [Escherichia coli W3110] | 80.94 | 2 | 510 1081 |  |  |
|  | [83754127](http://www.ncbi.nlm.nih.gov/entrez/query.fcgi?cmd=Search&db=Protein&term=83754127&doptcmdl=GenPept) | Chain K, Crystal Structure Of The Bacterial Ribosome From Escherichia Coli At 3.5 A Resolution. This File Contains The 50s Subunit Of The Second 70s Ribosome. The Entire Crystal Structure Contains Two 70s Ribosomes And Is Described In Remark 400. | 80.94 | 2 | 510 1081 |  |  |
| 294 | [91211432](http://www.ncbi.nlm.nih.gov/entrez/query.fcgi?cmd=Search&db=Protein&term=91211432&doptcmdl=GenPept) | putative oxidoreductase [Escherichia coli UTI89] | 80.84 | 1 | 1631 |  |  |
|  | [91073006](http://www.ncbi.nlm.nih.gov/entrez/query.fcgi?cmd=Search&db=Protein&term=91073006&doptcmdl=GenPept) | hypothetical oxidoreductase YeiT [Escherichia coli UTI89] | 80.84 | 1 | 1631 |  |  |
|  | [89108963](http://www.ncbi.nlm.nih.gov/entrez/query.fcgi?cmd=Search&db=Protein&term=89108963&doptcmdl=GenPept) | predicted oxidoreductase [Escherichia coli W3110] | 80.84 | 1 | 1631 |  |  |
|  | [85675260](http://www.ncbi.nlm.nih.gov/entrez/query.fcgi?cmd=Search&db=Protein&term=85675260&doptcmdl=GenPept) | predicted oxidoreductase [Escherichia coli W3110] | 80.84 | 1 | 1631 |  |  |
|  | [83588127](http://www.ncbi.nlm.nih.gov/entrez/query.fcgi?cmd=Search&db=Protein&term=83588127&doptcmdl=GenPept) | COG0493: NADPH-dependent glutamate synthase beta chain and related oxidoreductases [Escherichia coli 101-1] | 80.84 | 1 | 1631 |  |  |
| 295 | [91073187](http://www.ncbi.nlm.nih.gov/entrez/query.fcgi?cmd=Search&db=Protein&term=91073187&doptcmdl=GenPept) | acetyl-coenzyme A carboxylase carboxyl transferase subunit beta [Escherichia coli UTI89] | 80.33 | 2 | 430 1670 |  |  |
|  | [89109136](http://www.ncbi.nlm.nih.gov/entrez/query.fcgi?cmd=Search&db=Protein&term=89109136&doptcmdl=GenPept) | acetylCoA carboxylase, beta (carboxyltranferase) subunit [Escherichia coli W3110] | 80.33 | 2 | 430 1670 |  |  |
|  | [87117416](http://www.ncbi.nlm.nih.gov/entrez/query.fcgi?cmd=Search&db=Protein&term=87117416&doptcmdl=GenPept) | AccD [Escherichia coli] [MASS=32434] | 80.33 | 2 | 430 1670 |  |  |
|  | [87117414](http://www.ncbi.nlm.nih.gov/entrez/query.fcgi?cmd=Search&db=Protein&term=87117414&doptcmdl=GenPept) | AccD [Escherichia coli] | 80.33 | 2 | 430 1670 |  |  |
|  | [87117412](http://www.ncbi.nlm.nih.gov/entrez/query.fcgi?cmd=Search&db=Protein&term=87117412&doptcmdl=GenPept) | AccD [Escherichia coli] | 80.33 | 2 | 430 1670 |  |  |
| 296 | [91210980](http://www.ncbi.nlm.nih.gov/entrez/query.fcgi?cmd=Search&db=Protein&term=91210980&doptcmdl=GenPept) | selenophosphate synthetase [Escherichia coli UTI89] | 80.27 | 2 | 1397 1683 |  |  |
|  | [91072554](http://www.ncbi.nlm.nih.gov/entrez/query.fcgi?cmd=Search&db=Protein&term=91072554&doptcmdl=GenPept) | selenophosphate synthase [Escherichia coli UTI89] | 80.27 | 2 | 1397 1683 |  |  |
|  | [89108603](http://www.ncbi.nlm.nih.gov/entrez/query.fcgi?cmd=Search&db=Protein&term=89108603&doptcmdl=GenPept) | selenophosphate synthase [Escherichia coli W3110] | 80.27 | 2 | 1397 1683 |  |  |
|  | [85675113](http://www.ncbi.nlm.nih.gov/entrez/query.fcgi?cmd=Search&db=Protein&term=85675113&doptcmdl=GenPept) | selenophosphate synthase [Escherichia coli W3110] | 80.27 | 2 | 1397 1683 |  |  |
|  | [83586061](http://www.ncbi.nlm.nih.gov/entrez/query.fcgi?cmd=Search&db=Protein&term=83586061&doptcmdl=GenPept) | COG0709: Selenophosphate synthase [Escherichia coli 101-1] [MASS=36673] | 80.27 | 2 | 1397 1683 |  |  |
| 297 | [91212108](http://www.ncbi.nlm.nih.gov/entrez/query.fcgi?cmd=Search&db=Protein&term=91212108&doptcmdl=GenPept) | RNA polymerase sigma factor RpoS [Escherichia coli UTI89] | 80.02 | 1 | 169 |  |  |
|  | [91073682](http://www.ncbi.nlm.nih.gov/entrez/query.fcgi?cmd=Search&db=Protein&term=91073682&doptcmdl=GenPept) | sigma factor RpoS [Escherichia coli UTI89] | 80.02 | 1 | 169 |  |  |
|  | [89109528](http://www.ncbi.nlm.nih.gov/entrez/query.fcgi?cmd=Search&db=Protein&term=89109528&doptcmdl=GenPept) | RNA polymerase, sigma S (sigma 38) factor [Escherichia coli W3110] | 80.02 | 1 | 169 |  |  |
|  | [882634](http://www.ncbi.nlm.nih.gov/entrez/query.fcgi?cmd=Search&db=Protein&term=882634&doptcmdl=GenPept) | RNA polymerase sigma subunit RpoS (sigma-38) | 80.02 | 1 | 169 |  |  |
|  | [85675562](http://www.ncbi.nlm.nih.gov/entrez/query.fcgi?cmd=Search&db=Protein&term=85675562&doptcmdl=GenPept) | RNA polymerase, sigma S (sigma 38) factor [Escherichia coli W3110] [MASS=33556] | 80.02 | 1 | 169 |  |  |
| 298 | [91211641](http://www.ncbi.nlm.nih.gov/entrez/query.fcgi?cmd=Search&db=Protein&term=91211641&doptcmdl=GenPept) | long-chain fatty acid outer membrane transporter [Escherichia coli UTI89] | 79.77 | 1 | 563 |  |  |
|  | [91073215](http://www.ncbi.nlm.nih.gov/entrez/query.fcgi?cmd=Search&db=Protein&term=91073215&doptcmdl=GenPept) | bifunctional long-chain fatty acids transporter [Escherichia coli UTI89] | 79.77 | 1 | 563 |  |  |
|  | [89109164](http://www.ncbi.nlm.nih.gov/entrez/query.fcgi?cmd=Search&db=Protein&term=89109164&doptcmdl=GenPept) | long-chain fatty acid outer membrane transporter [Escherichia coli W3110] | 79.77 | 1 | 563 |  |  |
|  | [83588365](http://www.ncbi.nlm.nih.gov/entrez/query.fcgi?cmd=Search&db=Protein&term=83588365&doptcmdl=GenPept) | COG2067: Long-chain fatty acid transport protein [Escherichia coli 101-1] [MASS=47959] | 79.77 | 1 | 563 |  |  |
|  | [75210150](http://www.ncbi.nlm.nih.gov/entrez/query.fcgi?cmd=Search&db=Protein&term=75210150&doptcmdl=GenPept) | COG2067: Long-chain fatty acid transport protein [Escherichia coli B171] | 79.77 | 1 | 563 |  |  |
| 299 | [91210244](http://www.ncbi.nlm.nih.gov/entrez/query.fcgi?cmd=Search&db=Protein&term=91210244&doptcmdl=GenPept) | malonyl CoA-acyl carrier protein transacylase [Escherichia coli UTI89] | 79.05 | 2 | 1259 1561 |  |  |
|  | [91071818](http://www.ncbi.nlm.nih.gov/entrez/query.fcgi?cmd=Search&db=Protein&term=91071818&doptcmdl=GenPept) | malonyl CoA-acyl carrier protein transacylase [Escherichia coli UTI89] | 79.05 | 2 | 1259 1561 |  |  |
|  | [89107938](http://www.ncbi.nlm.nih.gov/entrez/query.fcgi?cmd=Search&db=Protein&term=89107938&doptcmdl=GenPept) | malonyl-CoA-[acyl-carrier-protein] transacylase [Escherichia coli W3110] | 79.05 | 2 | 1259 1561 |  |  |
|  | [77416724](http://www.ncbi.nlm.nih.gov/entrez/query.fcgi?cmd=Search&db=Protein&term=77416724&doptcmdl=GenPept) | Malonyl CoA-acyl carrier protein transacylase (MCT) | 79.05 | 2 | 1259 1561 |  |  |
|  | [77416723](http://www.ncbi.nlm.nih.gov/entrez/query.fcgi?cmd=Search&db=Protein&term=77416723&doptcmdl=GenPept) | Malonyl CoA-acyl carrier protein transacylase (MCT) | 79.05 | 2 | 1259 1561 |  |  |
| 300 | [91212626](http://www.ncbi.nlm.nih.gov/entrez/query.fcgi?cmd=Search&db=Protein&term=91212626&doptcmdl=GenPept) | putative ABC transporter ATP-binding protein YhbG [Escherichia coli UTI89] | 78.16 | 1 | 153 |  |  |
|  | [91074200](http://www.ncbi.nlm.nih.gov/entrez/query.fcgi?cmd=Search&db=Protein&term=91074200&doptcmdl=GenPept) | putative ATP-binding component of a transport system [Escherichia coli UTI89] | 78.16 | 1 | 153 |  |  |
|  | [89109964](http://www.ncbi.nlm.nih.gov/entrez/query.fcgi?cmd=Search&db=Protein&term=89109964&doptcmdl=GenPept) | predicted transporter subunit [Escherichia coli W3110] | 78.16 | 1 | 153 |  |  |
|  | [85675995](http://www.ncbi.nlm.nih.gov/entrez/query.fcgi?cmd=Search&db=Protein&term=85675995&doptcmdl=GenPept) | predicted transporter subunit [Escherichia coli W3110] | 78.16 | 1 | 153 |  |  |
|  | [83585421](http://www.ncbi.nlm.nih.gov/entrez/query.fcgi?cmd=Search&db=Protein&term=83585421&doptcmdl=GenPept) | COG1137: ABC-type (unclassified) transport system, ATPase component [Escherichia coli 101-1] | 78.16 | 1 | 153 |  |  |
| 301 | [91211639](http://www.ncbi.nlm.nih.gov/entrez/query.fcgi?cmd=Search&db=Protein&term=91211639&doptcmdl=GenPept) | hypothetical protein UTI89_C2627 [Escherichia coli UTI89] | 78.00 | 1 | 944 |  |  |
|  | [91073213](http://www.ncbi.nlm.nih.gov/entrez/query.fcgi?cmd=Search&db=Protein&term=91073213&doptcmdl=GenPept) | hypothetical protein UTI89_C2627 [Escherichia coli UTI89] | 78.00 | 1 | 944 |  |  |
|  | [90111421](http://www.ncbi.nlm.nih.gov/entrez/query.fcgi?cmd=Search&db=Protein&term=90111421&doptcmdl=GenPept) | conserved protein [Escherichia coli str. K-12 substr. MG1655] | 78.00 | 1 | 944 |  |  |
|  | [89109163](http://www.ncbi.nlm.nih.gov/entrez/query.fcgi?cmd=Search&db=Protein&term=89109163&doptcmdl=GenPept) | hypothetical protein [Escherichia coli W3110] | 78.00 | 1 | 944 |  |  |
|  | [87082087](http://www.ncbi.nlm.nih.gov/entrez/query.fcgi?cmd=Search&db=Protein&term=87082087&doptcmdl=GenPept) | conserved protein [Escherichia coli str. K-12 substr. MG1655] | 78.00 | 1 | 944 |  |  |
| 302 | [91210793](http://www.ncbi.nlm.nih.gov/entrez/query.fcgi?cmd=Search&db=Protein&term=91210793&doptcmdl=GenPept) | hypothetical protein YnfB precursor [Escherichia coli UTI89] | 76.54 | 1 | 385 |  |  |
|  | [91072367](http://www.ncbi.nlm.nih.gov/entrez/query.fcgi?cmd=Search&db=Protein&term=91072367&doptcmdl=GenPept) | hypothetical protein YnfB precursor [Escherichia coli UTI89] | 76.54 | 1 | 385 |  |  |
|  | [89108424](http://www.ncbi.nlm.nih.gov/entrez/query.fcgi?cmd=Search&db=Protein&term=89108424&doptcmdl=GenPept) | hypothetical protein [Escherichia coli W3110] | 76.54 | 1 | 385 |  |  |
|  | [85675032](http://www.ncbi.nlm.nih.gov/entrez/query.fcgi?cmd=Search&db=Protein&term=85675032&doptcmdl=GenPept) | hypothetical protein [Escherichia coli W3110] | 76.54 | 1 | 385 |  |  |
|  | [83587918](http://www.ncbi.nlm.nih.gov/entrez/query.fcgi?cmd=Search&db=Protein&term=83587918&doptcmdl=GenPept) | hypothetical protein Ecol1_01000951 [Escherichia coli 101-1] | 76.54 | 1 | 385 |  |  |
| 303 | [91210263](http://www.ncbi.nlm.nih.gov/entrez/query.fcgi?cmd=Search&db=Protein&term=91210263&doptcmdl=GenPept) | hypothetical protein UTI89_C1236 [Escherichia coli UTI89] | 75.71 | 2 | 634 1457 |  |  |
|  | [91071837](http://www.ncbi.nlm.nih.gov/entrez/query.fcgi?cmd=Search&db=Protein&term=91071837&doptcmdl=GenPept) | hypothetical protein YcfP [Escherichia coli UTI89] | 75.71 | 2 | 634 1457 |  |  |
|  | [90111213](http://www.ncbi.nlm.nih.gov/entrez/query.fcgi?cmd=Search&db=Protein&term=90111213&doptcmdl=GenPept) | conserved protein [Escherichia coli str. K-12 substr. MG1655] | 75.71 | 2 | 634 1457 |  |  |
|  | [89107954](http://www.ncbi.nlm.nih.gov/entrez/query.fcgi?cmd=Search&db=Protein&term=89107954&doptcmdl=GenPept) | hypothetical protein [Escherichia coli W3110] | 75.71 | 2 | 634 1457 |  |  |
|  | [87081832](http://www.ncbi.nlm.nih.gov/entrez/query.fcgi?cmd=Search&db=Protein&term=87081832&doptcmdl=GenPept) | conserved protein [Escherichia coli str. K-12 substr. MG1655] | 75.71 | 2 | 634 1457 |  |  |
| 304 | [91212862](http://www.ncbi.nlm.nih.gov/entrez/query.fcgi?cmd=Search&db=Protein&term=91212862&doptcmdl=GenPept) | shikimate kinase I [Escherichia coli UTI89] | 75.22 | 2 | 555 849 |  |  |
|  | [91074436](http://www.ncbi.nlm.nih.gov/entrez/query.fcgi?cmd=Search&db=Protein&term=91074436&doptcmdl=GenPept) | shikimate kinase I [Escherichia coli UTI89] | 75.22 | 2 | 555 849 |  |  |
|  | [90111581](http://www.ncbi.nlm.nih.gov/entrez/query.fcgi?cmd=Search&db=Protein&term=90111581&doptcmdl=GenPept) | shikimate kinase I [Escherichia coli str. K-12 substr. MG1655] | 75.22 | 2 | 555 849 |  |  |
|  | [89110620](http://www.ncbi.nlm.nih.gov/entrez/query.fcgi?cmd=Search&db=Protein&term=89110620&doptcmdl=GenPept) | shikimate kinase I [Escherichia coli W3110] | 75.22 | 2 | 555 849 |  |  |
|  | [87082255](http://www.ncbi.nlm.nih.gov/entrez/query.fcgi?cmd=Search&db=Protein&term=87082255&doptcmdl=GenPept) | shikimate kinase I [Escherichia coli str. K-12 substr. MG1655] | 75.22 | 2 | 555 849 |  |  |
| 305 | [91210287](http://www.ncbi.nlm.nih.gov/entrez/query.fcgi?cmd=Search&db=Protein&term=91210287&doptcmdl=GenPept) | adenylosuccinate lyase [Escherichia coli UTI89] | 74.23 | 2 | 788 1599 |  |  |
|  | [91071861](http://www.ncbi.nlm.nih.gov/entrez/query.fcgi?cmd=Search&db=Protein&term=91071861&doptcmdl=GenPept) | adenylosuccinate lyase [Escherichia coli UTI89] | 74.23 | 2 | 788 1599 |  |  |
|  | [89107977](http://www.ncbi.nlm.nih.gov/entrez/query.fcgi?cmd=Search&db=Protein&term=89107977&doptcmdl=GenPept) | adenylosuccinate lyase [Escherichia coli W3110] | 74.23 | 2 | 788 1599 |  |  |
|  | [83587303](http://www.ncbi.nlm.nih.gov/entrez/query.fcgi?cmd=Search&db=Protein&term=83587303&doptcmdl=GenPept) | COG0015: Adenylosuccinate lyase [Escherichia coli 101-1] | 74.23 | 2 | 788 1599 |  |  |
|  | [82544382](http://www.ncbi.nlm.nih.gov/entrez/query.fcgi?cmd=Search&db=Protein&term=82544382&doptcmdl=GenPept) | adenylosuccinate lyase [Shigella boydii Sb227] | 74.23 | 2 | 788 1599 |  |  |
| 306 | [91210785](http://www.ncbi.nlm.nih.gov/entrez/query.fcgi?cmd=Search&db=Protein&term=91210785&doptcmdl=GenPept) | L-allo-threonine dehydrogenase, NAD(P)-binding [Escherichia coli UTI89] | 73.94 | 1 | 1213 |  |  |
|  | [91072359](http://www.ncbi.nlm.nih.gov/entrez/query.fcgi?cmd=Search&db=Protein&term=91072359&doptcmdl=GenPept) | probable oxidoreductase YdfG [Escherichia coli UTI89] [MASS=27850] | 73.94 | 1 | 1213 |  |  |
|  | [89108380](http://www.ncbi.nlm.nih.gov/entrez/query.fcgi?cmd=Search&db=Protein&term=89108380&doptcmdl=GenPept) | L-allo-threonine dehydrogenase, NAD(P)-binding [Escherichia coli W3110] | 73.94 | 1 | 1213 |  |  |
|  | [83587890](http://www.ncbi.nlm.nih.gov/entrez/query.fcgi?cmd=Search&db=Protein&term=83587890&doptcmdl=GenPept) | COG4221: Short-chain alcohol dehydrogenase of unknown specificity [Escherichia coli 101-1] | 73.94 | 1 | 1213 |  |  |
|  | [82776857](http://www.ncbi.nlm.nih.gov/entrez/query.fcgi?cmd=Search&db=Protein&term=82776857&doptcmdl=GenPept) | 3-hydroxy acid dehydrogenase [Shigella dysenteriae Sd197] | 73.94 | 1 | 1213 |  |  |
| 307 | [91213233](http://www.ncbi.nlm.nih.gov/entrez/query.fcgi?cmd=Search&db=Protein&term=91213233&doptcmdl=GenPept) | tryptophanase [Escherichia coli UTI89] | 73.75 | 1 | 1501 |  |  |
|  | [91074807](http://www.ncbi.nlm.nih.gov/entrez/query.fcgi?cmd=Search&db=Protein&term=91074807&doptcmdl=GenPept) | tryptophanase [Escherichia coli UTI89] | 73.75 | 1 | 1501 |  |  |
|  | [90111643](http://www.ncbi.nlm.nih.gov/entrez/query.fcgi?cmd=Search&db=Protein&term=90111643&doptcmdl=GenPept) | tryptophanase/L-cysteine desulfhydrase, PLP-dependent [Escherichia coli str. K-12 substr. MG1655] | 73.75 | 1 | 1501 |  |  |
|  | [89110303](http://www.ncbi.nlm.nih.gov/entrez/query.fcgi?cmd=Search&db=Protein&term=89110303&doptcmdl=GenPept) | tryptophanase/L-cysteine desulfhydrase, PLP-dependent [Escherichia coli W3110] | 73.75 | 1 | 1501 |  |  |
|  | [87082323](http://www.ncbi.nlm.nih.gov/entrez/query.fcgi?cmd=Search&db=Protein&term=87082323&doptcmdl=GenPept) | tryptophanase/L-cysteine desulfhydrase, PLP-dependent [Escherichia coli str. K-12 substr. MG1655] | 73.75 | 1 | 1501 |  |  |
| 308 | [999531](http://www.ncbi.nlm.nih.gov/entrez/query.fcgi?cmd=Search&db=Protein&term=999531&doptcmdl=GenPept) | Chain A, Structural Basis For Transfer Rna Aminoaceylation By Escherichia Coli Glutaminyl-Trna Synthetase | 72.73 | 2 | 141 1651 |  |  |
|  | [999529](http://www.ncbi.nlm.nih.gov/entrez/query.fcgi?cmd=Search&db=Protein&term=999529&doptcmdl=GenPept) | Chain A, Structural Basis Of Anticodon Loop Recognition By Glutaminyl-Trna Synthetase | 72.73 | 2 | 141 1651 |  |  |
|  | [91209722](http://www.ncbi.nlm.nih.gov/entrez/query.fcgi?cmd=Search&db=Protein&term=91209722&doptcmdl=GenPept) | glutaminyl-tRNA synthetase [Escherichia coli UTI89] | 72.73 | 2 | 141 1651 |  |  |
|  | [91071296](http://www.ncbi.nlm.nih.gov/entrez/query.fcgi?cmd=Search&db=Protein&term=91071296&doptcmdl=GenPept) | glutaminyl-tRNA synthetase [Escherichia coli UTI89] | 72.73 | 2 | 141 1651 |  |  |
|  | [89107538](http://www.ncbi.nlm.nih.gov/entrez/query.fcgi?cmd=Search&db=Protein&term=89107538&doptcmdl=GenPept) | glutamyl-tRNA synthetase [Escherichia coli W3110] | 72.73 | 2 | 141 1651 |  |  |

| **Protein IDs*, cont.*** | | | | | | | |
| --- | --- | --- | --- | --- | --- | --- | --- |
| *Grp Nr.* | *Accession Number* | *Protein Name* | *Protein Score* | *Unique PSMs* | *PSM Serial Nrs.* | *Other Grp.* | *Score (other)* |
| 309 | [91212652](http://www.ncbi.nlm.nih.gov/entrez/query.fcgi?cmd=Search&db=Protein&term=91212652&doptcmdl=GenPept) | cytochrome d ubiquinol oxidase subunit III [Escherichia coli UTI89] | 72.67 | 2 | 640 1521 |  |  |
|  | [91074226](http://www.ncbi.nlm.nih.gov/entrez/query.fcgi?cmd=Search&db=Protein&term=91074226&doptcmdl=GenPept) | hypothetical protein UTI89_C3663 [Escherichia coli UTI89] | 72.67 | 2 | 640 1521 |  |  |
|  | [90111560](http://www.ncbi.nlm.nih.gov/entrez/query.fcgi?cmd=Search&db=Protein&term=90111560&doptcmdl=GenPept) | conserved protein [Escherichia coli str. K-12 substr. MG1655] | 72.67 | 2 | 640 1521 |  |  |
|  | [89109995](http://www.ncbi.nlm.nih.gov/entrez/query.fcgi?cmd=Search&db=Protein&term=89109995&doptcmdl=GenPept) | hypothetical protein [Escherichia coli W3110] | 72.67 | 2 | 640 1521 |  |  |
|  | [87082232](http://www.ncbi.nlm.nih.gov/entrez/query.fcgi?cmd=Search&db=Protein&term=87082232&doptcmdl=GenPept) | conserved protein [Escherichia coli str. K-12 substr. MG1655] | 72.67 | 2 | 640 1521 |  |  |
| 310 | [91212475](http://www.ncbi.nlm.nih.gov/entrez/query.fcgi?cmd=Search&db=Protein&term=91212475&doptcmdl=GenPept) | 3,4-dihydroxy-2-butanone 4-phosphate synthase [Escherichia coli UTI89] | 72.47 | 1 | 740 |  |  |
|  | [91074049](http://www.ncbi.nlm.nih.gov/entrez/query.fcgi?cmd=Search&db=Protein&term=91074049&doptcmdl=GenPept) | 3,4-dihydroxy-2-butanone 4-phosphate synthase [Escherichia coli UTI89] | 72.47 | 1 | 740 |  |  |
|  | [89109811](http://www.ncbi.nlm.nih.gov/entrez/query.fcgi?cmd=Search&db=Protein&term=89109811&doptcmdl=GenPept) | 3,4 dihydroxy-2-butanone-4-phosphate synthase [Escherichia coli W3110] | 72.47 | 1 | 740 |  |  |
|  | [882571](http://www.ncbi.nlm.nih.gov/entrez/query.fcgi?cmd=Search&db=Protein&term=882571&doptcmdl=GenPept) | 3,4-dihydroxy-2-butanone 4-phosphate synthase | 72.47 | 1 | 740 |  |  |
|  | [85675844](http://www.ncbi.nlm.nih.gov/entrez/query.fcgi?cmd=Search&db=Protein&term=85675844&doptcmdl=GenPept) | 3,4 dihydroxy-2-butanone-4-phosphate synthase [Escherichia coli W3110] | 72.47 | 1 | 740 |  |  |
| 311 | [91212922](http://www.ncbi.nlm.nih.gov/entrez/query.fcgi?cmd=Search&db=Protein&term=91212922&doptcmdl=GenPept) | putative oxidoreductase YhhX [Escherichia coli UTI89] | 72.35 | 1 | 557 |  |  |
|  | [91074496](http://www.ncbi.nlm.nih.gov/entrez/query.fcgi?cmd=Search&db=Protein&term=91074496&doptcmdl=GenPept) | putative oxidoreductase YhhX [Escherichia coli UTI89] | 72.35 | 1 | 557 |  |  |
|  | [89110572](http://www.ncbi.nlm.nih.gov/entrez/query.fcgi?cmd=Search&db=Protein&term=89110572&doptcmdl=GenPept) | predicted oxidoreductase with NAD(P)-binding Rossmann-fold domain [Escherichia coli W3110] | 72.35 | 1 | 557 |  |  |
|  | [85676603](http://www.ncbi.nlm.nih.gov/entrez/query.fcgi?cmd=Search&db=Protein&term=85676603&doptcmdl=GenPept) | predicted oxidoreductase with NAD(P)-binding Rossmann-fold domain [Escherichia coli W3110] | 72.35 | 1 | 557 |  |  |
|  | [83588694](http://www.ncbi.nlm.nih.gov/entrez/query.fcgi?cmd=Search&db=Protein&term=83588694&doptcmdl=GenPept) | COG0673: Predicted dehydrogenases and related proteins [Escherichia coli 101-1] | 72.35 | 1 | 557 |  |  |
| 312 | [91210258](http://www.ncbi.nlm.nih.gov/entrez/query.fcgi?cmd=Search&db=Protein&term=91210258&doptcmdl=GenPept) | purine nucleoside phosphoramidase [Escherichia coli UTI89] | 71.93 | 1 | 528 |  |  |
|  | [91071832](http://www.ncbi.nlm.nih.gov/entrez/query.fcgi?cmd=Search&db=Protein&term=91071832&doptcmdl=GenPept) | HIT-like protein YcfF [Escherichia coli UTI89] | 71.93 | 1 | 528 |  |  |
|  | [89107949](http://www.ncbi.nlm.nih.gov/entrez/query.fcgi?cmd=Search&db=Protein&term=89107949&doptcmdl=GenPept) | purine nucleoside phosphoramidase [Escherichia coli W3110] | 71.93 | 1 | 528 |  |  |
|  | [83584870](http://www.ncbi.nlm.nih.gov/entrez/query.fcgi?cmd=Search&db=Protein&term=83584870&doptcmdl=GenPept) | COG0537: Diadenosine tetraphosphate (Ap4A) hydrolase and other HIT family hydrolases [Escherichia coli 101-1] [MASS=13255] | 71.93 | 1 | 528 |  |  |
|  | [83569575](http://www.ncbi.nlm.nih.gov/entrez/query.fcgi?cmd=Search&db=Protein&term=83569575&doptcmdl=GenPept) | COG0537: Diadenosine tetraphosphate (Ap4A) hydrolase and other HIT family hydrolases [Shigella dysenteriae 1012] | 71.93 | 1 | 528 |  |  |
| 313 | [91210880](http://www.ncbi.nlm.nih.gov/entrez/query.fcgi?cmd=Search&db=Protein&term=91210880&doptcmdl=GenPept) | hypothetical protein UTI89_C1859 [Escherichia coli UTI89] | 71.79 | 1 | 1178 |  |  |
|  | [91072454](http://www.ncbi.nlm.nih.gov/entrez/query.fcgi?cmd=Search&db=Protein&term=91072454&doptcmdl=GenPept) | hypothetical protein UTI89_C1859 [Escherichia coli UTI89] [MASS=11299] | 71.79 | 1 | 1178 |  |  |
|  | [89108507](http://www.ncbi.nlm.nih.gov/entrez/query.fcgi?cmd=Search&db=Protein&term=89108507&doptcmdl=GenPept) | hypothetical protein [Escherichia coli W3110] | 71.79 | 1 | 1178 |  |  |
|  | [85675066](http://www.ncbi.nlm.nih.gov/entrez/query.fcgi?cmd=Search&db=Protein&term=85675066&doptcmdl=GenPept) | hypothetical protein [Escherichia coli W3110] | 71.79 | 1 | 1178 |  |  |
|  | [83754037](http://www.ncbi.nlm.nih.gov/entrez/query.fcgi?cmd=Search&db=Protein&term=83754037&doptcmdl=GenPept) | Chain B, Solution Structure Of Ydhr Protein From Escherichia Coli [MASS=13863] | 71.79 | 1 | 1178 |  |  |
| 314 | [91209746](http://www.ncbi.nlm.nih.gov/entrez/query.fcgi?cmd=Search&db=Protein&term=91209746&doptcmdl=GenPept) | putative hydrolase-oxidase [Escherichia coli UTI89] | 71.68 | 1 | 1064 |  |  |
|  | [91071320](http://www.ncbi.nlm.nih.gov/entrez/query.fcgi?cmd=Search&db=Protein&term=91071320&doptcmdl=GenPept) | conserved hypothetical protein [Escherichia coli UTI89] | 71.68 | 1 | 1064 |  |  |
|  | [89107568](http://www.ncbi.nlm.nih.gov/entrez/query.fcgi?cmd=Search&db=Protein&term=89107568&doptcmdl=GenPept) | conserved metal-binding protein [Escherichia coli W3110] | 71.68 | 1 | 1064 |  |  |
|  | [84027846](http://www.ncbi.nlm.nih.gov/entrez/query.fcgi?cmd=Search&db=Protein&term=84027846&doptcmdl=GenPept) | UPF0135 protein ybgI | 71.68 | 1 | 1064 |  |  |
|  | [84027845](http://www.ncbi.nlm.nih.gov/entrez/query.fcgi?cmd=Search&db=Protein&term=84027845&doptcmdl=GenPept) | UPF0135 protein ybgI | 71.68 | 1 | 1064 |  |  |
| 315 | [91213292](http://www.ncbi.nlm.nih.gov/entrez/query.fcgi?cmd=Search&db=Protein&term=91213292&doptcmdl=GenPept) | branched-chain amino acid aminotransferase [Escherichia coli UTI89] | 71.65 | 1 | 5 |  |  |
|  | [91074866](http://www.ncbi.nlm.nih.gov/entrez/query.fcgi?cmd=Search&db=Protein&term=91074866&doptcmdl=GenPept) | branched-chain amino acid aminotransferase [Escherichia coli UTI89] | 71.65 | 1 | 5 |  |  |
|  | [89110246](http://www.ncbi.nlm.nih.gov/entrez/query.fcgi?cmd=Search&db=Protein&term=89110246&doptcmdl=GenPept) | branched-chain amino-acid aminotransferase [Escherichia coli W3110] | 71.65 | 1 | 5 |  |  |
|  | [87117962](http://www.ncbi.nlm.nih.gov/entrez/query.fcgi?cmd=Search&db=Protein&term=87117962&doptcmdl=GenPept) | IlvE [Escherichia coli] [MASS=33041] | 71.65 | 1 | 5 |  |  |
|  | [87117960](http://www.ncbi.nlm.nih.gov/entrez/query.fcgi?cmd=Search&db=Protein&term=87117960&doptcmdl=GenPept) | IlvE [Escherichia coli] | 71.65 | 1 | 5 |  |  |
| 316 | [91213301](http://www.ncbi.nlm.nih.gov/entrez/query.fcgi?cmd=Search&db=Protein&term=91213301&doptcmdl=GenPept) | thioredoxin [Escherichia coli UTI89] | 71.53 | 1 | 437 |  |  |
|  | [91074875](http://www.ncbi.nlm.nih.gov/entrez/query.fcgi?cmd=Search&db=Protein&term=91074875&doptcmdl=GenPept) | thioredoxin 1 [Escherichia coli UTI89] | 71.53 | 1 | 437 |  |  |
|  | [89110236](http://www.ncbi.nlm.nih.gov/entrez/query.fcgi?cmd=Search&db=Protein&term=89110236&doptcmdl=GenPept) | thioredoxin 1 [Escherichia coli W3110] | 71.53 | 1 | 437 |  |  |
|  | [88942920](http://www.ncbi.nlm.nih.gov/entrez/query.fcgi?cmd=Search&db=Protein&term=88942920&doptcmdl=GenPept) | Thioredoxin-1 (Trx-1) (Trx) | 71.53 | 1 | 437 |  |  |
|  | [88942919](http://www.ncbi.nlm.nih.gov/entrez/query.fcgi?cmd=Search&db=Protein&term=88942919&doptcmdl=GenPept) | Thioredoxin-1 (Trx-1) (Trx) | 71.53 | 1 | 437 |  |  |
| 317 | [91209113](http://www.ncbi.nlm.nih.gov/entrez/query.fcgi?cmd=Search&db=Protein&term=91209113&doptcmdl=GenPept) | dimethyladenosine transferase [Escherichia coli UTI89] | 70.63 | 1 | 178 |  |  |
|  | [91070687](http://www.ncbi.nlm.nih.gov/entrez/query.fcgi?cmd=Search&db=Protein&term=91070687&doptcmdl=GenPept) | dimethyladenosine transferase [Escherichia coli UTI89] | 70.63 | 1 | 178 |  |  |
|  | [89106935](http://www.ncbi.nlm.nih.gov/entrez/query.fcgi?cmd=Search&db=Protein&term=89106935&doptcmdl=GenPept) | S-adenosylmethionine-6-N',N'-adenosyl (rRNA) dimethyltransferase [Escherichia coli W3110] | 70.63 | 1 | 178 |  |  |
|  | [86516105](http://www.ncbi.nlm.nih.gov/entrez/query.fcgi?cmd=Search&db=Protein&term=86516105&doptcmdl=GenPept) | KsgA [Escherichia coli] [MASS=28420] | 70.63 | 1 | 178 |  |  |
|  | [86516103](http://www.ncbi.nlm.nih.gov/entrez/query.fcgi?cmd=Search&db=Protein&term=86516103&doptcmdl=GenPept) | KsgA [Escherichia coli] | 70.63 | 1 | 178 |  |  |
| 318 | [91212749](http://www.ncbi.nlm.nih.gov/entrez/query.fcgi?cmd=Search&db=Protein&term=91212749&doptcmdl=GenPept) | 50S ribosomal protein L22 [Escherichia coli UTI89] | 70.28 | 2 | 828 1044 |  |  |
|  | [91074323](http://www.ncbi.nlm.nih.gov/entrez/query.fcgi?cmd=Search&db=Protein&term=91074323&doptcmdl=GenPept) | 50S ribosomal subunit protein L22 [Escherichia coli UTI89] | 70.28 | 2 | 828 1044 |  |  |
|  | [89110695](http://www.ncbi.nlm.nih.gov/entrez/query.fcgi?cmd=Search&db=Protein&term=89110695&doptcmdl=GenPept) | 50S ribosomal subunit protein L22 [Escherichia coli W3110] | 70.28 | 2 | 828 1044 |  |  |
|  | [85676726](http://www.ncbi.nlm.nih.gov/entrez/query.fcgi?cmd=Search&db=Protein&term=85676726&doptcmdl=GenPept) | 50S ribosomal subunit protein L22 [Escherichia coli W3110] | 70.28 | 2 | 828 1044 |  |  |
|  | [83754135](http://www.ncbi.nlm.nih.gov/entrez/query.fcgi?cmd=Search&db=Protein&term=83754135&doptcmdl=GenPept) | Chain S, Crystal Structure Of The Bacterial Ribosome From Escherichia Coli At 3.5 A Resolution. This File Contains The 50s Subunit Of The Second 70s Ribosome. The Entire Crystal Structure Contains Two 70s Ribosomes And Is Described In Remark 400. | 70.28 | 2 | 828 1044 |  |  |
| 319 | [91211808](http://www.ncbi.nlm.nih.gov/entrez/query.fcgi?cmd=Search&db=Protein&term=91211808&doptcmdl=GenPept) | phosphoribosylaminoimidazole-succinocarboxamide synthase [Escherichia coli UTI89] | 69.69 | 1 | 1654 |  |  |
|  | [91073382](http://www.ncbi.nlm.nih.gov/entrez/query.fcgi?cmd=Search&db=Protein&term=91073382&doptcmdl=GenPept) | phosphoribosylaminoimidazole-succinocarboxamide synthetase [Escherichia coli UTI89] | 69.69 | 1 | 1654 |  |  |
|  | [89109282](http://www.ncbi.nlm.nih.gov/entrez/query.fcgi?cmd=Search&db=Protein&term=89109282&doptcmdl=GenPept) | phosphoribosylaminoimidazole-succinocarboxamide synthetase [Escherichia coli W3110] | 69.69 | 1 | 1654 |  |  |
|  | [83586875](http://www.ncbi.nlm.nih.gov/entrez/query.fcgi?cmd=Search&db=Protein&term=83586875&doptcmdl=GenPept) | COG0152: Phosphoribosylaminoimidazolesuccinocarboxamide (SAICAR) synthase [Escherichia coli 101-1] | 69.69 | 1 | 1654 |  |  |
|  | [75211441](http://www.ncbi.nlm.nih.gov/entrez/query.fcgi?cmd=Search&db=Protein&term=75211441&doptcmdl=GenPept) | COG0152: Phosphoribosylaminoimidazolesuccinocarboxamide (SAICAR) synthase [Escherichia coli B171] | 69.69 | 1 | 1654 |  |  |
| 320 | [91209065](http://www.ncbi.nlm.nih.gov/entrez/query.fcgi?cmd=Search&db=Protein&term=91209065&doptcmdl=GenPept) | molybdenum cofactor biosynthesis protein [Escherichia coli UTI89] | 69.67 | 2 | 215 1359 |  |  |
|  | [91070639](http://www.ncbi.nlm.nih.gov/entrez/query.fcgi?cmd=Search&db=Protein&term=91070639&doptcmdl=GenPept) | molybdopterin biosynthesis mog protein [Escherichia coli UTI89] [MASS=21367] | 69.67 | 2 | 215 1359 |  |  |
|  | [89106893](http://www.ncbi.nlm.nih.gov/entrez/query.fcgi?cmd=Search&db=Protein&term=89106893&doptcmdl=GenPept) | predicted molybdochelatase [Escherichia coli W3110] | 69.67 | 2 | 215 1359 |  |  |
|  | [85674278](http://www.ncbi.nlm.nih.gov/entrez/query.fcgi?cmd=Search&db=Protein&term=85674278&doptcmdl=GenPept) | predicted molybdochelatase [Escherichia coli W3110] | 69.67 | 2 | 215 1359 |  |  |
|  | [84028147](http://www.ncbi.nlm.nih.gov/entrez/query.fcgi?cmd=Search&db=Protein&term=84028147&doptcmdl=GenPept) | Molybdopterin biosynthesis mog protein | 69.67 | 2 | 215 1359 |  |  |
| 321 | [91210997](http://www.ncbi.nlm.nih.gov/entrez/query.fcgi?cmd=Search&db=Protein&term=91210997&doptcmdl=GenPept) | conserved protein YeaD [Escherichia coli UTI89] | 69.51 | 1 | 52 |  |  |
|  | [91072571](http://www.ncbi.nlm.nih.gov/entrez/query.fcgi?cmd=Search&db=Protein&term=91072571&doptcmdl=GenPept) | conserved protein YeaD [Escherichia coli UTI89] | 69.51 | 1 | 52 |  |  |
|  | [90111330](http://www.ncbi.nlm.nih.gov/entrez/query.fcgi?cmd=Search&db=Protein&term=90111330&doptcmdl=GenPept) | conserved protein [Escherichia coli str. K-12 substr. MG1655] | 69.51 | 1 | 52 |  |  |
|  | [89108619](http://www.ncbi.nlm.nih.gov/entrez/query.fcgi?cmd=Search&db=Protein&term=89108619&doptcmdl=GenPept) | hypothetical protein [Escherichia coli W3110] | 69.51 | 1 | 52 |  |  |
|  | [87081973](http://www.ncbi.nlm.nih.gov/entrez/query.fcgi?cmd=Search&db=Protein&term=87081973&doptcmdl=GenPept) | conserved protein [Escherichia coli str. K-12 substr. MG1655] | 69.51 | 1 | 52 |  |  |
| 322 | [91209326](http://www.ncbi.nlm.nih.gov/entrez/query.fcgi?cmd=Search&db=Protein&term=91209326&doptcmdl=GenPept) | xanthine-guanine phosphoribosyltransferase [Escherichia coli UTI89] | 69.12 | 1 | 425 |  |  |
|  | [91070900](http://www.ncbi.nlm.nih.gov/entrez/query.fcgi?cmd=Search&db=Protein&term=91070900&doptcmdl=GenPept) | xanthine-guanine phosphoribosyltransferase [Escherichia coli UTI89] | 69.12 | 1 | 425 |  |  |
|  | [89107111](http://www.ncbi.nlm.nih.gov/entrez/query.fcgi?cmd=Search&db=Protein&term=89107111&doptcmdl=GenPept) | guanine-hypoxanthine phosphoribosyltransferase [Escherichia coli W3110] | 69.12 | 1 | 425 |  |  |
|  | [84027729](http://www.ncbi.nlm.nih.gov/entrez/query.fcgi?cmd=Search&db=Protein&term=84027729&doptcmdl=GenPept) | Xanthine phosphoribosyltransferase (Xanthine-guanine phosphoribosyltransferase) (XGPRT) | 69.12 | 1 | 425 |  |  |
|  | [83586314](http://www.ncbi.nlm.nih.gov/entrez/query.fcgi?cmd=Search&db=Protein&term=83586314&doptcmdl=GenPept) | COG0503: Adenine/guanine phosphoribosyltransferases and related PRPP-binding proteins [Escherichia coli 101-1] | 69.12 | 1 | 425 |  |  |
| 323 | [91212889](http://www.ncbi.nlm.nih.gov/entrez/query.fcgi?cmd=Search&db=Protein&term=91212889&doptcmdl=GenPept) | predicted gluconate transport-associated protein [Escherichia coli UTI89] | 68.24 | 1 | 458 |  |  |
|  | [91074463](http://www.ncbi.nlm.nih.gov/entrez/query.fcgi?cmd=Search&db=Protein&term=91074463&doptcmdl=GenPept) | hypothetical protein UTI89_C3915 [Escherichia coli UTI89] | 68.24 | 1 | 458 |  |  |
|  | [89110596](http://www.ncbi.nlm.nih.gov/entrez/query.fcgi?cmd=Search&db=Protein&term=89110596&doptcmdl=GenPept) | predicted gluconate transport associated protein [Escherichia coli W3110] | 68.24 | 1 | 458 |  |  |
|  | [85676627](http://www.ncbi.nlm.nih.gov/entrez/query.fcgi?cmd=Search&db=Protein&term=85676627&doptcmdl=GenPept) | predicted gluconate transport associated protein [Escherichia coli W3110] | 68.24 | 1 | 458 |  |  |
|  | [83588721](http://www.ncbi.nlm.nih.gov/entrez/query.fcgi?cmd=Search&db=Protein&term=83588721&doptcmdl=GenPept) | COG0316: Uncharacterized conserved protein [Escherichia coli 101-1] | 68.24 | 1 | 458 |  |  |
| 324 | [91211440](http://www.ncbi.nlm.nih.gov/entrez/query.fcgi?cmd=Search&db=Protein&term=91211440&doptcmdl=GenPept) | GTP cyclohydrolase I [Escherichia coli UTI89] | 68.22 | 1 | 1291 |  |  |
|  | [91073014](http://www.ncbi.nlm.nih.gov/entrez/query.fcgi?cmd=Search&db=Protein&term=91073014&doptcmdl=GenPept) | FolE subunit of GTP cyclohydrolase I [Escherichia coli UTI89] | 68.22 | 1 | 1291 |  |  |
|  | [89108970](http://www.ncbi.nlm.nih.gov/entrez/query.fcgi?cmd=Search&db=Protein&term=89108970&doptcmdl=GenPept) | GTP cyclohydrolase I [Escherichia coli W3110] | 68.22 | 1 | 1291 |  |  |
|  | [85675267](http://www.ncbi.nlm.nih.gov/entrez/query.fcgi?cmd=Search&db=Protein&term=85675267&doptcmdl=GenPept) | GTP cyclohydrolase I [Escherichia coli W3110] | 68.22 | 1 | 1291 |  |  |
|  | [83588134](http://www.ncbi.nlm.nih.gov/entrez/query.fcgi?cmd=Search&db=Protein&term=83588134&doptcmdl=GenPept) | COG0302: GTP cyclohydrolase I [Escherichia coli 101-1] | 68.22 | 1 | 1291 |  |  |
| 325 | [91212825](http://www.ncbi.nlm.nih.gov/entrez/query.fcgi?cmd=Search&db=Protein&term=91212825&doptcmdl=GenPept) | FKBP-type peptidyl-prolyl cis-trans isomerase (rotamase) [Escherichia coli UTI89] | 68.16 | 1 | 1082 |  |  |
|  | [91074399](http://www.ncbi.nlm.nih.gov/entrez/query.fcgi?cmd=Search&db=Protein&term=91074399&doptcmdl=GenPept) | FKBP-type peptidyl-prolyl cis-trans isomerase (rotamase) [Escherichia coli UTI89] | 68.16 | 1 | 1082 |  |  |
|  | [89110661](http://www.ncbi.nlm.nih.gov/entrez/query.fcgi?cmd=Search&db=Protein&term=89110661&doptcmdl=GenPept) | FKBP-type peptidyl prolyl cis-trans isomerase [Escherichia coli W3110] | 68.16 | 1 | 1082 |  |  |
|  | [862299](http://www.ncbi.nlm.nih.gov/entrez/query.fcgi?cmd=Search&db=Protein&term=862299&doptcmdl=GenPept) | slyD gene product | 68.16 | 1 | 1082 |  |  |
|  | [85676692](http://www.ncbi.nlm.nih.gov/entrez/query.fcgi?cmd=Search&db=Protein&term=85676692&doptcmdl=GenPept) | FKBP-type peptidyl prolyl cis-trans isomerase [Escherichia coli W3110] | 68.16 | 1 | 1082 |  |  |
| 326 | [91212574](http://www.ncbi.nlm.nih.gov/entrez/query.fcgi?cmd=Search&db=Protein&term=91212574&doptcmdl=GenPept) | Hypothetical acetyltransferase YhbS [Escherichia coli UTI89] | 68.09 | 1 | 1711 |  |  |
|  | [91074148](http://www.ncbi.nlm.nih.gov/entrez/query.fcgi?cmd=Search&db=Protein&term=91074148&doptcmdl=GenPept) | Hypothetical acetyltransferase YhbS [Escherichia coli UTI89] | 68.09 | 1 | 1711 |  |  |
|  | [89109921](http://www.ncbi.nlm.nih.gov/entrez/query.fcgi?cmd=Search&db=Protein&term=89109921&doptcmdl=GenPept) | predicted acyltransferase with acyl-CoA N-acyltransferase domain [Escherichia coli W3110] | 68.09 | 1 | 1711 |  |  |
|  | [85675952](http://www.ncbi.nlm.nih.gov/entrez/query.fcgi?cmd=Search&db=Protein&term=85675952&doptcmdl=GenPept) | predicted acyltransferase with acyl-CoA N-acyltransferase domain [Escherichia coli W3110] | 68.09 | 1 | 1711 |  |  |
|  | [83585797](http://www.ncbi.nlm.nih.gov/entrez/query.fcgi?cmd=Search&db=Protein&term=83585797&doptcmdl=GenPept) | COG3153: Predicted acetyltransferase [Escherichia coli 101-1] | 68.09 | 1 | 1711 |  |  |
| 327 | [91209666](http://www.ncbi.nlm.nih.gov/entrez/query.fcgi?cmd=Search&db=Protein&term=91209666&doptcmdl=GenPept) | citrate lyase subunit gamma [Escherichia coli UTI89] | 67.85 | 1 | 1469 |  |  |
|  | [91071240](http://www.ncbi.nlm.nih.gov/entrez/query.fcgi?cmd=Search&db=Protein&term=91071240&doptcmdl=GenPept) | citrate lyase acyl carrier protein (gamma chain) [Escherichia coli UTI89] | 67.85 | 1 | 1469 |  |  |
|  | [89107484](http://www.ncbi.nlm.nih.gov/entrez/query.fcgi?cmd=Search&db=Protein&term=89107484&doptcmdl=GenPept) | citrate lyase, acyl carrier (gamma) subunit [Escherichia coli W3110] | 67.85 | 1 | 1469 |  |  |
|  | [83586948](http://www.ncbi.nlm.nih.gov/entrez/query.fcgi?cmd=Search&db=Protein&term=83586948&doptcmdl=GenPept) | COG3052: Citrate lyase, gamma subunit [Escherichia coli 101-1] | 67.85 | 1 | 1469 |  |  |
|  | [75210755](http://www.ncbi.nlm.nih.gov/entrez/query.fcgi?cmd=Search&db=Protein&term=75210755&doptcmdl=GenPept) | COG3052: Citrate lyase, gamma subunit [Escherichia coli B171] | 67.85 | 1 | 1469 |  |  |
| 328 | [91213474](http://www.ncbi.nlm.nih.gov/entrez/query.fcgi?cmd=Search&db=Protein&term=91213474&doptcmdl=GenPept) | ATP-dependent protease peptidase subunit [Escherichia coli UTI89] | 67.59 | 1 | 749 |  |  |
|  | [91075048](http://www.ncbi.nlm.nih.gov/entrez/query.fcgi?cmd=Search&db=Protein&term=91075048&doptcmdl=GenPept) | ATP-dependent hslVU protease peptidase subunit hslV [Escherichia coli UTI89] | 67.59 | 1 | 749 |  |  |
|  | [89110097](http://www.ncbi.nlm.nih.gov/entrez/query.fcgi?cmd=Search&db=Protein&term=89110097&doptcmdl=GenPept) | peptidase component of the HslUV protease [Escherichia coli W3110] | 67.59 | 1 | 749 |  |  |
|  | [85676128](http://www.ncbi.nlm.nih.gov/entrez/query.fcgi?cmd=Search&db=Protein&term=85676128&doptcmdl=GenPept) | peptidase component of the HslUV protease [Escherichia coli W3110] | 67.59 | 1 | 749 |  |  |
|  | [83586224](http://www.ncbi.nlm.nih.gov/entrez/query.fcgi?cmd=Search&db=Protein&term=83586224&doptcmdl=GenPept) | COG5405: ATP-dependent protease HslVU (ClpYQ), peptidase subunit [Escherichia coli 101-1] | 67.59 | 1 | 749 |  |  |
| 329 | [91211049](http://www.ncbi.nlm.nih.gov/entrez/query.fcgi?cmd=Search&db=Protein&term=91211049&doptcmdl=GenPept) | transcriptional regulator KdgR [Escherichia coli UTI89] | 67.51 | 1 | 452 |  |  |
|  | [91072623](http://www.ncbi.nlm.nih.gov/entrez/query.fcgi?cmd=Search&db=Protein&term=91072623&doptcmdl=GenPept) | transcriptional regulator KdgR [Escherichia coli UTI89] | 67.51 | 1 | 452 |  |  |
|  | [89108667](http://www.ncbi.nlm.nih.gov/entrez/query.fcgi?cmd=Search&db=Protein&term=89108667&doptcmdl=GenPept) | predicted DNA-binding transcriptional regulator [Escherichia coli W3110] | 67.51 | 1 | 452 |  |  |
|  | [83584506](http://www.ncbi.nlm.nih.gov/entrez/query.fcgi?cmd=Search&db=Protein&term=83584506&doptcmdl=GenPept) | COG1414: Transcriptional regulator [Escherichia coli 101-1] | 67.51 | 1 | 452 |  |  |
|  | [82543749](http://www.ncbi.nlm.nih.gov/entrez/query.fcgi?cmd=Search&db=Protein&term=82543749&doptcmdl=GenPept) | putative regulator [Shigella boydii Sb227] | 67.51 | 1 | 452 |  |  |
| 330 | [91212216](http://www.ncbi.nlm.nih.gov/entrez/query.fcgi?cmd=Search&db=Protein&term=91212216&doptcmdl=GenPept) | protease III precursor [Escherichia coli UTI89] | 67.50 | 1 | 213 |  |  |
|  | [91073790](http://www.ncbi.nlm.nih.gov/entrez/query.fcgi?cmd=Search&db=Protein&term=91073790&doptcmdl=GenPept) | protease III precursor [Escherichia coli UTI89] | 67.50 | 1 | 213 |  |  |
|  | [89109604](http://www.ncbi.nlm.nih.gov/entrez/query.fcgi?cmd=Search&db=Protein&term=89109604&doptcmdl=GenPept) | protease III [Escherichia coli W3110] | 67.50 | 1 | 213 |  |  |
|  | [85675637](http://www.ncbi.nlm.nih.gov/entrez/query.fcgi?cmd=Search&db=Protein&term=85675637&doptcmdl=GenPept) | protease III [Escherichia coli W3110] | 67.50 | 1 | 213 |  |  |
|  | [83584778](http://www.ncbi.nlm.nih.gov/entrez/query.fcgi?cmd=Search&db=Protein&term=83584778&doptcmdl=GenPept) | COG1025: Secreted/periplasmic Zn-dependent peptidases, insulinase-like [Escherichia coli 101-1] [MASS=107728] | 67.50 | 1 | 213 |  |  |
| 331 | [91211807](http://www.ncbi.nlm.nih.gov/entrez/query.fcgi?cmd=Search&db=Protein&term=91211807&doptcmdl=GenPept) | hypothetical protein UTI89_C2802 [Escherichia coli UTI89] | 66.27 | 1 | 300 |  |  |
|  | [91073381](http://www.ncbi.nlm.nih.gov/entrez/query.fcgi?cmd=Search&db=Protein&term=91073381&doptcmdl=GenPept) | hypothetical protein UTI89_C2802 [Escherichia coli UTI89] | 66.27 | 1 | 300 |  |  |
|  | [89109281](http://www.ncbi.nlm.nih.gov/entrez/query.fcgi?cmd=Search&db=Protein&term=89109281&doptcmdl=GenPept) | hypothetical protein [Escherichia coli W3110] | 66.27 | 1 | 300 |  |  |
|  | [85675419](http://www.ncbi.nlm.nih.gov/entrez/query.fcgi?cmd=Search&db=Protein&term=85675419&doptcmdl=GenPept) | conserved hypothetical protein [Escherichia coli W3110] | 66.27 | 1 | 300 |  |  |
|  | [83586876](http://www.ncbi.nlm.nih.gov/entrez/query.fcgi?cmd=Search&db=Protein&term=83586876&doptcmdl=GenPept) | COG2321: Predicted metalloprotease [Escherichia coli 101-1] | 66.27 | 1 | 300 |  |  |

| **Protein IDs*, cont.*** | | | | | | | |
| --- | --- | --- | --- | --- | --- | --- | --- |
| *Grp Nr.* | *Accession Number* | *Protein Name* | *Protein Score* | *Unique PSMs* | *PSM Serial Nrs.* | *Other Grp.* | *Score (other)* |
| 332 | [91212794](http://www.ncbi.nlm.nih.gov/entrez/query.fcgi?cmd=Search&db=Protein&term=91212794&doptcmdl=GenPept) | hypothetical protein YjaG [Escherichia coli UTI89] | 65.55 | 1 | 102 |  |  |
|  | [91074368](http://www.ncbi.nlm.nih.gov/entrez/query.fcgi?cmd=Search&db=Protein&term=91074368&doptcmdl=GenPept) | hypothetical protein YjaG [Escherichia coli UTI89] [MASS=22580] | 65.55 | 1 | 102 |  |  |
|  | [89110039](http://www.ncbi.nlm.nih.gov/entrez/query.fcgi?cmd=Search&db=Protein&term=89110039&doptcmdl=GenPept) | hypothetical protein [Escherichia coli W3110] | 65.55 | 1 | 102 |  |  |
|  | [85676070](http://www.ncbi.nlm.nih.gov/entrez/query.fcgi?cmd=Search&db=Protein&term=85676070&doptcmdl=GenPept) | conserved hypothetical protein [Escherichia coli W3110] | 65.55 | 1 | 102 |  |  |
|  | [83584829](http://www.ncbi.nlm.nih.gov/entrez/query.fcgi?cmd=Search&db=Protein&term=83584829&doptcmdl=GenPept) | COG3068: Uncharacterized protein conserved in bacteria [Escherichia coli 101-1] | 65.55 | 1 | 102 |  |  |
| 333 | [94730546](http://www.ncbi.nlm.nih.gov/entrez/query.fcgi?cmd=Search&db=Protein&term=94730546&doptcmdl=GenPept) | 30S ribosomal protein S17 | 64.61 | 2 | 679 731 |  |  |
|  | [94730545](http://www.ncbi.nlm.nih.gov/entrez/query.fcgi?cmd=Search&db=Protein&term=94730545&doptcmdl=GenPept) | 30S ribosomal protein S17 | 64.61 | 2 | 679 731 |  |  |
|  | [94730544](http://www.ncbi.nlm.nih.gov/entrez/query.fcgi?cmd=Search&db=Protein&term=94730544&doptcmdl=GenPept) | 30S ribosomal protein S17 | 64.61 | 2 | 679 731 |  |  |
|  | [91212743](http://www.ncbi.nlm.nih.gov/entrez/query.fcgi?cmd=Search&db=Protein&term=91212743&doptcmdl=GenPept) | 30S ribosomal protein S17 [Escherichia coli UTI89] | 64.61 | 2 | 679 731 |  |  |
|  | [91074317](http://www.ncbi.nlm.nih.gov/entrez/query.fcgi?cmd=Search&db=Protein&term=91074317&doptcmdl=GenPept) | 30S ribosomal subunit protein S17 [Escherichia coli UTI89] | 64.61 | 2 | 679 731 |  |  |
| 334 | [91210937](http://www.ncbi.nlm.nih.gov/entrez/query.fcgi?cmd=Search&db=Protein&term=91210937&doptcmdl=GenPept) | 6-phosphofructokinase 2 [Escherichia coli UTI89] | 64.23 | 2 | 524 1640 |  |  |
|  | [91072511](http://www.ncbi.nlm.nih.gov/entrez/query.fcgi?cmd=Search&db=Protein&term=91072511&doptcmdl=GenPept) | 6-phosphofructokinase II [Escherichia coli UTI89] | 64.23 | 2 | 524 1640 |  |  |
|  | [89108562](http://www.ncbi.nlm.nih.gov/entrez/query.fcgi?cmd=Search&db=Protein&term=89108562&doptcmdl=GenPept) | 6-phosphofructokinase II [Escherichia coli W3110] | 64.23 | 2 | 524 1640 |  |  |
|  | [85675091](http://www.ncbi.nlm.nih.gov/entrez/query.fcgi?cmd=Search&db=Protein&term=85675091&doptcmdl=GenPept) | 6-phosphofructokinase II [Escherichia coli W3110] | 64.23 | 2 | 524 1640 |  |  |
|  | [83586023](http://www.ncbi.nlm.nih.gov/entrez/query.fcgi?cmd=Search&db=Protein&term=83586023&doptcmdl=GenPept) | COG1105: Fructose-1-phosphate kinase and related fructose-6-phosphate kinase (PfkB) [Escherichia coli 101-1] [MASS=32587] | 64.23 | 2 | 524 1640 |  |  |
| 335 | [91209753](http://www.ncbi.nlm.nih.gov/entrez/query.fcgi?cmd=Search&db=Protein&term=91209753&doptcmdl=GenPept) | type II citrate synthase [Escherichia coli UTI89] | 62.74 | 1 | 132 |  |  |
|  | [91071327](http://www.ncbi.nlm.nih.gov/entrez/query.fcgi?cmd=Search&db=Protein&term=91071327&doptcmdl=GenPept) | citrate synthase [Escherichia coli UTI89] | 62.74 | 1 | 132 |  |  |
|  | [89107578](http://www.ncbi.nlm.nih.gov/entrez/query.fcgi?cmd=Search&db=Protein&term=89107578&doptcmdl=GenPept) | citrate synthase [Escherichia coli W3110] | 62.74 | 1 | 132 |  |  |
|  | [85674748](http://www.ncbi.nlm.nih.gov/entrez/query.fcgi?cmd=Search&db=Protein&term=85674748&doptcmdl=GenPept) | citrate synthase [Escherichia coli W3110] | 62.74 | 1 | 132 |  |  |
|  | [83585348](http://www.ncbi.nlm.nih.gov/entrez/query.fcgi?cmd=Search&db=Protein&term=83585348&doptcmdl=GenPept) | COG0372: Citrate synthase [Escherichia coli 101-1] | 62.74 | 1 | 132 |  |  |
| 336 | [91212500](http://www.ncbi.nlm.nih.gov/entrez/query.fcgi?cmd=Search&db=Protein&term=91212500&doptcmdl=GenPept) | hypothetical protein YqjI [Escherichia coli UTI89] | 62.65 | 1 | 1672 |  |  |
|  | [91074074](http://www.ncbi.nlm.nih.gov/entrez/query.fcgi?cmd=Search&db=Protein&term=91074074&doptcmdl=GenPept) | hypothetical protein YqjI [Escherichia coli UTI89] | 62.65 | 1 | 1672 |  |  |
|  | [89109840](http://www.ncbi.nlm.nih.gov/entrez/query.fcgi?cmd=Search&db=Protein&term=89109840&doptcmdl=GenPept) | predicted transcriptional regulator [Escherichia coli W3110] | 62.65 | 1 | 1672 |  |  |
|  | [882593](http://www.ncbi.nlm.nih.gov/entrez/query.fcgi?cmd=Search&db=Protein&term=882593&doptcmdl=GenPept) | ORF_o207 | 62.65 | 1 | 1672 |  |  |
|  | [85675871](http://www.ncbi.nlm.nih.gov/entrez/query.fcgi?cmd=Search&db=Protein&term=85675871&doptcmdl=GenPept) | predicted transcriptional regulator [Escherichia coli W3110] | 62.65 | 1 | 1672 |  |  |
| 337 | [91213712](http://www.ncbi.nlm.nih.gov/entrez/query.fcgi?cmd=Search&db=Protein&term=91213712&doptcmdl=GenPept) | phosphatidylserine decarboxylase [Escherichia coli UTI89] | 62.39 | 2 | 1196 1317 |  |  |
|  | [91075286](http://www.ncbi.nlm.nih.gov/entrez/query.fcgi?cmd=Search&db=Protein&term=91075286&doptcmdl=GenPept) | phosphatidylserine decarboxylase; phospholipid synthesis [Escherichia coli UTI89] [MASS=35992] | 62.39 | 2 | 1196 1317 |  |  |
|  | [89110883](http://www.ncbi.nlm.nih.gov/entrez/query.fcgi?cmd=Search&db=Protein&term=89110883&doptcmdl=GenPept) | phosphatidylserine decarboxylase [Escherichia coli W3110] | 62.39 | 2 | 1196 1317 |  |  |
|  | [85676914](http://www.ncbi.nlm.nih.gov/entrez/query.fcgi?cmd=Search&db=Protein&term=85676914&doptcmdl=GenPept) | phosphatidylserine decarboxylase [Escherichia coli W3110] | 62.39 | 2 | 1196 1317 |  |  |
|  | [83587281](http://www.ncbi.nlm.nih.gov/entrez/query.fcgi?cmd=Search&db=Protein&term=83587281&doptcmdl=GenPept) | COG0688: Phosphatidylserine decarboxylase [Escherichia coli 101-1] | 62.39 | 2 | 1196 1317 |  |  |
| 338 | [91210618](http://www.ncbi.nlm.nih.gov/entrez/query.fcgi?cmd=Search&db=Protein&term=91210618&doptcmdl=GenPept) | thiol peroxidase [Escherichia coli UTI89] | 62.05 | 1 | 1588 |  |  |
|  | [91072192](http://www.ncbi.nlm.nih.gov/entrez/query.fcgi?cmd=Search&db=Protein&term=91072192&doptcmdl=GenPept) | thiol peroxidase [Escherichia coli UTI89] | 62.05 | 1 | 1588 |  |  |
|  | [89108171](http://www.ncbi.nlm.nih.gov/entrez/query.fcgi?cmd=Search&db=Protein&term=89108171&doptcmdl=GenPept) | lipid hydroperoxide peroxidase [Escherichia coli W3110] | 62.05 | 1 | 1588 |  |  |
|  | [85674905](http://www.ncbi.nlm.nih.gov/entrez/query.fcgi?cmd=Search&db=Protein&term=85674905&doptcmdl=GenPept) | lipid hydroperoxide peroxidase [Escherichia coli W3110] | 62.05 | 1 | 1588 |  |  |
|  | [83587465](http://www.ncbi.nlm.nih.gov/entrez/query.fcgi?cmd=Search&db=Protein&term=83587465&doptcmdl=GenPept) | COG2077: Peroxiredoxin [Escherichia coli 101-1] | 62.05 | 1 | 1588 |  |  |
| 339 | [993029](http://www.ncbi.nlm.nih.gov/entrez/query.fcgi?cmd=Search&db=Protein&term=993029&doptcmdl=GenPept) | glutaredoxin-like protein [Escherichia coli] | 60.95 | 1 | 1477 |  |  |
|  | [91210868](http://www.ncbi.nlm.nih.gov/entrez/query.fcgi?cmd=Search&db=Protein&term=91210868&doptcmdl=GenPept) | hypothetical protein UTI89_C1845 [Escherichia coli UTI89] | 60.95 | 1 | 1477 |  |  |
|  | [91072442](http://www.ncbi.nlm.nih.gov/entrez/query.fcgi?cmd=Search&db=Protein&term=91072442&doptcmdl=GenPept) | hypothetical protein UTI89_C1845 [Escherichia coli UTI89] | 60.95 | 1 | 1477 |  |  |
|  | [89108496](http://www.ncbi.nlm.nih.gov/entrez/query.fcgi?cmd=Search&db=Protein&term=89108496&doptcmdl=GenPept) | hypothetical protein [Escherichia coli W3110] | 60.95 | 1 | 1477 |  |  |
|  | [83587988](http://www.ncbi.nlm.nih.gov/entrez/query.fcgi?cmd=Search&db=Protein&term=83587988&doptcmdl=GenPept) | COG0278: Glutaredoxin-related protein [Escherichia coli 101-1] | 60.95 | 1 | 1477 |  |  |
| 340 | [91213073](http://www.ncbi.nlm.nih.gov/entrez/query.fcgi?cmd=Search&db=Protein&term=91213073&doptcmdl=GenPept) | glycyl-tRNA synthetase subunit alpha [Escherichia coli UTI89] | 60.75 | 1 | 128 |  |  |
|  | [91074647](http://www.ncbi.nlm.nih.gov/entrez/query.fcgi?cmd=Search&db=Protein&term=91074647&doptcmdl=GenPept) | glycine tRNA synthetase, alpha subunit [Escherichia coli UTI89] | 60.75 | 1 | 128 |  |  |
|  | [89110453](http://www.ncbi.nlm.nih.gov/entrez/query.fcgi?cmd=Search&db=Protein&term=89110453&doptcmdl=GenPept) | glycine tRNA synthetase, alpha subunit [Escherichia coli W3110] | 60.75 | 1 | 128 |  |  |
|  | [85676484](http://www.ncbi.nlm.nih.gov/entrez/query.fcgi?cmd=Search&db=Protein&term=85676484&doptcmdl=GenPept) | glycine tRNA synthetase, alpha subunit [Escherichia coli W3110] [MASS=34774] | 60.75 | 1 | 128 |  |  |
|  | [83588587](http://www.ncbi.nlm.nih.gov/entrez/query.fcgi?cmd=Search&db=Protein&term=83588587&doptcmdl=GenPept) | COG0752: Glycyl-tRNA synthetase, alpha subunit [Escherichia coli 101-1] | 60.75 | 1 | 128 |  |  |
| 341 | [91213156](http://www.ncbi.nlm.nih.gov/entrez/query.fcgi?cmd=Search&db=Protein&term=91213156&doptcmdl=GenPept) | bifunctional phosphopantothenoylcysteine decarboxylase/phosphopantothenate synthase [Escherichia coli UTI89] | 59.96 | 2 | 154 1029 |  |  |
|  | [91074730](http://www.ncbi.nlm.nih.gov/entrez/query.fcgi?cmd=Search&db=Protein&term=91074730&doptcmdl=GenPept) | flavoprotein affecting synthesis of DNA and pantothenate metabolism [Escherichia coli UTI89] | 59.96 | 2 | 154 1029 |  |  |
|  | [90111624](http://www.ncbi.nlm.nih.gov/entrez/query.fcgi?cmd=Search&db=Protein&term=90111624&doptcmdl=GenPept) | fused 4'-phosphopantothenoylcysteine decarboxylase/phosphopantothenoylcysteine synthetase, FMN-binding [Escherichia coli str. K-12 substr. MG1655] | 59.96 | 2 | 154 1029 |  |  |
|  | [89110372](http://www.ncbi.nlm.nih.gov/entrez/query.fcgi?cmd=Search&db=Protein&term=89110372&doptcmdl=GenPept) | fused 4'-phosphopantothenoylcysteine decarboxylase and phosphopantothenoylcysteine synthetase, FMN-binding [Escherichia coli W3110] | 59.96 | 2 | 154 1029 |  |  |
|  | [87082301](http://www.ncbi.nlm.nih.gov/entrez/query.fcgi?cmd=Search&db=Protein&term=87082301&doptcmdl=GenPept) | fused 4'-phosphopantothenoylcysteine decarboxylase/phosphopantothenoylcysteine synthetase, FMN-binding [Escherichia coli str. K-12 substr. MG1655] | 59.96 | 2 | 154 1029 |  |  |
| 342 | [91213749](http://www.ncbi.nlm.nih.gov/entrez/query.fcgi?cmd=Search&db=Protein&term=91213749&doptcmdl=GenPept) | 30S ribosomal protein S6 [Escherichia coli UTI89] | 59.74 | 1 | 355 |  |  |
|  | [91207791](http://www.ncbi.nlm.nih.gov/entrez/query.fcgi?cmd=Search&db=Protein&term=91207791&doptcmdl=GenPept) | 30S ribosomal protein S6 | 59.74 | 1 | 355 |  |  |
|  | [91207790](http://www.ncbi.nlm.nih.gov/entrez/query.fcgi?cmd=Search&db=Protein&term=91207790&doptcmdl=GenPept) | 30S ribosomal protein S6 | 59.74 | 1 | 355 |  |  |
|  | [91207789](http://www.ncbi.nlm.nih.gov/entrez/query.fcgi?cmd=Search&db=Protein&term=91207789&doptcmdl=GenPept) | 30S ribosomal protein S6 | 59.74 | 1 | 355 |  |  |
|  | [91075323](http://www.ncbi.nlm.nih.gov/entrez/query.fcgi?cmd=Search&db=Protein&term=91075323&doptcmdl=GenPept) | 30S ribosomal protein S6 [Escherichia coli UTI89] | 59.74 | 1 | 355 |  |  |
| 343 | [92090569](http://www.ncbi.nlm.nih.gov/entrez/query.fcgi?cmd=Search&db=Protein&term=92090569&doptcmdl=GenPept) | 50S ribosomal protein L19 | 59.67 | 2 | 765 772 |  |  |
|  | [91211940](http://www.ncbi.nlm.nih.gov/entrez/query.fcgi?cmd=Search&db=Protein&term=91211940&doptcmdl=GenPept) | 50S ribosomal protein L19 [Escherichia coli UTI89] | 59.67 | 2 | 765 772 |  |  |
|  | [91073514](http://www.ncbi.nlm.nih.gov/entrez/query.fcgi?cmd=Search&db=Protein&term=91073514&doptcmdl=GenPept) | 50S ribosomal subunit protein L19 [Escherichia coli UTI89] | 59.67 | 2 | 765 772 |  |  |
|  | [90109944](http://www.ncbi.nlm.nih.gov/entrez/query.fcgi?cmd=Search&db=Protein&term=90109944&doptcmdl=GenPept) | 50S ribosomal protein L19 | 59.67 | 2 | 765 772 |  |  |
|  | [90109943](http://www.ncbi.nlm.nih.gov/entrez/query.fcgi?cmd=Search&db=Protein&term=90109943&doptcmdl=GenPept) | 50S ribosomal protein L19 | 59.67 | 2 | 765 772 |  |  |
| 344 | [91213387](http://www.ncbi.nlm.nih.gov/entrez/query.fcgi?cmd=Search&db=Protein&term=91213387&doptcmdl=GenPept) | TatABCE protein translocation system subunit [Escherichia coli UTI89] | 59.39 | 1 | 253 |  |  |
|  | [91074961](http://www.ncbi.nlm.nih.gov/entrez/query.fcgi?cmd=Search&db=Protein&term=91074961&doptcmdl=GenPept) | sec-independent protein twin-arginine translocase subunit tatC [Escherichia coli UTI89] | 59.39 | 1 | 253 |  |  |
|  | [89110182](http://www.ncbi.nlm.nih.gov/entrez/query.fcgi?cmd=Search&db=Protein&term=89110182&doptcmdl=GenPept) | TatABCE protein translocation system subunit [Escherichia coli W3110] | 59.39 | 1 | 253 |  |  |
|  | [85676213](http://www.ncbi.nlm.nih.gov/entrez/query.fcgi?cmd=Search&db=Protein&term=85676213&doptcmdl=GenPept) | TatABCE protein translocation system subunit [Escherichia coli W3110] | 59.39 | 1 | 253 |  |  |
|  | [83585901](http://www.ncbi.nlm.nih.gov/entrez/query.fcgi?cmd=Search&db=Protein&term=83585901&doptcmdl=GenPept) | COG0805: Sec-independent protein secretion pathway component TatC [Escherichia coli 101-1] | 59.39 | 1 | 253 |  |  |
| 345 | [91209268](http://www.ncbi.nlm.nih.gov/entrez/query.fcgi?cmd=Search&db=Protein&term=91209268&doptcmdl=GenPept) | DL-methionine transporter substrate-binding subunit [Escherichia coli UTI89] | 59.39 | 1 | 113 |  |  |
|  | [91070842](http://www.ncbi.nlm.nih.gov/entrez/query.fcgi?cmd=Search&db=Protein&term=91070842&doptcmdl=GenPept) | D-methionine-binding transport system MetQ precursor [Escherichia coli UTI89] | 59.39 | 1 | 113 |  |  |
|  | [89107078](http://www.ncbi.nlm.nih.gov/entrez/query.fcgi?cmd=Search&db=Protein&term=89107078&doptcmdl=GenPept) | DL-methionine transporter subunit [Escherichia coli W3110] | 59.39 | 1 | 113 |  |  |
|  | [83584735](http://www.ncbi.nlm.nih.gov/entrez/query.fcgi?cmd=Search&db=Protein&term=83584735&doptcmdl=GenPept) | COG1464: ABC-type metal ion transport system, periplasmic component/surface antigen [Escherichia coli 101-1] | 59.39 | 1 | 113 |  |  |
|  | [83568974](http://www.ncbi.nlm.nih.gov/entrez/query.fcgi?cmd=Search&db=Protein&term=83568974&doptcmdl=GenPept) | COG1464: ABC-type metal ion transport system, periplasmic component/surface antigen [Shigella dysenteriae 1012] | 59.39 | 1 | 113 |  |  |
| 346 | [91209827](http://www.ncbi.nlm.nih.gov/entrez/query.fcgi?cmd=Search&db=Protein&term=91209827&doptcmdl=GenPept) | hypothetical protein UTI89_C0796 [Escherichia coli UTI89] | 59.03 | 2 | 562 744 |  |  |
|  | [91071401](http://www.ncbi.nlm.nih.gov/entrez/query.fcgi?cmd=Search&db=Protein&term=91071401&doptcmdl=GenPept) | hypothetical membrane protein YbhG [Escherichia coli UTI89] [MASS=36402] | 59.03 | 2 | 562 744 |  |  |
|  | [89107646](http://www.ncbi.nlm.nih.gov/entrez/query.fcgi?cmd=Search&db=Protein&term=89107646&doptcmdl=GenPept) | predicted membrane fusion protein (MFP) component of efflux pump, membrane anchor [Escherichia coli W3110] | 59.03 | 2 | 562 744 |  |  |
|  | [83586098](http://www.ncbi.nlm.nih.gov/entrez/query.fcgi?cmd=Search&db=Protein&term=83586098&doptcmdl=GenPept) | COG0845: Membrane-fusion protein [Escherichia coli 101-1] | 59.03 | 2 | 562 744 |  |  |
|  | [75210611](http://www.ncbi.nlm.nih.gov/entrez/query.fcgi?cmd=Search&db=Protein&term=75210611&doptcmdl=GenPept) | COG0845: Membrane-fusion protein [Escherichia coli B171] | 59.03 | 2 | 562 744 |  |  |
| 347 | [91214115](http://www.ncbi.nlm.nih.gov/entrez/query.fcgi?cmd=Search&db=Protein&term=91214115&doptcmdl=GenPept) | hypothetical protein UTI89_C5170 [Escherichia coli UTI89] | 58.73 | 1 | 1020 |  |  |
|  | [91075689](http://www.ncbi.nlm.nih.gov/entrez/query.fcgi?cmd=Search&db=Protein&term=91075689&doptcmdl=GenPept) | hypothetical protein UTI89_C5170 [Escherichia coli UTI89] | 58.73 | 1 | 1020 |  |  |
|  | [89111105](http://www.ncbi.nlm.nih.gov/entrez/query.fcgi?cmd=Search&db=Protein&term=89111105&doptcmdl=GenPept) | hypothetical protein [Escherichia coli W3110] | 58.73 | 1 | 1020 |  |  |
|  | [85677136](http://www.ncbi.nlm.nih.gov/entrez/query.fcgi?cmd=Search&db=Protein&term=85677136&doptcmdl=GenPept) | conserved hypothetical protein [Escherichia coli W3110] | 58.73 | 1 | 1020 |  |  |
|  | [83585304](http://www.ncbi.nlm.nih.gov/entrez/query.fcgi?cmd=Search&db=Protein&term=83585304&doptcmdl=GenPept) | COG3045: Uncharacterized protein conserved in bacteria [Escherichia coli 101-1] [MASS=15567] | 58.73 | 1 | 1020 |  |  |
| 348 | [91209725](http://www.ncbi.nlm.nih.gov/entrez/query.fcgi?cmd=Search&db=Protein&term=91209725&doptcmdl=GenPept) | ferric uptake regulator [Escherichia coli UTI89] | 58.57 | 1 | 970 |  |  |
|  | [91071299](http://www.ncbi.nlm.nih.gov/entrez/query.fcgi?cmd=Search&db=Protein&term=91071299&doptcmdl=GenPept) | subunit of Fur transcriptional dual regulator [Escherichia coli UTI89] | 58.57 | 1 | 970 |  |  |
|  | [89107541](http://www.ncbi.nlm.nih.gov/entrez/query.fcgi?cmd=Search&db=Protein&term=89107541&doptcmdl=GenPept) | DNA-binding transcriptional dual regulator [Escherichia coli W3110] | 58.57 | 1 | 970 |  |  |
|  | [83584543](http://www.ncbi.nlm.nih.gov/entrez/query.fcgi?cmd=Search&db=Protein&term=83584543&doptcmdl=GenPept) | COG0735: Fe2+/Zn2+ uptake regulation proteins [Escherichia coli 101-1] | 58.57 | 1 | 970 |  |  |
|  | [83569133](http://www.ncbi.nlm.nih.gov/entrez/query.fcgi?cmd=Search&db=Protein&term=83569133&doptcmdl=GenPept) | COG0735: Fe2+/Zn2+ uptake regulation proteins [Shigella dysenteriae 1012] | 58.57 | 1 | 970 |  |  |
| 349 | [91210930](http://www.ncbi.nlm.nih.gov/entrez/query.fcgi?cmd=Search&db=Protein&term=91210930&doptcmdl=GenPept) | 50S ribosomal protein L20 [Escherichia coli UTI89] | 58.16 | 2 | 732 909 |  |  |
|  | [91072504](http://www.ncbi.nlm.nih.gov/entrez/query.fcgi?cmd=Search&db=Protein&term=91072504&doptcmdl=GenPept) | 50S ribosomal subunit protein L20 [Escherichia coli UTI89] | 58.16 | 2 | 732 909 |  |  |
|  | [89108556](http://www.ncbi.nlm.nih.gov/entrez/query.fcgi?cmd=Search&db=Protein&term=89108556&doptcmdl=GenPept) | 50S ribosomal subunit protein L20 [Escherichia coli W3110] | 58.16 | 2 | 732 909 |  |  |
|  | [85675088](http://www.ncbi.nlm.nih.gov/entrez/query.fcgi?cmd=Search&db=Protein&term=85675088&doptcmdl=GenPept) | 50S ribosomal subunit protein L20 [Escherichia coli W3110] | 58.16 | 2 | 732 909 |  |  |
|  | [83754133](http://www.ncbi.nlm.nih.gov/entrez/query.fcgi?cmd=Search&db=Protein&term=83754133&doptcmdl=GenPept) | Chain Q, Crystal Structure Of The Bacterial Ribosome From Escherichia Coli At 3.5 A Resolution. This File Contains The 50s Subunit Of The Second 70s Ribosome. The Entire Crystal Structure Contains Two 70s Ribosomes And Is Described In Remark 400. | 58.16 | 2 | 732 909 |  |  |
| 350 | [91212730](http://www.ncbi.nlm.nih.gov/entrez/query.fcgi?cmd=Search&db=Protein&term=91212730&doptcmdl=GenPept) | 50S ribosomal protein L30 [Escherichia coli UTI89] | 57.75 | 1 | 1217 |  |  |
|  | [91074304](http://www.ncbi.nlm.nih.gov/entrez/query.fcgi?cmd=Search&db=Protein&term=91074304&doptcmdl=GenPept) | 50S ribosomal subunit protein L30 [Escherichia coli UTI89] | 57.75 | 1 | 1217 |  |  |
|  | [89110708](http://www.ncbi.nlm.nih.gov/entrez/query.fcgi?cmd=Search&db=Protein&term=89110708&doptcmdl=GenPept) | 50S ribosomal subunit protein L30 [Escherichia coli W3110] | 57.75 | 1 | 1217 |  |  |
|  | [85676739](http://www.ncbi.nlm.nih.gov/entrez/query.fcgi?cmd=Search&db=Protein&term=85676739&doptcmdl=GenPept) | 50S ribosomal subunit protein L30 [Escherichia coli W3110] | 57.75 | 1 | 1217 |  |  |
|  | [84028077](http://www.ncbi.nlm.nih.gov/entrez/query.fcgi?cmd=Search&db=Protein&term=84028077&doptcmdl=GenPept) | 50S ribosomal protein L30 | 57.75 | 1 | 1217 |  |  |
| 351 | [91209377](http://www.ncbi.nlm.nih.gov/entrez/query.fcgi?cmd=Search&db=Protein&term=91209377&doptcmdl=GenPept) | hypothetical protein YkgG [Escherichia coli UTI89] | 57.29 | 1 | 669 |  |  |
|  | [91070951](http://www.ncbi.nlm.nih.gov/entrez/query.fcgi?cmd=Search&db=Protein&term=91070951&doptcmdl=GenPept) | hypothetical protein YkgG [Escherichia coli UTI89] | 57.29 | 1 | 669 |  |  |
|  | [90111113](http://www.ncbi.nlm.nih.gov/entrez/query.fcgi?cmd=Search&db=Protein&term=90111113&doptcmdl=GenPept) | predicted transporter [Escherichia coli str. K-12 substr. MG1655] | 57.29 | 1 | 669 |  |  |
|  | [89107182](http://www.ncbi.nlm.nih.gov/entrez/query.fcgi?cmd=Search&db=Protein&term=89107182&doptcmdl=GenPept) | predicted transporter [Escherichia coli W3110] | 57.29 | 1 | 669 |  |  |
|  | [87081718](http://www.ncbi.nlm.nih.gov/entrez/query.fcgi?cmd=Search&db=Protein&term=87081718&doptcmdl=GenPept) | predicted transporter [Escherichia coli str. K-12 substr. MG1655] | 57.29 | 1 | 669 |  |  |
| 352 | [91209232](http://www.ncbi.nlm.nih.gov/entrez/query.fcgi?cmd=Search&db=Protein&term=91209232&doptcmdl=GenPept) | serine endoprotease [Escherichia coli UTI89] | 57.00 | 1 | 164 |  |  |
|  | [91070806](http://www.ncbi.nlm.nih.gov/entrez/query.fcgi?cmd=Search&db=Protein&term=91070806&doptcmdl=GenPept) | periplasmic serine protease DegP [Escherichia coli UTI89] | 57.00 | 1 | 164 |  |  |
|  | [89107042](http://www.ncbi.nlm.nih.gov/entrez/query.fcgi?cmd=Search&db=Protein&term=89107042&doptcmdl=GenPept) | serine endoprotease (protease Do), membrane-associated [Escherichia coli W3110] | 57.00 | 1 | 164 |  |  |
|  | [84029529](http://www.ncbi.nlm.nih.gov/entrez/query.fcgi?cmd=Search&db=Protein&term=84029529&doptcmdl=GenPept) | Protease do precursor | 57.00 | 1 | 164 |  |  |
|  | [84029528](http://www.ncbi.nlm.nih.gov/entrez/query.fcgi?cmd=Search&db=Protein&term=84029528&doptcmdl=GenPept) | Protease do precursor | 57.00 | 1 | 164 |  |  |
| 353 | [91073916](http://www.ncbi.nlm.nih.gov/entrez/query.fcgi?cmd=Search&db=Protein&term=91073916&doptcmdl=GenPept) | hypothetical protein YggL [Escherichia coli UTI89] [MASS=13994] | 57.00 | 1 | 1582 |  |  |
|  | [90111519](http://www.ncbi.nlm.nih.gov/entrez/query.fcgi?cmd=Search&db=Protein&term=90111519&doptcmdl=GenPept) | predicted protein [Escherichia coli str. K-12 substr. MG1655] | 57.00 | 1 | 1582 |  |  |
|  | [89109736](http://www.ncbi.nlm.nih.gov/entrez/query.fcgi?cmd=Search&db=Protein&term=89109736&doptcmdl=GenPept) | hypothetical protein [Escherichia coli W3110] | 57.00 | 1 | 1582 |  |  |
|  | [87082190](http://www.ncbi.nlm.nih.gov/entrez/query.fcgi?cmd=Search&db=Protein&term=87082190&doptcmdl=GenPept) | predicted protein [Escherichia coli str. K-12 substr. MG1655] | 57.00 | 1 | 1582 |  |  |
|  | [85675769](http://www.ncbi.nlm.nih.gov/entrez/query.fcgi?cmd=Search&db=Protein&term=85675769&doptcmdl=GenPept) | hypothetical protein [Escherichia coli W3110] | 57.00 | 1 | 1582 |  |  |
| 354 | [91210040](http://www.ncbi.nlm.nih.gov/entrez/query.fcgi?cmd=Search&db=Protein&term=91210040&doptcmdl=GenPept) | dihydroorotate dehydrogenase 2 [Escherichia coli UTI89] | 55.56 | 1 | 106 |  |  |
[truncated: 309,052 more chars]
